# Supplementary material for: A Bischler-Napieralski and homo-Mannich sequence enables diversified syntheses of sarpagine alkaloids and analogues
Source: Nat Commun. 2023 Sep 9;14:5560. doi: 10.1038/s41467-023-41268-9 (PMC10492809; doi:10.1038/s41467-023-41268-9)
Supplement: Supplementary file 1 — Supplementary Information [file 41467_2023_41268_MOESM1_ESM.pdf]

Supplementary Information

A Bischler-Napieralski and Homo-Mannich Sequence Enables Diversified Syntheses of Sarpagine Alkaloids and Analogues

Hanyue Qiu[1]†, Xinghai Fei[1]†, Jiaojiao Yang[1]†, Zhen Qiao[1]†, Shan Yuan[1]†, Hu Zhang[1], Ling He[1], and Min Zhang[1]*

[1]Chongqing Key Laboratory of Natural Product Synthesis and Drug Research, Innovative Drug Research Center, School of Pharmaceutical Sciences, Chongqing University, Chongqing 401331, China.

†These authors contributed equally: Hanyue Qiu, Xinghai Fei, Jiaojiao Yang, Zhen Qiao, Shan Yuan.

*e-mail: minzhang@cqu.edu.cn

Table of Contents **Page**

1. General Information ... **S1**

2. Experimental procedures and characterization data ... **S2**

2.1. **Supplementary Table 1** Optimization of the Bischler-Napieralski reaction of **1** ... **S2**

2.2. **Supplementary Table 2** Optimization of the homo-Mannich reaction of **2** ... **S2**

2.3. General preparation of **11** ... **S3**

2.4. General preparation of **1** ... **S5**

2.5. General preparation of **2a** and **2e-2l** ... **S9**

2.6. Preparation of **2b** ... **S12**

2.7. Preparation of **2c** ... **S13**

2.8. Preparation of **2d** ... **S14**

2.9. General preparation of **3** ... **S14**

2.10. General preparation of **13** ... **S19**

2.11. General preparation of **14** ... **S22**

2.12. General procedure for preparation of **14-1** ... **S26**

2.13. General procedure for preparation of **15a**, **15ae**, **15af**, **15ag**, **15ai**, and **15aj** ... **S28**

2.14. Preparation of **15ad** ... **S31**

2.15. Preparation of **15al** ... **S32**

2.16. Preparation of **15am** ... **S32**

2.17. Preparation of **15an** ... **S33**

2.18. Preparation of **15ao** ... **S34**

2.19. Preparation of **15ap** ... **S34**

2.20. Preparation of **15aq** ... **S35**

2.21. Preparation of **15ar** ... **S36**

2.22. Preparation of (+)-vellosimine (**4**) ... **S36**

2.23. Preparation of **14aa** ... **S37**

2.24. Preparation of **15aa** ... **S37**

2.25. Preparation of (+)-N_a-methylvellosimine (**5**) ... **S38**

2.26. **Supplementary Table 3** ^{1}H NMR spectroscopic data comparison of (+)-N_a-methylvellosimine (**5**) ... **S38**

2.27. **Supplementary Table 4** ^{13}C NMR spectroscopic data comparison of (+)-N_a-methylvellosimine (**5**) ... **S39**

2.28. Preparation of **15l** ... **S40**

2.29. Preparation of (+)-10-methoxyvellosimine (**6**) **S40**

2.30. **Supplementary Table 5** ^{1}H NMR spectroscopic data comparison of (+)-10-methoxyvellosimine (**6**) **S41**

2.31. **Supplementary Table 6** ^{13}C NMR spectroscopic data comparison of (+)-10-methoxyvellosimine (**6**) **S41**

2.32. Preparation of **16** **S42**

2.33. Preparation of **17** **S43**

2.34. Preparation of (–)-alkaloid Q3 (**7**) **S43**

2.35. **Supplementary Table 7** ^{1}H NMR spectroscopic data comparison of (–)-alkaloid Q3 (**7**) **S44**

2.36. **Supplementary Table 8** ^{13}C NMR spectroscopic data comparison of (–)-alkaloid Q3 (**7**) **S44**

2.37. Preparation of **15as** **S45**

2.38. Preparation of **18** **S46**

2.39. Preparation of **19** **S46**

2.40. Preparation of **20** **S47**

2.41. Preparation of **20-1** **S48**

2.42. Preparation of (+)-polyneuridine (**8**) **S48**

2.43. **Supplementary Table 9** ^{1}H NMR spectroscopic data comparison of (+)-polyneuridine (**8**) **S49**

2.44. **Supplementary Table 10** ^{13}C NMR spectroscopic data comparison of (+)-polyneuridine (**8**) **S49**

2.45. Preparation of (–)-macusine A (**9**) **S50**

2.46. **Supplementary Table 11** ^{1}H NMR spectroscopic data comparison of (–)-macusine A (**9**) **S51**

2.47. **Supplementary Table 12** ^{13}C NMR spectroscopic data comparison of (–)-macusine A (**9**) **S51**

2.48. Preparation of **21** **S52**

2.49. Preparation of (+)-dehydrovoachalotine (**10**) **S52**

2.50. **Supplementary Table 13** ^{1}H NMR spectroscopic data comparison of (+)-dehydrovoachalotine (**10**) **S53**

2.51. **Supplementary Table 14** ^{13}C NMR spectroscopic data comparison of (+)-dehydrovoachalotine (**10**) **S54**

3. Supplementary Figures **S54**

4. NMR spectra **S55**

NMR spectra of **1a** **S56**

NMR spectra of **1e** **S58**

NMR spectra of **1f** **S60**

NMR spectra of **1g** **S62**

NMR spectra of **1h** **S64**

NMR spectra of **1i** **S66**

NMR spectra of **1j** **S68**

NMR spectra of **1k** **S70**

NMR spectra of **2a** **S72**

NMR spectra of **2a** **S74**

NMR spectra of **2b** **S76**

NMR spectra of **2c** **S78**

NMR spectra of **2d** **S80**

NMR spectra of **2e** **S82**

NMR spectra of **2f** **S84**

NMR spectra of **2g** **S86**

NMR spectra of **2h** **S88**

NMR spectra of **2i** **S90**

NMR spectra of **2j** **S92**

NMR spectra of **2k** **S94**

NMR spectra of **2l** **S96**

NMR spectra of **3a** **S98**

NMR spectra of **3b** **S100**

NMR spectra of **3c** **S102**

NMR spectra of **3d** **S104**

NMR spectra of **3e** **S106**

NMR spectra of **3f** **S108**

NMR spectra of **3g** **S110**

NMR spectra of **3h** **S112**

NMR spectra of **3i** **S114**

NMR spectra of **3j** **S116**

NMR spectra of **3k** **S118**

NMR spectra of **3l** **S120**

NMR spectra of **13a** **S122**

NMR spectra of **13e** **S124**

NMR spectra of **13f** **S126**

NMR spectra of **13g** **S128**

NMR spectra of **13i** **S130**
NMR spectra of **13j** **S132**
NMR spectra of **13l** **S134**
NMR spectra of **14a** **S136**
NMR spectra of **14e** **S138**
NMR spectra of **14f** **S140**
NMR spectra of **14g** **S142**
NMR spectra of **14i** **S144**
NMR spectra of **14j** **S146**
NMR spectra of **14l** **S148**
NMR spectra of **14-1e** **S150**
NMR spectra of **14-1f** **S152**
NMR spectra of **14-1g** **S154**
NMR spectra of **14-1i** **S156**
NMR spectra of **14-1j** **S158**
NMR spectra of **15ad** **S160**
NMR spectra of **15ae** **S162**
NMR spectra of **15af** **S164**
NMR spectra of **15ag** **S166**
NMR spectra of **15ai** **S168**
NMR spectra of **15aj** **S170**
NMR spectra of **15al** **S172**
NMR spectra of **15am** **S174**
NMR spectra of **15an** **S176**
NMR spectra of **15ao** **S178**
NMR spectra of **15ap** **S180**
NMR spectra of **15aq** **S182**
NMR spectra of **15ar** **S184**
NMR spectra of **14aa** **S186**
NMR spectra of **15aa** **S188**
NMR spectra of (+)-N_a-methylvellosimine (**5**) **S190**

NMR spectra of **15l** **S192**

NMR spectra of (+)-10-methoxyvellosimine (**6**) **S194**

NMR spectra of **16** **S196**

NMR spectra of **17** **S198**

NMR spectra of (–)-alkaloid Q3 (**7**) **S200**

NMR spectra of **15as** **S202**

NMR spectra of **18** **S204**

NMR spectra of **19** **S206**

NMR spectra of **20** **S208**

NMR spectra of **20-1** **S210**

NMR spectra of (+)-polyneuridine (**8**) **S212**

NMR spectra of (–)-macusine A (**9**) **S214**

NMR spectra of **21** **S216**

NMR spectra of (+)-dehydrovoachalotine (**10**) **S218**

5. References **S220**

1. General Information

All reagents were obtained from Adamas, Accela, or Acros and were used without further purification unless otherwise noted. All reactions were carried out under a positive pressure of inert gas in the oven-dried glassware with magnetic stirring. Unless otherwise stated, all solvents employed in the reactions were distilled from appropriate drying agents prior to use. ^1H NMR spectra were obtained on an Agilent 400MR or 600MR DD2 spectrometer at ambient temperature. Data were reported as follows: chemical shift on the δ scale using residual proton solvent as internal standard [δ 7.26 ($CDCl_3$); 2.50 (DMSO-d_6); 3.31 (CD_3OD); TMS: 0.00 ppm], multiplicity (s = singlet, d = doublet, t = triplet, q = quartet, m = multiplet, dd = doublet of doublets, brs = broad singlet), integration, and coupling constant (J) in hertz (Hz). ^{13}C NMR spectra were obtained with proton decoupling on an Agilent 400MR (400 MHz) or 600MR DD2 spectrometer and were reported in ppm with residual solvent for the internal standard. IR spectra were recorded on a Bruker 100 FT–IR spectrometer and were reported in terms of frequency of absorption (cm^{-1}). ESI-HRMS was performed on Bruker Solari X 7.0 T spectrometer. Melting points were recorded on an SGW X–4A apparatus. Optical rotations were measured with a Rudolph polarimeter.

2. Experimental Procedures and Characterization Data

2.1 Supplementary Table 1 Optimization of the Bischler-Napieralski reaction of 1a[a]

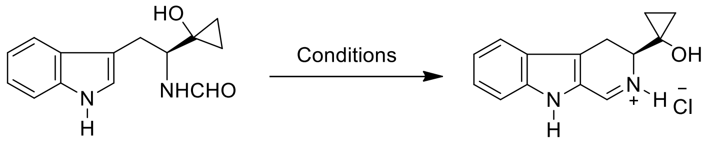

1a **2a**

Entry	Amide activator	Base	Solvent	Yield (%)[b]
1[c]	Tf_2O	2-ClPyr	CH_2Cl_2	28
2[c]	Tf_2O	DTBMP	CH_2Cl_2	trace
3	$POCl_3$	–	CH_2Cl_2	45
4	P_2O_5	–	CH_2Cl_2	5
5	$POCl_3$	–	Et_2O	trace
6	$POCl_3$	–	2-Me-THF	trace
7	$POCl_3$	–	1,4-dioxane	10
8	$POCl_3$	–	THF	60
9	$POCl_3$	–	DME	5
10	$POCl_3$	–	CH_3CN	0
11	$POCl_3$	–	^{t}BuOH	0
12	$POCl_3$	–	THF	60
13[d]	$POCl_3$	–	THF	64
14[e]	$POCl_3$	–	THF	69
15[f]	$POCl_3$	–	THF	63

[a]Reaction conditions: **1a** (0.2 mmol), amide activator (1 mmol), base (0.24 mmol) in solvent (2 mL) under an atmosphere of argon (balloon) at room temperature. [b]Isolated yield of **2a**. [c]The reaction was carried out at –78 °C. [d]THF (0.4 mL) was used. [e]THF (0.2 mL) was used. [f]THF (0.13 mL) was used.

2.2 Supplementary Table 2 Optimization of the homo-Mannich reaction of 2a[a]

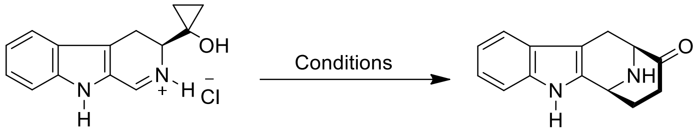

2a **3a**

Entry	[M]	Solvent	Time	Yield (%)[b]
1	$CuCl_2$	THF	48 h	0
2	CuO	THF	48 h	0
3	$Cu(OAc)_2$	THF	48 h	0
4	$Cu(acac)_2$	THF	48 h	0
5	$Cu(OTf)_2$	THF	48 h	0
6	$Mn(dpm)_3$	THF	2 h	30
7	$Mn(acac)_3$	THF	5 h	10
8	$Mn(OAc)_3$	THF	48 h	0
9	$Sc(OTf)_3$	THF	48 h	0
10	$Zn(OTf)_2$	THF	48 h	0
11	$Fe(NO_3)_3$	THF	24 h	0
12	$Fe_2(SO_4)_3$	THF	48 h	0
13	$Fe(acac)_3$	THF	48 h	0

14	$ZnCl_2$	THF	48 h	0
15	$AgNO_3$	THF	48 h	0
16	$Fe(OTf)_3$	THF	15 min	64
17[c]	$Fe(OTf)_3$	THF	30 min	40
18[d]	$Fe(OTf)_3$	THF	30 min	0
19[e]	$Fe(OTf)_3$	THF	1 h	20
20[f]	$Fe(OTf)_3$	THF	25 min	55
21	$Fe(OTf)_3$	2-Me-THF	20 min	trace
22	$Fe(OTf)_3$	Et_2O	20 min	0
23	$Fe(OTf)_3$	1,4-dioxane	10 min	68
24	$Fe(OTf)_3$	DME	20 min	0
25	$Fe(OTf)_3$	CH_2Cl_2	20 min	0
26	$Fe(OTf)_3$	toluene	20 min	0
27	$Fe(OTf)_3$	MeCN	20 min	0
28	$Fe(OTf)_3$	DMF	20 min	0
29	$Fe(OTf)_3$	DMSO	20 min	0

[a]Reaction conditions: **2a** (0.2 mmol) and [M] (0.4 mmol) in solvent (2 mL) under an atmosphere of argon (balloon) at room temperature. [b]Isolated yield of **3a**. [c]$NaHCO_3$ (0.4 mmol) was used. [d]4 Å·MS (200 mg) was used. [e]$Fe(OTf)_3$ (0.2 mmol) was used. [f]$Fe(OTf)_3$ (0.6 mmol) was used.

2.3 General procedure for the preparation of 11

Compounds **11** were prepared with **method 1** and **method 2** by following the published procedures.[1-3]

method 1:

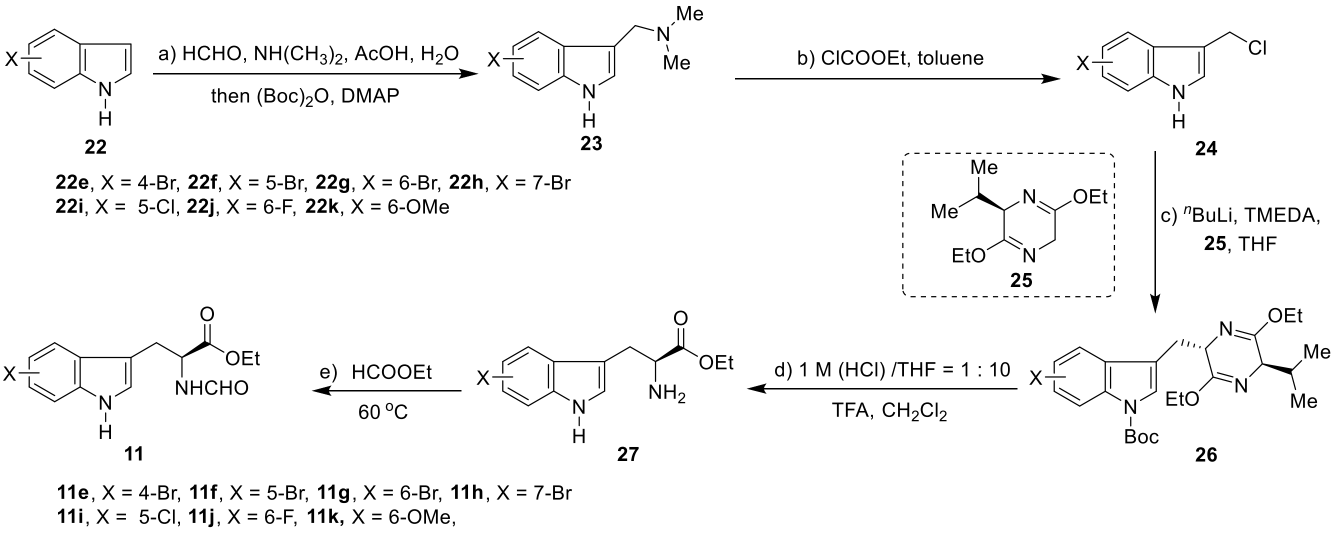

method 2:

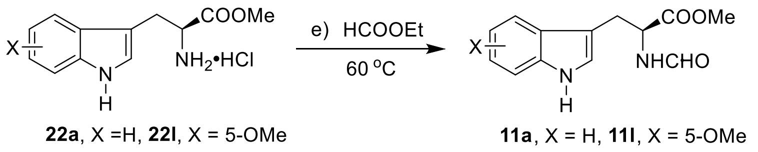

Method 1: To a solution of formaldehyde (1.1 equiv) in AcOH (0.65 M), 1,4-dioxane (0.65 M), and H_2O (0.048 M) was added dimethylamine (1.1 equiv) at 0 °C. Afterward, a solution of indoles **22** (1 equiv) in 1,4-dioxane (0.65 M) was added dropwise. The reaction mixture was warmed to room temperature and stirred for 6 h.The solution

was then cooled to 0 °C, and an aqueous solution of NaOH (4 M) was added to the mixture until the pH value reached 12. The resulting solution was extracted with EtOAc. The organic phase was washed with brine, dried over Na_2SO_4, filtered, and concentrated in vacuo. The crude product was used directly in the next step without further purification.

To a solution of the above crude amine in THF (0.5 M) were added $(Boc)_2O$ (1.2 equiv), DMAP (0.1 equiv), and TEA (0.07 equiv) at room temperature. The mixture was stirred for 1 h before a saturated aqueous solution of NH_4Cl was added to quench the reaction. The resulting mixture was extracted with EtOAc. The combined organic phase was washed with brine, dried over Na_2SO_4, filtered, and concentrated under reduced pressure. The crude product was purified by flash column chromatography on silica gel (petroleum ether/EtOAc) to afford **23**.

To a solution of **23** (1 equiv) in dry toluene (0.2 M) was added ethyl chloroformate (1.2 equiv) under argon. The resulting mixture was stirred at room temperature for 20 h, and concentrated in vacuo to afford crude product **24**.

To a solution of chiral auxiliary **25** (1 equiv) in THF (0.15 M) was added ^{n}BuLi (1 equiv) dropwise at –78 °C. The reaction mixture was stirred at –78 °C for 30 min and then TMEDA (1.3 equiv) was added. The reaction mixture was stirred at –78 °C for an additional 30 min, and a solution of **24** (1 equiv) in THF was added dropwise over 10 min. The mixture was stirred for an additional 12 h before a saturated aqueous solution of NH_4Cl was added to quench the reaction. The resulting mixture was extracted with EtOAc and washed with brine. The combined organic phase was dried over Na_2SO_4, filtered, and concentrated in vacuo. The crude product was purified by flash chromatography on silica gel (petroleum ether/EtOAc) to afford **26**.

To a solution of **26** (1 equiv) in THF (0.2 M) was added an aqueous solution of HCl (2 M, 2.5 equiv) at 0 °C. The mixture was warmed to room temperature and stirred for an additional 12 h before a saturated aqueous solution of $NaHCO_3$ was added to quench the reaction. The resulting mixture was extracted with EtOAc and washed with brine. The combined organic phase was dried over Na_2SO_4, filtered, and concentrated in vacuo. The crude product was used directly in the next step without further purification.

To a solution of the above crude ester in CH_2Cl_2 (0.1 M) was added TFA (0.1 M) at 0 °C. The mixture was refluxed for 4 h, cooled to room temperature, and concentrated under reduced pressure. The solution was then cooled to 0 °C and an aqueous solution of NaOH (1 M) was added to the mixture until the pH value reached 12. The resulting solution was extracted with EtOAc. The organic phase was washed with brine, dried over Na_2SO_4, and filtered. The mixture was concentrated in vacuo to afford crude product **27**.

A mixture of **27** (1 equiv) and ethyl formate (0.4 M) was refluxed for 8 h. The mixture was then cooled to room temperature, and concentrated in vacuo. The crude product was purified by flash chromatography on silica gel (petroleum ether/EtOAc) to afford **11e-11k**.

Method 2: A mixture of **22a** or **22l** (1 equiv) and ethyl formate (0.4 M) was refluxed for 8 h. The mixture was then cooled to room temperature, and concentrated in vacuo. The crude product was purified by flash chromatography on silica gel (petroleum ether/EtOAc) to afford **11a** or **11k**.

2.4 General procedure for the preparation of 1

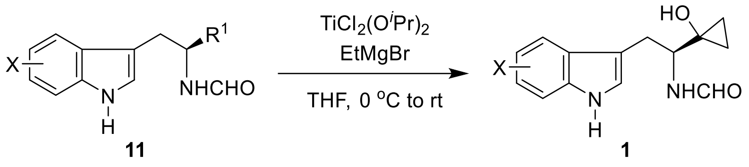

R^1 = COOEt or COOMe

To a solution of $TiCl_4$ (1.1 mL, 10 mmol) in dry THF (10 mL) under an atmosphere of argon was added $Ti(O^iPr)_4$ (2.8 mL, 10 mmol) dropwise at 0 °C. The mixture was stirred for 30 min at room temperature to obtain 1 M solution of $TiCl_2(O^iPr)_2$.

To a solution of **11** (0.1 mmol) in dry THF (1 mL) was added freshly prepared $TiCl_2(O^iPr)_2$ solution (1 M in THF, 0.1 mL) under an atmosphere of argon at –78 °C. The reaction mixture was added with EtMgBr (2 M in THF, 0.3 mL, 0.6 mmol) dropwise, and was kept stirring at room temperature. After **11** was fully consumed as judged by TLC analysis, a saturated aqueous solution of NH_4Cl (5 mL) was added to quench the reaction. The resulting mixture was filtered through a pad of Celite, and extracted with EtOAc (3 × 10 mL). The combined organic phase was washed with a saturated aqueous solution of NaCl (3 × 10 mL), dried over Na_2SO_4, filtered, and concentrated under reduced pressure. The residue was purified by flash column chromatography on silica gel to afford the pure product **1**.

(*S*)-*N*-(1-(1-hydroxycyclopropyl)-2-(1*H*-indol-3-yl)ethyl)formamide (1a)

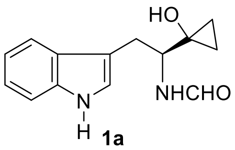

1a

Purification by column chromatography on silica gel (petroleum ether/EtOAc = 2/1, R_f = 0.20) afforded **1a** (15.8 mg, 65%) as a white solid.

^{1}H NMR (400 MHz, DMSO-d_6): δ 10.73 (s, 1H), 7.98 (d, J = 15.3 Hz, 2H), 7.56 (t, J = 10.3 Hz, 1H), 7.33 (d, J = 7.8 Hz, 1H), 7.15–7.03 (m, 2H), 6.98 (t, J = 7.2 Hz, 1H), 5.47 (s, 1H), 3.85 (td, J = 9.5, 4.2 Hz, 1H), 3.22–3.12 (m, 1H), 2.89 (dd, J = 14.7, 9.9 Hz, 1H), 0.64–0.51 (m, 3H), 0.47–0.39 (m, 1H) ppm. **^{13}C NMR** (100 MHz, DMSO-d_6): δ 160.8, 136.2, 127.5, 123.0, 120.8, 118.3, 118.2, 111.7, 111.6, 111.3, 56.7, 53.7, 27.0, 12.1, 11.2 ppm. **IR** (KBr): ν_{max} = 3303, 2923, 1668, 1457, 1388, 1231, 1014, 744, 426 cm^{-1}. **HRMS**(m/z): Calcd for $C_{14}H_{16}N_2NaO_2$, $[M+Na]^+$, 267.1104; found 267.1095. **m.p.**: 153.7–154.8 ℃. **$[\alpha]_D^{25}$**: –160.8 (*c* 0.26, MeOH).

(*S*)-*N*-(2-(4-bromo-1*H*-indol-3-yl)-1-(1-hydroxycyclopropyl)ethyl)formamide (1e)

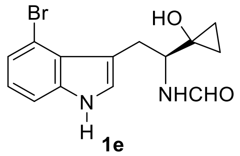

1e

Purification by column chromatography on silica gel (petroleum ether/EtOAc = 3/1, R_f = 0.20) afforded **1e** (19.3 mg, 60%) as a white solid.

^{1}H NMR (400 MHz, DMSO-d_6): δ 10.96 (s, 1H), 7.97 (d, J = 8.9 Hz, 1H), 7.92 (s, 1H), 7.76–7.68 (m, 1H), 7.29 (d, J = 8.5 Hz, 1H), 7.21–7.10 (m, 2H), 5.48–5.41 (m, 1H), 3.80 (td, J = 9.5, 4.4 Hz, 1H)., 3.20–3.05 (m, 1H), 2.87–2.76 (m, 1H), 0.62–0.47 (m, 3H), 0.43–0.35 (m, 1H) ppm. **^{13}C NMR** (100 MHz, DMSO-d_6): δ 160.8, 136.1, 127.4, 123.0, 120.8, 118.3, 118.1, 111.6, 111.3, 56.7, 53.6, 27.0, 12.1, 11.1 ppm. **IR** (KBr): v_{max} = 3296, 2922, 1668, 1456, 1378, 1229, 1013, 744 cm^{-1}. **HRMS**(m/z): Calcd for $C_{14}H_{14}BrN_2O_2$, $[M–H]^-$, 321.0244; found 321.0229. **m.p.**: 174.9–176.0 °C. **$[\alpha]_D^{25}$**: –88.0 (*c* 0.3, MeOH).

(*S*)-*N*-(2-(5-bromo-1*H*-indol-3-yl)-1-(1-hydroxycyclopropyl)ethyl)formamide (1f)

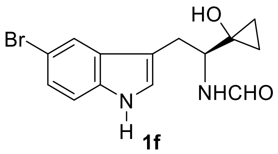

1f

Purification by column chromatography on silica gel (petroleum ether/EtOAc = 3/1, R_f = 0.30) afforded **1f** (16.4 mg, 51%) as a white solid.

^{1}H NMR (400 MHz, DMSO-d_6): δ 10.96 (s, 1H), 7.97 (d, J = 8.6 Hz, 1H), 7.92 (s, 1H), 7.77–7.69 (m, 1H), 7.29 (d, J = 8.3 Hz, 1H), 7.21–7.10 (m, 2H), 5.46 (s, 1H), 3.80 (td, J = 9.6, 4.4 Hz, 1H), 3.20–3.06 (m, 1H), 2.88–2.75 (m, 1H), 0.58–0.46 (m, 3H), 0.42–0.35 (m, 1H) ppm. **^{13}C NMR** (100 MHz, DMSO-d_6): δ 160.8, 134.8, 129.3, 124.9, 123.1, 120.6, 113.3, 111.6, 110.8, 56.4, 53.6, 26.6, 12.0, 10.8 ppm. **IR** (KBr): v_{max} = 3295, 2915, 1667, 1458, 1227, 948, 766 cm^{-1}. **HRMS**(m/z): Calcd for $C_{14}H_{15}BrN_2NaO_2$, $[M+Na]^+$, 345.0209; found 345.0199. **m.p.**: 164.4–166.2 ℃. **$[\alpha]_D^{25}$**: –57.8 (*c* 0.14, MeOH).

(*S*)-*N*-(2-(6-bromo-1*H*-indol-3-yl)-1-(1-hydroxycyclopropyl)ethyl)formamide (1g)

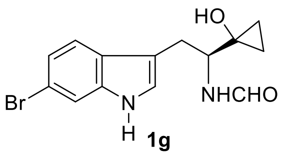

1g

Purification by column chromatography on silica gel (petroleum ether/EtOAc = 3/1, R_f = 0.30) afforded **1g** (17.4 mg, 54%) as a white solid.

^{1}H NMR (400 MHz, CD_3OD): δ 7.98 (s, 1H), 7.51–7.46 (m, 2H), 7.13–7.06 (m, 2H), 3.89 (dd, J = 9.5, 5.1 Hz, 1H), 3.23 (dd, J = 14.8, 5.1 Hz, 1H), 3.01 (dd, J = 14.9, 9.5 Hz, 1H), 0.69 – 0.60 (m, 3H), 0.53–0.46 (m, 1H) ppm.

^{13}C NMR (100 MHz, CD_3OD): δ 163.4, 138.8, 127.9, 125.6, 124.8, 122.6, 120.8, 120.6, 115.6, 115.0, 113.1, 61.5, 57.9, 56.2, 28.0, 13.3, 12.5 ppm. **IR** (KBr): ν_{max} = 3297, 2932, 1667, 1549, 1216, 1017, 801 cm^{-1}. **HRMS** (m/z): Calcd for $C_{14}H_{14}BrN_2O_2$, $[M–H]^-$, 321.0228; found 321.0244. **m. p.**: 184.9–186.0 ℃. **$[\alpha]_D^{25}$**: –42.8 (*c* 0.12, MeOH).

(*S*)-*N*-(2-(7-bromo-1*H*-indol-3-yl)-1-(1-hydroxycyclopropyl)ethyl)formamide (1h)

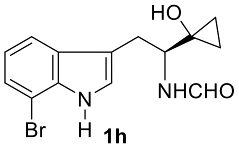

1h

Purification by column chromatography on silica gel (CH_2Cl_2/MeOH = 10/1, R_f = 0.40) afforded **1h** (16.1 mg, 50%) as a white solid.

^{1}H NMR (600 MHz, DMSO-d_6): δ 10.97 (s, 1H), 7.98 (d, *J* = 9.3 Hz, 1H), 7.92 (d, *J* = 2.1 Hz, 1H), 7.57 (d, *J* = 7.8 Hz, 1H), 7.27 (d, *J* = 7.3 Hz, 1H), 7.17 (d, *J* = 2.4 Hz, 1H), 6.93 (td, *J* = 7.7, 1.8 Hz, 1H), 5.47 (s, 1H), 3.80 (td, *J* = 9.7, 4.1 Hz, 1H), 3.18–3.09 (m, 1H), 2.85 (dd, *J* = 14.8, 10.1 Hz, 1H), 0.58–0.46 (m, 3H), 0.44–0.36 (m, 1H) ppm. **^{13}C NMR** (150 MHz, DMSO-d_6): δ 160.7, 134.3, 129.2, 124.4, 123.3, 119.6, 117.9, 113.1, 104.1, 56.6, 53.5, 26.9, 12.1, 11.1 ppm. **IR** (KBr): ν_{max} = 3438, 2917, 1662, 1553, 1489, 1016, 734 cm^{-1}. **HRMS** (m/z): Calcd for $C_{14}H_{14}BrN_2O_2$, $[M–H]^-$, 321.0229; found 321.0244. **m. p.**: 185.0–185.7 ℃. **$[\alpha]_D^{25}$**: –32.7 (*c* 0.16, MeOH).

(*S*)-*N*-(2-(5-chloro-1*H*-indol-3-yl)-1-(1-hydroxycyclopropyl)ethyl)formamide (1i)

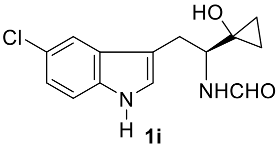

1i

Purification by column chromatography on silica gel (petroleum ether/EtOAc = 2/1, R_f = 0.10) afforded **1i** (19.7 mg, 71%) as a white solid.

^{1}H NMR (400 MHz, DMSO-d_6): δ 10.99 (s, 1H), 8.02 (d, *J* = 9.0 Hz, 1H), 7.97 (s, 1H), 7.64 (s, 1H), 7.37 (d, *J* = 8.6 Hz, 1H), 7.23 (s, 1H), 7.08 (dd, *J* = 8.5, 2.2 Hz, 1H), 5.51 (s, 1H), 3.85 (td, *J* = 9.4, 4.7 Hz, 1H), 3.18 (dd, *J* = 14.8, 4.5 Hz, 1H), 2.86 (dd, *J* = 14.8, 9.6 Hz, 1H), 0.66–0.51 (m, 3H), 0.48–0.42 (m, 1H) ppm. **^{13}C NMR** (100 MHz, DMSO-d_6): δ 160.8, 134.6, 128.7, 125.1, 123.0, 120.7, 117.7, 112.9, 111.7., 56.6, 53.7, 26.8, 12.1, 11.0 ppm. **IR** (KBr): ν_{max} = 3437, 2916, 1667, 1460, 1223, 1099, 797 cm^{-1}. **HRMS** (m/z): Calcd for $C_{14}H_{14}ClN_2O_2$, $[M–H]^-$, 277.0737; found 277.0749. **m. p.**: 156.4–158.0 °C. **$[\alpha]_D^{25}$**: –22.0 (*c* 0.5, MeOH).

(*S*)-*N*-(2-(6-fluoro-1*H*-indol-3-yl)-1-(1-hydroxycyclopropyl)ethyl)formamide (1j)

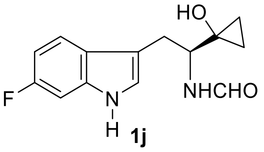

1j

Purification by column chromatography on silica gel (petroleum ether/EtOAc = 2/1, R_f = 0.20) afforded **1j** (17.5 mg, 68%) as a white solid.

^{1}H NMR (400 MHz, DMSO-d_6): δ 10.80 (s, 1H), 7.98 (d, J = 8.9 Hz, 1H), 7.93 (s, 1H), 7.57–7.47 (m, 1H), 7.14–7.05 (m, 2H), 6.82 (t, J = 8.6 Hz, 1H), 5.45 (s, 1H), 3.81 (td, J = 9.4, 4.2 Hz, 1H), 3.20–3.08 (m, 1H), 2.85 (dd, J = 14.7, 9.9 Hz, 1H), 0.62–0.47 (m, 3H), 0.44–0.35 (m, 1H) ppm. **^{13}C NMR** (100 MHz, DMSO-d_6): δ 160.8, 158.9 (J_{C-F} = 232.0 Hz), 136.0 (J_{C-F} = 12.8 Hz), 124.4, 123.6 (J_{C-F} = 2.2 Hz), 119.4 (J_{C-F} = 10.0 Hz), 112.0, 106.6 (J_{C-F} = 24.1 Hz), 97.3 (J_{C-F} = 25.4 Hz), 56.7, 53.7, 27.0, 12.2, 11.2 ppm. **IR** (KBr): ν_{max} = 3437, 2921, 1669, 1456, 1342, 1017, 803 cm^{-1}. **HRMS** (m/z): Calcd for $C_{14}H_{14}FN_2O_2$, $[M–H]^-$, 261.1035; found 261.1044. **m. p.**: 169.2–171.0 ℃. **$[\alpha]_D^{25}$**: –31.0 (c 0.6, MeOH).

(*S*)-*N*-(2-(6-bromo-1*H*-indol-3-yl)-1-(1-hydroxycyclopropyl)ethyl)formamide (1k)

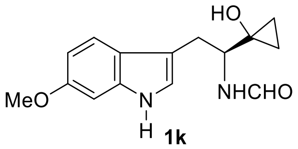

1k

Purification by column chromatography on silica gel (CH_2Cl_2/MeOH = 10/1, R_f = 0.50) afforded **1k** (16.4 mg, 60%) as a white solid.

^{1}H NMR (400 MHz, DMSO-d_6): δ 10.52 (s, 1H), 7.96 (d, J = 9.0 Hz, 1H), 7.92 (s, 1H), 7.40 (d, J = 8.7 Hz, 1H), 6.99–6.91 (m, 1H), 6.82 (t, J = 3.6 Hz, 1H), 6.62 (dd, J = 8.6, 2.3 Hz, 1H), 5.43 (d, J = 5.1 Hz, 1H), 3.80 (dt, J = 9.5, 4.8 Hz, 1H), 3.74 (s, 3H), 3.10 (dd, J = 14.9, 3.8 Hz, 1H), 2.80 (dd, J = 14.9, 9.8 Hz, 1H), 0.58–0.47 (m, 3H), 0.44–0.35 (m, 1H) ppm. **^{13}C NMR** (100 MHz, DMSO-d_6): δ 160.8, 152.9, 131.3, 127.8, 123.6, 111.9, 111.5, 110.8, 100.3, 56.7, 55.3, 53.6, 27.0, 12.1, 11.1 ppm. **IR** (KBr): ν_{max} = 3328, 2941, 1670, 1484, 1214, 1064, 831 cm^{-1}. **HRMS** (m/z): Calcd for $C_{15}H_{19}N_2NaO_3$, $[M+Na]^+$, 297.1211; found 297.1209. **m. p.**: 147.2–148.3 ℃. **$[\alpha]_D^{25}$**: –47.8 (c 0.1, MeOH).

(*S*)-*N*-(1-(1-hydroxycyclopropyl)-2-(5-methoxy-1*H*-indol-3-yl)ethyl)formamide (1l)

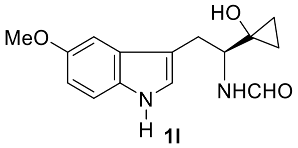

1l

Purification by column chromatography on silica gel (petroleum ether/EtOAc = 2/1, R_f = 0.20) afforded **1l** (17.5 mg, 64%) as a white solid.

^{1}H NMR (400 MHz, DMSO-d_6): δ 10.57 (s, 1H), 8.00 (d, J = 9.0 Hz, 1H), 7.94 (s, 1H), 7.23–7.17 (m, 1H), 7.09–7.02 (m, 2H), 6.70 (dd, J = 8.7, 2.3 Hz, 1H), 5.47 (s, 1H), 3.81 (dt, J = 9.6, 4.8 Hz, 1H), 3.76 (s, 3H), 3.12 (dd, J = 14.9, 3.8 Hz, 1H), 2.87–2.78 (m, 1H), 0.61–0.49 (m, 3H), 0.44–0.37 (m, 1H) ppm. **^{13}C NMR** (100 MHz, DMSO-

d_6): δ 160.9, 153.0, 131.4, 127.9, 123.7, 120.0, 111.6, 110.9, 100.4, 56.8, 55.5, 53.7, 27.1, 12.2, 11.2 ppm. **IR** (KBr): ν_{max} = 3319, 2938, 1669, 1485, 1414, 1021, 797 cm^{-1}. **HRMS** (m/z): Calcd for $C_{15}H_{18}N_2NaO_3$, $[M+Na]^+$, 297.1211; found 297.1211. **m.p.**: 142.2–145.7 °C. $[\alpha]_D^{25}$: –59.1 (*c* 1.26, MeOH).

2.5 General procedure for the preparation of 2a and 2e-2l

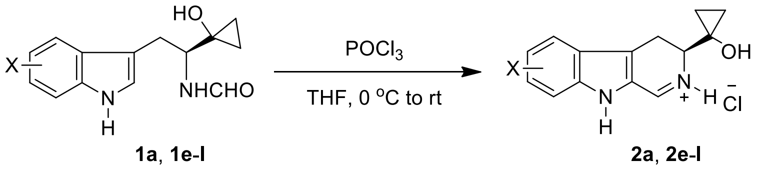

To a solution of cyclopropanol **1** (1.0 mmol) in dry THF (1 mL) under an atmosphere of argon was added $POCl_3$ (5.0 mmol) dropwise at 0 ℃. The reaction solution was warmed to room temperature and stirred for an additional 30 min. After **1** was completely consumed as judged by TLC analysis, the resulting mixture was concentrated under reduced pressure. The crude product was purified by flash column chromatography on silica gel to afford the pure product **2**.

(*S*)-1-(4,9-dihydro-3*H*-pyrido[3,4-*b*]indol-3-yl)cyclopropan-1-ol hydrochloride (2a)

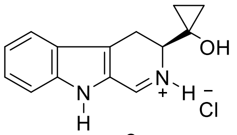

2a

Purification by column chromatography on silica gel (CH_2Cl_2/MeOH = 10/1, R_f = 0.20) afforded **2a** (188.7 mg, 72%) as a yellow solid.

^{1}H NMR (400 MHz, CD_3OD): δ 8.83 (s, 1H), 7.77 (d, *J* = 8.3 Hz, 1H), 7.58–7.45 (m, 2H), 7.28–7.19 (m, 1H), 3.80 (t, *J* = 11.0 Hz, 1H), 3.57 (d, *J* = 11.3 Hz, 2H), 1.02–0.82 (m, 4H) ppm. **^{13}C NMR** (100 MHz, CD_3OD): δ 155.5, 143.7, 130.7, 126.8, 125.9, 125.7, 123.1, 122.9 114.5, 63.0, 56.1, 22.8, 13.7, 12.5 ppm. **IR** (KBr): ν_{max} = 3283, 3015, 1297, 1550, 748, 545 cm^{-1}. **HRMS**(m/z): Calcd for $C_{14}H_{15}N_2O$, $[M–Cl]^+$, 227.1179; found 227.1171. **m.p.**: 164.4–166.2 ℃. $[\alpha]_D^{25}$: +127.9 (*c* 0.74, MeOH).

(*S*)-1-(5-bromo-4,9-dihydro-3*H*-pyrido[3,4-*b*]indol-3-yl)cyclopropan-1-ol hydrochloride (2e)

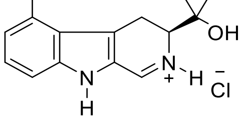

2e

Purification by column chromatography on silica gel (CH_2Cl_2/MeOH = 10/1, R_f = 0.30) afforded **2e** (261.8 mg, 77%) as a yellow solid.

^{1}H NMR (600 MHz, DMSO-d_6): δ 12.67 (s, 1H), 9.01 (s, 1H), 7.59 (d, J = 8.4 Hz, 1H), 7.39 (d, J = 7.4 Hz, 1H), 7.30 (t, J = 7.9 Hz, 1H), 5.98 (s, 1H), 3.82–3.68 (m, 3H), 0.88–0.75 (m, 4H) ppm. **^{13}C NMR** (150 MHz, DMSO-d_6): δ 155.9, 141.7, 128.9, 125.4, 124.9, 123.0, 122.7, 115.7, 113.3, 60.4, 55.4, 22.9, 13.1, 11.5 ppm. **IR** (KBr): v_{max} = 3213, 2927, 1731, 1560, 1410, 1200, 741 cm^{-1}. **HRMS**(m/z): Calcd for $C_{14}H_{12}BrN_2O$, [M–Cl–2H]$^-$, 303.0124; found 303.0138. **m.p.**: 245.2–245.9 ℃. **$[\alpha]_D^{25}$**: +32.5 (c 0.13, MeOH).

(*S*)-1-(6-bromo-4,9-dihydro-3*H*-pyrido[3,4-*b*]indol-3-yl)cyclopropan-1-ol hydrochloride (2f)

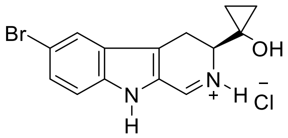

2f

Purification by column chromatography on silica gel (CH_2Cl_2/MeOH = 10/1, R_f = 0.20) afforded **2f** (204 mg, 60%) as a yellow solid.

^{1}H NMR (400 MHz, DMSO-d_6): δ 12.79 (s, 1H), 12.79 (s, 1H), 12.37 (s, 1H), 9.03 (s, 1H), 8.07 (s, 1H), 7.59–7.49 (m, 2H), 5.92 (s, 1H), 3.80 (dd, J = 11.9, 8.9 Hz, 1H), 3.49–3.42 (m, 2H), 0.87–0.71 (m, 4H) ppm. **^{13}C NMR** (100 MHz, DMSO-d_6): δ 156.0, 139.4, 130.7, 125.5, 125.4, 124.1, 122.5, 115.7, 113.7, 60.5, 55.3, 21.2, 13.0, 11.4 ppm. **IR** (KBr): v_{max} = 3456, 2927, 1560, 1410, 1133, 960, 741 cm^{-1}. **HRMS** (M/Z): Calcd for $C_{14}H_{12}BrN_2O$, [M–Cl–2H]$^-$, 303.0124; found 303.0138. **m. p.**: 237.5–238.4 ℃. **$[\alpha]_D^{25}$**: +320.2 (c 0.18, MeOH).

(*S*)-1-(7-bromo-4,9-dihydro-3*H*-pyrido[3,4-*b*]indol-3-yl)cyclopropan-1-ol hydrochloride (2g)

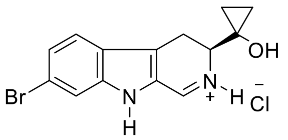

2g

Purification by column chromatography on silica gel (CH_2Cl_2/MeOH = 10/1, R_f = 0.40) afforded **2g** (244.8 mg, 72%) as a yellow solid.

^{1}H NMR (400 MHz, CD_3OD): δ 8.91 (s, 1H), 7.77 (s, 1H), 7.73 (d, J = 13.1 Hz, 1H), 7.36 (d, J = 12.7 Hz, 1H), 3.83 (t, J = 16.3 Hz, 1H), 3.60 (d, J = 15.8 Hz, 2H), 1.05–0.86 (m, 4H) ppm. **^{13}C NMR** (150 MHz, CD_3OD): δ 156.8, 143.6, 126.6, 126.4, 126.3, 124.3, 124.2, 117.0, 62.8, 55.9, 22.5, 13.6, 12.4 ppm. **IR** (KBr): v_{max} = 3457, 2923, 1711, 1631, 1435, 1166, 745 cm^{-1}. **HRMS** (m/z): Calcd for $C_{14}H_{12}BrN_2O$, [M–Cl–2H]$^-$, 303.0124; found 303.0138. **m. p.**: 242.1–243.5 ℃. **$[\alpha]_D^{25}$**: +30.8 (c 1.1, MeOH).

(*S*)-8-bromo-3-(1-hydroxycyclopropyl)-4,9-dihydro-3*H*-pyrido[3,4-*b*]indol-2-ium chloride (2h)

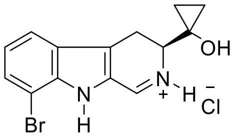

2h

Purification by column chromatography on silica gel (CH_2Cl_2/MeOH = 10/1, R_f = 0.40) afforded **2h** (278.8 mg, 82%) as a yellow solid.

^{1}H NMR (600 MHz, DMSO-*d*$_6$): δ 12.97 (s, 1H), 12.47 (s, 1H), 8.86 (s, 1H), 7.84 (d, *J* = 8.1 Hz, 1H), 7.70 (d, *J* = 7.4 Hz, 1H), 7.14 (t, *J* = 7.8 Hz, 1H), 5.96 (s, 1H), 3.85 (t, *J* = 10.3 Hz, 1H), 3.49 (t, *J* = 10.9 Hz, 2H), 0.88–0.82 (m, 2H), 0.79–0.75 (m, 2H) ppm. **^{13}C NMR** (100 MHz, DMSO-*d*$_6$): δ 155.7, 139.0, 130.5, 125.5, 125.5, 124.3, 122.5, 121.5, 105.5, 60.6, 55.5, 21.5, 13.0, 11.5 ppm. **IR** (KBr): ν_{max} = 3521, 2929, 1661, 1212, 959, 800 cm^{-1}. **HRMS** (m/z): Calcd for $C_{14}H_{14}BrN_2O$, [M–Cl]$^+$, 305.0285; found 305.0284. **m. p.**: 238.2–240.0 ℃. **[*α*]$_D^{25}$**: +292.8 (*c* 0.14, MeOH).

(*S*)-1-(6-chloro-4,9-dihydro-3*H*-pyrido[3,4-*b*]indol-3-yl)cyclopropan-1-ol hydrochloride (2i)

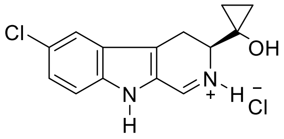

2i

Purification by column chromatography on silica gel (CH_2Cl_2/MeOH = 10/1, R_f = 0.10) afforded **2i** (210.2 mg, 71%) as a yellow solid.

^{1}H NMR (400 MHz, DMSO-*d*$_6$): δ 12.97 (s, 1H), 12.53 (s, 1H), 9.05 (s, 1H), 7.90 (s, 1H), 7.58 (d, *J* = 9.0 Hz, 1H), 7.39 (dd, *J* = 9.0, 2.1 Hz, 1H), 3.79 (t, *J* = 10.2 Hz, 1H), 3.44 (dd, *J* = 10.3, 5.6 Hz, 2H), 0.87–0.81 (m, 2H), 0.79–0.71 (m, 2H) ppm. **^{13}C NMR** (100 MHz, DMSO-*d*$_6$): δ 156.0, 139.3, 128.4, 125.8, 125.7, 124.8, 122.7, 120.9, 115.4, 60.5, 55.4, 21.3, 13.0, 11.5 ppm. **IR** (KBr): ν_{max} = 3440, 2956, 1550, 1208, 1023, 804 cm^{-1}. **HRMS** (m/z): Calcd for $C_{14}H_{14}ClN_2O$, [M–Cl]$^+$, 261.0791; found 261.0789. **m. p.**: 236.0–237.1 ℃. **[*α*]$_D^{25}$**: +164.8 (*c* 0.85, MeOH).

(*S*)-1-(7-fluoro-4,9-dihydro-3*H*-pyrido[3,4-*b*]indol-3-yl)cyclopropan-1-ol hydrochloride (2j)

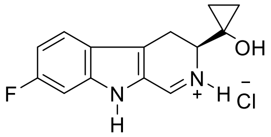

2j

Purification by column chromatography on silica gel (CH_2Cl_2/MeOH = 10/1, R_f = 0.10) afforded **2j** (182.0 mg, 65%) as a yellow solid.

^{1}H NMR (400 MHz, DMSO-*d*$_6$): δ 12.77 (s, 1H), 12.42 (s, 1H), 8.99 (s, 1H), 7.85 (dd, *J* = 9.0, 5.6 Hz, 1H), 7.37 (dd, *J* = 10.0, 1.8 Hz, 1H), 7.08 (td, *J* = 9.4, 2.4 Hz, 1H), 5.95 (s, 1H), 3.82 (t, *J* = 11.3 Hz, 1H), 3.51–3.41 (m, 2H),

0.89–0.71 (m, 4H) ppm. **^{13}C NMR** (100 MHz, DMSO-d_6): δ 162.6 ($J_{C\text{-}F}$ = 243.2 Hz), 155.1, 141.4 ($J_{C\text{-}F}$ = 13.5 Hz), 125.4, 124.2, 124.0 ($J_{C\text{-}F}$ = 11.2 Hz), 121.2, 111.3 ($J_{C\text{-}F}$ = 26.0 Hz), 99.0 ($J_{C\text{-}F}$ = 26.4 Hz), 60.4, 55.2, 21.5, 13.0, 11.4 ppm. **IR** (KBr): ν_{max} = 3440, 2963, 1546, 1213, 946, 804 cm^{-1}. **HRMS** (m/z): Calcd for $C_{14}H_{14}FN_2O$, [M–Cl]$^+$, 245.1087; found 245.1084. **m. p.**: 233.8–235.4 ℃. **$[\alpha]_D^{25}$**: +144.7 (*c* 0.76, MeOH).

(*S*)-3-(1-hydroxycyclopropyl)-7-methoxy-4,9-dihydro-3*H*-pyrido[3,4-*b*]indol-2-ium chloride (2k)

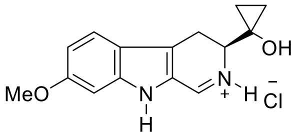

2k

Purification by column chromatography on silica gel (CH_2Cl_2/MeOH = 10/1, R_f = 0.10) afforded **2k** (210.3 mg, 72%) as a yellow solid.

^{1}H NMR (400 MHz, DMSO-d_6): δ 12.15 (s, 1H), 8.80 (s, 1H), 7.67 (d, J = 8.9 Hz, 1H), 6.96 (s, 1H), 6.82 (d, J = 9.0 Hz, 1H), 3.84 (s, 3H), 3.70 (dd, J = 13.4, 8.8 Hz, 1H), 3.44–3.25 (m, 2H), 0.86–0.65 (m, 4H) ppm. **^{13}C NMR** (100 MHz, DMSO-d_6): δ 161.0, 143.3, 125.3, 124.3, 123.0, 119.0, 114.5, 100.9, 93.9, 60.3, 55.6, 54.9, 21.8, 12.9, 11.4 ppm. **IR** (KBr): ν_{max} = 3440, 2952, 1556, 1283, 1124, 965 cm^{-1}. **HRMS** (m/z): Calcd for $C_{15}H_{17}N_2O_2$, [M–Cl]$^+$, 257.1286; found 257.1284. **m. p.**: 200.2–202.0 ℃. **$[\alpha]_D^{25}$**: +343.4 (*c* 0.28, MeOH).

(*S*)-3-(1-hydroxycyclopropyl)-6-methoxy-4,9-dihydro-3*H*-pyrido[3,4-*b*]indol-2-ium chloride (2l)

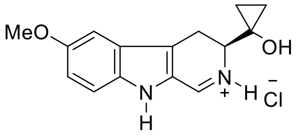

2l

Purification by column chromatography on silica gel (CH_2Cl_2/MeOH = 10/1, R_f = 0.10) afforded **2l** (236.6 mg, 81%) as a yellow solid.

^{1}H NMR (400 MHz, DMSO-d_6): δ 12.56 (s, 1H), 12.35–12.21 (m, 1H), 8.92 (s, 1H), 7.47 (d, J = 9.1 Hz, 1H), 7.18 (s, 1H) 7.08 (d, J = 9.0 Hz, 1H), 5.96 (s, 1H), 3.80 (s, 3H), 3.45–3.36 (m, 3H), 0.88–0.69 (m, 4H) ppm. **^{13}C NMR** (100 MHz, DMSO-d_6): δ 154.8, 154.5, 137.2, 125.1, 124.4, 122.6, 121.3, 114.7, 100.4, 60.5, 55.4, 55.2, 21.6, 12.9, 11.3 ppm. **IR** (KBr): ν_{max} = 3457, 2923, 1711, 1631, 1435, 1166, 745 cm^{-1}. **HRMS** (m/z): Calcd for $C_{15}H_{17}N_2O_2$, [M–Cl]$^+$, 257.1290; found 257.1286. **m. p.**: 132.5–134.2 ℃. **$[\alpha]_D^{25}$**: +69.9 (*c* 0.54, MeOH).

2.6 Preparation of 2b

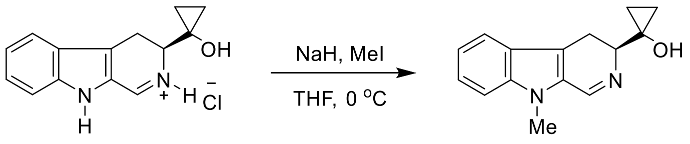

2a **2b**

To a mixture of NaH (60% dispersion on mineral oil, 24 mg, 0.6 mmol) in dry THF (3 mL) was added **2a** (78.6 mg, 0.3 mmol) at 0 ℃. The reaction mixture was stirred at room temperature for 15 min, and then MeI (85.2 mg, 0.6 mmol) was added dropwise. The mixture was stirred for an additional 2 h at room temperature before a saturated aqueous solution of NH_4Cl (20 mL) was added to quench the reaction. The reaction mixture was extracted with EtOAc (3 × 15 mL) and washed with brine (3 × 15 mL). The combined organic extracts were dried over Na_2SO_4, filtered, and concentrated. The crude product was purified by flash column chromatography on silica gel (CH_2Cl_2/MeOH = 10/1, R_f = 0.20) to afford **2b** (64.8 mg, 90%) as a yellow oil.

^{1}H NMR (400 MHz, $CDCl_3$): δ 8.48 (d, J = 3.1 Hz, 1H), 7.60 (d, J = 8.0 Hz, 1H), 7.34–7.29 (m, 2H), 7.15 (ddd, J = 8.0, 5.4, 2.5 Hz, 1H), 3.80 (s, 3H), 3.24 (ddd, J = 15.8, 8.2, 3.0 Hz, 1H), 3.13–2.94 (m, 3H), 1.11–0.94 (m, 2H), 0.86–0.77 (m, 1H), 0.71–0.63 (m, 1H) ppm. **^{13}C NMR** (100 MHz, $CDCl_3$): δ 151.0, 139.1, 129.2, 125.3, 124.8, 120.6, 120.5, 118.3, 110.2, 65.5, 57.0, 29.7, 21.4, 13.9, 11.5 ppm. **IR** (KBr): ν_{max} = 3054, 2929, 1540, 1463, 1289, 742 cm^{-1}. **HRMS** (m/z): Calcd for $C_{15}H_{17}N_2O$, $[M+H]^+$, 241.1335; found 241.1326. **$[\alpha]_D^{25}$**: +125.1 (*c* 0.35, MeOH).

2.7 Preparation of 2c

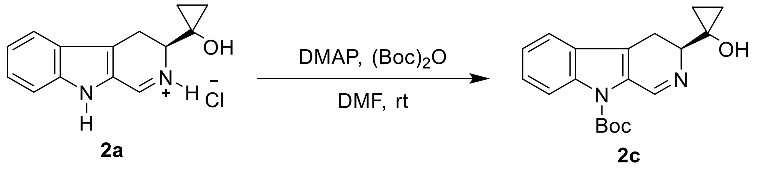

To a solution of **2a** (78.6 mg, 0.3 mmol) in DMF (3 mL) were added DMAP (73.2 mg, 0.6 mmol) and $(Boc)_2O$ (130.9 mg, 0.6 mmol). The mixture was stirred for 45 min at room temperature before a saturated aqueous solution of NH_4Cl (10 mL) was added to quench the reaction. The mixture was then extracted with EtOAc (4 ×20 mL) and washed with brine (4 ×20 mL). The combined organic extracts were dried over Na_2SO_4, filtered, and concentrated in vacuo. The crude product was purified by flash column chromatography on silica gel (CH_2Cl_2/MeOH = 10/1, R_f = 0.50) to afford **2c** (85.8 mg, 79%) as a white solid.

^{1}H NMR (400 MHz, $CDCl_3$): δ 8.89 (s, 1H), 8.19 (d, J = 8.2 Hz, 1H), 7.56 (d, J = 7.6 Hz, 1H), 7.40 (s, 1H), 7.33–7.23 (m, 1H), 3.25–3.13 (m, 1H), 3.05–2.90 (m, 2H), 1.68 (s, 9H), 1.31–1.20 (m, 1H), 1.04–0.96 (m, 1H), 0.84–0.75 (m, 1H), 0.71–0.63 (m, 1H) ppm. **^{13}C NMR** (100 MHz, $CDCl_3$): δ 153.6, 149.9, 149.9, 137.3, 128.8, 127.6, 127.1, 125.4, 123.6, 120.2, 116.4, 110.2, 85.0, 63.9, 57.6, 28.4, 20.9, 13.5, 11.3 ppm. **IR** (KBr): ν_{max} = 3440, 2978, 1732, 1372, 1143, 745 cm^{-1}. **HRMS** (m/z): Calcd for $C_{19}H_{23}N_2O_3$, $[M+H]^+$, 327.1709; found 327.1706. **m. p.**: 74.9–76.0 ℃. **$[\alpha]_D^{25}$**: +18.7 (*c* 0.45, MeOH).

2.8 Preparation of 2d

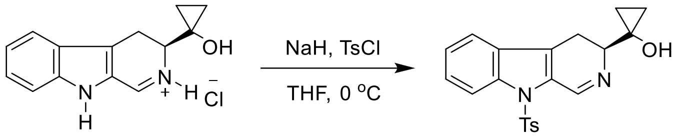

To a mixture of NaH (60% dispersion on mineral oil, 80 mg, 2.0 mmol) in dry THF (10 mL) was added **2a** (76.8 mg, 0.3 mmol) at 0 ℃. The reaction mixture was stirred at room temperature for 15 min, and then TsCl (228 mg, 1.2 mmol) was added slowly. The mixture was stirred at room temperature until the full consumption of **2a**, and a saturated aqueous solution of NH_4Cl (10 mL) was added to quench the reaction. The resulting mixture was extracted with EtOAc (3 × 15 mL), and washed with brine (3 × 15 mL). The combined organic extract was dried over Na_2SO_4, filtered, and concentrated in vacuo. The crude product was purified by flash column chromatography on silica gel (CH_2Cl_2/MeOH = 10/1, R_f = 0.40) to afford **2d** (102.6 mg, 90%) as a yellow oil.

^{1}H NMR (400 MHz, $CDCl_3$): δ 8.16 (d, J = 8.4 Hz, 1H), 7.62 (d, J = 8.1 Hz, 2H), 7.51 (d, J = 7.9 Hz, 1H), 7.44 (t, J = 7.8 Hz, 1H), 7.29 (t, J = 7.5 Hz, 1H), 7.10 (d, J = 8.0 Hz, 2H), 3.18–2.86 (m, 3H), 2.27 (s, 3H), 1.10–1.02 (m, 1H), 0.98 (dt, J = 10.6, 5.6 Hz, 1H), 0.86–0.78 (m, 1H), 0.62 (dt, J = 11.0, 5.7 Hz, 1H) ppm. **^{13}C NMR** (100 MHz, $CDCl_3$): δ 151.8, 145.3, 137.4, 134.8, 130.0, 128.9, 127.9, 127.8, 127.4, 126.7, 124.5, 120.6, 115.3, 64.3, 57.7, 21.7, 21.0, 13.6, 11.3 ppm. **IR** (KBr): ν_{max} = 3440, 2929, 1670, 1547, 1175, 674 cm^{-1}. **HRMS** (m/z): Calcd for $C_{21}H_{21}N_2O_3S$, [M+H]$^+$, 381.1267; found 381.1252. $[\alpha]_D^{25}$: +95.2 (c 0.21, MeOH).

2.9 General procedure for the preparation of 3

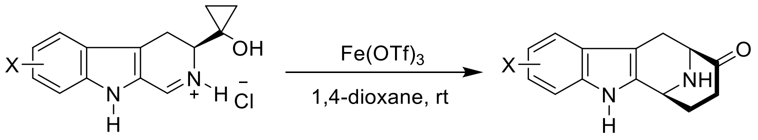

To a solution of **2** (0.2 mmol) in dry 1,4-dioxane (2 mL) under an atmosphere of argon was added $Fe(OTf)_3$ (0.4 mmol). The reaction mixture was stirred for 10 min at room temperature, and then was quenched with a saturated aqueous solution of $NaHCO_3$ (10 mL). After filtration through a pad of Celite, the resulting mixture was extracted with EtOAc (3 × 20 mL). The combined organic phase was washed with brine (3 × 20 mL), dried over Na_2SO_4, filtered, and concentrated. The crude product was purified by flash column chromatography on silica gel to afford the pure product **3**. Compounds **3b**, **3c** and **3d** were prepared following the above procedure from the corresponding free imine **2b**, **2c**, and **2d**, respectively.

(6*S*, 10*S*)-5,6,7,8,10,11-hexahydro-9*H*-6,10-epiminocycloocta[*b*]indol-9-one (3a)

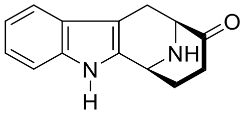

3a

Purification by column chromatography on silica gel (petroleum ether/EtOAc = 1/1, R_f = 0.10) afforded **3a** (32.7 mg, 72%) as a yellow solid.

^{1}H NMR (400 MHz, $CDCl_3$): δ 8.02 (s, 1H), 7.46 (d, J = 7.7 Hz, 1H), 7.32 (d, J = 8.0 Hz, 1H), 7.21–7.15 (m, 1H), 7.12 (t, J = 7.4 Hz, 1H), 4.30 (d, J = 3.7 Hz, 1H), 3.94 (d, J = 6.7 Hz, 1H), 3.11 (dd, J = 16.5, 6.8 Hz, 1H), 2.82 (d, J = 16.5 Hz, 1H), 2.52–2.39 (m, 2H), 2.20–2.07 (m, 3H) ppm. **^{13}C NMR** (100 MHz, $CDCl_3$): δ 211.1, 135.9, 134.1, 127.1, 122.3, 119.9, 118.3, 111.1, 107.7, 60.0, 46.3, 35.2, 32.2, 26.0 ppm. **IR** (KBr): ν_{max} = 3610, 3184, 1703, 1401, 745, 541 cm^{-1}. **HRMS** (m/z): Calcd for $C_{14}H_{15}N_2O$, $[M+H]^+$, 227.1179; found 227.1184. **m. p.**: 67.5–68.4 ℃. **$[\alpha]^{25}_{D}$**: –48.2 (c 0.17, $CHCl_3$).

(6*S*, 10*S*)-5-methyl-5,6,7,8,10,11-hexahydro-9*H*-6,10-epiminocycloocta[*b*]indol-9-one (3b)

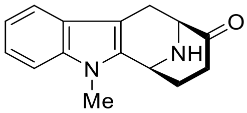

3b

Purification by column chromatography on silica gel (petroleum ether/EtOAc = 10/1, R_f = 0.25) afforded **3b** (43.4 mg, 90%) as a yellow oil.

^{1}H NMR (400 MHz, $CDCl_3$): δ 7.48 (d, J = 7.9 Hz, 1H), 7.31 (d, J = 8.2 Hz, 1H), 7.27–7.20 (m, 1H), 7.12 (t, J = 7.5 Hz, 1H), 4.41 (s, 1H), 3.96 (d, J = 6.8 Hz, 1H), 3.66 (s, 3H), 3.15 (dd, J = 16.5, 6.9 Hz, 1H), 2.84 (d, J = 16.5 Hz, 1H), 2.54–2.45 (m, 2H), 2.38 (s, 1H), 2.22–2.08 (m, 2H) ppm. **^{13}C NMR** (100 MHz, $CDCl_3$): δ 210.6, 137.1, 135.2, 126.6, 121.8, 119.5, 118.3, 109.0, 106.6, 59.8, 45.1, 35.1, 31.5, 29.5, 26.0. ppm. **IR** (KBr): ν_{max} = 2927, 1710, 1469, 1100, 742 cm^{-1}. **HRMS** (m/z): Calcd for $C_{15}H_{17}N_2O$, $[M+H]^+$, 241.1335; found 241.1327. **$[\alpha]^{25}_{D}$**: –67.0 (c 0.15, $CHCl_3$).

***tert*-butyl (6*S*, 10*S*)-9-oxo-6,7,8,9,10,11-hexahydro-5*H*-6,10-epiminocycloocta[*b*]indole-5-carboxylate (3c)**

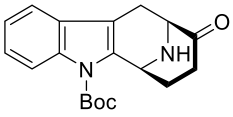

3c

Purification by column chromatography on silica gel (petroleum ether/EtOAc = 10/1, R_f = 0.30) afforded **3c** (51.7 mg, 79%) as a yellow oil.

^{1}H NMR (400 MHz, $CDCl_3$): δ 8.15 (d, J = 8.3 Hz, 1H), 7.40 (d, J = 7.6 Hz, 1H), 7.30 (t, J = 7.7 Hz, 1H), 7.24 (dd, J = 11.7, 5.1 Hz, 1H), 4.92 (s, 1H), 3.92 (d, J = 6.9 Hz, 1H), 3.04 (dd, J = 17.1, 7.0 Hz, 1H), 2.76 (d, J = 17.1 Hz, 1H), 2.56–2.43 (m, 2H), 2.34 (s, 1H), 2.24–2.13 (m, 2H), 1.70 (s, 9H) ppm. **^{13}C NMR** (100 MHz, $CDCl_3$): δ

210.8, 150.2, 135.8, 129.0, 124.6, 123.0, 118.0, 115.9, 114.3, 84.3, 58.8, 46.9, 35.2, 31.5, 28.4, 25.7 ppm. **IR** (KBr): ν_{max} = 2977, 1727, 1454, 1369, 1139, 749 cm^{-1}. **HRMS** (m/z): Calcd for $C_{19}H_{23}N_2O_3$, $[M+H]^+$, 327.1709; found 327.1706. $[\alpha]_D^{25}$: –120.8 (*c* 0.5, $CHCl_3$).

(6*S*, 10*S*)-5-tosyl-5,6,7,8,10,11-hexahydro-9*H*-6,10-epiminocycloocta[*b*]indol-9-one (3d)

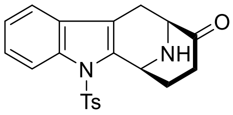

3d

Purification by column chromatography on silica gel (CH_2Cl_2/MeOH = 10/1, R_f = 0.50) afforded **3d** (41.1 mg, 90%) as a yellow oil.

^{1}H NMR (400 MHz, $CDCl_3$): δ 8.14 (d, *J* = 8.3 Hz, 1H), 7.64 (d, *J* = 8.0 Hz, 2H), 7.37–7.32 (m, 2H), 7.29–7.23 (m, 2H), 7.19 (d, *J* = 8.1 Hz, 2H), 4.92 (s, 1H), 3.89 (d, *J* = 7.0 Hz, 1H), 3.00 (dd, *J* = 17.2, 7.0 Hz, 1H), 2.72 (d, *J* = 17.1 Hz, 1H), 2.57–2.49 (m, 3H), 2.33 (s, 3H), 2.18–2.03 (m, 3H) ppm. **^{13}C NMR** (100 MHz, $CDCl_3$): δ 210.4, 145.2, 136.4, 135.5, 130.1, 129.5, 126.4, 125.2, 124.0, 118.6, 117.0, 114.9, 58.4, 47.1, 35.2, 32.5, 25.6, 21.7 ppm. **IR** (KBr): ν_{max} = 2927, 1713, 1371, 975, 657 cm^{-1}. **HRMS** (m/z): Calcd for $C_{21}H_{21}N_2O_3S$, $[M+H]^+$, 381.1273; found 381.1269. $[\alpha]_D^{25}$: –39.9 (*c* 0.13, $CHCl_3$).

(6*S*, 10*S*)-1-bromo-5,6,7,8,10,11-hexahydro-9*H*-6,10-epiminocycloocta[*b*]indol-9-one (3e)

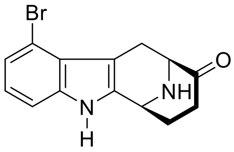

3e

Purification by column chromatography on silica gel (CH_2Cl_2/MeOH = 10/1, R_f = 0.15) afforded **3e** (42.6 mg, 77%) as a yellow solid.

^{1}H NMR (600 MHz, $CDCl_3$): δ 8.03 (s, 1H), 7.26 (s, 1H), 7.25–7.21 (m, 1H), 6.98 (t, *J* = 7.9 Hz, 1H), 4.33 (s, 1H), 3.95 (d, *J* = 6.6 Hz, 1H), 3.38 (dd, *J* = 17.0, 6.7 Hz, 1H), 3.30 (d, *J* = 17.0 Hz, 1H), 2.55–2.41 (m, 2H), 2.24 (s, 1H), 2.20–2.12 (m, 2H) ppm. **^{13}C NMR** (150 MHz, $CDCl_3$): δ 210.5, 136.8, 135.1, 126.3, 123.9, 123.1, 113.9, 110.2, 108.6, 59.9, 46.3, 35.2, 32.2, 27.8 ppm. **IR** (KBr): ν_{max} = 3398, 2917, 1687, 1127, 733 cm^{-1}. **HRMS** (m/z): Calcd for $C_{14}H_{12}BrN_2O$, $[M–H]^-$, 303.0123; found 303.0138. **m.p.**: 229.0–231.0 ℃. $[\alpha]_D^{25}$: –18.0 (*c* 0.24, $CHCl_3$).

(6*S*, 10*S*)-2-bromo-5,6,7,8,10,11-hexahydro-9*H*-6,10-epiminocycloocta[*b*]indol-9-one (3f)

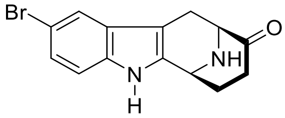

3f

Purification by column chromatography on silica gel (CH_2Cl_2/MeOH = 10/1, R_f = 0.30) afforded **3f** (40.7 mg, 60%) as a yellow solid.

^{1}H NMR (400 MHz, $CDCl_3$): δ 8.04 (s, 1H), 7.57 (s, 1H), 7.26–7.22 (m, 1H), 7.18 (d, *J* = 8.5 Hz, 1H), 4.34 (s, 1H), 3.94 (d, *J* = 6.6 Hz, 1H), 3.06 (dd, *J* = 16.5, 6.7 Hz, 1H), 2.77 (d, *J* = 16.5 Hz, 1H), 2.62 (s, 1H), 2.56–2.41 (m, 2H), 2.18–2.07 (m, 2H) ppm. **^{13}C NMR** (100 MHz, $CDCl_3$): δ 210.5, 135.4, 134.5, 128.8, 125.1, 121.0, 113.2, 112.5, 107.6, 59.7, 46.2, 35.1, 32.1, 25.8 ppm. **IR** (KBr): ν_{max} = 3300, 2928, 1705, 1442, 1263, 797, 736 cm^{-1}. **HRMS** (m/z): Calcd for $C_{14}H_{14}BrN_2O$, $[M+H]^+$, 305.0284; found 305.0274. **m.p.**: 214.1–215.3 ℃. $[\alpha]_D^{25}$: –133.0 (*c* 0.12, $CHCl_3$).

(6*S*, 10*S*)-3-bromo-5,6,7,8,10,11-hexahydro-9*H*-6,10-epiminocycloocta[*b*]indol-9-one (3g)

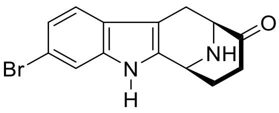

3g

Purification by column chromatography on silica gel (CH_2Cl_2/MeOH = 10/1, R_f = 0.30) afforded **3g** (41.3 mg, 72%) as a yellow solid.

^{1}H NMR (400 MHz, $CDCl_3$): δ 8.01 (s, 1H), 7.46 (d, *J* = 1.1 Hz, 1H), 7.30 (d, *J* = 8.4 Hz, 1H), 7.21 (dd, *J* = 8.4, 1.4 Hz, 1H), 4.33 (d, *J* = 3.8 Hz, 1H), 3.95 (d, *J* = 6.6 Hz, 1H), 3.09 (dd, *J* = 16.5, 6.7 Hz, 1H), 2.79 (d, *J* = 16.4 Hz, 1H), 2.54–2.42 (m, 2H), 2.28 (s, 1H), 2.18–2.08 (m, 2H) ppm. **^{13}C NMR** (100 MHz, $CDCl_3$): δ 210.7, 136.6, 134.7, 125.9, 123.2, 119.5, 115.6, 114.1, 108.0, 59.7, 46.2, 35.2, 32.0, 25.8 ppm. **IR** (KBr): ν_{max} = 3398, 2925, 1703,1463, 1150, 851, 734 cm^{-1}. **HRMS** (m/z): Calcd for $C_{14}H_{14}BrN_2O$, $[M+H]^+$, 305.0286; found 305.0284. **m.p.**: 208.0–209.3 ℃. $[\alpha]_D^{25}$: –37.0 (*c* 0.42, $CHCl_3$).

(6*S*, 10*S*)-4-bromo-5,6,7,8,10,11-hexahydro-9*H*-6,10-epiminocycloocta[*b*]indol-9-one (3h)

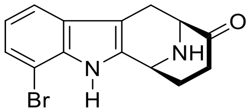

3h

Purification by column chromatography on silica gel (CH_2Cl_2/MeOH = 10/1, R_f = 0.30) afforded **3h** (45.2 mg, 82%) as a yellow solid.

^{1}H NMR (600 MHz, $CDCl_3$): δ 8.20 (s, 1H), 7.38 (d, *J* = 7.8 Hz, 1H), 7.32 (d, *J* = 7.6 Hz, 1H), 6.99 (t, *J* = 7.7 Hz, 1H), 4.43 (s, 1H), 3.97 (d, *J* = 6.6 Hz, 1H), 3.11 (dd, *J* = 16.4, 6.7 Hz, 1H), 2.88 (s, 1H), 2.81 (d, *J* = 16.5 Hz, 1H), 2.55–2.40 (m, 2H), 2.22–2.09 (m, 2H) ppm. **^{13}C NMR** (150 MHz, $CDCl_3$): δ 210.3, 134.6, 128.2, 124.7, 121.2, 117.5, 109.0, 104.8, 59.6, 46.2, 35.1, 31.8, 25.9 ppm. **IR** (KBr): ν_{max} = 3398, 2917, 1687, 1397, 1127, 733, 610 cm^{-1}. **HRMS** (m/z): Calcd for $C_{14}H_{14}BrN_2O$, $[M+H]^+$, 305.0285; found 305.0284. $[\alpha]_D^{25}$: –67.9 (*c* 0.25, $CHCl_3$).

(6*S*, 10*S*)-2-chloro-5,6,7,8,10,11-hexahydro-9*H*-6,10-epiminocycloocta[*b*]indol-9-one (3i)

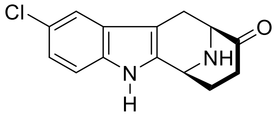

3i

Purification by column chromatography on silica gel (CH_2Cl_2/MeOH = 10/1, R_f = 0.45) afforded **3i** (39.5 mg, 71%) as a yellow solid.

^{1}H NMR (400 MHz, $CDCl_3$): *δ* 8.04 (s, 1H), 7.41 (s, 1H), 7.22 (s, 1H), 7.13 (d, *J* = 7.0 Hz, 1H), 4.40 (s, 1H), 3.98 (d, *J* = 6.6 Hz, 1H), 3.11 (dd, *J* = 16.5, 6.7 Hz, 1H), 2.79 (d, *J* = 16.5 Hz, 1H), 2.56–2.44 (m, 2H), 2.20–2.06 (m, 3H) ppm. **^{13}C NMR** (100 MHz, $CDCl_3$): δ 210.0, 135.0, 134.3, 128.0, 125.7, 122.6, 117.9, 112.1, 107.4, 59.4, 46.2, 35.0, 31.5, 25.6 ppm. **IR** (KBr): ν_{max} = 3433, 2924, 1709, 1444, 1263, 799, 748 cm^{-1}. **HRMS** (m/z): Calcd for $C_{14}H_{14}ClN_2O$, $[M+H]^+$, 261.0791; found 261.0789. **m.p.**: 124.0–125.7 ℃. $[\alpha]_D^{25}$: –138.1 (*c* 0.18, $CHCl_3$).

(6*S*,10*S*)-3-fluoro-5,6,7,8,10,11-hexahydro-9*H*-6,10-epiminocycloocta[*b*]indol-9-one (3j)

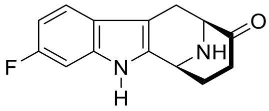

3j

Purification by column chromatography on silica gel (CH_2Cl_2/MeOH = 10/1, R_f = 0.60) afforded **3j** (29.3 mg, 68%) as a yellow solid.

^{1}H NMR (400 MHz, DMSO-d_6): *δ* 10.98 (s, 1H), 7.34 (dd, *J* = 8.5, 5.6 Hz, 1H), 7.09 (dd, *J* = 10.2, 2.1 Hz, 1H), 6.83–6.76 (m, 1H), 4.19 (s, 1H), 3.66 (d, *J* = 6.2 Hz, 1H), 2.92 (dd, *J* = 15.9, 6.5 Hz, 1H), 2.59 (d, *J* = 15.9 Hz, 1H), 2.42–2.27 (m, 2H), 2.06–1.96 (m, 1H), 1.95–1.83 (m, 1H) ppm. **^{13}C NMR** (150 MHz, DMSO-d_6): *δ* 212.0, 158.6 (d, $J_{C\text{-}F}$ = 233.3 Hz), 136.4 (d, $J_{C\text{-}F}$ = 3.0 Hz), 135.5 (d, $J_{C\text{-}F}$ = 12.0 Hz), 123.5, 118.3 (d, $J_{C\text{-}F}$ = 10.5 Hz), 106.5 (d, $J_{C\text{-}F}$ = 24.0 Hz), 106.0, 97.3 (d, $J_{C\text{-}F}$ = 25.5 Hz), 59.0, 45.1, 34.9, 31.5, 25.1 ppm. **IR** (KBr): ν_{max} = 3404, 2923, 1617, 1129, 797, 610 cm^{-1}. **HRMS** (m/z): Calcd for $C_{14}H_{14}FN_2O$, $[M+H]^+$, 245.1087; found 245.1084. **m.p.**: 205.3–207.0 ℃. $[\alpha]_D^{25}$: –106.0 (*c* 0.50, $CHCl_3$).

(6*S*, 10*S*)-3-methoxy-5,6,7,8,10,11-hexahydro-9*H*-6,10-epiminocycloocta[*b*]indol-9-one (3k)

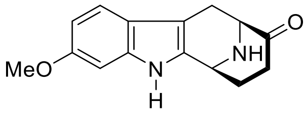

3k

Purification by column chromatography on silica gel (CH_2Cl_2/MeOH = 10/1, R_f = 0.50) afforded **3k** (26.1 mg, 72%) as a yellow solid.

^{1}H NMR (400 MHz, $CDCl_3$): *δ* 7.78 (s, 1H), 7.33 (d, *J* = 8.6 Hz, 1H), 6.84 (d, *J* = 2.2 Hz, 1H), 6.78 (dd, *J* = 8.6, 2.3 Hz, 1H), 4.31 (s, 1H), 3.94 (d, *J* = 6.6 Hz, 1H), 3.84 (s, 3H), 3.09 (dd, *J* = 16.5, 6.7 Hz, 1H), 2.78 (d, *J* = 16.5

Hz, 1H), 2.53–2.41 (m, 2H), 2.22–2.09 (m, 2H), 1.94 (s, 1H) ppm. **^{13}C NMR** (100 MHz, $CDCl_3$): δ 210.9, 156.7, 136.7, 132.6, 121.4, 118.9, 109.3, 107.7, 95.2, 59.9, 55.9, 46.3, 35.2, 32.0, 26.0 ppm. **IR** (KBr): ν_{max} = 2928, 1705, 1482, 1456, 1215, 1029, 800, 736 cm^{-1}. **HRMS** (m/z): Calcd for $C_{15}H_{17}N_2O_2$, [M+H]$^+$, 257.1286; found 257.1284. **m. p.**: 105.1–106.7 ℃. **[α]$_D^{25}$**: –18.9 (*c* 0.7, $CHCl_3$).

(6*S*, 10*S*)-2-methoxy-5,6,7,8,10,11-hexahydro-9*H*-6,10-epiminocycloocta[*b*]indol-9-one (3l)

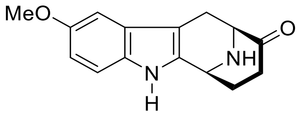

3l

Purification by column chromatography on silica gel (CH_2Cl_2/MeOH = 10/1, R_f = 0.25) afforded **3l** (28.2 mg, 81%) as a yellow solid.

^{1}H NMR (400 MHz, $CDCl_3$): δ 7.85 (s, 1H), 7.21 (d, *J* = 8.7 Hz, 1H), 6.90 (s, 1H), 6.83 (d, *J* = 8.6 Hz, 1H), 4.29 (s, 1H), 3.94 (d, *J* = 6.6 Hz, 1H), 3.85 (s, 3H), 3.09 (dd, *J* = 16.4, 6.7 Hz, 1H), 2.78 (d, *J* = 16.4 Hz, 1H), 2.55–2.38 (m, 2H), 2.29 (s, 1H), 2.18–2.07 (m, 2H) ppm. **^{13}C NMR** (100 MHz, $CDCl_3$): δ 211.0, 154.3, 134.9, 130.8, 127.5, 112.1, 111.8, 107.6, 100.4, 59.9, 56.0, 46.3, 35.2, 32.2, 26.0 ppm. **IR** (KBr): ν_{max} = 3394, 2927, 1704, 1482, 1214, 1028, 800 cm^{-1}. **HRMS** (m/z): Calcd for $C_{15}H_{17}N_2O_2$, [M+H]$^+$, 257.1286; found 257.1284. **m.p.**: 93.1–94.6 ℃. **[*α*]$_D^{25}$**: –104.0 (*c* 0.35, $CHCl_3$).

2.10 General procedure for the preparation of 13

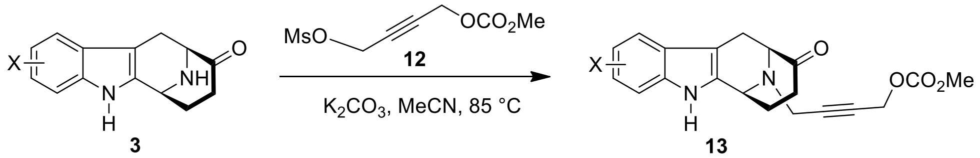

To a solution of **3** (0.1 mmol) and K_2CO_3 (27.6 mg, 0.2 mmol) in dry MeCN (1 mL) was added **12** (26.6 mg, 1.2 mmol). After being stirred at 85 ℃ for 5 h, the resulting mixture was cooled to the ambient temperature. The mixture was diluted with H_2O (10 mL), and was extracted with EtOAc (3 × 10 mL). The combined organic phase was washed with brine (3 × 10 mL), dried over Na_2SO_4, filtered, and concentrated. The residue was purified by flash column chromatography on silica gel to afford the pure product **13**.

methyl (4-((6*S*, 10*S*)-9-oxo-6,7,8,9,10,11-hexahydro-5*H*-6,10-epiminocycloocta[*b*]indol-12-yl)but-2-yn-1-yl) carbonate (13a)

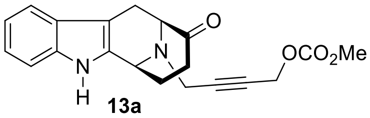

Purification by column chromatography on silica gel (petroleum ether/EtOAc = 10/1, R_f = 0.20) afforded **13a** (28.5 mg, 81%) as a yellow oil.

^{1}H NMR (400 MHz, $CDCl_3$): δ 7.98 (s, 1H), 7.46 (d, *J* = 7.8 Hz, 1H), 7.34 (d, *J* = 8.0 Hz, 1H), 7.22–7.16 (m, 1H), 7.12 (t, *J* = 7.4 Hz, 1H), 4.75 (s, 2H), 4.30 (s, 1H), 3.88 (d, *J* = 6.6 Hz, 1H), 3.82 (s, 3H), 3.46 (q, *J* = 16.3 Hz, 2H), 3.14 (dd, *J* = 17.0, 6.6 Hz, 1H), 2.68 (d, *J* = 17.0 Hz, 1H), 2.55–2.41 (m, 2H), 2.15–2.02 (m, 2H) ppm. **^{13}C NMR** (100 MHz, $CDCl_3$): δ 209.5, 155.3, 136.1, 131.2, 126.8, 122.3, 119.9, 118.4, 111.1, 106.6, 83.6, 78.3, 64.4, 55.9, 55.3, 50.9, 41.7, 34.3, 30.1, 20.2 ppm. **IR** (KBr): ν_{max} = 3611, 3300, 3195, 1660, 746, 542 cm^{-1}. **HRMS** (m/z): Calcd for $C_{20}H_{21}N_2O_4$, $[M+H]^+$, 353.1496; found 353.1503. $[\alpha]_D^{25}$: –148.3 (*c* 0.1, MeOH).

4-((6*S*, 10*S*)-1-bromo-9-oxo-6,7,8,9,10,11-hexahydro-5*H*-6,10epiminocycloocta[*b*]indol-12-yl)but-2-yn-1-yl methyl carbonate (13e)

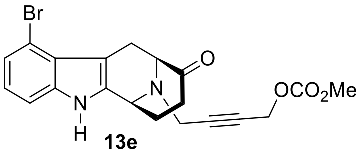

13e

Purification by column chromatography on silica gel (petroleum ether/EtOAc = 2/1, R_f = 0.16) afforded **13e** (21.5 mg, 50%) as a yellow oil.

^{1}H NMR (400 MHz, $CDCl_3$): δ 8.19 (s, 1H), 7.28–7.20 (m, 2H), 6.98 (t, *J* = 7.9 Hz, 1H), 4.74 (s, 2H), 4.29 (s, 1H), 3.85 (d, *J* = 6.6 Hz, 1H), 3.81 (s, 3H), 3.55–3.35 (m, 3H), 3.14 (d, *J* = 17.6 Hz, 1H), 2.55–2.40 (m, 2H), 2.18–2.02 (m, 2H) ppm. **^{13}C NMR** (100 MHz, $CDCl_3$): δ 209.3, 155.3, 137.1, 132.2, 125.9, 123.8, 123.1, 113.9, 110.3, 107.3, 83.5, 78.4, 64.4, 55.9, 55.3, 50.8, 41.7, 34.3, 30.1, 22.1 ppm. **IR** (KBr): ν_{max} = 3685, 3413, 2925, 1749, 1267, 617 cm^{-1}. **HRMS** (m/z): Calcd for $C_{20}H_{20}BrN_2O_4$, $[M+H]^+$, 431.0601; found 431.0598. $[\alpha]_D^{25}$: –68.7 (*c* 0.15, $CHCl_3$).

4-((6*S*, 10*S*)-2-bromo-9-oxo-6,7,8,9,10,11-hexahydro-5*H*-6,10-epiminocycloocta[*b*]indol-12-yl)but-2-yn-1-yl methyl carbonate (13f)

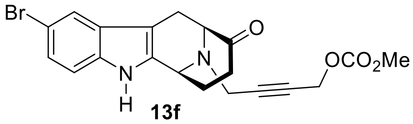

13f

Purification by column chromatography on silica gel (petroleum ether/EtOAc = 2/1, R_f = 0.16) afforded **13f** (28.8 mg, 67%) as a yellow oil.

^{1}H NMR (400 MHz, $CDCl_3$): δ 8.26 (s, 1H), 7.55 (s, 1H), 7.27 (s, 1H), 7.28–7.21 (m, 1H), 7.18 (d, *J* = 8.5 Hz, 1H), 4.73 (s, 2H), 4.27 (s, 1H), 3.85 (d, *J* = 6.2 Hz, 1H), 3.80 (s, 3H), 3.43 (q, *J* = 16.3 Hz, 2H), 3.06 (dd, *J* = 17.0, 6.5 Hz, 1H), 2.61 (d, *J* = 17.0 Hz, 1H), 2.50–2.38 (m, 2H), 2.08–1.99 (m, 2H) ppm. **^{13}C NMR** (100 MHz, $CDCl_3$): δ 209.4, 155.3, 134.7, 132.7, 128.4, 125.0, 121.0, 113.0, 112.5, 106.2, 83.4, 78.4, 64.1, 55.8, 55.2, 50.8, 41.7, 34.3, 29.9, 20.1 ppm. **IR** (KBr): ν_{max} = 3478, 3413, 2923, 1750, 1265, 799, 620 cm^{-1}. **HRMS** (m/z): Calcd for $C_{20}H_{20}BrN_2O_4$, $[M+H]^+$, 431.0601; found 431.0599. $[\alpha]_D^{25}$: –101.5 (*c* 0.20, $CHCl_3$).

4-((6*S*, 10*S*)-3-bromo-9-oxo-6,7,8,9,10,11-hexahydro-5*H*-6,10-epiminocycloocta[*b*]indol-12-yl)but-2-yn-1-yl methyl carbonate (13g)

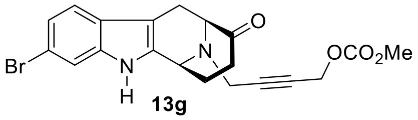

13g

Purification by column chromatography on silica gel (petroleum ether/EtOAc = 2/1, R_f = 0.2) afforded **13g** (27.5 mg, 64%) as a yellow oil.

^{1}H NMR (400 MHz, $CDCl_3$): δ 8.01 (s, 1H), 7.48 (s, 1H), 7.30 (d, *J* = 8.4 Hz, 1H), 7.21 (d, *J* = 8.4 Hz, 1H), 4.74 (s, 2H), 4.29 (d, *J* = 3.8 Hz, 1H), 3.87 (d, *J* = 6.5 Hz, 1H), 3.81 (s, 3H), 3.46 (q, *J* = 16.3 Hz, 2H), 3.11 (dd, *J* = 17.0, 6.7 Hz, 1H), 2.65 (d, *J* = 17.0 Hz, 1H), 2.55–2.40 (m, 2H). 2.10–2.01 (m, 2H) ppm. **^{13}C NMR** (100 MHz, $CDCl_3$): δ 209.3, 155.3, 136.9, 132.0, 125.7, 123.3, 119.6 115.7, 114.1, 106.9, 83.4, 78.5, 64.2, 55.9, 55.3, 50.7, 41.8, 34.3, 30.0, 20.1 ppm. **IR** (KBr): ν_{max} = 3650, 3311, 1748, 1267, 697, 554 cm^{-1}. **HRMS** (m/z): Calcd for $C_{20}H_{20}BrN_2O_4$, $[M+H]^+$, 431.0601; found 431.0599. $[\alpha]_D^{25}$: –140 (*c* 0.15, $CHCl_3$).

4-((6*S*, 10*S*)-2-chloro-9-oxo-6,7,8,9,10,11-hexahydro-5*H*-6,10-epiminocycloocta[*b*]indol-12-yl)but-2-yn-1-yl methyl carbonate (13i)

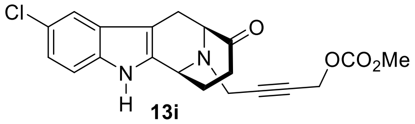

13i

Purification by column chromatography on silica gel (petroleum ether/EtOAc = 2/1, R_f = 0.30) afforded **13i** (23.6 mg, 61%) as a yellow oil.

^{1}H NMR (400 MHz, $CDCl_3$): δ 8.23 (s, 1H), 7.39 (s, 1H), 7.22 (d, *J* = 8.6 Hz, 1H), 7.11 (d, *J* = 8.6 Hz, 1H), 4.73 (s, 2H), 4.27 (s, 1H), 3.86 (d, *J* = 6.5 Hz, 1H), 3.80 (s, 3H), 3.43 (q, *J* = 16.4 Hz, 2H), 3.07 (dd, *J* = 17.0, 6.6 Hz, 1H), 2.62 (d, *J* = 17.0 Hz, 1H), 2.52–1.40 (m, 2H). 2.09–1.98 (m, 2H) ppm. **^{13}C NMR** (100 MHz, $CDCl_3$): δ 209.4, 155.3, 134.4, 132.9, 127.8, 125.6, 122.5, 117.9, 112.1, 106.3, 83.4, 78.4, 64.2, 55.8, 55.3, 50.8, 41.7 34.3, 30.0, 20.1 ppm. **IR** (KBr): ν_{max} = 3641, 3314, 1661, 1592, 682, 552 cm^{-1}. **HRMS** (m/z): Calcd for $C_{20}H_{20}ClN_2O_4$, $[M+H]^+$, 387.1106; found 387.1104. $[\alpha]_D^{25}$: –69.8 (*c* 0.21, $CHCl_3$).

4-((6*S*, 10*S*)-3-fluoro-9-oxo-6,7,8,9,10,11-hexahydro-5*H*-6,10-epiminocycloocta[*b*]indol-12-yl)but-2-yn-1-yl methyl carbonate (13j)

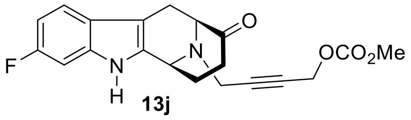

13j

Purification by column chromatography on silica gel (petroleum ether/EtOAc = 2/1, R_f = 0.20) afforded **13j** (22.2 mg, 60%) as a yellow oil.

^{1}H NMR (400 MHz, $CDCl_3$): δ 8.15 (s, 1H), 7.33 (dd, J = 8.5, 5.3 Hz, 1H), 7.01 (dd, J = 9.6, 2.0 Hz, 1H), 6.87 (td, J = 9.6, 2.1 Hz, 1H), 4.74 (s, 2H), 4.27 (s, 1H), 3.86 (d, J = 6.4 Hz, 1H), 3.81 (s, 3H), 3.44 (q, J = 16.3 Hz, 2H), 3.10 (dd, J = 17.0, 6.6 Hz, 1H), 2.64 (d, J = 17.0 Hz, 1H), 2.52–2.40 (m, 2H), 2.12–2.00 (m, 2H) ppm. **^{13}C NMR** (100 MHz, $CDCl_3$): δ 209.6, 161.2, 158.9, 155.3, 136.0 (d, $J_{C\text{-}F}$ = 12.3 Hz), 131.5 (d, $J_{C\text{-}F}$ = 3.4 Hz), 123.3, 118.9 (d, $J_{C\text{-}F}$ = 10.1 Hz), 108.5, 108.3, 106.6, 97.8, 97.6, 83.5, 78.4, 64.2, 55.8, 55.3, 50.8, 41.7, 34.3, 30.0, 20.2 ppm. **IR** (KBr): v_{max} = 3659, 3311, 1751, 1268, 687, 556 cm^{-1}. **HRMS** (m/z): Calcd for $C_{20}H_{20}FN_2O_4$, $[M+H]^+$, 371.1402; found 371.1400. $[\alpha]_D^{25}$: –43.3 (c 0.18, $CHCl_3$).

4-((6*S*, 10*S*)-2-methoxy-9-oxo-6,7,8,9,10,11-hexahydro-5*H*-6,10-epiminocycloocta[*b*]indol-12-yl)but-2-yn-1-yl methyl carbonate (13l)

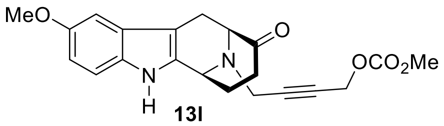

Purification by column chromatography on silica gel (petroleum ether/EtOAc = 2/1, R_f = 0.30) afforded **13l** (19.9 mg, 52%) as a yellow oil.

^{1}H NMR (400 MHz, $CDCl_3$): δ 7.88 (s, 1H), 7.22 (d, J = 8.7 Hz, 1H), 6.89 (d, J = 2.1 Hz, 1H), 6.83 (dd, J = 8.7, 2.3 Hz, 1H), 4.74 (s, 2H), 4.27 (s, 1H), 3.88 (d, J = 6.5 Hz, 1H), 3.84 (s, 3H), 3.81 (s, 3H), 3.46 (q, J = 16.3 Hz, 2H), 3.11 (dd, J = 17.0, 6.7 Hz, 1H), 2.64 (d, J = 16.9 Hz, 1H), 2.52–2.39 (m, 2H), 2.12–2.02 (m, 2H) ppm. **^{13}C NMR** (100 MHz, $CDCl_3$): δ 209.5, 155.3, 154.4, 131.9, 131.0, 127.2, 112.2, 111.8, 106.4, 100.4, 83.6, 78.4, 64.3, 56.0, 55.9, 55.3, 50.9, 41.7, 34.4, 30.2, 20.3 ppm. **IR** (KBr): v_{max} = 3483, 1961, 1728, 1638, 1451, 1160, 1044, 741 cm^{-1}. **HRMS** (m/z): Calcd for $C_{21}H_{23}N_2O_5$, $[M+H]^+$, 383.1601; found 383.1609. $[\alpha]_D^{25}$: –179.2 (c 0.12, $CHCl_3$).

2.11 General procedure for the preparation of 14

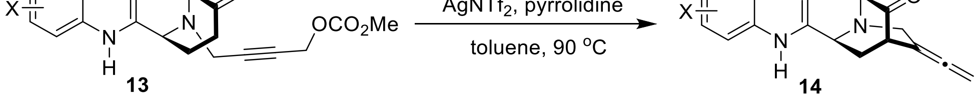

To a solution of **13** (1.0 mmol) in dry toluene (50 mL) were added $AgNTf_2$ (1.1 mmol) and pyrrolidine (5.0 mmol) at room temperature. The resulting mixture was stirred at 90 ℃ for 20 min. The mixture was then cooled to room temperature, and filtered through a pad of Celite. The filtrate was concentrated in vacuo and the residue was purified by flash column chromatography on silica gel to afford the pure product **14**.

(6*S*, 10*R*, 11a*S*)-9-vinylidene-5,6,9,10,11a,12-hexahydro-6,10-methanoindolo[3,2-*b*]quinolizin-11(8*H*)-one (14a)

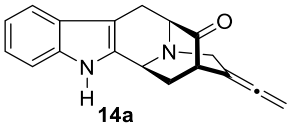

14a

Purification by column chromatography on silica gel (petroleum ether/EtOAc = 2/1, R_f = 0.30) afforded **14a** (215.4 mg, 78%) as a white solid.

^{1}H NMR (400 MHz, $CDCl_3$): δ 7.76 (s, 1H), 7.48 (d, J = 7.7 Hz, 1H), 7.27 (d, J = 7.9 Hz, 1H), 7.18–7.07 (m, 2H), 4.86 (q, J = 4.2 Hz, 2H), 4.27 (dd, J = 9.6, 2.4 Hz, 1H), 3.94 (t, J = 4.4 Hz, 2H), 3.70 (d, J = 6.2 Hz, 1H), 3.31 (dd, J = 15.6, 1.5 Hz, 1H), 3.05–2.96 (m, 2H), 2.61 (ddd, J = 12.2, 9.5, 2.1 Hz, 1H), 2.26–2.17 (m, 1H) ppm. **^{13}C NMR** (100 MHz, $CDCl_3$): δ 216.3, 202.6, 136.4, 136.1, 127.0, 122.2, 119.9, 118.7, 111.0, 105.7, 95.5, 78.5, 64.5, 51.8, 50.5, 46.1, 36.5, 22.7 ppm. **IR** (KBr): ν_{max} = 3451, 1729, 1642, 1455, 1438, 1302, 1263, 1076, 853, 731 cm^{-1}. **HRMS** (ESI): Calcd for $C_{18}H_{17}N_2O$, $[M+H]^+$, 277.1335; found 277.1341. **m. p.**: 269.3–270.1 ℃. **[α]$_D^{25}$**: –137.6 (*c* 0.1, MeOH).

(6*S*, 10*R*, 11a*S*)-1-bromo-9-vinylidene-6,8,9,10,11a,12-hexahydro-6,10-methanoindolo[3,2-*b*]quinolizin-11(5*H*)-one (14e)

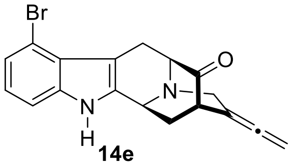

14e

Purification by column chromatography on silica gel (petroleum ether/EtOAc = 3/1, R_f = 0.50) afforded **14e** (208.4 mg, 56%) as a white solid.

^{1}H NMR (400 MHz, $CDCl_3$): δ 8.28 (s, 1H), 7.20 (d, J = 7.7 Hz, 2H), 6.94 (t, J = 7.8 Hz, 1H), 4.89 (d, J = 2.1 Hz, 2H), 4.30 (d, J = 8.7 Hz, 1H), 3.92 (s, 2H), 3.82–3.70 (m, 2H), 3.29 (dd, J = 16.0, 6.1 Hz, 1H), 3.04 (s, 1H), 2.64–2.54 (m, 1H), 2.19 (d, J = 11.4 Hz, 1H) ppm. **^{13}C NMR** (100 MHz, $CDCl_3$): δ 215.6, 202.7, 137.3, 136.9, 126.1, 123.8, 123.1, 114.2, 110.3, 106.4, 95.0, 78.7, 64.4, 51.6, 50.5, 45.9, 36.2, 24.6 ppm. **IR** (KBr): ν_{max} = 3665, 3303, 3195, 1724, 1572, 685, 553 cm^{-1}. **HRMS** (m/z): Calcd for $C_{18}H_{16}BrN_2O$, $[M+H]^+$, 355.0440; found 355.0438. **m.p.**: 279.1–280.2 ℃. $[\alpha]_D^{25}$: –100.0 (*c* 0.13, $CHCl_3$).

(6*S*, 10*R*, 11a*S*)-2-bromo-9-vinylidene-5,6,9,10,11a,12-hexahydro-6,10-methanoindolo[3,2-*b*]quinolizin-11(8*H*)-one (14f)

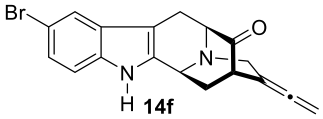

14f

Purification by column chromatography on silica gel (petroleum ether/EtOAc = 3/1, R_f = 0.20) afforded **14f** (234.4 mg, 63%) as a white solid.

^{1}H NMR (400 MHz, $CDCl_3$): δ 7.83 (s, 1H), 7.59 (s, 1H), 7.22 (dd, J = 8.6, 1.6 Hz, 1H), 7.12 (d, J = 8.6 Hz, 1H), 4.86 (d, J = 2.9 Hz, 2H), 4.25 (d, J = 8.2 Hz, 1H), 3.93 (t, J = 4.1 Hz, 2H), 3.69 (d, J = 5.9 Hz, 1H), 3.25 (d, J = 15.3 Hz, 1H), 3.02 (d, J = 1.4 Hz, 1H), 2.94 (dd, J = 15.6, 6.3 Hz, 1H), 2.61 (t, J = 10.5 Hz, 1H), 2.18 (d, J = 12.8 Hz, 1H) ppm. **^{13}C NMR** (100 MHz, $CDCl_3$): δ 216.0, 202.6, 137.4, 135.1, 128.8, 124.9, 121.4, 113.1, 112.4, 105.5, 95.2, 78.6, 64.3, 51.8, 50.4, 45.9, 36.4, 22.5 ppm. **IR** (KBr): v_{max} = 3673, 3302, 2924, 1725, 1592, 800 cm^{-1}. **HRMS** (m/z): Calcd for $C_{18}H_{16}BrN_2O$, $[M+H]^+$, 355.0441; found 355.0439. **m.p.**:185.3–186.7 ℃. $[\alpha]_D^{25}$: –35.0 (c 0.1, $CHCl_3$).

(6*S*, 10*R*, 11a*S*)-3-bromo-9-vinylidene-5,6,9,10,11a,12-hexahydro-6,10-methanoindolo[3,2-*b*]quinolizin-11(8*H*)-one (14g)

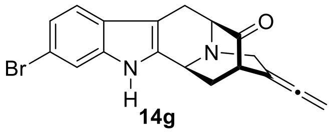

14g

Purification by column chromatography on silica gel (petroleum ether/EtOAc = 3/1, R_f = 0.30) afforded **14g** (178.6 mg, 48%) as a white solid.

^{1}H NMR (400 MHz, $CDCl_3$): δ 7.94 (s, 1H), 7.36 (s, 1H), 7.31 (d, J = 8.2 Hz, 1H), 7.18 (d, J = 7.9 Hz, 1H), 4.86 (s, 2H), 4.17 (d, J = 8.9 Hz, 1H), 3.91 (s, 2H), 3.69 (d, J = 5.3 Hz, 1H), 3.26 (d, J = 15.6 Hz, 1H), 3.04–2.88 (m, 2H), 2.57 (t, J = 10.9 Hz, 1H), 2.16 (d, J = 12.6 Hz, 1H) ppm. **^{13}C NMR** (100 MHz, $CDCl_3$): δ 216.1, 202.6, 137.2, 136.7, 125.9, 123.2, 119.9, 115.6, 114.1, 105.8, 95.2, 78.6, 64.3, 51.7, 50.3, 45.9, 36.4, 22.5 ppm. **IR** (KBr): v_{max} = 3641, 3319, 1661, 1592, 679, 552 cm^{-1}. **HRMS** (m/z): Calcd for $C_{18}H_{16}BrN_2O$, $[M+H]^+$, 355.0441; found: 355.0439. **m.p.**: 133.2–133.5 ℃. $[\alpha]_D^{25}$: –18.1 (c 0.21, $CDCl_3$).

(6*S*, 10*R*, 11a*S*)-2-chloro-9-vinylidene-5,6,9,10,11a,12-hexahydro-6,10-methanoindolo[3,2-*b*]quinolizin-11(8*H*)-one (14i)

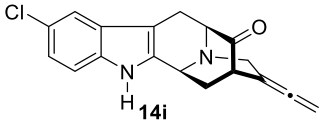

14i

Purification by column chromatography on silica gel (petroleum ether/EtOAc = 3/1, R_f = 0.20) afforded **14i** (154.2 mg, 47%) as a white solid.

^{1}H NMR (400 MHz, $CDCl_3$): δ 8.03 (s, 1H), 7.43 (s, 1H), 7.08 (dt, J = 8.6, 5.1 Hz, 2H), 4.86 (d, J = 4.3 Hz, 2H), 4.17 (d, J = 8.3 Hz, 1H), 3.98–3.86 (m, 2H), 3.68 (d, J = 5.9 Hz, 1H), 3.24 (d, J = 15.5 Hz, 1H), 3.03–2.89 (m, 2H),

2.59–2.49 (m, 1H), 2.13 (d, *J* = 12.8 Hz, 1H) ppm. **^{13}C NMR** (100 MHz, $CDCl_3$): δ 216.2, 202.6, 137.6, 134.8, 128.1, 125.6, 122.4, 118.2, 112.0, 105.4, 95.1, 78.6, 64.3, 51.7, 50.4, 45.9, 36.4, 22.5 ppm. **IR** (KBr): ν_{max} = 3640, 3318, 1660, 1593, 684, 552 cm^{-1}. **HRMS** (m/z): Calcd for $C_{18}H_{16}ClN_2O$, $[M+H]^+$, 311.0946; found 311.0943. **m.p.**: 115.5–117.2 ℃. **$[\alpha]_D^{25}$**: –41.8 (*c* 0.11, $CHCl_3$).

(6*S*, 10*R*, 11a*S*)-3-fluoro-9-vinylidene-5,6,9,10,11a,12-hexahydro-6,10-methanoindolo[3,2-*b*]quinolizin-11(8*H*)-one (14j)

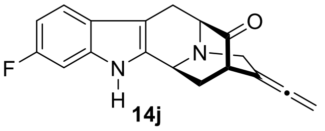

14j

Purification by column chromatography on silica gel (petroleum ether/EtOAc = 3/1, R_f = 0.50) afforded **14j** (231.0 mg, 74%) as a white solid.

^{1}H NMR (400 MHz, $CDCl_3$): δ 7.81 (s, 1H), 7.36 (dd, *J* = 7.9, 5.6 Hz, 1H), 6.94 (d, *J* = 9.4 Hz, 1H), 6.85 (t, *J* = 8.7 Hz, 1H), 4.85 (d, *J* = 2.8 Hz, 2H), 4.23 (d, *J* = 9.0 Hz, 1H), 3.93 (s, 2H), 3.69 (d, *J* = 5.7 Hz, 1H), 3.26 (d, *J* = 15.6 Hz, 1H), 3.05–2.90 (m, 2H), 2.59 (t, *J* = 11.0 Hz, 1H), 2.20 (d, *J* = 12.7 Hz, 1H) ppm. **^{13}C NMR** (100 MHz, $CDCl_3$): δ 216.2, 202.6, 161.2, 136.4, 123.6, 119.3 (d, $J_{C\text{-}F}$ = 10.1 Hz), 108.5, 108.3, 105.8, 97.8 (d, $J_{C\text{-}F}$ = 26.0 Hz), 95.4, 78.5, 64.4, 51.8, 50.5, 46.0, 36.5, 22.6 ppm. **IR** (KBr): ν_{max} = 3660, 3310, 1723, 1580, 728, 555 cm^{-1}. **HRMS** (m/z): Calcd for $C_{18}H_{16}FN_2O$, $[M+H]^+$, 295.1241; found: 295.1239. **m.p.**: 201.4–202.7 ℃. **$[\alpha]_D^{25}$**: –117.6 (*c* 0.17, $CHCl_3$).

(6*S*, 10*R*, 11a*S*)-2-methoxy-9-vinylidene-5,6,9,10,11a,12-hexahydro-6,10-methanoindolo[3,2-*b*]quinolizin-11(8*H*)-one (14l)

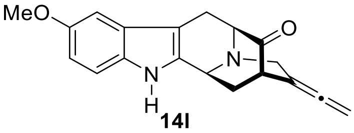

14l

Purification by column chromatography on silica gel (petroleum ether/EtOAc = 3/1, R_f = 0.20) afforded **14l** (168.4 mg, 55%) as a colorless oil.

^{1}H NMR (400 MHz, $CDCl_3$): δ 7.72 (s, 1H), 7.15 (d, *J* = 8.7 Hz, 1H), 6.92 (s, 1H), 6.80 (d, *J* = 8.7 Hz, 1H), 4.93–4.80 (m, 2H), 4.26 (d, *J* = 9.2 Hz, 1H), 3.93 (s, 2H), 3.83 (s, 3H), 3.72 (d, *J* = 5.9 Hz, 1H), 3.27 (d, *J* = 15.5 Hz, 1H), 3.05–2.93 (m, 2H), 2.64–2.56 (m, 1H), 2.18 (d, *J* = 12.9 Hz, 1H) ppm. **^{13}C NMR** (100 MHz, $CDCl_3$): δ 215.9, 202.6, 154.3, 136.6, 131.4, 127.4, 112.1, 111.7, 105.4, 100.7, 95.1, 78.6, 64.4, 55.9, 51.7, 50.5, 45.9, 36.4, 22.6 ppm. **IR** (KBr): ν_{max} = 2920, 1724, 1615, 1199, 1137, 1056 cm^{-1}. **HRMS** (m/z): Calcd for $C_{19}H_{19}N_2O_2$, $[M+H]^+$, 307.1447; found: 307.1442. **$[\alpha]_D^{25}$**: –209.0 (*c* 0.1, $CHCl_3$).

2.12 General procedure for the preparation of 14-1

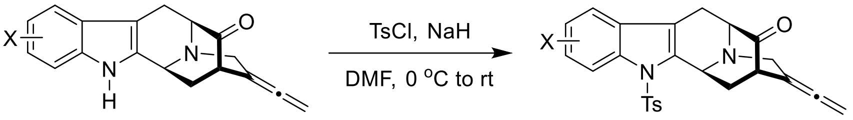

To a mixture of NaH (60% dispersion on mineral oil, 8 mg, 0.2 mmol) in dry DMF (1 mL) was added **14** (0.1 mmol) at 0 ℃. The reaction mixture was stirred at room temperature for 30 min, and then was added with TsCl (0.12 mmol). After **14** was fully consumed as judged by TLC analysis, the reaction mixture was extracted with EtOAc (3 ×10 mL), and was washed with brine (3 ×10 mL). The combined organic extract was dried over Na_2SO_4, filtered, and concentrated. The crude product was purified by flash column chromatography on silica gel to afford **14-1**.

(6*S*, 10*R*, 11a*S*)-1-bromo-5-tosyl-9-vinylidene-5,6,9,10,11a,12-hexahydro-6,10-methanoindolo[3,2-*b*]quinolizin-11(8*H*)-one (14-1e)

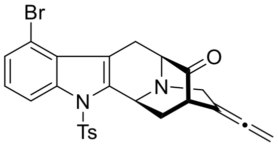

14-1e

Purification by column chromatography on silica gel (petroleum ether/EtOAc = 2/1, R_f = 0.60) afforded **14-1e** (26.3 mg, 50%) as a white solid.

^{1}H NMR (400 MHz, $CDCl_3$): δ 8.03 (d, J = 8.3 Hz, 1H), 7.61 (d, J = 8.3 Hz, 2H), 7.35 (d, J = 7.7 Hz, 1H), 7.21 (d, J = 8.1 Hz, 2H), 7.09 (t, J = 8.1 Hz, 1H), 4.93–4.80 (m, 3H) 4.03 (dt, J = 16.5, 4.8 Hz, 1H), 3.91 (dt, J = 16.5, 3.6 Hz, 1H), 3.66 (dd, J = 20.2, 11.9 Hz, 2H), 3.15 (dd, J = 16.7, 6.8 Hz, 1H), 3.06 (s, 1H), 2.90–2.81 (m, 1H), 2.34 (s, 3H), 2.29 (d, J = 10.7 Hz, 1H) ppm. **^{13}C NMR** (100 MHz, $CDCl_3$): δ 215.9, 202.7, 145.5, 138.7, 137.4, 135.5, 130.3, 128.2, 128.0, 126.4, 125.6, 114.3, 113.6, 94.9, 78.6, 62.9, 51.9, 51.1, 46.0, 37.1, 24.9, 21.7 ppm. **IR** (KBr): ν_{max} = 3685, 3126, 1728, 1564, 579 cm^{-1}. **HRMS** (m/z): Calcd for $C_{25}H_{22}BrN_2O_3S$, $[M+H]^+$, 509.0529; found: 509.0526. **m.p.**: 202.7–203.9 ℃. **$[\alpha]_D^{25}$**: –78.7 (c 0.08, $CHCl_3$).

(6*S*, 10*R*, 11a*S*)-2-bromo-5-tosyl-9-vinylidene-5,6,9,10,11a,12-hexahydro-6,10-methanoindolo[3,2-*b*]quinolizin-11(8*H*)-one (14-1f)

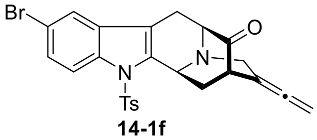

14-1f

Purification by column chromatography on silica gel (petroleum ether/EtOAc = 2/1, R_f = 0.30) afforded **14-1f** (31.6 mg, 62%) as a white solid.

^{1}H NMR (400 MHz, $CDCl_3$): δ 7.92 (d, J = 8.8 Hz, 1H), 7.61 (d, J = 8.3 Hz, 2H), 7.50 (d, J = 1.6 Hz, 1H), 7.37

(dd, J = 8.8, 1.8 Hz, 1H), 7.21 (d, J = 8.1 Hz, 2H), 4.93–4.77 (m, 3H), 4.03 (dt, J = 16.4, 4.8 Hz, 1H), 3.92 (dt, J = 16.5, 3.6 Hz, 1H), 3.64 (d, J = 6.4 Hz, 1H), 3.14 (d, J = 16.1 Hz, 1H), 3.08–3.01 (m, 1H), 2.89–2.78 (m, 2H), 2.34 (s, 3H), 2.31–2.24 (m, 1H) ppm. **^{13}C NMR** (100 MHz, $CDCl_3$): δ 215.6, 202.7, 149.4, 145.5, 138.8, 135.5, 135.2, 131.1, 130.3, 127.8, 126.4, 121.8, 117.4, 116.1, 113.5, 94.8, 78.6, 62.9, 52.1, 51.2, 46.1, 37.2, 22.3, 21.7 ppm. **IR** (KBr): ν_{max} = 3649, 3310, 1682, 1589, 678, 550 cm^{-1}. **HRMS** (m/z): Calcd for $C_{25}H_{22}BrN_2O_3S$, $[M+H]^+$, 509.0529; found: 509.0525. **m.p.**: 169.5–171.8 ℃. $[\alpha]_D^{25}$: +90.9 (c 0.11, $CHCl_3$).

(6*S*, 10*R*, 11a*S*)-3-bromo-5-tosyl-9-vinylidene-5,6,9,10,11a,12-hexahydro-6,10-methanoindolo[3,2-*b*]quinolizin-11(8*H*)-one (14-1g)

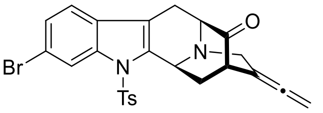

14-1g

Purification by column chromatography on silica gel (petroleum ether/EtOAc = 2/1, R_f = 0.30) afforded **14-1g** (33.1 mg, 65%) as a white solid.

^{1}H NMR (400 MHz, $CDCl_3$): δ 8.24 (s, 1H), 7.64 (d, J = 8.0 Hz, 2H), 7.35 (d, J = 8.1 Hz, 1H), 7.24 (t, J = 7.5 Hz, 3H), 4.93–4.82 (m, 2H), 4.78 (d, J = 8.9 Hz, 1H), 3.97 (dd, J = 42.7, 16.4 Hz, 2H), 3.64 (d, J = 6.1 Hz, 1H), 3.16 (d, J = 16.1 Hz, 1H), 3.04 (s, 1H), 2.90–2.77 (m, 2H), 2.35 (s, 3H), 2.27 (d, J = 13.7 Hz, 1H) ppm. **^{13}C NMR** (100 MHz, $CDCl_3$): δ 215.7, 202.7, 138.0, 137.1, 135.5, 130.4, 128.2, 127.2, 126.4, 119.9, 118.6, 117.7, 113.8, 94.8, 78.7, 62.8, 52.1, 51.1, 46.1, 37.2, 22.3, 21.8 ppm. **IR** (KBr): ν_{max} = 3640, 3323 1660, 1593, 679, 551 cm^{-1}. **HRMS** (m/z): Calcd for $C_{25}H_{22}BrN_2O_3S$, $[M+H]^+$, 509.0529; found: 509.0526. **m.p.**: 202.7–203.9 ℃. $[\alpha]_D^{25}$: +37.1 (c 0.14, $CHCl_3$).

(6*S*, 10*R*, 11a*S*)-2-chloro-5-tosyl-9-vinylidene-5,6,9,10,11a,12-hexahydro-6,10-methanoindolo[3,2-*b*]quinolizin-11(8*H*)-one (14-1i)

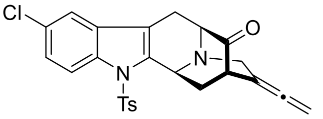

14-1i

Purification by column chromatography on silica gel (petroleum ether/EtOAc = 2/1, R_f = 0.30) afforded **14-1i** (33.1 mg, 65%) as a white solid.

^{1}H NMR (400 MHz, $CDCl_3$): δ 7.97 (d, J = 8.9 Hz, 1H), 7.61 (d, J = 8.3 Hz, 2H), 7.34 (d, J = 1.7 Hz, 1H), 7.25–7.17 (m, 3H), 4.92–4.76 (m, 3H), 4.03 (dt, J = 16.5, 4.8 Hz, 1H), 3.92 (dt, J = 16.5, 3.5 Hz, 1H), 3.64 (d, J = 6.4 Hz, 1H), 3.14 (d, J = 16.1 Hz, 1H), 3.04 (d, J = 0.9 Hz, 1H), 2.89–2.78 (m, 2H), 2.33 (s, 3H), 2.28 (d, J = 13.7 Hz, 1H) ppm. **^{13}C NMR** (100 MHz, $CDCl_3$): δ 215.4, 202.7, 145.4, 138.8, 135.4, 134.8, 130.6, 130.2, 129.7, 126.4,

125.1, 118.7, 115.6, 113.6, 94.7, 78.6, 62.8, 51.9, 51.1, 46.0, 37.1, 22.2, 21.7 ppm. **IR** (KBr): v_{max} = 3660, 3311, 3205, 1661, 1592, 681, 552 cm^{-1}. **HRMS** (m/z): Calcd for $C_{25}H_{22}ClN_2O_3S$, $[M+H]^+$, 465.1034; found: 465.1030. **m.p.**: 150.3–151.8 ℃. $[\alpha]_D^{25}$: +41.5 (*c* 0.11, CHCl$_3$).

(6*S*, 10*R*, 11a*S*)-3-fluoro-5-tosyl-9-vinylidene-5,6,9,10,11a,12-hexahydro-6,10-methanoindolo[3,2-*b*]quinolizin-11(8*H*)-one (14-1j)

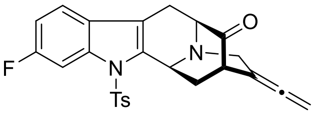

14-1j

Purification by column chromatography on silica gel (petroleum ether/EtOAc = 2/1, R_f = 0.35) afforded **14-1j** (31.5 mg, 70%) as a white solid.

^{1}H NMR (400 MHz, CDCl$_3$): δ 7.79 (d, *J* = 9.9 Hz, 1H), 7.64 (d, *J* = 8.0 Hz, 2H), 7.28 (dd, *J* = 9.3, 6.7 Hz, 1H), 7.21 (d, *J* = 8.0 Hz, 2H), 6.98 (t, *J* = 8.3 Hz, 1H), 4.91–4.76 (m, 3H), 4.07–3.85 (m, 2H), 3.64 (d, *J* = 6.3 Hz, 1H), 3.16 (d, *J* = 16.2 Hz, 1H), 3.04 (s, 1H), 2.91–2.77 (m, 2H), 2.39–2.23 (m, 4H) ppm. **^{13}C NMR** (100 MHz, CDCl$_3$): δ 215.8, 202.7, 162.3, 159.9, 145.4, 137.7, 136.7, 135.5, 130.3, 126.4, 125.6, 119.6 (d, $J_{C\text{-}F}$ = 9.8 Hz), 113.8, 112.2, 111.9, 102.4, 102.1, 94.9, 78.6, 62.9, 52.1, 51.2, 46.1, 37.2, 22.3, 21.7 ppm. **IR** (KBr): v_{max} = 3640, 3311, 1660, 1592, 680, 550 cm^{-1}. **HRMS** (m/z): Calcd for $C_{25}H_{22}FN_2O_3S$, $[M+H]^+$, 449.1330; found: 449.1327. **m.p.**: 151.3–152.8 ℃. $[\alpha]_D^{25}$: –77.8 (*c* 0.09, CHCl$_3$).

2.13 General procedure for the preparation of 15a, 15ae, 15af, 15ag, 15ai, and 15aj

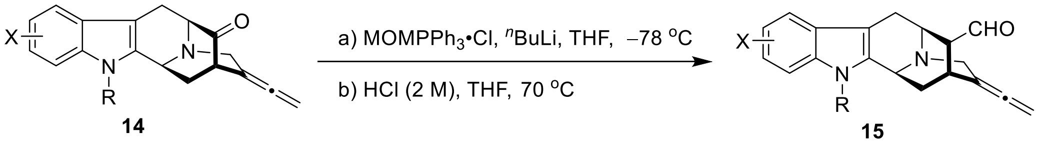

14a, X = H, R = H, **14-1e**, X = 4-Br, R = Ts
14-1f, X = 5-Br, R = Ts, **14-1g**, X = 6-Br, R = Ts
14-1i, X = 5-Cl, R = Ts, **14-1j**, X = 6-F, R = Ts

To a solution of MOMPPh$_3$ Cl (0.5 mmol) in THF (2 mL) was added dropwise ^{n}BuLi (2.0 M in hexane, 0.22 mL, 0.44 mmol) at –78 ℃. The reaction mixture was stirred for 30 min, and then was added with a solution of substrate **14** (0.05 mmol) in THF (1 mL). The mixture was stirred for an additional 1 h before H_2O (15 mL) was added slowly to quench the reaction. The resulting solution was extracted with EtOAc (3 × 15 mL). The organic phase was washed with brine (3 × 10 mL), dried over Na_2SO_4, filtered, and concentrated under reduced pressure. The crude product was directly used in the next reaction.

To a mixture of the above crude product in THF (1 mL) was added HCl (2 M, 1 mL). The mixture was stirred at 70 ℃ for 5 h before a saturated aqueous solution of NaCl (2 mL) was added slowly to quench the reaction. The

resulting mixture was extracted with EtOAc (3 × 15 mL). The combined organic phase was washed with brine (1 ×10 mL), dried over Na_2SO_4, filtered, and concentrated. The crude product was purified by flash column chromatography on silica gel to afford **15**.

(6*S*, 10*S*, 11*S*, 11a*S*)-9-vinylidene-5,6,8,9,10,11,11a,12-octahydro-6,10-methanoindolo[3,2-*b*]quinolizine-11-carbaldehyde (15a)

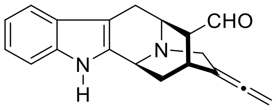

15a

Purification by column chromatography on silica gel (petroleum ether/EtOAc = 2/1, R_f = 0.20) afforded **15a** (14.5 mg, 50%). The spectroscopic data of **15a** are in agreement with the literature values.[1]

(6*S*, 10*S*, 11*S*, 11a*S*)-1-bromo-5-tosyl-9-vinylidene-5,6,8,9,10,11,11a,12-octahydro-6,10-methanoindolo[3,2-*b*]quinolizine-11-carbaldehyde (15ae)

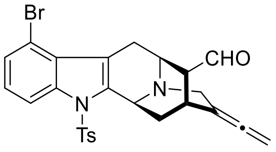

15ae

Purification by column chromatography on silica gel (petroleum ether/EtOAc = 10/1, R_f = 0.30) afforded **15ae** (15.2 mg, 58%) as a white solid.

^{1}H NMR (400 MHz, $CDCl_3$): δ 9.71 (s, 1H), 8.07 (d, *J* = 8.4 Hz, 1H), 7.62 (d, *J* = 8.3 Hz, 2H), 7.35 (d, *J* = 7.8 Hz, 1H), 7.20 (d, *J* = 8.1 Hz, 2H), 7.09 (t, *J* = 8.1 Hz, 1H), 4.75 (t, *J* = 4.2 Hz, 2H), 4.70 (d, *J* = 8.9 Hz, 1H), 3.76 (t, *J* = 4.2 Hz, 2H), 3.70 (t, *J* = 6.2 Hz, 1H), 3.29 (dd, *J* = 16.8, 5.5 Hz, 1H), 3.05 (d, *J* = 16.8 Hz, 1H), 2.97 (s, 1H), 2.48 (dd, *J* = 17.1, 6.4 Hz, 1H), 2.37–2.28 (m, 4H), 1.93 (d, *J* = 13.5 Hz, 1H) ppm. **^{13}C NMR** (100 MHz, $CDCl_3$): δ 202.5, 200.6, 145.5, 140.1, 137.4, 135.5, 130.3, 128.7, 128.0, 126.5, 125.4, 114.2, 113.7, 113.3, 94.9, 77.4, 54.1, 52.3, 51.1, 49.2, 34.6, 30.4, 30.1, 21.7 ppm. **IR** (KBr): ν_{max} = 3681, 3159, 1723, 1569, 574 cm^{-1}. **HRMS** (m/z): Calcd for $C_{26}H_{22}BrN_2O_3S$, $[M–H]^-$, 521.0532; found: 521.0522. **m.p.**: 216.3–217.5 ℃. $[\alpha]_D^{25}$: –27.8 (*c* 0.09, $CHCl_3$).

(6*S*, 10*S*, 11*S*, 11a*S*)-2-bromo-5-tosyl-9-vinylidene-5,6,8,9,10,11,11a,12-octahydro-6,10-methanoindolo[3,2-*b*]quinolizine-11-carbaldehyde (15af)

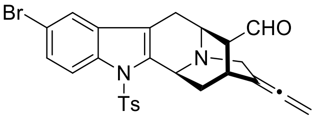

15af

Purification by column chromatography on silica gel (petroleum ether/EtOAc = 10/1, R_f = 0.20) afforded **15af** (19.1 mg, 75%) as a white solid.

^{1}H NMR (400 MHz, $CDCl_3$): δ 9.69 (s, 1H), 7.96 (d, J = 8.8 Hz, 1H), 7.61 (d, J = 8.0 Hz, 2H), 7.47 (s, 1H), 7.37 (d, J = 8.6 Hz, 1H), 7.19 (d, J = 7.9 Hz, 2H), 4.75 (s, 2H), 4.67 (d, J = 9.0 Hz, 1H), 3.76 (s, 2H), 3.73–3.65 (m, 1H), 3.05–2.93 (m, 2H), 2.54–2.40 (m, 2H), 2.33 (s, 3H), 2.25 (d, J = 6.9 Hz, 1H), 1.91 (d, J = 13.4 Hz, 1H) ppm. **^{13}C NMR** (100 MHz, $CDCl_3$): δ 202.5, 200.6, 145.4, 140.2, 135.4, 135.2, 131.8, 130.3, 127.6, 126.4, 121.5, 117.3, 116.1, 112.5, 94.8, 77.6, 53.9, 52.4, 51.2, 49.3, 34.1, 30.4, 27.4, 21.7 ppm. **IR** (KBr): ν_{max} = 3640, 3310, 2925, 1718, 1593, 673, 547 cm^{-1}. **HRMS** (m/z): Calcd for $C_{26}H_{22}BrN_2O_3S$, $[M–H]^-$, 521.0532; found: 521.0517. **m.p.**: 121.5–123.2 ℃. $[\alpha]_D^{25}$: +21.5 (c 0.13, $CHCl_3$).

(6*S*, 10*S*, 11*S*, 11a*S*)-3-bromo-5-tosyl-9-vinylidene-5,6,8,9,10,11,11a,12-octahydro-6,10-methanoindolo[3,2-*b*]quinolizine-11-carbaldehyde (15ag)

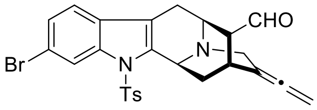

15ag

Purification by column chromatography on silica gel (petroleum ether/EtOAc = 10/1, R_f = 0.20) afforded **15ag** (18.1 mg, 71%) as a white solid.

^{1}H NMR (400 MHz, $CDCl_3$): δ 9.70 (s, 1H), 8.27 (d, J = 1.3 Hz, 1H), 7.64 (d, J = 8.3 Hz, 2H), 7.35 (dd, J = 8.3, 1.5 Hz, 1H), 7.25–7.17 (m, 3H), 4.76 (t, J = 4.2 Hz, 2H), 4.64 (d, J = 8.2 Hz, 1H), 3.77 (t, J = 4.3 Hz, 2H), 3.69 (t, J = 6.2 Hz, 1H), 3.05–2.96 (m, 2H), 2.51–2.43 (m, 2H), 2.34 (s, 3H), 2.26 (d, J = 7.1 Hz, 1H), 1.95–1.88 (m, 1H) ppm. **^{13}C NMR** (100 MHz, $CDCl_3$): δ 202.6, 200.6, 145.5, 139.3, 137.1, 135.5, 130.3, 128.8, 127.1, 126.4, 119.8, 118.3, 117.7, 112.8, 94.8, 77.6, 53.9, 52.4, 51.1, 49.3, 34.1, 30.4, 27.4, 21.8 ppm. **IR** (KBr): ν_{max} = 3640, 3326, 1660, 1594, 673, 550 cm^{-1}. **HRMS**(m/z): Calcd for $C_{26}H_{22}BrN_2O_3S$, $[M–H]^-$, 521.0532; found: 521.0516. **m.p.**: 190.1–190.8 ℃. $[\alpha]_D^{25}$: +27.3 (c 0.11, $CHCl_3$).

(6*S*, 10*S*, 11*S*, 11a*S*)-2-chloro-5-tosyl-9-vinylidene-5,6,8,9,10,11,11a,12-octahydro-6,10-methanoindolo[3,2-*b*]quinolizine-11-carbaldehyde (15ai)

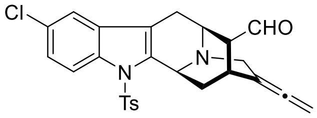

15ai

Purification by column chromatography on silica gel (petroleum ether/EtOAc = 10/1, R_f = 0.40) afforded **15ai** (14.4 mg, 62%) as a white solid.

^{1}H NMR (400 MHz, $CDCl_3$): δ 9.69 (s, 1H), 8.01 (d, J = 8.8 Hz, 1H), 7.61 (d, J = 8.3 Hz, 2H), 7.31 (d, J = 1.8 Hz, 1H), 7.27–7.17 (m, 3H), 4.76 (t, J = 4.2 Hz, 2H), 4.66 (d, J = 8.6 Hz, 1H), 3.77 (t, J = 4.2 Hz, 2H), 3.69 (t, J = 6.1 Hz, 1H), 3.05–2.94 (m, 2H), 2.53–2.41 (m, 2H), 2.33 (s, 3H), 2.25 (d, J = 7.1 Hz, 1H), 1.92 (d, J = 13.5 Hz, 1H)

ppm. **^{13}C NMR** (100 MHz, $CDCl_3$): δ 202.5, 200.6, 147.5, 145.4, 140.3, 135.5, 134.8, 131.3, 130.3, 130.2, 129.7, 126.4, 124.9, 118.5, 115.7, 112.6, 94.8, 77.6, 53.9, 52.5, 51.2, 49.3, 34.1, 30.4, 27.4, 21.7 ppm. **IR** (KBr): ν_{max} = 3649, 3313, 1672, 1590, 681, 551 cm^{-1}. **HRMS** (m/z): Calcd for $C_{26}H_{24}ClN_2O_3S$, $[M+H]^+$, 479.1191; found: 479.1192. **m.p.**: 216.3–217.5 ℃. **$[\alpha]_D^{25}$**: +45.6 (*c* 0.09, $CHCl_3$).

(6*S*, 10*S*, 11*S*, 11a*S*)-3-fluoro-5-tosyl-9-vinylidene-5,6,8,9,10,11,11a,12-octahydro-6,10-methanoindolo[3,2-*b*]quinolizine-11-carbaldehyde (15aj)

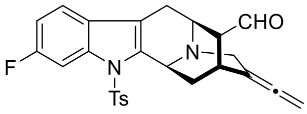

15aj

Purification by column chromatography on silica gel (petroleum ether/EtOAc = 10/1, R_f = 0.40) afforded **15aj** (14.4 mg, 64%) as a white solid.

^{1}H NMR (400 MHz, $CDCl_3$): δ 9.68 (s, 1H), 7.79 (dd, *J* = 10.1, 1.9 Hz, 1H), 7.67 (d, *J* = 8.2 Hz, 2H), 7.28–7.18 (m, 3H), 6.98 (td, *J* = 8.9, 2.1 Hz, 1H), 4.85–4.70 (m, 3H), 3.88–3.74 (m, 3H), 3.12 (d, *J* = 12.3 Hz, 1H), 3.01 (s, 1H), 2.51 (d, *J* = 16.0 Hz, 2H), 2.38–2.24 (m, 4H), 1.99 (d, *J* = 13.6 Hz, 1H) ppm. **^{13}C NMR** (100 MHz, $CDCl_3$): δ 202.7, 200.6, 145.4, 139.0, 136.8, 135.5, 130.3, 126.5, 119.3 (d, $J_{C\text{-}F}$ = 9.7 Hz), 112.8, 111.9 (d, $J_{C\text{-}F}$ = 25.0 Hz), 102.5, 102.2, 94.9, 77.6, 53.9, 52.4, 51.2, 49.4, 34.0, 30.4, 27.5, 21.7 ppm. **IR** (KBr): ν_{max} = 3640, 3314, 1661, 1592, 720, 543 cm^{-1}. **HRMS** (m/z): Calcd for $C_{26}H_{22}FN_2O_3S$, $[M–H]^-$, 461.1343; found: 461.1343. **m.p.**: 150.3 – 151.6 ℃. **$[\alpha]_D^{25}$**: +26.4 (*c* 0.14, $CHCl_3$).

2.14 Preparation of 15ad

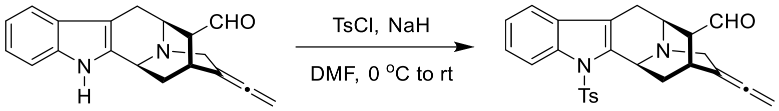

To a mixture of NaH (60% dispersion on mineral oil, 2.9 mg, 0.12 mmol) in dry DMF (1 mL) was added **15a** (29 mg, 0.1 mmol) at 0 ℃. The reaction mixture was stirred at room temperature for 30 min, and then was added with TsCl (28.6 mg, 0.15 mmol) in dry DMF (1.0 mL). The reaction mixture was stirred for an additional 3 h. The reaction mixture was extracted with EtOAc (3 × 10 mL), and was washed with brine (3 × 10 mL). The combined organic extracts were dried over anhydrous Na_2SO_4, filtered, and concentrated in vacuo. The crude product was purified by flash column chromatography on silica gel (petroleum ether/EtOAc = 10/1, R_f = 0.10) to afford **15ad** (15.5 mg, 70%) as a white solid.

^{1}H NMR (400 MHz, $CDCl_3$): δ 9.69 (s, 1H), 8.08 (d, *J* = 8.1 Hz, 1H), 7.63 (d, *J* = 8.0 Hz, 2H), 7.34 (d, *J* = 7.5 Hz, 1H), 7.30–7.20 (m, 2H), 7.17 (d, *J* = 8.0 Hz, 2H), 4.74 (t, *J* = 4.4 Hz, 2H), 4.69 (d, *J* = 8.8 Hz, 1H), 3.77 (t, *J* = 4.4

Hz, 2H), 3.68 (t, J = 6.4 Hz, 1H), 3.03 (dd, J = 16.2, 5.6 Hz, 1H), 2.96 (s, 1H), 2.47 (dd, J = 21.9, 13.6 Hz, 2H), 2.30 (s, 3H), 2.27 (d, J = 7.5 Hz, 1H), 1.92 (d, J = 13.5 Hz, 1H) ppm. **^{13}C NMR** (100 MHz, $CDCl_3$): δ 202.8, 200.5, 145.1, 138.7, 135.7, 130.1, 129.9, 126.4, 124.8, 123.8, 118.7, 114.7, 113.1, 95.0, 53.9, 52.5, 51.2, 49.4, 34.1, 30.4, 27.5, 21.7 ppm. **IR** (KBr): ν_{max} = 2926, 1961, 1717, 1636, 1451, 1371, 1218, 1156 cm^{-1}. **HRMS** (m/z): Calcd for $C_{26}H_{24}N_2NaO_3S$, $[M+Na]^+$, 467.1400; found: 467.1400. **m.p.**: 100.6–104.3 ℃. **$[\alpha]_D^{25}$**: +175.7 (c 0.047, MeOH).

2.15 Preparation of 15al

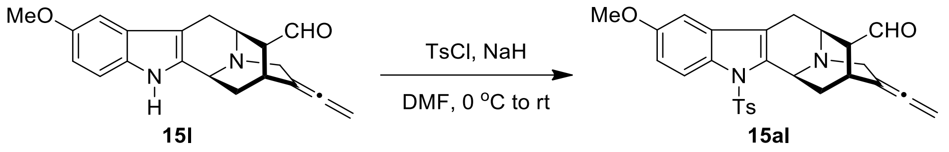

To a mixture of NaH (60% dispersion on mineral oil, 2.9 mg, 0.12 mmol) in dry DMF (1 mL) was added **15l** (32.0 mg, 0.1 mmol) at 0 ℃. The reaction mixture was stirred at room temperature for 30 min, and then was added with TsCl (28.6 mg, 0.15 mmol) in dry DMF (1.0 mL). The reaction mixture was stirred for an additional 3 h. The reaction mixture was extracted with EtOAc (3 × 10 mL), and was washed with brine (3 × 10 mL). The combined organic extracts were dried over Na_2SO_4, filtered, and concentrated in vacuo. The crude product was purified by flash column chromatography on silica gel (petroleum ether/EtOAc = 10/1, R_f = 0.30) to afford **15al** (11.6 mg, 50%) as a white solid.

^{1}H NMR (400 MHz, $CDCl_3$): δ 9.69 (s, 1H), 7.97 (d, J = 9.0 Hz, 1H), 7.60 (d, J = 8.1 Hz, 2H), 7.16 (d, J = 8.0 Hz, 2H), 6.91–6.84 (m, 1H), 6.76 (d, J = 1.8 Hz, 1H), 4.75 (s, 2H), 4.67 (d, J = 9.1 Hz, 1H), 3.84–3.74 (m, 5H), 3.71–3.64 (m, 1H), 3.05–3.90 (m, 2H), 2.50–2.41 (m, 2H), 2.34–2.25 (m, 4H), 1.93 (d, J = 13.3 Hz, 1H) ppm. **^{13}C NMR** (100 MHz, $CDCl_3$): δ 202.9, 200.5, 156.8, 144.9, 139.4, 135.6, 131.0, 130.9, 130.1, 126.4, 115.6, 113.3, 113.2, 101.3, 94.9, 77.6, 55.8, 53.9, 52.4, 51.3, 49.4, 34.0, 30.4, 27.5, 21.7 ppm. **IR** (KBr): ν_{max} = 2920, 1718, 1615, 1460, 1168, 739 cm^{-1}. **HRMS** (m/z): Calcd for $C_{27}H_{27}N_2O_4S$, $[M+H]^+$, 475.1686; found: 475.1688. **m.p.**: 121.5–123.2 ℃. **$[\alpha]_D^{25}$**: +94.2 (c 0.13, $CHCl_3$).

2.16 Preparation of 15am

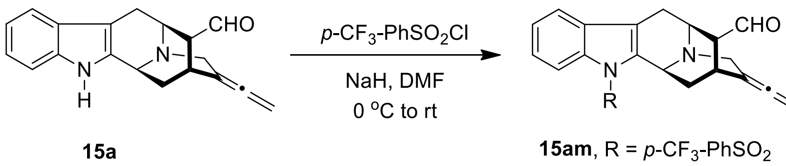

To a mixture of NaH (60% dispersion on mineral oil, 8 mg, 0.2 mmol) in dry DMF (2 mL) was added **15a** (29 mg, 0.1 mmol) at 0 ℃. The reaction mixture was stirred at room temperature for 30 min, and then was added with 4-(trifluoromethyl)benzenesulfonyl chloride (29.3 mg, 0.12 mmol). The reaction mixture was stirred for an additional

2 h. The resulting mixture was extracted with EtOAc (3 × 15 mL), and was washed with brine (3 × 10 mL). The combined organic extract was dried over Na_2SO_4, filtered, and concentrated in vacuo. The crude product was purified by flash column chromatography on silica gel (petroleum ether/EtOAc = 10/1, R_f = 0.20) to afford **15am** (42.8 mg, 83% yield) as a colorless oil.

^{1}H NMR (400 MHz, $CDCl_3$): δ 9.70 (s, 1H), 8.08 (d, J = 8.1 Hz, 1H), 7.87 (d, J = 8.3 Hz, 2H), 7.66 (d, J = 8.3 Hz, 2H), 7.40–7.22 (m, 3H), 4.76 (t, J = 4.1 Hz, 2H), 4.67 (d, J = 8.9 Hz, 1H), 3.78 (t, J = 4.2 Hz, 2H), 3.75–3.67 (m, 1H), 3.05 (dd, J = 16.2, 5.1 Hz, 1H), 2.98 (s, 1H), 2.49 (dd, J = 22.5, 13.3 Hz, 2H), 2.28 (d, J = 7.1 Hz, 1H), 1.93 (d, J = 13.4 Hz, 1H) ppm. **^{13}C NMR** (150 MHz, $CDCl_3$): δ 202.6, 200.5, 141.8, 138.4, 136.4, 135.4 (d, $J_{C\text{-}F}$ = 30.0 Hz), 130.1, 126.9, 126.8 (q, $J_{C\text{-}F}$ = 7.0, 3.4 Hz), 125.3, 124.4, 119.0, 114.6, 114.3, 94.7, 77.7, 53.9, 52.4, 51.2, 49.3, 34.0, 30.4, 27.5 ppm. **IR** (KBr): ν_{max} = 3444, 2924, 1719, 1379, 1216, 750, 611 cm^{-1}. **HRMS** (m/z): Calcd for $C_{26}H_{22}F_3N_2O_3S$, $[M+H]^+$, 499.1298; found: 499.1297. $[\alpha]_D^{25}$: –122.8 (c 0.15, $CHCl_3$).

2.17 Preparation of 15an

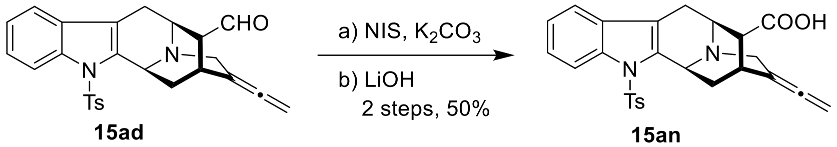

To a solution of **15ad** (10 mg, 0.023 mmol) in dry MeOH (1 mL) were added NIS (13 mg, 0.058 mmol) and K_2CO_3 (8 mg, 0.058 mmol). The reaction mixture was stirred for 1 h before a solution of $Na_2S_2O_3$ (10 mL) was added to quench the reaction. The mixture was extracted with EtOAc (3 × 15 mL). The combined organic phase was washed with brine (3 × 10 mL), dried over Na_2SO_4, filtered, and concentrated. The crude product was directly used for the next reaction.

To a mixture of the above crude product (9.3 mg, 0.02 mmol) in MeOH/H_2O (2 : 1, 1 mL) was added LiOH (1.1 mg, 0.05 mmol). The reaction mixture was stirred at room temperature for 2 h. The mixture was extracted with EtOAc (3 × 15 mL). The combined organic phase was washed with brine (3 × 10 mL), dried over Na_2SO_4, filtered, and concentrated. The crude product was purified by flash column chromatography on silica gel (petroleum ether/EtOAc = 5/1, R_f = 0.30) to afford **15an** (5.4 mg, 50% yield for 2 steps) as a colorless oil.

^{1}H NMR (400 MHz, CD_3OD): δ 8.07 (d, J = 8.3 Hz, 1H), 7.72 (d, J = 8.2 Hz, 2H), 7.40 (d, J = 7.6 Hz, 1H), 7.33–7.20 (m, 4H), 4.79 (s, 2H), 3.95–3.75 (m, 2H), 3.66–3.46 (m, 2H), 3.02 (dd, J = 16.5, 5.0 Hz, 1H), 2.93 (s, 1H), 2.67 (d, J = 16.5 Hz, 1H), 2.47–2.39 (m, 1H), 2.36–2.28 (m, 4H), 1.91 (d, J = 13.4 Hz, 1H) ppm. **^{13}C NMR** (100 MHz, CD_3OD): δ 201.0, 145.4, 137.3, 136.3, 135.4, 129.7, 126.2, 124.5, 123.5, 118.4, 114.1, 112.9, 94.2, 76.0,

72.4, 63.0, 52.0, 51.3, 50.4, 46.5, 33.7, 31.9, 26.3, 20.0 ppm. **IR** (KBr): ν_{max} = 3687, 2925, 1715 1220, 1033, 618 cm^{-1}. **HRMS** (m/z): Calcd for $C_{26}H_{25}N_2O_4S$, $[M+H]^+$, 461.1530; found: 461.1532. $[\alpha]_D^{25}$: +36.0 (*c* 0.9, $CHCl_3$).

2.18 Preparation of 15ao

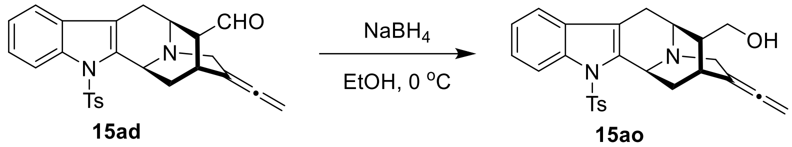

To a solution of **15ad** (46.2 mg, 0.1 mmol) in EtOH (1 mL) was added $NaBH_4$ (18.9 mg, 0.5 mmol) at 0 ℃. The reaction was stirred for 1 h before a saturated aqueous solution of NH_4Cl (10 mL) was added to quench the reaction. The resulting mixture was extracted with EtOAc (3 × 15 mL). The combined organic phase was washed with brine (3 × 10 mL), dried over Na_2SO_4, filtered, and concentrated. The crude product was purified by flash column chromatography (petroleum ether/EtOAc = 2/1, R_f = 0.10) to afford **15ao** (39.5 mg, 85% yield) as a colorless oil. **^{1}H NMR** (400 MHz, $CDCl_3$): δ 8.08 (d, *J* = 8.0 Hz, 1H), 7.64 (d, *J* = 8.3 Hz, 2H), 7.42 (d, *J* = 7.0 Hz, 1H), 7.32–7.26 (m, 1H), 7.23 (d, *J* = 7.3 Hz, 1H), 7.17 (d, *J* = 8.2 Hz, 2H), 4.78 (t, *J* = 4.2 Hz, 2H), 4.67 (d, *J* = 7.6 Hz, 1H), 4.20 (s, 1H), 3.81–3.66 (m, 2H), 3.62–3.51 (m, 1H), 3.02 (d, *J* = 16.1 Hz, 1H), 2.82 (dd, *J* = 16.0, 6.0 Hz, 1H), 2.60 (d, *J* = 1.9 Hz, 1H), 2.31 (s, 3H), 2.24 (d, *J* = 11.8 Hz, 1H), 2.08 (dt, *J* = 14.1, 3.2 Hz, 1H), 1.31 (s, 2H) ppm. **^{13}C NMR** (100 MHz, $CDCl_3$): δ 200.9, 144.9, 138.1, 137.2, 136.4, 135.8, 130.1, 129.2, 126.4, 124.7, 123.7, 118.9, 115.3, 114.6, 99.0, 77.0, 70.3, 54.6, 52.6, 50.6, 35.1, 25.7, 21.7, 21.4 ppm. **IR** (KBr): ν_{max} = 3413, 1637, 1261, 1091, 1019, 748, 581 cm^{-1}. **HRMS** (m/z): Calcd for $C_{26}H_{27}N_2O_3S$, $[M+H]^+$, 447.1737; found: 447.1736. $[\alpha]_D^{25}$: +38.5 (*c* 0.13, $CHCl_3$).

2.19 Preparation of 15ap

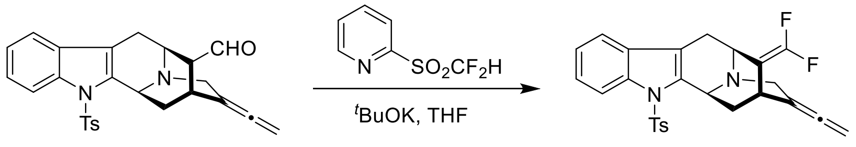

To a solution of **15ad** (44.4 mg, 0.1 mmol) in THF (1 mL) was added ^{t}BuOK (22.4 mg, 0.2 mmol) at room temperature. After being stirred for 30 min, the reaction mixture was added with 2-((difluoromethyl)sulfonyl)pyridine (28.9 mg, 0.15 mmol) in THF (1 mL), and was stirred for an additional 1 h. The resulting solution was then quenched with a saturated aqueous solution of $NaHCO_3$ (10 mL) followed by extraction with EtOAc (3 × 15 mL). The combined organic phase was washed with brine (3 × 10 mL), dried over Na_2SO_4,

filtered, and concentrated. The crude product was purified by flash column chromatography (petroleum ether/EtOAc = 2/1, R_f = 0.30) to afford **15ap** (22.2 mg, 46% yield) as a colorless oil.

^{1}H NMR (400 MHz, $CDCl_3$): δ 8.08 (d, J = 8.1 Hz, 1H), 7.67–7.60 (m, 2H), 7.37 (d, J = 7.5 Hz, 1H), 7.31–7.25 (m, 1H), 7.25–7.20 (m, 1H), 7.17 (d, J = 8.1 Hz, 2H), 4.81 (s, 2H), 4.73 (d, J = 7.6 Hz, 1H), 4.03 (s, 1H), 3.96–3.79 (m, 2H), 3.20 (s, 1H), 3.00 (s, 2H), 2.44 (dd, J = 12.3, 10.5 Hz, 1H), 2.31 (s, 3H), 1.89 (d, J = 13.1 Hz, 1H) ppm. **^{13}C NMR** (100 MHz, $CDCl_3$): δ 200.0, 149.7, 144.9, 145.0, 137.9, 137.5, 136.5, 135.8, 130.1, 129.9, 126.5, 124.7, 123.7, 121.2, 119.8, 118.7, 114.7, 113.2, 97.6, 90.5, 78.0, 52.5, 50.9, 50.6, 34.6, 29.8, 29.3, 24.9, 21.7 ppm. **IR** (KBr): ν_{max} = 2925, 1961, 1746, 1416, 1258, 575 cm^{-1}. **HRMS** (m/z): Calcd for $C_{26}H_{23}F_2N_2O_2S$, $[M+H]^+$, 465.1443; found: 465.1444. **m.p.**: 121.4–123.5 ℃. **$[\alpha]_D^{25}$**: +36.0 (c 0.9, $CHCl_3$).

2.20 Preparation of 15aq

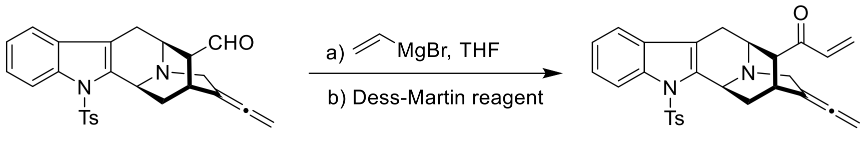

15ad 15aq

To a solution of **15ad** (44.4 mg, 0.1 mmol) in THF (1 mL) was added dropwise vinylmagnesium bromide (1 M in THF, 0.15 mL, 0.15 mmol) at 0 ℃. The reaction mixture was stirred for 1 h before a saturated aqueous solution of NH_4Cl (10 mL) was added to quench the reaction. The resulting mixture was extracted with EtOAc (3 × 15 mL). The combined organic phase was washed with brine (3 × 15 mL), dried over Na_2SO_4, filtered, and concentrated under reduced pressure. The crude product was directly used for the next reaction.

To a mixture of the above crude product (38.7 mg, 0.08 mmol) in CH_2Cl_2 (1 mL) was added Dess-Martin reagent (50.9 mg, 0.12 mmol) at room temperature. The mixture was stirred for 1 h before a saturated aqueous solution of $NaHCO_3$ (15 mL) was added to quench the reaction. The resulting mixture was extracted with EtOAc (3 × 15 mL). The combined organic phase was washed with brine (3 × 15 mL), dried over Na_2SO_4, filtered, and concentrated. The crude product was purified by flash column chromatography on silica gel (petroleum ether/EtOAc = 2/1, R_f = 0.20) to afford **15aq** (24.4 mg, 53% yield for 2 steps) as a colorless oil.

^{1}H NMR (400 MHz, $CDCl_3$): δ 8.09 (d, J = 8.2 Hz, 1H), 7.66 (d, J = 7.7 Hz, 2H), 7.37–7.28 (m, 2H), 7.25–7.14 (m, 3H), 6.39 (dd, J = 17.3, 10.6 Hz, 1H), 6.20 (d, J = 17.4 Hz, 1H), 5.75 (d, J = 10.5 Hz, 1H), 4.78–4.62 (m, 3H), 4.02 (s, 1H), 3.79 (dd, J = 33.5, 16.2 Hz, 2H), 3.02 (dd, J = 16.1, 4.9 Hz, 1H), 2.81 (s, 1H), 2.64 (d, J = 7.1 Hz, 1H), 2.52–2.43 (m, 1H), 2.36 (d, J = 16.4 Hz, 1H), 2.31 (s, 3H), 1.97 (d, J = 13.7 Hz, 1H) ppm. **^{13}C NMR** (100 MHz, $CDCl_3$): δ 200.9, 199.1, 145.1, 136.5, 135.7, 134.9, 130.2, 130.0, 128.9, 126.5, 124.7, 123.7, 118.7, 114.7, 113.3, 77.4, 52.2, 51.9, 50.9, 49.5, 34.5, 33.0, 27.4, 21.7 ppm. **IR** (KBr): ν_{max} = 3403, 2924, 1693, 1615, 1403,

1304, 541 cm^{-1}. **HRMS** (m/z): Calcd for $C_{28}H_{26}N_2O_3S$, $[M+H]^+$, 471.1737; found: 471.1738. **$[\alpha]_D^{25}$**: +75.2 (*c* 0.25, $CHCl_3$).

2.21 Preparation of 15ar

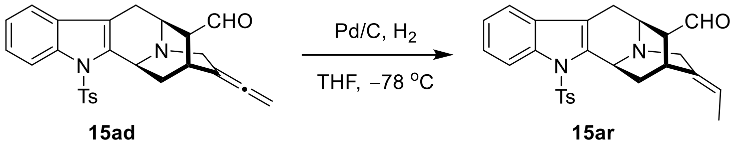

A mixture of **15ad** (46.2 mg, 0.1 mmol) and Pd/C (10%, 10.6 mg, 0.01 mmol) in MeOH (5 mL) was stirred at –78 ℃ under an atmosphere of H_2 (balloon) for 3 h. After filtration through a pad of Celite and removal of the solvent, the residue was purified by flash column chromatography on silica gel (petroleum ether/EtOAc = 1/1, R_f = 0.20) to afford **15ar** (40.7 mg, 91% yield) as a colorless oil.

^{1}H NMR (600 MHz, $CDCl_3$): δ 9.64 (s, 1H), 8.09 (d, *J* = 8.3 Hz, 1H), 7.64 (d, *J* = 8.0 Hz, 2H), 7.35 (d, *J* = 7.6 Hz, 1H), 7.29 (t, *J* = 7.7 Hz, 1H), 7.23 (t, *J* = 7.4 Hz, 1H), 7.17 (d, *J* = 7.9 Hz, 2H), 5.36 (d, *J* = 6.6 Hz, 1H), 4.70 (d, *J* = 9.6 Hz, 1H), 3.69–3.58 (m, 3H), 3.22 (s, 1H), 3.02 (dd, *J* = 16.1, 4.9 Hz, 1H), 2.50 (d, *J* = 16.1 Hz, 1H), 2.37 (d, *J* = 7.3 Hz, 1H), 2.34–2.28 (m, 4H), 1.97 (d, *J* = 13.4 Hz, 1H), 1.62 (d, *J* = 6.4 Hz, 3H) ppm. **^{13}C NMR** (150 MHz, $CDCl_3$): δ 202.7, 144.9, 139.0, 136.6, 135.8, 134.0, 130.1, 130.0, 126.4, 124.7, 123.7, 118.6, 117.3, 114.7, 113.2, 56.3, 55.5, 51.6, 49.3, 34.2, 27.4, 26.9, 21.7, 12.8 ppm. **IR** (KBr): ν_{max} = 2919, 1719, 1452, 1371, 1173, 1119, 749, 578 cm^{-1}. **HRMS** (m/z): Calcd for $C_{26}H_{27}N_2O_3S$, $[M+H]^+$, 447.1737; found: 447.1737. **$[\alpha]_D^{25}$**: +142.7 (*c* 0.2, $CHCl_3$).

2.22 Preparation of (+)-vellosimine (4)

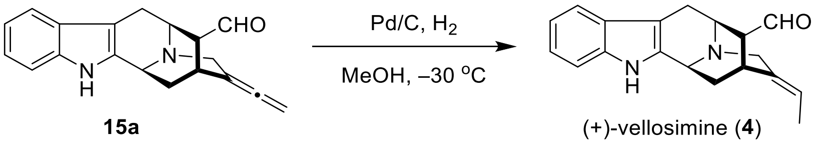

A mixture of **15a** (14.5 mg, 0.05 mmol) and Pd/C (10%, 5.3 mg, 0.005 mmol) in MeOH (2 mL) was stirred at –30 °C under an atmosphere of H_2 (balloon) for 5 h. After the filtration through a pad of Celite and removal of the solvent, the residue was purified by flash column chromatography on silica gel (petroleum ether/EtOAc = 1/1, R_f = 0.10) to afford (+)-vellosimine (**4**) (12.4 mg, 85%, E/Z>20:1) as a white solid. The data of (+)-vellosimine (**4**) is consistent with our previous report.[1]

2.23 Preparation of 14aa

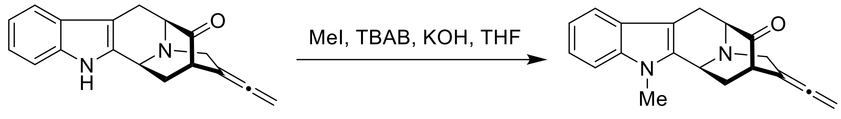

To a solution of **14a** (300 mg, 4.3 mmol) in dry THF (22 mL) were added K_2CO_3 (244 mg, 1.1 mmol), TBAB (21 mg, 0.06 mmol) and MeI (0.27 mL, 4.3 mmol). After being stirred at room temperature for 2 h under an atmosphere of argon, the reaction mixture was filtered through a pad of Celite, washed with CH_2Cl_2 (10 mL), and concentrated under reduced pressure. The residue was dissolved in CH_2Cl_2 (10 mL), and washed successively with H_2O (3 × 15 mL) and a saturated aqueous solution of $NaHCO_3$ (3 × 15 mL). The combined organic layer was dried over Na_2SO_4, filtered, and concentrated in vacuo. The crude product was purified by flash column chromatography on silica gel (petroleum ether/EtOAc = 3 :1, R_f = 0.20) to afford **14aa** (221 mg, 70%) as a white solid.

^{1}H NMR (400 MHz, $CDCl_3$): δ 7.50 (d, J = 7.8 Hz, 1H), 7.27 (d, J = 7.3 Hz, 1H), 7.20 (t, J = 7.6 Hz, 1H), 7.10 (t, J = 7.4 Hz, 1H), 4.88 (q, J = 4.2 Hz, 2H), 4.38 (dd, J = 9.5, 2.5 Hz, 1H), 4.00 (dt, J = 7.6, 4.4 Hz, 2H), 3.71 (d, J = 6.3 Hz, 1H), 3.61 (s, 3H), 3.33 (d, J = 15.5 Hz, 1H), 3.08–2.97 (m, 2H), 2.70 (ddd, J = 12.0, 9.6, 2.1 Hz, 1H), 2.16 (dt, J = 13.0, 3.3 Hz, 1H) ppm. **^{13}C NMR** (100 MHz, $CDCl_3$): δ 216.3, 202.6, 137.6, 137.5, 126.6, 121.6, 119.3, 118.7, 108.9, 104.5, 95.4, 78.5, 64.5, 52.1, 49.4, 45.8, 35.9, 29.5, 22.8 ppm. **IR** (KBr): ν_{max} = 3451, 1729, 1642, 1455, 1438, 1302, 1263, 1076, 853, 731 cm^{-1}. **HRMS** (m/z): Calcd for $C_{18}H_{17}N_2O$, $[M+H]^+$, 277.1335; found: 277.1341. **m.p.**: 270.0–271.5 ºC. $[\alpha]_D^{25}$: –137.6 (c 0.1, MeOH).

2.24 Preparation of 15aa

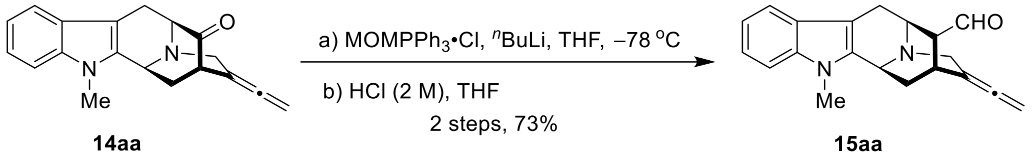

To a solution of $MOMPPh_3$ Cl (34.2 mg, 1.0 mmol) in THF (2 mL) was added dropwise ^{n}BuLi (2.0 M in hexane 0.4 mL, 0.8 mmol) at –78 ℃. After stirring for 30 min, the reaction mixture was added with a solution of **14aa** (29.0 mg, 0.1 mmol) in THF (1 mL). The resulting mixture was stirred for an additional 1 h before H_2O (1 mL) was added slowly to quench the reaction. The resulting mixture was extracted with EtOAc (3 × 15 mL). The organic phase was washed with brine (3 × 10 mL), dried over Na_2SO_4, filtered, and concentrated under reduced pressure. The crude product was directly used in the next reaction.

To a mixture of the above crude product in THF (1 mL) was added HCl (2 M, 1 mL) at 70 ℃ for 5 h before a saturated aqueous solution of NaCl (2 mL) was added slowly to quench the reaction. The resulting mixture was

extracted with EtOAc (3 × 15 mL). The combined organic phase was washed with brine (3 × 15 mL), dried over Na_2SO_4, filtered, and concentrated under reduced pressure. The crude product was purified by flash column chromatography on silica gel (petroleum ether/EtOAc = 5/1, R_f = 0.30) to afford **15aa** (22.2 mg, 73%) as a white solid.

^{1}H NMR (400 MHz, $CDCl_3$): δ 9.71 (s, 1H), 7.47 (d, J = 7.8 Hz, 1H), 7.30 (d, J = 8.2 Hz, 1H), 7.20 (t, J = 7.6 Hz, 1H), 7.10 (t, J = 7.5 Hz, 1H), 4.75 (q, J = 5.2 Hz, 2H), 4.27 (dd, J = 9.3, 2.4 Hz, 1H), 3.86–3.73 (m, 2H), 3.70 (t, J = 6.0 Hz, 1H), 3.65 (s, 3H), 3.17 (dd, J = 15.6, 5.2 Hz, 1H), 2.95 (s, 1H), 2.67–2.60 (m, 1H), 2.40 (d, J = 7.3 Hz, 1H), 2.29 (t, J = 11.2 Hz, 1H), 1.74 (d, J = 12.5 Hz, 1H) ppm. **^{13}C NMR** (100 MHz, $CDCl_3$): δ 202.9, 200.5, 139.1, 121.3, 119.2, 118.4, 109.0, 103.2, 95.3, 77.4, 53.4, 52.5, 50.8, 49.1, 32.4, 30.2, 29.5, 27.5 ppm. **IR** (KBr): ν_{max} = 2715, 1960, 1716, 1638, 1470, 1124, 848, 743 cm^{-1}. **HRMS** (m/z): Calcd for $C_{20}H_{19}N_2O$, $[M–H]^-$, 303.1503; found: 303.1494. **m.p.**: 179.0–188.9 °C. $[\alpha]_D^{25}$: +72.7 (*c* 0.047, MeOH).

2.25 Preparation of (+)-N_a-methylvellosimine (5)

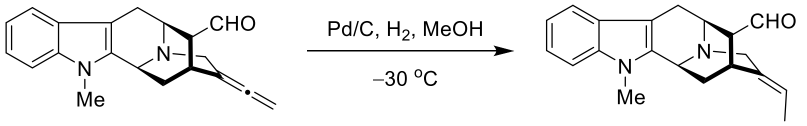

15aa (+)-N_a-methylvellosimine (**5**)

A mixture of **15aa** (150 mg, 0.49 mmol) and Pd/C (10%, 52.4 mg, 0.05 mmol) in MeOH (10 mL) was stirred at –30 °C under an atmosphere of H_2 (balloon) for 3 h. After the filtration through a pad of Celite and removal of the solvent, the crude product was purified by flash column chromatography on silica gel (petroleum ether/EtOAc = 1/1, R_f = 0.20) to afford (+)-N_a-methylvellosimine (**5**) (135 mg, 89%, E/Z>20:1) as a white solid.

^{1}H NMR (400 MHz, $CDCl_3$) δ 9.64 (s, 1H), 7.47 (d, J = 7.8 Hz, 1H), 7.30 (d, J = 8.2 Hz, 1H), 7.20 (t, J = 7.6 Hz, 1H), 7.10 (t, J = 7.4 Hz, 1H), 5.36 (q, J = 7.1 Hz, 1H), 4.27 (d, J = 9.6 Hz, 1H), 3.65 (s, 3H), 3.63–3.59 (m, 3H), 3.22–3.19 (m, 1H), 3.15 (dd, J = 15.5, 5.3 Hz, 1H), 2.62 (d, J = 15.5 Hz, 1H), 2.49 (d, J = 7.6 Hz, 1H), 2.13 (ddd, J = 12.1, 10.0, 2.0 Hz, 1H), 1.81–1.72 (m, 1H), 1.62 (d, J = 6.4 Hz, 3H) ppm. **^{13}C NMR** (100 MHz, $CDCl_3$): δ 202.8, 139.2, 137.4, 134.4, 127.2, 121.1, 119.0, 118.2, 117.0, 108.8, 103.2, 56.2, 54.9, 50.5, 49.4, 32.4, 29.4, 27.3, 26.6, 12.7 ppm. **IR** (KBr): v_{max} = 2871, 1715, 1637, 1470, 741. **HRMS** (m/z): Calcd for $C_{20}H_{21}N_2O$, $[M+H]^+$, 305.1659; found: 305.1655 cm^{-1}. **m.p.**: 256.3–262.3 °C. $[\alpha]_D^{25}$: +22.8 (*c* 0.01, MeOH).

2.26 Supplementary Table 3 ^{1}H NMR spectroscopic data comparison of (+)-N_a-methylvellosimine (5)

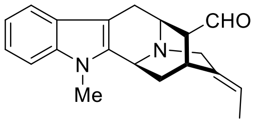

(+)-N_a-methylvellosimine (**5**)

Entry	δ_H of our synthetic (+)-N_a-methylvellosimine (**5**) (400 MHz, $CDCl_3$)	δ_H of Martin's synthetic (+)-N_a-methylvellosimin (**5**)[4] (500 MHz, $CDCl_3$)	$\Delta\delta_H$ (δ_{ours} vs $\delta_{Martin's}$)
1	9.64 (s, 1H)	9.64 (d, *J* = 1.0 Hz, 1H)	0
2	7.47 (d, *J* = 7.8 Hz, 1H)	7.47 (ddd, *J* = 7.0, 1.0, 1.0 Hz, 1H)	0
3	7.30 (d, *J* = 8.2 Hz, 1H)	7.30 (d, *J* = 8.0 Hz, 1H)	0
4	7.20 (t, *J* = 7.6 Hz, 1H)	7.20 (ddd, *J* = 8.0, 7.0, 1.0 Hz, 1 H)	0
5	7.10 (t, *J* = 7.4 Hz, 1H)	7.09 (ddd, *J* = 7.5, 7.0, 1.0 Hz, 1H)	+0.01
6	5.36 (q, *J* = 7.1 Hz, 1H)	5.36 (q, *J* = 7.0 Hz, 1H)	0
7	4.27 (d, *J* = 9.6 Hz, 1H)	4.27 (dd, *J* =10.0, 2.0 Hz, 1H)	0
8	3.63–3.59 (m, 3H)	3.67–3.60 (m, 3H)	—
9	3.65 (s, 3H)	3.65 (s, 3H)	0
10	3.22–3.19 (m, 1H)	3.19–3.21 (m, 1H)	—
11	3.15 (dd, *J* = 15.5, 5.3 Hz, 1H)	3.15 (dd, *J* = 15.5, 5.0 Hz, 1H)	0
12	2.62 (d, *J* = 15.5 Hz, 1H)	2.62 (dd, *J* = 15.5, 1.5 Hz, 1H)	0
13	2.49 (d, *J* = 7.6 Hz, 1H)	2.49 (d, *J* = 7.5 Hz, 1H)	0
14	2.13 (ddd, *J* = 12.1, 10.0, 2.0 Hz, 1H)	2.13 (ddd, *J* = 12.5, 9.5, 2.0 Hz, 1H)	0
15	1.81–1.72 (m, 1H)	1.77 (ddd, *J* = 12.5, 4.0, 2.0 Hz)	—
16	1.62 (d, *J* = 6.4 Hz, 3H)	1.62 (dt, *J* = 7.0, 2.0 Hz, 3H)	0

2.27 Supplementary Table 4 ^{13}C NMR spectroscopic data comparison of (+)-N_a-methylvellosimine (5)

Entry	δ_C of our synthetic (+)-N_a-methylvellosimine (**5**) (100 MHz, $CDCl_3$)	δ_C of Martin's synthetic (+)-N_a-methylvellosimin (**5**)[4] (125 MHz, $CDCl_3$)	$\Delta\delta_C$ (δ_{ours} vs $\delta_{Martin's}$)
1	202.8	202.8	0
2	139.2	139.2	0
3	137.4	137.4	0
4	134.4	134.4	0
5	127.2	127.2	0
6	121.1	121.1	0
7	119.0	119.0	0
8	118.2	118.2	0
9	117.0	117.0	0
10	108.8	108.8	0
11	103.2	103.1	+0.1
12	56.2	56.2	0
13	54.9	54.9	0
14	50.6	50.6	0
15	49.4	49.4	+0
16	32.4	32.4	0
17	29.4	29.4	0
18	27.3	27.3	0
19	26.6	26.6	0
20	12.7	12.6	+0.1

2.28 Preparation of 15l

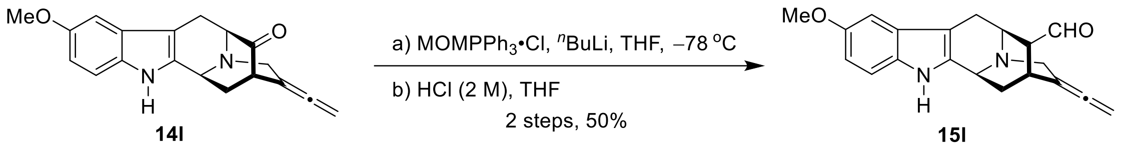

To a solution of $MOMPPh_3$ Cl (34.2 mg, 1.0 mmol) in THF (2 mL) was added dropwise ^{n}BuLi (2.0 M in hexane 0.4 mL, 0.8 mmol) at –78 °C. After stirring for 30 min, the reaction mixture was added with a solution of **14l** (30.6 mg, 0.1 mmol) in THF (1 mL). The resulting mixture was stirred for an additional 1 h, and the resulting solution was extracted with EtOAc (3 × 15 mL). The combined organic phase was washed with brine (3 × 10 mL), dried over Na_2SO_4, filtered, and concentrated under reduced pressure. The crude product was directly used in the next reaction.

To a mixture of the above crude product in THF (1 mL) was added HCl (2 M, 1 mL) under stirring at 70 °C for 5 h before a saturated aqueous solution of NaCl (2 mL) was added slowly to quench the reaction. The resulting mixture was extracted with EtOAc (3 × 15 mL). The combined organic phase was washed with brine (3 × 10 mL), dried over Na_2SO_4, filtered, and concentrated under reduced pressure. The crude product was purified by flash column chromatography on silica gel (petroleum ether/EtOAc = 2/1, R_f = 0.40) to afford **15l** (25.9 mg, 80%) as a colorless oil.

^{1}H NMR (400 MHz, $CDCl_3$) δ 9.71 (s, 1H), 7.69 (s, 1H), 7.20 (d, J = 8.7 Hz, 1H), 6.91 (d, J = 2.5 Hz, 1H), 6.81 (dd, J = 8.8, 2.5 Hz, 1H), 4.74 (q, J = 4.6 Hz, 2H), 4.17 (d, J = 9.8 Hz, 1H), 3.85 (s, 3H), 3.80–3.68 (m, 3H), 3.13 (dd, J = 15.5, 5.3 Hz, 1H), 2.95 (s, 1H), 2.58 (d, J = 15.5 Hz, 1H), 2.43 (d, J = 7.3 Hz, 1H), 2.23 (t, J = 11.6 Hz, 1H), 1.80 (d, J = 12.7 Hz, 1H) ppm. **^{13}C NMR** (100 MHz, $CDCl_3$) δ 202.9, 200.5, 154.4, 138.5, 131.5, 128.1, 111.7, 104.4, 100.7, 95.4, 56.1, 53.6, 52.3, 50.9, 50.3, 33.2, 30.6, 27.5 ppm. **IR** (KBr): ν_{max} = 2921, 1726, 1323, 1199, 1057, 797 cm^{-1}. **HRMS** (m/z): Calcd for $C_{20}H_{21}N_2O_2$, $[M+H]^+$, 321.1597; found: 321.1599. $[\alpha]_D^{25}$: +47.2 (c 0.21, $CHCl_3$).

2.29 Preparation of (+)-10-methoxyvellosimine (6)

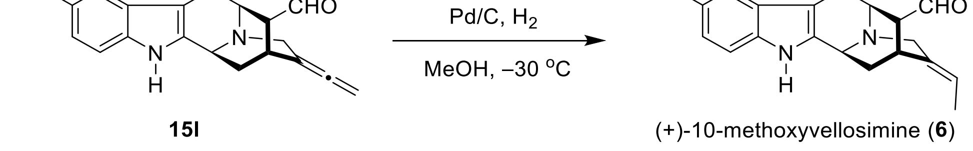

A mixture of **15l** (14.1 mg, 0.044 mmol) and Pd/C (10%, 4.7 mg, 0.004 mmol) in MeOH (1 mL) was stirred at –30 °C under an atmosphere of H_2 (balloon) for 45 min. After the filtration through a pad of Celite and removal of the solvent, the crude product was purified by flash column chromatography on silica gel (petroleum ether/EtOAc

= 1/1, R_f = 0.20) to afford (+)-10-methoxyvellosimine (**6**) (8.1 mg, 57%, E/Z>20:1) as a white solid.

^{1}H NMR (600 MHz, DMSO-d_6): δ 10.66 (s, 1H), 9.56 (s, 1H), 7.16 (d, J = 8.7 Hz, 1H), 6.86 (d, J = 2.1 Hz, 1H), 6.65 (dd, J = 8.7, 2.3 Hz, 1H), 5.24 (d, J = 6.8 Hz, 1H), 4.09 (d, J = 9.6 Hz, 1H), 3.73 (s, 3H), 3.53–3.40 (m, 3H), 3.20 (s, 1H), 2.87 (dd, J = 15.0, 5.1 Hz, 1H), 2.43 (d, J = 8.3 Hz, 1H), 2.41 (s, 1H), 1.97 (t, J = 11.3 Hz, 1H), 1.69 (d, J = 12.5 Hz, 1H), 1.56 (d, J = 6.7 Hz, 3H) ppm. **^{13}C NMR** (150 MHz, DMSO-d_6): δ 204.0, 153.4, 140.3, 136.3, 131.6, 127.8, 115.6, 112.0, 110.5, 102.5, 100.3, 55.7, 55.6, 54.8, 50.3, 50.0, 33.2, 27.3, 26.7, 12.7 ppm. **IR** (KBr): ν_{max} = 2487, 2993, 1751, 1621, 1465, 1167, 745 cm^{-1}. **HRMS** (m/z): Calcd for $C_{20}H_{23}N_2O_2$, $[M+H]^+$, 323.1754; found 323.1747. **m. p.**: 240.0–241.0 °C. $[\alpha]_D^{25}$: +72.0 (c 0.1, MeOH).

2.30 Supplementary Table 5 ^{1}H NMR spectroscopic data comparison of (+)-10-methoxyvellosimine (6)

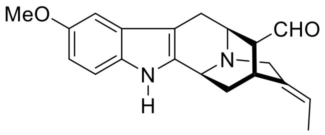

(+)-10-methoxyvellosimine (**6**)

Entry	δ_H of our synthetic (+)-10-methoxyvellosimine (**6**) (600 MHz, DMSO-d_6)	δ_H of Cook's synthetic (+)-10-methoxyvellosimine (**6**) (300 MHz, DMSO-d_6)[5]	$\Delta\delta_H$ (δ_{ours} vs $\delta_{Cook's}$)
1	10.66 (s, 1H)	10.66 (s, 1H)	0
2	9.56 (s, 1H)	9.57 (s, 1H)	–0.01
3	7.16 (d, J = 8.7 Hz, 1H)	7.17 (d, J = 8.7 Hz, 1H)	–0.01
4	6.86 (d, J = 2.1 Hz, 1H)	6.87 (d, J = 2.1 Hz, 1H)	–0.01
5	6.65 (dd, J = 8.7, 2.3 Hz, 1H)	6.66 (dd, J = 8.7, 2.3 Hz, 1H)	–0.01
6	5.24 (d, J = 6.8 Hz, 1H)	5.24 (d, J = 6.8 Hz, 1H)	0
7	4.09 (d, J = 9.6 Hz, 1H)	4.10 (d, J = 9.6 Hz, 1H)	–0.01
8	3.73 (s, 3H)	3.73 (s, 3H)	0
9	3.53–3.40 (m, 2H)	3.53–3.40 (m,3H)	—
10	3.20 (s, 1H)	3.20 (t, J = 2.0 Hz, 1H)	0
11	2.87 (dd, J = 15.0, 5.1 Hz, 1H)	2.85 (dd, J = 15.1, 5.0 Hz, 1H)	+0.02
12	2.43 (d, J = 8.3 Hz, 1H)	2.45 (d, J = 5.5 Hz, 1H)	–0.02
13	2.41 (s, 1H)	2.41 (bs, 1H)	0
14	1.97 (t, J = 11.3 Hz, 1H)	1.97 (ddd, J = 22.2, 11.0,1.3 Hz, 1H)	0
15	1.69 (d, J = 12.5 Hz, 1H)	1.69 (dt, J = 12.4, 2.9 Hz, 1H)	0
16	1.56 (d, J = 6.7 Hz, 3H)	1.56 (d, J = 6.7 Hz, 3H)	0

2.31 Supplementary Table 6 ^{13}C NMR spectroscopic data comparison of (+)-10-methoxyvellosimine (6)

Entry	δ_C of our synthetic (+)-10-methoxyvellosimine (**6**) (150 MHz, DMSO-d_6)	δ_C of Cook's synthetic (+)-10-methoxyvellosimine (**6**) (75 MHz, DMSO-d_6)[5]	$\Delta\delta_C$ (δ_{ours} vs $\delta_{Cook's}$)
1	204.0	204.0	0

2	153.4	153.4	0
3	140.3	140.3	0
4	136.3	136.4	–0.1
5	131.6	131.5	+0.1
6	127.8	127.8	0
7	115.6	115.6	0
8	112.0	112.0	0
9	110.5	110.5	0
10	102.5	102.5	0
11	100.3	100.2	+0.1
12	55.7	55.7	0
13	55.6	55.6	0
14	54.8	54.8	0
15	50.3	50.2	+0.1
16	50.0	50.0	0
17	33.2	33.2	0
18	27.3	27.3	0
19	26.7	26.7	0
20	12.7	12.7	0

2.32 Preparation of 16

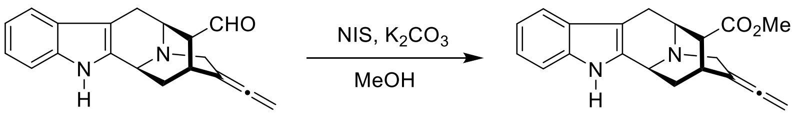

15a 16

To a solution of **15a** (203.1 mg, 0.7 mmol) in MeOH (22 mL) were added NIS (386 mg, 1.7 mmol) and K_2CO_3 (378 mg, 1.7 mmol). The mixture was stirred at room temperature for 2 h under an atmosphere of argon in the dark. The reaction mixture was quenched with a saturated aqueous solution of NH_4Cl (10 mL). The organic phase was dissolved in CH_2Cl_2 (50 mL), washed with brine (30 mL), dried over Na_2SO_4, filtered, and concentrated under reduced pressure. The crude product was purified by flash column chromatography on silica gel (petroleum ether/EtOAc = 2/1, R_f = 0.40) to afford **16** (190.6 mg, 85%) as a white solid.

^{1}H NMR (400 MHz, $CDCl_3$): δ 8.00 (s, 1H), 7.46 (d, J = 7.4 Hz, 1H), 7.24 (d, J = 6.9 Hz, 1H), 7.11 (dt, J = 14.4, 7.0 Hz, 2H), 4.79–4.65 (m, 2H), 4.06 (d, J = 9.5 Hz, 1H), 3.86–3.60 (m, 6H), 3.12 (dd, J = 15.6, 4.9 Hz, 1H), 2.86 (s, 1H), 2.68 (d, J = 15.6 Hz, 1H), 2.47 (d, J = 7.3 Hz, 1H), 2.11 (t, J = 11.2 Hz, 1H), 1.73(d, J=13.1Hz, 1H) ppm. **^{13}C NMR** (100 MHz, $CDCl_3$): δ 200.9, 174.1, 137.7, 136.5, 127.7, 121.8, 119.6, 118.3, 111.0, 104.4, 95.7, 76.7, 53.2, 52.2, 52.0, 49.6, 46.2, 33.1, 32.2, 27.5 ppm. **IR** (KBr): ν_{max} = 3520, 1960, 1731, 1631, 1489, 1269, 1209, 1180 cm^{-1}. **HRMS** (m/z): Calcd for $C_{20}H_{21}N_2O_2$, $[M+H]^+$, 321.1598; found 321.1597. **m. p.**: 218.7–223.0 °C. $[\alpha]_D^{25}$: –139.9 (c 0.09, MeOH).

2.33 Preparation of 17

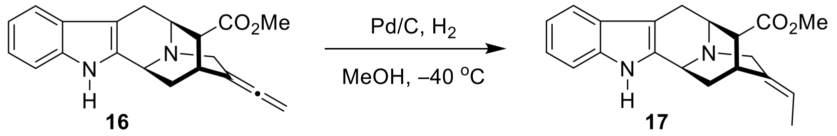

A mixture of **16** (150.5 mg, 0.47 mmol) and Pd/C (10%, 53 mg, 0.05 mmol) in MeOH (10 mL) was stirred at –40 °C under an atmosphere of H_2 (balloon) for 2 h. After the filtration through a pad of Celite and removal of the solvent, the crude product was purified by flash column chromatography on silica gel (petroleum ether/EtOAc = 1/1, R_f = 0.25) to afford **17** (136.3 mg, 90%, E/Z>20:1) as a white solid.

^{1}H NMR (400 MHz, $CDCl_3$): δ 7.95 (s, 1H), 7.46 (d, J = 7.5 Hz, 1H), 7.25 (s, 1H), 7.17–7.05 (m, 2H), 5.32 (d, J = 6.7 Hz, 1H), 4.08 (d, J = 9.3 Hz, 1H), 3.73–3.48 (m, 6H), 3.16 (s, 1H), 3.11 (dd, J = 15.6, 5.0 Hz, 1H), 2.66 (d, J = 15.6 Hz, 1H), 2.54 (d, J = 7.6 Hz, 1H), 1.98 (t, J = 11.2 Hz, 1H), 1.74 (d, J = 12.5 Hz, 1H), 1.59 (d, J = 6.6 Hz, 3H) ppm. **^{13}C NMR** (100 MHz, $CDCl_3$): δ 174.3, 138.0, 136.5, 134.7, 127.8, 121.7, 119.6, 118.3, 116.9, 111.0, 104.6, 56.0, 52.9, 51.8, 50.1, 47.0, 33.3, 28.9, 27.4, 12.9 ppm. **IR** (KBr): ν_{max} = 3480, 1731, 1636, 1622, 1451, 1302, 1208, 1016 cm^{-1}. **HRMS** (m/z): Calcd for $C_{20}H_{23}N_2O_2$, $[M+H]^+$, 323.1754; found 323.1757. **m. p.**: 196.0–202.0 °C. **$[\alpha]_D^{25}$**: +4.5 (c 1.0, MeOH).

2.34 Preparation of (–)-alkaloid Q3 (7)

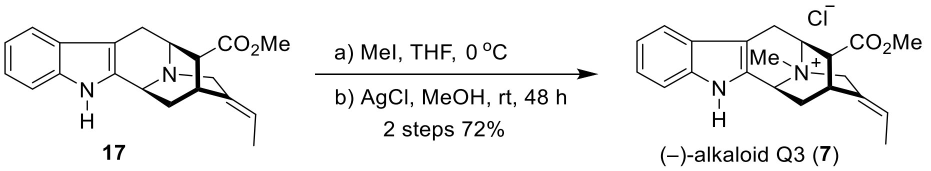

To a solution of **17** (100 mg, 0.31mmol) in THF (10 mL) was added MeI (0.1 mL, 1.6 mmol) at 0 °C under an atmosphere of argon. The reaction was stirred at room temperature for 3 h. The mixture was extracted with EtOAc (3 × 15 mL). The organic phase was washed with brine (3 × 10 mL), dried over Na_2SO_4, filtered, and concentrated under reduced pressure. The crude product was directly used in the next reaction.

To a mixture of the above crude product (122.3 mg, 0.26 mmol) in MeOH (10 mL) was added AgCl (189.0 mg, 1.3 mmol). The reaction mixture was stirred at room temperature for 48 h under an atmosphere of argon in the dark. The excess silver chloride and the resulting silver iodide were removed by filtration, and the solid was washed with methanol (20 mL). The crude product was purified by flash column chromatography on silica gel (dichloromethane/methanol = 10/1, R_f = 0.20) to afford (–)-alkaloid Q3 (**7**) (83.1 mg, 72% for 2 steps) as a white solid.

^{1}H NMR (600 MHz, CD_3OD): δ 7.52 (d, J = 7.9 Hz, 1H), 7.40 (d, J = 8.2 Hz, 1H), 7.20 (t, J = 7.6 Hz, 1H), 7.09

(t, J = 7.5 Hz, 1H), 5.58 (q, J = 6.9 Hz, 1H), 4.99 (d, J = 10.2 Hz, 1H), 4.48 (d, J = 15.5 Hz, 1H), 4.43–4.39 (m, 1H), 4.28 (d, J = 15.6 Hz, 1H), 3.74 (s, 3H), 3.61–3.56 (m, 1H), 3.42 (dd, J = 17.2, 4.9 Hz, 1H), 3.31–3.30 (m, 1H), 3.18 (s, 3H), 3.08 (d, J = 17.0 Hz, 1H), 2.98 (dd, J = 27.6, 7.6 Hz, 1H), 2.60–2.53 (m, 1H), 2.25 (dd, J = 13.2, 3.5 Hz, 1H), 1.66 (dd, J = 22.3, 6.9 Hz, 3H) ppm. **^{13}C NMR** (150 MHz, CD_3OD): δ 170.5, 137.4, 130.5, 126.6, 125.9, 122.5, 120.8, 119.5, 117.9, 111.2, 100.0, 64.3, 62.5, 60.4, 51.6, 46.7, 46.4, 31.0, 27.2, 23.4, 11.4 ppm. **IR** (KBr): ν_{max} = 3547, 3471, 1736, 1619, 1638, 1451, 1332, 1206, 1082 cm^{-1}. **HRMS** (m/z): Calcd for $C_{21}H_{25}N_2O_2$, $[M–Cl]^+$, 337.1910; found 337.1912. **m. p.**: 107.5–112.4 °C. $[\alpha]_D^{25}$: –22.1 (c 0.15, MeOH).

2.35 Supplementary Table 7 ^{1}H NMR spectroscopic data comparison of (–)-alkaloid Q3 (7)

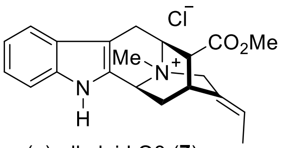

(–)-alkaloid Q3 (**7**)

Entry	δ_H of our synthetic (–)-alkaloid Q3 (**7**) (600 MHz, CD_3OD)	δ_H of Cook's synthetic (–)-alkaloid Q3 (**7**)[6] (300 MHz, CD_3OD)	$\Delta\delta_H$ (δ_{ours} vs $\delta_{Cook's}$)
1	7.52 (d, J = 7.9 Hz, 1H)	7.52 (dt, J = 7.8, 0.9 Hz, 1H)	0
2	7.40 (d, J = 8.2 Hz, 1H)	7.40 (dt, J = 8.1, 0.8 Hz, 1H)	0
3	7.20 (t, J = 7.6 Hz, 1H)	7.20 (ddd, J = 8.2, 7.1, 1.2 Hz, 1H)	0
4	7.09 (t, J = 7.5 Hz, 1H)	7.10 (ddd, J = 8.0, 7.1, 1.1 Hz, 1H)	–0.01
5	5.58 (q, J = 6.9 Hz, 1H)	5.58 (q, J = 6.8 Hz, 1H)	0
6	4.99 (d, J = 10.2 Hz, 1H)	4.98 (d, J = 10.2 Hz, 1H)	+0.01
7	4.48 (d, J = 15.5 Hz, 1H)	4.56 (dt, J = 15.5, 2.4 Hz,1H)	–0.06
8	4.43–4.39 (m, 1H)	4.45 (m, 1H)	—
9	4.28 (d, J = 15.6 Hz, H)	4.30 (d, J = 15.5 Hz, H)	+0.02
10	3.74 (s, 3H)	3.76 (s, 3H)	–0.02
11	3.61–3.56 (m, 1H)	3.60 (m, 1H)	—
12	3.42 (dd, J = 17.2, 5.0 Hz, 1H)	3.41 (dd, J = 17.4, 4.9 Hz,1H)	+0.01
13	3.31–3.30 (m, 1H)	3.33 (m, 1H)	—
14	3.18 (s, 3H)	3.17 (s, 3H)	+0.01
15	3.08 (d, J = 17.0 Hz, 1H)	3.08 (br d, J = 17.5 Hz, 1H)	0
16	2.98 (dd, J = 27.6, 7.6 Hz, 1H)	3.03 (dd, J = 7.7, 1.8 Hz,1H)	–0.02
17	2.59–2.53 (m, 1H)	2.58 (ddd, J = 12.1, 10.3, 1.7 Hz 1H)	–0.02
18	2.25 (dd, J = 13.2, 3.5 Hz1H)	2.26 (dd, J = 13.3, 3.2 Hz 1H)	–0.01
19	1.66 (dd, J = 22.3, 6.9 Hz, 3H)	1.68 (ddd, J = 8.3, 2.3, 2.2 Hz, 3H)	–0.02

2.36 Supplementary Table 8 ^{13}C NMR spectroscopic data comparison of (–)-alkaloid Q3 (7)

Entry	δ_C of our synthetic (–)-alkaloid Q3 (**7**) (150 MHz, CD_3OD)	δ_C of Cook's synthetic (–)-alkaloid Q3 (**7**)[6] (75 MHz, CD_3OD)	$\Delta\delta_C$ (δ_{ours} vs $\delta_{Cook's}$)

1	170.5	170.5	0
2	137.4	137.4	0
3	130.5	130.5	0
4	126.6	126.6	0
5	125.9	125.9	0
6	122.5	122.5	0
7	120.8	120.8	0
8	119.5	119.5	0
9	117.9	117.9	0
10	111.2	111.2	0
11	100.0	100.0	0
12	64.3	64.2	+0.1
13	62.5	62.5	0
14	60.4	60.4	0
15	51.6	51.6	0
16	46.7	47.2	–0.5
17	46.4	46.8	–0.4
18	31.0	31.0	0
19	27.2	27.2	0
20	23.4	23.4	0
21	11.4	11.4	0

2.37 Preparation of 15as

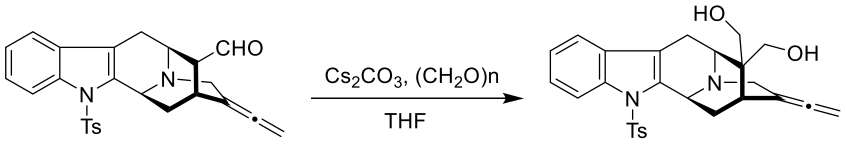

15ad **15as**

To a solution of **15ad** (3.6 g, 8.1 mmol) in THF (25 mL) was added Cs_2CO_3 (21.1g, 64.8 mmol), and $(CH_2O)_n$ (5.8 g, 64.8 mmol). The reaction was stirred at 60 ℃ for 1 h under an atmosphere of argon. After the filtration through a pad of Celite and removal of the solvent, the residue was purified by flash column chromatography on silica gel (EtOAc/petroleum ether = 2/1, R_f = 0.30) to afford **15as** (3.1 g, 81%) as a white solid.

^{1}H NMR (400 MHz, CD_3Cl_3): δ 8.08 (d, J = 8.0 Hz, 1H), 7.64 (d, J = 7.5 Hz, 2H), 7.36 (d, J = 7.3 Hz, 1H), 7.32–7.22 (m, 2H), 7.17 (d, J = 7.7 Hz, 2H), 4.78 (s, 2H), 4.63 (d, J = 8.7 Hz, 1H), 3.87–3.63 (m, 5H), 3.35 (d, J = 10.6 Hz, 1H), 2.97 (s, 1H), 2.84 (s, 2H), 2.56 (s, 1H), 2.35–2.24 (m, 4H), 1.90 (d, J = 13.8 Hz, 1H) ppm. **^{13}C NMR** (100 MHz, CD_3Cl_3): δ 200.5, 145.0, 137.7, 136.4, 135.7, 130.1, 129.1, 126.4, 124.7, 123.8, 118.7, 114.9, 114.6, 98.1, 77.0, 70.8, 64.6, 55.5, 52.6, 50.7, 42.3, 32.5, 29.8, 22.8, 21.7 ppm. **IR** (KBr): ν_{max} = 1961, 1637, 1451, 1370, 1171, 1037, 1021 cm^{-1}. **HRMS** (m/z): Calcd for $C_{27}H_{29}N_2O_4S$, $[M+H]^+$, 477.1843; found: 477.1844. **m. p.**: 202.3–207.9 °C. **$[\alpha]_D^{25}$**: +22.5 (c 0.027, MeOH).

2.38 Preparation of 18

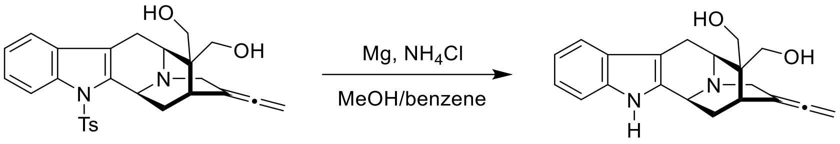

15as **18**

To a solution of **15as** (3 g, 6.3 mmol) in MeOH/benzene (1 : 1, 25 mL) was added Mg (0.7 g, 29 mmol), and NH_4Cl (6.7 g, 125.9 mmol). The reaction mixture was stirred at room temperature for 3 h before a solution of HCl (1 M, 10 mL) was added. The resulting solution was extracted with EtOAc (3 × 50 mL). The organic phase was washed with brine (3 × 20 mL), and was concentrated in vacuo after filtration. The crude product was purified by flash column chromatography on silica gel (petroleum ether/EtOAc = 1/2, R_f = 0.30) to afford **18** (1.9 g, 92%) as a white solid.

^{1}H NMR (400 MHz, CD_3OD): δ 7.37 (d, J = 7.7 Hz, 1H), 7.27 (d, J = 8.0 Hz, 1H), 7.04 (t, J = 7.5 Hz, 1H), 6.96 (t, J = 7.4 Hz, 1H), 4.75 (t, J = 4.4 Hz, 2H), 4.15 (dd, J = 10.1, 2.7 Hz, 1H), 3.78–3.67 (m, 4H), 3.61 (d, J = 10.5 Hz, 1H). 3.31–3.22 (m, 4H), 2.93 (dd, J = 16.3, 6.2 Hz, 1H), 2.79 (d, J = 16.3 Hz, 1H), 2.46 (s, 1H), 2.12–2.04 (m, 1H), 2.01–1.94 (m, 1H) ppm. **^{13}C NMR** (100 MHz, CD_3OD): δ 201.9, 137.8.0, 137.8, 127.4, 122.2, 119.9, 118.7, 112.0, 106.2, 99.1, 76.8, 68.6, 60.1, 58.7, 52.9, 50.8, 43.8, 33.2, 30.2, 23.5 ppm. **IR** (KBr): ν_{max} = 3457, 1961, 1638, 1451 1066, 1018 cm^{-1}. **HRMS** (m/z): Calcd for $C_{20}H_{23}N_2O_2$, $[M+H]^+$, 323.1754; found: 323.1750. **m. p.**: 111.3–126.1 °C. **$[\alpha]_D^{25}$**: +22.5 (c 0.033, MeOH).

2.39 Preparation of 19

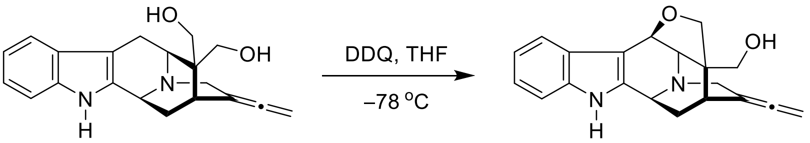

18 **19**

To a solution of **18** (1.8 g, 5.6 mmol) in THF (25 mL) was added DDQ (2.7 g, 11.8 mmol) at –78 °C. The reaction mixture was stirred at room temperature for 1 h under an atmosphere of argon before a saturated aqueous solution of $NaHCO_3$ solution (20 mL) was added to quench the reaction. The resulting mixture was extracted with CH_2Cl_2 (3 × 20 mL). The organic phase was washed with brine (3 × 20 mL), and was concentrated under reduced pressure. The residue was dissolved in a mixed solvent of CH_2Cl_2/MeOH = (10 : 1, 20 mL) and 20% $NH_3·H_2O$ (10 mL), washed with brine (10 mL), and concentrated in vacuo after filtration. The residue was purified by flash column chromatography on silica gel (petroleum ether/EtOAc = 1/1, R_f = 0.26) to afford **19** (1.5 g, 81%) as a white solid.

^{1}H NMR (400 MHz, CD_3OD): δ 7.56 (d, J = 7.8 Hz, 1H), 7.33 (d, J = 8.0 Hz, 1H), 7.10 (t, J = 6.9 Hz, 1H), 7.04

(t, J = 7.1 Hz, 1H), 5.62 (d, J = 7.5 Hz, 1H), 4.84 (q, J = 4.2 Hz, 2H), 4.12–4.05 (m, 1H), 3.87–3.76 (m, 2H), 3.71–3.61 (m, 2H), 3.52 (dd, J = 9.5, 2.1 Hz, 1H), 3.43 (d, J = 10.5 Hz, 1H), 3.04 (d, J = 7.5 Hz, 1H), 2.78 (t, J = 3.0 Hz, 1H), 2.16 (ddd, J = 13.2, 10.2, 2.8 Hz, 1H), 2.06 (dt, J = 13.7, 3.5 Hz, 1H) ppm. **^{13}C NMR** (100 MHz, CD_3OD): δ 202.3, 143.0, 137.8, 127.5, 122.6, 120.6, 119.3, 112.3, 103.9, 97.8, 77.4, 73.5, 67.4, 67.1, 64.0, 52.2, 49.8, 46.8, 33.5, 30.5 ppm. **IR** (KBr): v_{max} = 3458, 1961, 1637, 1451, 1092, 1044 cm^{-1}. **HRMS** (m/z): Calcd for $C_{20}H_{21}N_2O_2$, $[M+H]^+$, 321.1598; found: 321.1594. **m. p.**: 56.4–61.2 °C. $[\alpha]_D^{25}$: +60.0 (c 0.033, MeOH).

2.40 Preparation of 20

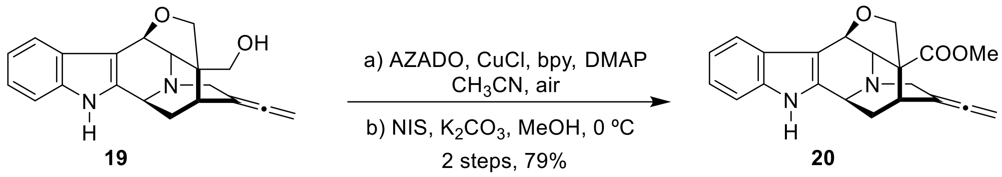

To a solution of **19** (1.9 g, 5.9 mmol) in CH_3CN (25 mL) were added AZADO (90 mg, 0.6 mmol), CuCl (117 mg, 1.2 mmol), bpy (185 mg, 0.6 mmol) and DMAP (750 mg, 3.0 mmol). The reaction mixture was stirred at room temperature for 30 min under an atmosphere of air. The reaction mixture was filtered through a pad of Celite and washed with CH_2Cl_2 (3 × 30 mL). The combined organic extracts were dried over Na_2SO_4, filtered, and concentrated. The crude product was directly used in the next reaction.

To a mixture of the above crude product in MeOH (12.5 mL) were added NIS (3.3 g, 7.4 mmol) and K_2CO_3 (1 g, 7.4 mmol). The reaction mixture was stirred at room temperature for 2 h under an atmosphere of argon in the dark before a saturated aqueous solution of $Na_2S_2O_3$ (10 mL) was added to quench the reaction. The resulting mixture was extracted with CH_2Cl_2 (3 × 20 mL), washed with brine (3 × 10 mL), dried over Na_2SO_4, filtered, and concentrated in vacuo. The crude product was purified by flash column chromatography on silica gel (petroleum ether/EtOAc = 2/1, R_f = 0.20) to product **20** (1.6 g, 79%) as a white solid.

^{1}H NMR (400 MHz, DMSO-d_6): δ 11.10 (s, 1H), 7.46 (d, J = 7.7 Hz, 1H), 7.32 (d, J = 8.0 Hz, 1H), 7.06 (t, J = 7.5 Hz, 1H), 6.99 (t, J = 7.4 Hz, 1H), 5.59 (d, J = 7.6 Hz, 1H), 4.80 (t, J = 4.4 Hz, 2H), 4.28 (d, J = 7.7 Hz, 1H), 4.00 (dd, J = 8.2, 5.3 Hz, 1H), 3.85-3.71 (m, 2H), 3.67 (s, 3H), 3.62-3.49 (m, 2H), 2.84 (t, J = 2.9 Hz, 1H), 2.01 (dd, J = 8.2, 3.2 Hz, 2H) ppm. **^{13}C NMR** (100 MHz, DMSO-d_6): δ 199.8, 175.3, 143.0, 135.7, 125.9, 120.9, 119.1, 118.1, 111.5, 102.0, 96.9, 77.2, 71.6, 67.2, 60.8, 52.8, 52.3, 50.6, 46.9, 34.4, 28.2 ppm. **IR** (KBr): v_{max} = 3450, 1961, 1636, 1451, 1261, 1092 cm^{-1}. **HRMS** (m/z): Calcd for $C_{21}H_{20}N_2NaO_3$, $[M+Na]^+$, 371.1366; found: 371.1363. **m. p.**: 100.6–104.3 °C. $[\alpha]_D^{25}$: +175.7 (c 0.047, MeOH).

2.41 Preparation of 20-1

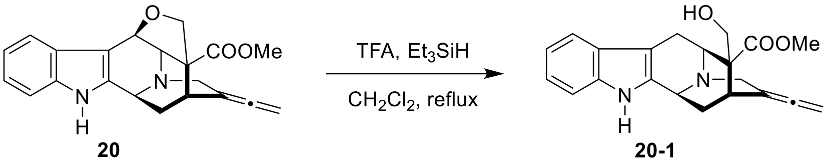

To a solution of **20** (1 g, 2.9 mmol) in CH_2Cl_2 (40 mL) were added Et_3SiH (4.6 mL, 28.7 mmol), and TFA (2.1 mL, 45.9 mmol). The reaction mixture was stirred at 40 ºC for 48 h under an atmosphere of argon. After concentration, the residue was dissolved in CH_2Cl_2 (20 mL). A saturated aqueous solution of $NaHCO_3$ (10 mL) was added to the mixture to adjust the pH value to 8. The organic phase was diluted in CH_2Cl_2 (20 mL), washed with brine (15 mL), dried over anhydrous Na_2SO_4, filtered, and concentrated in vacuo. The crude product was purified by flash column chromatography on silica gel (petroleum ether/EtOAc = 1/2, R_f = 0.10) to afford **20-1** (700.7 mg, 69%) as a white solid.

^{1}H NMR (400 MHz, $CDCl_3$): δ 8.00 (s, 1H), 7.46 (d, J = 7.4 Hz, 1H), 7.28 (d, J = 8.0 Hz, 1H), 7.18–7.06 (m, 2H), 4.71 (s, 2H), 4.32 (d, J = 5.9 Hz, 1H), 4.01 (d, J = 8.9 Hz, 1H), 3.84–3.78 (m, 1H), 3.76 (s, 3H), 3.68–3.60 (m, 2H), 3.57–3.48 (m, 1H), 3.08 (dd, J = 16.5, 6.1 Hz, 1H), 2.97–2.85 (m, 2H), 2.06–1.99 (m, 1H), 1.78 (d, J = 13.4 Hz, 1H), 1.27 (t, J = 11.3 Hz, 1H) ppm. **^{13}C NMR** (100 MHz, $CDCl_3$): δ 200.5, 176.3, 136.6, 136.3, 126.5, 121.9, 119.6, 118.5, 111.1, 106.0, 97.7, 62.9, 53.7, 53.1, 52.6, 52.0, 48.8, 34.4, 28.3, 22.4 ppm. **IR** (KBr): ν_{max} = 3483, 1961, 1728, 1638, 1451, 1160, 1044, 741 cm^{-1}. **HRMS** (m/z): Calcd for $C_{21}H_{23}N_2O_3$, $[M+H]^+$, 351.1703; found: 351.1700. **m. p.**: 121.4–135.4 °C. **$[\alpha]_D^{25}$**: +41.0 (*c* 0.033, MeOH).

2.42 Preparation of (+)-polyneuridine (8)

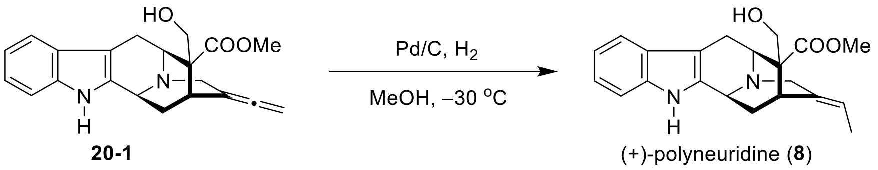

To a mixture of **20-1** (320 mg, 0.9 mmol) in MeOH (10 mL) was added Pd/C (10%, 98.1 mg, 0.09 mmol). The reaction mixture was stirred at –30 °C under an atmosphere of H_2 (balloon) for 2 h. After filtration through a pad of Celite and removal of the solvent, the crude product was purified by flash column chromatography on silica gel (dichloromethane/methanol = 10/1, R_f = 0.30) to afford (+)-polyneuridine (**8**) (278.9 mg, 88%, E/Z>20:1) as a white solid.

^{1}H NMR (400 MHz, $CDCl_3$): δ 7.96 (s, 1H), 7.48 (d, J = 7.6 Hz, 1H), 7.27 (d, J = 2.8 Hz, 1H), 7.15 (t, J = 7.5 Hz, 1H), 7.10 (t, J = 7.5 Hz, 1H), 5.27 (q, J = 6.9 Hz, 1H), 4.26 (d, J = 6.2 Hz, 1H), 3.99 (dd, J = 9.7, 4.2 Hz, 1H), 3.73

(s, 3H), 3.69–3.52 (m, 4H), 3.19 (d, J = 6.4 Hz, 1H), 3.09 (dd, J = 16.5, 6.4 Hz, 1H), 2.93 (d, J = 16.4 Hz, 1H), 1.91–1.80 (m, 2H), 1.60 (d, J = 6.7 Hz, 3H). ppm. **^{13}C NMR** (100 MHz, $CDCl_3$): δ 176.4, 136.9, 136.4, 136.2, 126.5, 121.6, 119.5, 118.4, 116.1, 110.9, 106.1, 63.2, 55.7, 53.6, 53.4, 52.2, 49.0, 30.6, 28.9, 22.4, 12.8 ppm. **IR** (KBr): ν_{max} = 3479, 1735, 1638, 1617, 1454, 1261, 1033, 801 cm^{-1}. **HRMS** (m/z): Calcd for $C_{21}H_{25}N_2O_3$, $[M+H]^+$, 353.1860; found: 353.1857. **m. p.**: 138.5–145.7 °C. **$[\alpha]_D^{25}$**: +5.9 (c 0.5, CH_2Cl_2).

2.43 Supplementary Table 9 ^{1}H NMR spectroscopic data comparison of (+)-polyneuridine (8)

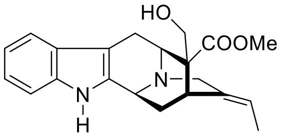

(+)-polyneuridine (**8**)

Entry	δ_H of our synthetic (+)-polyneuridine (**8**) (400 MHz, $CDCl_3$)	δ_H of Cook's synthetic (+)-polyneuridine (**8**)[7] (300 MHz, $CDCl_3$)	δ_H of natural (+)-polyneuridine (**8**)[8] (300 MHz, $CDCl_3$)	$\Delta\delta_H$ (δ_{ours} vs $\delta_{natural}$)
1	7.96 (s, 1H)	8.48 (s, 1H)	7.81 (br s, 1H)	+0.15
2	7.48 (d, J = 7.6 Hz, 1H)	7.48 (d, J = 7.5 Hz, 1H)	7.48 (d, 1H)	0
3	7.27 (d, J = 2.8 Hz, 1H)	7.34 (d, J = 7.8 Hz, 1H)	7.31 (d, 1H)	–0.04
4	7.15 (t, J = 7.5 Hz, 1H)	7.16 (t, 1H)	7.15 (t, 1H)	0
5	7.10 (t, J = 7.5 Hz, 1H)	7.10 (t, 1H)	7.10 (t, 1H)	0
6	5.27 (q, J = 6.9 Hz, 1H)	5.23 (br q, J = 6.8 Hz, 1H)	5.28 (br q, 1H)	–0.01
7	4.26 (d, J = 6.2 Hz, 1H)	4.35 (d, J = 6.2 Hz, 1H)	4.27 (br d, 1H)	–0.01
8	3.99 (dd, J = 9.7, 4.2 Hz, 1H)	4.18 (d, J = 9.0 Hz, 1H)	4.06 (dd, 1H)	–0.07
9	3.73 (s, 3H)	3.73 (s, 3H)	3.73 (s, 3H)	0
10	3.69–3.52 (m, 4H)	3.65 (d, J = 8.2 Hz 1H)<br>3.57 (m, 2H)<br>3.50 (m, 1H)	3.71–3.61 (m, 4H)	—
11	3.19 (d, J = 6.4 Hz, 1H)	3.18 (dd, 1H)	3.21 (dd, 1H)	–0.02
12	3.09 (dd, J = 16.5, 6.4 Hz, 1H)	3.12 (dd, J = 6.3 Hz, 1H)	3.10 (dd, 1H)	–0.01
13	2.93 (d, J = 16.4 Hz, 1H)	3.01 (br d, 1H)	2.94 (br d, 1H)	–0.01
14	1.91–1.80 (m, 2H)	1.95 (ddd, 1H)<br>1.85 (ddd, 1H)	1.91 (ddd, 1H)<br>1.85 (ddd, 1H)	—
15	1.60 (d, J = 6.8 Hz, 3H)	1.59 (d, J = 6.8 Hz, 3H)	1.60 (br d, 3H)	0

2.44 Supplementary Table 10 ^{13}C NMR spectroscopic data comparison of (+)-polyneuridine (8)

Entry	δ_C of our synthetic (+)-polyneuridine (**8**) (100 MHz, $CDCl_3$)	δ_C of Cook's synthetic (+)-polyneuridine (**8**)[7] (75 MHz, $CDCl_3$)	δ_C of natural (+)-polyneuridine (**8**)[8] (75 MHz, $CDCl_3$)	$\Delta\delta_C$ (δ_{ours} vs $\delta_{natural}$)
1	176.4	175.7	176.4	0
2	136.9	136.1	136.9	0
3	136.4	135.8	136.5	–0.1
4	136.2	134.5	136.2	0

5	126.5	126.1	126.5	0
6	121.6	121.7	121.6	0
7	119.5	119.4	119.5	0
8	118.4	118.2	118.3	+0.1
9	116.1	116.7	116.0	+0.1
10	110.9	111.1	110.8	+0.1
11	106.1	105.6	106.2	–0.1
12	63.2	63.1	63.2	0
13	55.7	55.1	55.8	–0.1
14	53.6	53.6	53.6	0
15	53.4	53.3	53.4	0
16	52.2	52.2	52.2	0
17	49.0	48.8	49.0	0
18	30.6	30.4	30.6	0
19	28.9	28.6	28.9	0
20	22.4	21.9	22.3	+0.1
21	12.8	12.6	12.7	+0.1

2.45 Preparation of (–)-macusine A (9)

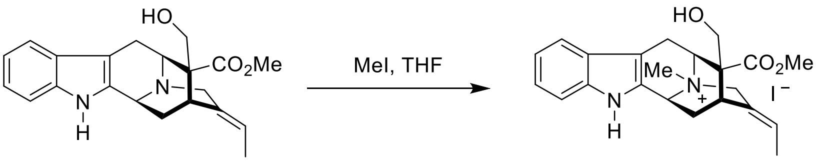

(+)-polyneuridine (**8**) (–)-macusine A (**9**)

To a solution of (+)-polyneuridine (**8**) (200.7 mg, 0.57 mmol) in THF (10 mL) was added MeI at 0 °C under an atmosphere of argon. The reaction was stirred at room temperature for about 10 h, and the resulting solution was extracted with EtOAc (3 × 15 mL). The organic phase was washed with brine (3 × 10 mL), dried over Na_2SO_4, filtered, and concentrated under reduced pressure. The residue was purified by flash column chromatography on silica gel (dichloromethane/methanol = 10/1, R_f = 0.20) to afford (–)-macusine A (**9**) (236.6 mg, 84%) as a white solid.

^{1}H NMR (400 MHz, CD_3OD): δ 7.56 (d, J = 7.9 Hz, 1H), 7.42 (d, J = 8.2 Hz, 1H), 7.22 (t, J = 7.6 Hz, 1H), 7.12 (t, J = 7.5 Hz, 1H), 5.50 (q, J = 6.8 Hz, 1H), 5.03 (d, J = 6.4 Hz, 1H), 4.95 (d, J = 10.5 Hz, 1H), 4.49 (d, J = 16.2 Hz, 1H), 4.31 (d, J = 16.2 Hz, 1H), 3.87 (d, J = 17.9 Hz, 1H), 3.78 (s, 3H), 3.76–3.73 (m, 1H), 3.63–3.67 (m, 1H), 3.42–3.39 (m, 1H), 3.39–3.35 (m, 1H), 3.26 (s, 3H), 2.48 (ddd, J = 13.5, 11.0, 2.3 Hz, 1H), 2.17–2.11 (m, 1H), 1.71 (d, J = 6.9 Hz, 3H) ppm. **^{13}C NMR** (100 MHz, CD_3OD): δ 172.7, 137.2, 130.2, 127.2, 124.8, 122.5, 119.5, 119.4, 118.2, 111.2, 101.6, 64.7, 64.0, 62.4, 59.1, 55.2, 51.8, 48.5, 30.0, 28.6, 18.6, 11.3. ppm. **IR** (KBr): ν_{max} = 3481, 1732, 1645, 1453, 1268, 1074, 1016 cm^{-1}. **HRMS** (m/z): Calcd for $C_{22}H_{27}N_2O_3$, $[M–I]^+$, 367.2013; found: 367.2016. **m. p.**: 246.5–248.1 °C. **$[\alpha]_D^{25}$**: –48.6 (c 0.1, MeOH).

2.46 Supplementary Table 11 ^{1}H NMR spectroscopic data comparison of (–)-macusine A (9)

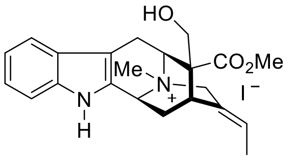

(–)-macusine A (**9**)

Entry	δ_H of our synthetic (–)-macusine A (**9**) (400 MHz, CD_3OD)	δ_H of Cook's synthetic (–)-macusine A (**9**)[7] (300 MHz, CD_3OD)	$\Delta\delta_H$ (δ_{ours} vs $\delta_{Cook's}$)
1	7.56 (d, *J* = 7.9 Hz, 1H)	7.57 (d, *J* = 8.6 Hz, 1H)	–0.01
2	7.42 (d, *J* = 8.2 Hz, 1H)	7.42 (d, *J* = 8.2 Hz, 1H)	0
3	7.22(t, *J* = 7.8 Hz, 1H)	7.23 (t, 1H)	–0.01
4	7.12 (t, *J* = 7.5 Hz, 1H)	7.12 (t, 1H)	0
5	5.50 (q, *J* = 6.8 Hz, 1H)	5.51 (q, 1H)	–0.01
6	5.03 (d, *J* = 6.4 Hz, 1H)	5.04 (d, *J* = 5.9 Hz, 1H)	–0.01
7	4.95 (d, *J* = 10.5 Hz, 1H)	4.93 (m, 1H)	+0.02
8	4.49 (d, *J* = 16.2 Hz, 1H)	4.48 (d, *J* = 20.3 Hz, 1H)	+0.01
9	4.31 (d, *J* = 16.2 Hz, 1H)	4.29 (d, *J* = 18.0 Hz, 1H)	+0.02
10	3.87 (d, *J* = 17.9 Hz, 1H)		—
11	3.78 (s, 3H)	3.78 (s, 3H)	0
12	3.76–3.73 (m, 1H),	3.70 (m, 2H)	—
13	3.63–3.67 (m, 1H)	3.42 (m, 2H)	—
14	3.39–3.35 (m, 1H)	3.36–3.31 (m, 1H)	—
15	3.26 (s, 3H)	3.26 (s, 3H)	0
16	2.48 (ddd, *J* = 13.5, 10.9, 2.2 Hz, 1H)	2.47 (m, 1H)	+0.01
17	2.17–2.11 (m, 1H)	2.15 (m, 1H)	—
18	1.71 (d, *J* = 6.9 Hz, 3H)	1.71 (d, *J* = 11.0 Hz, 3H)	0

2.47 Supplementary Table 12 ^{13}C NMR spectroscopic data comparison of (–)-macusine A (9)

Entry	δ_C of our synthetic (–)-macusine A (**9**) (100 MHz, CD_3OD)	δ_C of Cook's synthetic (–)-macusine A (**9**)[7] (75 MHz, CD_3OD)	$\Delta\delta_C$ (δ_{ours} vs $\delta_{Cook's}$)
1	172.7	172.7	0
2	137.2	137.1	+0.1
3	130.2	130.0	+0.2
4	127.2	127.2	0
5	124.8	124.8	0
6	122.5	122.5	0
7	119.5	119.5	0
8	119.4	119.3	+0.1
9	118.2	118.2	0
10	111.2	111.1	+0.1
11	101.6	101.6	0
12	64.7	64.6	+1.6
13	64.0	63.9	+0.1
14	62.4	62.4	0

15	59.1	59.1	0
16	55.2	55.1	+0.1
17	51.8	51.8	0
18	48.5	29.9	+18.6
19	30.0	29.2	+0.8
20	28.6	28.6	0
21	18.6	18.6	0
22	11.3	11.3	0

2.48 Preparation of 21

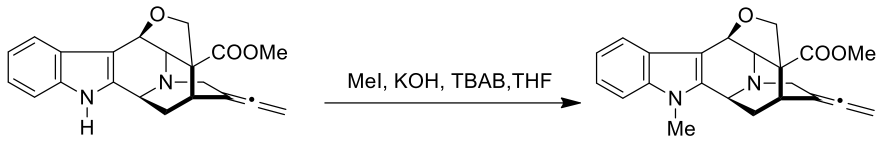

20 **21**

To a solution of **20** (1.5 g, 4.3 mmol) in THF (22 mL) were added KOH (319.2 mg, 5.7 mmol), TBAB (29 mg, 0.09 mmol), and MeI (0.4 mL, 5.7 mmol). The reaction mixture was stirred at room temperature under an atmosphere of argon. After being stirred for 1 h, the reaction mixture was filtered through a pad of Celite and washed with CH_2Cl_2 (50 mL). After concentration, the residue was dissolved in CH_2Cl_2 (50 mL), washed with H_2O (20 mL), and a saturated aqueous solution of $NaHCO_3$ (20 mL). The combined organic layer was dried over Na_2SO_4, filtered, and concentrated. The crude product was purified by flash column chromatography on silica gel (petroleum ether/EtOAc = 2/1, R_f = 0.24) to afford **21** (1.1 g, 73%) as a white solid.

^{1}H NMR (400 MHz, $CDCl_3$): δ 7.68 (d, J = 7.8 Hz, 1H), 7.30 (d, J = 8.1 Hz, 1H), 7.23 (t, J = 7.7 Hz, 1H), 7.15 (t, J = 7.4 Hz, 1H), 5.78 (d, J = 7.7 Hz, 1H), 4.73 (t, J = 4.4 Hz, 2H), 4.54 (d, J = 7.7 Hz, 1H), 4.07 (dd, J = 10.3, 3.4 Hz, 1H), 3.95 (d, J = 10.2 Hz, 1H), 3.82 (q, J = 4.1 Hz, 2H), 3.75 (s, 3H), 3.69–3.61 (m, 4H),, 2.97 (t, J = 3.0 Hz, 1H), 2.16 (ddd, J = 13.2, 10.3, 2.8 Hz, 1H), 2.00 (dt, J = 13.6, 3.6 Hz, 1H) ppm. **^{13}C NMR** (100 MHz, $CDCl_3$): δ 200.7, 175.7, 143.1, 137.5, 126.2, 121.8, 120.1, 119.1, 109.2, 103.2, 96.6, 77.1, 72.5, 68.2, 61.4, 53.4, 52.7, 51.9, 46.8, 34.8, 29.4, 28.6 ppm. **IR** (KBr): ν_{max} = 1961, 1636, 1452, 1261, 1092, 1021, 800 cm^{-1}. **HRMS** (m/z): Calcd for $C_{22}H_{21}N_2O_3$, $[M–H]^-$, 361.1555; found: 361.1557. **m. p.**: 202.3–207.5 °C. $[\alpha]_D^{25}$: -21.5 (*c* 0.08, MeOH).

2.49 Preparation of (+)-dehydrovoachalotine (10)

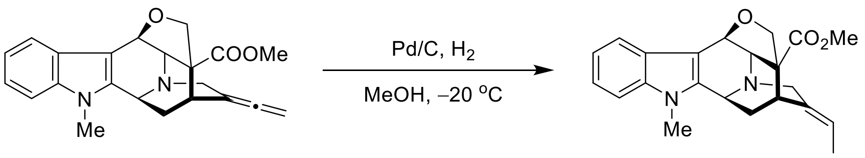

21 (+)-dehydrovoachalotine (**10**)

To a mixture of **21** (289.7 mg, 0.8 mmol) in MeOH (15 mL) was added Pd/C (10%, 106.0 mg, 0.1 mmol). The

reaction was stirred at –20 °C under an atmosphere of H_2 (balloon) for 4 h. After the filtration through a pad of Celite and removal of the solvent, the crude product was purified by flash column chromatography on silica gel (dichloromethane/methanol = 10/1, R_f = 0.2) to afford (+)-dehydrmmovoachalotine (**10**) (241.8 mg, 83%, E/Z>20:1) as a white solid.

^{1}H NMR (400 MHz, $CDCl_3$): δ 7.69 (d, J = 7.9 Hz, 1H), 7.31 (d, J = 8.2 Hz, 1H), 7.23 (t, J = 7.6 Hz, 1H), 7.16 (t, J = 6.8 Hz, 1H), 5.79 (d, J = 7.5 Hz, 1H), 5.36 (q, J = 6.9, 5.9 Hz, 1H), 4.51 (d, J = 7.4 Hz, 1H), 4.06 (d, J = 7.2 Hz, 1H), 3.94 (dd, J = 10.2, 2.1 Hz, 1H), 3.76–3.68 (m, 6H), 3.65 (s, 3H), 3.29 (t, J = 2.8 Hz, 1H), 2.09–1.93 (m, 2H), 1.61 (d, J = 6.7 Hz, 3H). ppm. **^{13}C NMR** (100 MHz, $CDCl_3$): δ 175.9, 143.4, 137.5, 135.4, 126.3, 121.7, 120.0, 119.1, 116.7, 109.1, 103.3, 72.6, 68.3, 61.4, 55.4, 53.9, 52.4, 47.0, 30.9, 29.4, 29.1, 12.9 ppm. **IR** (KBr): ν_{max} = 1730, 1617, 1468, 1454, 1241, 1214, 1092, 744 cm^{-1}. **HRMS** (m/z): Calcd for $C_{22}H_{23}N_2O_3$, $[M–H]^-$, 363.1714; found: 363.1709. **m.p.**: 237.3–240.1 ºC. **$[\alpha]_D^{25}$**: +122.0 (c 0.9, MeOH).

2.50 Supplementary Table 13 ^{1}H NMR spectroscopic data comparison of (+)-dehydrovoachalotine (10)

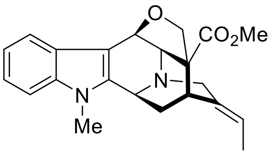

(+)-dehydrovoachalotine (**10**)

Entry	δ_H of our synthetic (+)-dehydrovoachalotine (**10**) (400 MHz, $CDCl_3$)	δ_H of Cook's synthetic (+)-dehydrovoachalotine (**10**)[9] (300 Hz, $CDCl_3$)	$\Delta\delta_H$ (δ_{ours} vs $\delta_{Cook's}$)
1	7.69 (d, J = 7.9 Hz, 1H)	7.70 (d, J = 7.5 Hz, 1H)	–0.01
2	7.31 (d, J = 8.2 Hz, 1H)<br>7.23 (t, J = 7.4 Hz, 1H)<br>7.16 (t, J = 7.4 Hz, 1H)	7.14–7.33 (m, 3H)	—
3	5.79 (d, J = 7.5 Hz, 1H)	5.78 (d, J = 7.7 Hz, 1H)	+0.01
4	5.36 (q, J = 6.9, 5.9 Hz, 1H)	5.36 (q, J = 7.6 Hz, 1H)	0
5	4.51 (d, J = 7.4 Hz, 1H)	4.52 (d, J = 7.7Hz, 1H)	–0.01
6	4.06 (d, J = 7.2 Hz, 1H)	4.07 (dd J = 9.4, 4.2 Hz, 1H)	–0.01
7	3.94 (dd, J = 10.2, 2.1 Hz, 1H)	3.94 (d, J = 10.1 Hz, 1H)	0
8	3.76–3.68 (m, 6H)	3.76–3.71 (m, 3H)<br>3.72 (s, 3H)	—
9	3.65 (s, 3H)	3.65 (s, 3H)	0
10	3.31–3.25 (m, 1H)	2.29 (t, J = 2.9 Hz, 1H)	—
11	2.09–1.93 (m, 2H)	2.03 (m, 2H)	—
12	1.61 (d, J = 7.1 Hz, 3H)	1.60 (d, J = 7.9 Hz, 3H)	+0.01

2.51 Supplementary Table 14 ^{13}C NMR spectroscopic data comparison of (+)-dehydrovoachalotine (10)

Entry	δ_C of our synthetic (+)-dehydrovoachalotine (**10**) (100 MHz, $CDCl_3$)	δ_C of Cook's synthetic (+)-dehydrovoachalotine (**10**)[9] (75 Hz, $CDCl_3$)	$\Delta\delta_C$ (δ_{ours} vs $\delta_{Cook's}$)
1	175.9	175.9	0
2	143.4	143.3	+0.1
3	137.5	137.7	–0.2
4	135.4	135.7	–0.3
5	126.3	126.4	–0.1
6	121.7	121.8	–0.1
7	120.0	120.1	–0.1
8	119.1	119.2	–0.1
9	116.7	116.5	+0.2
10	109.1	109.1	0
11	103.3	103.4	–0.1
12	72.6	72.6	0
13	68.3	68.3	0
14	61.4	61.4	0
15	55.4	55.4	0
16	53.9	53.9	0
17	52.4	52.2	+0.2
18	47.0	47.1	–0.1
19	30.9	31.0	–0.1
20	29.4	29.3	+0.1
21	29.1	29.1	0
22	12.9	12.6	+0.3

3. Supplementary Figures

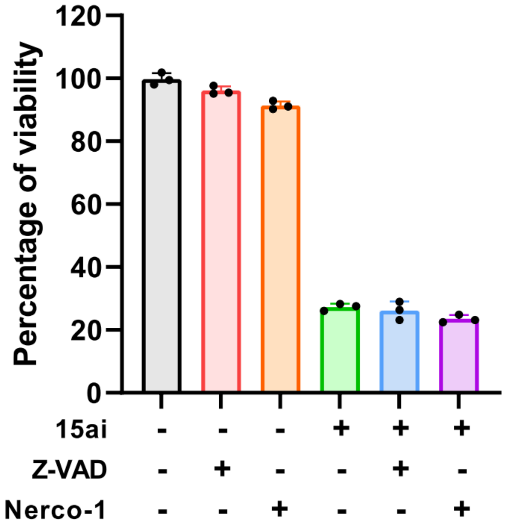

Supplementary Fig. 1 The percentage of cell viability after the combination of **15ai** (10 μM) with Z-VAD (10 μM) or Nerco-1 (10 μM) for 72 h. Data are presented as mean values ± SD, and n = 3 biologically independent replicates.

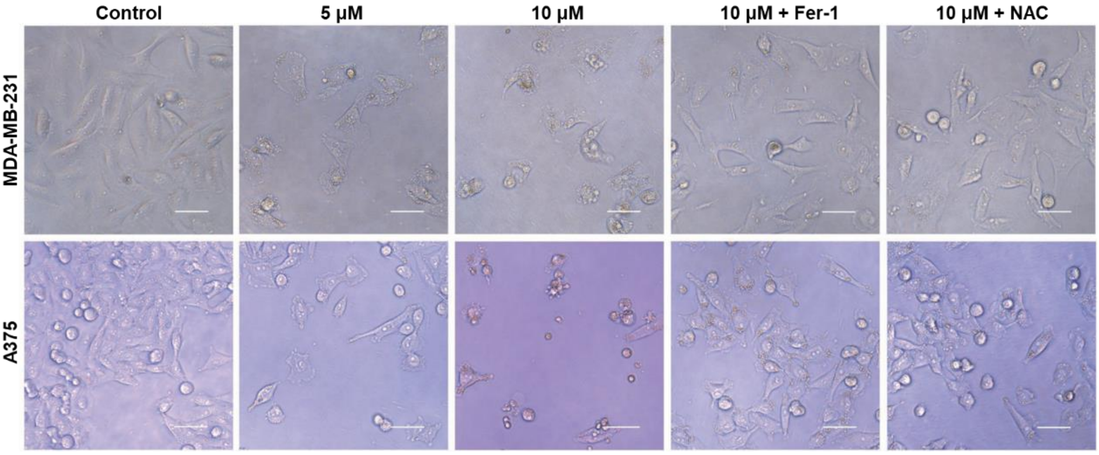

Supplementary Fig. 2 MDA-MB-231 and A375 cells treated with **15ai** alone or in combination with Fer-1 (10 μM) or NAC (5 mM). The morphology of cells upon different treatments was indicated for 72 h. Scale bar, 100 μm.

4. NMR spectra

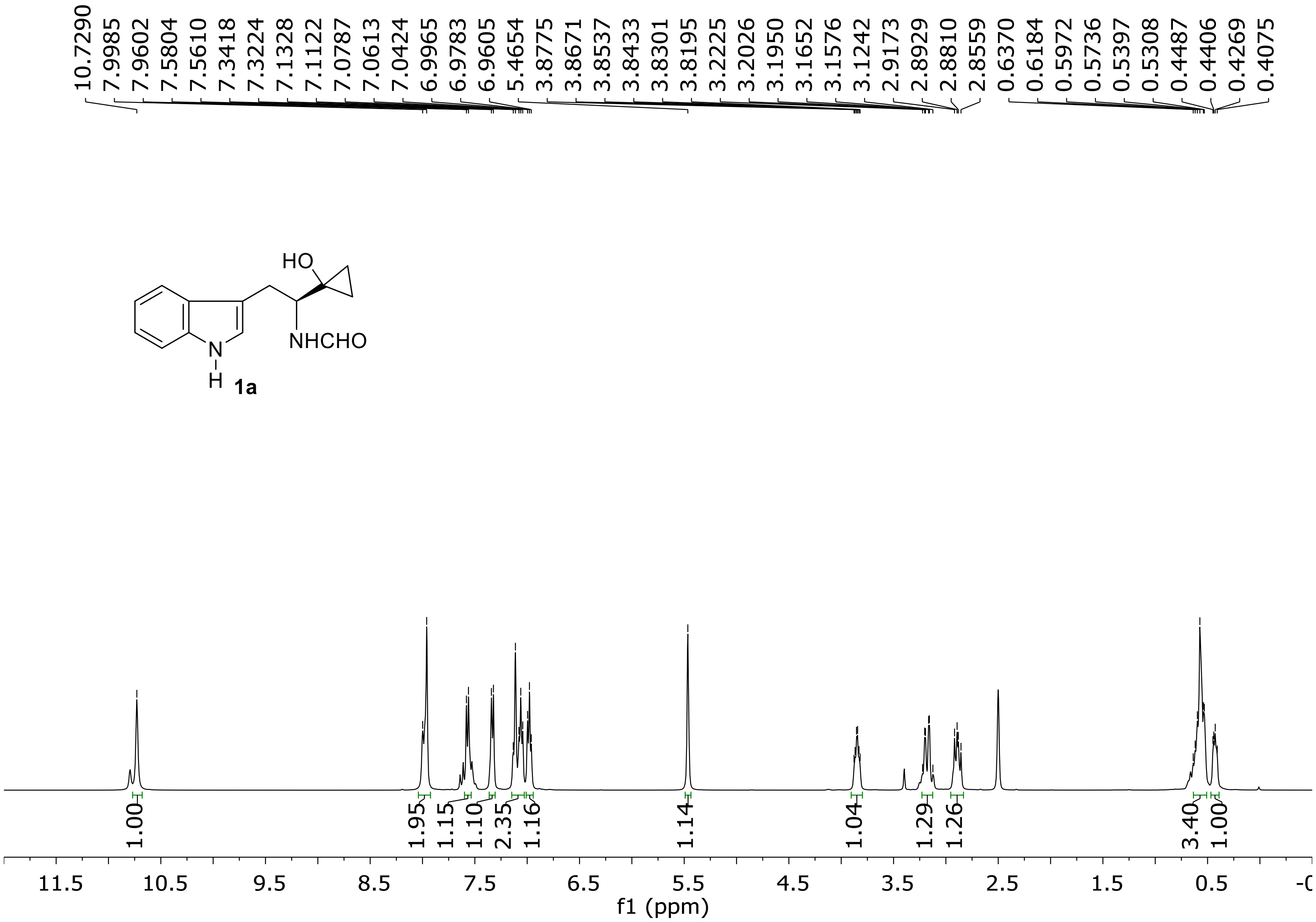

10.7290
7.9985
7.9602
7.5804
7.5610
7.3418
7.3224
7.1328
7.1122
7.0787
7.0613
7.0424
6.9965
6.9783
6.9605
5.4654
3.8775
3.8671
3.8537
3.8433
3.8301
3.8195
3.2225
3.2026
3.1950
3.1652
3.1576
3.1242
2.9173
2.8929
2.8810
2.8559
0.6370
0.6184
0.5972
0.5736
0.5397
0.5308
0.4487
0.4406
0.4269
0.4075
HO
NHCHO
N
H
1a
1.00
1.95
1.15
1.10
2.35
1.16
1.14
1.04
1.29
1.26
3.40
1.00
11.5
10.5
9.5
8.5
7.5
6.5
5.5
4.5
3.5
2.5
1.5
0.5
f1 (ppm)

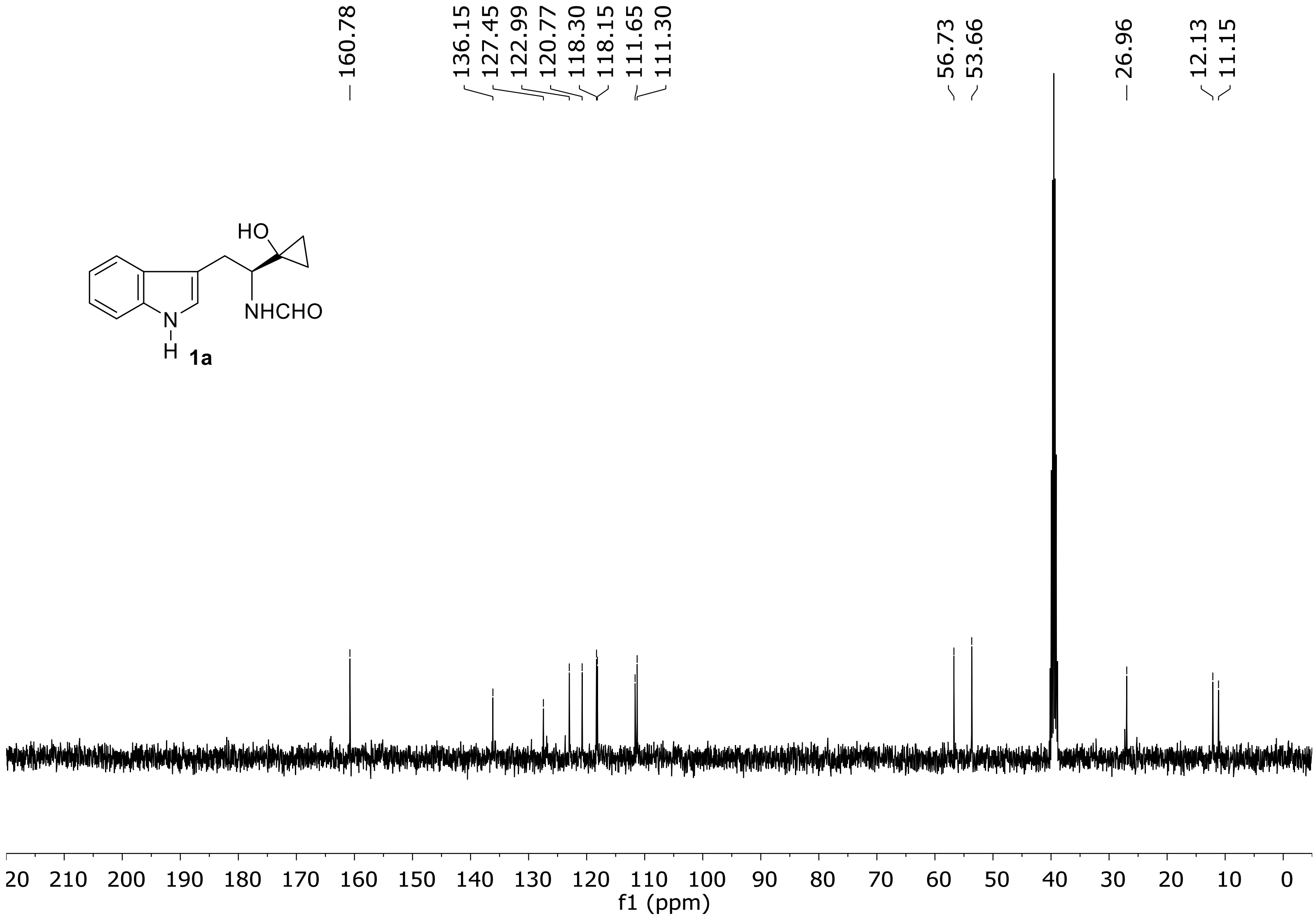
160.78
136.15
127.45
122.99
120.77
118.30
118.15
111.65
111.30
56.73
53.66
26.96
12.13
11.15
HO
NHCHO
N
H
1a
20 210 200 190 180 170 160 150 140 130 120 110 100 90 80 70 60 50 40 30 20 10 0
f1 (ppm)

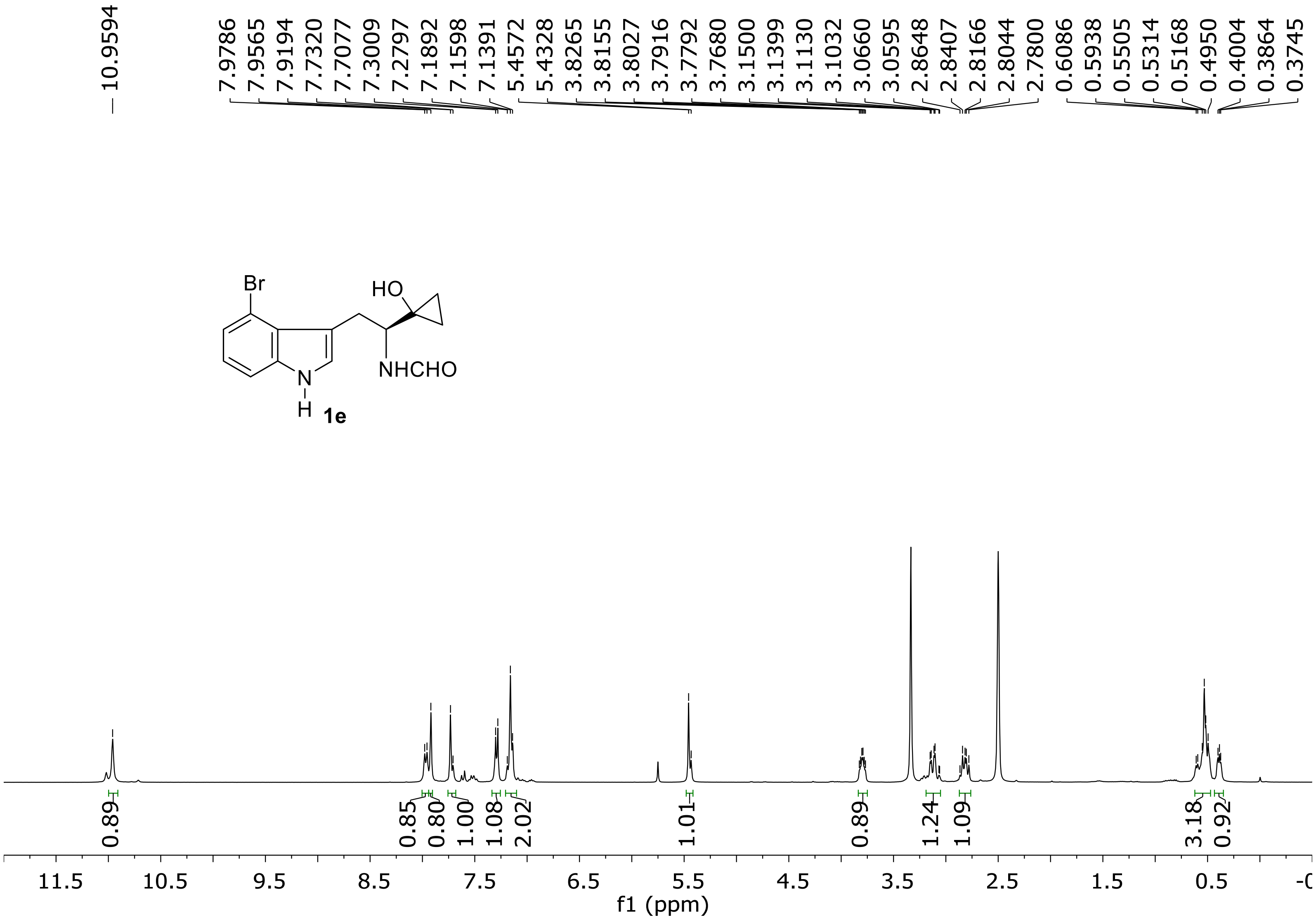
Br
HO
N
H
NHCHO
1e
10.9594
7.9786
7.9565
7.9194
7.7320
7.7077
7.3009
7.2797
7.1892
7.1598
7.1391
5.4572
5.4328
3.8265
3.8155
3.8027
3.7916
3.7792
3.7680
3.1500
3.1399
3.1130
3.1032
3.0660
3.0595
2.8648
2.8407
2.8166
2.8044
2.7800
0.6086
0.5938
0.5505
0.5314
0.5168
0.4950
0.4004
0.3864
0.3745
0.89
0.85
0.80
1.00
1.08
2.02
1.01
0.89
1.24
1.09
3.18
0.92
11.5
10.5
9.5
8.5
7.5
6.5
5.5
4.5
3.5
2.5
1.5
0.5
f1 (ppm)

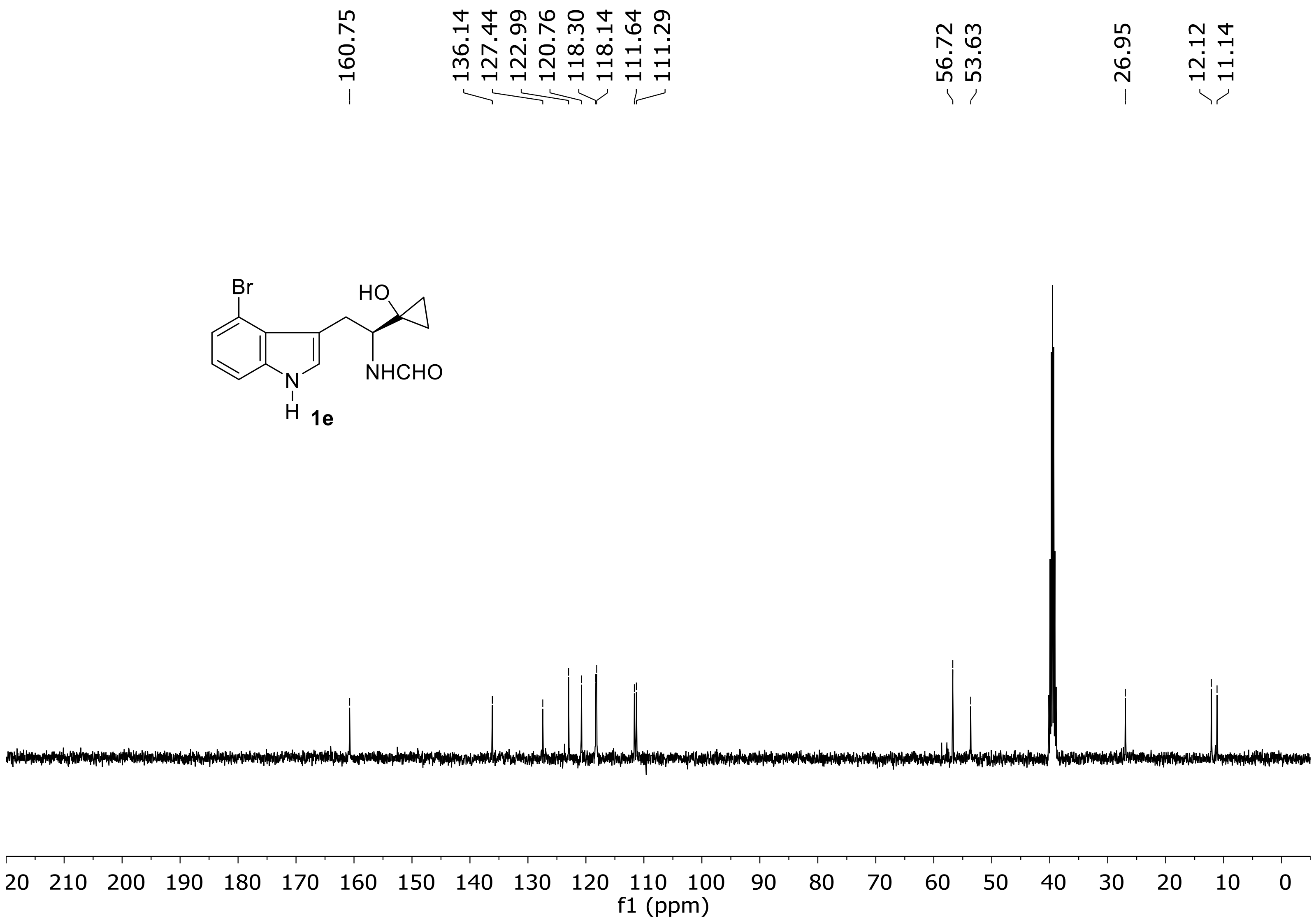
160.75
136.14
127.44
122.99
120.76
118.30
118.14
111.64
111.29
56.72
53.63
26.95
12.12
11.14
Br
HO
NHCHO
N
H
1e
20
210
200
190
180
170
160
150
140
130
120
110
100
90
80
70
60
50
40
30
20
10
0
f1 (ppm)

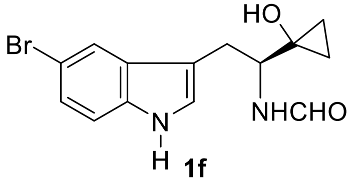
HO
Br
N
H
NHCHO

1f

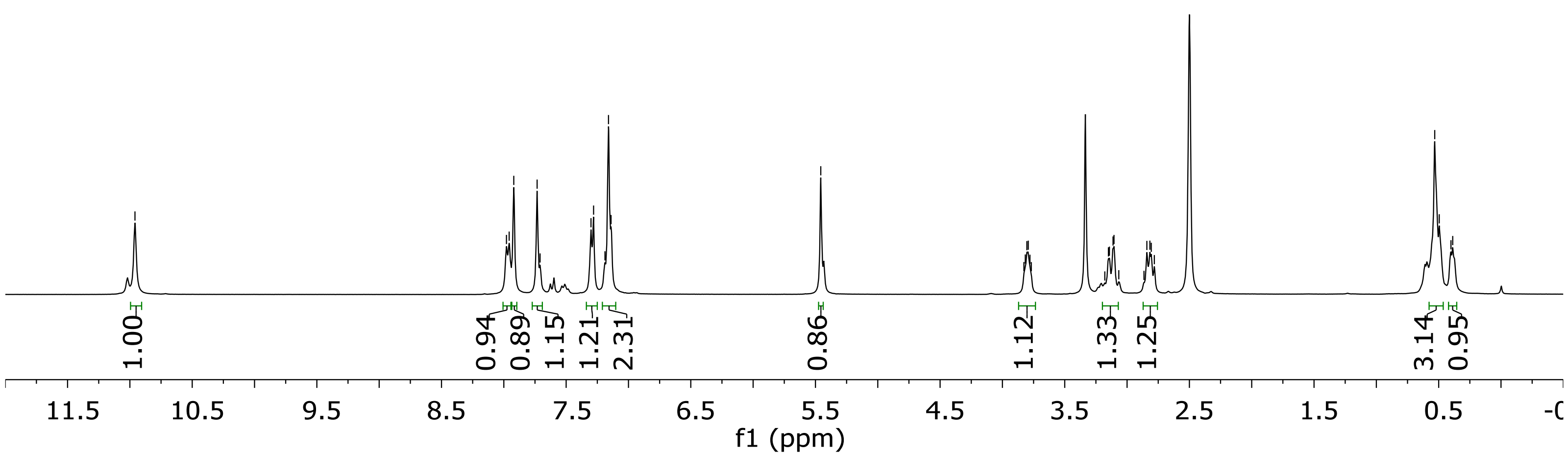
10.9592
7.9788
7.9573
7.9202
7.7330
7.7093
7.3007
7.2799
7.1886
7.1603
7.1399
5.4573
3.8275
3.8165
3.8040
3.7925
3.7792
3.7683
3.1496
3.1415
3.1125
3.1048
3.0663
2.8643
2.8412
2.8169
2.8058
2.7810
0.5317
0.4957
0.4015
1.00
0.94
0.89
1.15
1.21
2.31
0.86
1.12
1.33
1.25
3.14
0.95
11.5
10.5
9.5
8.5
7.5
6.5
5.5
4.5
3.5
2.5
1.5
0.5
f1 (ppm)

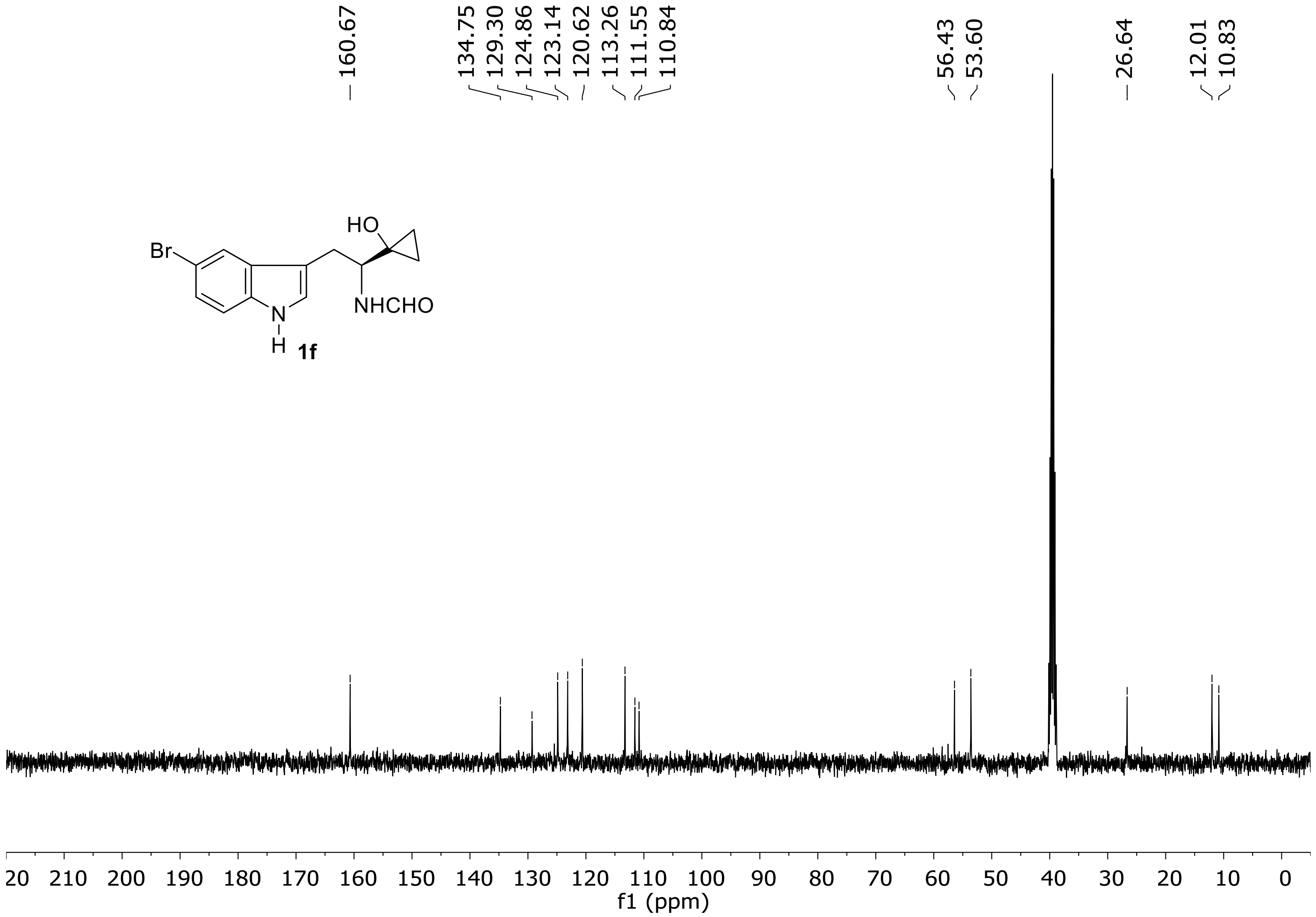
160.67
134.75
129.30
124.86
123.14
120.62
113.26
111.55
110.84
56.43
53.60
26.64
12.01
10.83
Br
HO
NHCHO
N
H
1f
20 210 200 190 180 170 160 150 140 130 120 110 100 90 80 70 60 50 40 30 20 10 0
f1 (ppm)

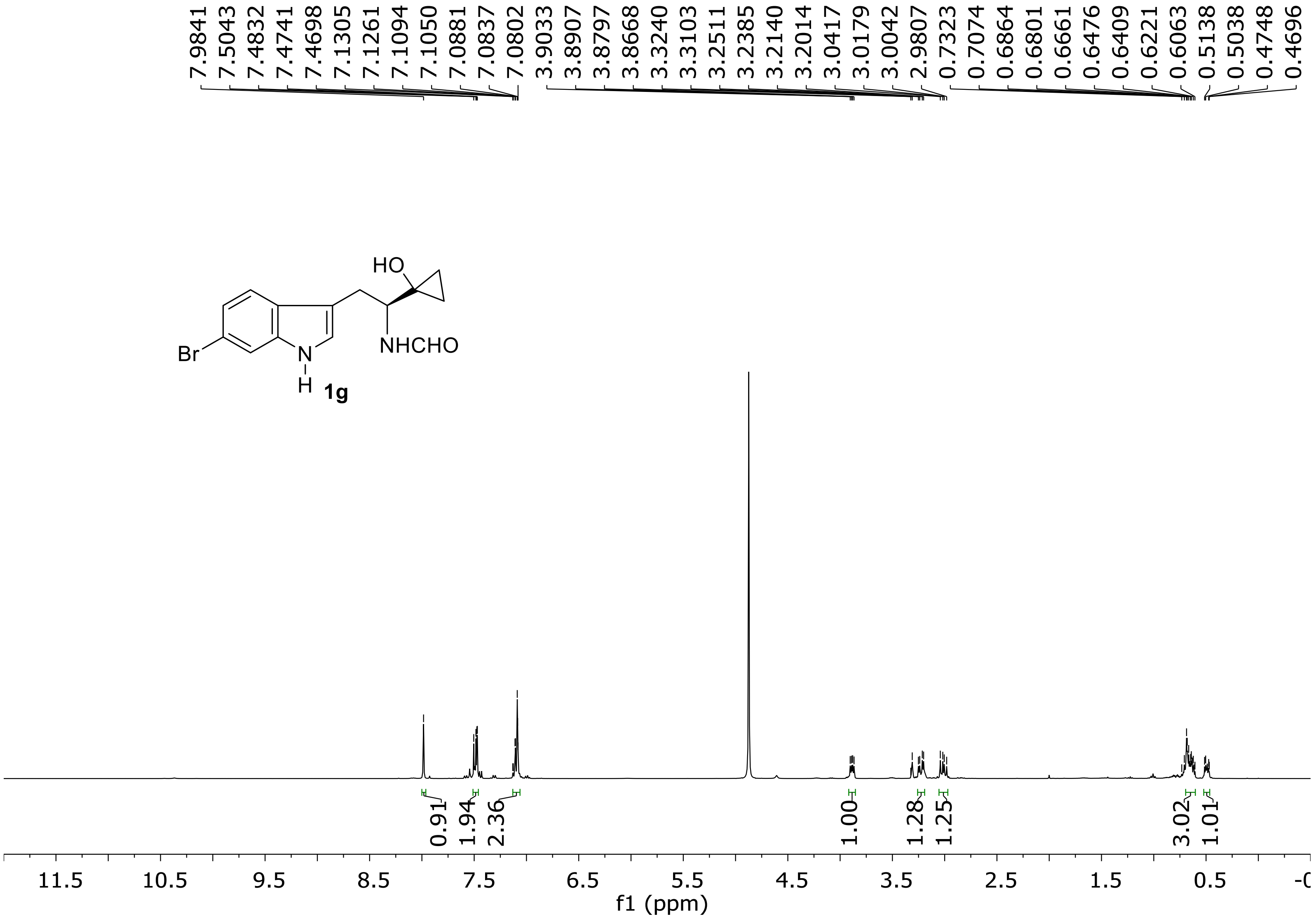
7.9841
7.5043
7.4832
7.4741
7.4698
7.1305
7.1261
7.1094
7.1050
7.0881
7.0837
7.0802
3.9033
3.8907
3.8797
3.8668
3.3240
3.3103
3.2511
3.2385
3.2140
3.2014
3.0417
3.0179
3.0042
2.9807
0.7323
0.7074
0.6864
0.6801
0.6661
0.6476
0.6409
0.6221
0.6063
0.5138
0.5038
0.4748
0.4696
HO
NHCHO
Br
N
H
1g
0.91
1.94
2.36
1.00
1.28
1.25
3.02
1.01
11.5
10.5
9.5
8.5
7.5
6.5
5.5
4.5
3.5
2.5
1.5
0.5
f1 (ppm)

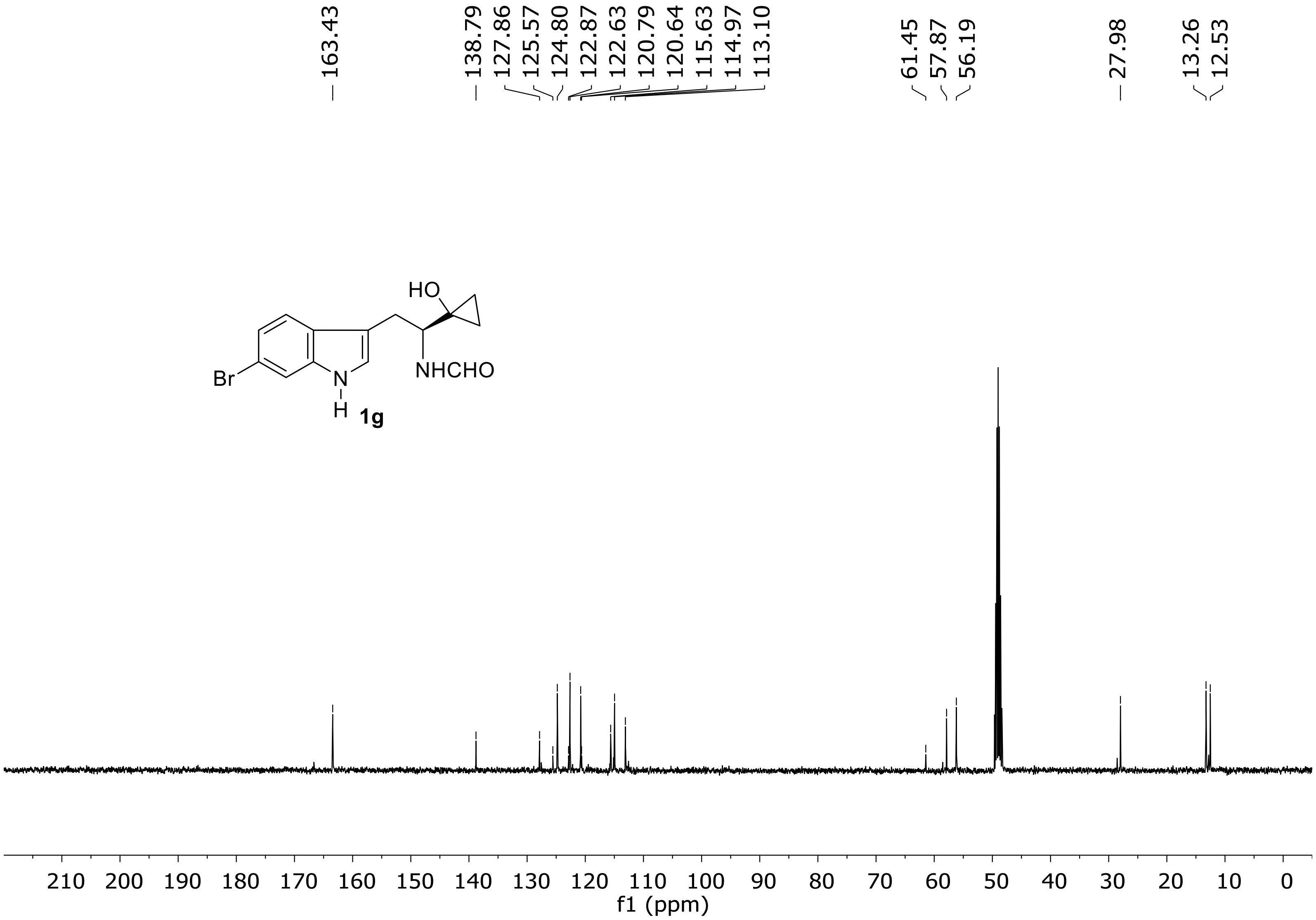

163.43
138.79
127.86
125.57
124.80
122.87
122.63
120.79
120.64
115.63
114.97
113.10
61.45
57.87
56.19
27.98
13.26
12.53
HO
Br
N
H
NHCHO
1g
210 200 190 180 170 160 150 140 130 120 110 100 90 80 70 60 50 40 30 20 10 0
f1 (ppm)

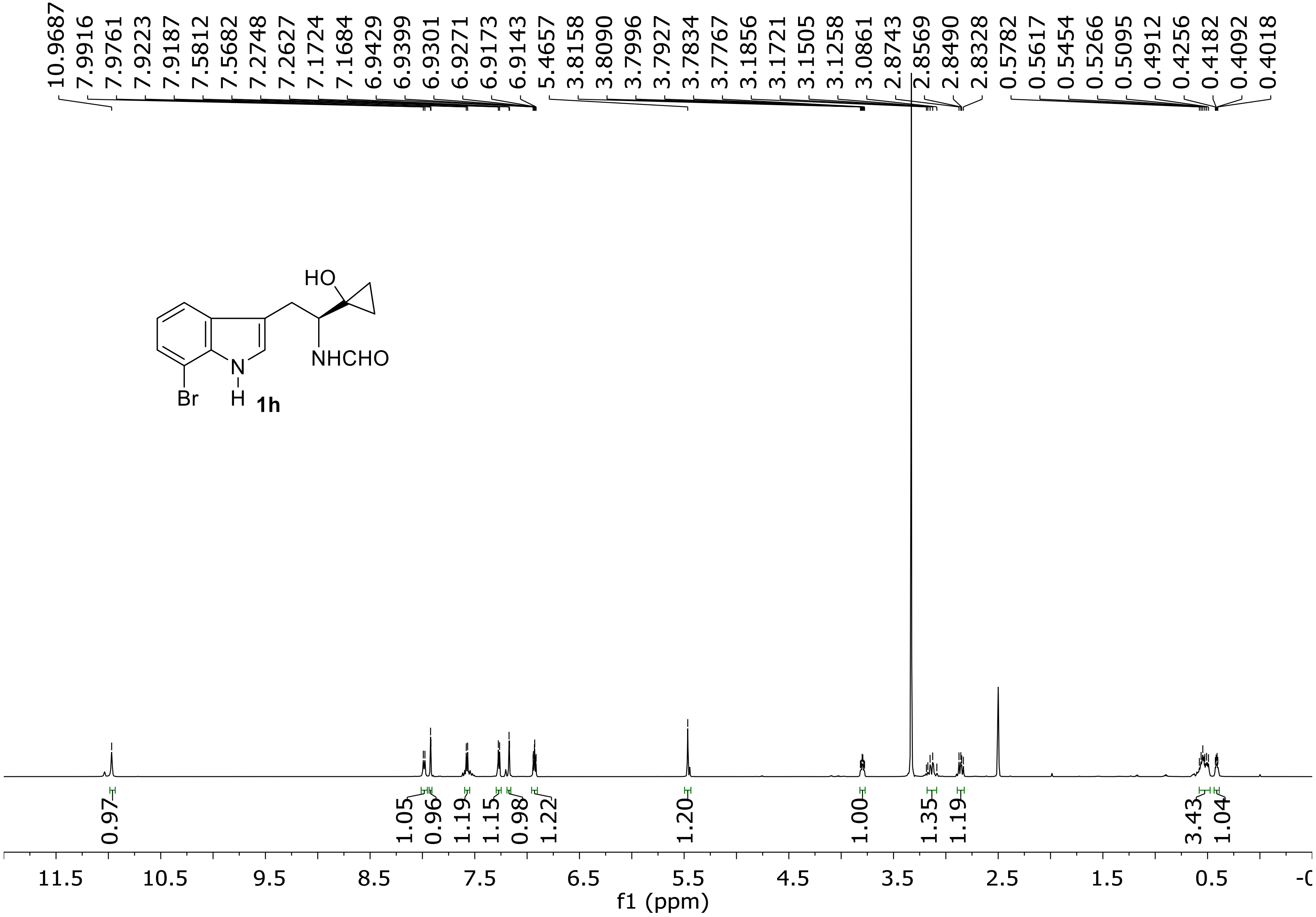
10.9687
7.9916
7.9761
7.9223
7.9187
7.5812
7.5682
7.2748
7.2627
7.1724
7.1684
6.9429
6.9399
6.9301
6.9271
6.9173
6.9143
5.4657
3.8158
3.8090
3.7996
3.7927
3.7834
3.7767
3.1856
3.1721
3.1505
3.1258
3.0861
2.8743
2.8569
2.8490
2.8328
0.5782
0.5617
0.5454
0.5266
0.5095
0.4912
0.4256
0.4182
0.4092
0.4018
HO
NHCHO
N
H
Br
1h
0.97
1.05
0.96
1.19
1.15
0.98
1.22
1.20
1.00
1.35
1.19
3.43
1.04
f1 (ppm)

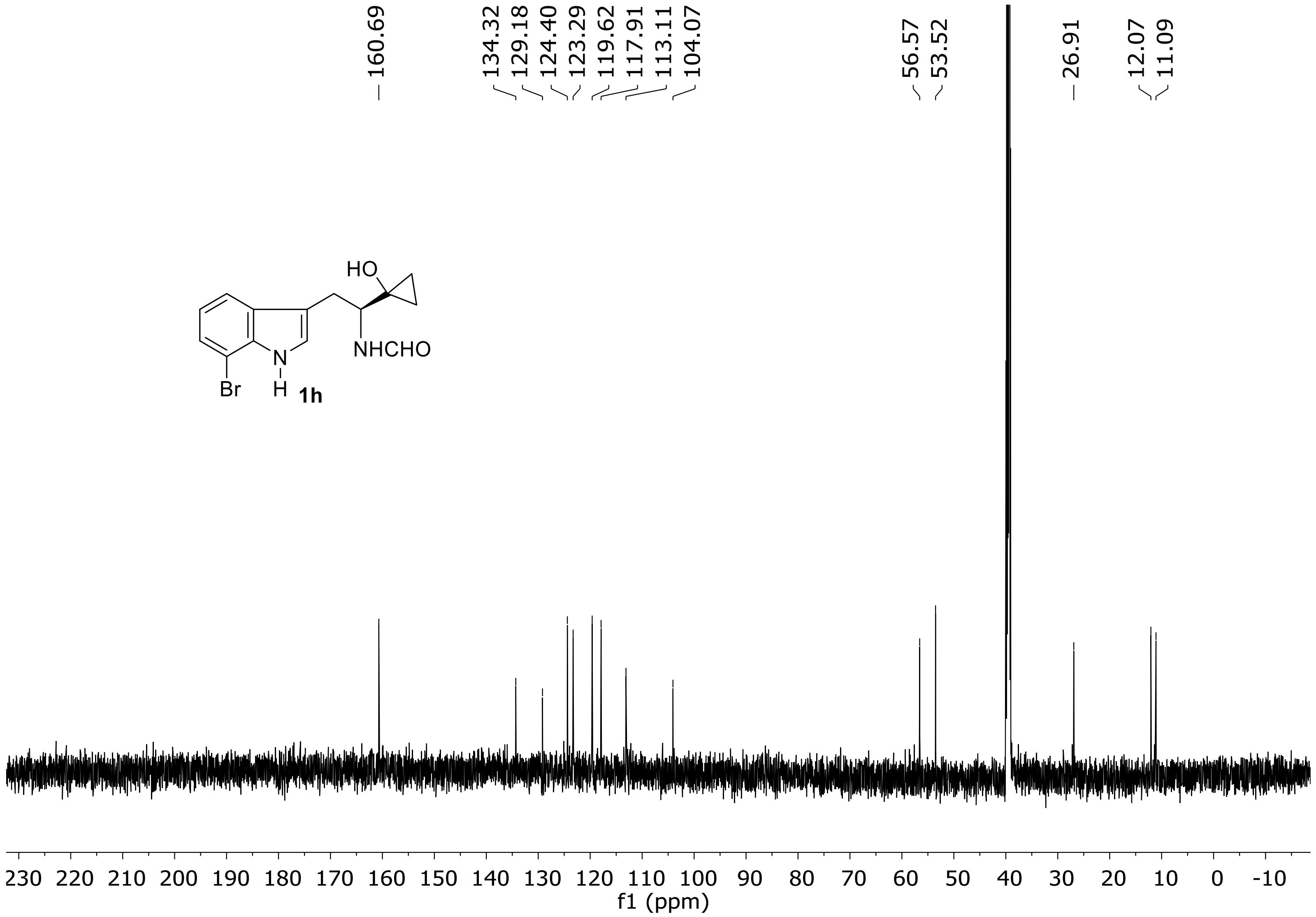
160.69
134.32
129.18
124.40
123.29
119.62
117.91
113.11
104.07
56.57
53.52
26.91
12.07
11.09
HO
NHCHO
Br
1h
230 220 210 200 190 180 170 160 150 140 130 120 110 100 90 80 70 60 50 40 30 20 10 0 -10
f1 (ppm)

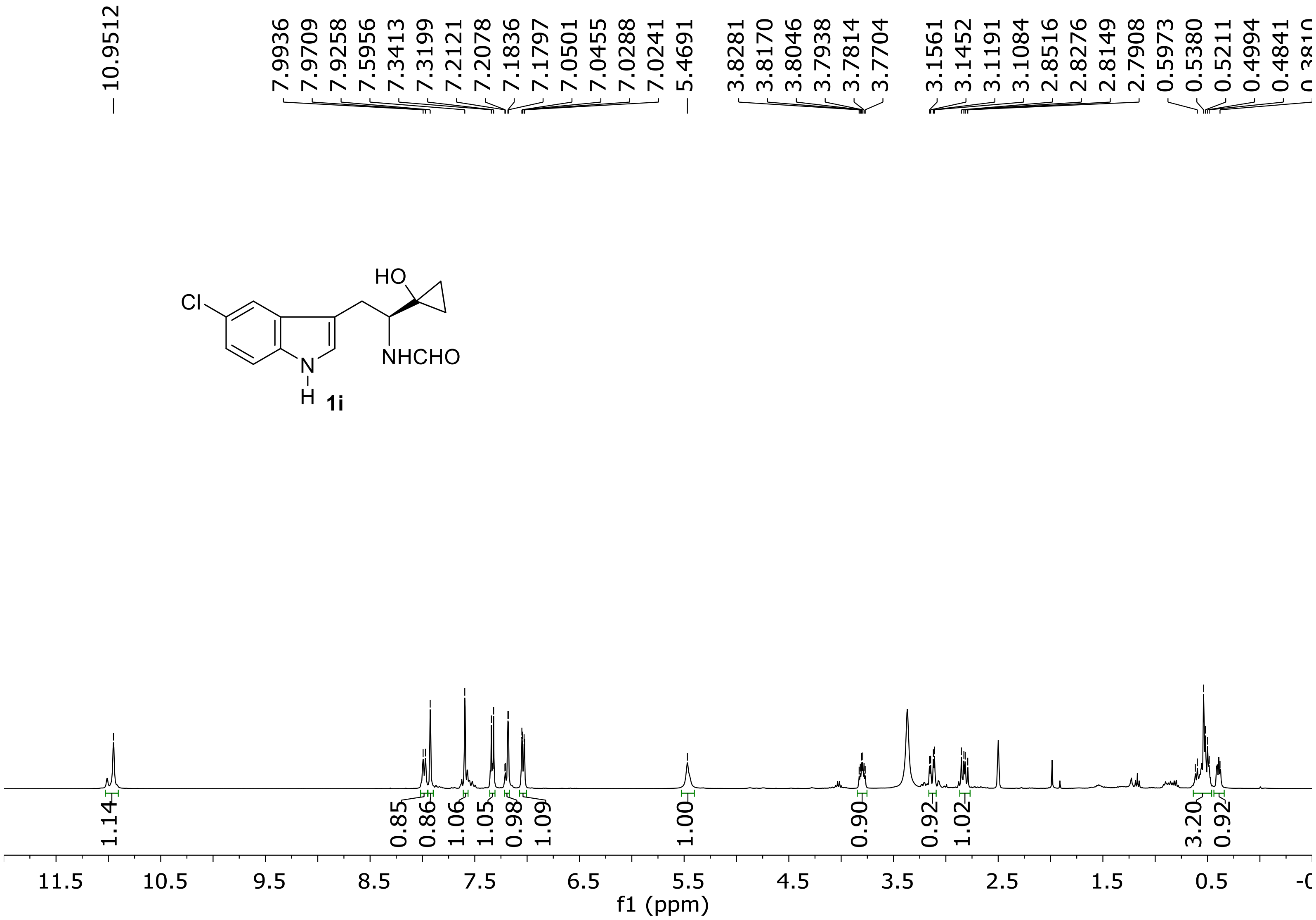
10.9512
7.9936
7.9709
7.9258
7.5956
7.3413
7.3199
7.2121
7.2078
7.1836
7.1797
7.0501
7.0455
7.0288
7.0241
5.4691
3.8281
3.8170
3.8046
3.7938
3.7814
3.7704
3.1561
3.1452
3.1191
3.1084
2.8516
2.8276
2.8149
2.7908
0.5973
0.5380
0.5211
0.4994
0.4841
HO
Cl
N
H
NHCHO
1i
1.14
0.85
0.86
1.06
1.05
0.98
1.09
1.00
0.90
0.92
1.02
3.20
0.92
11.5
10.5
9.5
8.5
7.5
6.5
5.5
4.5
3.5
2.5
1.5
0.5
f1 (ppm)

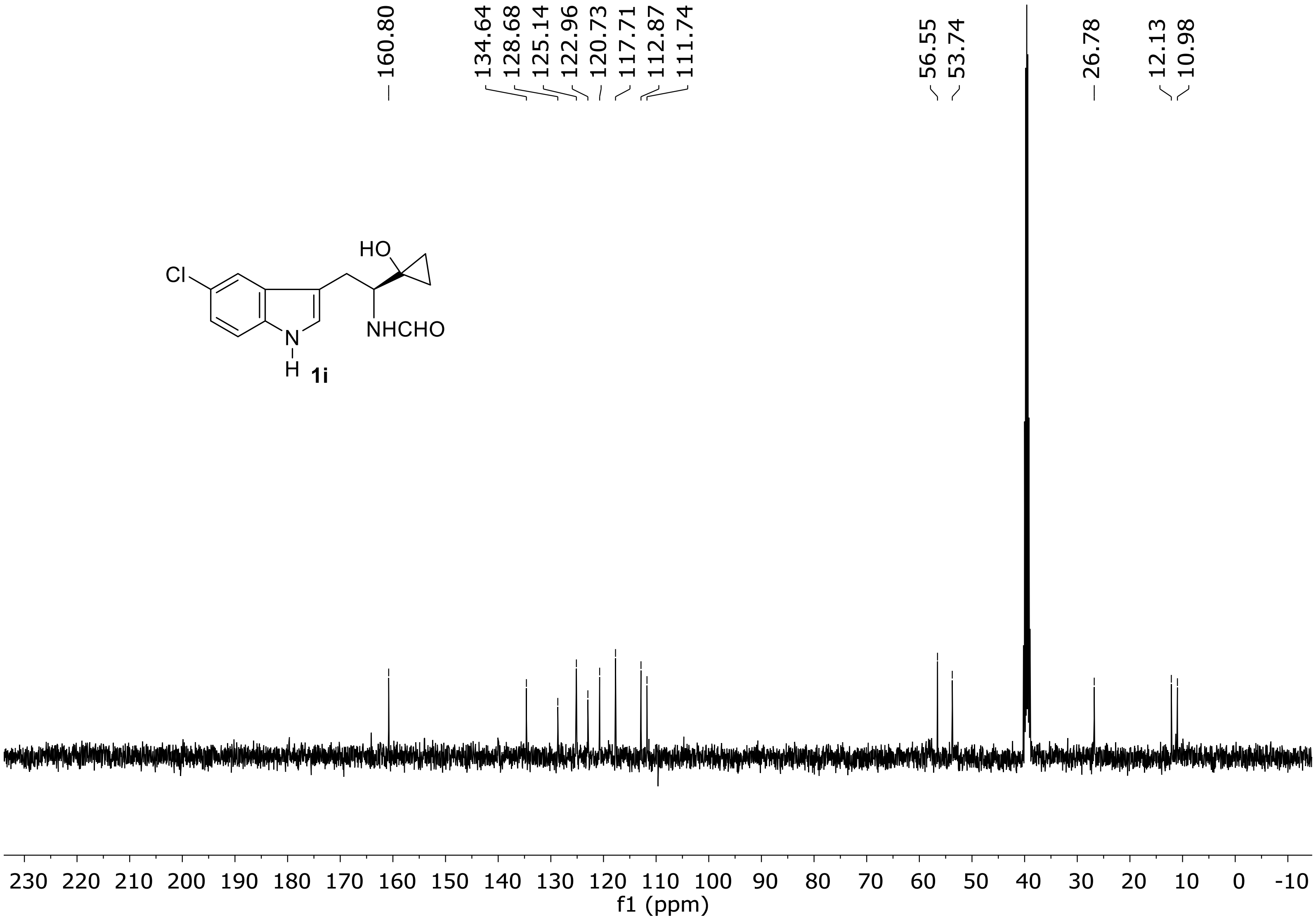
HO
Cl
N
H
NHCHO
1i
160.80
134.64
128.68
125.14
122.96
120.73
117.71
112.87
111.74
56.55
53.74
26.78
12.13
10.98
230 220 210 200 190 180 170 160 150 140 130 120 110 100 90 80 70 60 50 40 30 20 10 0 -10
f1 (ppm)

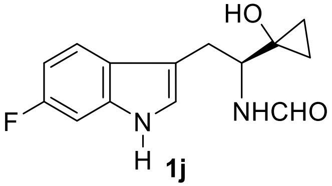
HO
NHCHO
F
N
H

1j

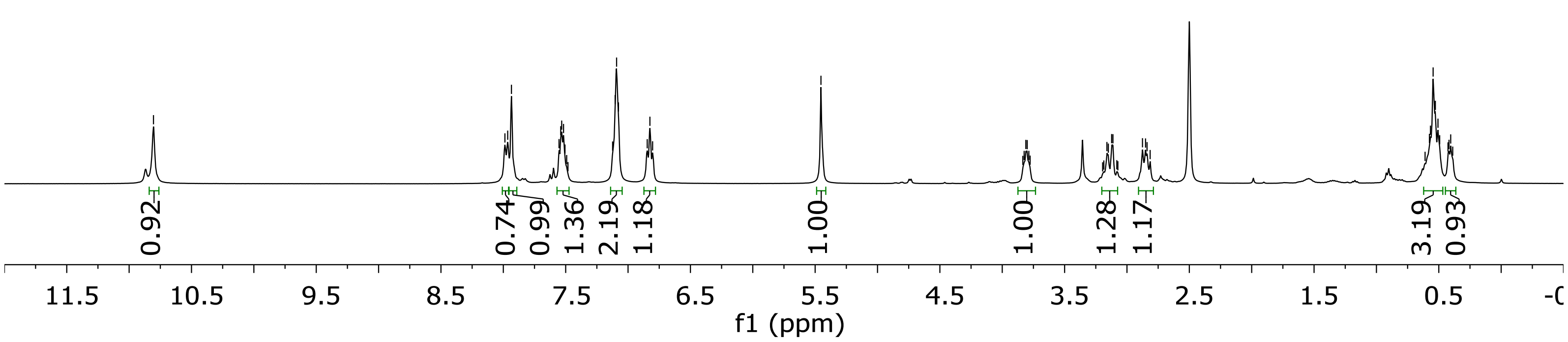
10.8049
7.9872
7.9649
7.9349
7.5534
7.5399
7.5321
7.5172
7.5056
7.4920
7.1238
7.1035
7.0920
7.0769
6.8463
6.8247
6.8032
5.4534
3.8348
3.8243
3.8110
3.8000
3.7867
3.7765
3.1856
3.1608
3.1494
3.1229
3.1124
3.0815
2.8757
2.8511
2.8392
2.8143
0.6100
0.5750
0.5668
0.5455
0.5277
0.5065
0.4930
0.4262
0.4181
0.4045
0.3883
0.92
0.74
0.99
1.36
2.19
1.18
1.00
1.00
1.28
1.17
3.19
0.93
11.5
10.5
9.5
8.5
7.5
6.5
5.5
4.5
3.5
2.5
1.5
0.5
f1 (ppm)

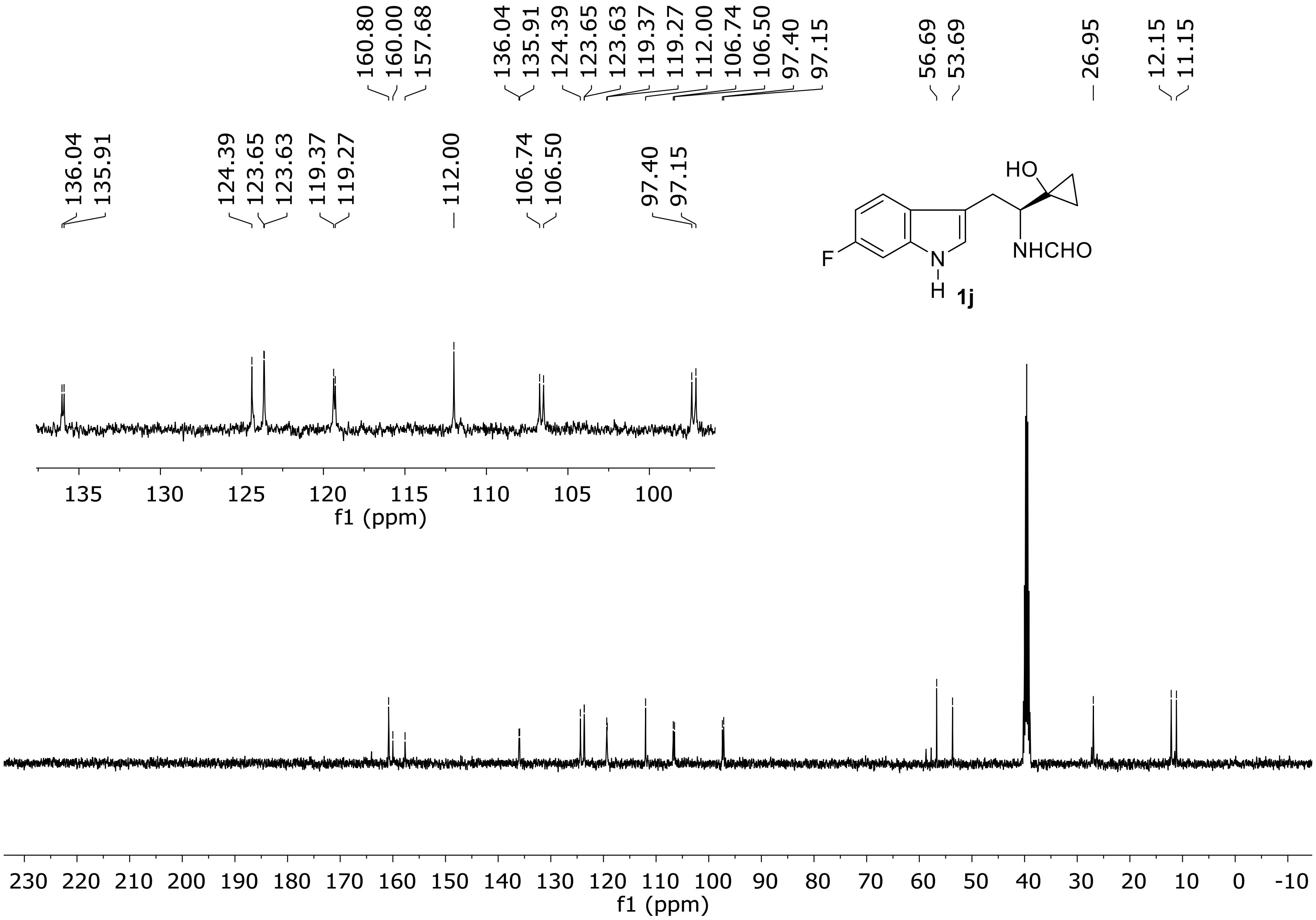
160.80
160.00
157.68
136.04
135.91
124.39
123.65
123.63
119.37
119.27
112.00
106.74
106.50
97.40
97.15
56.69
53.69
26.95
12.15
11.15
HO
F
N
H
NHCHO
1j
135 130 125 120 115 110 105 100
f1 (ppm)
230 220 210 200 190 180 170 160 150 140 130 120 110 100 90 80 70 60 50 40 30 20 10 0 -10
f1 (ppm)

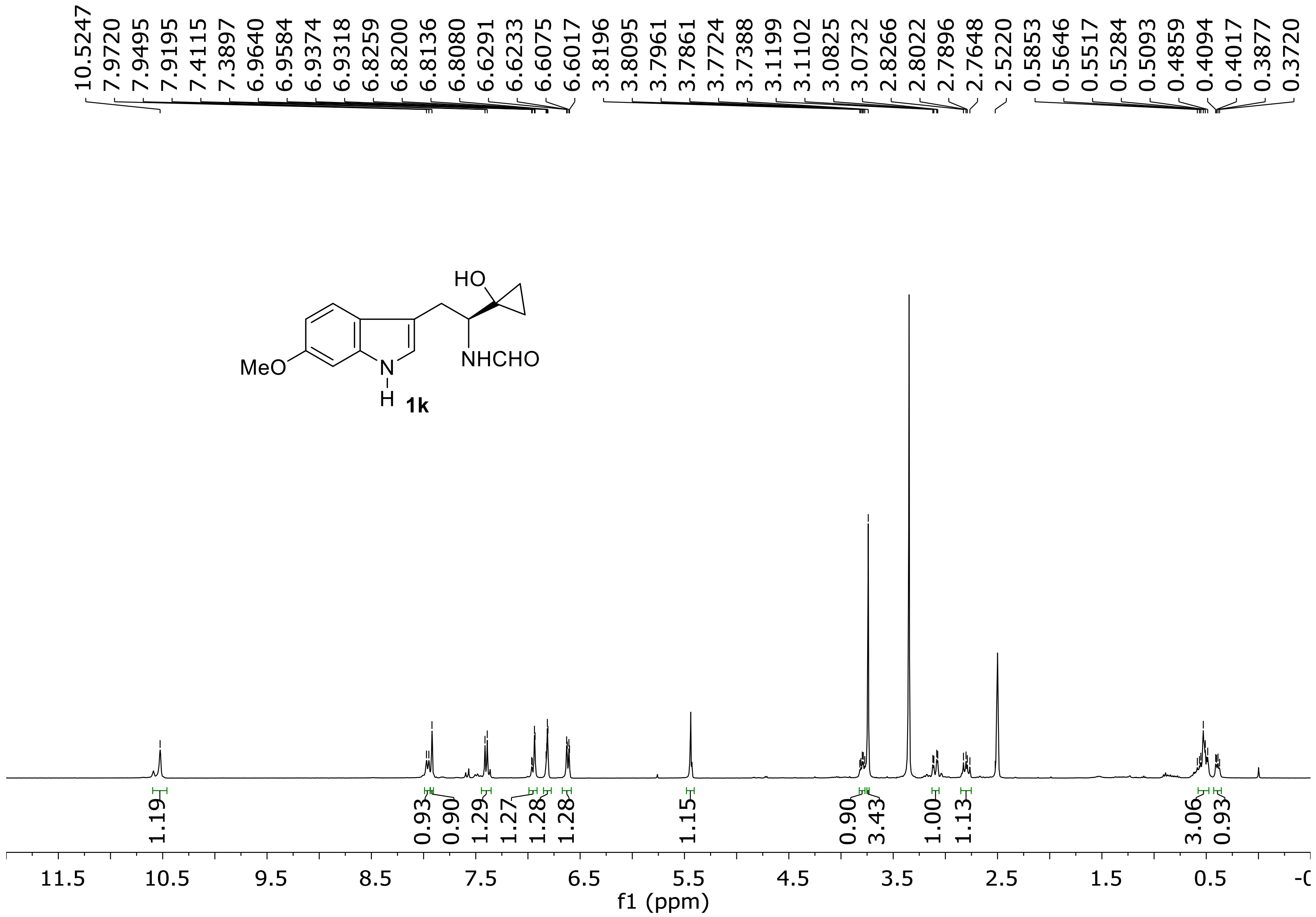
10.5247
7.9720
7.9495
7.9195
7.4115
7.3897
6.9640
6.9584
6.9374
6.9318
6.8259
6.8200
6.8136
6.8080
6.6291
6.6233
6.6075
6.6017
3.8196
3.8095
3.7961
3.7861
3.7724
3.7388
3.1199
3.1102
3.0825
3.0732
2.8266
2.8022
2.7896
2.7648
2.5220
0.5853
0.5646
0.5517
0.5284
0.5093
0.4859
0.4094
0.4017
0.3877
0.3720
HO
MeO
N
H
NHCHO
1k
1.19
0.93
0.90
1.29
1.27
1.28
1.28
1.15
0.90
3.43
1.00
1.13
3.06
0.93
11.5
10.5
9.5
8.5
7.5
6.5
5.5
4.5
3.5
2.5
1.5
0.5
f1 (ppm)

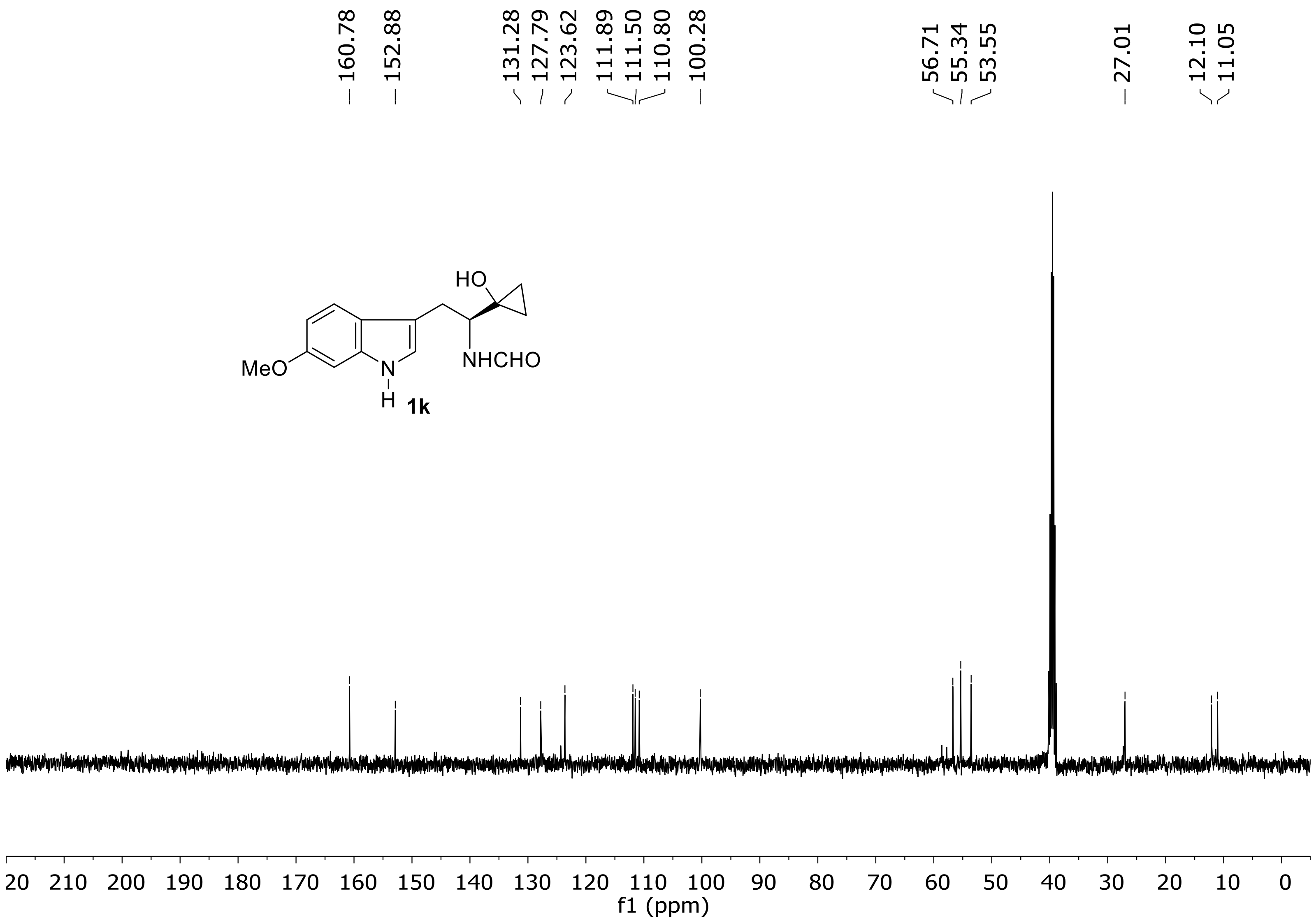

160.78
152.88
131.28
127.79
123.62
111.89
111.50
110.80
100.28
56.71
55.34
53.55
27.01
12.10
11.05
HO
MeO
N
H
1k
NHCHO
20 210 200 190 180 170 160 150 140 130 120 110 100 90 80 70 60 50 40 30 20 10 0
f1 (ppm)

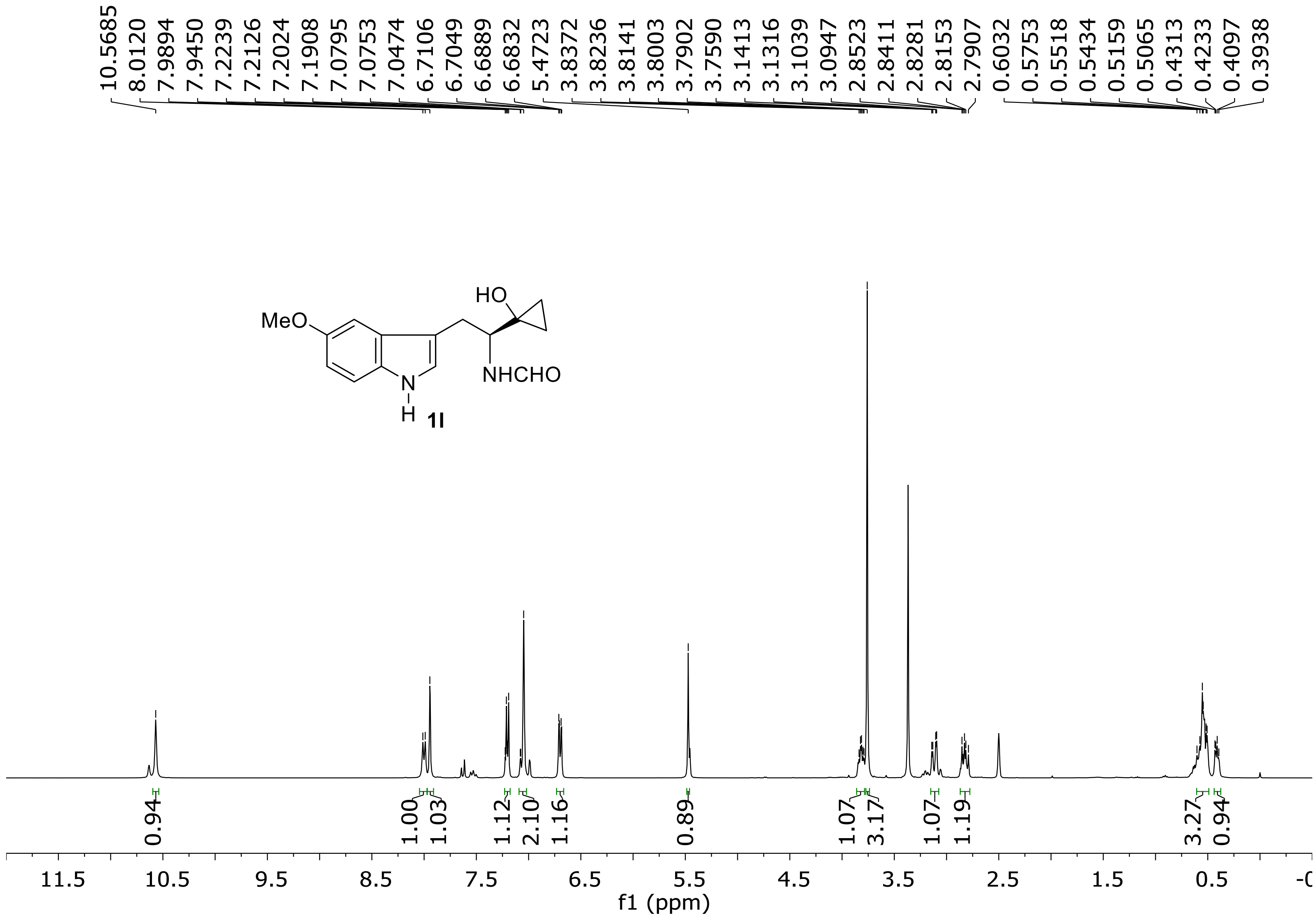
10.5685
8.0120
7.9894
7.9450
7.2239
7.2126
7.2024
7.1908
7.0795
7.0753
7.0474
6.7106
6.7049
6.6889
6.6832
5.4723
3.8372
3.8236
3.8141
3.8003
3.7902
3.7590
3.1413
3.1316
3.1039
3.0947
2.8523
2.8411
2.8281
2.8153
2.7907
0.6032
0.5753
0.5518
0.5434
0.5159
0.5065
0.4313
0.4233
0.4097
0.3938
HO
MeO
NHCHO
N
H
1l
0.94
1.00
1.03
1.12
2.10
1.16
0.89
1.07
3.17
1.07
1.19
3.27
0.94
11.5
10.5
9.5
8.5
7.5
6.5
5.5
4.5
3.5
2.5
1.5
0.5
f1 (ppm)

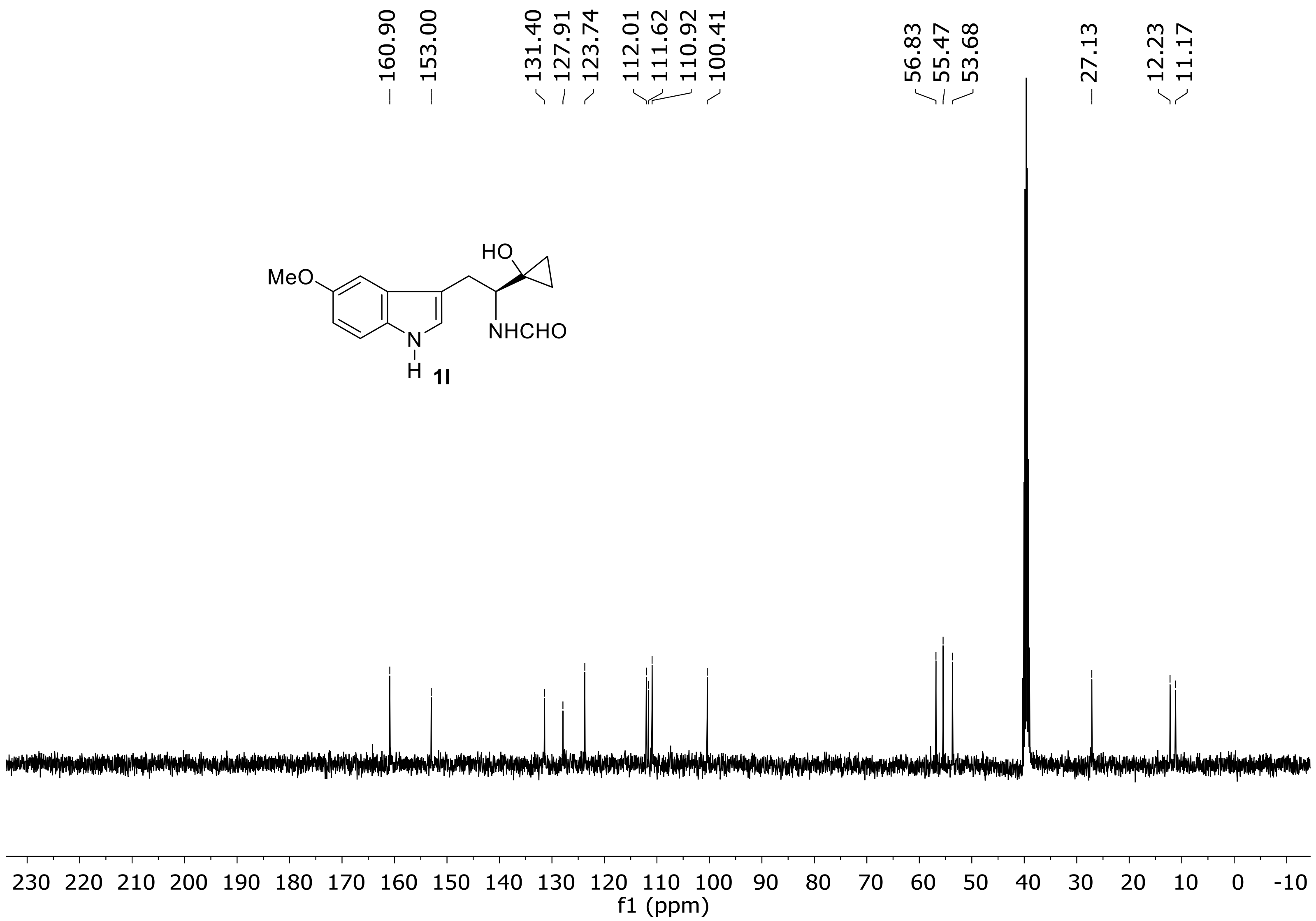
160.90
153.00
131.40
127.91
123.74
112.01
111.62
110.92
100.41
56.83
55.47
53.68
27.13
12.23
11.17
MeO
HO
NHCHO
N
H
1l
230 220 210 200 190 180 170 160 150 140 130 120 110 100 90 80 70 60 50 40 30 20 10 0 -10
f1 (ppm)

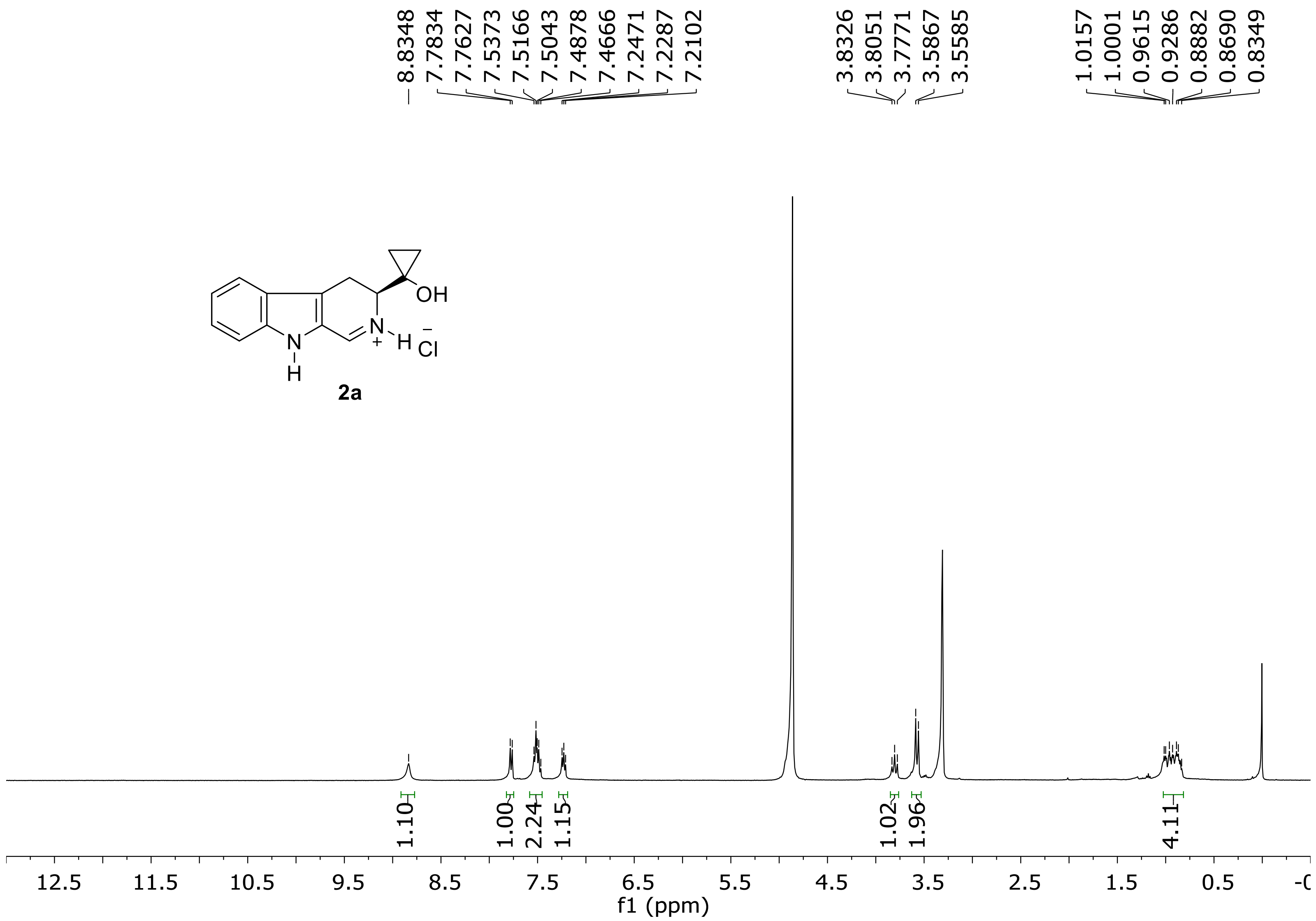
8.8348
7.7834
7.7627
7.5373
7.5166
7.5043
7.4878
7.4666
7.2471
7.2287
7.2102
3.8326
3.8051
3.7771
3.5867
3.5585
1.0157
1.0001
0.9615
0.9286
0.8882
0.8690
0.8349
OH
N
H
Cl
2a
1.10
1.00
2.24
1.15
1.02
1.96
4.11
12.5
11.5
10.5
9.5
8.5
7.5
6.5
5.5
4.5
3.5
2.5
1.5
0.5
f1 (ppm)

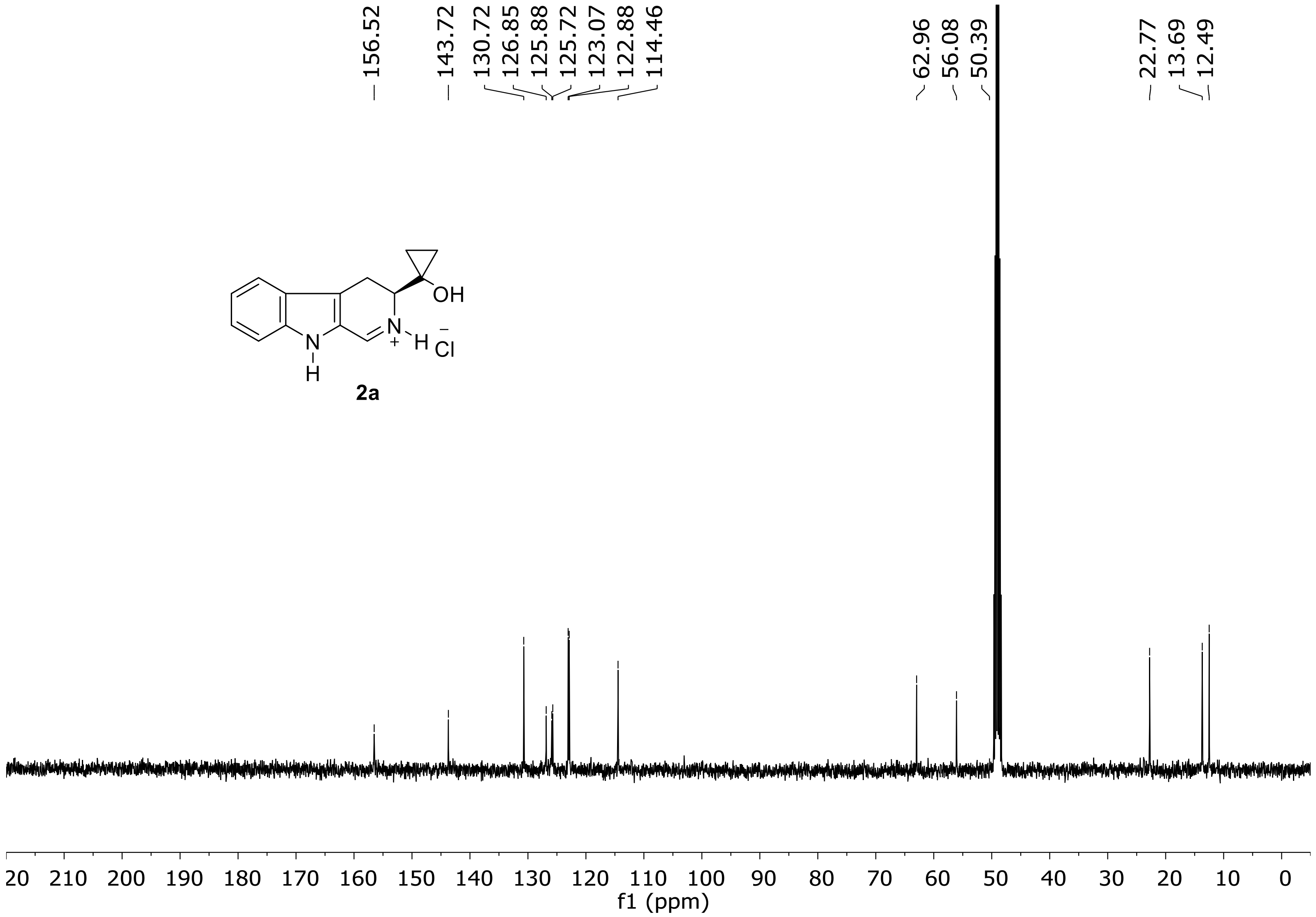
156.52
143.72
130.72
126.85
125.88
125.72
123.07
122.88
114.46
62.96
56.08
50.39
22.77
13.69
12.49
OH
N
H
Cl
2a
20
210
200
190
180
170
160
150
140
130
120
110
100
90
80
70
60
50
40
30
20
10
0
f1 (ppm)

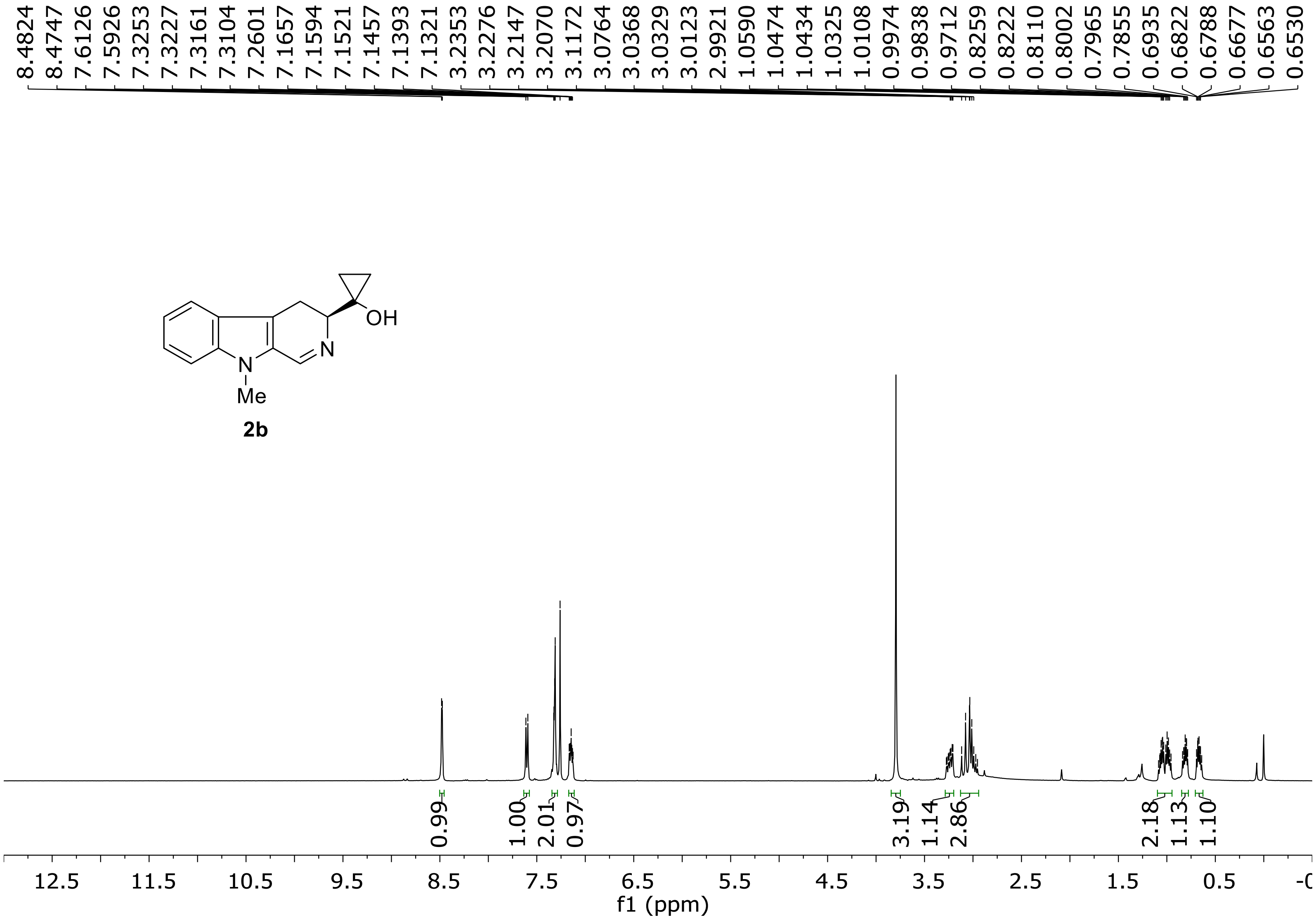
8.4824
8.4747
7.6126
7.5926
7.3253
7.3227
7.3161
7.3104
7.2601
7.1657
7.1594
7.1521
7.1457
7.1393
7.1321
3.2353
3.2276
3.2147
3.2070
3.1172
3.0764
3.0368
3.0329
3.0123
2.9921
1.0590
1.0474
1.0434
1.0325
1.0108
0.9974
0.9838
0.9712
0.8259
0.8222
0.8110
0.8002
0.7965
0.7855
0.6935
0.6822
0.6788
0.6677
0.6563
0.6530
OH
N
N
Me
2b
0.99
1.00
2.01
0.97
3.19
1.14
2.86
2.18
1.13
1.10
12.5
11.5
10.5
9.5
8.5
7.5
6.5
5.5
4.5
3.5
2.5
1.5
0.5
f1 (ppm)

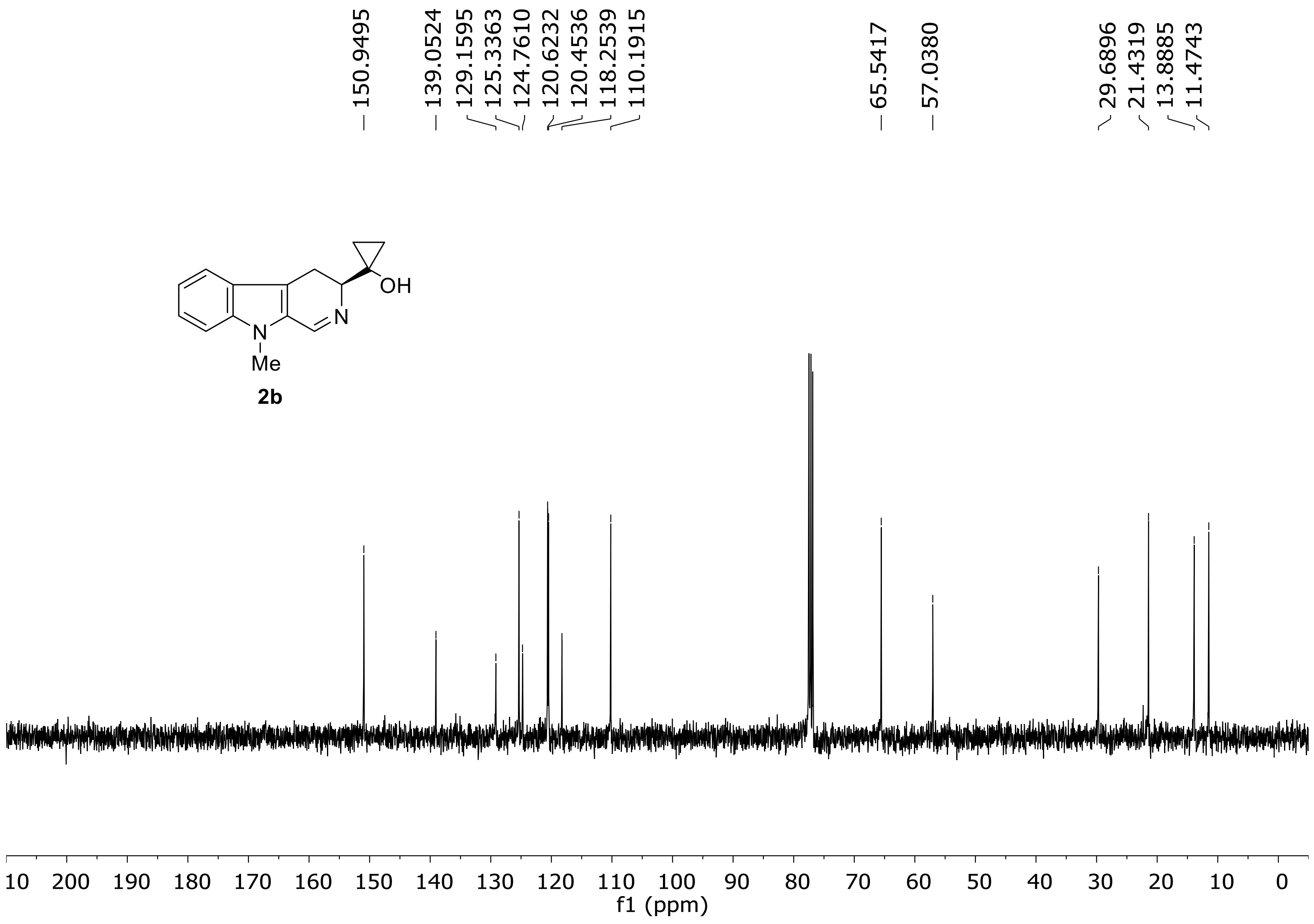

150.9495
139.0524
129.1595
125.3363
124.7610
120.6232
120.4536
118.2539
110.1915
65.5417
57.0380
29.6896
21.4319
13.8885
11.4743
OH
N
N
Me
2b
10 200 190 180 170 160 150 140 130 120 110 100 90 80 70 60 50 40 30 20 10 0
f1 (ppm)

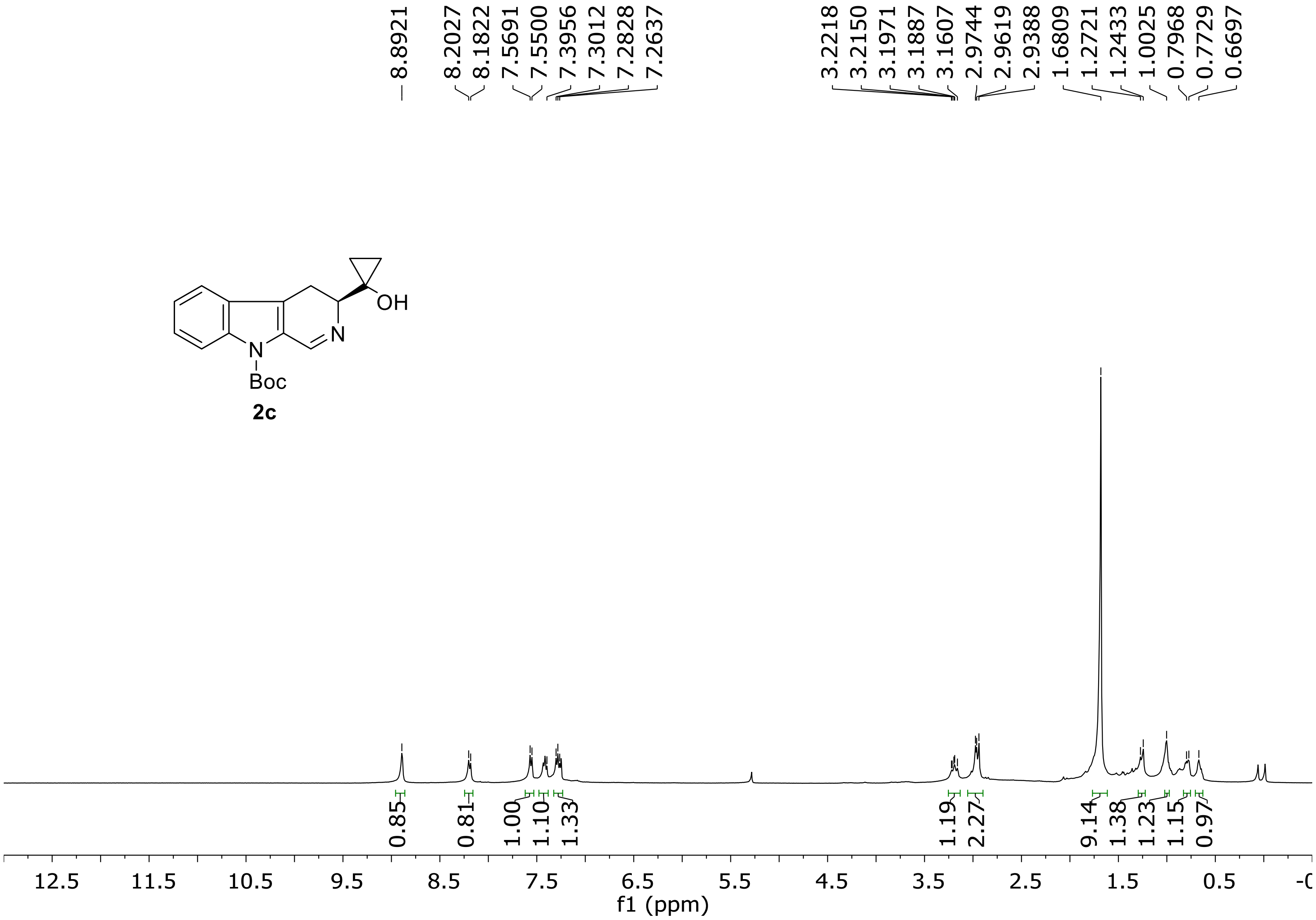
8.8921
8.2027
8.1822
7.5691
7.5500
7.3956
7.3012
7.2828
7.2637
3.2218
3.2150
3.1971
3.1887
3.1607
2.9744
2.9619
2.9388
1.6809
1.2721
1.2433
1.0025
0.7968
0.7729
0.6697
OH
N
N
Boc
2c
0.85
0.81
1.00
1.10
1.33
1.19
2.27
9.14
1.38
1.23
1.15
0.97
12.5
11.5
10.5
9.5
8.5
7.5
6.5
5.5
4.5
3.5
2.5
1.5
0.5
f1 (ppm)

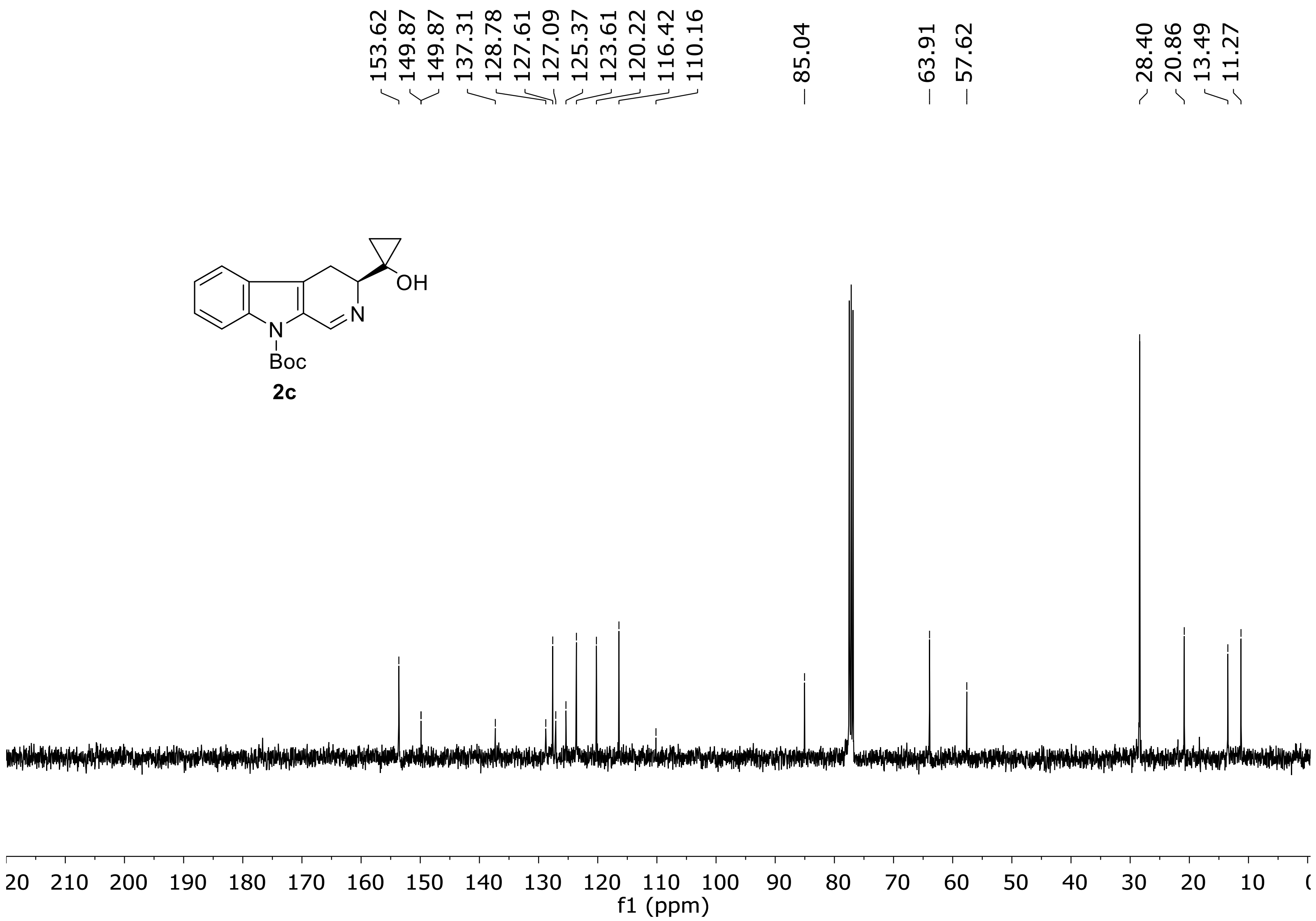
153.62
149.87
149.87
137.31
128.78
127.61
127.09
125.37
123.61
120.22
116.42
110.16
85.04
63.91
57.62
28.40
20.86
13.49
11.27
OH
N
N
Boc
2c
20 210 200 190 180 170 160 150 140 130 120 110 100 90 80 70 60 50 40 30 20 10
f1 (ppm)

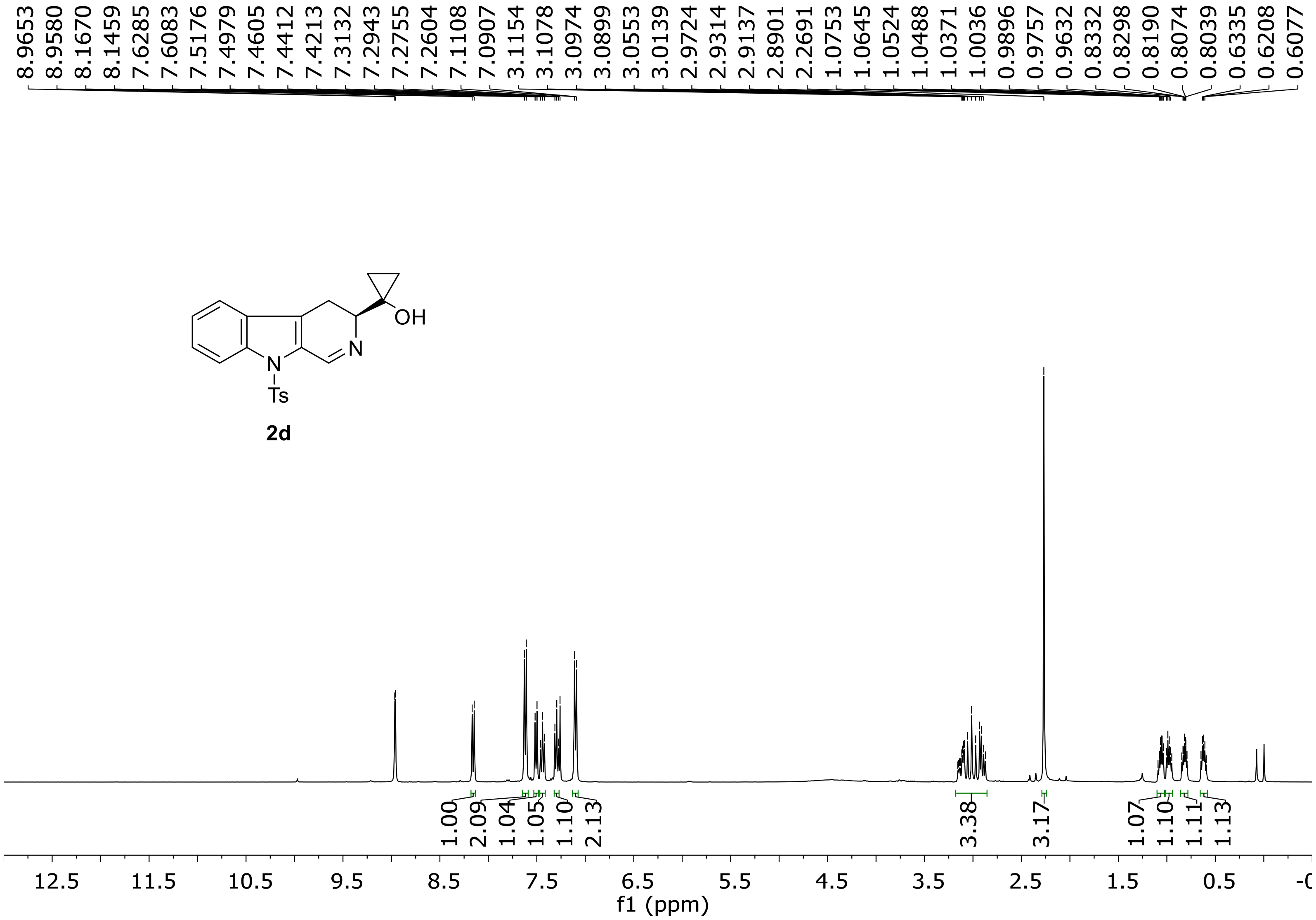
8.9653
8.9580
8.1670
8.1459
7.6285
7.6083
7.5176
7.4979
7.4605
7.4412
7.4213
7.3132
7.2943
7.2755
7.2604
7.1108
7.0907
3.1154
3.1078
3.0974
3.0899
3.0553
3.0139
2.9724
2.9314
2.9137
2.8901
2.2691
1.0753
1.0645
1.0524
1.0488
1.0371
1.0036
0.9896
0.9757
0.9632
0.8332
0.8298
0.8190
0.8074
0.8039
0.6335
0.6208
0.6077
OH
N
N
Ts
2d
1.00
2.09
1.04
1.05
1.10
2.13
3.38
3.17
1.07
1.10
1.11
1.13
12.5
11.5
10.5
9.5
8.5
7.5
6.5
5.5
4.5
3.5
2.5
1.5
0.5
f1 (ppm)

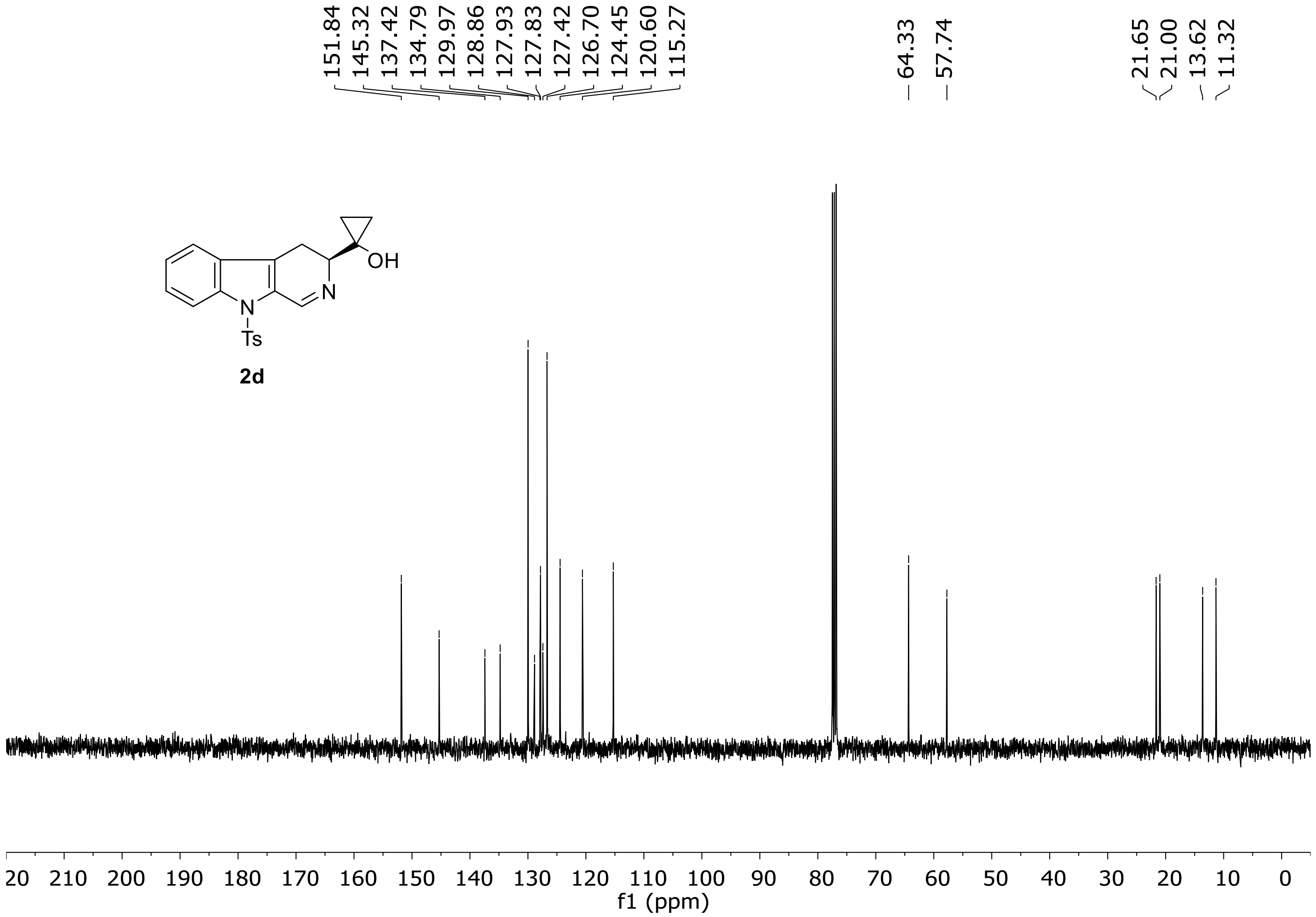
151.84
145.32
137.42
134.79
129.97
128.86
127.93
127.83
127.42
126.70
124.45
120.60
115.27
64.33
57.74
21.65
21.00
13.62
11.32
OH
N
N
Ts
20 210 200 190 180 170 160 150 140 130 120 110 100 90 80 70 60 50 40 30 20 10 0
f1 (ppm)

2d

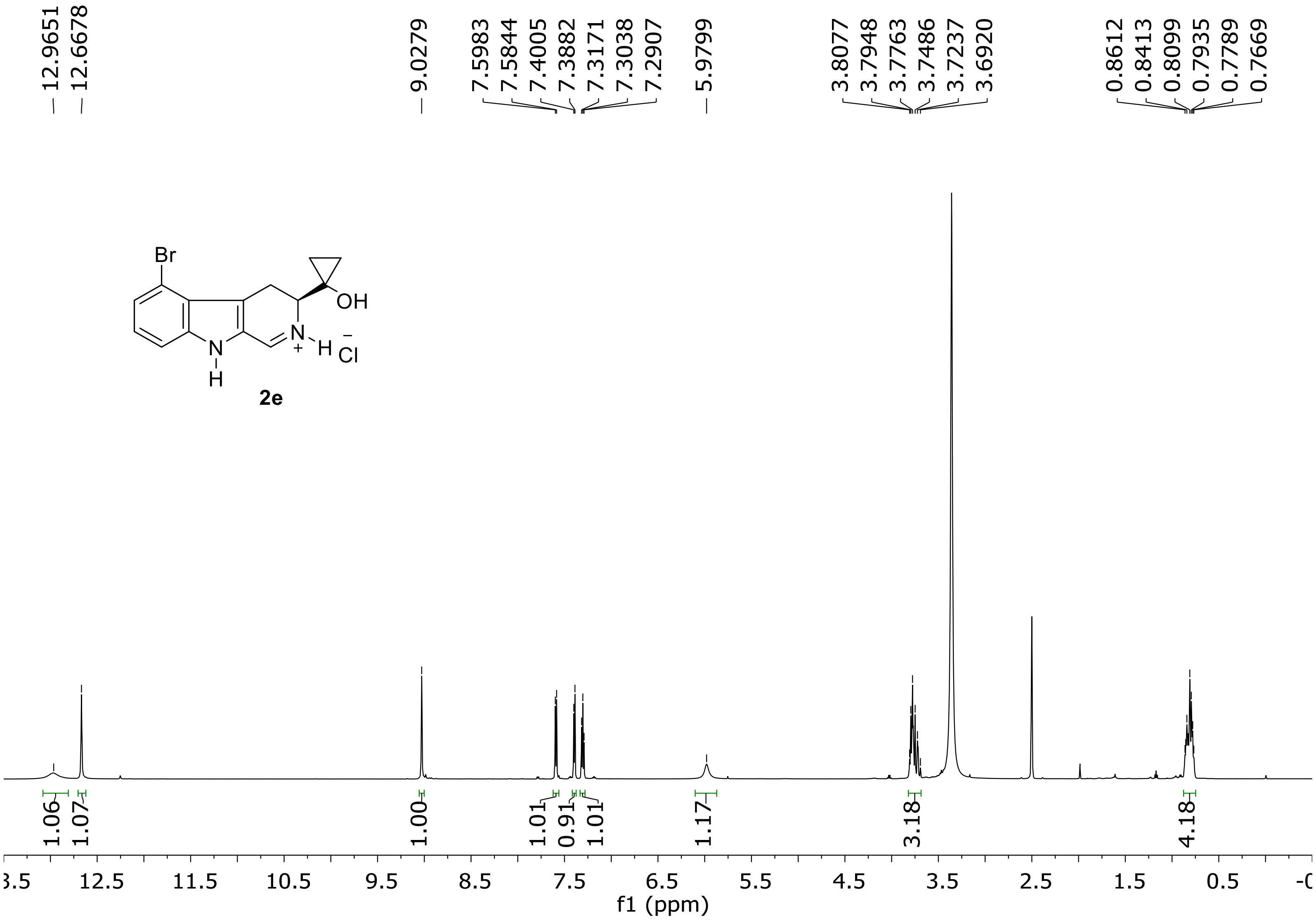

Br
OH
N
H
N
+
H
Cl
–
2e
12.9651
12.6678
9.0279
7.5983
7.5844
7.4005
7.3882
7.3171
7.3038
7.2907
5.9799
3.8077
3.7948
3.7763
3.7486
3.7237
3.6920
0.8612
0.8413
0.8099
0.7935
0.7789
0.7669
1.06
1.07
1.00
1.01
0.91
1.01
1.17
3.18
4.18
12.5
11.5
10.5
9.5
8.5
7.5
6.5
5.5
4.5
3.5
2.5
1.5
0.5
f1 (ppm)

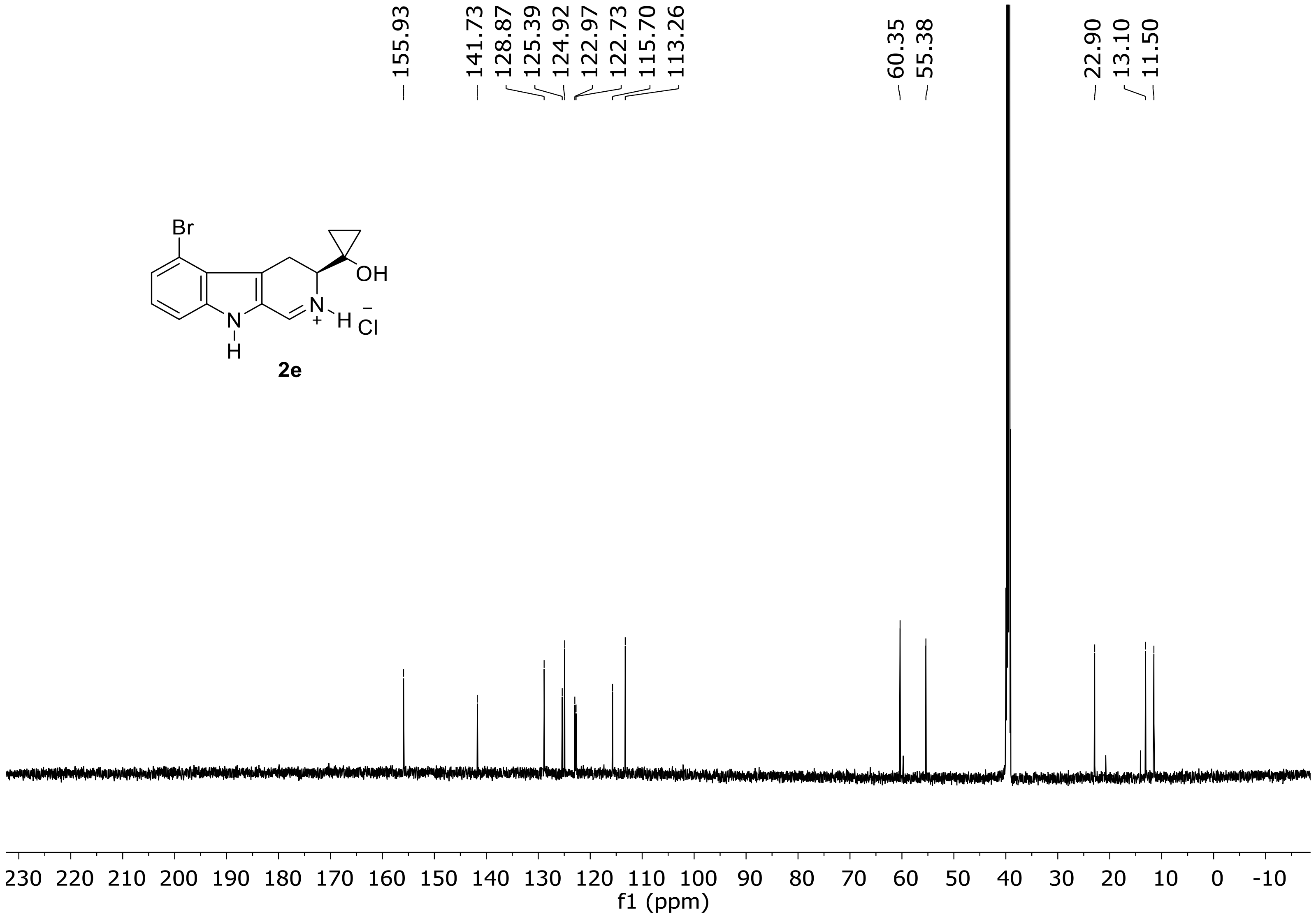

155.93
141.73
128.87
125.39
124.92
122.97
122.73
115.70
113.26
60.35
55.38
22.90
13.10
11.50
Br
OH
N
H
N
+
H
Cl
230 220 210 200 190 180 170 160 150 140 130 120 110 100 90 80 70 60 50 40 30 20 10 0 -10
f1 (ppm)

2e

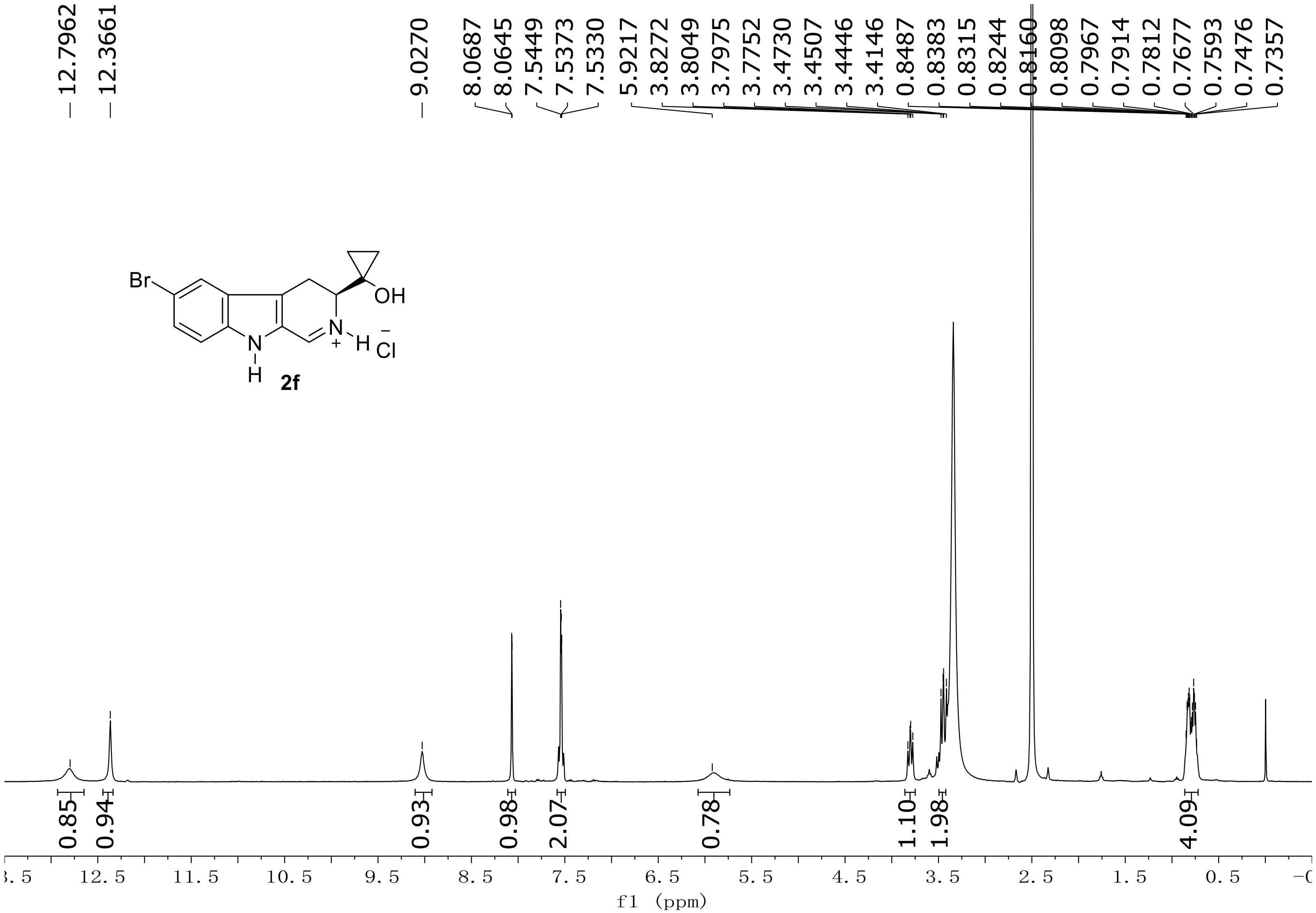
12.7962
12.3661
9.0270
8.0687
8.0645
7.5449
7.5373
7.5330
5.9217
3.8272
3.8049
3.7975
3.7752
3.4730
3.4507
3.4446
3.4146
0.8487
0.8383
0.8315
0.8244
0.8160
0.8098
0.7967
0.7914
0.7812
0.7677
0.7593
0.7476
0.7357
Br
OH
N
H
Cl
2f
0.85
0.94
0.93
0.98
2.07
0.78
1.10
1.98
4.09
12.5
11.5
10.5
9.5
8.5
7.5
6.5
5.5
4.5
3.5
2.5
1.5
0.5
f1 (ppm)

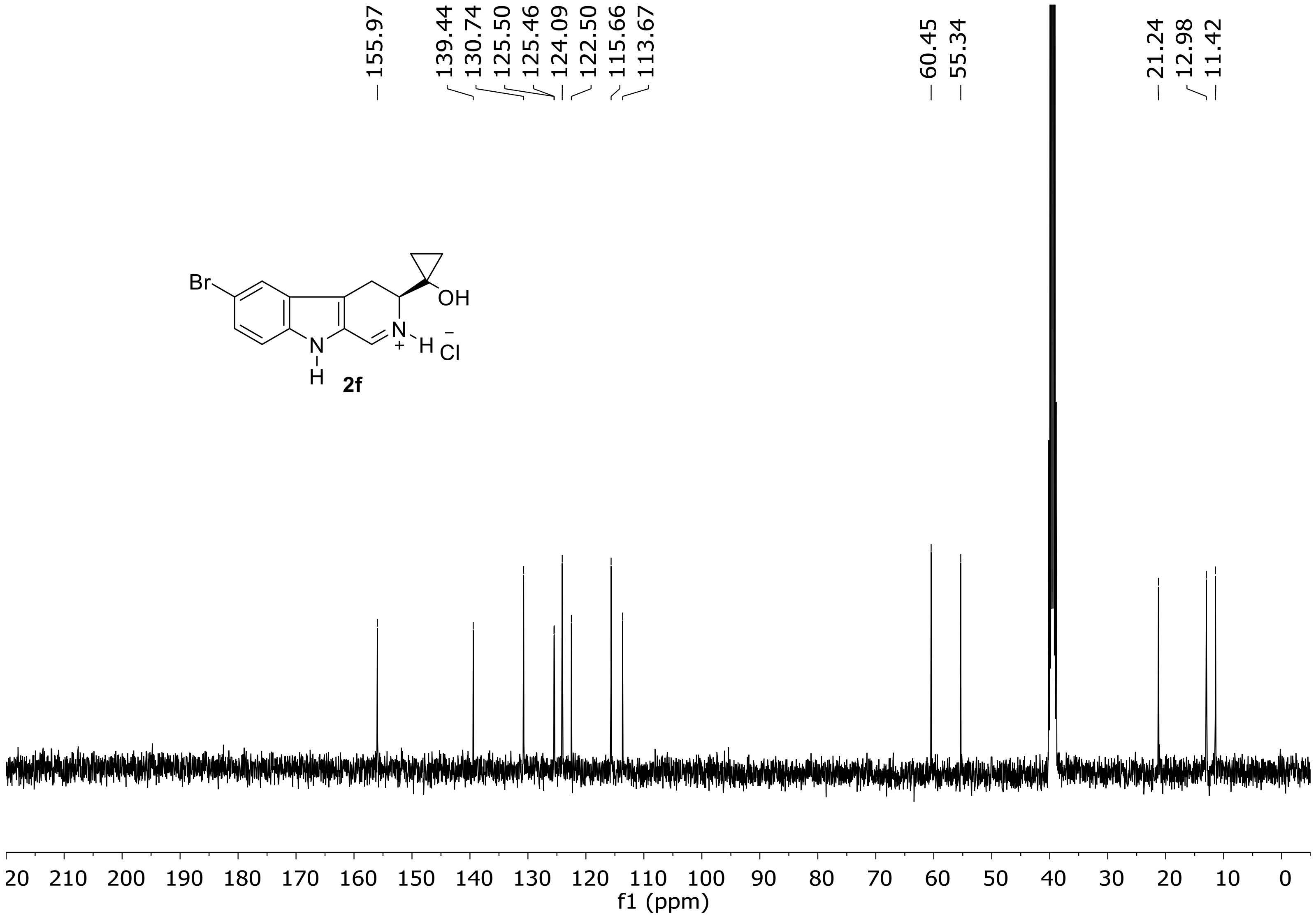
155.97
139.44
130.74
125.50
125.46
124.09
122.50
115.66
113.67
60.45
55.34
21.24
12.98
11.42
Br
OH
N
H
N
+
H
–
Cl
2f
20 210 200 190 180 170 160 150 140 130 120 110 100 90 80 70 60 50 40 30 20 10 0
f1 (ppm)

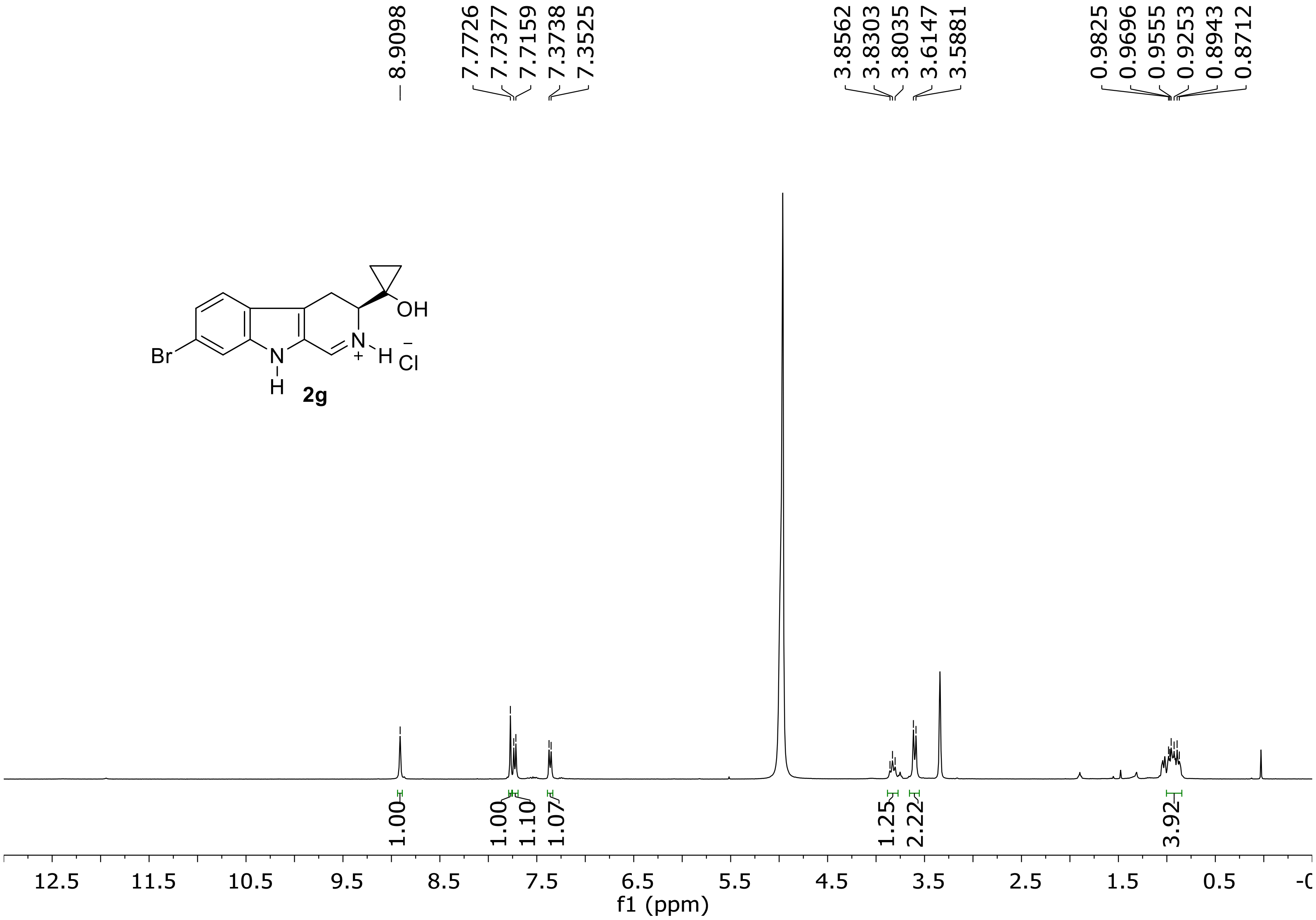
Br
N
H
N
+
H
OH
Cl
2g
8.9098
7.7726
7.7377
7.7159
7.3738
7.3525
3.8562
3.8303
3.8035
3.6147
3.5881
0.9825
0.9696
0.9555
0.9253
0.8943
0.8712
1.00
1.00
1.10
1.07
1.25
2.22
3.92
12.5
11.5
10.5
9.5
8.5
7.5
6.5
5.5
4.5
3.5
2.5
1.5
0.5
f1 (ppm)

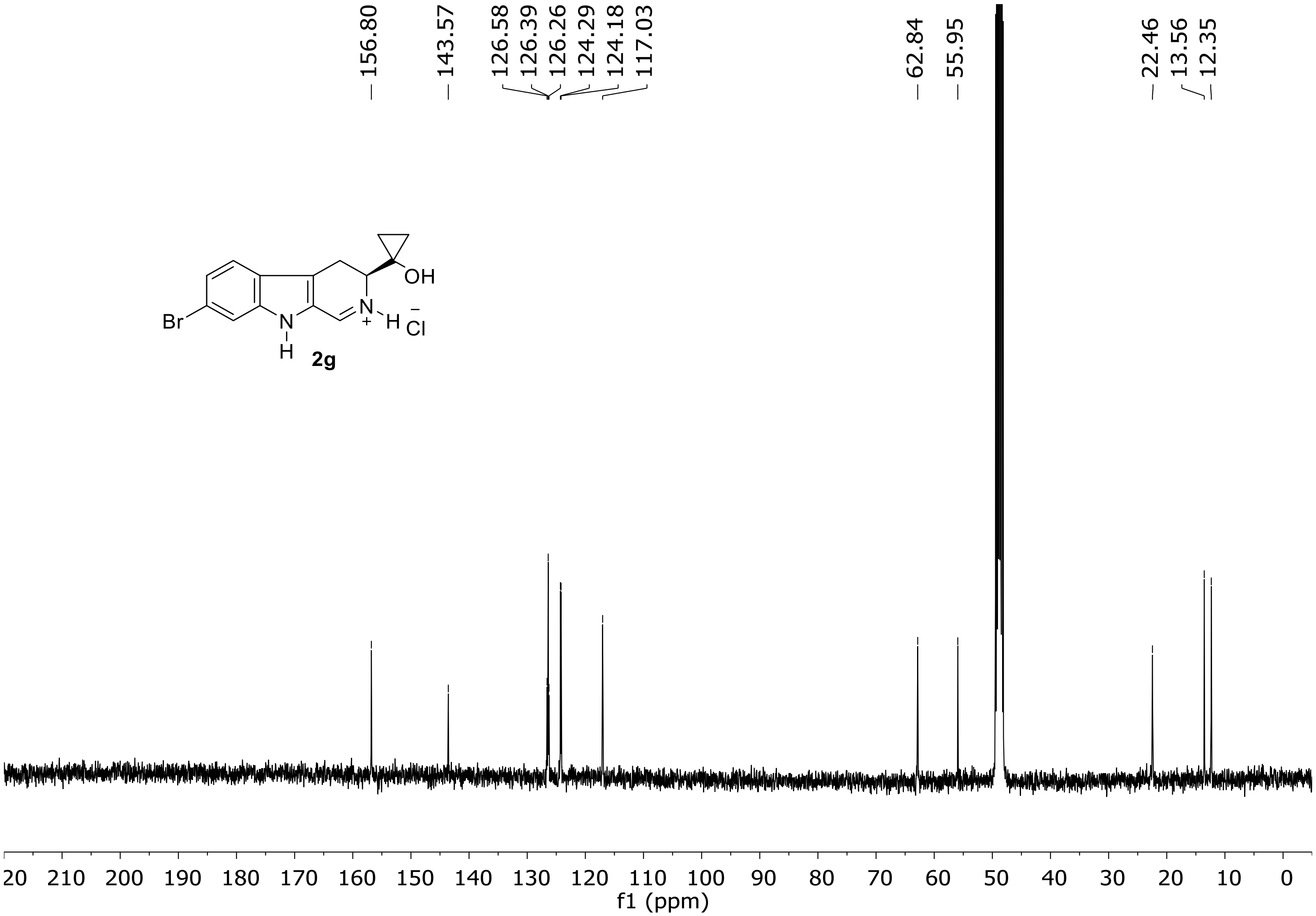
156.80
143.57
126.58
126.39
126.26
124.29
124.18
117.03
62.84
55.95
22.46
13.56
12.35
OH
Br
N
H
N
+
H
–
Cl
2g
20 210 200 190 180 170 160 150 140 130 120 110 100 90 80 70 60 50 40 30 20 10 0
f1 (ppm)

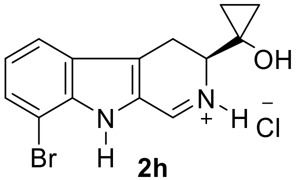
OH
N
H
Cl
Br
N
H
+
–

2h

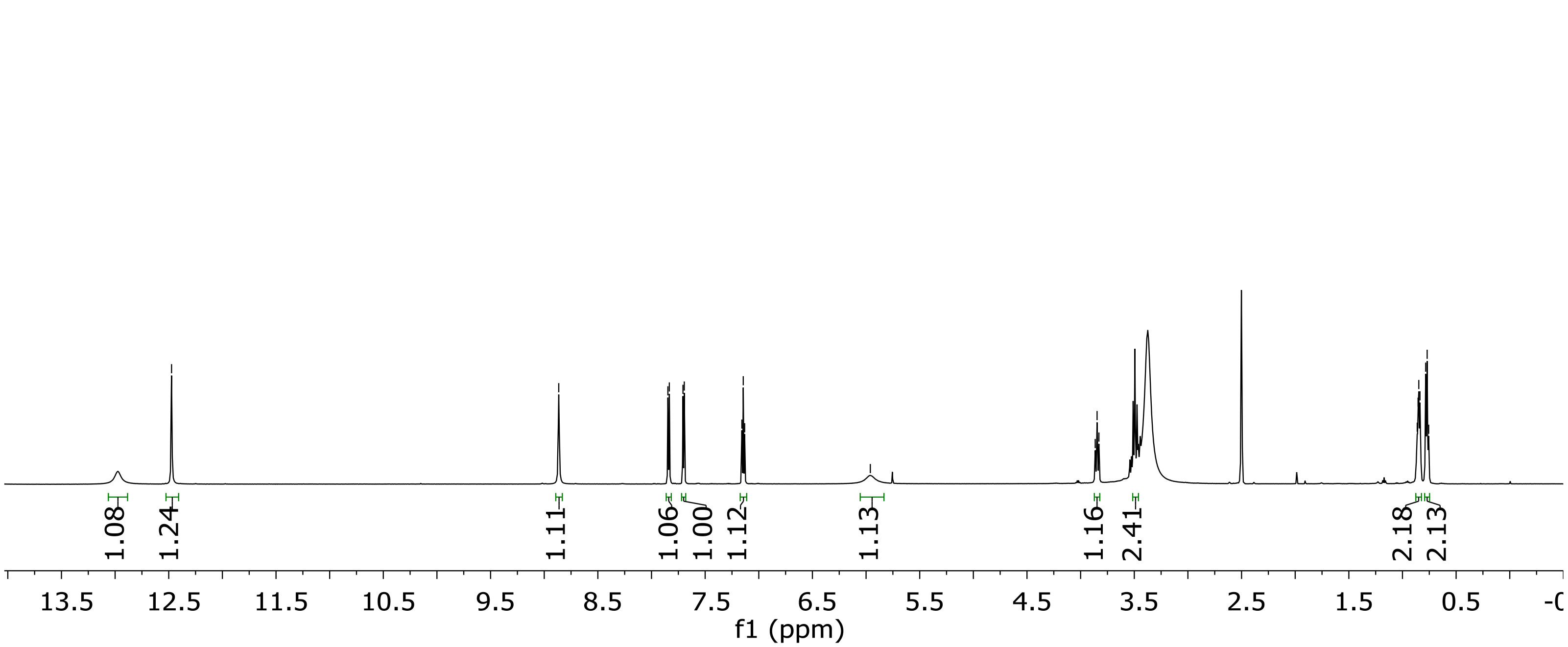
12.4737
8.8644
7.8472
7.8337
7.7069
7.6945
7.1577
7.1446
7.1317
5.9603
3.8640
3.8467
3.8296
0.8626
0.8548
0.8479
0.8371
0.7838
0.7699
0.7556
1.08
1.24
1.11
1.06
1.00
1.12
1.13
1.16
2.41
2.18
2.13
13.5
12.5
11.5
10.5
9.5
8.5
7.5
6.5
5.5
4.5
3.5
2.5
1.5
0.5
f1 (ppm)

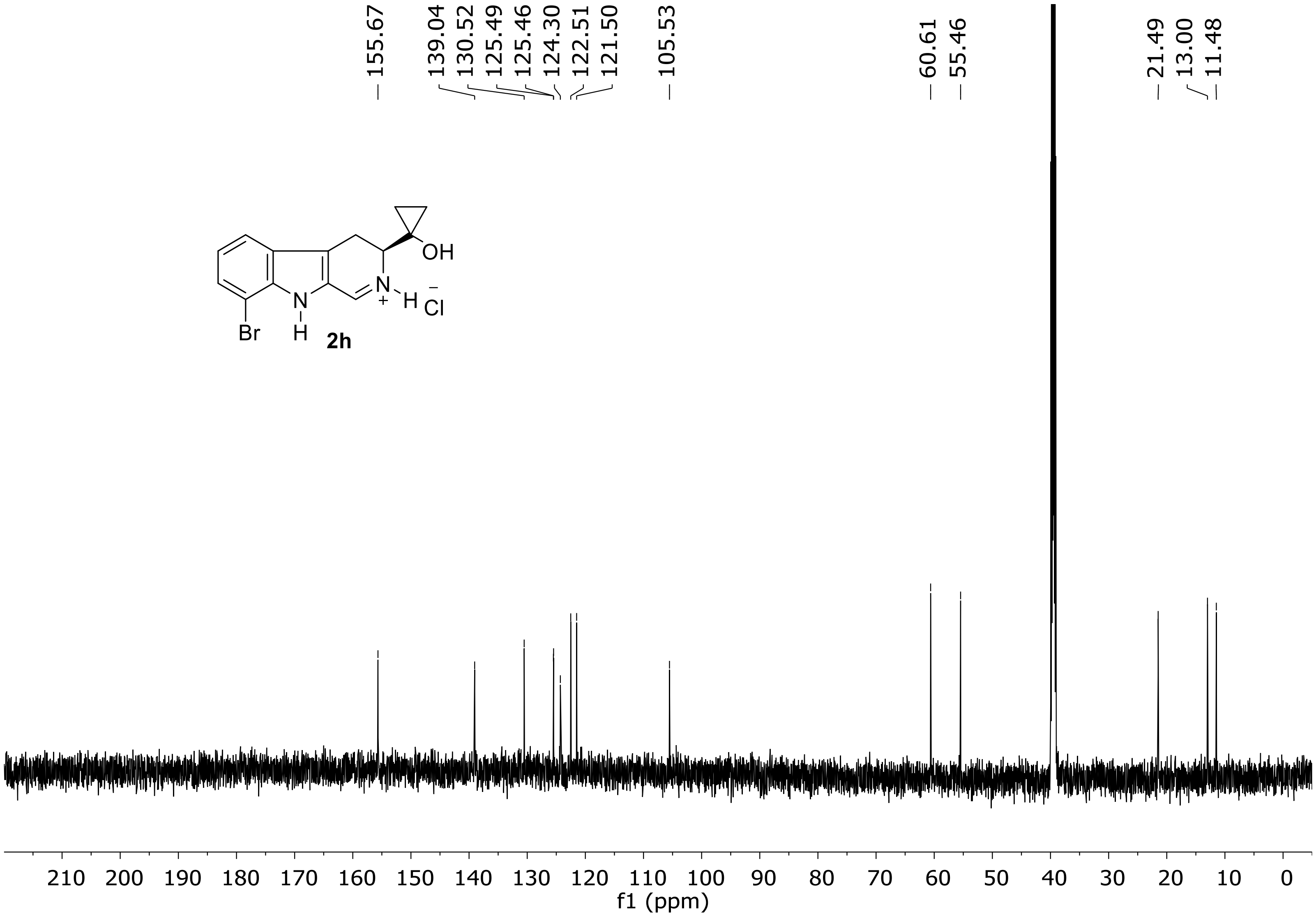
155.67
139.04
130.52
125.49
125.46
124.30
122.51
121.50
105.53
60.61
55.46
21.49
13.00
11.48
OH
N
H
Cl
Br
2h
210 200 190 180 170 160 150 140 130 120 110 100 90 80 70 60 50 40 30 20 10 0
f1 (ppm)

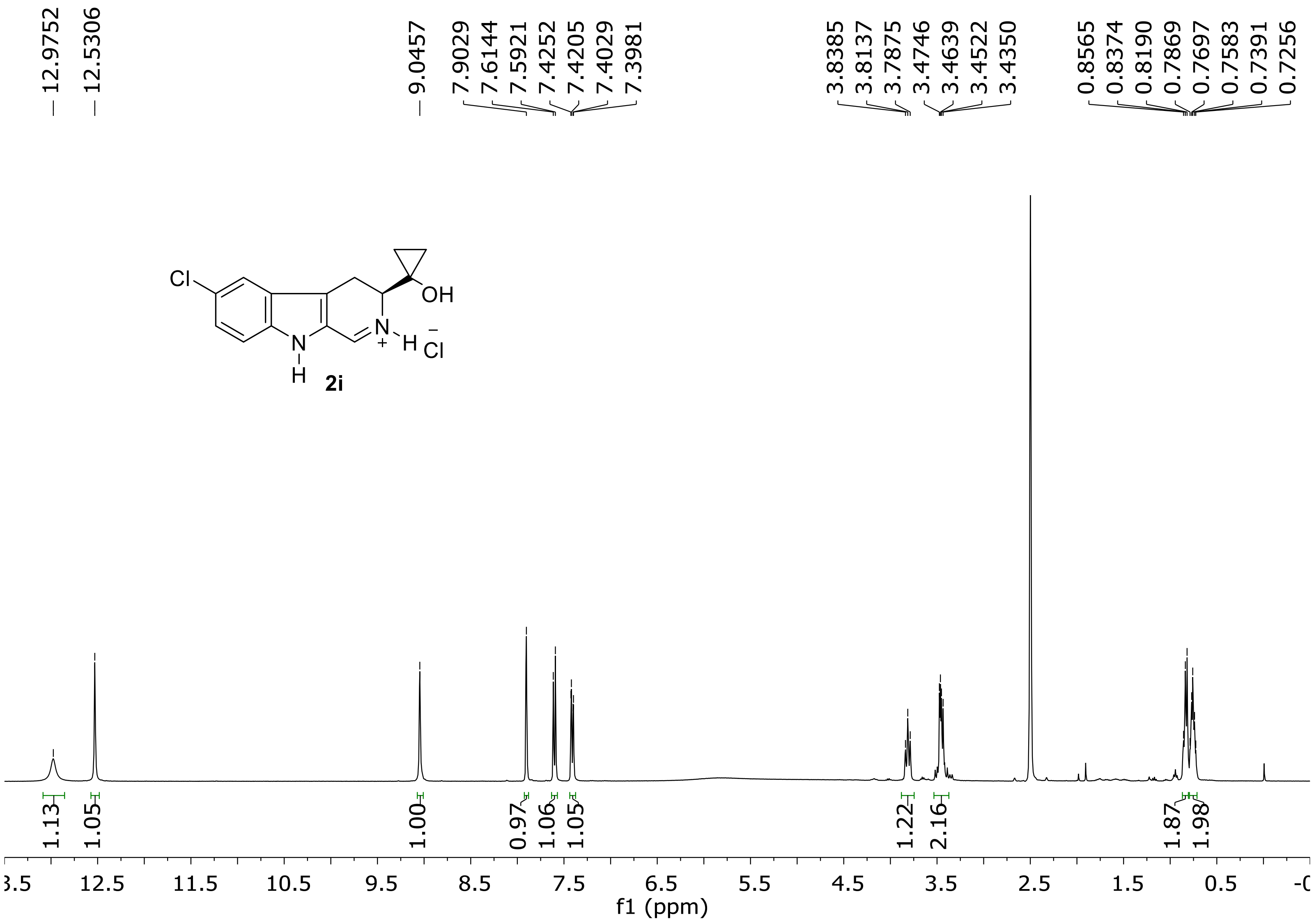
12.9752
12.5306
9.0457
7.9029
7.6144
7.5921
7.4252
7.4205
7.4029
7.3981
3.8385
3.8137
3.7875
3.4746
3.4639
3.4522
3.4350
0.8565
0.8374
0.8190
0.7869
0.7697
0.7583
0.7391
0.7256
Cl
OH
N
H
N
+
H
Cl
–
2i
1.13
1.05
1.00
0.97
1.06
1.05
1.22
2.16
1.87
1.98
12.5
11.5
10.5
9.5
8.5
7.5
6.5
5.5
4.5
3.5
2.5
1.5
0.5
f1 (ppm)

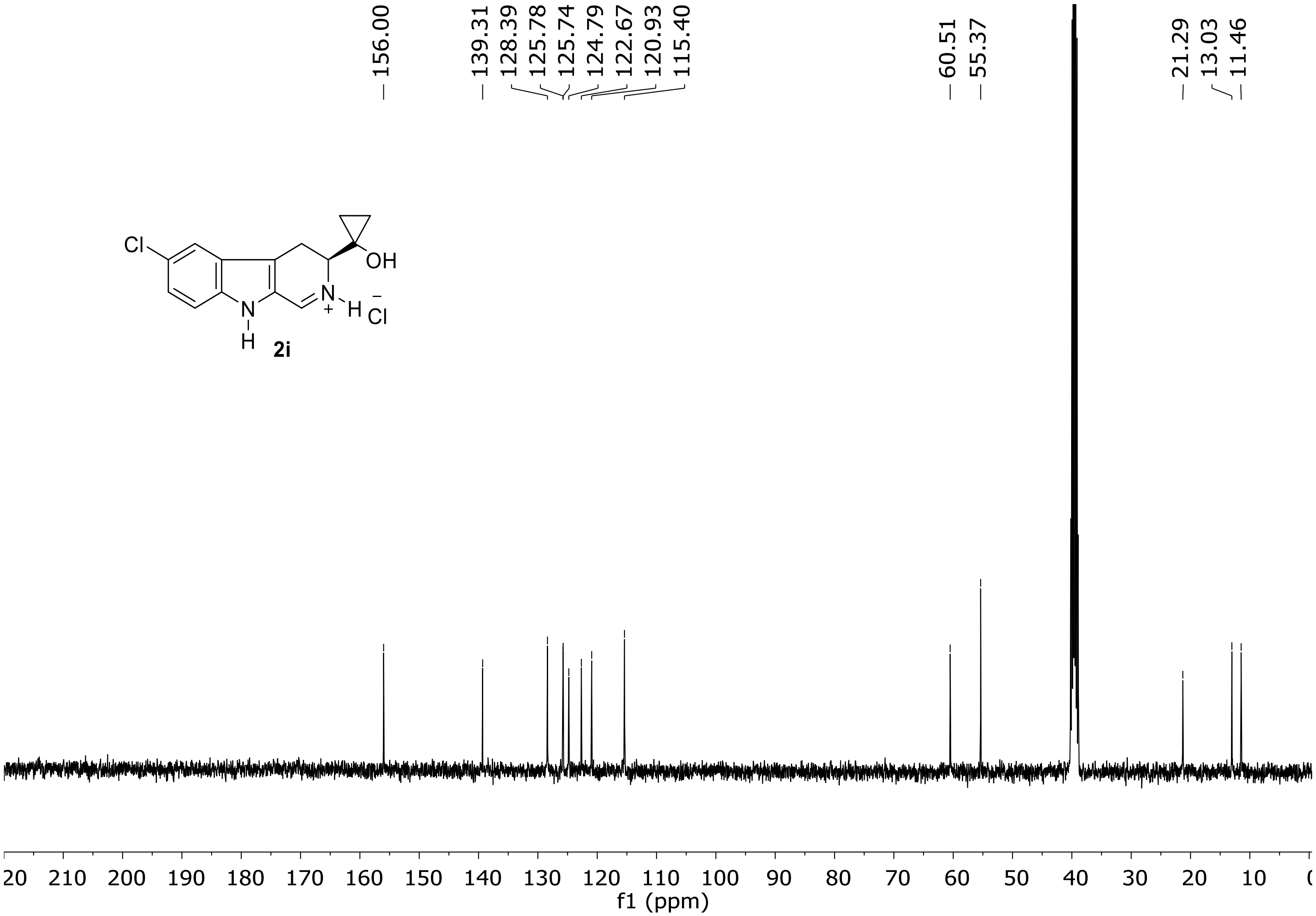
2i
156.00
139.31
128.39
125.78
125.74
124.79
122.67
120.93
115.40
60.51
55.37
21.29
13.03
11.46
220 210 200 190 180 170 160 150 140 130 120 110 100 90 80 70 60 50 40 30 20 10 0
f1 (ppm)

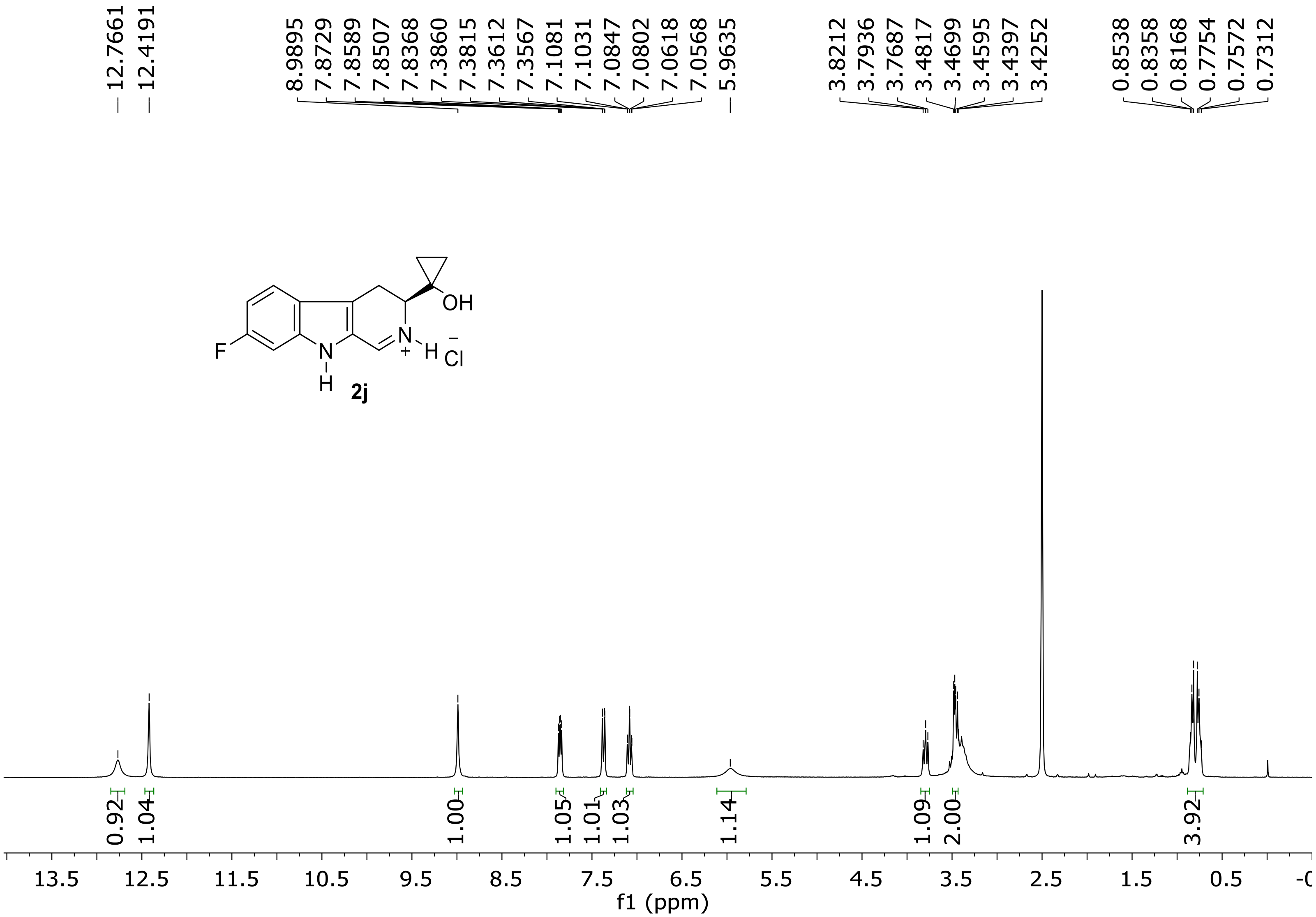
12.7661
12.4191
8.9895
7.8729
7.8589
7.8507
7.8368
7.3860
7.3815
7.3612
7.3567
7.1081
7.1031
7.0847
7.0802
7.0618
7.0568
5.9635
3.8212
3.7936
3.7687
3.4817
3.4699
3.4595
3.4397
3.4252
0.8538
0.8358
0.8168
0.7754
0.7572
0.7312
OH
F
N
H
N
H
+
–
Cl
2j
0.92
1.04
1.00
1.05
1.01
1.03
1.14
1.09
2.00
3.92
13.5
12.5
11.5
10.5
9.5
8.5
7.5
6.5
5.5
4.5
3.5
2.5
1.5
0.5
f1 (ppm)

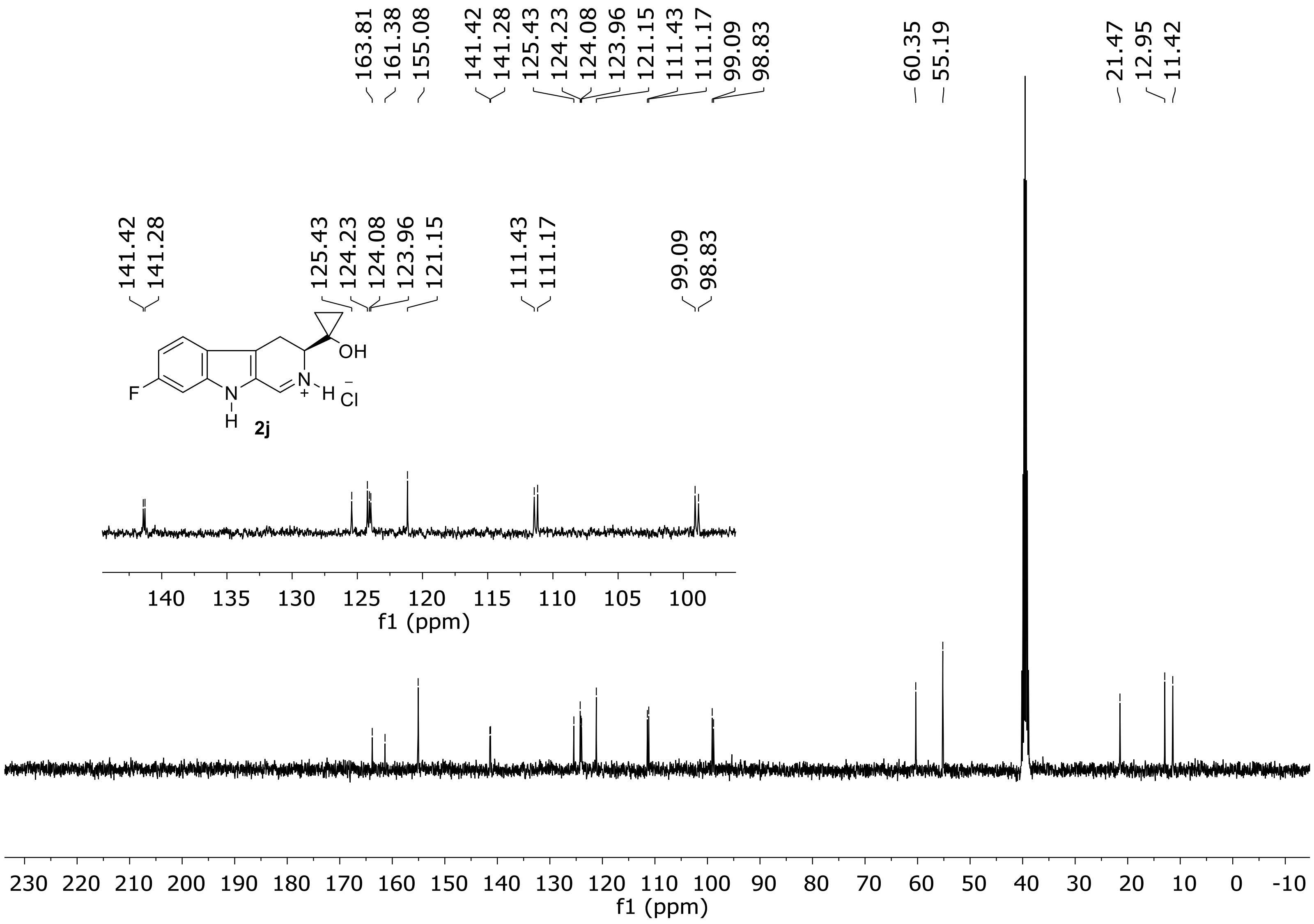
163.81
161.38
155.08
141.42
141.28
125.43
124.23
124.08
123.96
121.15
111.43
111.17
99.09
98.83
60.35
55.19
21.47
12.95
11.42
OH
F
N
H
N
H
Cl
2j
f1 (ppm)
f1 (ppm)

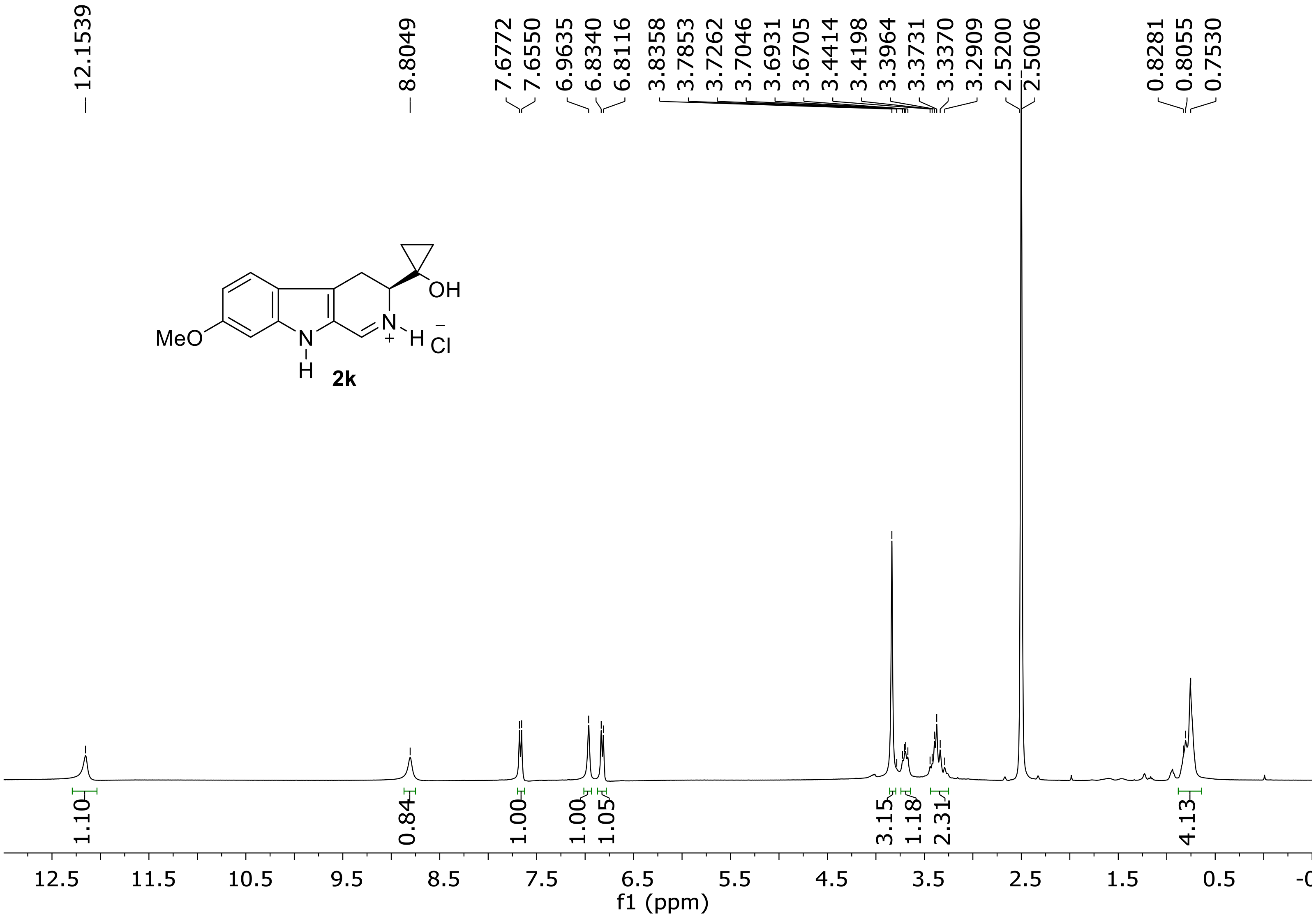

12.1539
8.8049
7.6772
7.6550
6.9635
6.8340
6.8116
3.8358
3.7853
3.7262
3.7046
3.6931
3.6705
3.4414
3.4198
3.3964
3.3731
3.3370
3.2909
2.5200
2.5006
0.8281
0.8055
0.7530
MeO
N
H
OH
N
H
Cl
2k
1.10
0.84
1.00
1.00
1.05
3.15
1.18
2.31
4.13
12.5
11.5
10.5
9.5
8.5
7.5
6.5
5.5
4.5
3.5
2.5
1.5
0.5
f1 (ppm)

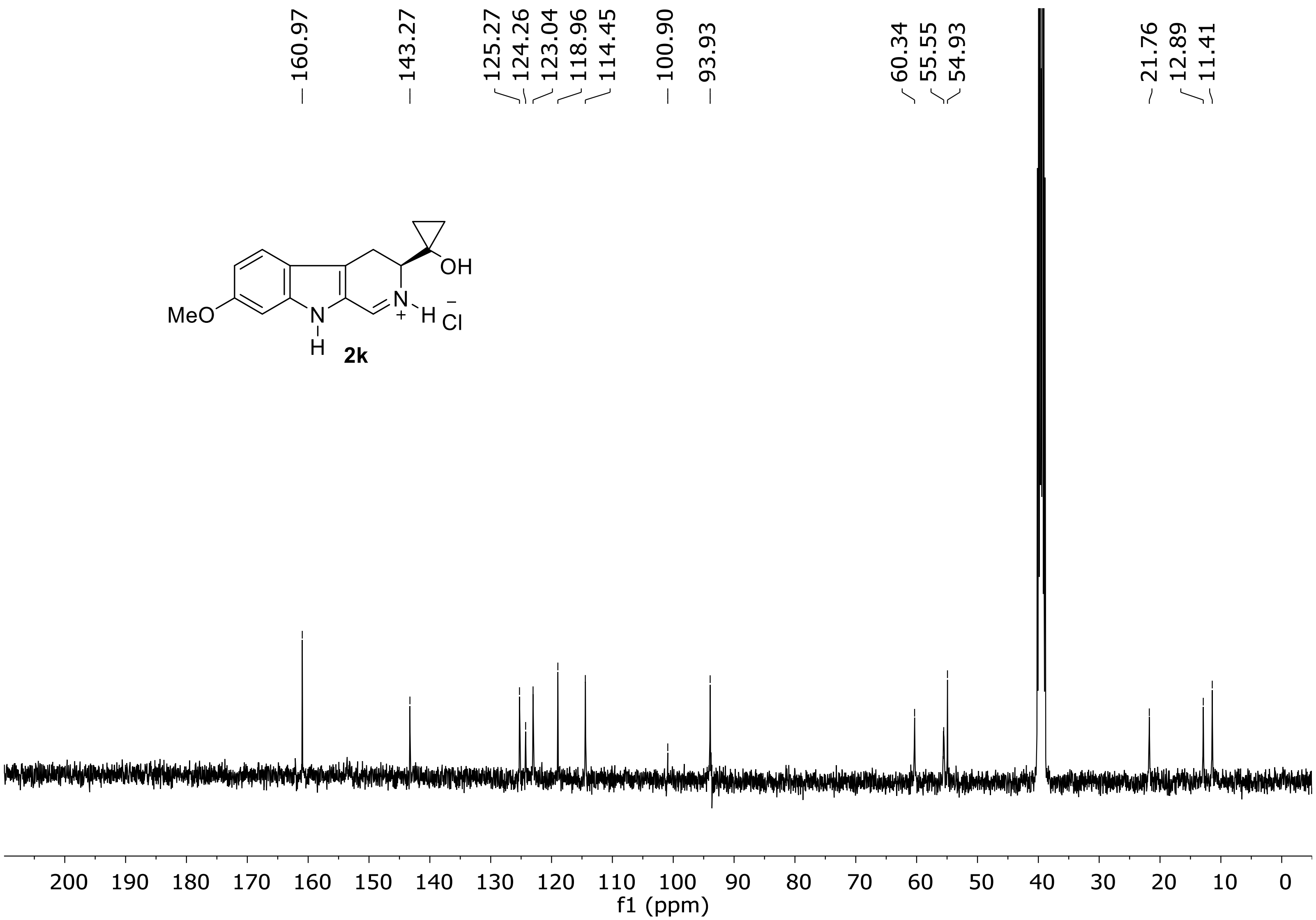
160.97
143.27
125.27
124.26
123.04
118.96
114.45
100.90
93.93
60.34
55.55
54.93
21.76
12.89
11.41
MeO
N
H
N
+
H
OH
Cl
–
2k
200 190 180 170 160 150 140 130 120 110 100 90 80 70 60 50 40 30 20 10 0
f1 (ppm)

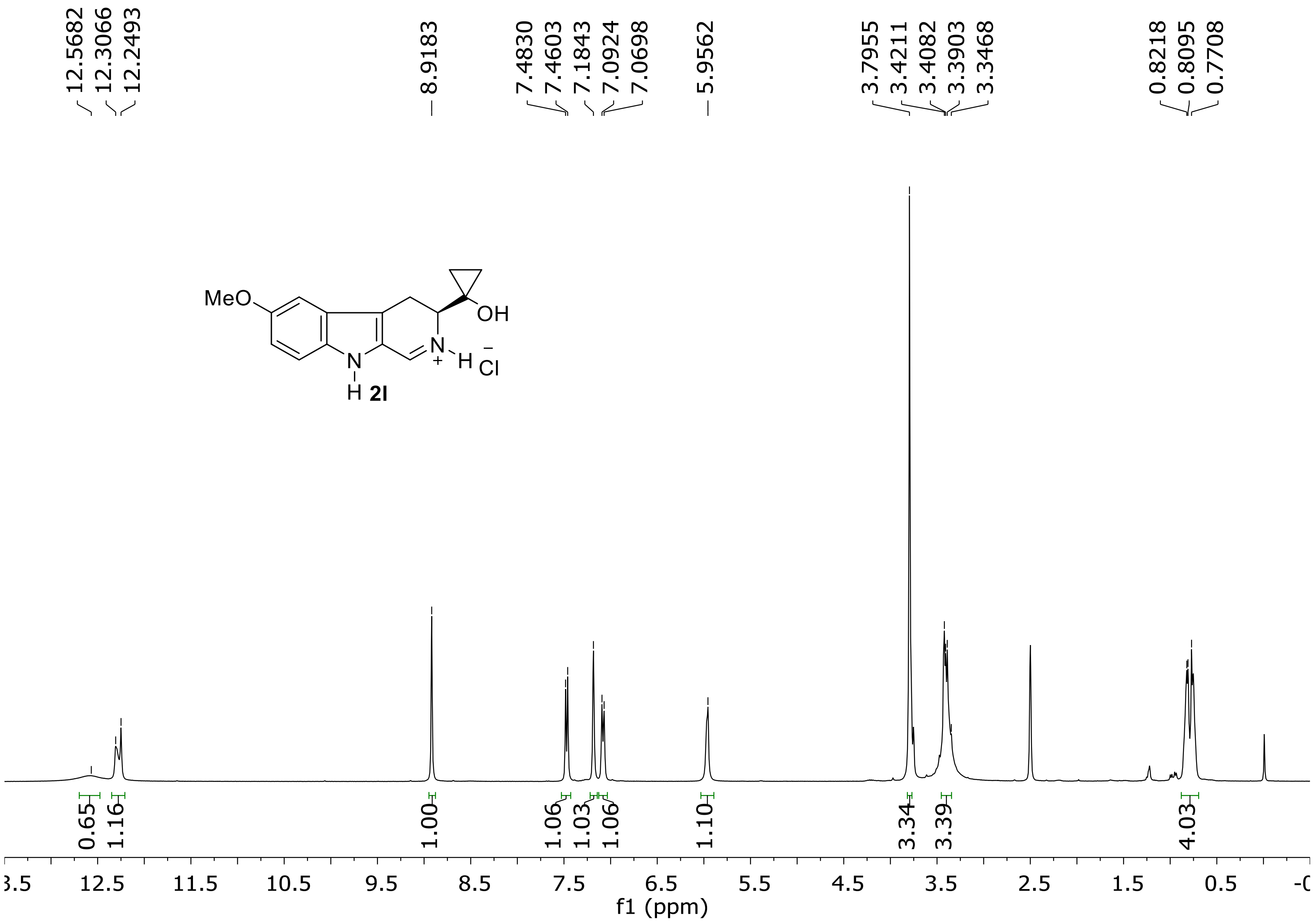
12.5682
12.3066
12.2493
8.9183
7.4830
7.4603
7.1843
7.0924
7.0698
5.9562
3.7955
3.4211
3.4082
3.3903
3.3468
0.8218
0.8095
0.7708
MeO
OH
N
H
Cl
2l
0.65
1.16
1.00
1.06
1.03
1.06
1.10
3.34
3.39
4.03
12.5
11.5
10.5
9.5
8.5
7.5
6.5
5.5
4.5
3.5
2.5
1.5
0.5
f1 (ppm)

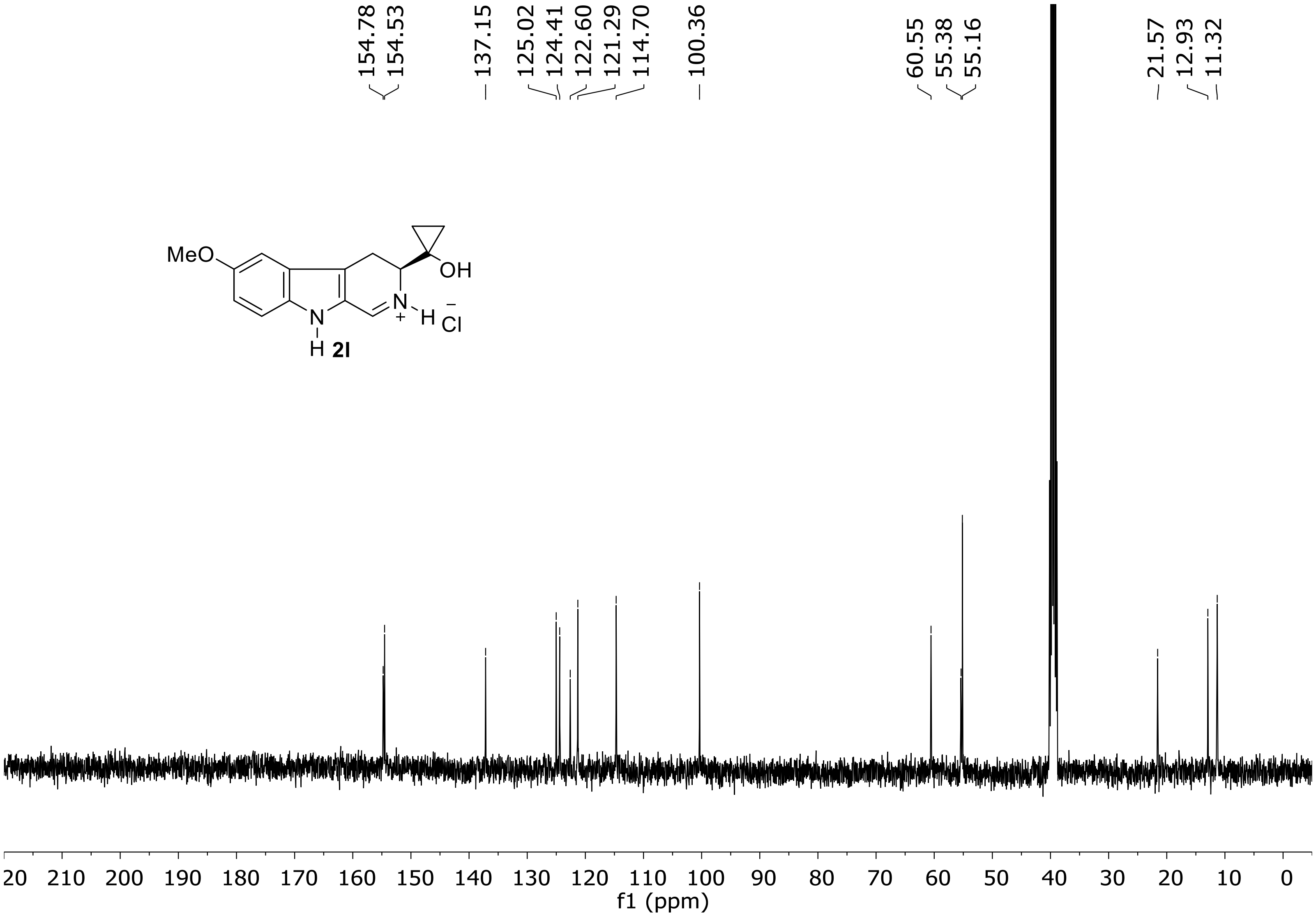
154.78
154.53
137.15
125.02
124.41
122.60
121.29
114.70
100.36
60.55
55.38
55.16
21.57
12.93
11.32
MeO
OH
N
H
Cl
2l
20 210 200 190 180 170 160 150 140 130 120 110 100 90 80 70 60 50 40 30 20 10 0
f1 (ppm)

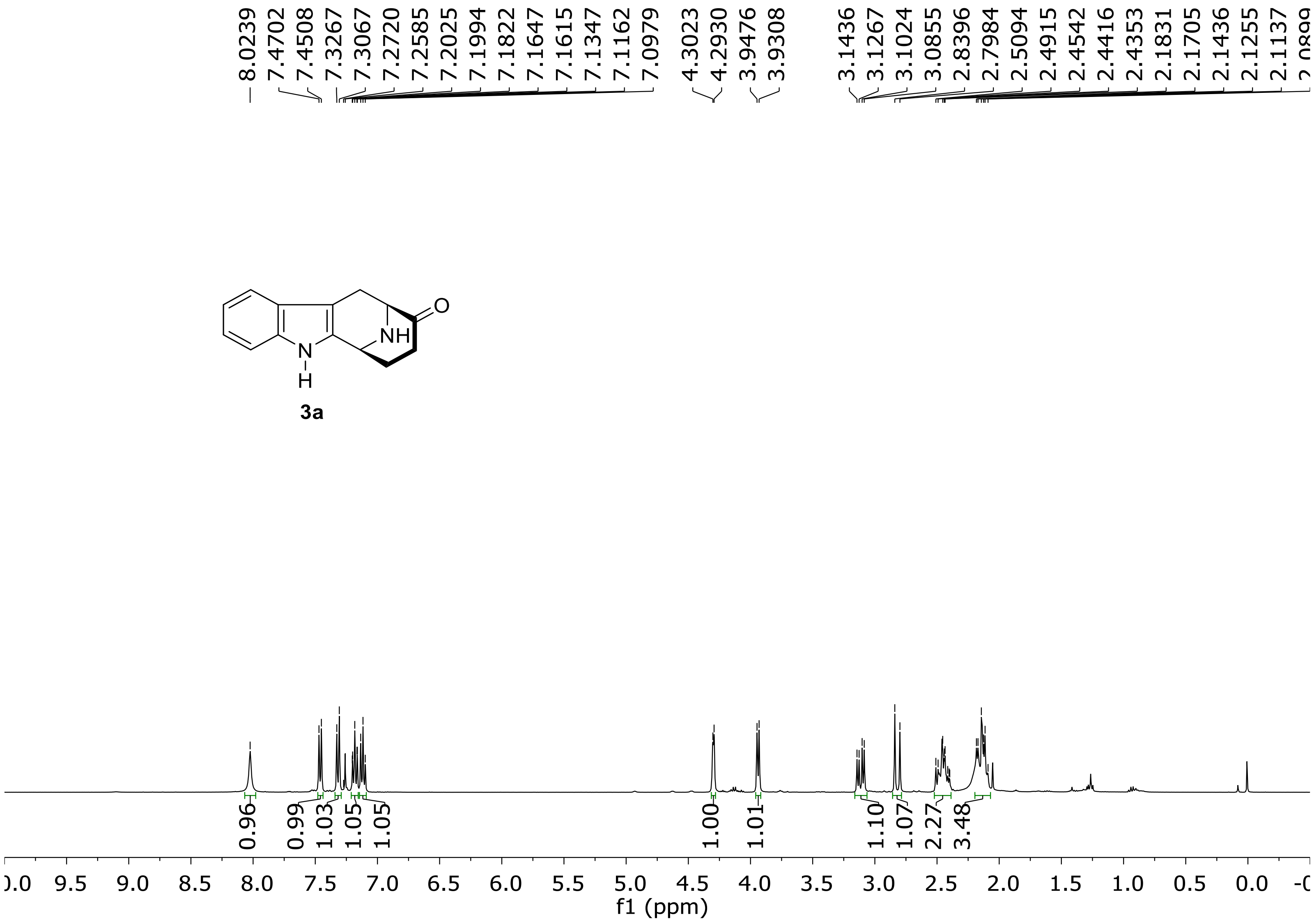
8.0239
7.4702
7.4508
7.3267
7.3067
7.2720
7.2585
7.2025
7.1994
7.1822
7.1647
7.1615
7.1347
7.1162
7.0979
4.3023
4.2930
3.9476
3.9308
3.1436
3.1267
3.1024
3.0855
2.8396
2.7984
2.5094
2.4915
2.4542
2.4416
2.4353
2.1831
2.1705
2.1436
2.1255
2.1137
NH
N
H
0.96
0.99
1.03
1.05
1.05
1.00
1.01
1.10
1.07
2.27
3.48
9.5
9.0
8.5
8.0
7.5
7.0
6.5
6.0
5.5
5.0
4.5
4.0
3.5
3.0
2.5
2.0
1.5
1.0
0.5
0.0
f1 (ppm)

3a

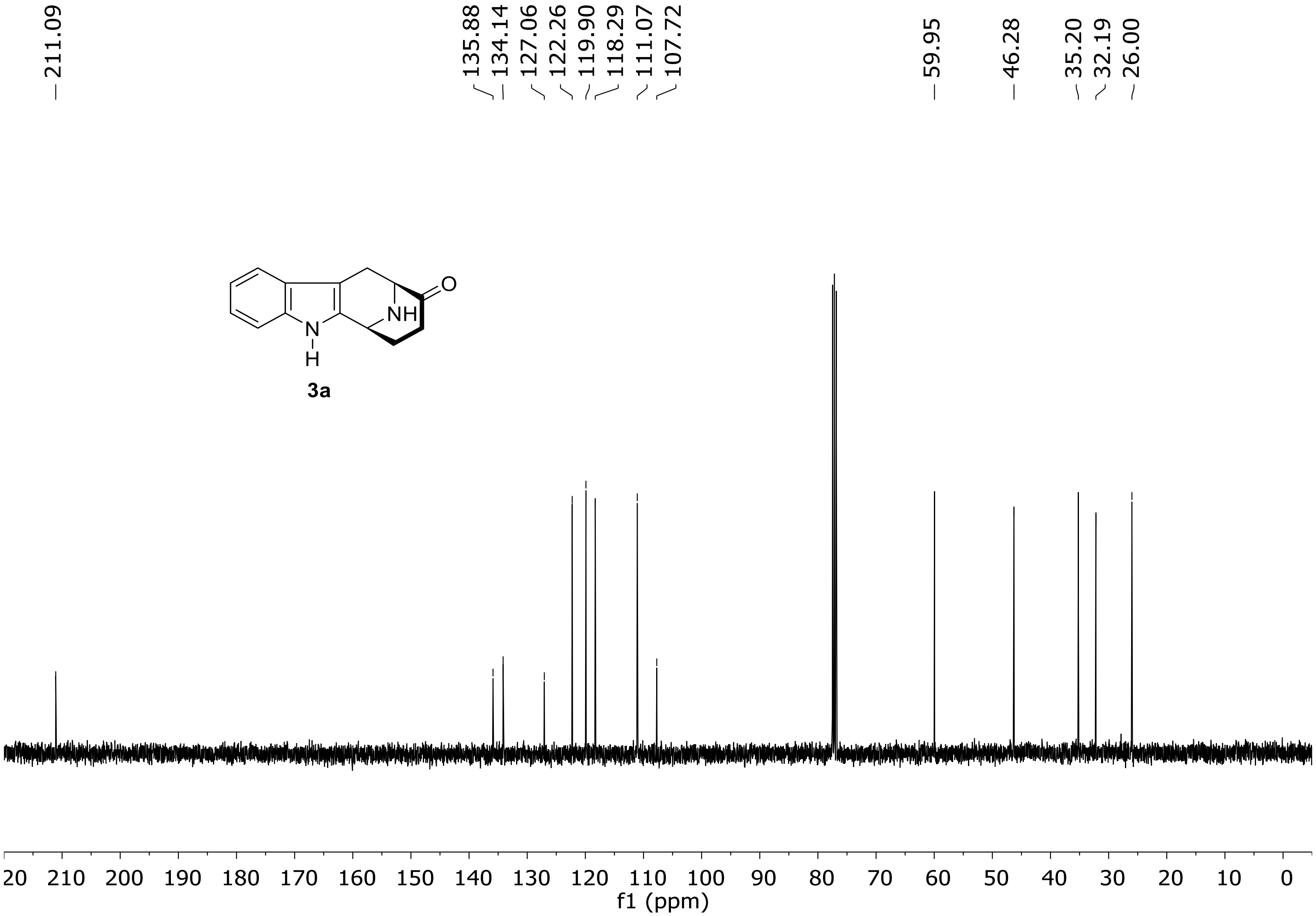
211.09
135.88
134.14
127.06
122.26
119.90
118.29
111.07
107.72
59.95
46.28
35.20
32.19
26.00
O
NH
N
H
3a
20 210 200 190 180 170 160 150 140 130 120 110 100 90 80 70 60 50 40 30 20 10 0
f1 (ppm)

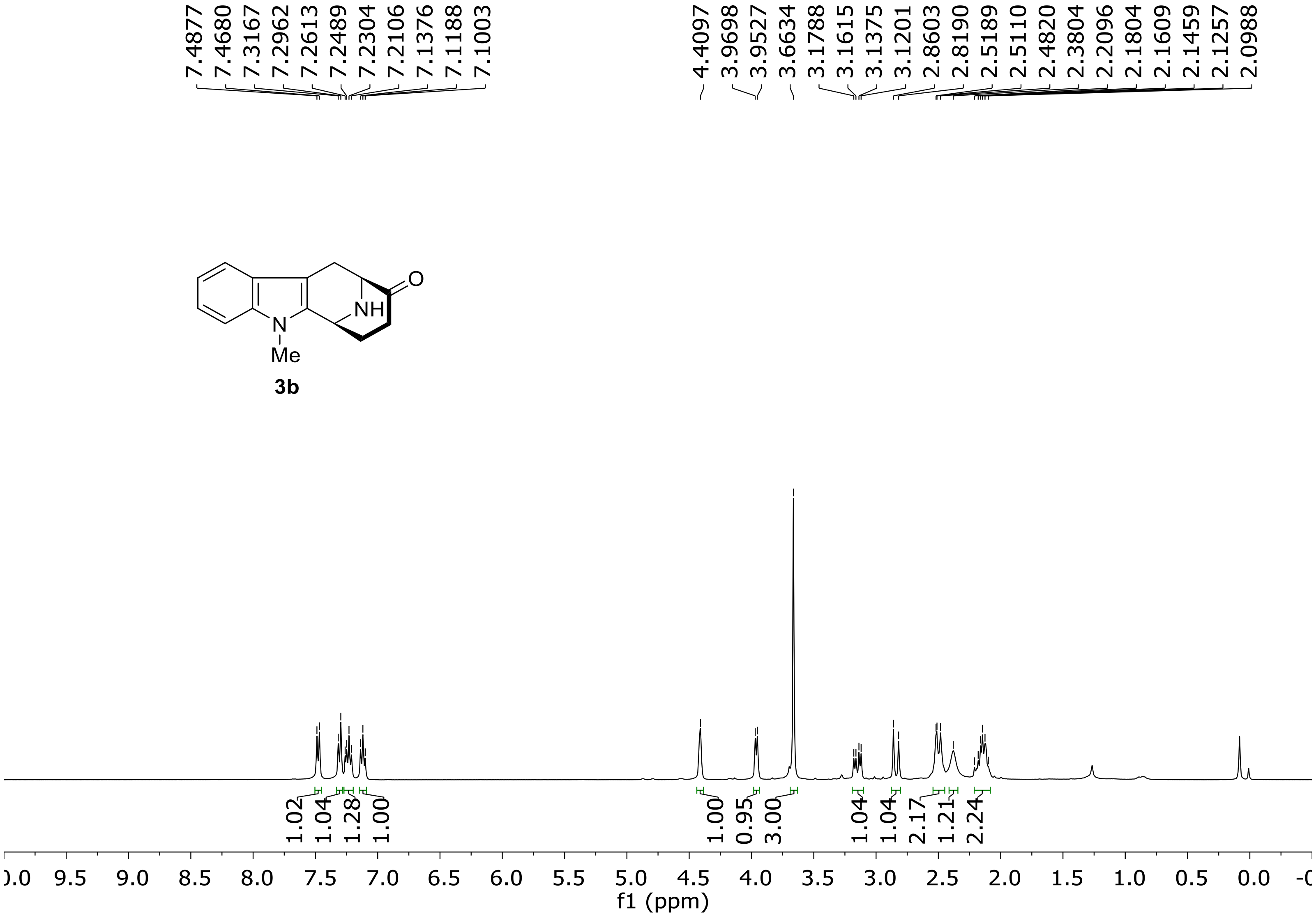
7.4877 7.4680 7.3167 7.2962 7.2613 7.2489 7.2304 7.2106 7.1376 7.1188 7.1003
4.4097 3.9698 3.9527 3.6634 3.1788 3.1615 3.1375 3.1201 2.8603 2.8190 2.5189 2.5110 2.4820 2.3804 2.2096 2.1804 2.1609 2.1459 2.1257 2.0988
NH
O
N
Me
3b
1.02 1.04 1.28 1.00 1.00 0.95 3.00 1.04 1.04 2.17 1.21 2.24
9.5 9.0 8.5 8.0 7.5 7.0 6.5 6.0 5.5 5.0 4.5 4.0 3.5 3.0 2.5 2.0 1.5 1.0 0.5 0.0
f1 (ppm)

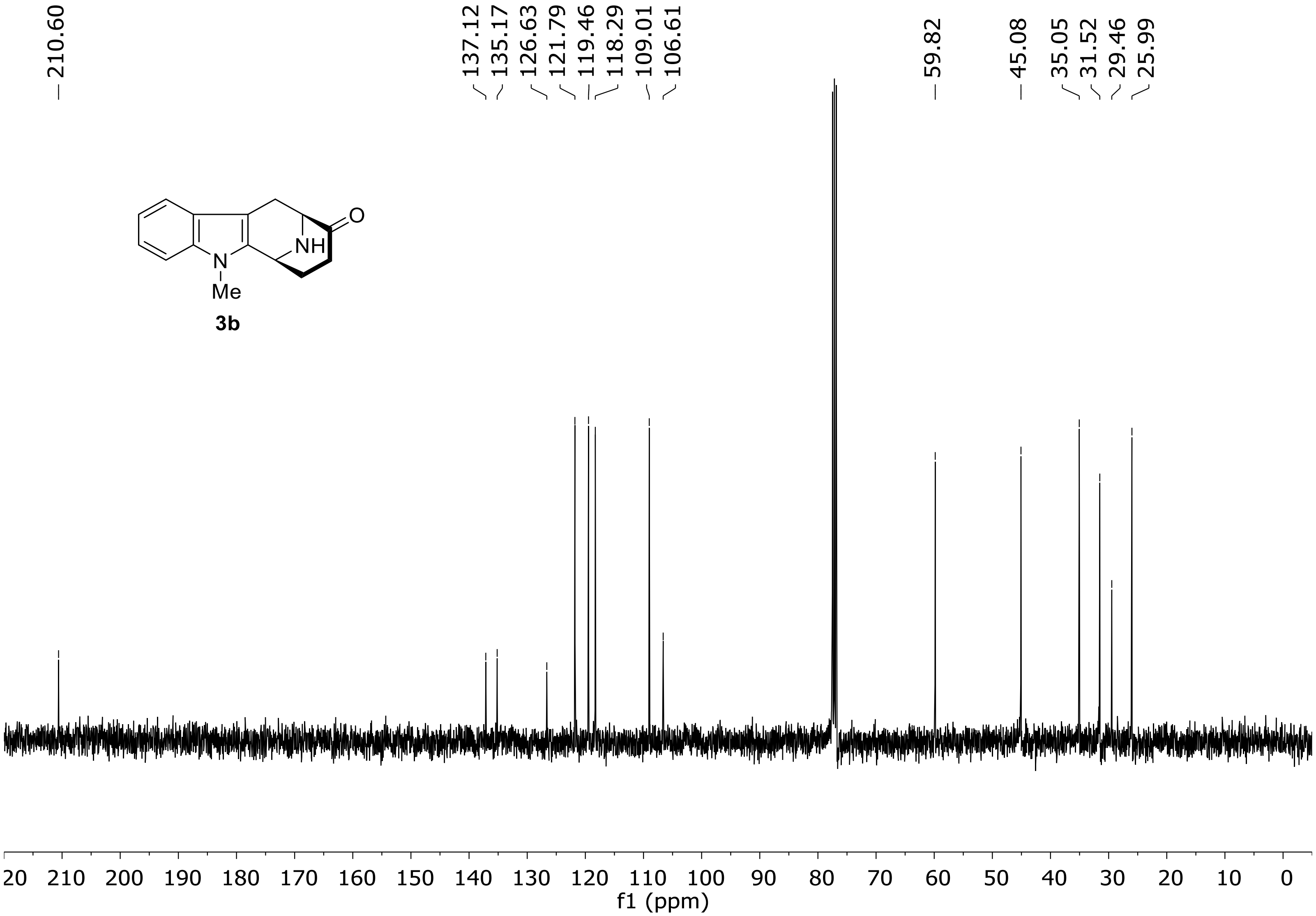
210.60
137.12
135.17
126.63
121.79
119.46
118.29
109.01
106.61
59.82
45.08
35.05
31.52
29.46
25.99
NH
O
N
Me
3b
20 210 200 190 180 170 160 150 140 130 120 110 100 90 80 70 60 50 40 30 20 10 0
f1 (ppm)

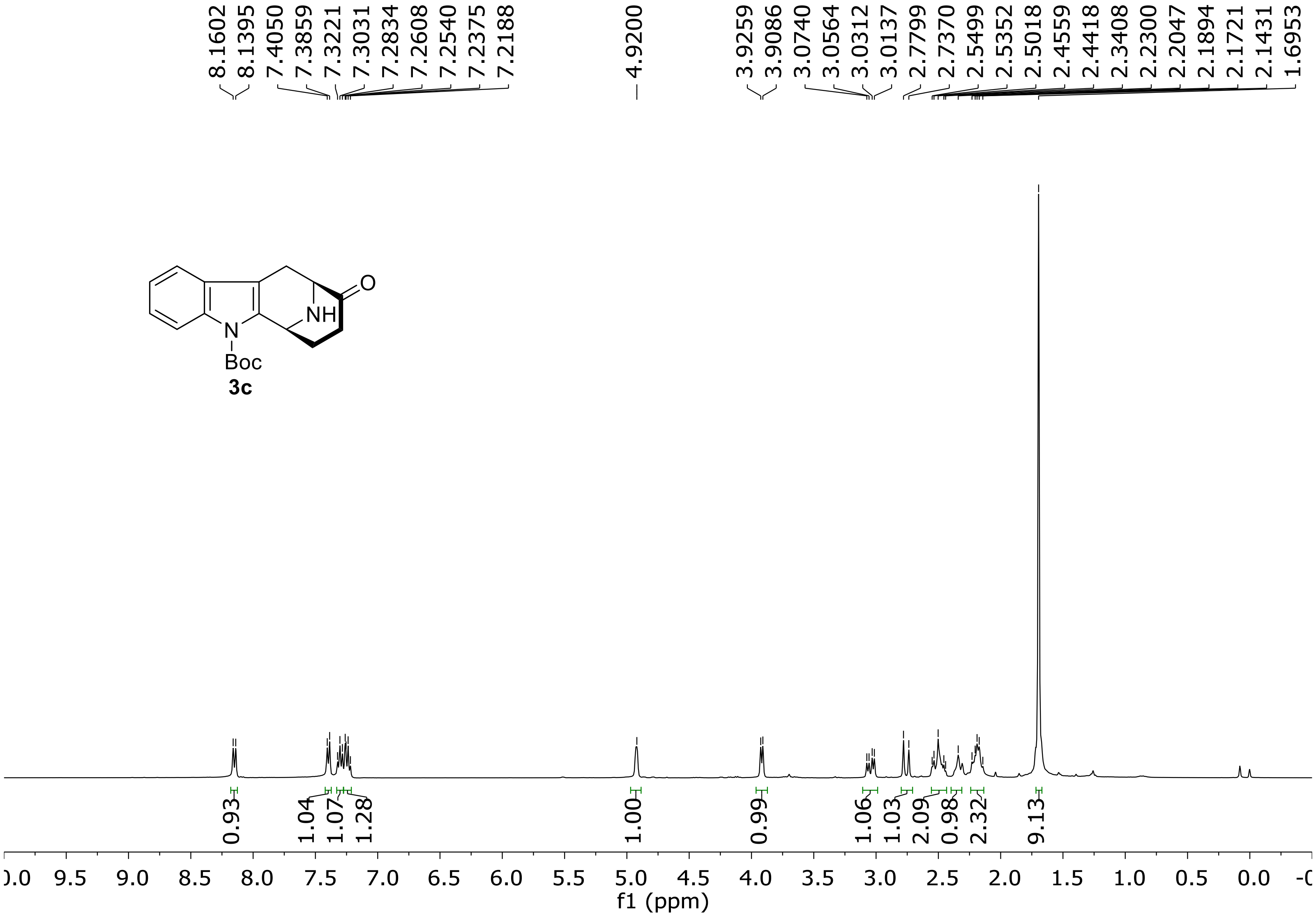
8.1602
8.1395
7.4050
7.3859
7.3221
7.3031
7.2834
7.2608
7.2540
7.2375
7.2188
4.9200
3.9259
3.9086
3.0740
3.0564
3.0312
3.0137
2.7799
2.7370
2.5499
2.5352
2.5018
2.4559
2.4418
2.3408
2.2300
2.2047
2.1894
2.1721
2.1431
1.6953
O
NH
N
Boc
3c
0.93
1.04
1.07
1.28
1.00
0.99
1.06
1.03
2.09
0.98
2.32
9.13
9.5
9.0
8.5
8.0
7.5
7.0
6.5
6.0
5.5
5.0
4.5
4.0
3.5
3.0
2.5
2.0
1.5
1.0
0.5
0.0
f1 (ppm)

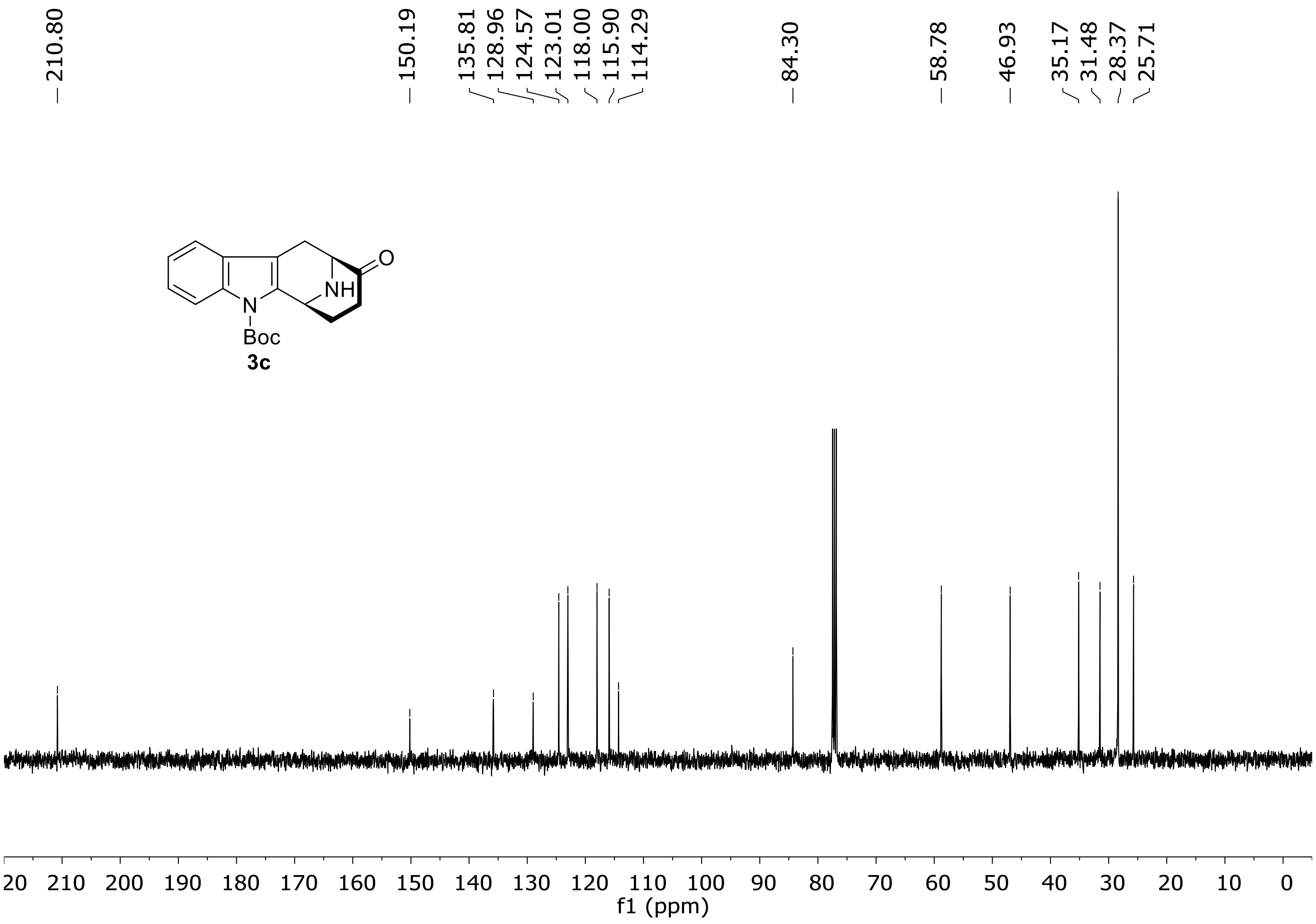
N
Boc
3c
NH
O
210.80
150.19
135.81
128.96
124.57
123.01
118.00
115.90
114.29
84.30
58.78
46.93
35.17
31.48
28.37
25.71
20
210
200
190
180
170
160
150
140
130
120
110
100
90
80
70
60
50
40
30
20
10
0
f1 (ppm)

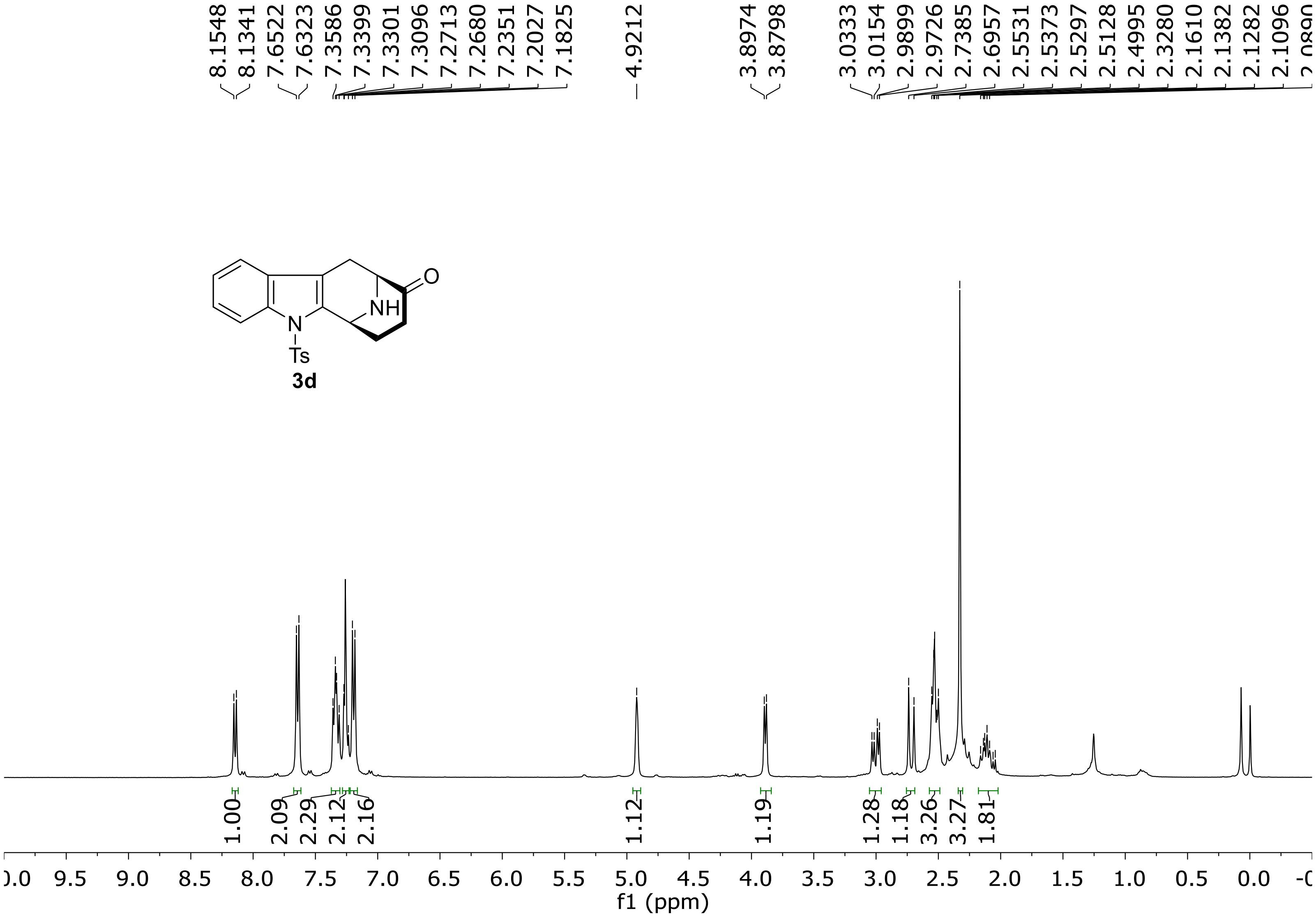
8.1548
8.1341
7.6522
7.6323
7.3586
7.3399
7.3301
7.3096
7.2713
7.2680
7.2351
7.2027
7.1825
4.9212
3.8974
3.8798
3.0333
3.0154
2.9899
2.9726
2.7385
2.6957
2.5531
2.5373
2.5297
2.5128
2.4995
2.3280
2.1610
2.1382
2.1282
2.1096
N
Ts
NH
O
3d
1.00
2.09
2.29
2.12
2.16
1.12
1.19
1.28
1.18
3.26
3.27
1.81
9.5
9.0
8.5
8.0
7.5
7.0
6.5
6.0
5.5
5.0
4.5
4.0
3.5
3.0
2.5
2.0
1.5
1.0
0.5
0.0
f1 (ppm)

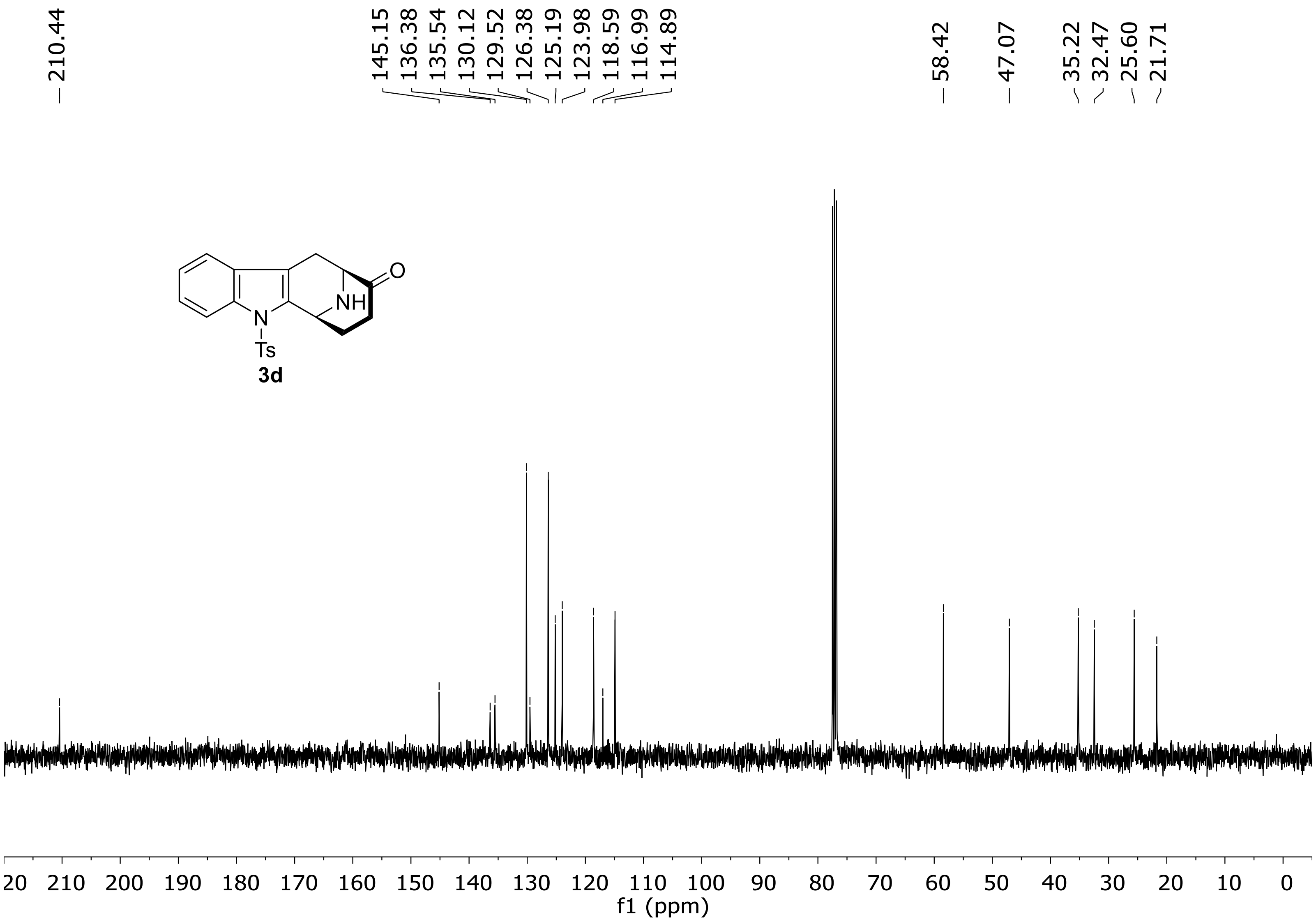
210.44
145.15
136.38
135.54
130.12
129.52
126.38
125.19
123.98
118.59
116.99
114.89
58.42
47.07
35.22
32.47
25.60
21.71
NH
O
N
Ts
3d
20 210 200 190 180 170 160 150 140 130 120 110 100 90 80 70 60 50 40 30 20 10 0
f1 (ppm)

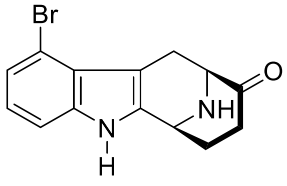

3e

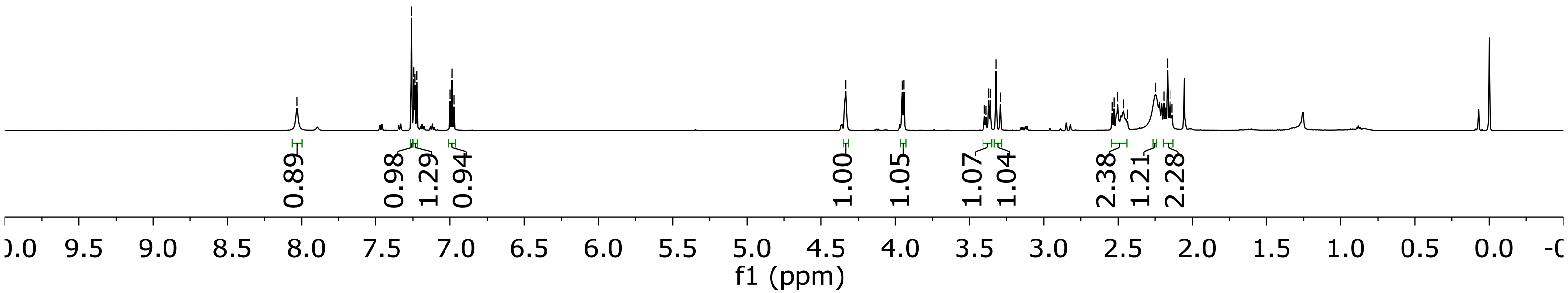

8.0316
7.2598
7.2447
7.2372
7.2244
6.9990
6.9859
6.9728
4.3330
3.9539
3.9429
3.3998
3.3886
3.3714
3.3602
3.3221
3.2938
2.5397
2.5268
2.5035
2.4623
2.4342
2.2473
2.1911
2.1669
2.1497
2.1355
0.89
0.98
1.29
0.94
1.00
1.05
1.07
1.04
2.38
1.21
2.28
f1 (ppm)

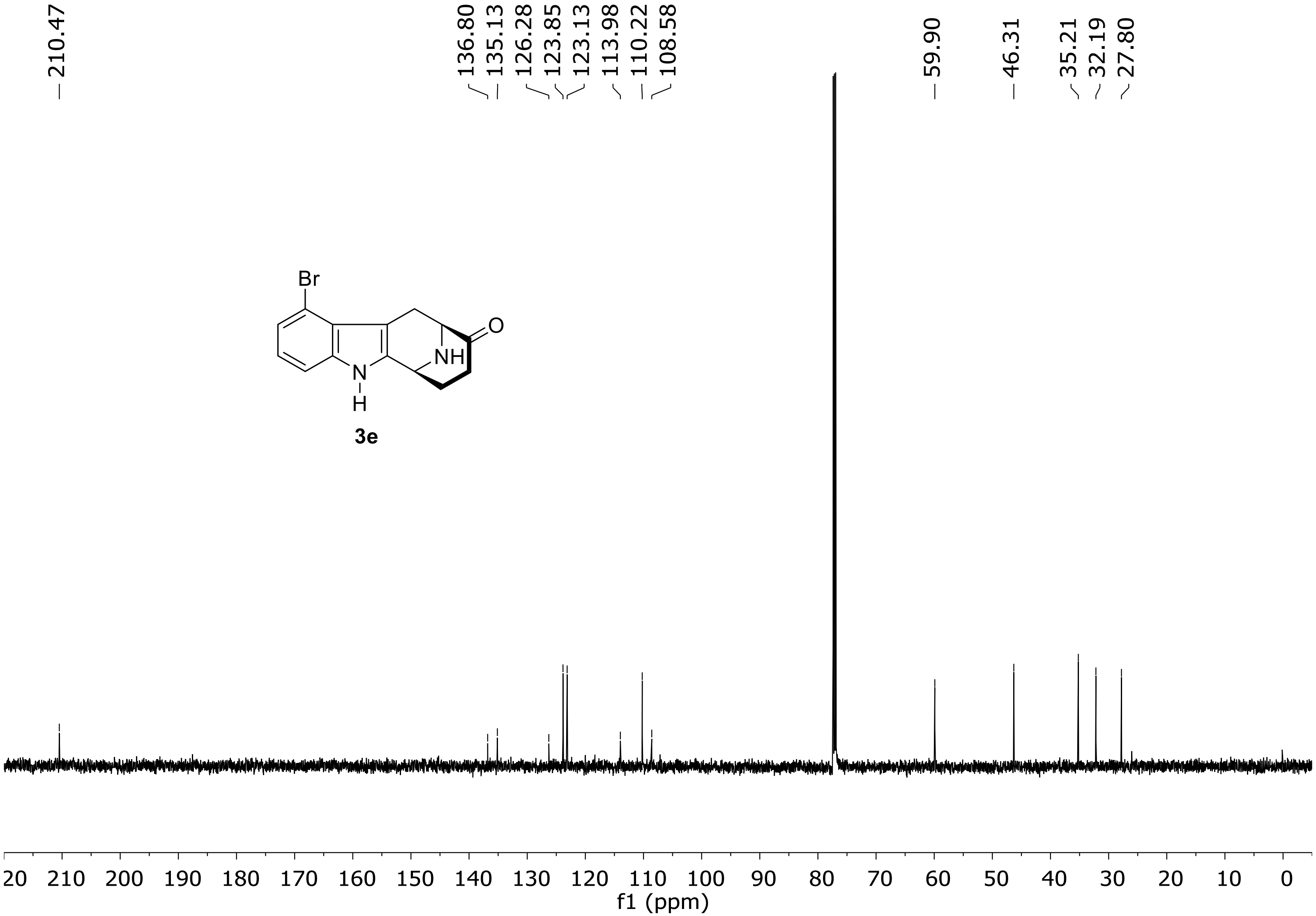
210.47
136.80
135.13
126.28
123.85
123.13
113.98
110.22
108.58
59.90
46.31
35.21
32.19
27.80
Br
O
NH
N
H
3e
20 210 200 190 180 170 160 150 140 130 120 110 100 90 80 70 60 50 40 30 20 10 0
f1 (ppm)

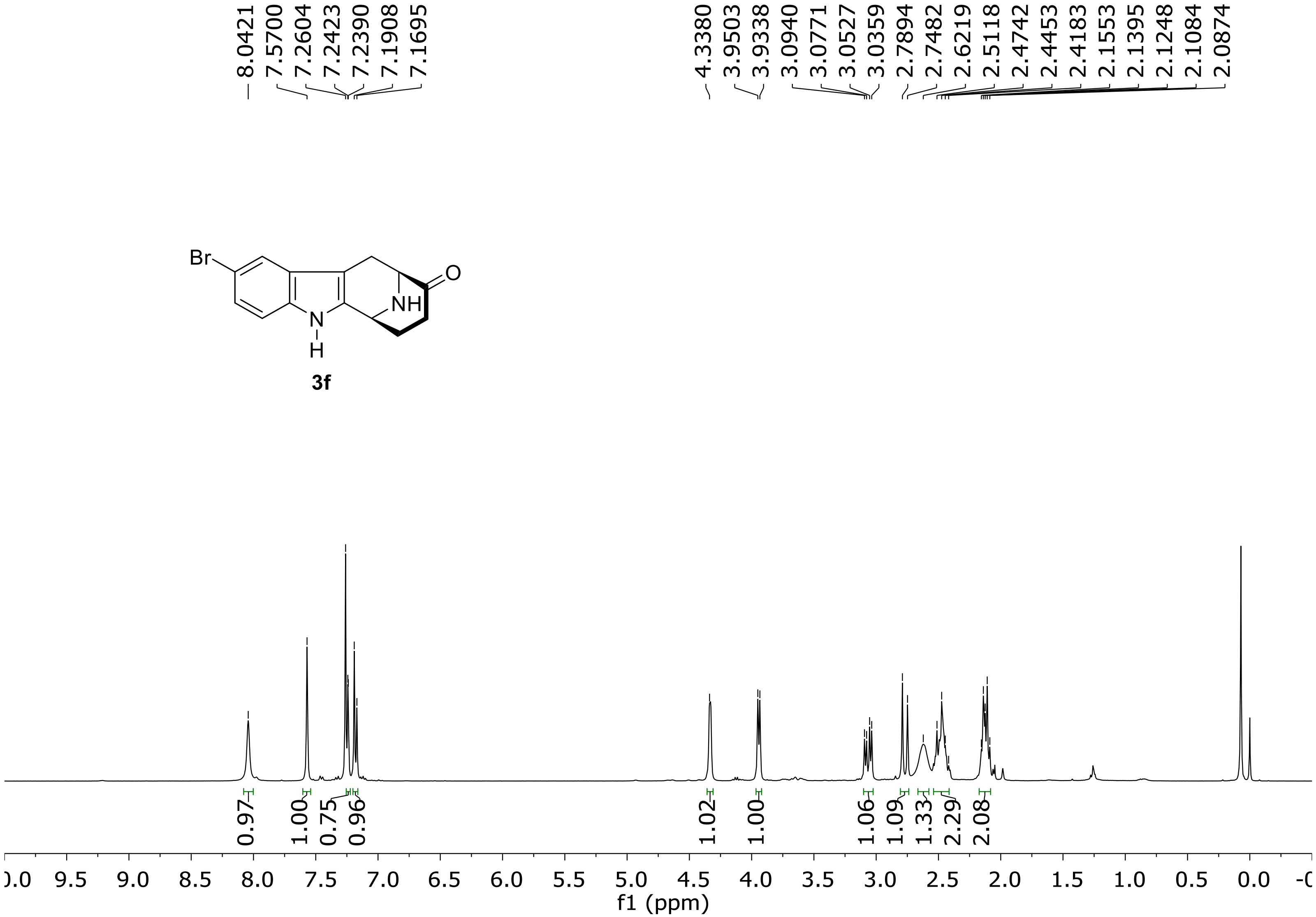
8.0421
7.5700
7.2604
7.2423
7.2390
7.1908
7.1695
4.3380
3.9503
3.9338
3.0940
3.0771
3.0527
3.0359
2.7894
2.7482
2.6219
2.5118
2.4742
2.4453
2.4183
2.1553
2.1395
2.1248
2.1084
2.0874
Br
NH
O
N
H
3f
0.97
1.00
0.75
0.96
1.02
1.00
1.06
1.09
1.33
2.29
2.08
f1 (ppm)

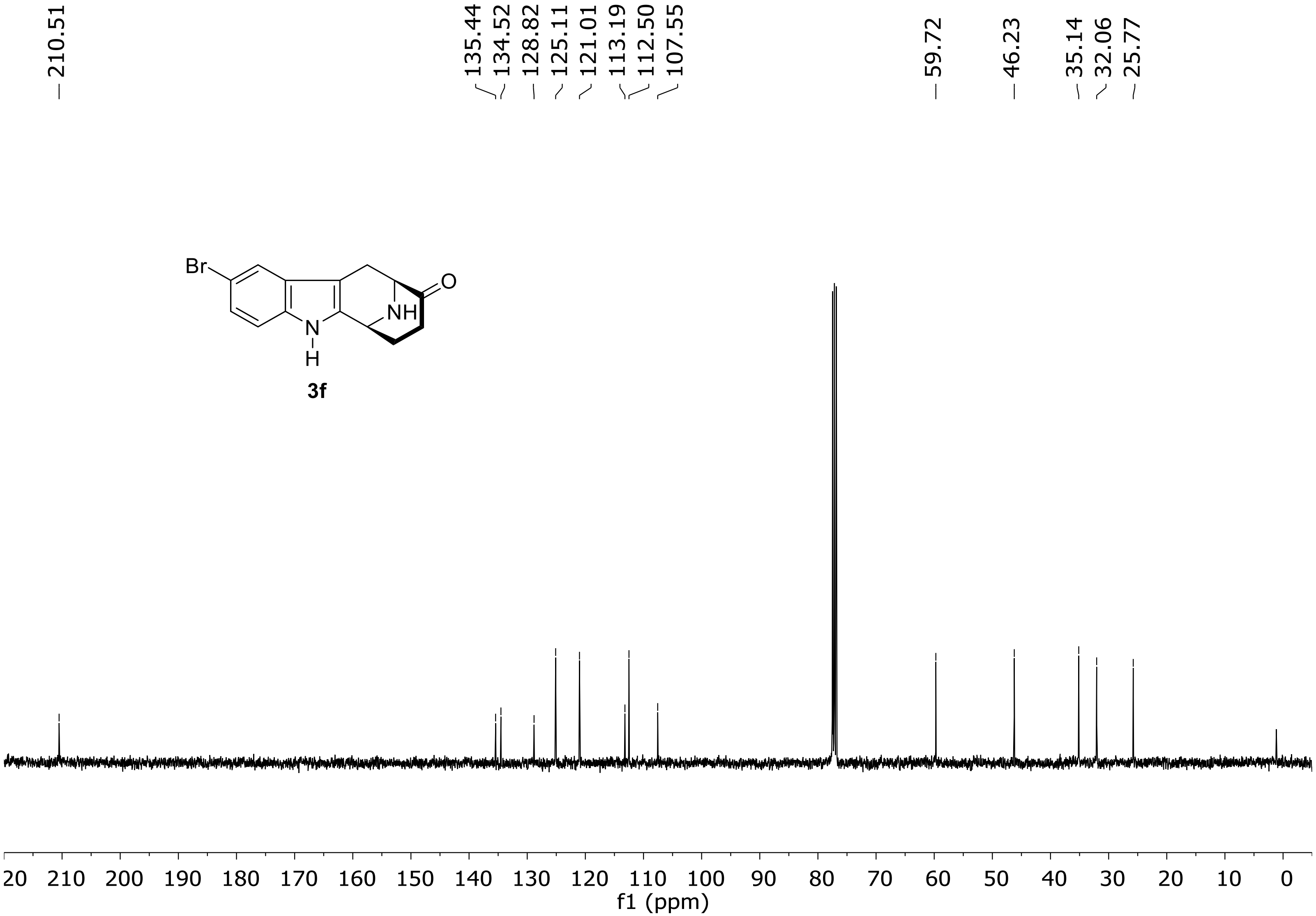

210.51
135.44
134.52
128.82
125.11
121.01
113.19
112.50
107.55
59.72
46.23
35.14
32.06
25.77
Br
NH
O
N
H
3f
20 210 200 190 180 170 160 150 140 130 120 110 100 90 80 70 60 50 40 30 20 10 0
f1 (ppm)

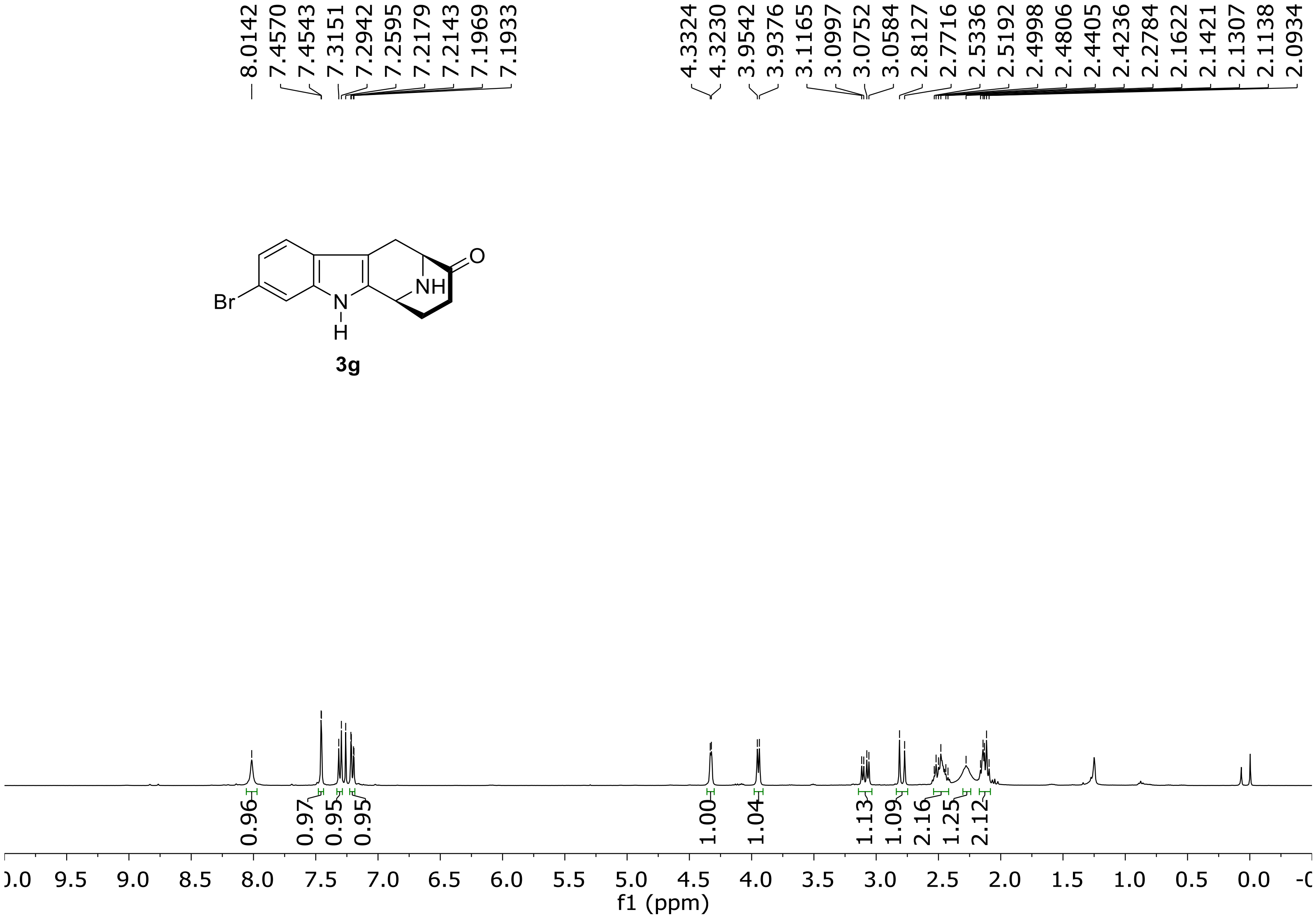
8.0142
7.4570
7.4543
7.3151
7.2942
7.2595
7.2179
7.2143
7.1969
7.1933
4.3324
4.3230
3.9542
3.9376
3.1165
3.0997
3.0752
3.0584
2.8127
2.7716
2.5336
2.5192
2.4998
2.4806
2.4405
2.4236
2.2784
2.1622
2.1421
2.1307
2.1138
2.0934
Br
N
H
NH
O
3g
0.96
0.97
0.95
0.95
1.00
1.04
1.13
1.09
2.16
1.25
2.12
9.5 9.0 8.5 8.0 7.5 7.0 6.5 6.0 5.5 5.0 4.5 4.0 3.5 3.0 2.5 2.0 1.5 1.0 0.5 0.0
f1 (ppm)

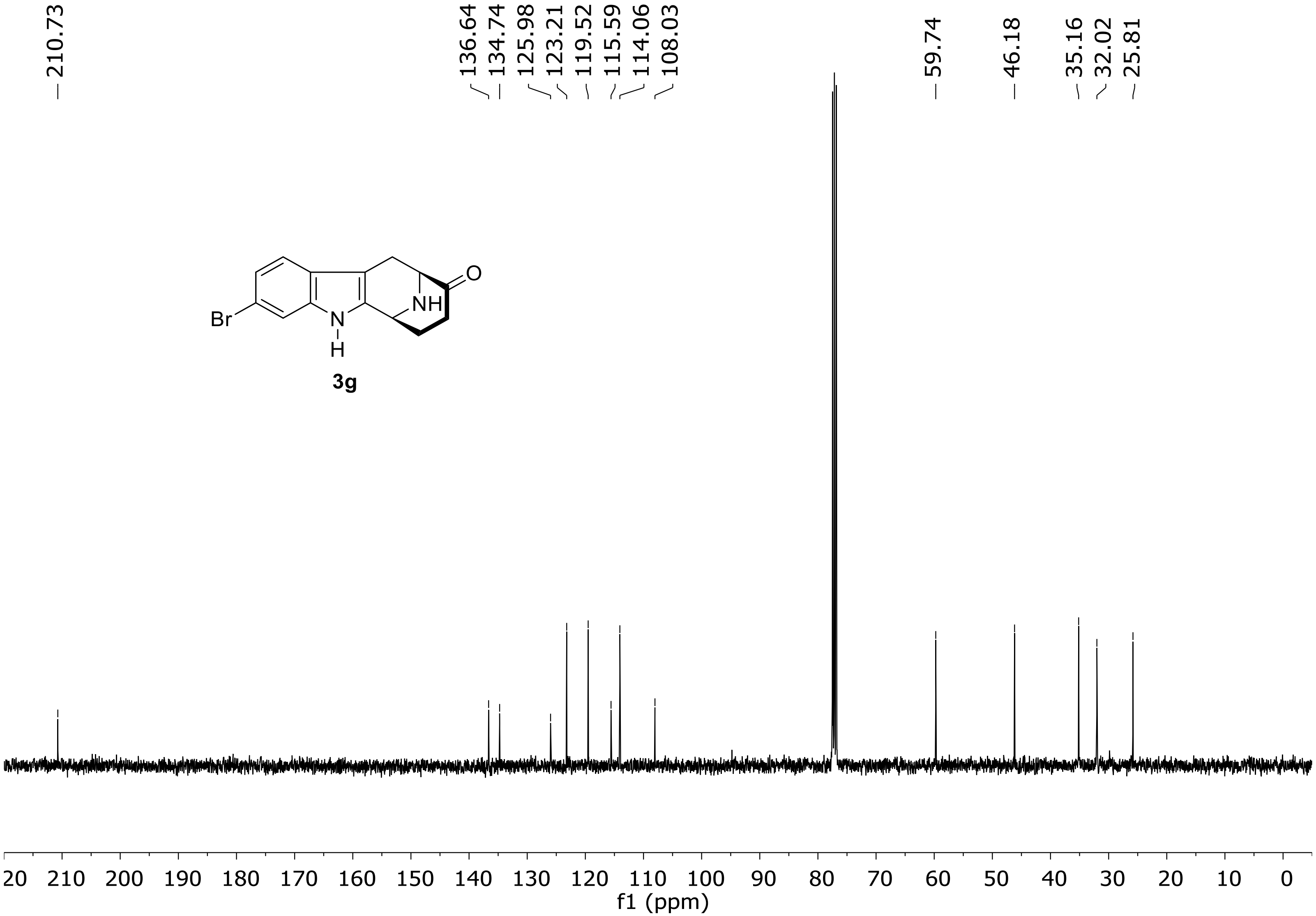

210.73
136.64
134.74
125.98
123.21
119.52
115.59
114.06
108.03
59.74
46.18
35.16
32.02
25.81
Br
N
H
NH
O
3g
20 210 200 190 180 170 160 150 140 130 120 110 100 90 80 70 60 50 40 30 20 10 0
f1 (ppm)

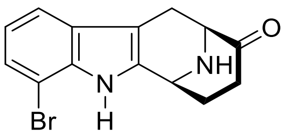

3h

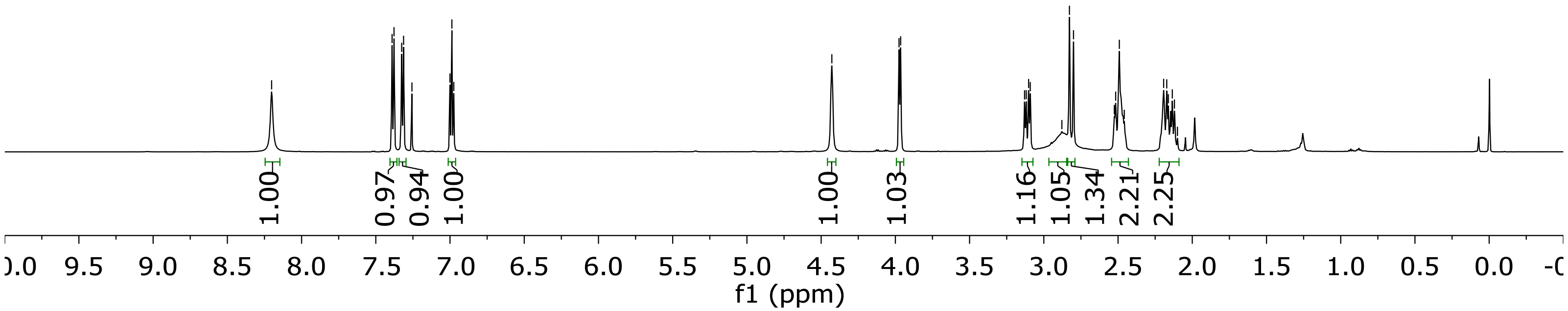

8.2017
7.3904
7.3773
7.3257
7.3130
7.2566
7.0003
6.9874
6.9746
4.4279
3.9754
3.9643
3.1298
3.1187
3.1024
3.0913
2.8784
2.8274
2.8000
2.5252
2.5160
2.4914
2.4580
2.1928
2.1719
2.1611
2.1345
2.1203
2.1001
1.00
0.97
0.94
1.00
1.00
1.03
1.16
1.05
1.34
2.21
2.25
f1 (ppm)

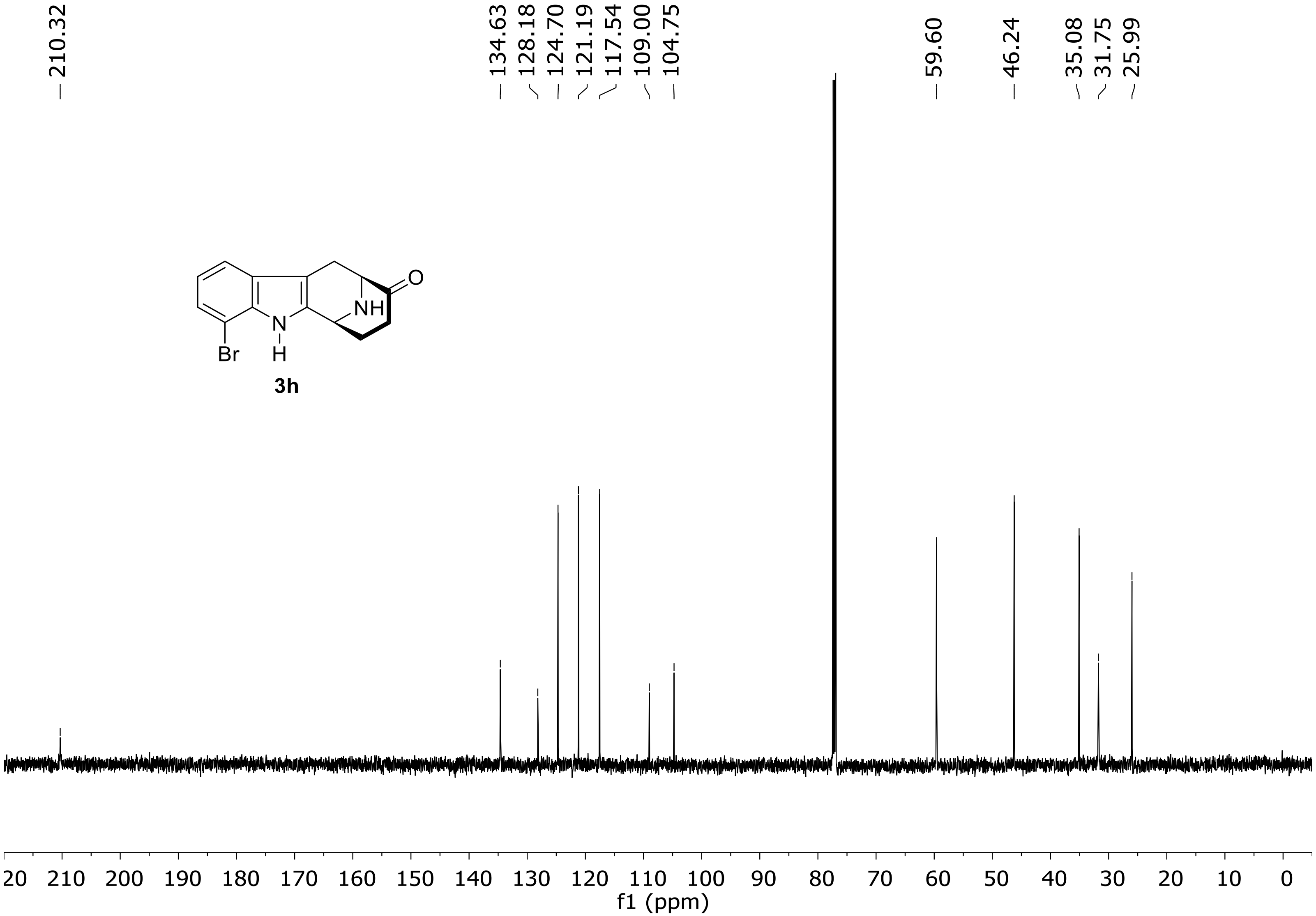
210.32
134.63
128.18
124.70
121.19
117.54
109.00
104.75
59.60
46.24
35.08
31.75
25.99
O
NH
N
H
Br
3h
20 210 200 190 180 170 160 150 140 130 120 110 100 90 80 70 60 50 40 30 20 10 0
f1 (ppm)

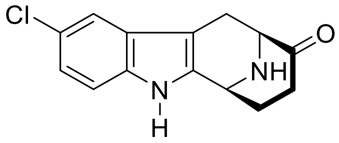
Cl
NH
O
N
H

3i

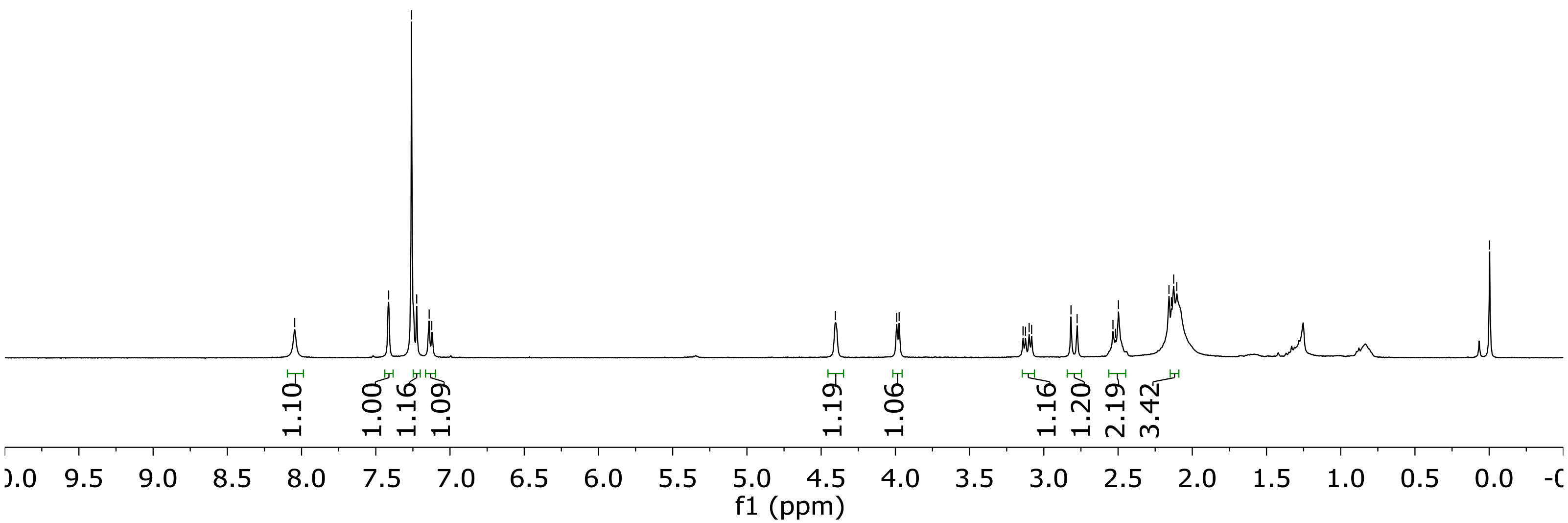
8.0463
7.4134
7.2593
7.2243
7.1405
7.1230
4.4038
3.9911
3.9747
3.1401
3.1233
3.0990
3.0821
2.8169
2.7757
2.5342
2.5170
2.4974
2.1570
2.1403
2.1254
2.1042
-0.0030
1.10
1.00
1.16
1.09
1.19
1.06
1.16
1.20
2.19
3.42
9.5
9.0
8.5
8.0
7.5
7.0
6.5
6.0
5.5
5.0
4.5
4.0
3.5
3.0
2.5
2.0
1.5
1.0
0.5
0.0
f1 (ppm)

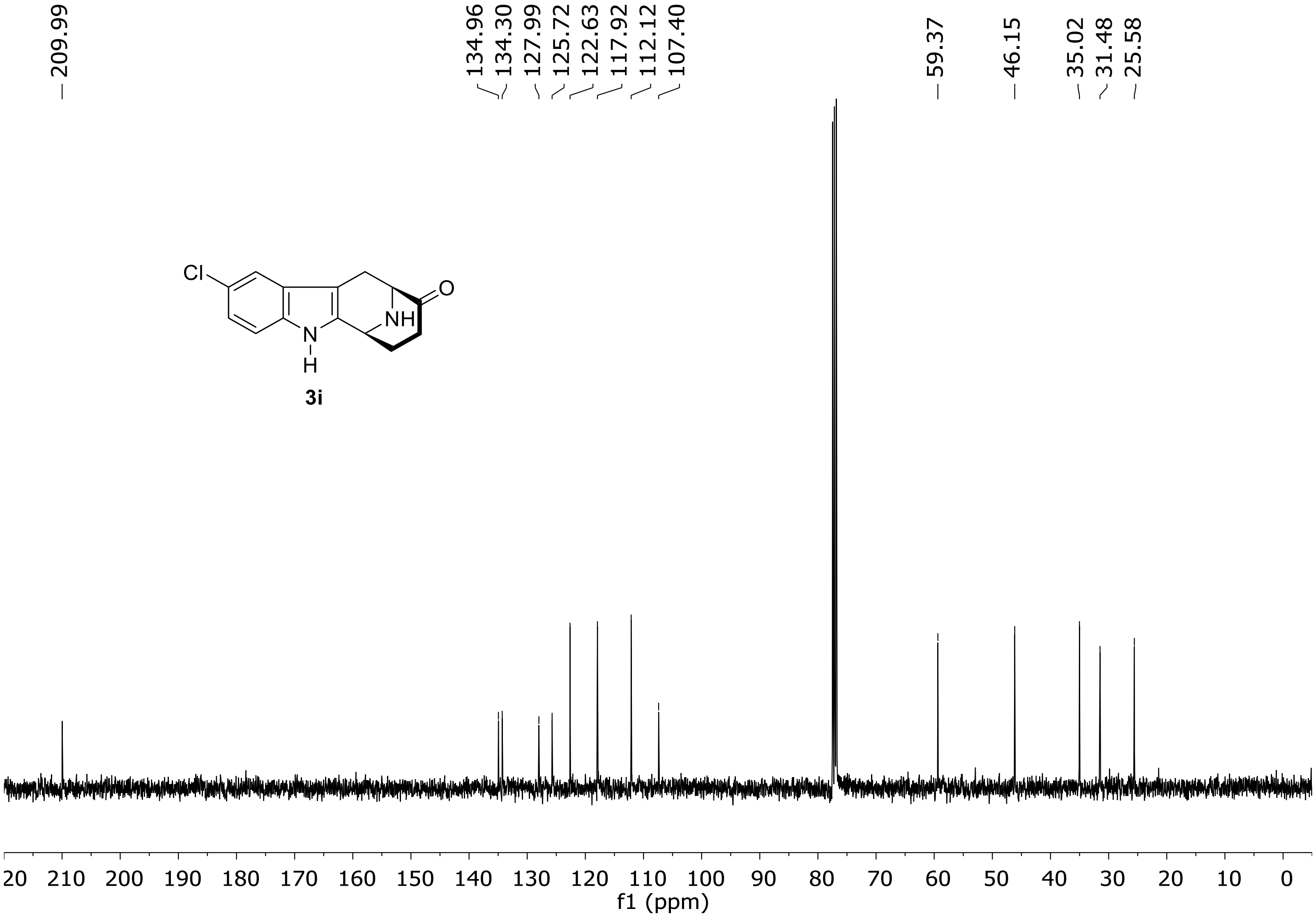
209.99
134.96
134.30
127.99
125.72
122.63
117.92
112.12
107.40
59.37
46.15
35.02
31.48
25.58
Cl
NH
O
N
H
3i
20 210 200 190 180 170 160 150 140 130 120 110 100 90 80 70 60 50 40 30 20 10 0
f1 (ppm)

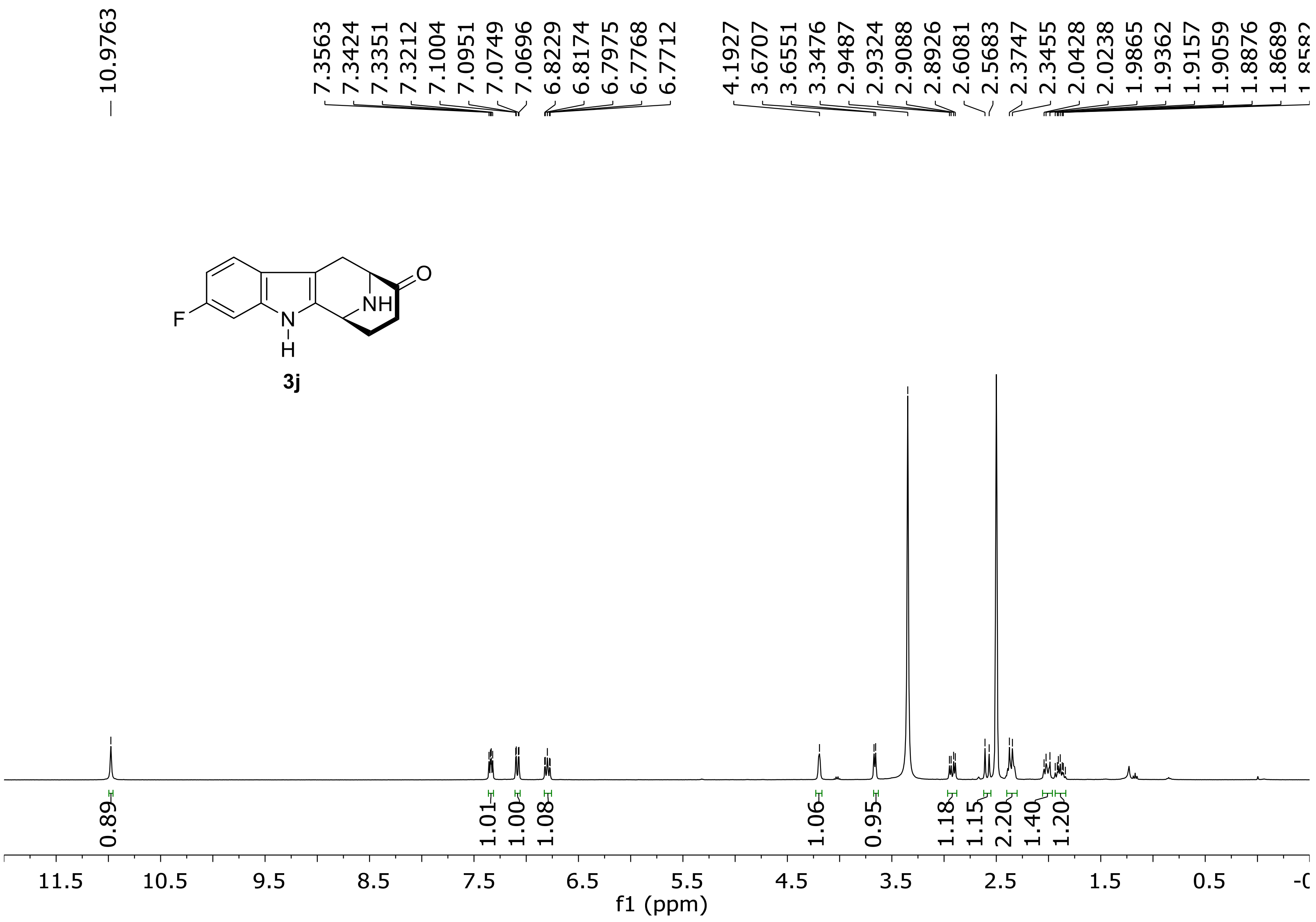
10.9763
7.3563
7.3424
7.3351
7.3212
7.1004
7.0951
7.0749
7.0696
6.8229
6.8174
6.7975
6.7768
6.7712
4.1927
3.6707
3.6551
3.3476
2.9487
2.9324
2.9088
2.8926
2.6081
2.5683
2.3747
2.3455
2.0428
2.0238
1.9865
1.9362
1.9157
1.9059
1.8876
1.8689
F
NH
N
H
O
3j
0.89
1.01
1.00
1.08
1.06
0.95
1.18
1.15
2.20
1.40
1.20
11.5
10.5
9.5
8.5
7.5
6.5
5.5
4.5
3.5
2.5
1.5
0.5
f1 (ppm)

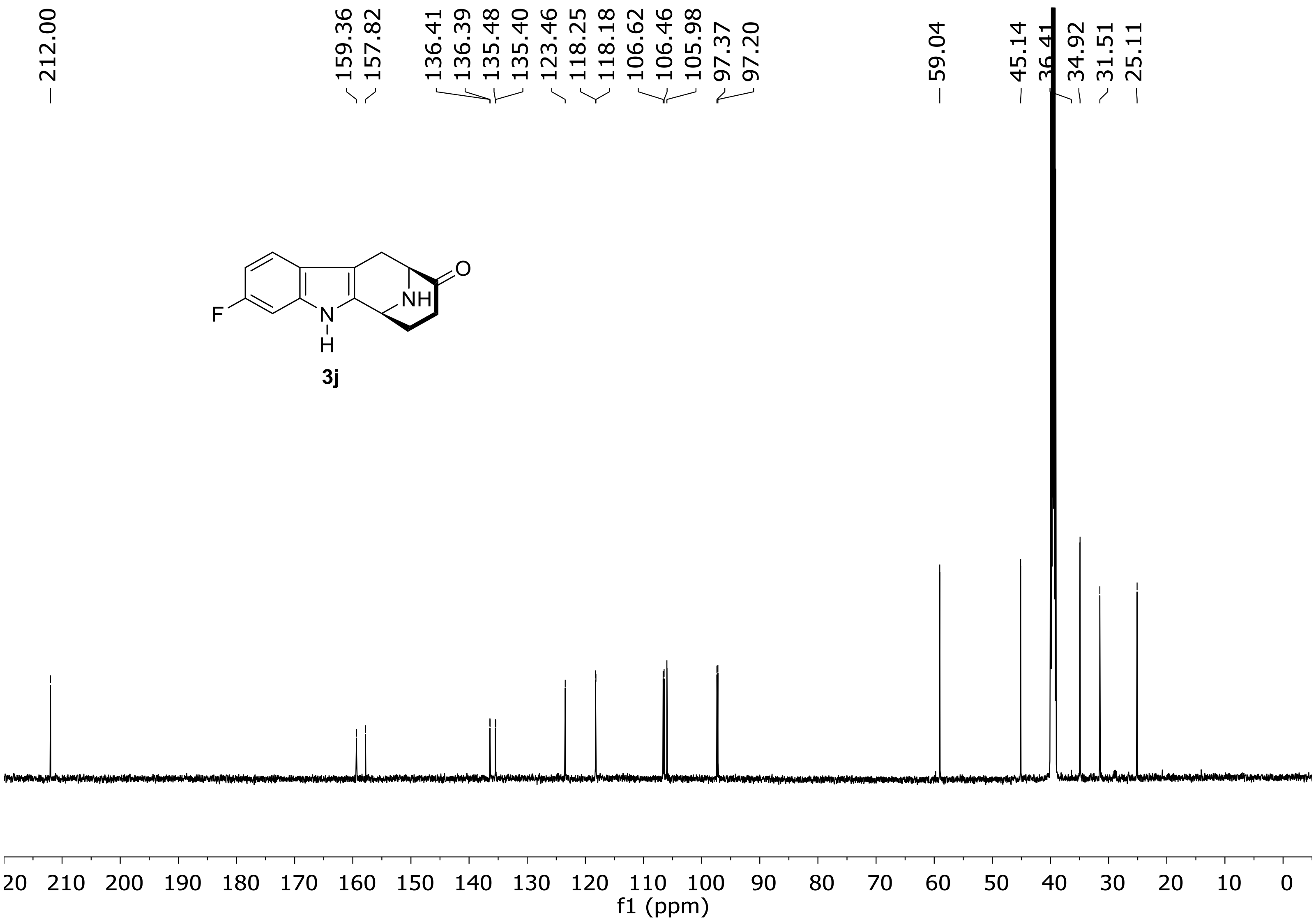
212.00
159.36
157.82
136.41
136.39
135.48
135.40
123.46
118.25
118.18
106.62
106.46
105.98
97.37
97.20
59.04
45.14
36.41
34.92
31.51
25.11
F
N
H
NH
O
3j
f1 (ppm)

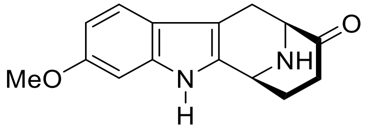
MeO
N
H
NH
O

3k

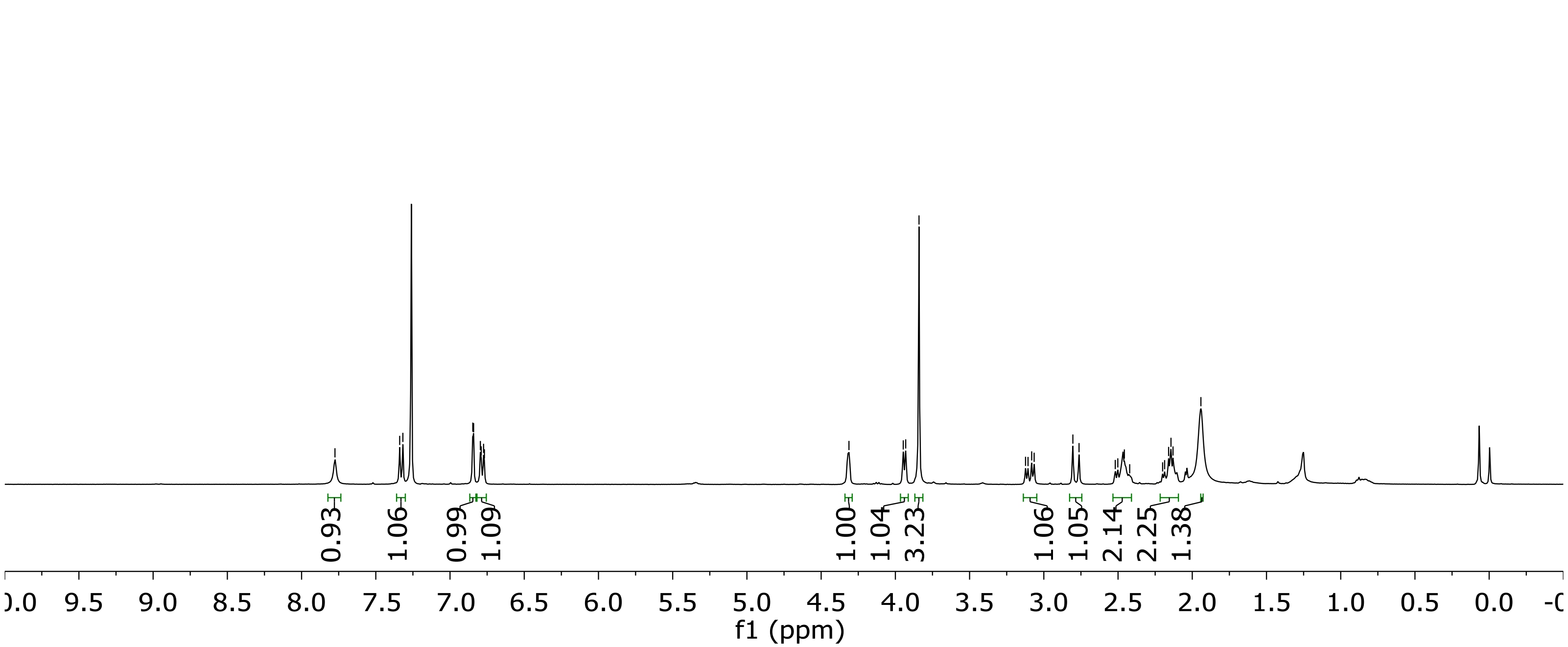
7.7750
7.3391
7.3176
6.8466
6.8411
6.7956
6.7898
6.7740
6.7684
4.3128
3.9470
3.9305
3.8408
3.1232
3.1065
3.0818
3.0651
2.8042
2.7629
2.5187
2.5017
2.4574
2.4216
2.1997
2.1862
2.1595
2.1433
2.1298
1.9419
0.93
1.06
0.99
1.09
1.00
1.04
3.23
1.06
1.05
2.14
2.25
1.38
9.5
9.0
8.5
8.0
7.5
7.0
6.5
6.0
5.5
5.0
4.5
4.0
3.5
3.0
2.5
2.0
1.5
1.0
0.5
0.0
f1 (ppm)

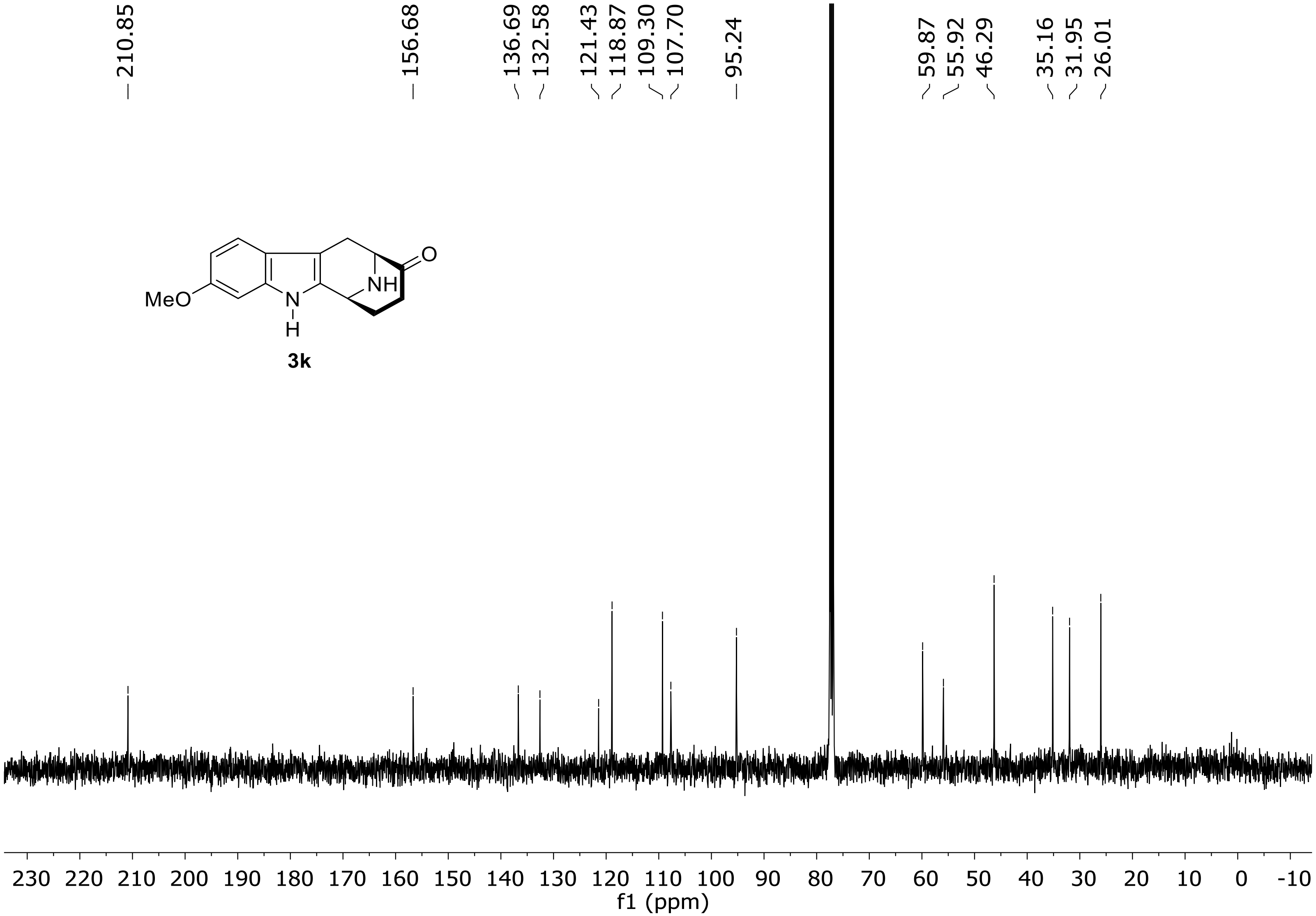
210.85
156.68
136.69
132.58
121.43
118.87
109.30
107.70
95.24
59.87
55.92
46.29
35.16
31.95
26.01
MeO
N
H
NH
O
3k
230 220 210 200 190 180 170 160 150 140 130 120 110 100 90 80 70 60 50 40 30 20 10 0 -10
f1 (ppm)

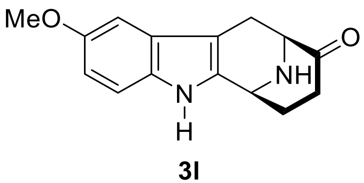

3l

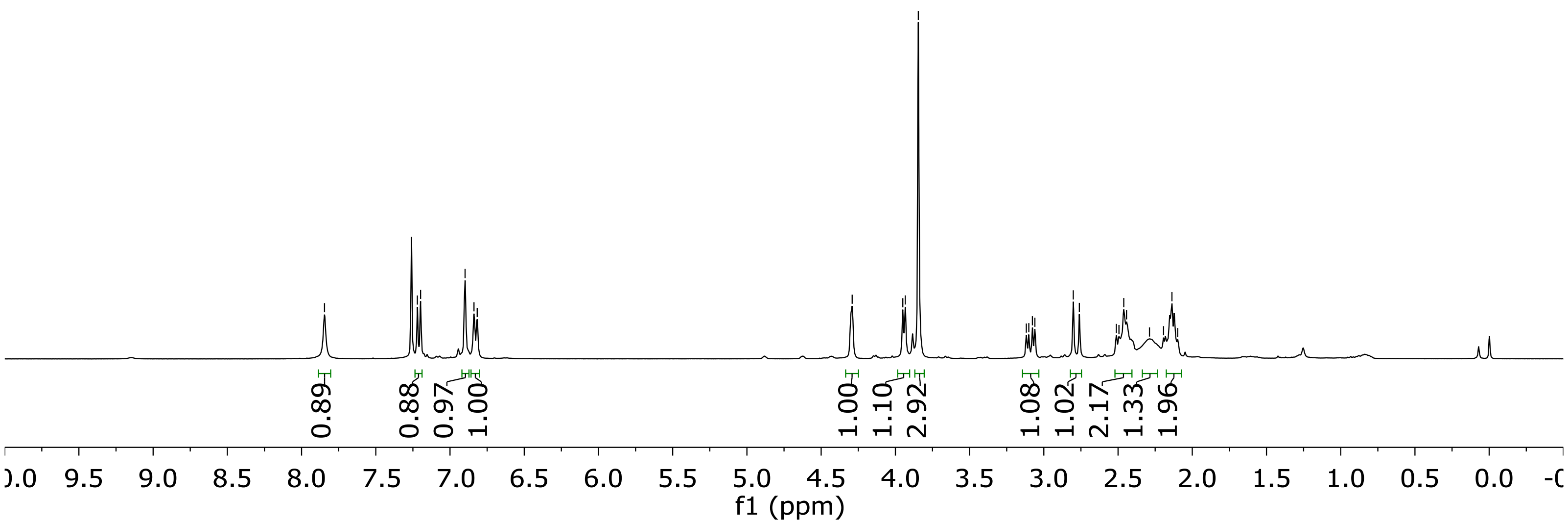
7.8454
7.2200
7.1982
6.8986
6.8385
6.8169
4.2910
3.9499
3.9335
3.8454
3.1179
3.1012
3.0770
3.0603
2.8016
2.7607
2.5115
2.4940
2.4614
2.4428
2.2882
2.1933
2.1370
2.0982
0.89
0.88
0.97
1.00
1.00
1.10
2.92
1.08
1.02
2.17
1.33
1.96
f1 (ppm)

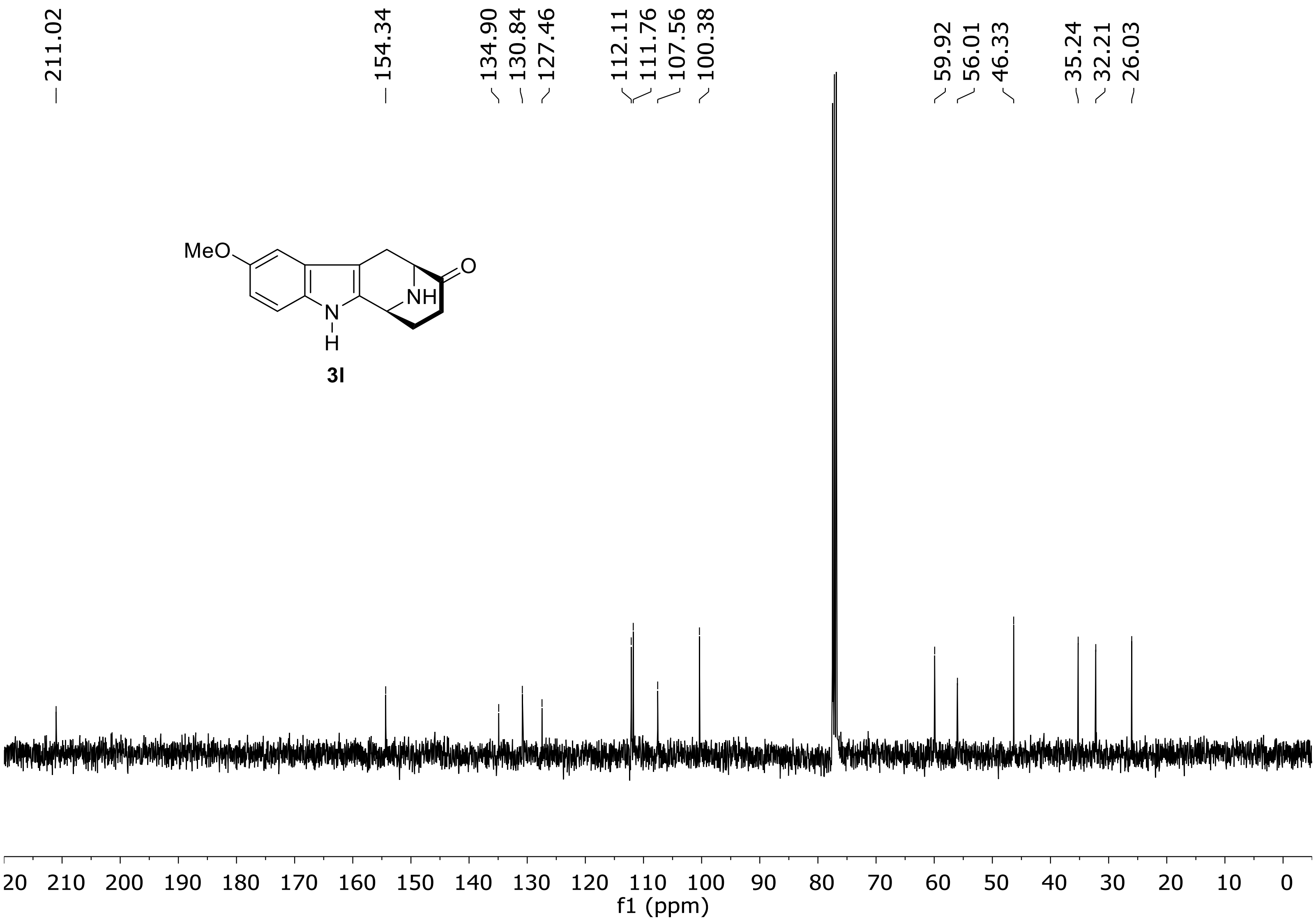
211.02
154.34
134.90
130.84
127.46
112.11
111.76
107.56
100.38
59.92
56.01
46.33
35.24
32.21
26.03
MeO
O
NH
N
H
3l
20 210 200 190 180 170 160 150 140 130 120 110 100 90 80 70 60 50 40 30 20 10 0
f1 (ppm)

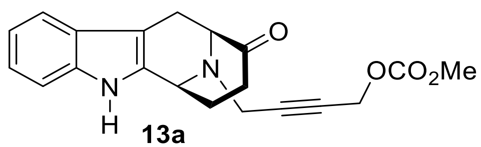
O
N
N
H
OCO2Me

13a

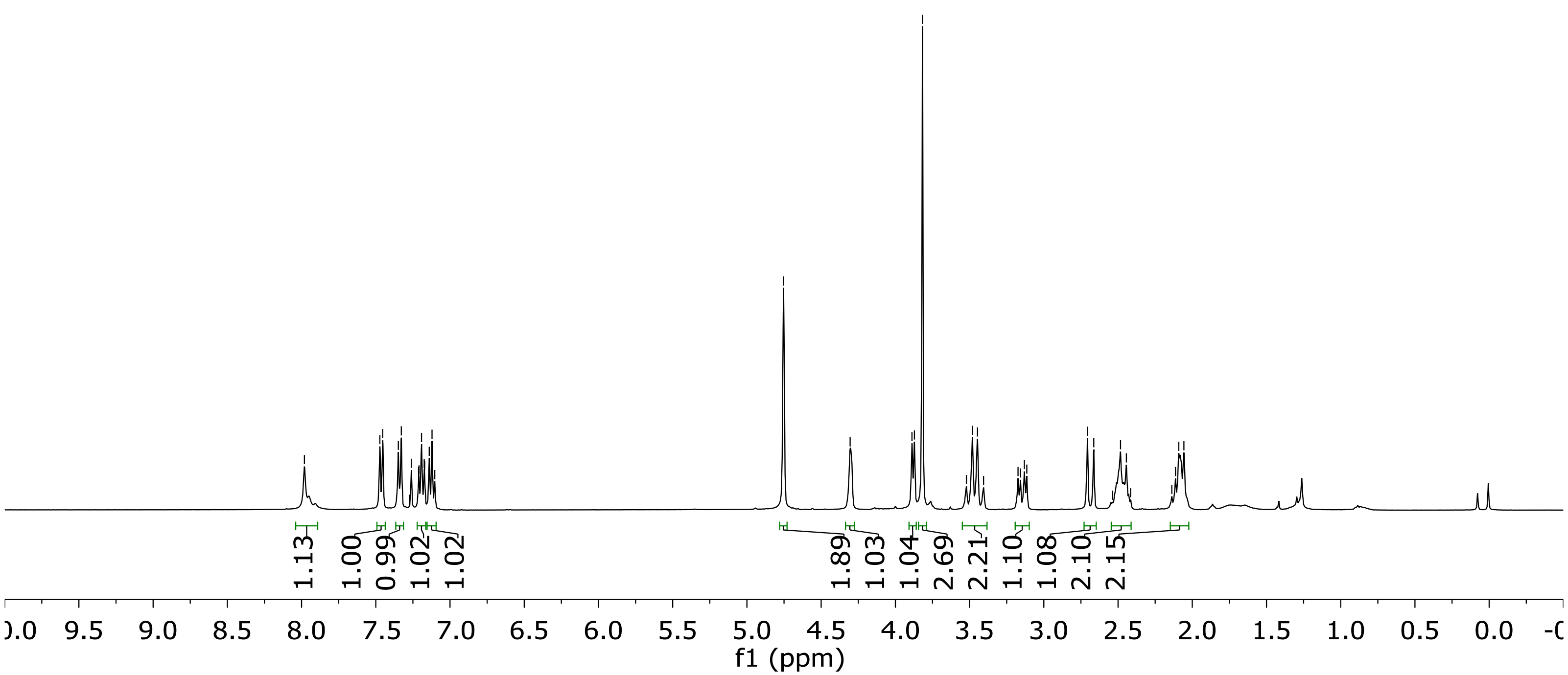
7.9808
7.4726
7.4532
7.3482
7.3282
7.2730
7.2605
7.2118
7.2087
7.1920
7.1741
7.1708
7.1401
7.1213
7.1029
4.7533
4.3047
3.8883
3.8717
3.8175
3.5215
3.4808
3.4468
3.4059
3.1738
3.1572
3.1311
3.1147
2.7061
2.6635
2.5363
2.4832
2.4439
2.4157
2.1377
2.1131
2.0909
2.0564
1.13
1.00
0.99
1.02
1.02
1.89
1.03
1.04
2.69
2.21
1.10
1.08
2.10
2.15
f1 (ppm)

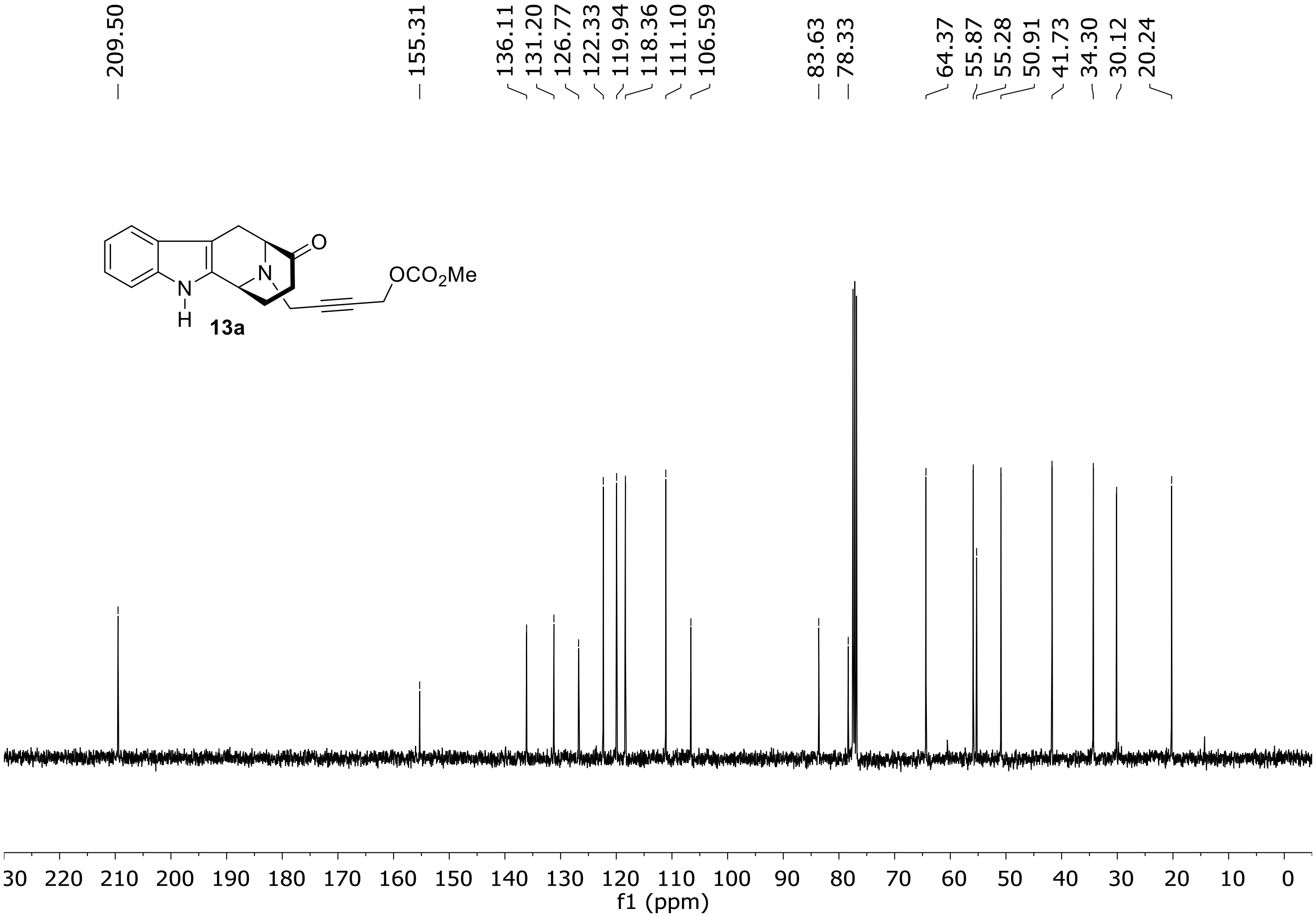
209.50
155.31
136.11
131.20
126.77
122.33
119.94
118.36
111.10
106.59
83.63
78.33
64.37
55.87
55.28
50.91
41.73
34.30
30.12
20.24
O
N
N
H
OCO2Me
13a
30
220
210
200
190
180
170
160
150
140
130
120
110
100
90
80
70
60
50
40
30
20
10
0
f1 (ppm)

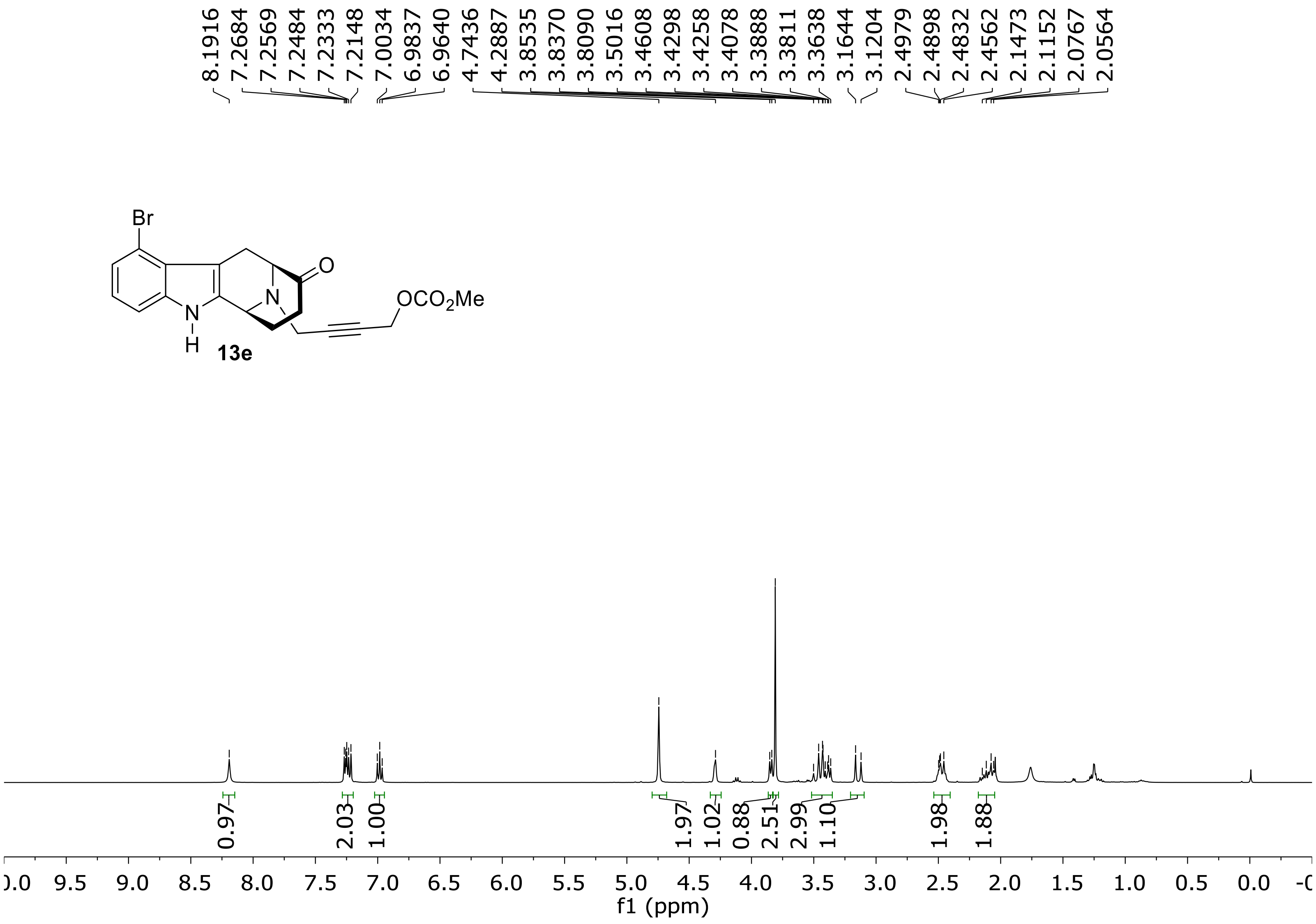
8.1916
7.2684
7.2569
7.2484
7.2333
7.2148
7.0034
6.9837
6.9640
4.7436
4.2887
3.8535
3.8370
3.8090
3.5016
3.4608
3.4298
3.4258
3.4078
3.3888
3.3811
3.3638
3.1644
3.1204
2.4979
2.4898
2.4832
2.4562
2.1473
2.1152
2.0767
2.0564
Br
O
N
H
N
OCO2Me
13e
0.97
2.03
1.00
1.97
1.02
0.88
2.51
2.99
1.10
1.98
1.88
f1 (ppm)

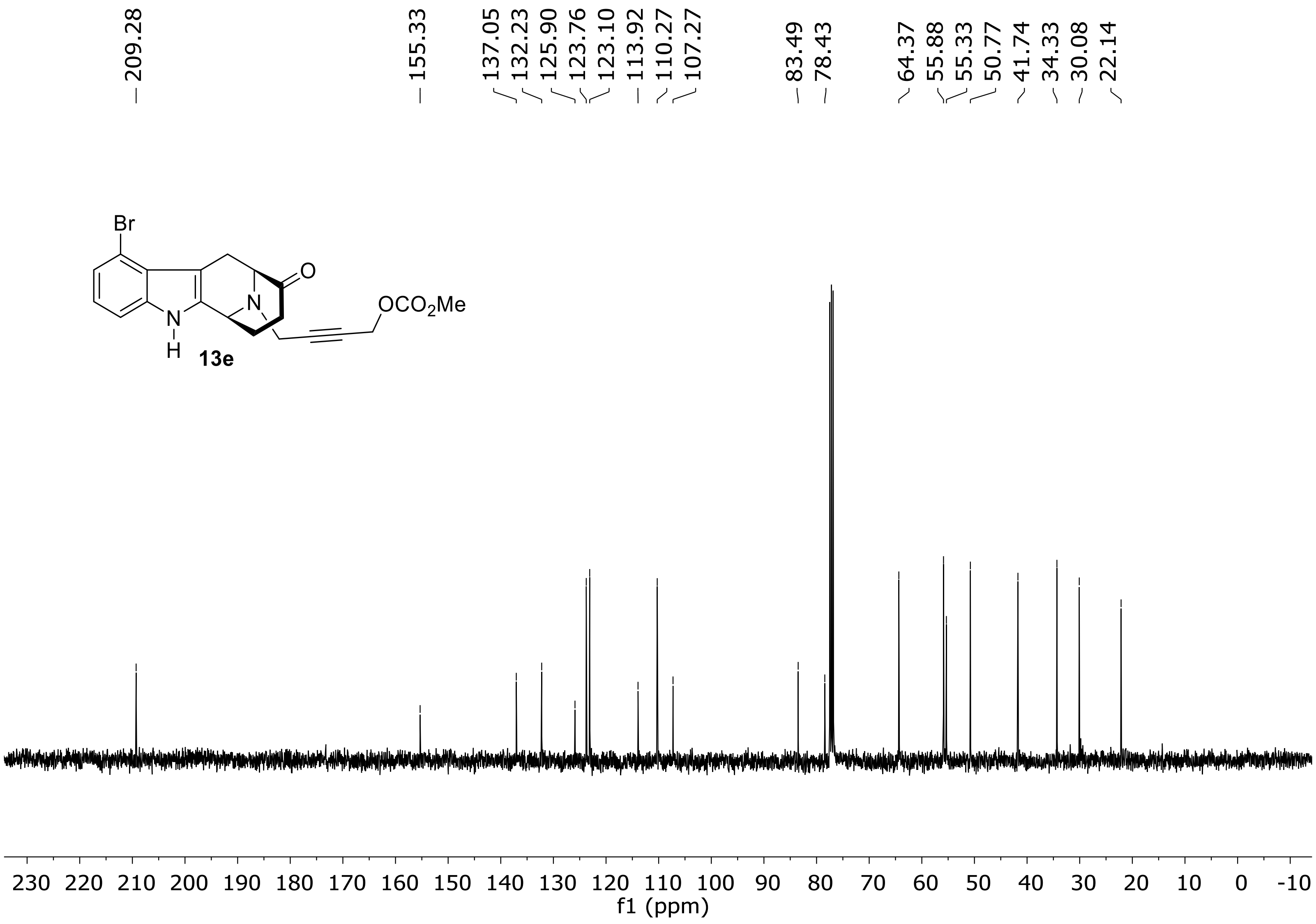
209.28
155.33
137.05
132.23
125.90
123.76
123.10
113.92
110.27
107.27
83.49
78.43
64.37
55.88
55.33
50.77
41.74
34.33
30.08
22.14
Br
O
N
N
H
OCO2Me
13e
230 220 210 200 190 180 170 160 150 140 130 120 110 100 90 80 70 60 50 40 30 20 10 0 -10
f1 (ppm)

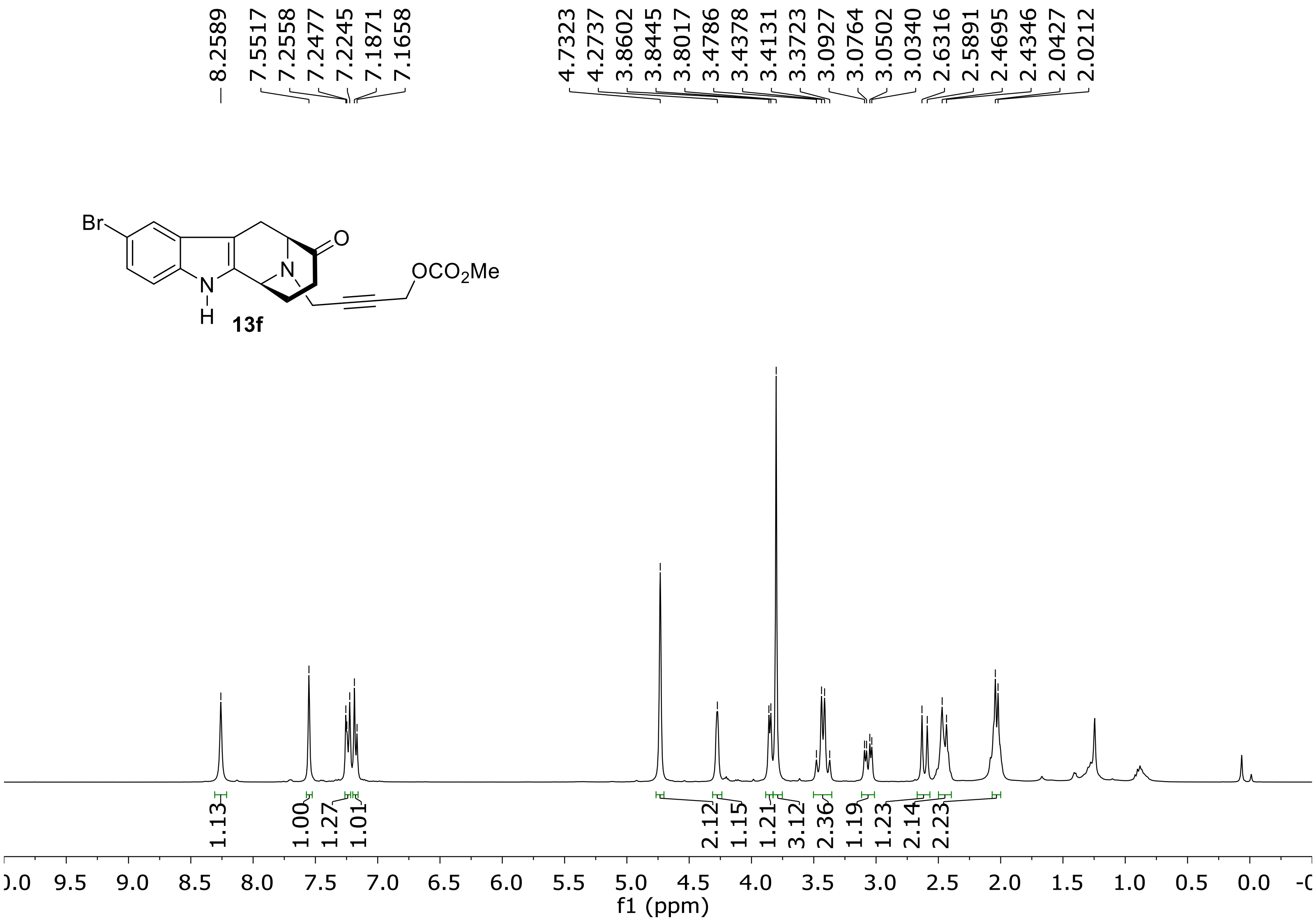

8.2589
7.5517
7.2558
7.2477
7.2245
7.1871
7.1658
4.7323
4.2737
3.8602
3.8445
3.8017
3.4786
3.4378
3.4131
3.3723
3.0927
3.0764
3.0502
3.0340
2.6316
2.5891
2.4695
2.4346
2.0427
2.0212
Br
O
N
OCO2Me
N
H
13f
1.13
1.00
1.27
1.01
2.12
1.15
1.21
3.12
2.36
1.19
1.23
2.14
2.23
f1 (ppm)

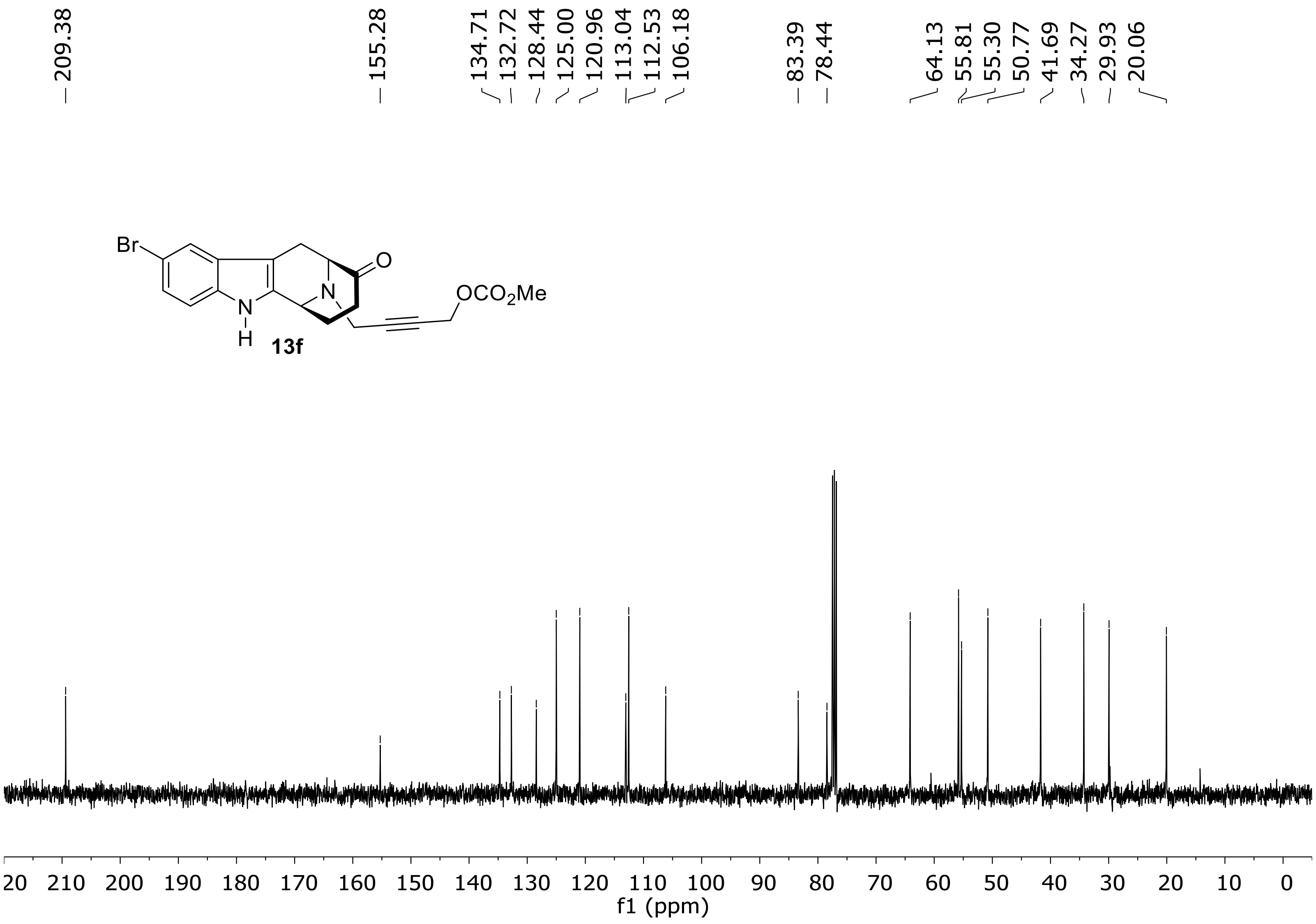
209.38
155.28
134.71
132.72
128.44
125.00
120.96
113.04
112.53
106.18
83.39
78.44
64.13
55.81
55.30
50.77
41.69
34.27
29.93
20.06
Br
O
N
N
H
OCO2Me
13f
20 210 200 190 180 170 160 150 140 130 120 110 100 90 80 70 60 50 40 30 20 10 0
f1 (ppm)

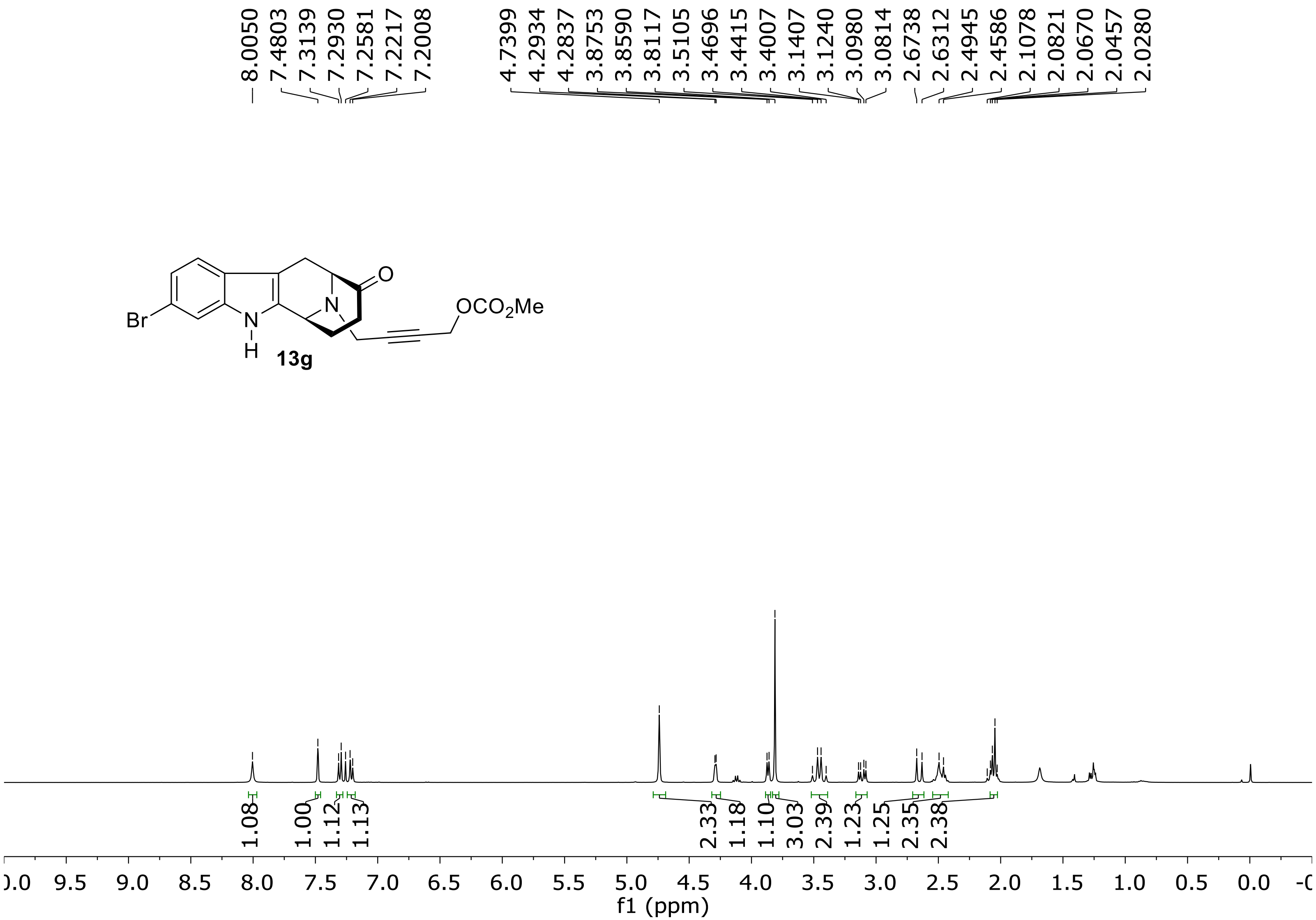
8.0050
7.4803
7.3139
7.2930
7.2581
7.2217
7.2008
4.7399
4.2934
4.2837
3.8753
3.8590
3.8117
3.5105
3.4696
3.4415
3.4007
3.1407
3.1240
3.0980
3.0814
2.6738
2.6312
2.4945
2.4586
2.1078
2.0821
2.0670
2.0457
2.0280
Br
N
H
N
O
OCO2Me
1.08
1.00
1.12
1.13
2.33
1.18
1.10
3.03
2.39
1.23
1.25
2.35
2.38
9.5
9.0
8.5
8.0
7.5
7.0
6.5
6.0
5.5
5.0
4.5
4.0
3.5
3.0
2.5
2.0
1.5
1.0
0.5
0.0
f1 (ppm)
13g

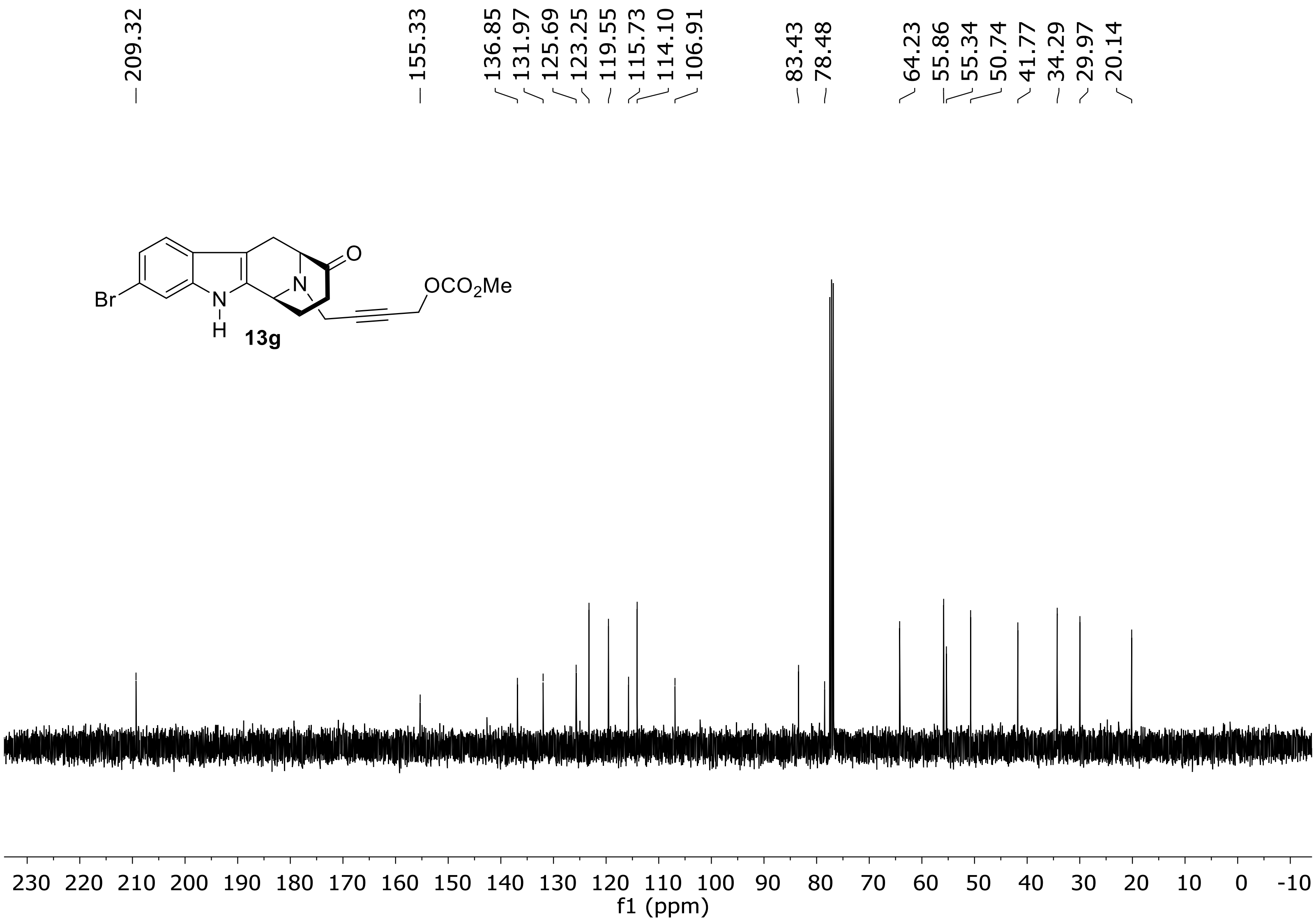
209.32
155.33
136.85
131.97
125.69
123.25
119.55
115.73
114.10
106.91
83.43
78.48
64.23
55.86
55.34
50.74
41.77
34.29
29.97
20.14
Br
N
H
N
O
OCO2Me
13g
230 220 210 200 190 180 170 160 150 140 130 120 110 100 90 80 70 60 50 40 30 20 10 0 -10
f1 (ppm)

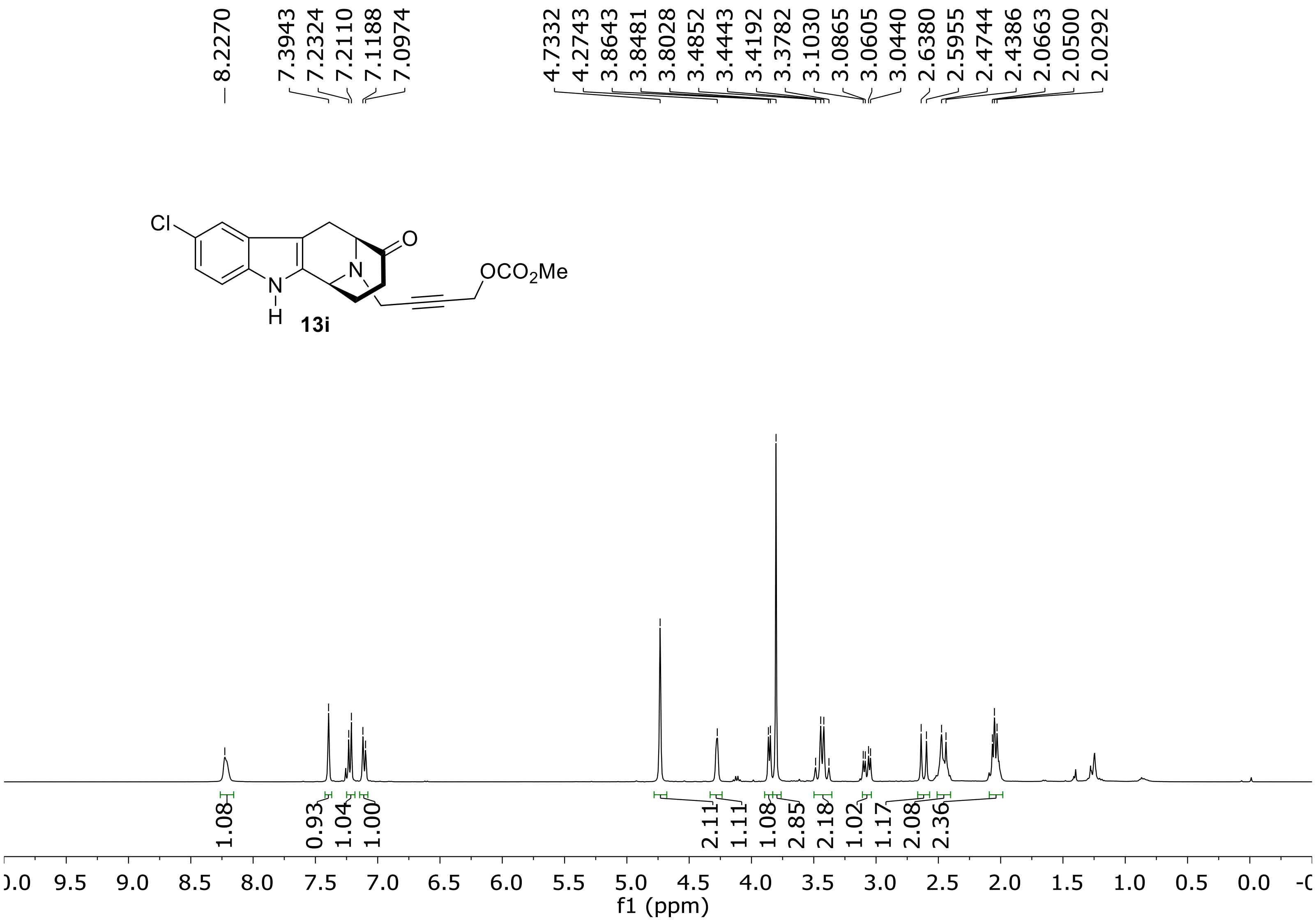
8.2270
7.3943
7.2324
7.2110
7.1188
7.0974
4.7332
4.2743
3.8643
3.8481
3.8028
3.4852
3.4443
3.4192
3.3782
3.1030
3.0865
3.0605
3.0440
2.6380
2.5955
2.4744
2.4386
2.0663
2.0500
2.0292
Cl
O
N
H
OCO2Me
13i
1.08
0.93
1.04
1.00
2.11
1.11
1.08
2.85
2.18
1.02
1.17
2.08
2.36
9.5
9.0
8.5
8.0
7.5
7.0
6.5
6.0
5.5
5.0
4.5
4.0
3.5
3.0
2.5
2.0
1.5
1.0
0.5
0.0
f1 (ppm)

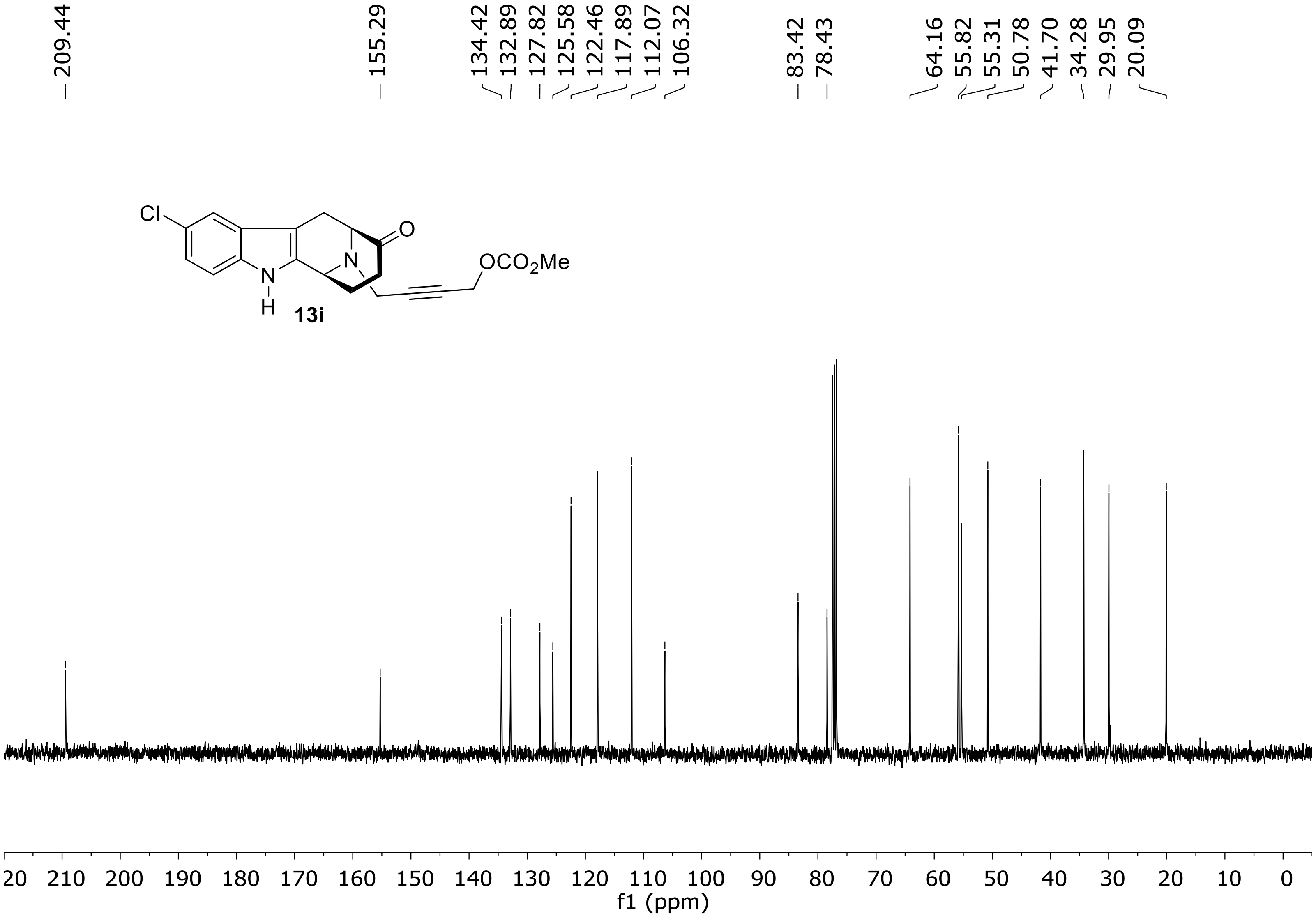
209.44
155.29
134.42
132.89
127.82
125.58
122.46
117.89
112.07
106.32
83.42
78.43
64.16
55.82
55.31
50.78
41.70
34.28
29.95
20.09
Cl
O
N
OCO2Me
N
H
13i
20 210 200 190 180 170 160 150 140 130 120 110 100 90 80 70 60 50 40 30 20 10 0
f1 (ppm)

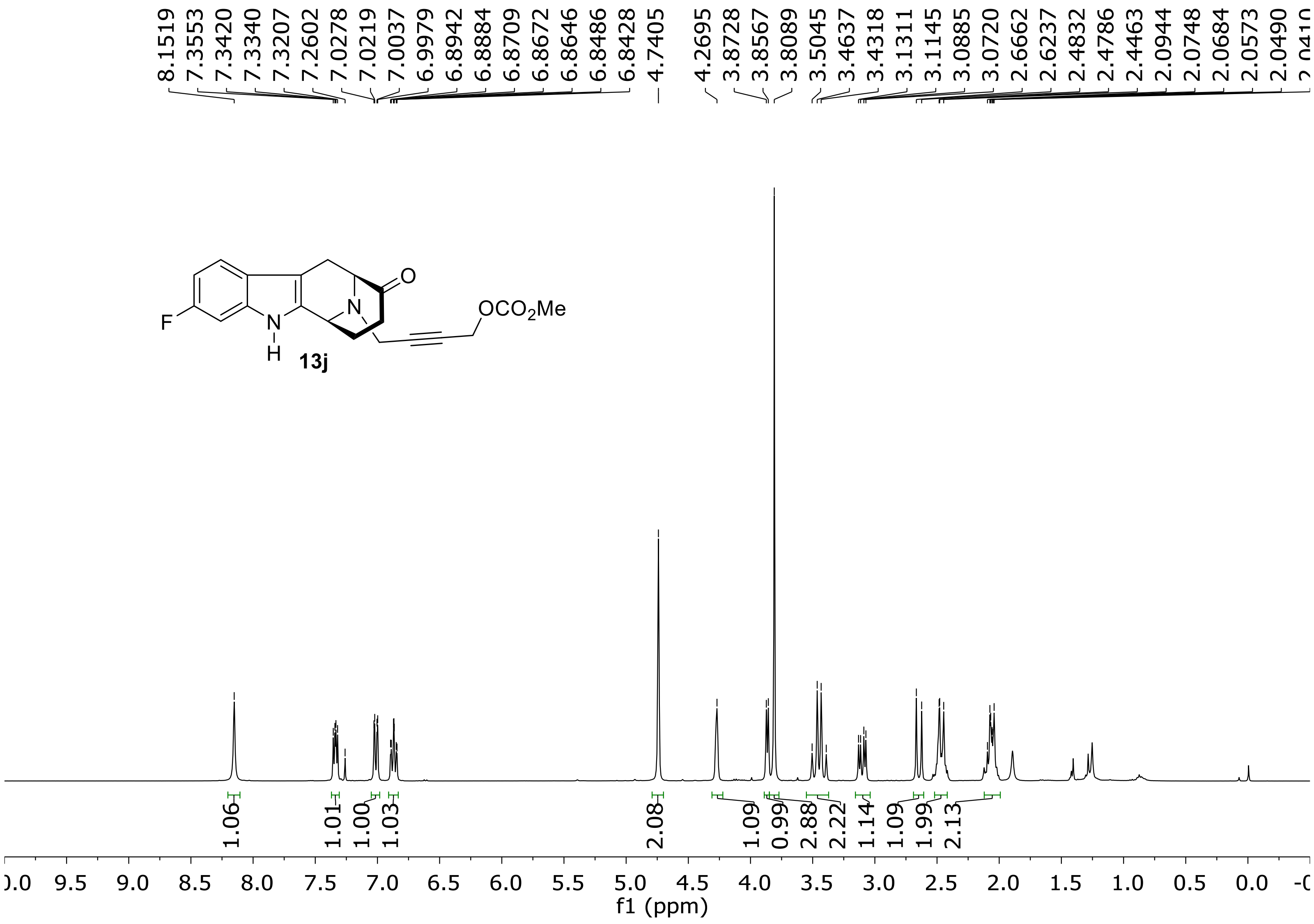
8.1519
7.3553
7.3420
7.3340
7.3207
7.2602
7.0278
7.0219
7.0037
6.9979
6.8942
6.8884
6.8709
6.8672
6.8646
6.8486
6.8428
4.7405
4.2695
3.8728
3.8567
3.8089
3.5045
3.4637
3.4318
3.1311
3.1145
3.0885
3.0720
2.6662
2.6237
2.4832
2.4786
2.4463
2.0944
2.0748
2.0684
2.0573
2.0490
F
N
H
N
O
OCO2Me
13j
1.06
1.01
1.00
1.03
2.08
1.09
0.99
2.88
2.22
1.14
1.09
1.99
2.13
9.5
9.0
8.5
8.0
7.5
7.0
6.5
6.0
5.5
5.0
4.5
4.0
3.5
3.0
2.5
2.0
1.5
1.0
0.5
0.0
f1 (ppm)

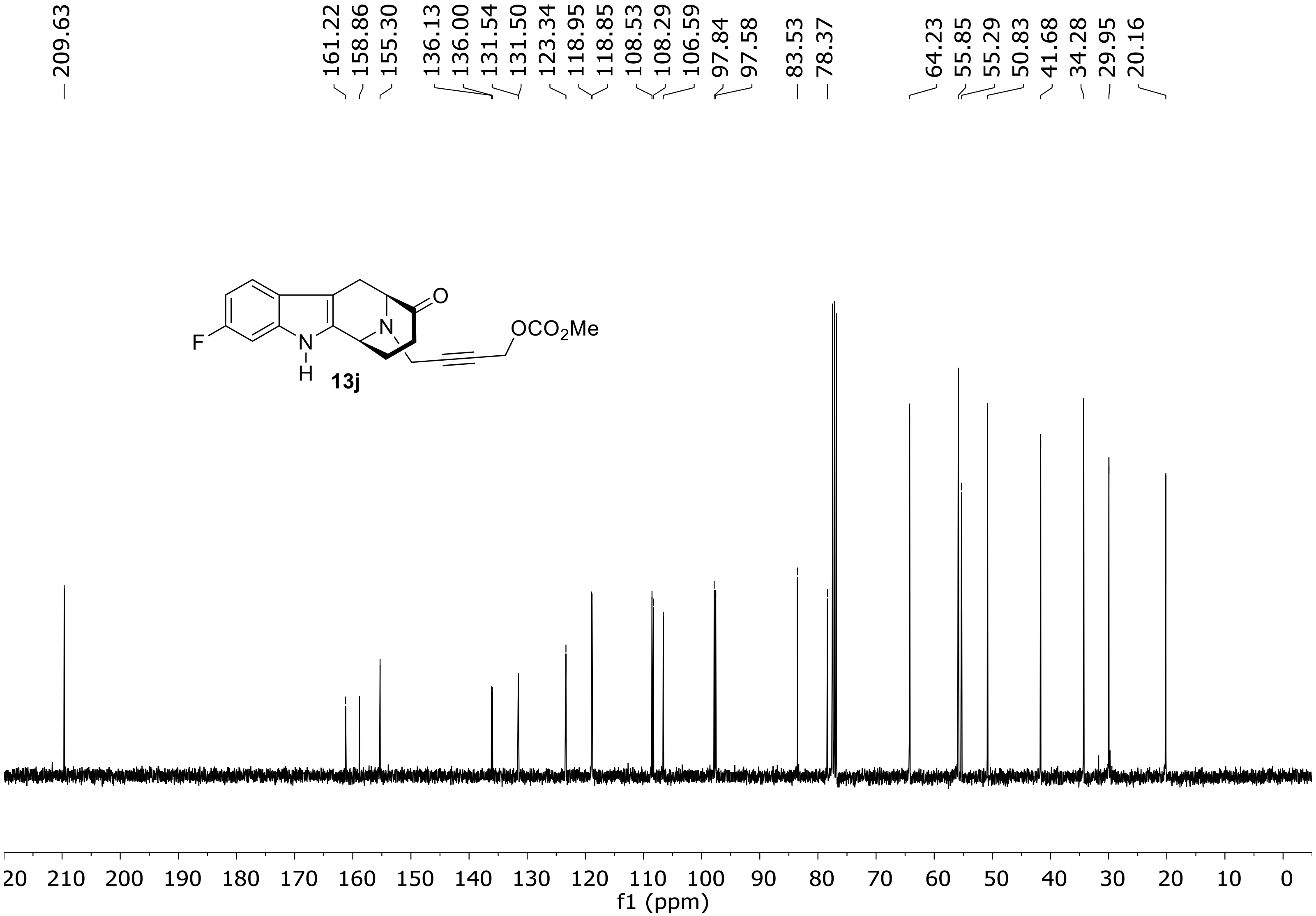
209.63
161.22
158.86
155.30
136.13
136.00
131.54
131.50
123.34
118.95
118.85
108.53
108.29
106.59
97.84
97.58
83.53
78.37
64.23
55.85
55.29
50.83
41.68
34.28
29.95
20.16
F
N
H
N
O
OCO2Me
13j
20 210 200 190 180 170 160 150 140 130 120 110 100 90 80 70 60 50 40 30 20 10 0
f1 (ppm)

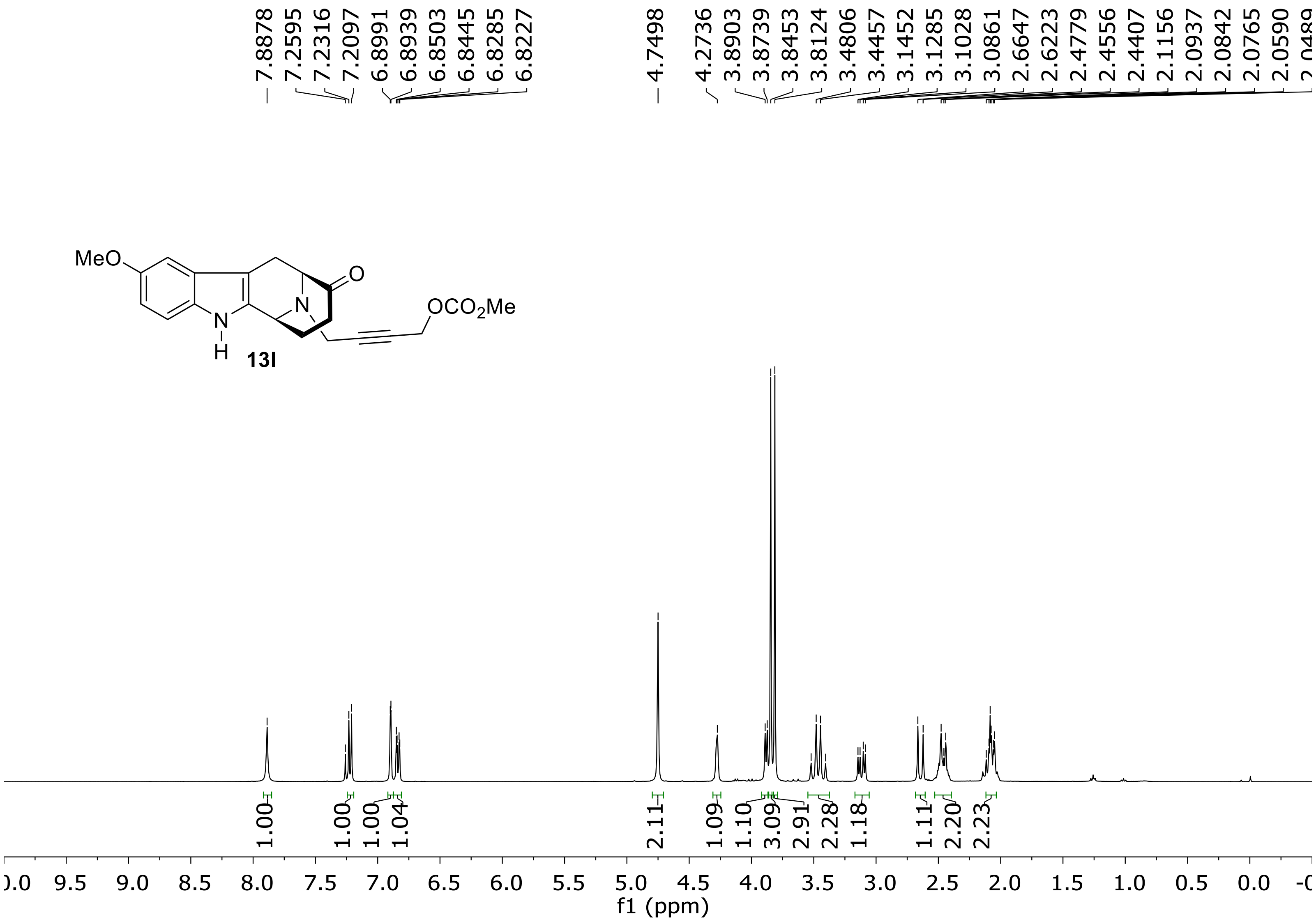
7.8878
7.2595
7.2316
7.2097
6.8991
6.8939
6.8503
6.8445
6.8285
6.8227
4.7498
4.2736
3.8903
3.8739
3.8453
3.8124
3.4806
3.4457
3.1452
3.1285
3.1028
3.0861
2.6647
2.6223
2.4779
2.4556
2.4407
2.1156
2.0937
2.0842
2.0765
2.0590
MeO
N
H
N
O
OCO2Me
13l
1.00
1.00
1.00
1.04
2.11
1.09
1.10
3.09
2.91
2.28
1.18
1.11
2.20
2.23
9.5
9.0
8.5
8.0
7.5
7.0
6.5
6.0
5.5
5.0
4.5
4.0
3.5
3.0
2.5
2.0
1.5
1.0
0.5
0.0
f1 (ppm)

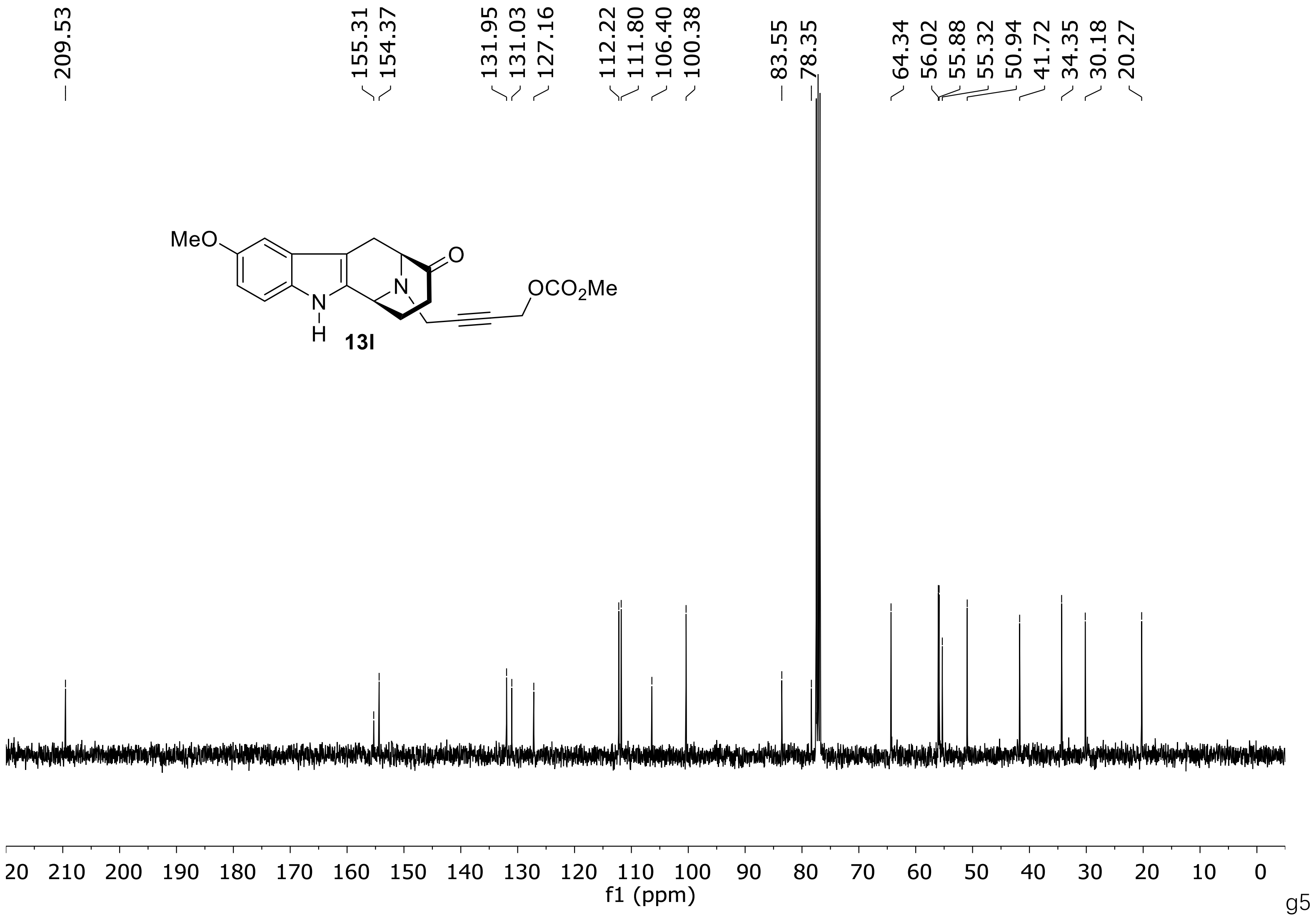
209.53
155.31
154.37
131.95
131.03
127.16
112.22
111.80
106.40
100.38
83.55
78.35
64.34
56.02
55.88
55.32
50.94
41.72
34.35
30.18
20.27
MeO
N
H
N
O
OCO2Me
13l
20 210 200 190 180 170 160 150 140 130 120 110 100 90 80 70 60 50 40 30 20 10 0
f1 (ppm)
g5

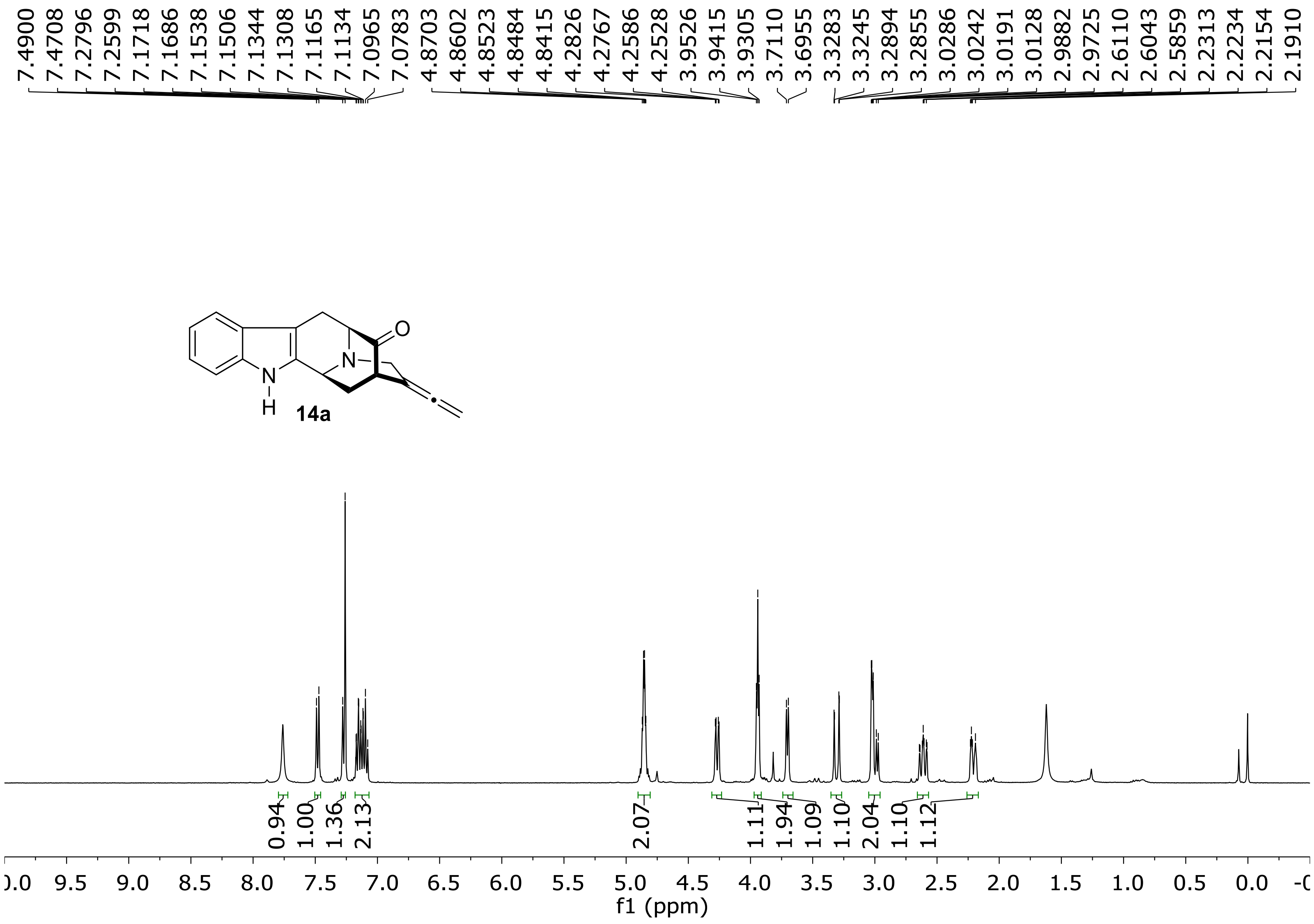
7.4900 7.4708 7.2796 7.2599 7.1718 7.1686 7.1538 7.1506 7.1344 7.1308 7.1165 7.1134 7.0965 7.0783 4.8703 4.8602 4.8523 4.8484 4.8415 4.2826 4.2767 4.2586 4.2528 3.9526 3.9415 3.9305 3.7110 3.6955 3.3283 3.3245 3.2894 3.2855 3.0286 3.0242 3.0191 3.0128 2.9882 2.9725 2.6110 2.6043 2.5859 2.2313 2.2234 2.2154 2.1910
O
N
N
H
0.94 1.00 1.36 2.13 2.07 1.11 1.94 1.09 1.10 2.04 1.10 1.12
9.5 9.0 8.5 8.0 7.5 7.0 6.5 6.0 5.5 5.0 4.5 4.0 3.5 3.0 2.5 2.0 1.5 1.0 0.5 0.0
f1 (ppm)

14a

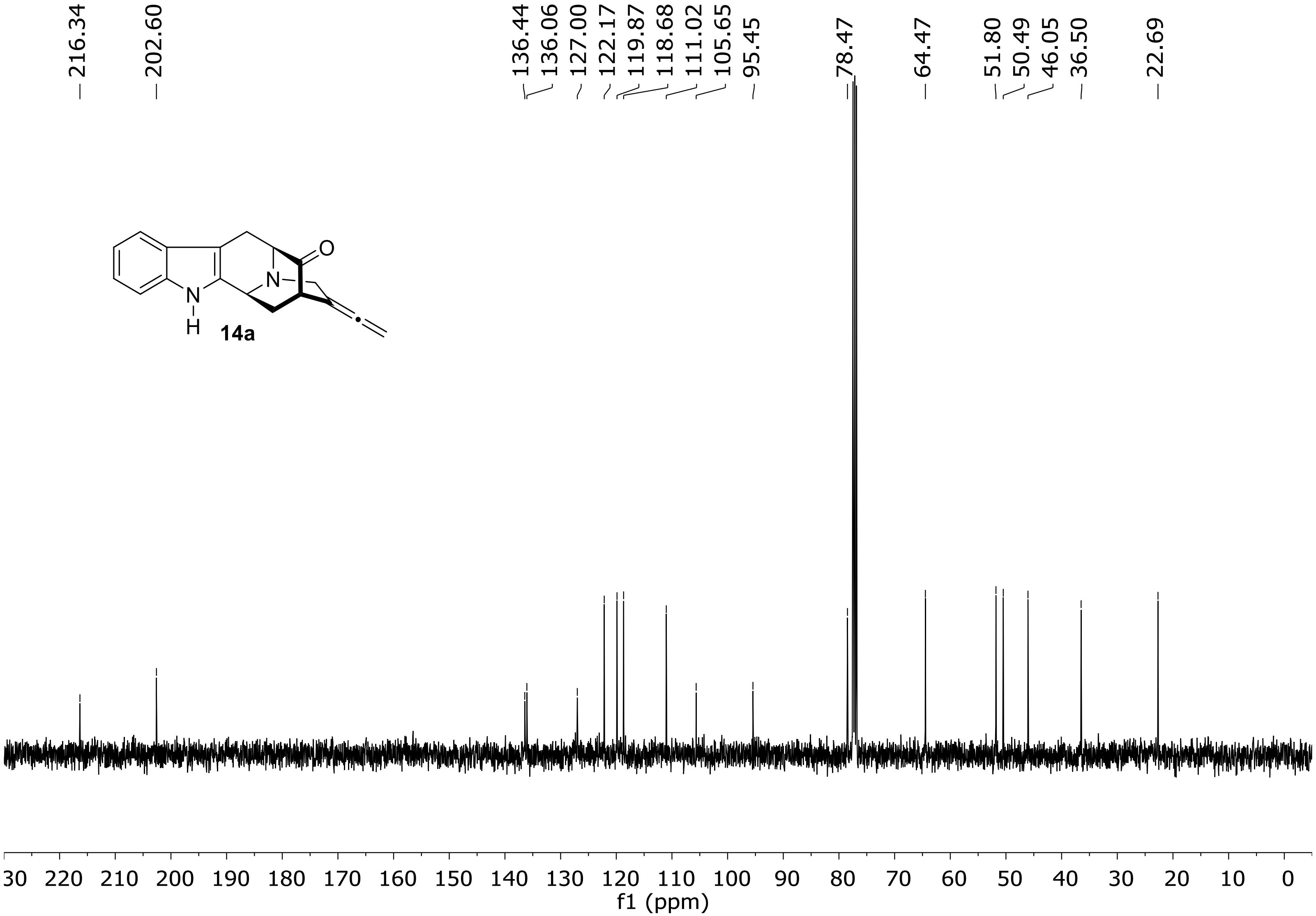
216.34
202.60
136.44
136.06
127.00
122.17
119.87
118.68
111.02
105.65
95.45
78.47
64.47
51.80
50.49
46.05
36.50
22.69
N
H
O
N
14a
30 220 210 200 190 180 170 160 150 140 130 120 110 100 90 80 70 60 50 40 30 20 10 0
f1 (ppm)

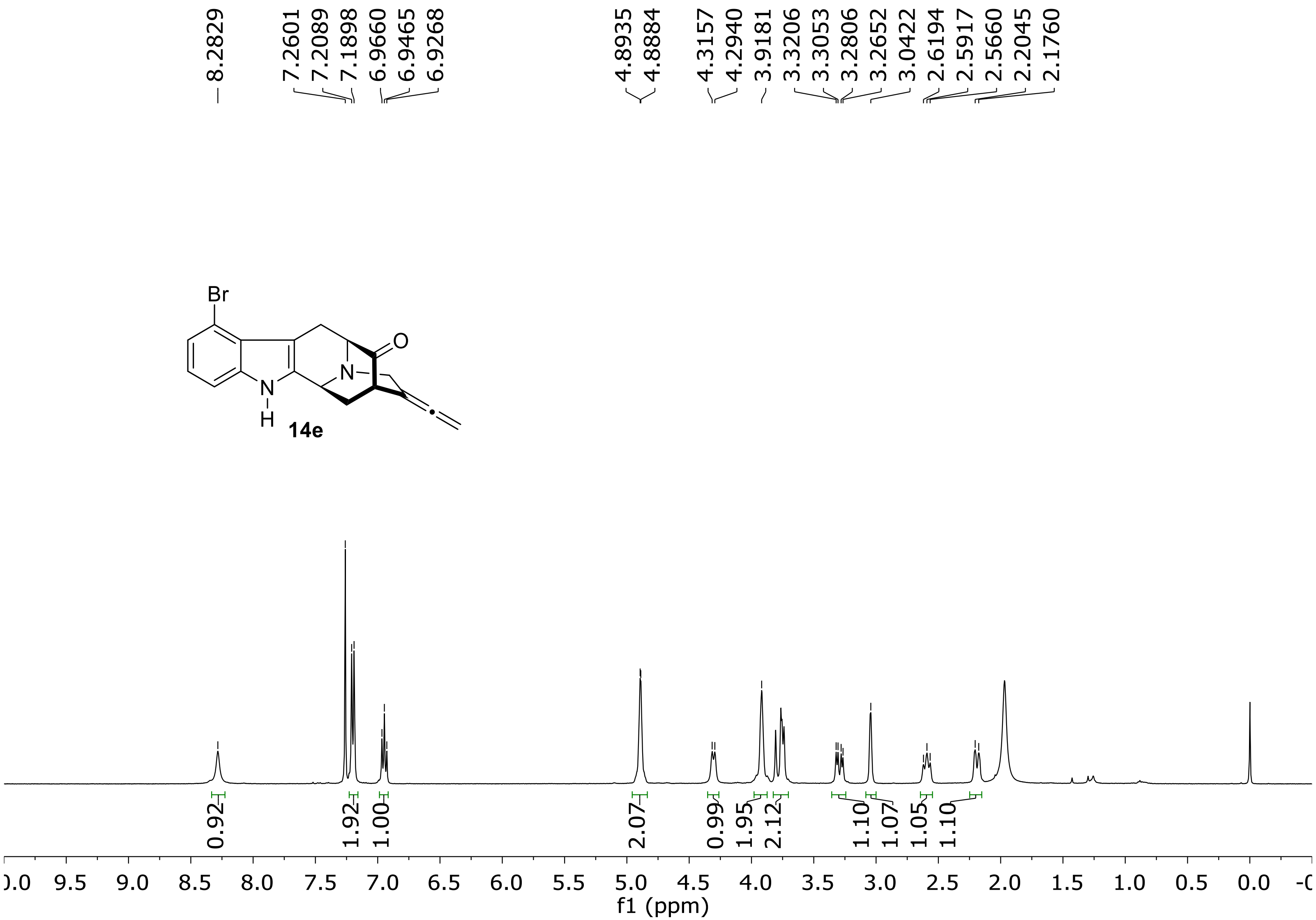

8.2829
7.2601
7.2089
7.1898
6.9660
6.9465
6.9268
4.8935
4.8884
4.3157
4.2940
3.9181
3.3206
3.3053
3.2806
3.2652
3.0422
2.6194
2.5917
2.5660
2.2045
2.1760
Br
O
N
N
H
14e
0.92
1.92
1.00
2.07
0.99
1.95
2.12
1.10
1.07
1.05
1.10
f1 (ppm)

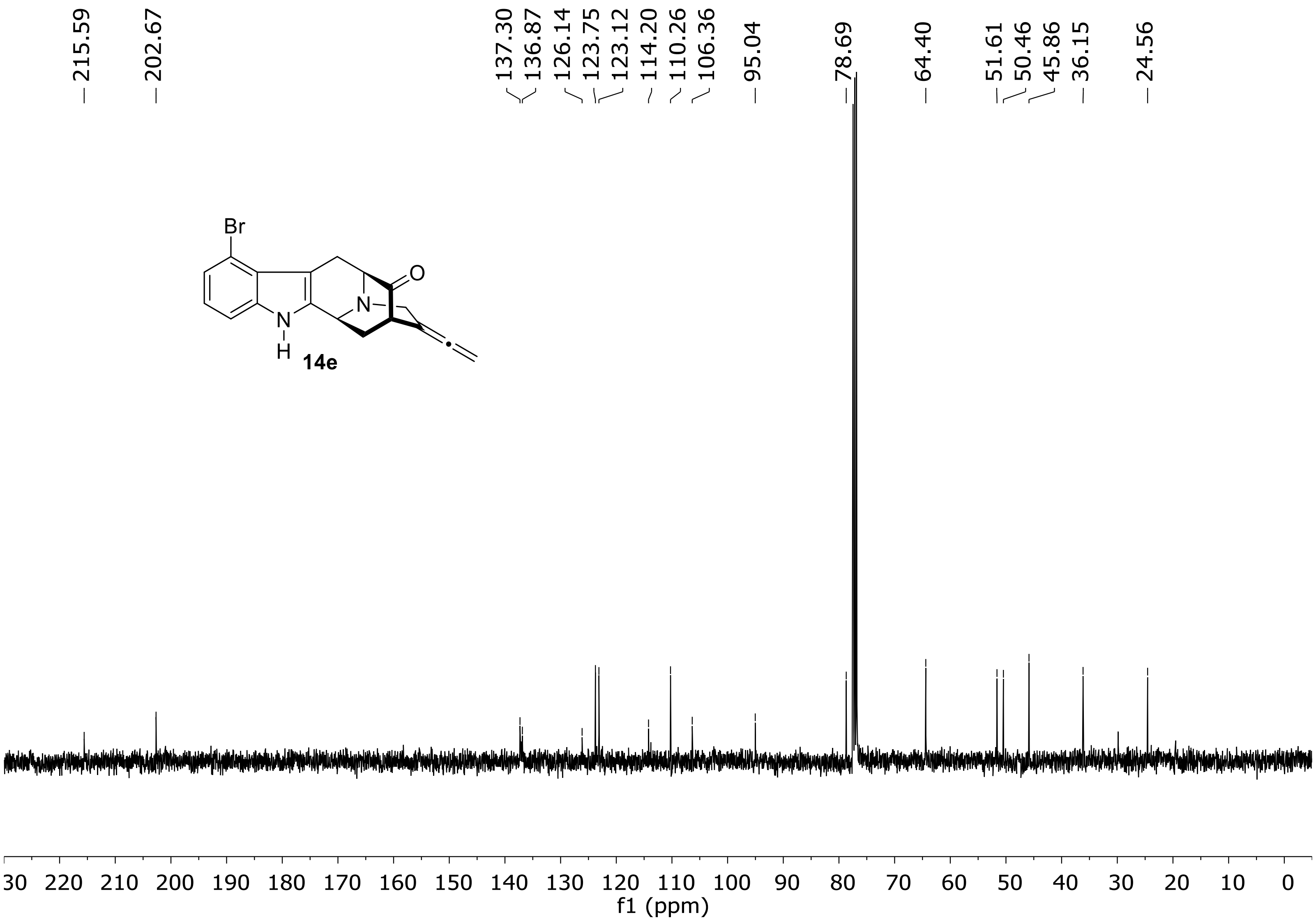
215.59
202.67
137.30
136.87
126.14
123.75
123.12
114.20
110.26
106.36
95.04
78.69
64.40
51.61
50.46
45.86
36.15
24.56
Br
O
N
N
H
14e
30 220 210 200 190 180 170 160 150 140 130 120 110 100 90 80 70 60 50 40 30 20 10 0
f1 (ppm)

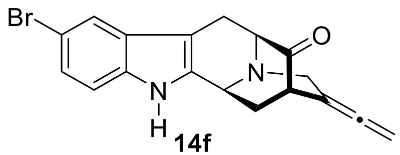

14f

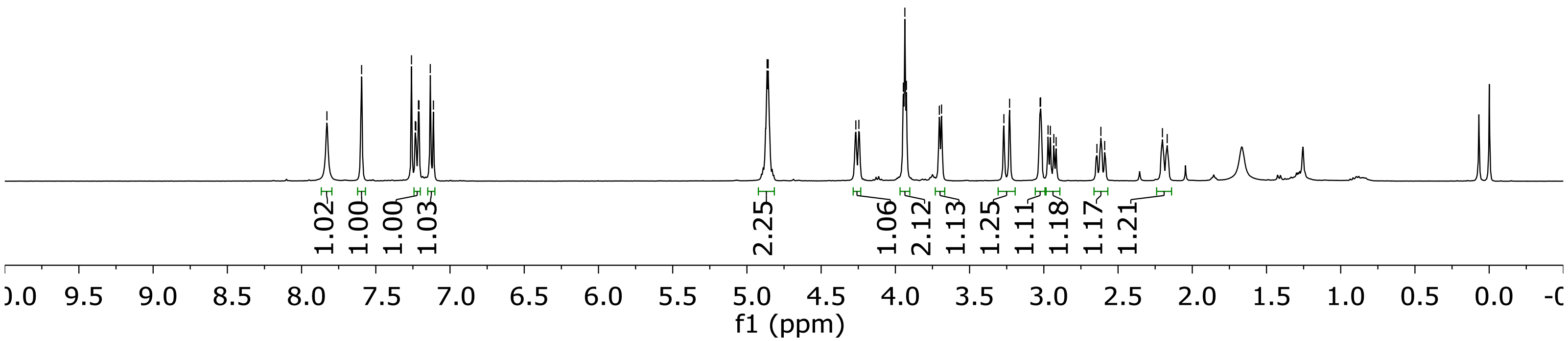
7.8292
7.5955
7.2596
7.2343
7.2303
7.2129
7.2089
7.1331
7.1117
4.8641
4.8568
4.2663
4.2459
3.9463
3.9361
3.9256
3.7039
3.6891
3.2691
3.2308
3.0249
3.0213
2.9716
2.9558
2.9326
2.9169
2.6423
2.6155
2.5899
2.2010
2.1690
1.02
1.00
1.00
1.03
2.25
1.06
2.12
1.13
1.25
1.11
1.18
1.17
1.21
f1 (ppm)

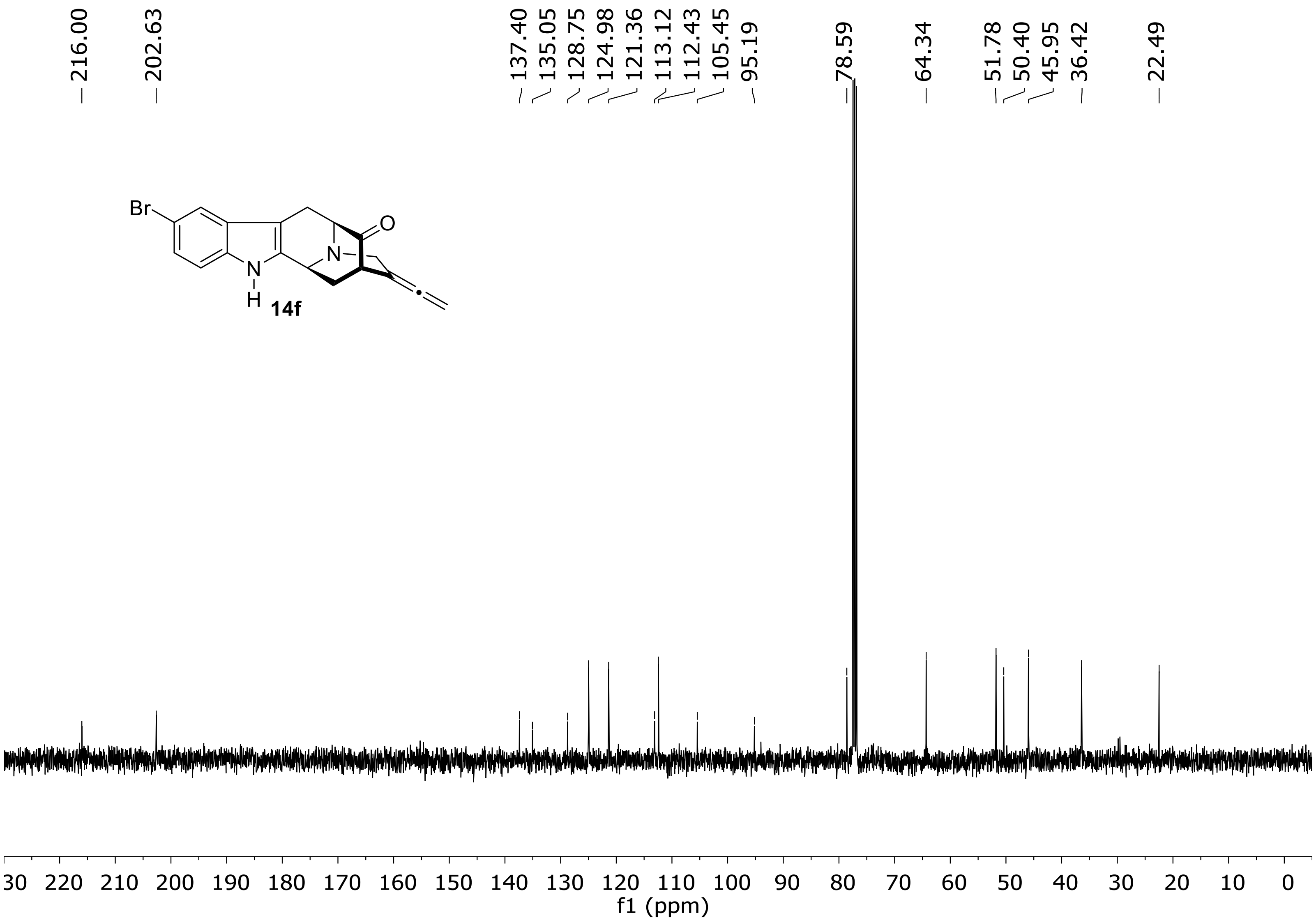
216.00
202.63
137.40
135.05
128.75
124.98
121.36
113.12
112.43
105.45
95.19
78.59
64.34
51.78
50.40
45.95
36.42
22.49
Br
O
N
N
H
14f
30 220 210 200 190 180 170 160 150 140 130 120 110 100 90 80 70 60 50 40 30 20 10 0
f1 (ppm)

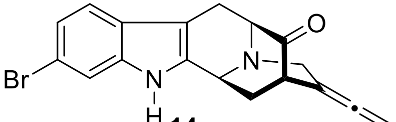
Br
N
H
N
O
14g

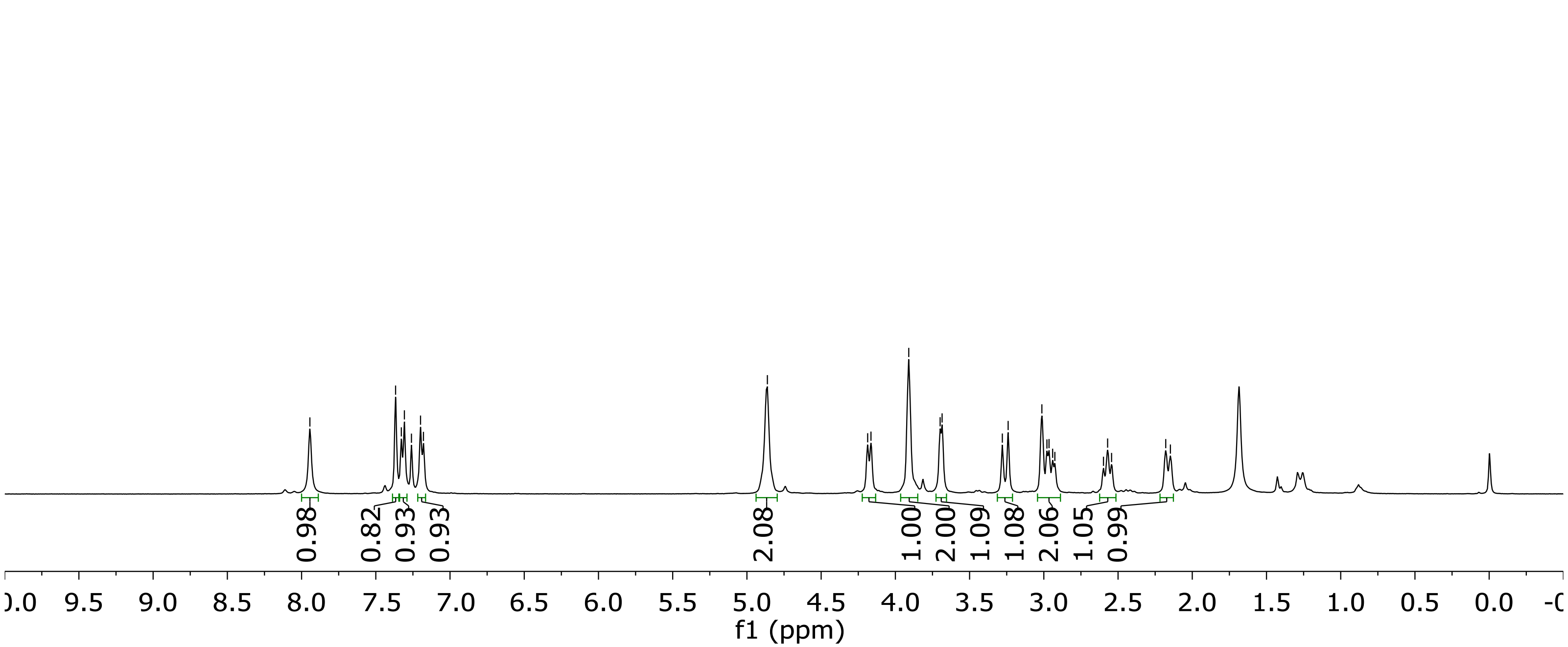
7.9441
7.3668
7.3280
7.3075
7.2598
7.1988
7.1790
4.8622
4.1867
4.1644
3.9099
3.6984
3.6852
3.2794
3.2404
3.0131
2.9792
2.9650
2.9407
2.9256
2.5982
2.5702
2.5436
2.1788
2.1473
0.98
0.82
0.93
0.93
2.08
1.00
2.00
1.09
1.08
2.06
1.05
0.99
0.0 9.5 9.0 8.5 8.0 7.5 7.0 6.5 6.0 5.5 5.0 4.5 4.0 3.5 3.0 2.5 2.0 1.5 1.0 0.5 0.0 -0
f1 (ppm)

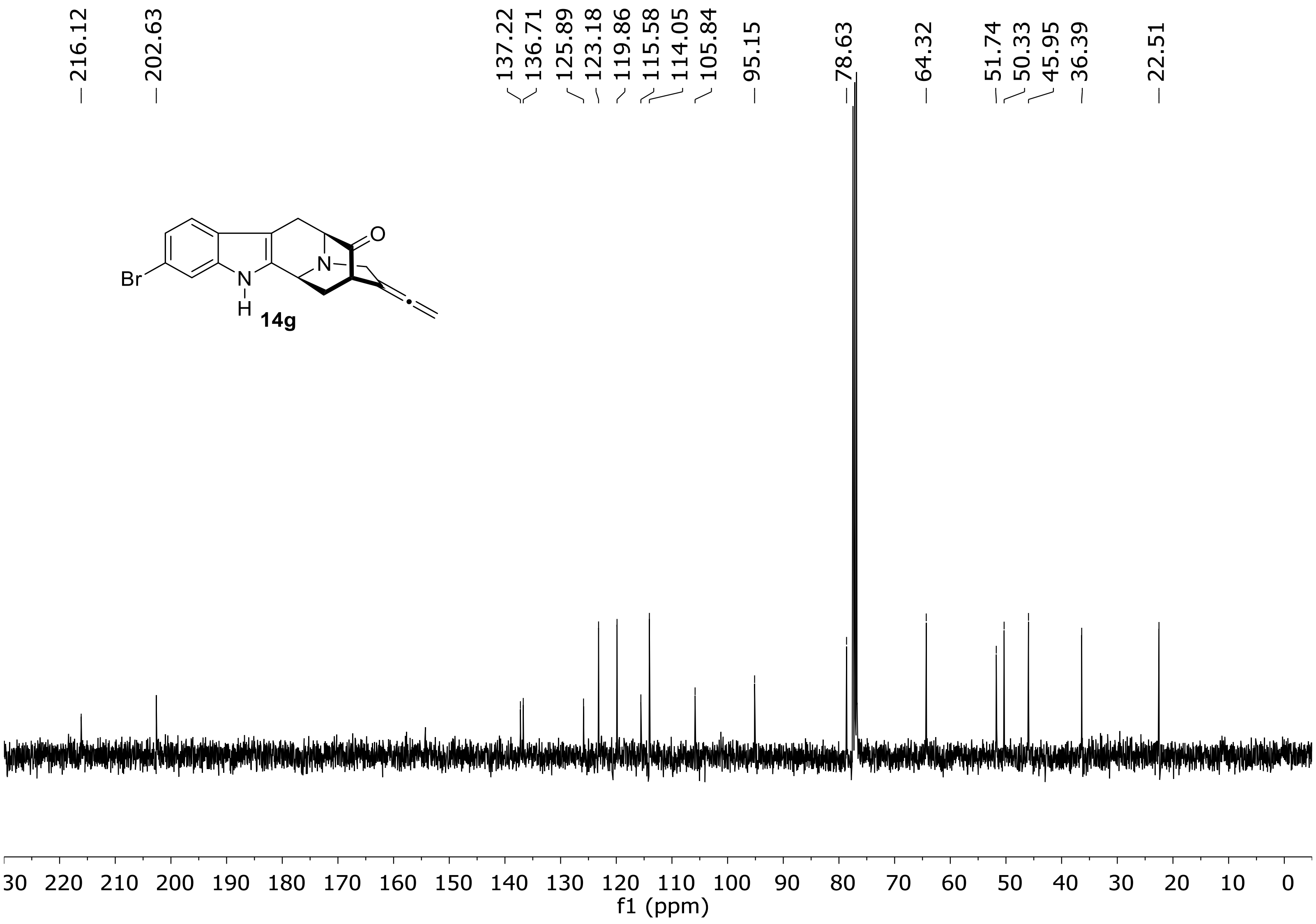
216.12
202.63
137.22
136.71
125.89
123.18
119.86
115.58
114.05
105.84
95.15
78.63
64.32
51.74
50.33
45.95
36.39
22.51
Br
N
H
N
O
14g
30 220 210 200 190 180 170 160 150 140 130 120 110 100 90 80 70 60 50 40 30 20 10 0
f1 (ppm)

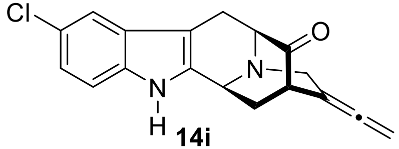

14i

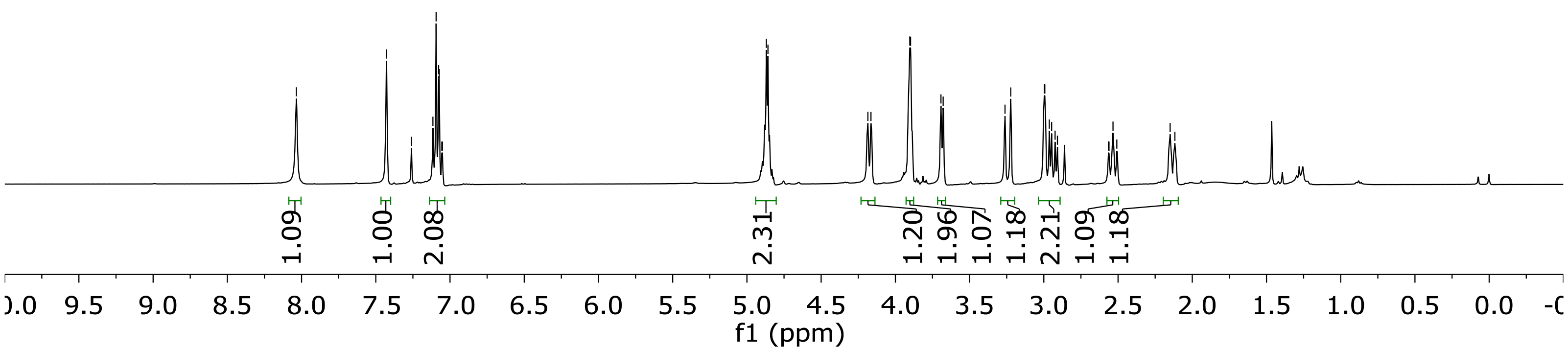
8.0351
7.4285
7.2597
7.1151
7.0938
7.0769
7.0728
7.0554
7.0513
4.8690
4.8583
4.1851
4.1644
3.9029
3.8977
3.6929
3.6781
3.2615
3.2229
2.9968
2.9932
2.9631
2.9473
2.9241
2.9084
2.5646
2.5610
2.5340
2.5084
2.1492
2.1171
1.09
1.00
2.08
2.31
1.20
1.96
1.07
1.18
2.21
1.09
1.18
f1 (ppm)

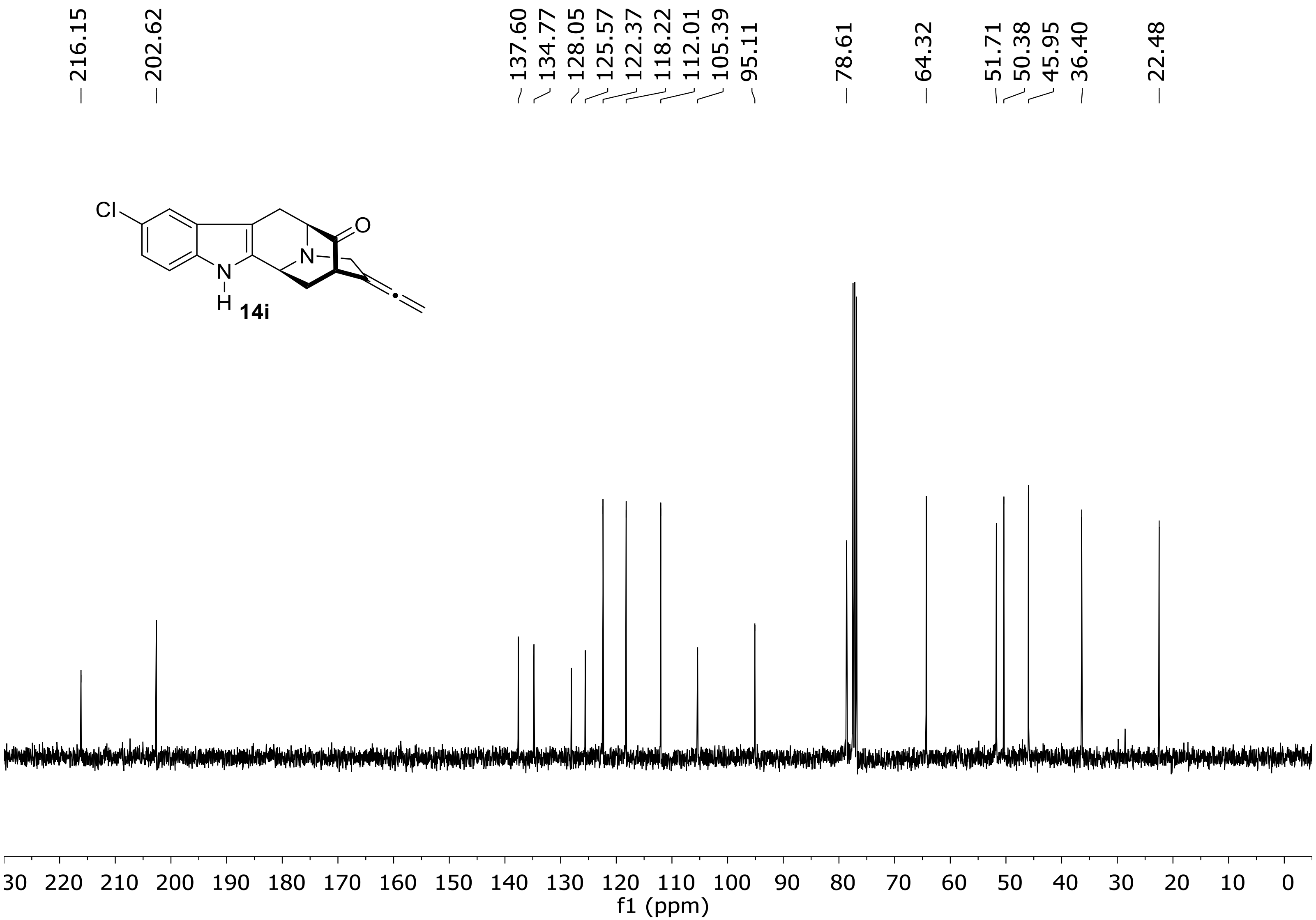
216.15
202.62
137.60
134.77
128.05
125.57
122.37
118.22
112.01
105.39
95.11
78.61
64.32
51.71
50.38
45.95
36.40
22.48
Cl
O
N
N
H
14i
30 220 210 200 190 180 170 160 150 140 130 120 110 100 90 80 70 60 50 40 30 20 10 0
f1 (ppm)

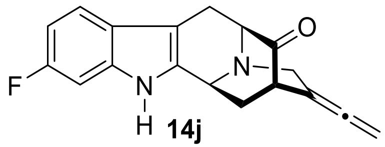

14j

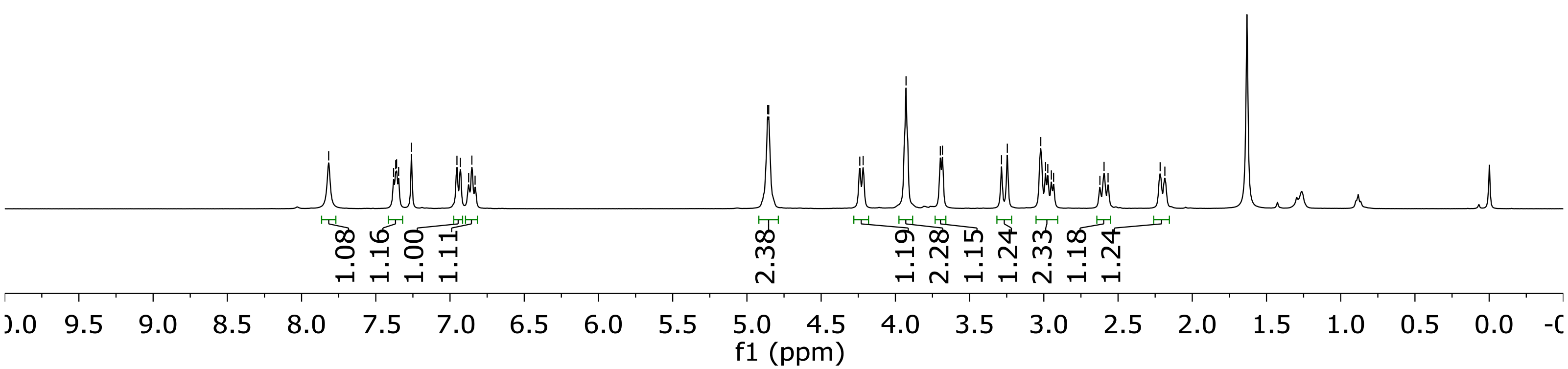

7.8175
7.3800
7.3659
7.3600
7.3462
7.2596
6.9534
6.9298
6.8746
6.8528
6.8312
4.8601
4.8531
4.2397
4.2172
3.9281
3.6972
3.6830
3.2848
3.2459
3.0206
2.9884
2.9731
2.9492
2.9341
2.6223
2.5943
2.5673
2.2164
2.1846
1.08
1.16
1.00
1.11
2.38
1.19
2.28
1.15
1.24
2.33
1.18
1.24
f1 (ppm)

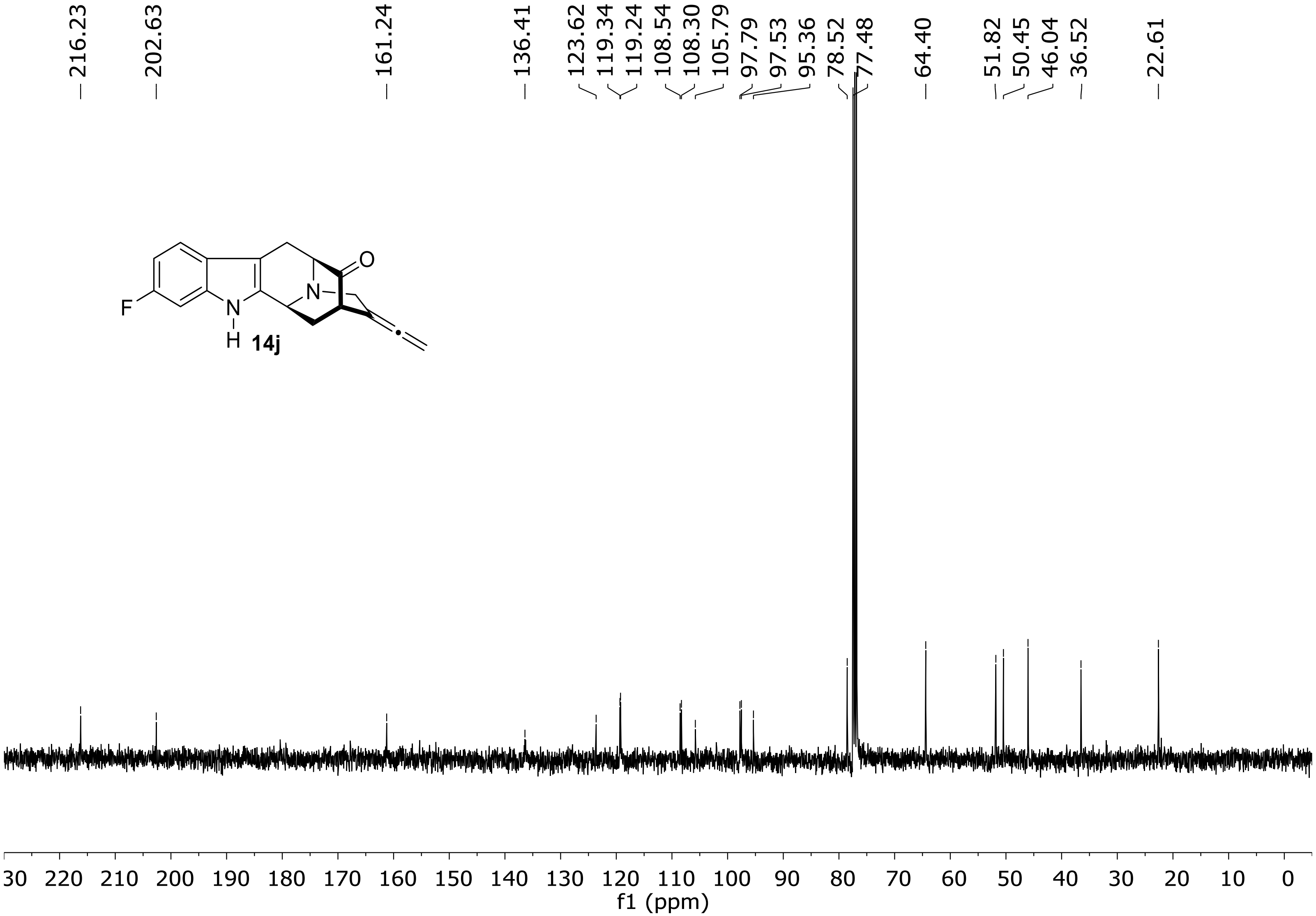
216.23
202.63
161.24
136.41
123.62
119.34
119.24
108.54
108.30
105.79
97.79
97.53
95.36
78.52
77.48
64.40
51.82
50.45
46.04
36.52
22.61
F
N
H
O
14j
30 220 210 200 190 180 170 160 150 140 130 120 110 100 90 80 70 60 50 40 30 20 10 0
f1 (ppm)

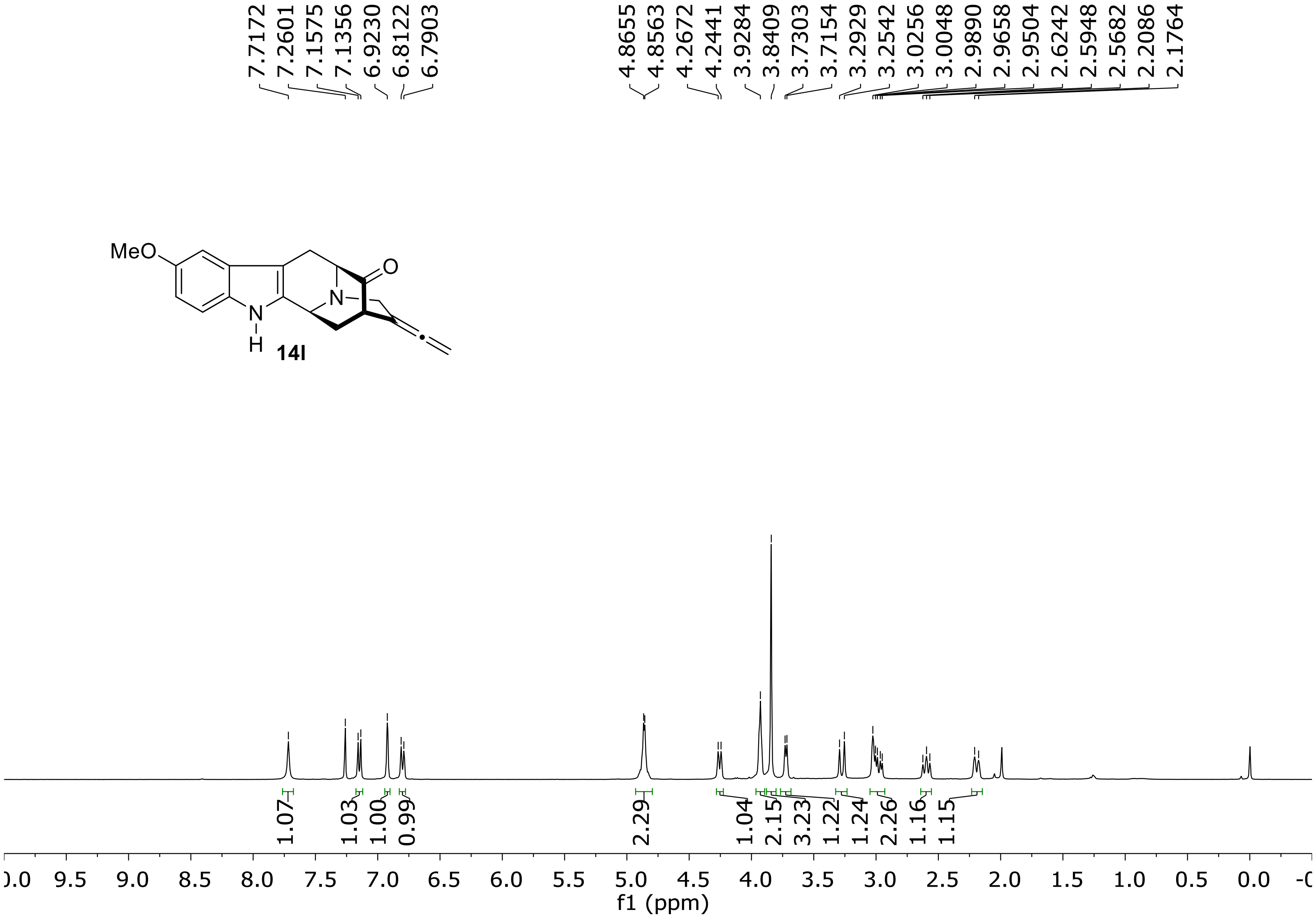

7.7172
7.2601
7.1575
7.1356
6.9230
6.8122
6.7903
4.8655
4.8563
4.2672
4.2441
3.9284
3.8409
3.7303
3.7154
3.2929
3.2542
3.0256
3.0048
2.9890
2.9658
2.9504
2.6242
2.5948
2.5682
2.2086
2.1764
MeO
O
N
N
H
14l
1.07
1.03
1.00
0.99
2.29
1.04
2.15
3.23
1.22
1.24
2.26
1.16
1.15
f1 (ppm)

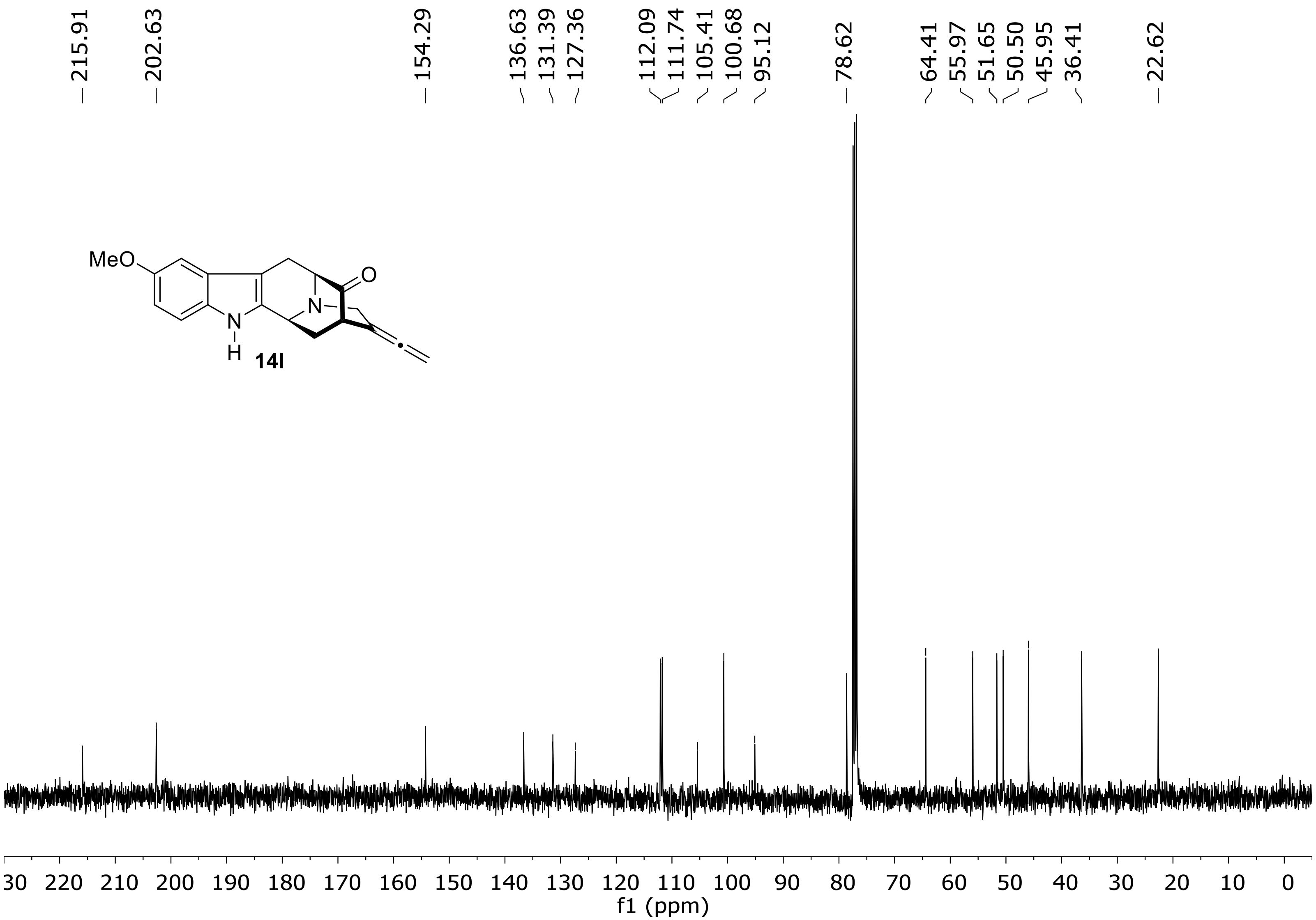
215.91
202.63
154.29
136.63
131.39
127.36
112.09
111.74
105.41
100.68
95.12
78.62
64.41
55.97
51.65
50.50
45.95
36.41
22.62
MeO
O
N
N
H
14l
30 220 210 200 190 180 170 160 150 140 130 120 110 100 90 80 70 60 50 40 30 20 10 0
f1 (ppm)

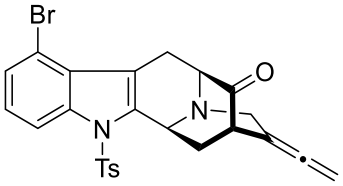
Br
O
N
N
Ts

14-1e

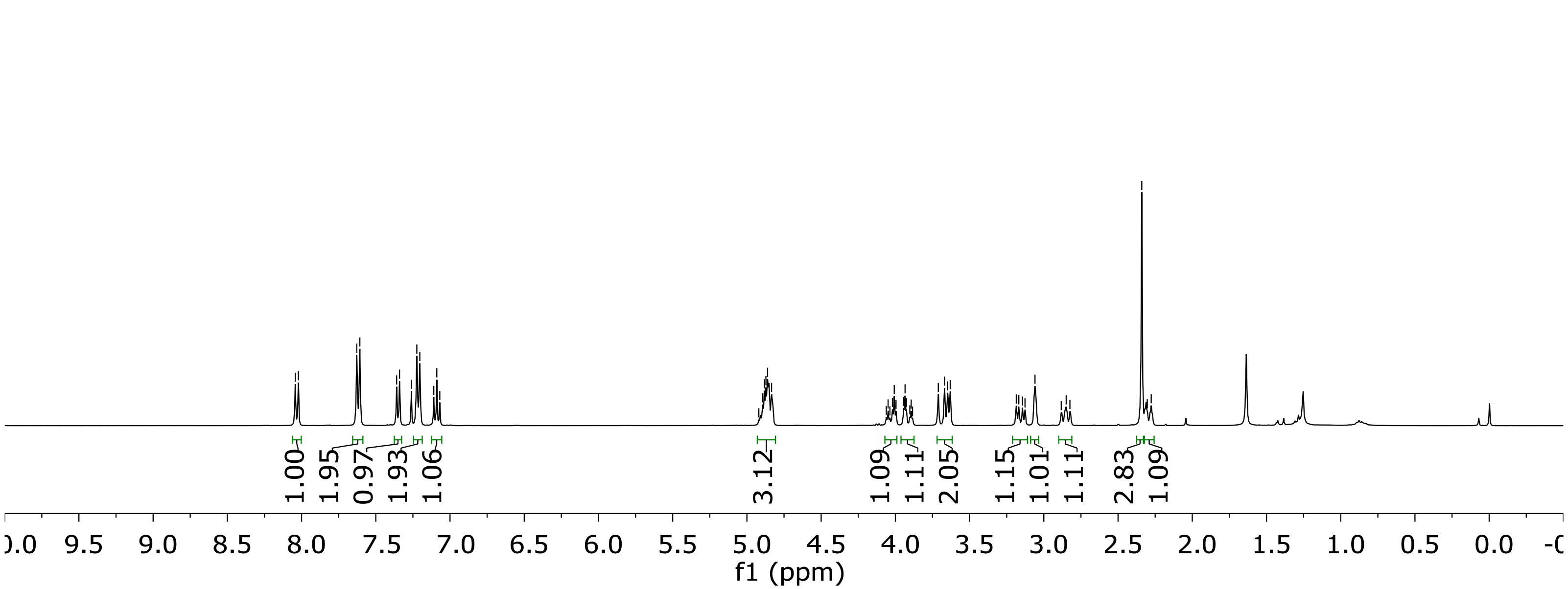
8.0425
8.0216
7.6281
7.6074
7.3596
7.3402
7.2602
7.2237
7.2034
7.1094
7.0891
7.0688
4.9197
4.8933
4.8823
4.8720
4.8609
4.8340
4.0490
4.0197
4.0077
3.9957
3.9436
3.9347
3.9257
3.8934
3.7108
3.6683
3.6477
3.6306
3.1854
3.1685
3.1438
3.1267
3.0594
2.8493
2.3401
2.3032
1.00
1.95
0.97
1.93
1.06
3.12
1.09
1.11
2.05
1.15
1.01
1.11
2.83
1.09
9.5
9.0
8.5
8.0
7.5
7.0
6.5
6.0
5.5
5.0
4.5
4.0
3.5
3.0
2.5
2.0
1.5
1.0
0.5
0.0
f1 (ppm)

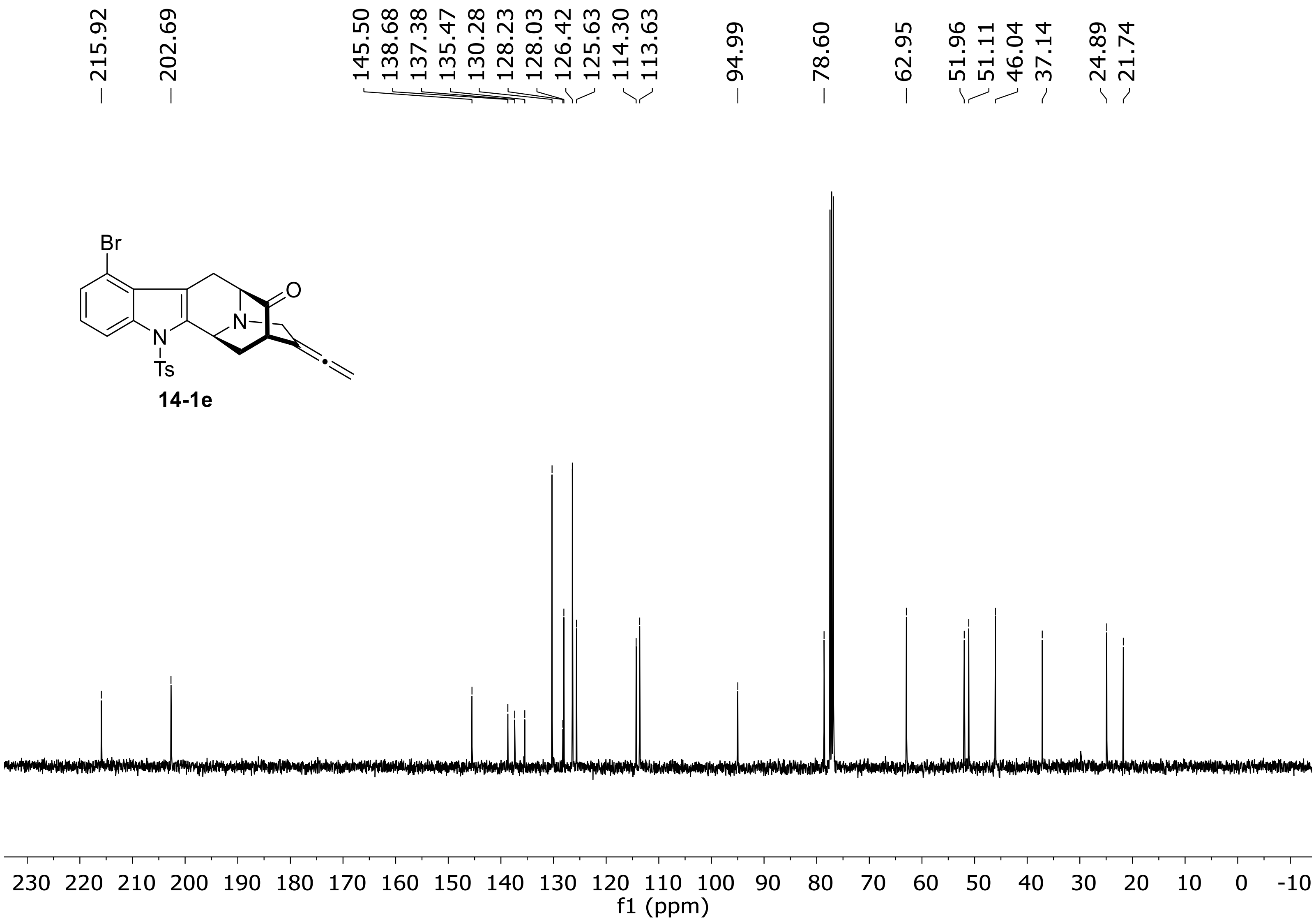

215.92
202.69
145.50
138.68
137.38
135.47
130.28
128.23
128.03
126.42
125.63
114.30
113.63
94.99
78.60
62.95
51.96
51.11
46.04
37.14
24.89
21.74
Br
O
N
N
Ts
230 220 210 200 190 180 170 160 150 140 130 120 110 100 90 80 70 60 50 40 30 20 10 0 -10
f1 (ppm)

14-1e

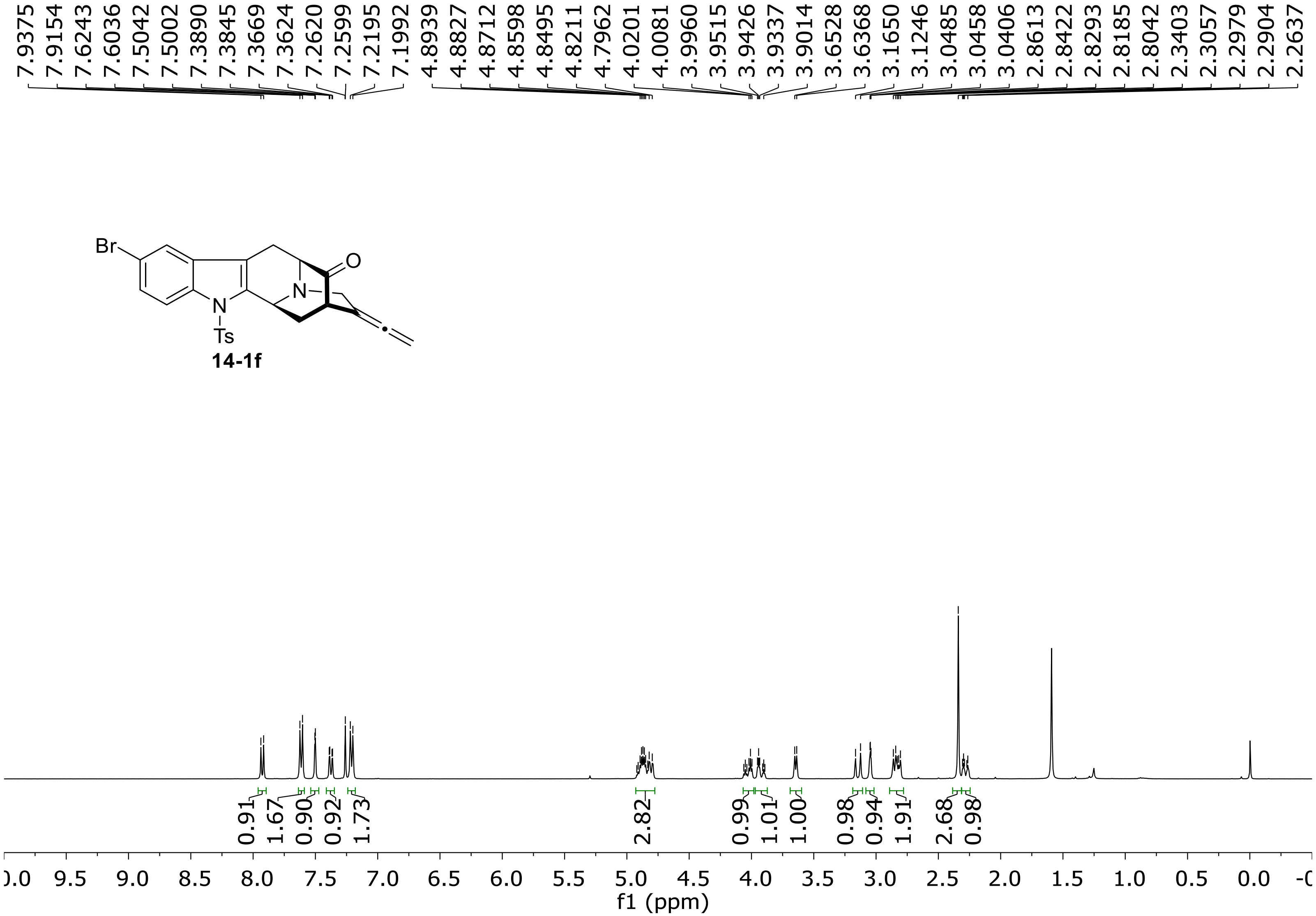
7.9375
7.9154
7.6243
7.6036
7.5042
7.5002
7.3890
7.3845
7.3669
7.3624
7.2620
7.2599
7.2195
7.1992
4.8939
4.8827
4.8712
4.8598
4.8495
4.8211
4.7962
4.0201
4.0081
3.9960
3.9515
3.9426
3.9337
3.9014
3.6528
3.6368
3.1650
3.1246
3.0485
3.0458
3.0406
2.8613
2.8422
2.8293
2.8185
2.8042
2.3403
2.3057
2.2979
2.2904
2.2637
Br
N
Ts
N
O
14-1f
0.91
1.67
0.90
0.92
1.73
2.82
0.99
1.01
1.00
0.98
0.94
1.91
2.68
0.98
0.0 9.5 9.0 8.5 8.0 7.5 7.0 6.5 6.0 5.5 5.0 4.5 4.0 3.5 3.0 2.5 2.0 1.5 1.0 0.5 0.0
f1 (ppm)

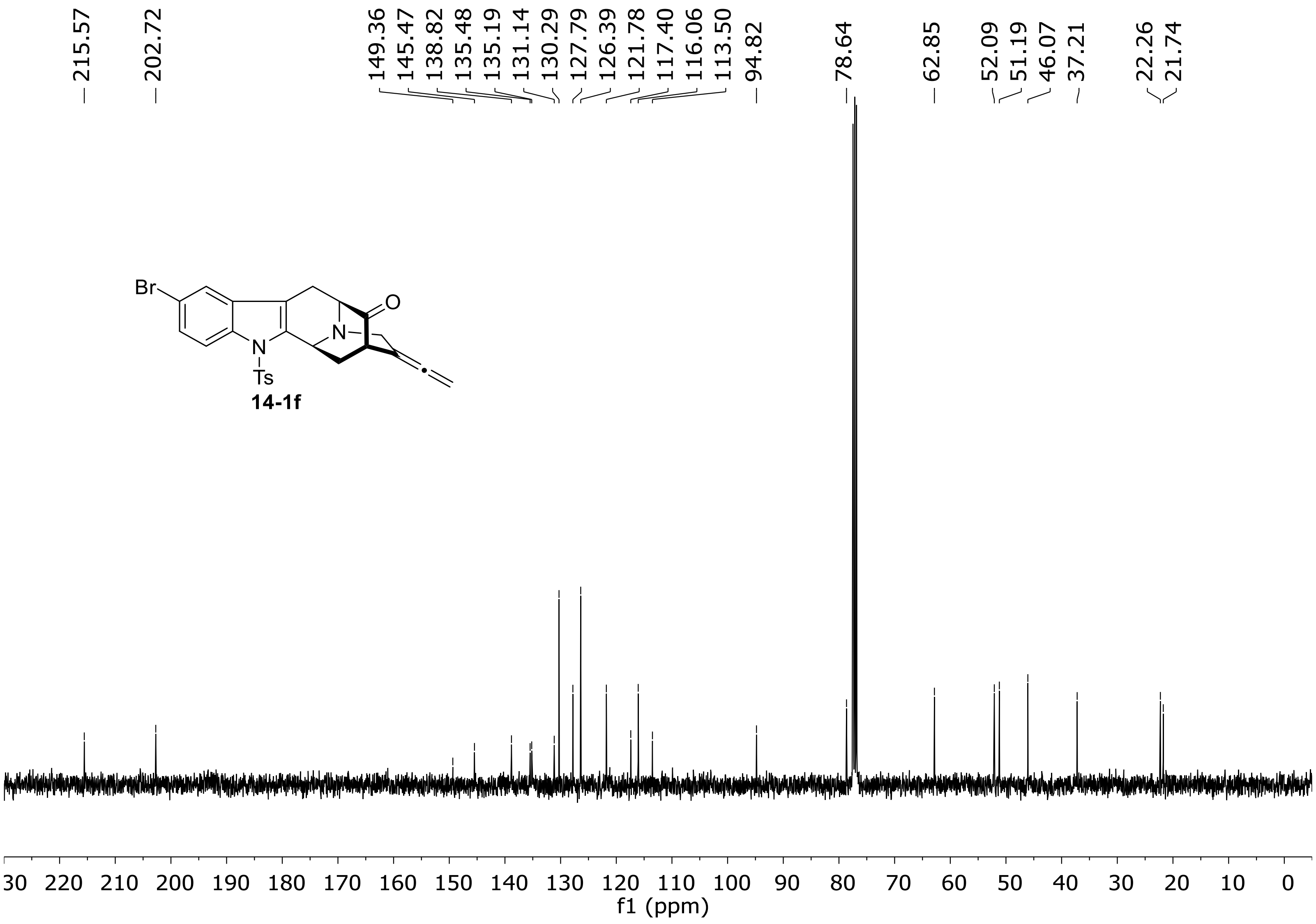
215.57
202.72
149.36
145.47
138.82
135.48
135.19
131.14
130.29
127.79
126.39
121.78
117.40
116.06
113.50
94.82
78.64
62.85
52.09
51.19
46.07
37.21
22.26
21.74
Br
O
N
N
Ts
14-1f
30 220 210 200 190 180 170 160 150 140 130 120 110 100 90 80 70 60 50 40 30 20 10 0
f1 (ppm)

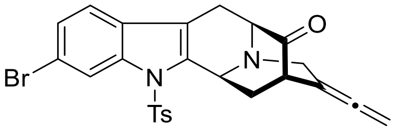

14-1g

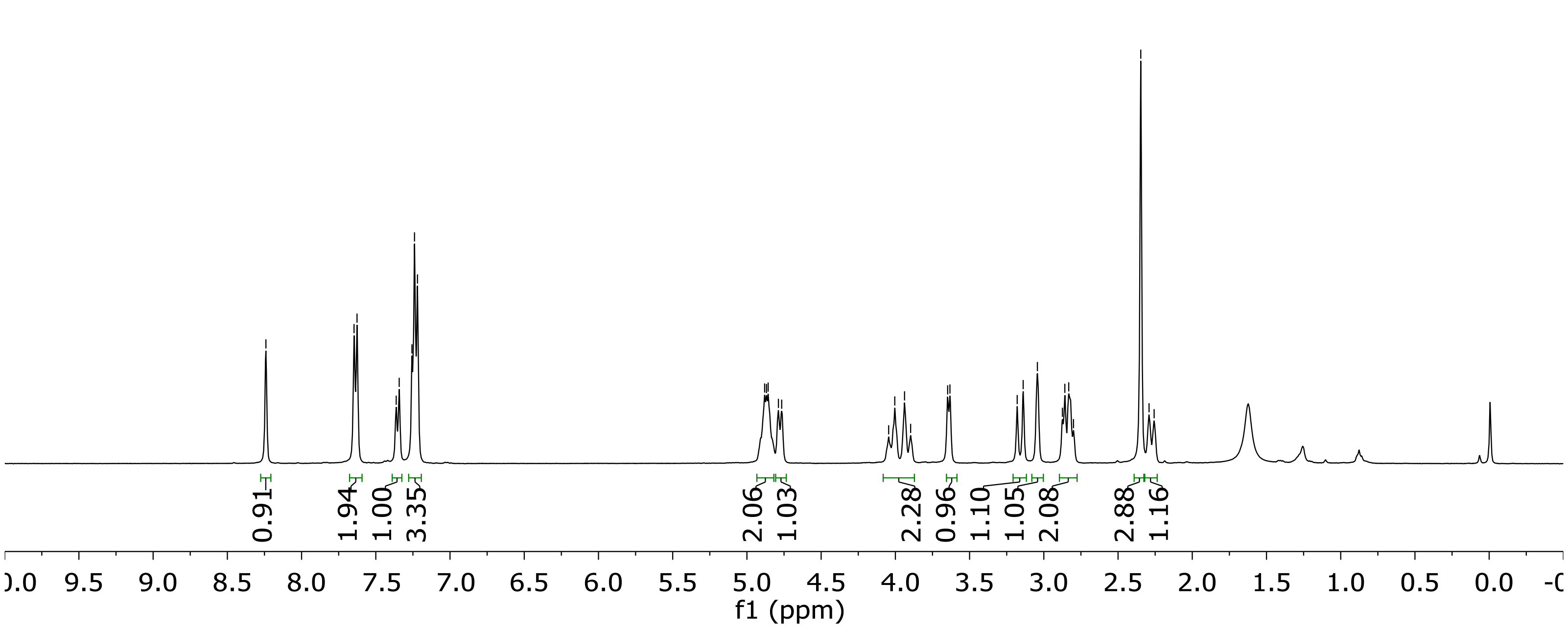
8.2402
7.6465
7.6264
7.3625
7.3423
7.2567
7.2393
7.2193
4.8807
4.8684
4.8571
4.7879
4.7657
4.0452
4.0040
3.9383
3.8972
3.6477
3.6324
3.1794
3.1391
3.0434
2.8748
2.8585
2.8322
2.8002
2.3469
2.2911
2.2569
0.91
1.94
1.00
3.35
2.06
1.03
2.28
0.96
1.10
1.05
2.08
2.88
1.16
f1 (ppm)

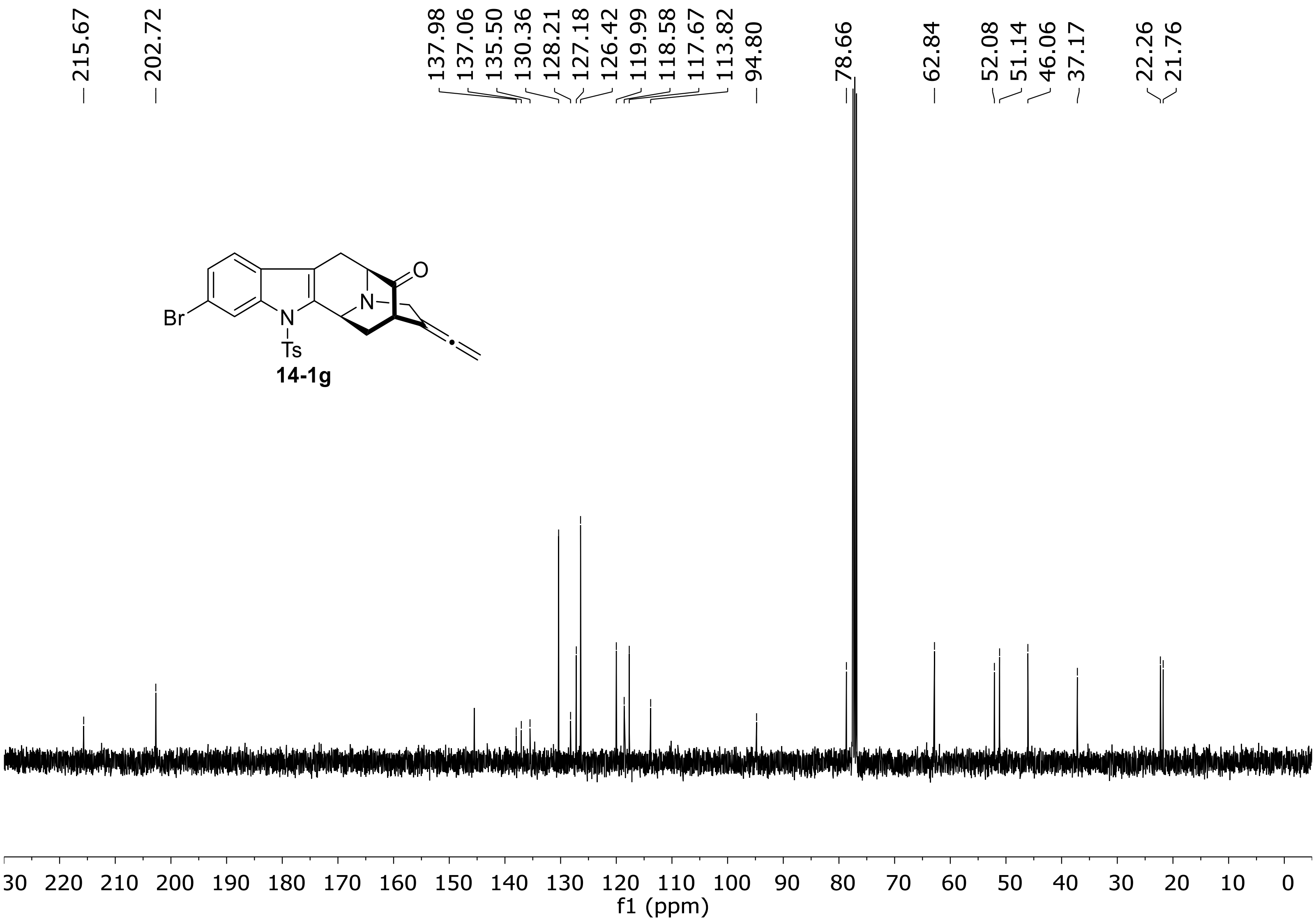
215.67
202.72
137.98
137.06
135.50
130.36
128.21
127.18
126.42
119.99
118.58
117.67
113.82
94.80
78.66
62.84
52.08
51.14
46.06
37.17
22.26
21.76
Br
N
Ts
N
O
14-1g
f1 (ppm)

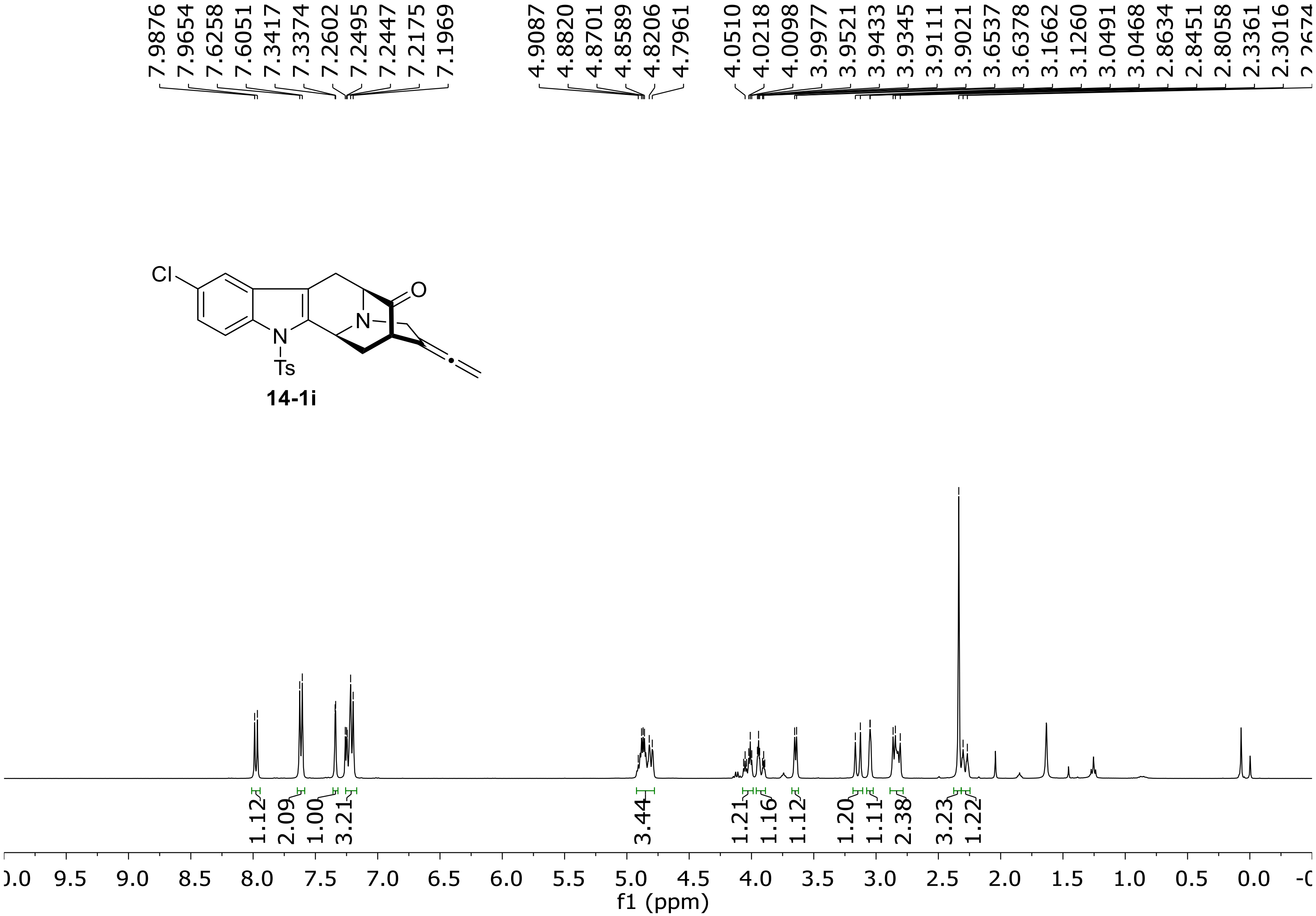
Cl
N
Ts
N
O
14-1i
7.9876
7.9654
7.6258
7.6051
7.3417
7.3374
7.2602
7.2495
7.2447
7.2175
7.1969
4.9087
4.8820
4.8701
4.8589
4.8206
4.7961
4.0510
4.0218
4.0098
3.9977
3.9521
3.9433
3.9345
3.9111
3.9021
3.6537
3.6378
3.1662
3.1260
3.0491
3.0468
2.8634
2.8451
2.8058
2.3361
2.3016
1.12
2.09
1.00
3.21
3.44
1.21
1.16
1.12
1.20
1.11
2.38
3.23
1.22
9.5
9.0
8.5
8.0
7.5
7.0
6.5
6.0
5.5
5.0
4.5
4.0
3.5
3.0
2.5
2.0
1.5
1.0
0.5
0.0
f1 (ppm)

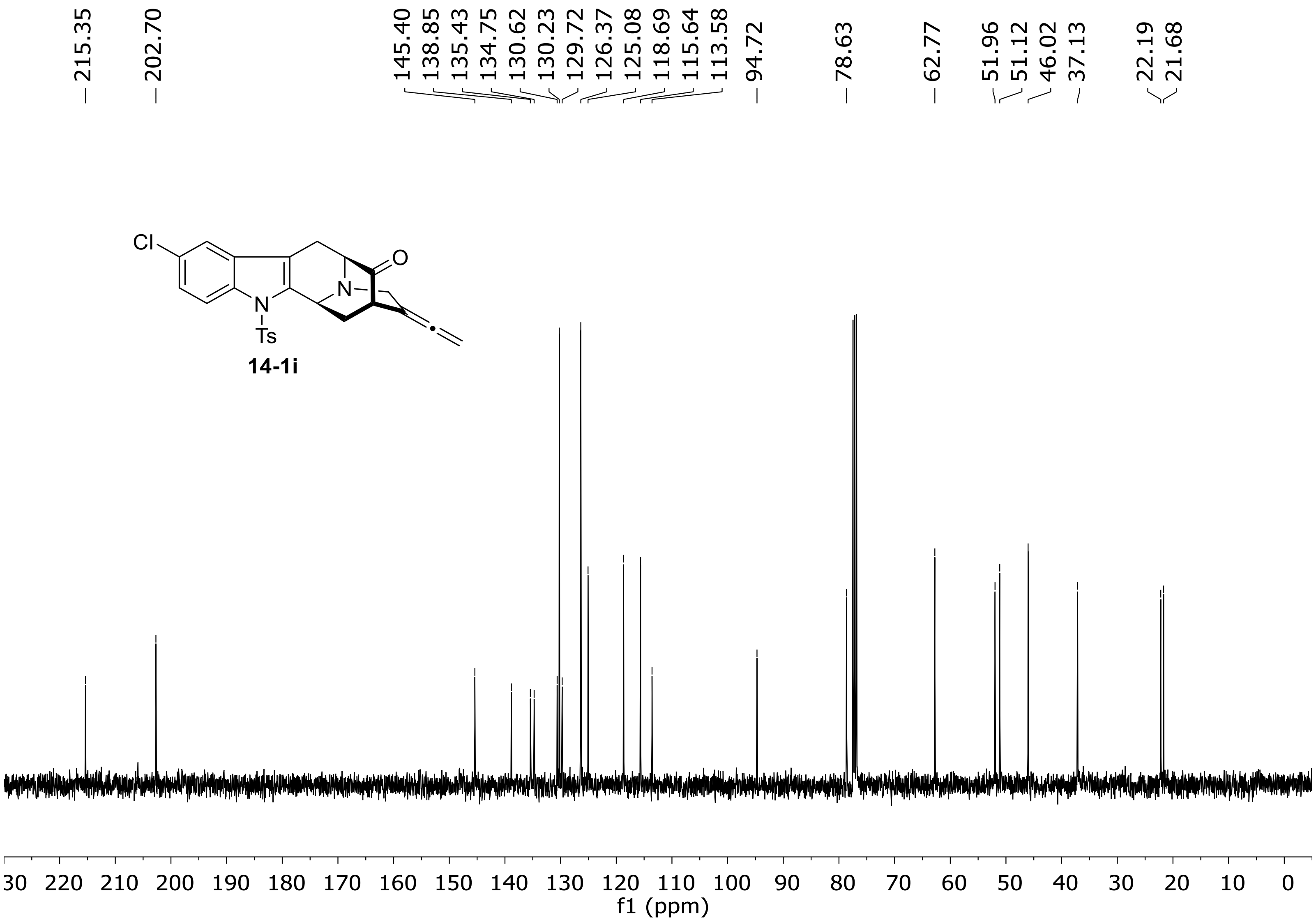
215.35
202.70
145.40
138.85
135.43
134.75
130.62
130.23
129.72
126.37
125.08
118.69
115.64
113.58
94.72
78.63
62.77
51.96
51.12
46.02
37.13
22.19
21.68
Cl
O
N
N
Ts
30 220 210 200 190 180 170 160 150 140 130 120 110 100 90 80 70 60 50 40 30 20 10 0
f1 (ppm)

14-1i

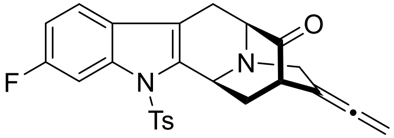
F
N
Ts
N
O

14-1j

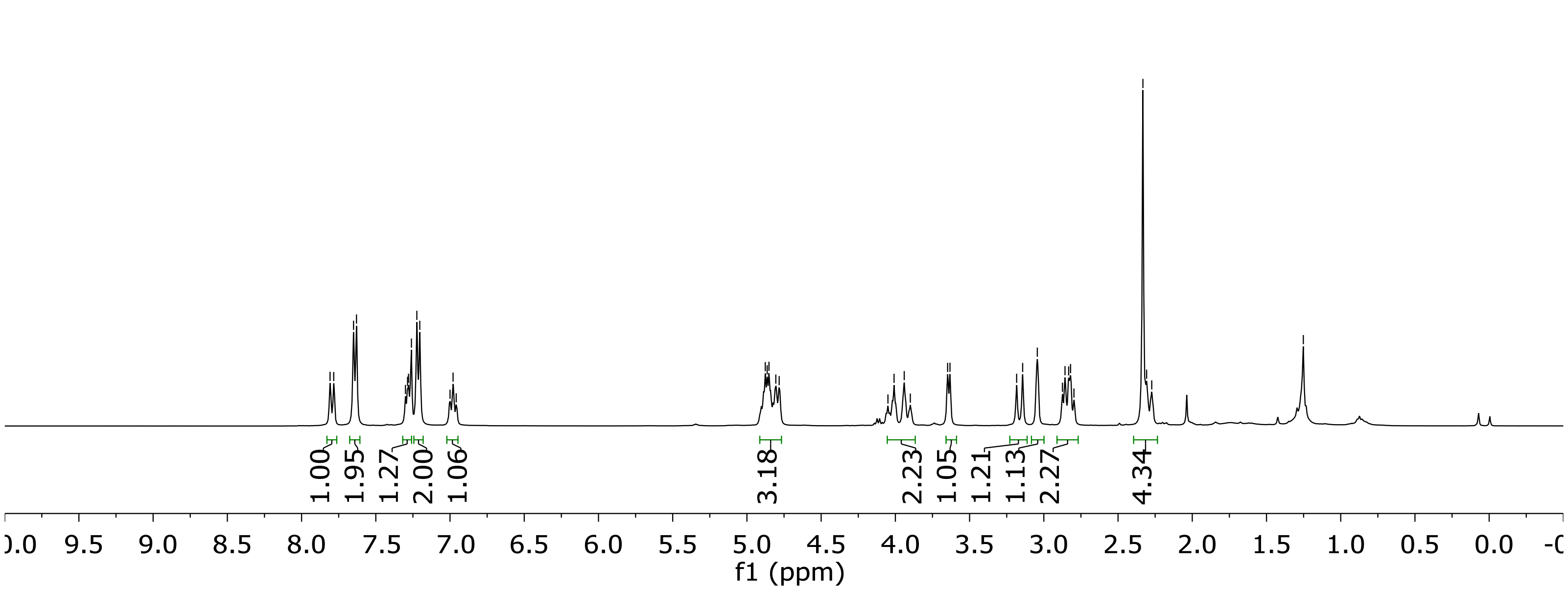
7.8082
7.7835
7.6500
7.6301
7.3002
7.2861
7.2795
7.2600
7.2236
7.2037
6.9999
6.9795
6.9584
4.8765
4.8637
4.8515
4.8053
4.7827
4.0500
4.0089
3.9404
3.8993
3.6479
3.6322
3.1830
3.1426
3.0439
2.8747
2.8573
2.8326
2.8199
2.7972
2.3326
2.3076
2.2730
1.2519
1.00
1.95
1.27
2.00
1.06
3.18
2.23
1.05
1.21
1.13
2.27
4.34
10.0 9.5 9.0 8.5 8.0 7.5 7.0 6.5 6.0 5.5 5.0 4.5 4.0 3.5 3.0 2.5 2.0 1.5 1.0 0.5 0.0
f1 (ppm)

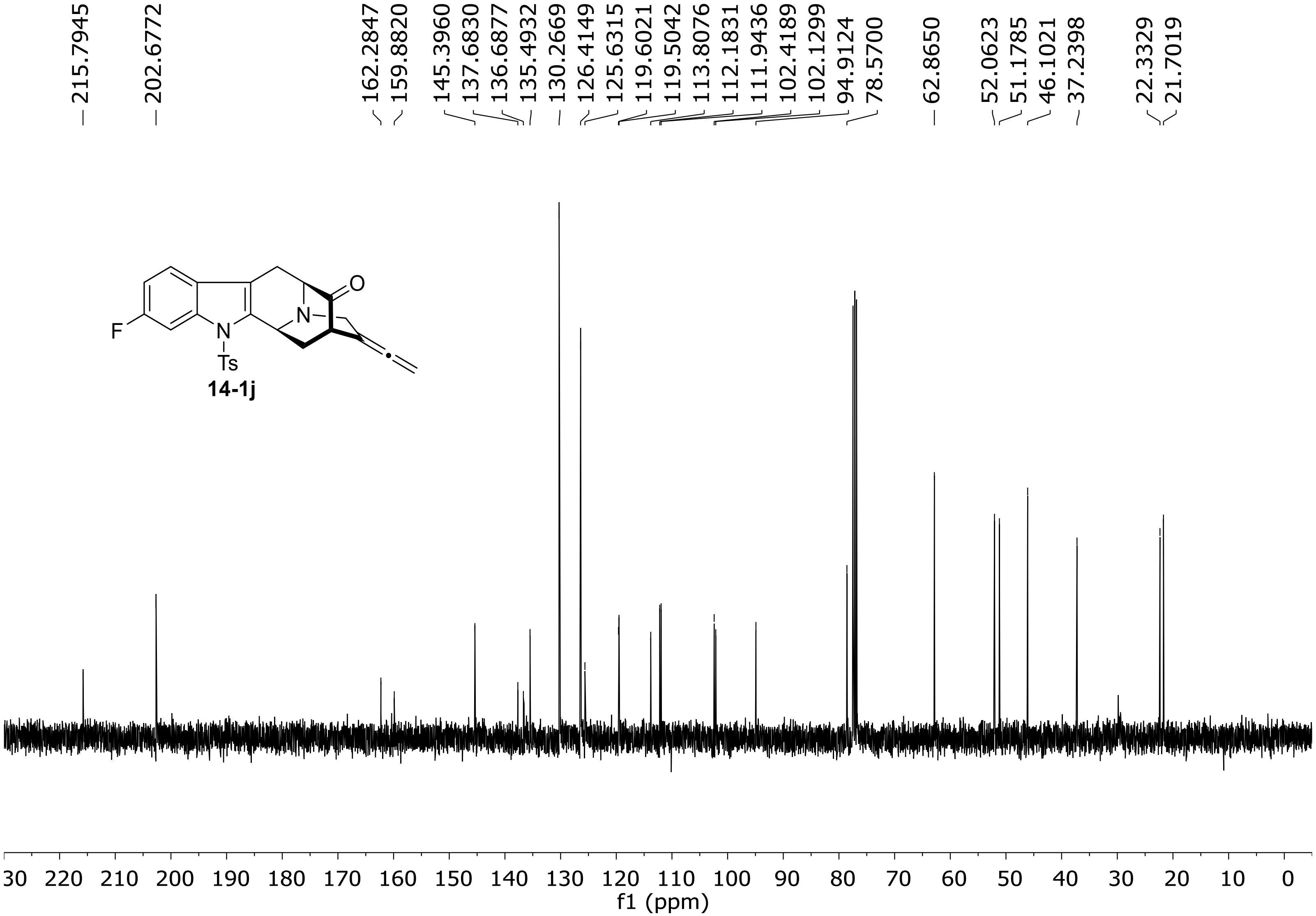
215.7945
202.6772
162.2847
159.8820
145.3960
137.6830
136.6877
135.4932
130.2669
126.4149
125.6315
119.6021
119.5042
113.8076
112.1831
111.9436
102.4189
102.1299
94.9124
78.5700
62.8650
52.0623
51.1785
46.1021
37.2398
22.3329
21.7019
O
N
F
N
Ts
14-1j
30 220 210 200 190 180 170 160 150 140 130 120 110 100 90 80 70 60 50 40 30 20 10 0
f1 (ppm)

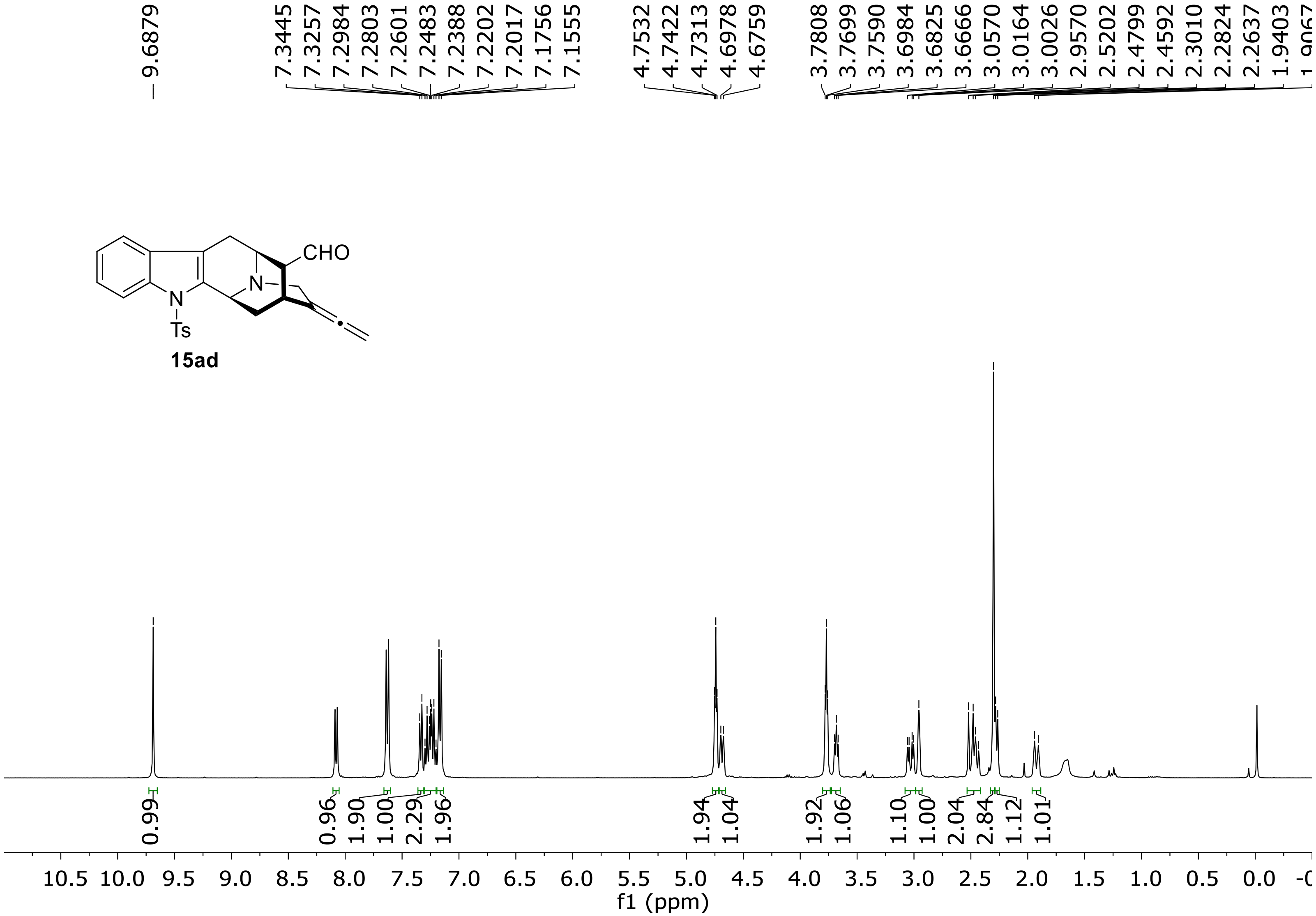
9.6879
7.3445
7.3257
7.2984
7.2803
7.2601
7.2483
7.2388
7.2202
7.2017
7.1756
7.1555
4.7532
4.7422
4.7313
4.6978
4.6759
3.7808
3.7699
3.7590
3.6984
3.6825
3.6666
3.0570
3.0164
3.0026
2.9570
2.5202
2.4799
2.4592
2.3010
2.2824
2.2637
1.9403
CHO
N
N
Ts
15ad
0.99
0.96
1.90
1.00
2.29
1.96
1.94
1.04
1.92
1.06
1.10
1.00
2.04
2.84
1.12
1.01
10.5 10.0 9.5 9.0 8.5 8.0 7.5 7.0 6.5 6.0 5.5 5.0 4.5 4.0 3.5 3.0 2.5 2.0 1.5 1.0 0.5 0.0
f1 (ppm)

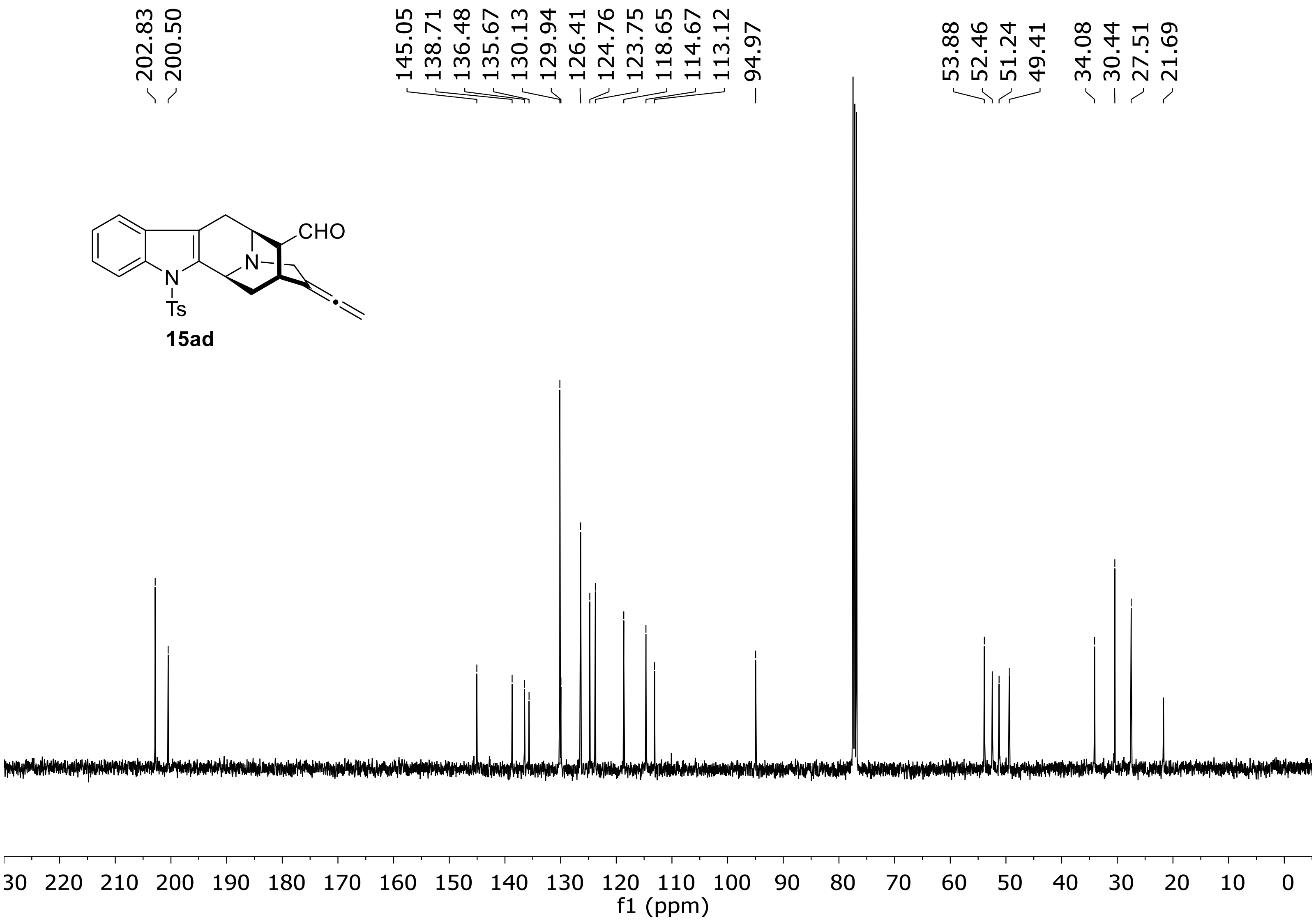
202.83
200.50
145.05
138.71
136.48
135.67
130.13
129.94
126.41
124.76
123.75
118.65
114.67
113.12
94.97
53.88
52.46
51.24
49.41
34.08
30.44
27.51
21.69
CHO
N
N
Ts
15ad
30 220 210 200 190 180 170 160 150 140 130 120 110 100 90 80 70 60 50 40 30 20 10 0
f1 (ppm)

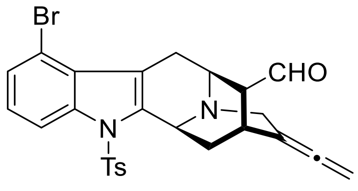

Br
CHO
N
N
Ts

15ae

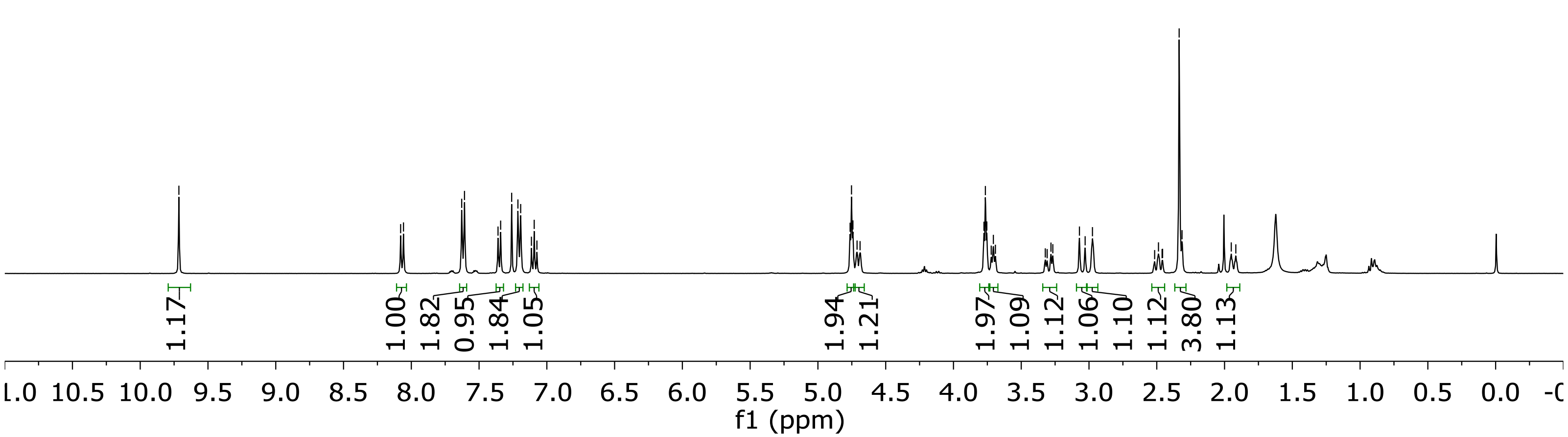

9.7147
8.0786
8.0577
7.6282
7.6076
7.3602
7.3407
7.2596
7.2133
7.1930
7.1137
7.0933
7.0732
4.7625
4.7522
4.7417
4.7113
4.6891
3.7750
3.7645
3.7539
3.7215
3.7057
3.6905
3.3227
3.2806
3.2670
3.0705
3.0285
2.9745
2.4875
2.4570
2.3349
2.3137
1.9505
1.17
1.00
1.82
0.95
1.84
1.05
1.94
1.21
1.97
1.09
1.12
1.06
1.10
1.12
3.80
1.13
f1 (ppm)

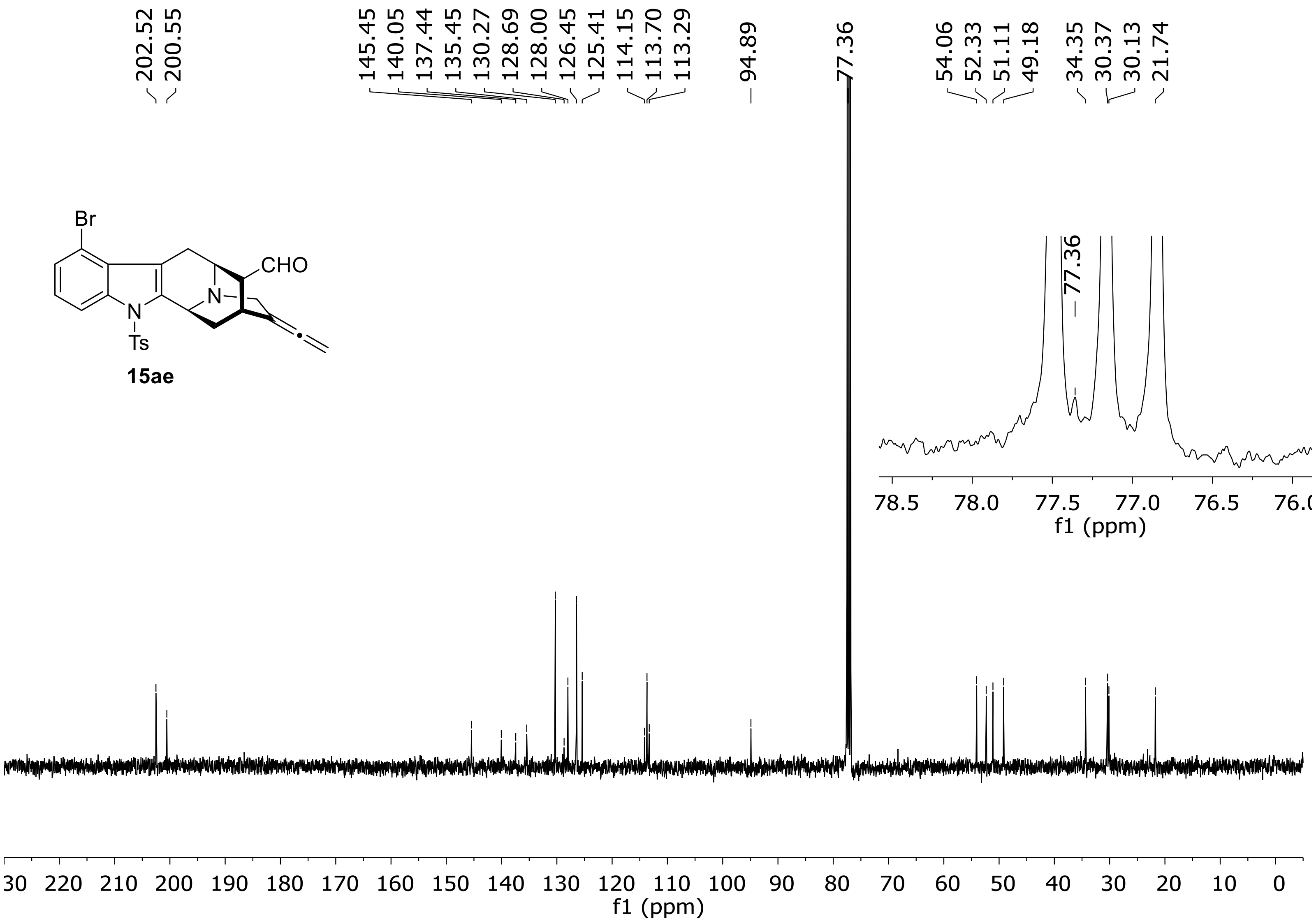
202.52
200.55
145.45
140.05
137.44
135.45
130.27
128.69
128.00
126.45
125.41
114.15
113.70
113.29
94.89
77.36
54.06
52.33
51.11
49.18
34.35
30.37
30.13
21.74
Br
CHO
N
N
Ts
15ae
77.36
78.5 78.0 77.5 77.0 76.5 76.(
f1 (ppm)
30 220 210 200 190 180 170 160 150 140 130 120 110 100 90 80 70 60 50 40 30 20 10 0
f1 (ppm)

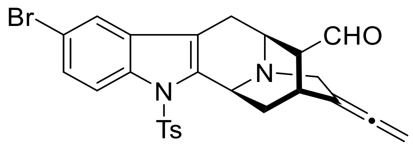

15af

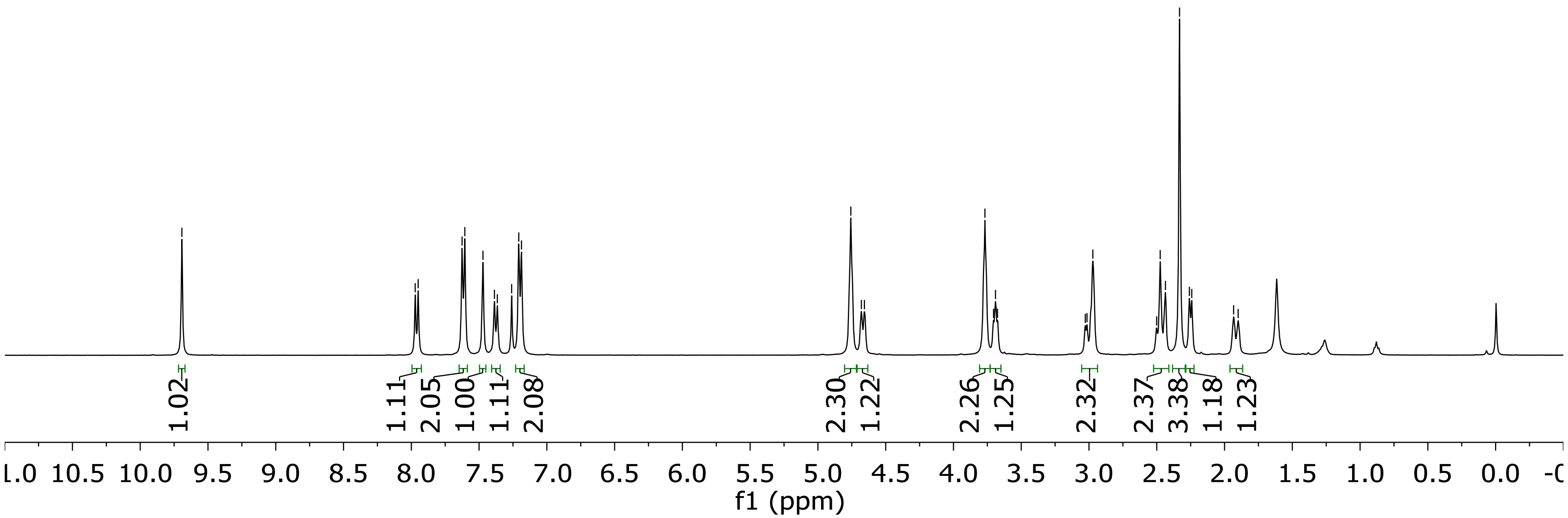
9.6926
7.9713
7.9494
7.6255
7.6054
7.4712
7.3867
7.3653
7.2598
7.2072
7.1874
4.7571
4.6793
4.6568
3.7679
3.7043
3.6897
3.6752
3.0276
3.0148
2.9717
2.5008
2.4748
2.4361
2.3321
2.2591
2.2419
1.9330
1.8995
1.02
1.11
2.05
1.00
1.11
2.08
2.30
1.22
2.26
1.25
2.32
2.37
3.38
1.18
1.23
10.5 10.0 9.5 9.0 8.5 8.0 7.5 7.0 6.5 6.0 5.5 5.0 4.5 4.0 3.5 3.0 2.5 2.0 1.5 1.0 0.5 0.0
f1 (ppm)

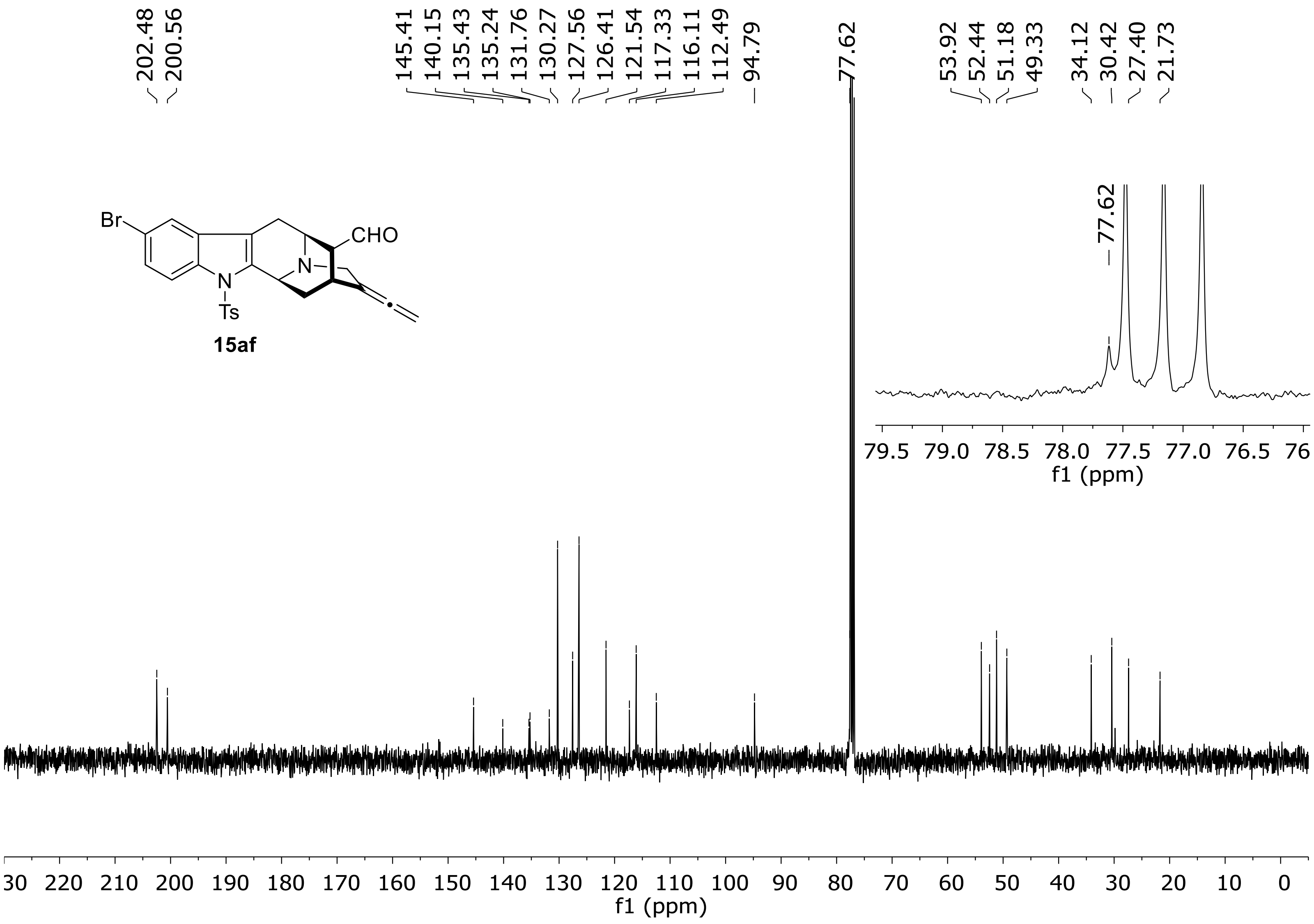
202.48
200.56
145.41
140.15
135.43
135.24
131.76
130.27
127.56
126.41
121.54
117.33
116.11
112.49
94.79
77.62
53.92
52.44
51.18
49.33
34.12
30.42
27.40
21.73
Br
CHO
N
N
Ts
15af
77.62
79.5 79.0 78.5 78.0 77.5 77.0 76.5 76
f1 (ppm)
30 220 210 200 190 180 170 160 150 140 130 120 110 100 90 80 70 60 50 40 30 20 10 0
f1 (ppm)

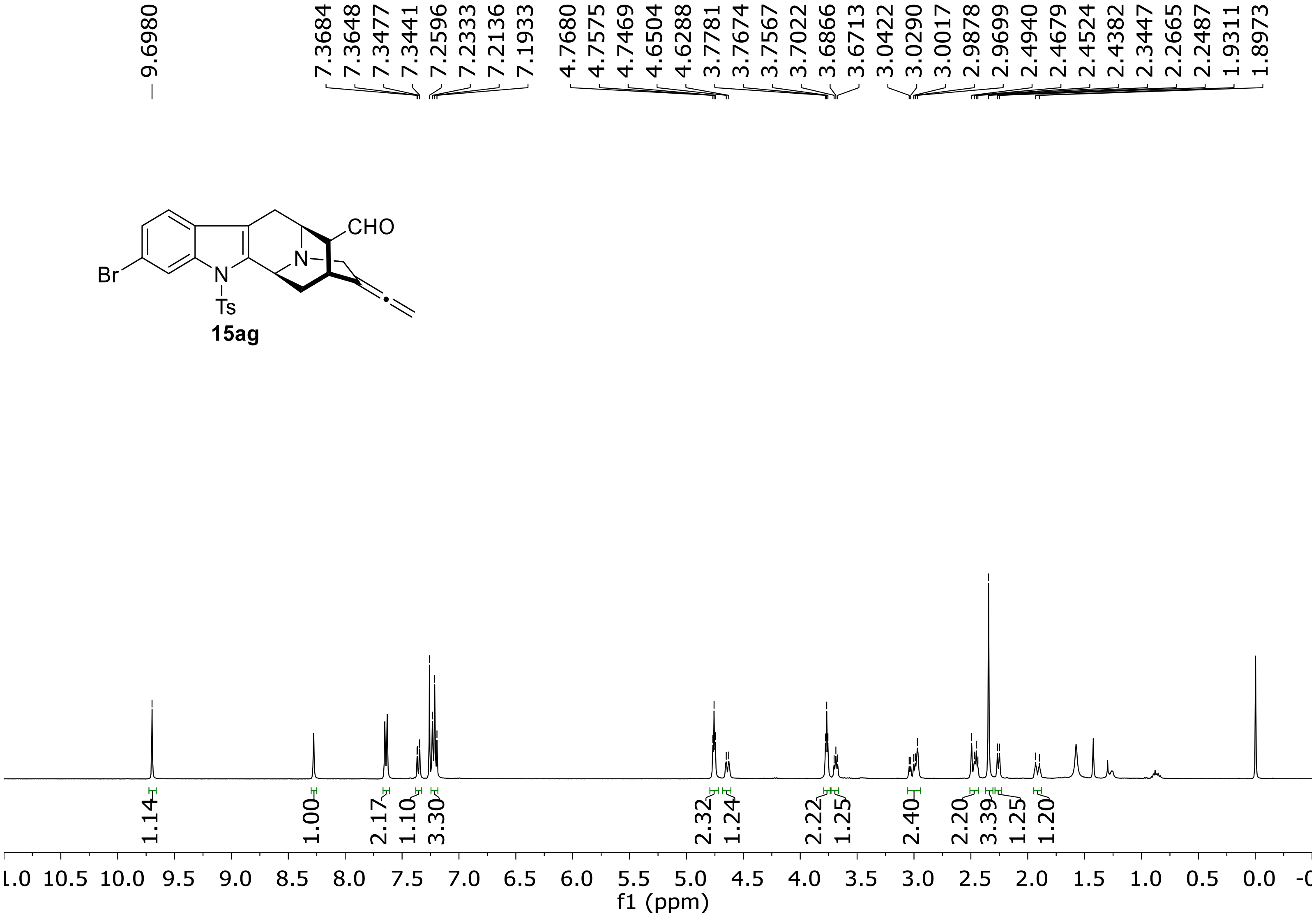
9.6980
7.3684
7.3648
7.3477
7.3441
7.2596
7.2333
7.2136
7.1933
4.7680
4.7575
4.7469
4.6504
4.6288
3.7781
3.7674
3.7567
3.7022
3.6866
3.6713
3.0422
3.0290
3.0017
2.9878
2.9699
2.4940
2.4679
2.4524
2.4382
2.3447
2.2665
2.2487
1.9311
1.8973
CHO
Br
N
Ts
N
15ag
1.14
1.00
2.17
1.10
3.30
2.32
1.24
2.22
1.25
2.40
2.20
3.39
1.25
1.20
10.5 10.0 9.5 9.0 8.5 8.0 7.5 7.0 6.5 6.0 5.5 5.0 4.5 4.0 3.5 3.0 2.5 2.0 1.5 1.0 0.5 0.0
f1 (ppm)

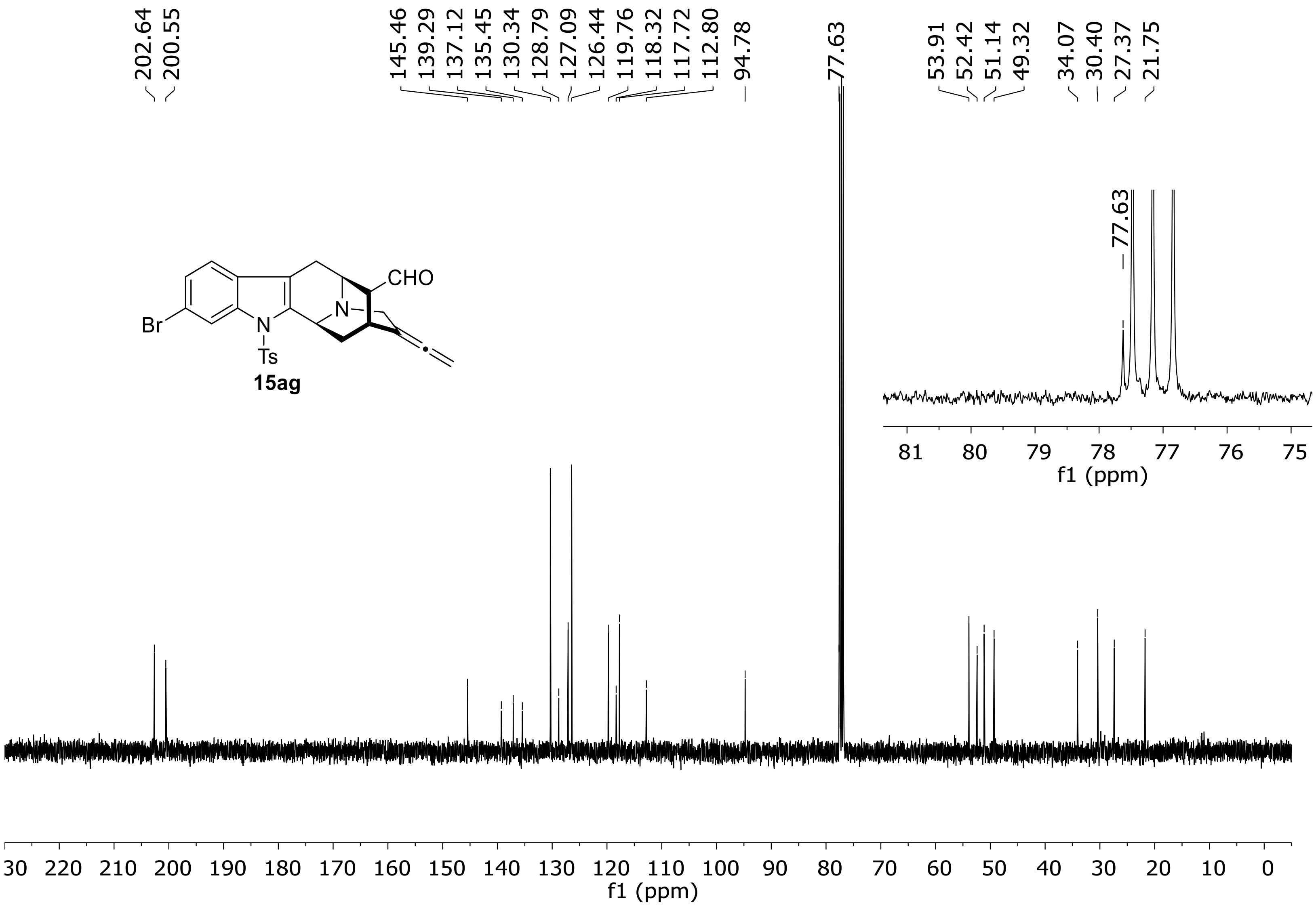

202.64
200.55
145.46
139.29
137.12
135.45
130.34
128.79
127.09
126.44
119.76
118.32
117.72
112.80
94.78
77.63
53.91
52.42
51.14
49.32
34.07
30.40
27.37
21.75
CHO
Br
N
N
Ts
15ag
77.63
81 80 79 78 77 76 75
f1 (ppm)
30 220 210 200 190 180 170 160 150 140 130 120 110 100 90 80 70 60 50 40 30 20 10 0
f1 (ppm)

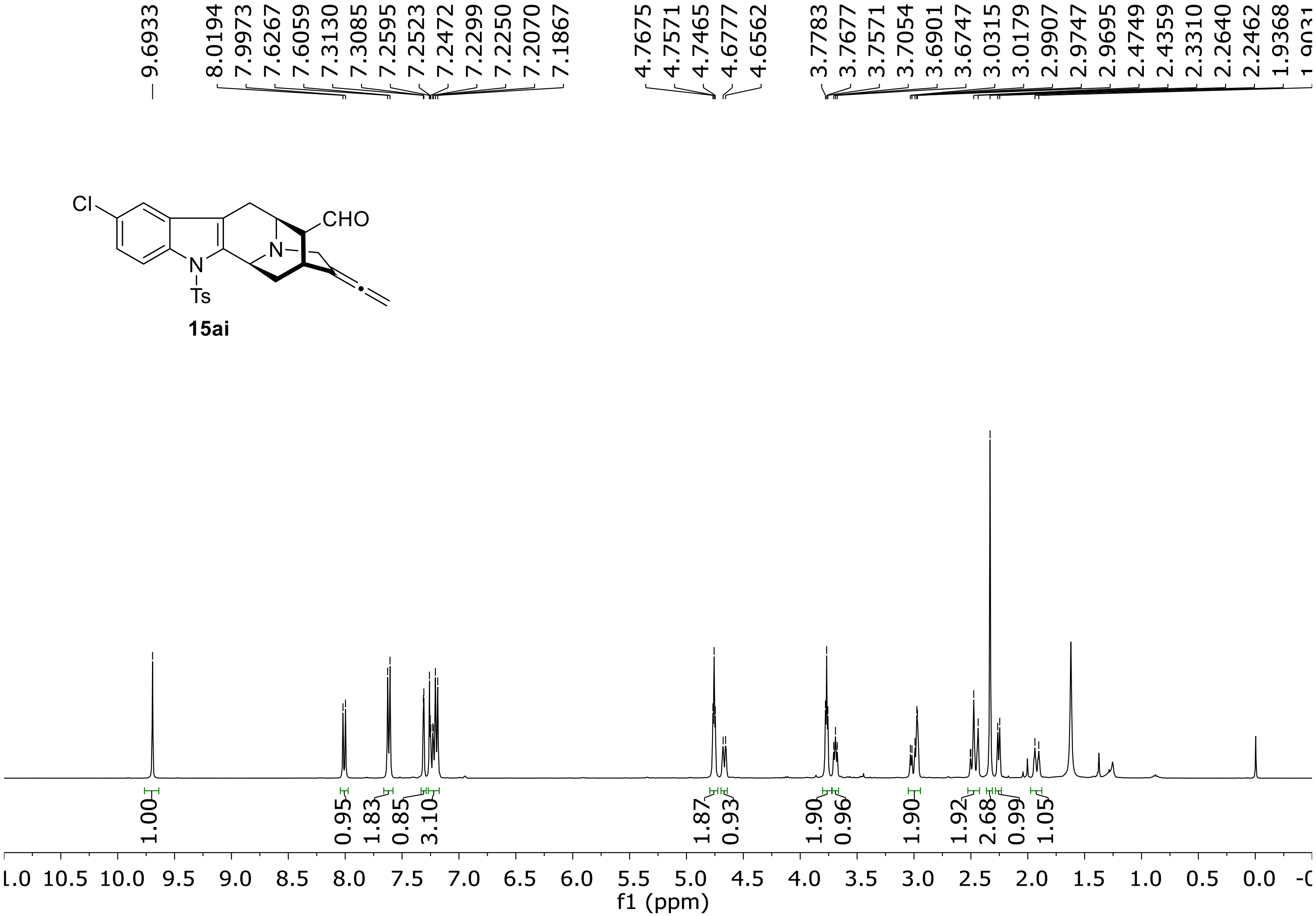
9.6933
8.0194
7.9973
7.6267
7.6059
7.3130
7.3085
7.2595
7.2523
7.2472
7.2299
7.2250
7.2070
7.1867
4.7675
4.7571
4.7465
4.6777
4.6562
3.7783
3.7677
3.7571
3.7054
3.6901
3.6747
3.0315
3.0179
2.9907
2.9747
2.9695
2.4749
2.4359
2.3310
2.2640
2.2462
1.9368
Cl
CHO
N
N
Ts
15ai
1.00
0.95
1.83
0.85
3.10
1.87
0.93
1.90
0.96
1.90
1.92
2.68
0.99
1.05
10.5 10.0 9.5 9.0 8.5 8.0 7.5 7.0 6.5 6.0 5.5 5.0 4.5 4.0 3.5 3.0 2.5 2.0 1.5 1.0 0.5 0.0
f1 (ppm)

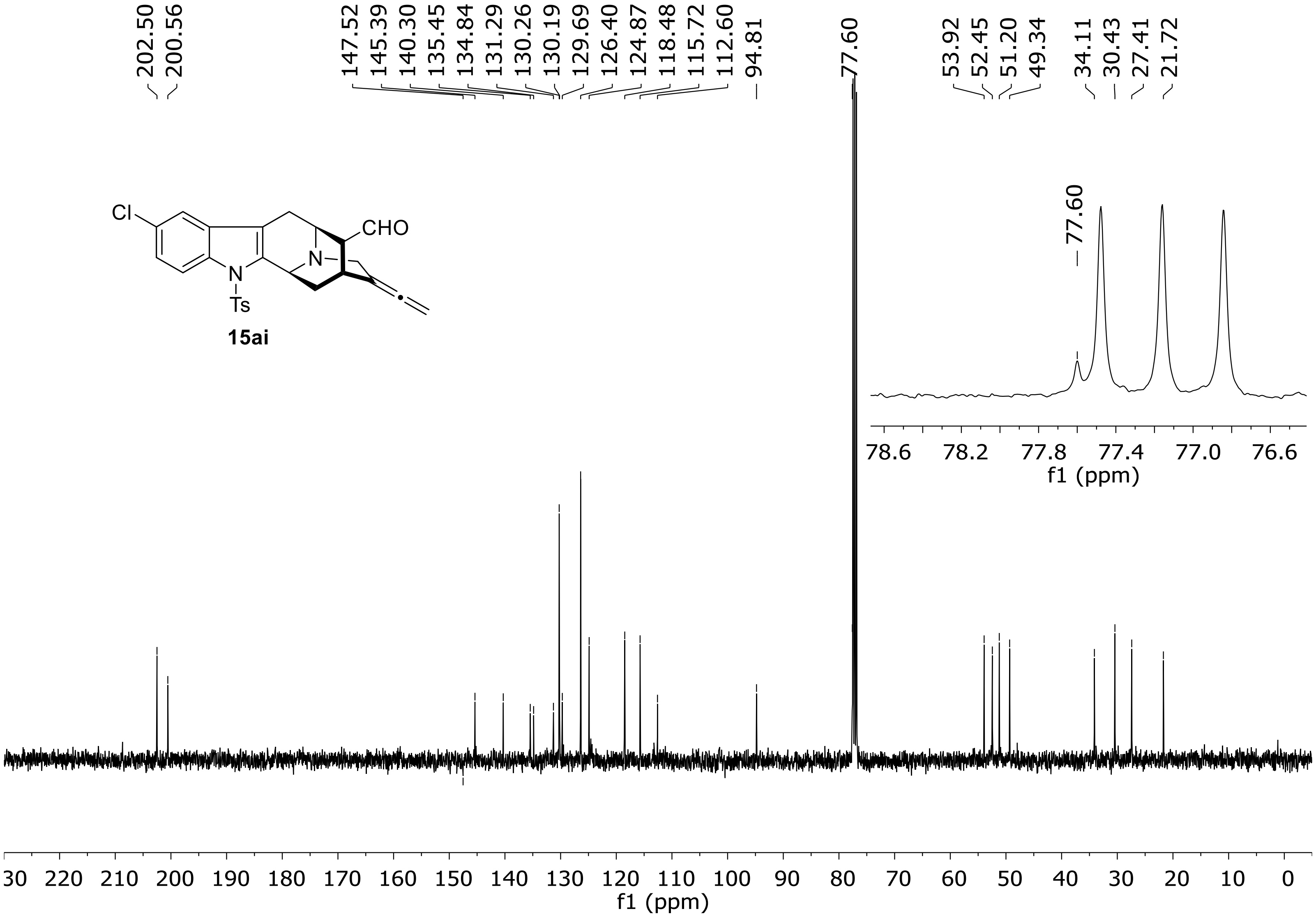
202.50
200.56
147.52
145.39
140.30
135.45
134.84
131.29
130.26
130.19
129.69
126.40
124.87
118.48
115.72
112.60
94.81
77.60
53.92
52.45
51.20
49.34
34.11
30.43
27.41
21.72
Cl
CHO
N
N
Ts
15ai
77.60
78.6 78.2 77.8 77.4 77.0 76.6
f1 (ppm)
30 220 210 200 190 180 170 160 150 140 130 120 110 100 90 80 70 60 50 40 30 20 10 0
f1 (ppm)

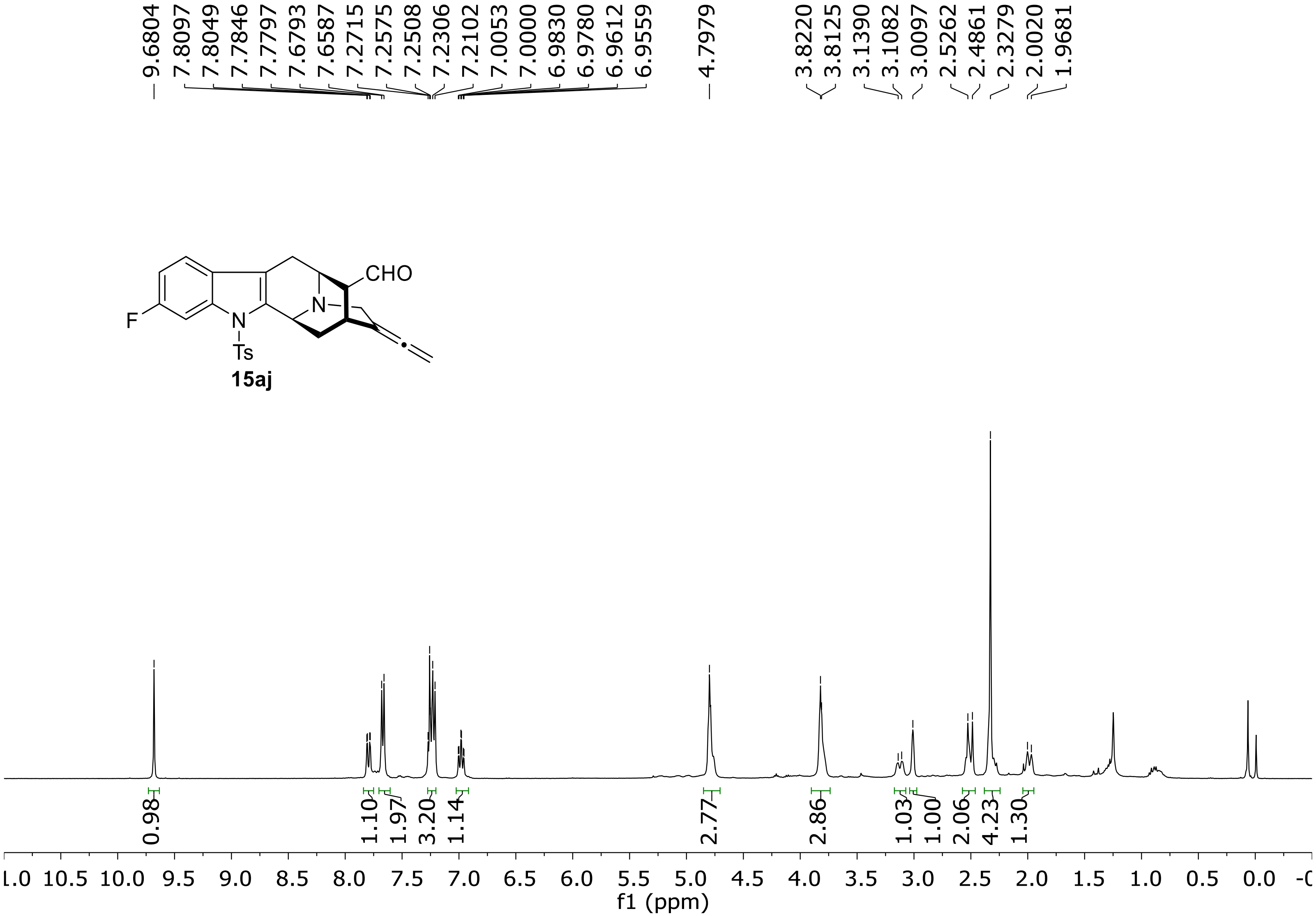
9.6804
7.8097
7.8049
7.7846
7.7797
7.6793
7.6587
7.2715
7.2575
7.2508
7.2306
7.2102
7.0053
7.0000
6.9830
6.9780
6.9612
6.9559
4.7979
3.8220
3.8125
3.1390
3.1082
3.0097
2.5262
2.4861
2.3279
2.0020
1.9681
CHO
F
N
N
Ts
15aj
0.98
1.10
1.97
3.20
1.14
2.77
2.86
1.03
1.00
2.06
4.23
1.30
10.5 10.0 9.5 9.0 8.5 8.0 7.5 7.0 6.5 6.0 5.5 5.0 4.5 4.0 3.5 3.0 2.5 2.0 1.5 1.0 0.5 0.0
f1 (ppm)

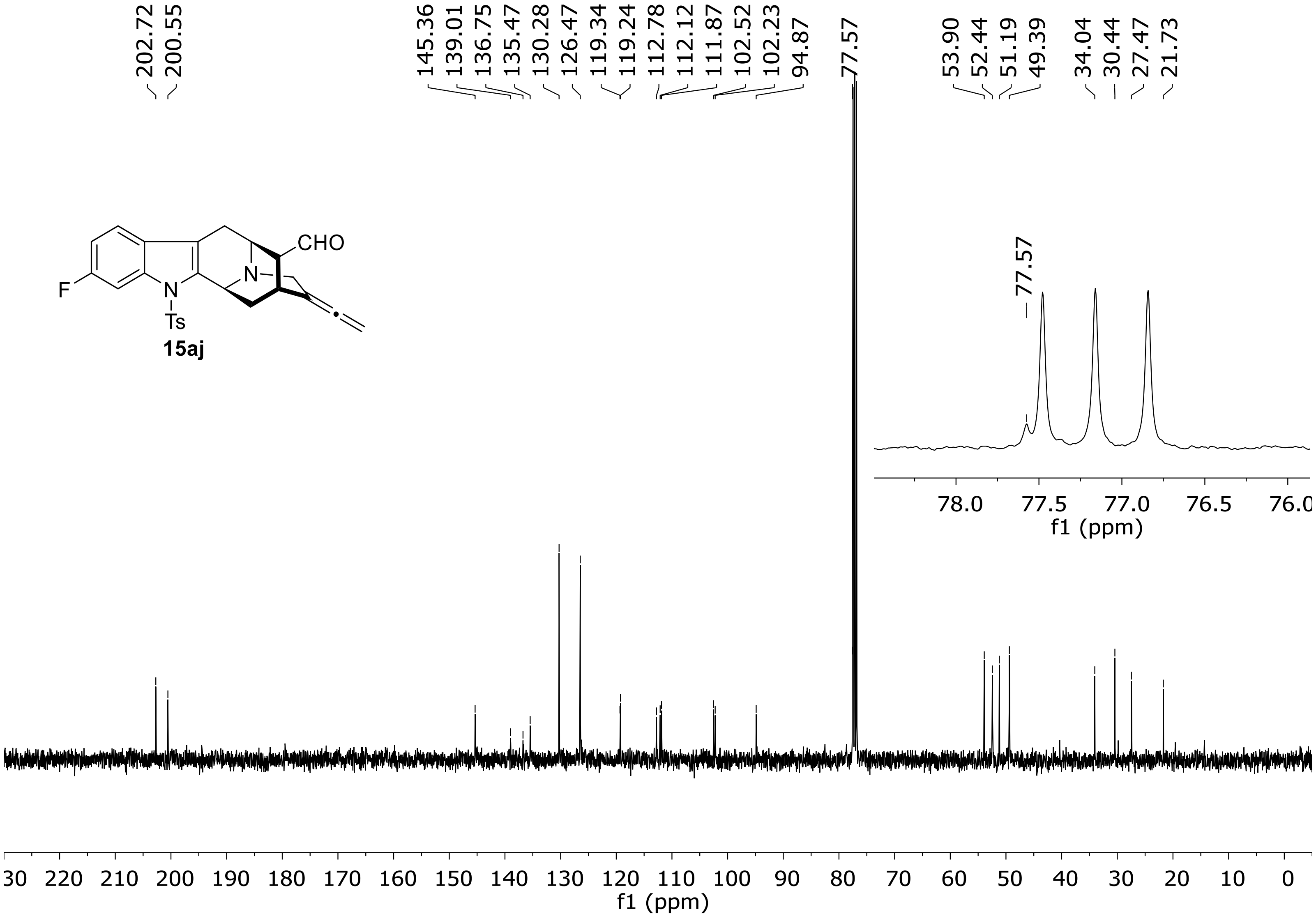

CHO
F
N
Ts
15aj
202.72
200.55
145.36
139.01
136.75
135.47
130.28
126.47
119.34
119.24
112.78
112.12
111.87
102.52
102.23
94.87
77.57
53.90
52.44
51.19
49.39
34.04
30.44
27.47
21.73
77.57
78.0
77.5
77.0
76.5
76.0
f1 (ppm)
30
220
210
200
190
180
170
160
150
140
130
120
110
100
90
80
70
60
50
40
30
20
10
0
f1 (ppm)

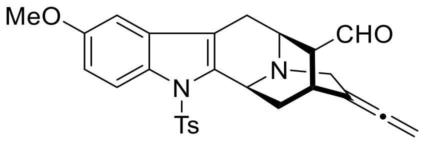
MeO
CHO
N
N
Ts

15al

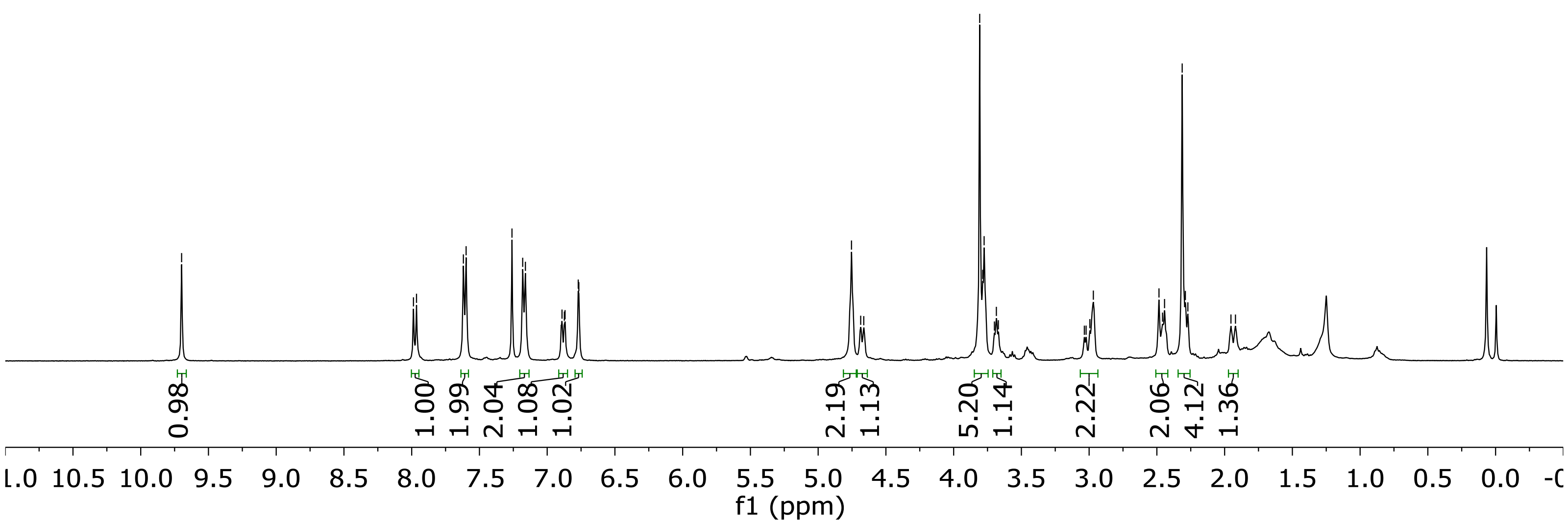
9.6982
7.9870
7.9644
7.6185
7.5983
7.2598
7.1806
7.1607
6.8906
6.8733
6.8681
6.7708
6.7662
4.7539
4.6860
4.6633
3.8077
3.7851
3.7749
3.6990
3.6846
3.6699
3.0352
3.0224
2.9945
2.9688
2.4843
2.4581
2.4434
2.3131
2.2897
2.2712
1.9532
1.9199
0.98
1.00
1.99
2.04
1.08
1.02
2.19
1.13
5.20
1.14
2.22
2.06
4.12
1.36
10.5 10.0 9.5 9.0 8.5 8.0 7.5 7.0 6.5 6.0 5.5 5.0 4.5 4.0 3.5 3.0 2.5 2.0 1.5 1.0 0.5 0.0
f1 (ppm)

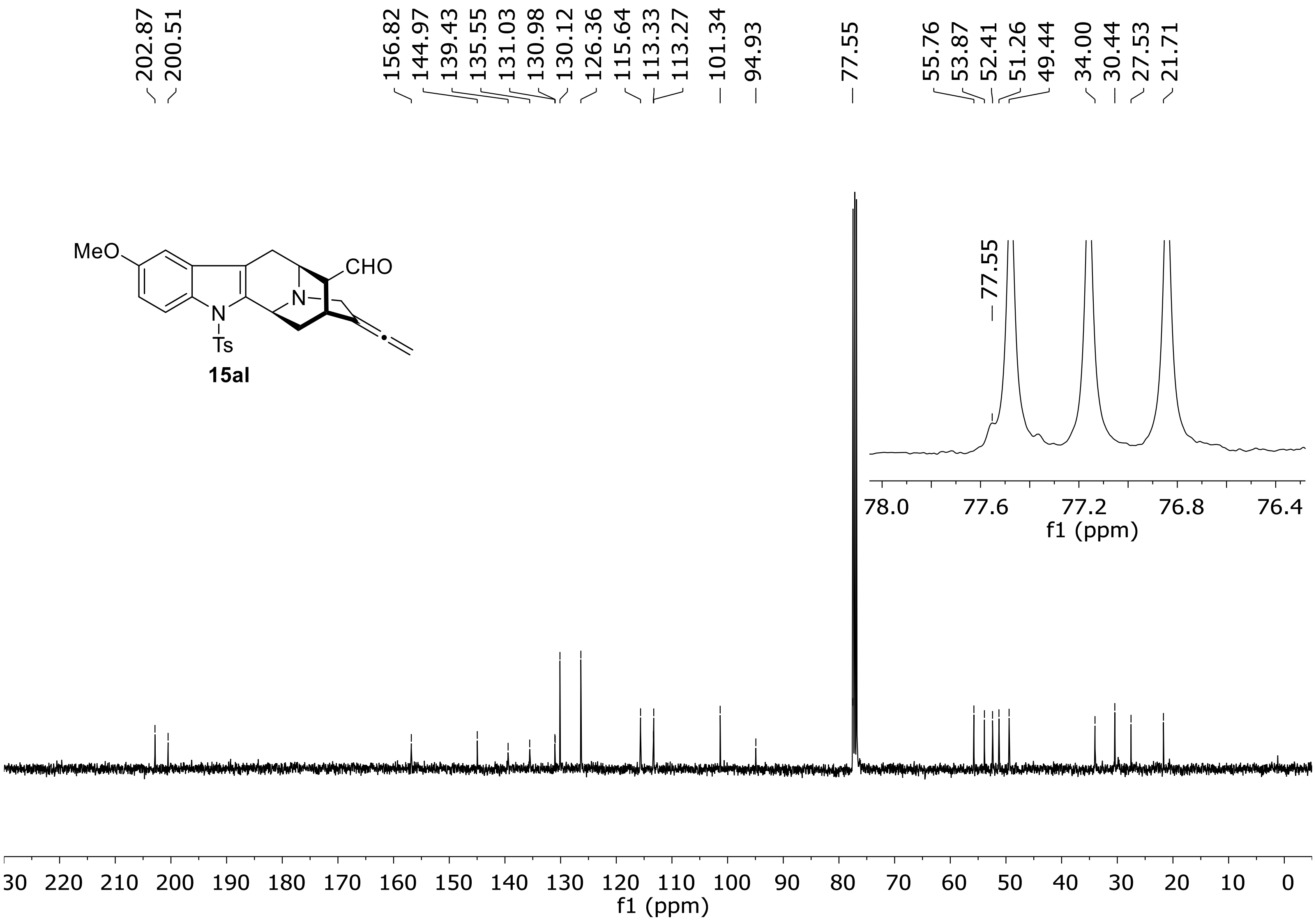
202.87
200.51
156.82
144.97
139.43
135.55
131.03
130.98
130.12
126.36
115.64
113.33
113.27
101.34
94.93
77.55
55.76
53.87
52.41
51.26
49.44
34.00
30.44
27.53
21.71
MeO
CHO
N
N
Ts
15al
77.55
78.0 77.6 77.2 76.8 76.4
f1 (ppm)
30 220 210 200 190 180 170 160 150 140 130 120 110 100 90 80 70 60 50 40 30 20 10 0
f1 (ppm)

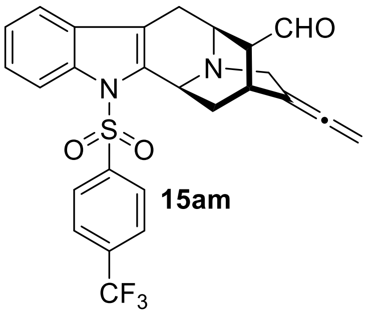
CHO
N
N
S
O
O
CF3

15am

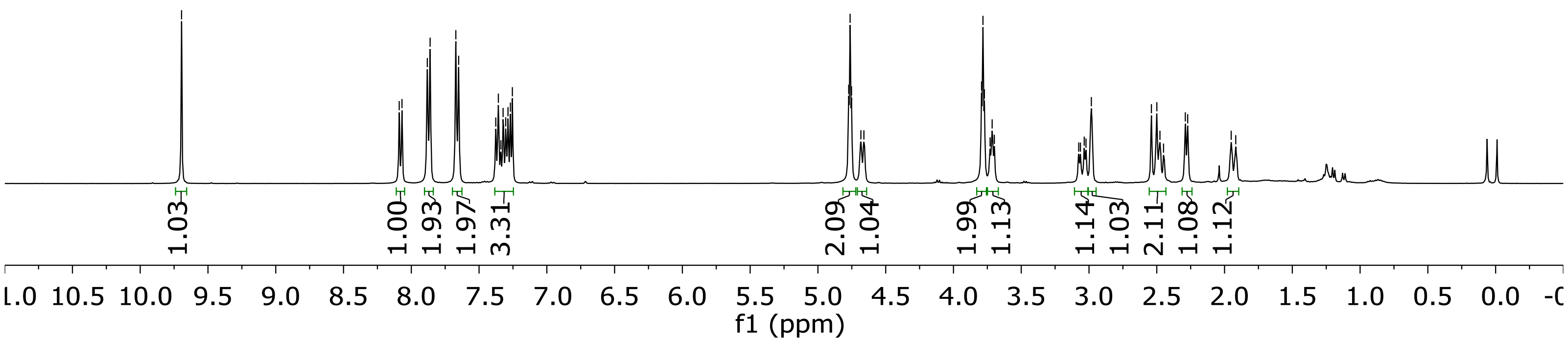
9.6951
8.0891
8.0688
7.8818
7.8612
7.6715
7.6507
7.3767
7.3577
7.3389
7.3229
7.3034
7.2867
7.2688
7.2543
4.7729
4.7626
4.7523
4.6830
4.6607
3.7928
3.7823
3.7719
3.7296
3.7145
3.6985
3.0759
3.0631
3.0353
3.0225
2.9827
2.5395
2.4995
2.4766
2.2896
2.2720
1.9509
1.03
1.00
1.93
1.97
3.31
2.09
1.04
1.99
1.13
1.14
1.03
2.11
1.08
1.12
10.5 10.0 9.5 9.0 8.5 8.0 7.5 7.0 6.5 6.0 5.5 5.0 4.5 4.0 3.5 3.0 2.5 2.0 1.5 1.0 0.5 0.0
f1 (ppm)

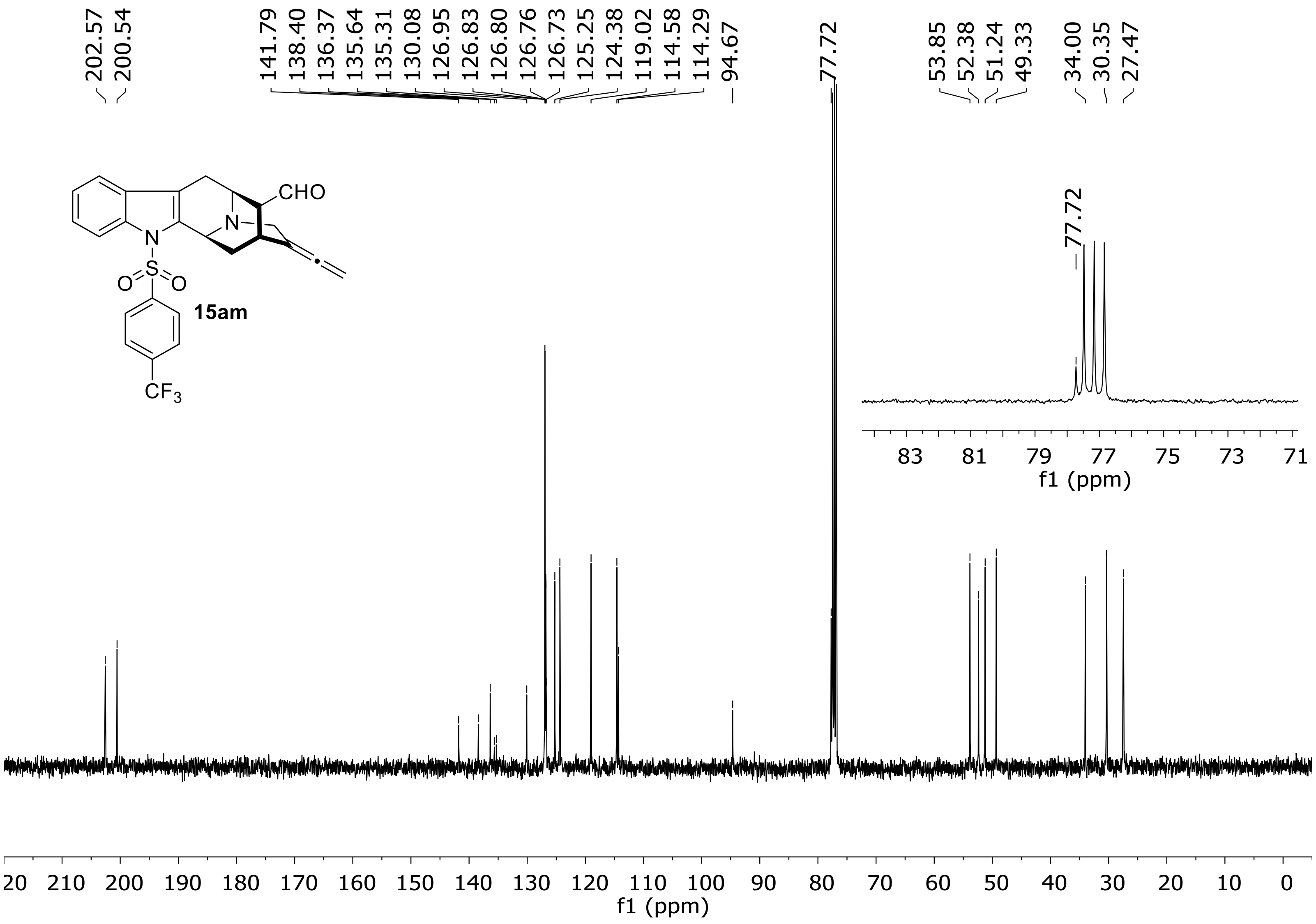
202.57
200.54
141.79
138.40
136.37
135.64
135.31
130.08
126.95
126.83
126.80
126.76
126.73
125.25
124.38
119.02
114.58
114.29
94.67
77.72
53.85
52.38
51.24
49.33
34.00
30.35
27.47
CHO
N
N
O
S
O
CF3
15am
77.72
83
81
79
77
75
73
71
f1 (ppm)
20
210
200
190
180
170
160
150
140
130
120
110
100
90
80
70
60
50
40
30
20
10
0
f1 (ppm)

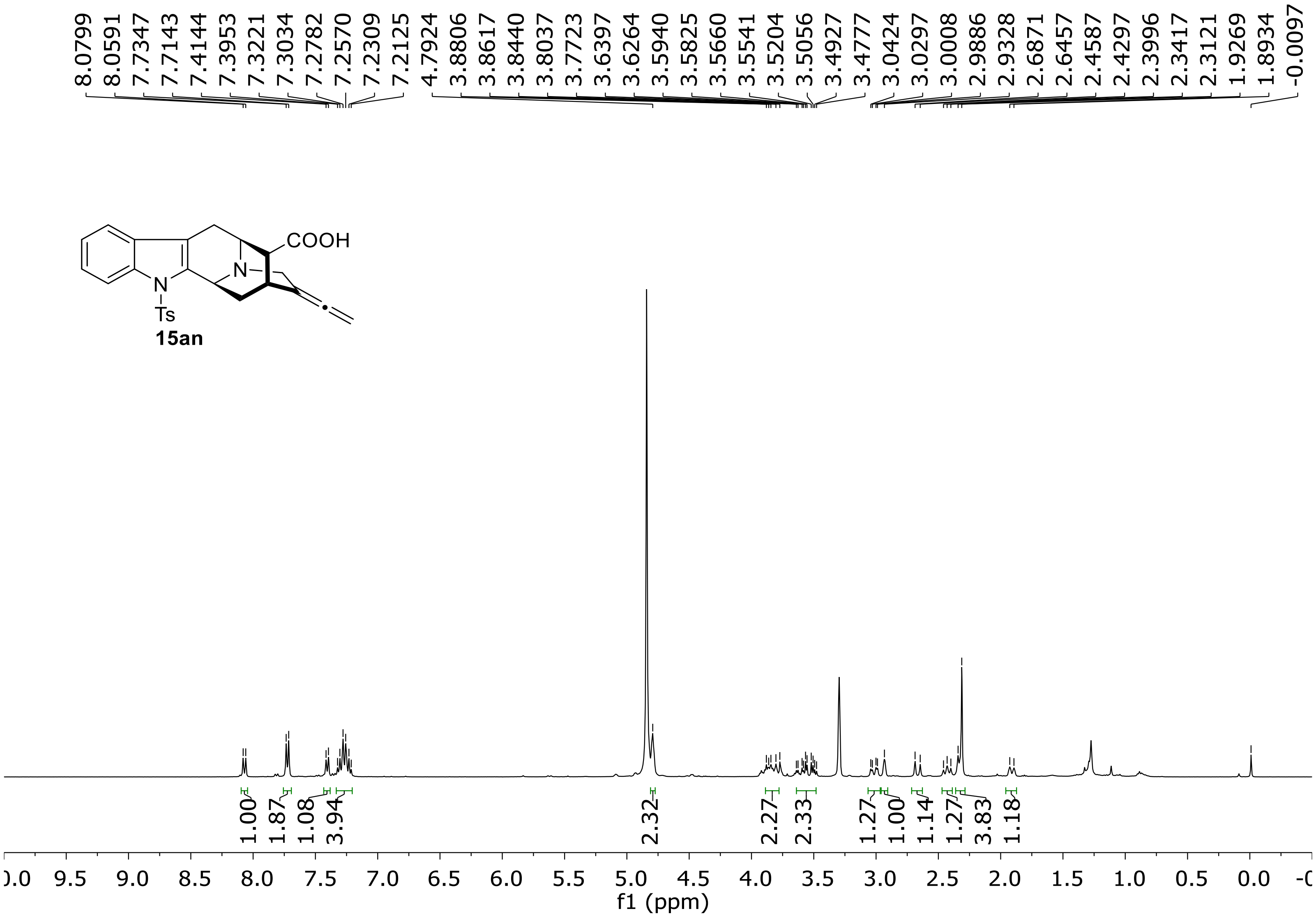
8.0799 8.0591 7.7347 7.7143 7.4144 7.3953 7.3221 7.3034 7.2782 7.2570 7.2309 7.2125 4.7924 3.8806 3.8617 3.8440 3.8037 3.7723 3.6397 3.6264 3.5940 3.5825 3.5660 3.5541 3.5204 3.5056 3.4927 3.4777 3.0424 3.0297 3.0008 2.9886 2.9328 2.6871 2.6457 2.4587 2.4297 2.3996 2.3417 2.3121 1.9269 1.8934 -0.0097
COOH
N
N
Ts
15an
1.00 1.87 1.08 3.94 2.32 2.27 2.33 1.27 1.00 1.14 1.27 3.83 1.18
f1 (ppm)

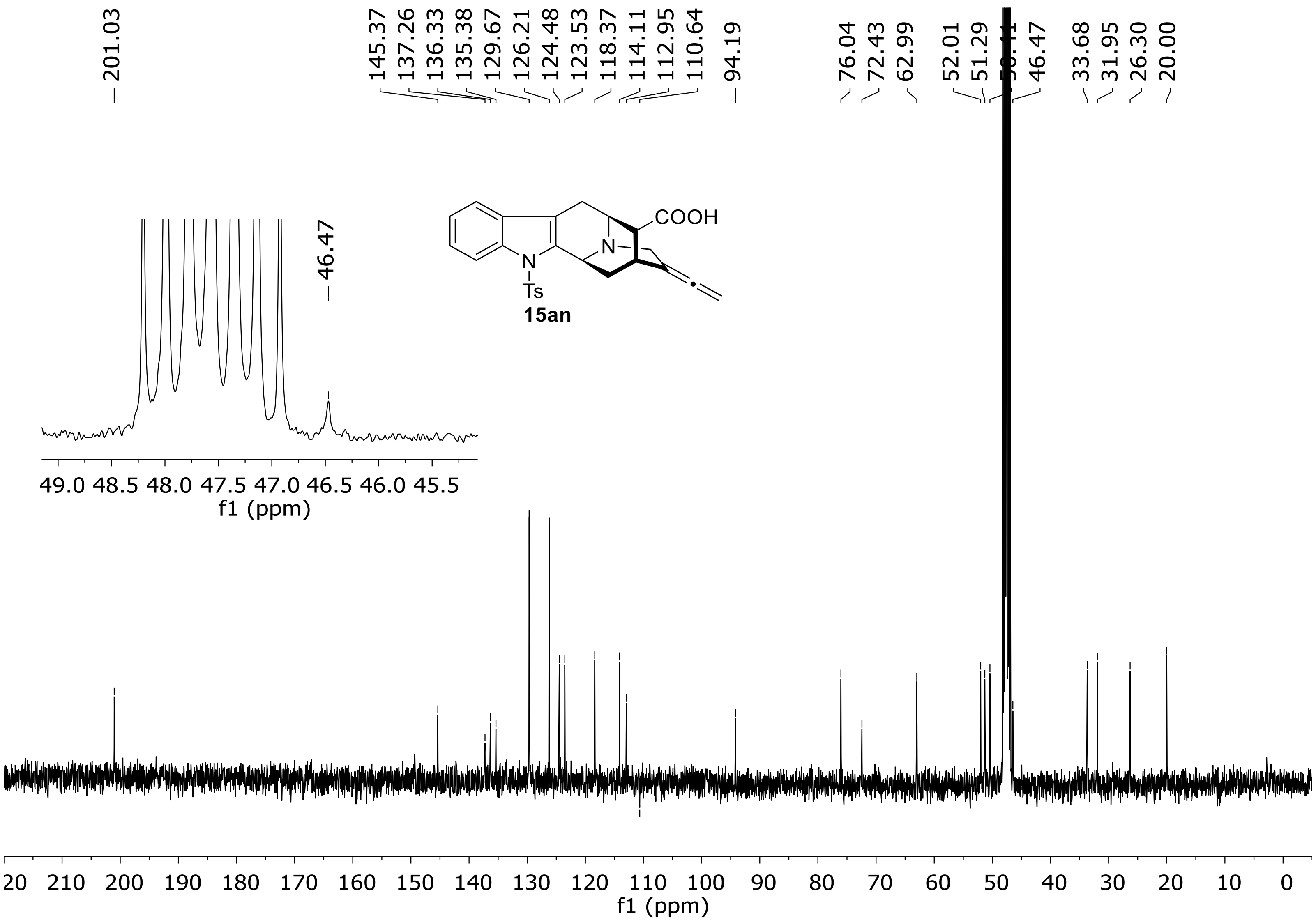
201.03
145.37
137.26
136.33
135.38
129.67
126.21
124.48
123.53
118.37
114.11
112.95
110.64
94.19
76.04
72.43
62.99
52.01
51.29
46.47
33.68
31.95
26.30
20.00
COOH
N
N
Ts
15an
46.47
49.0 48.5 48.0 47.5 47.0 46.5 46.0 45.5
f1 (ppm)
20 210 200 190 180 170 160 150 140 130 120 110 100 90 80 70 60 50 40 30 20 10 0
f1 (ppm)

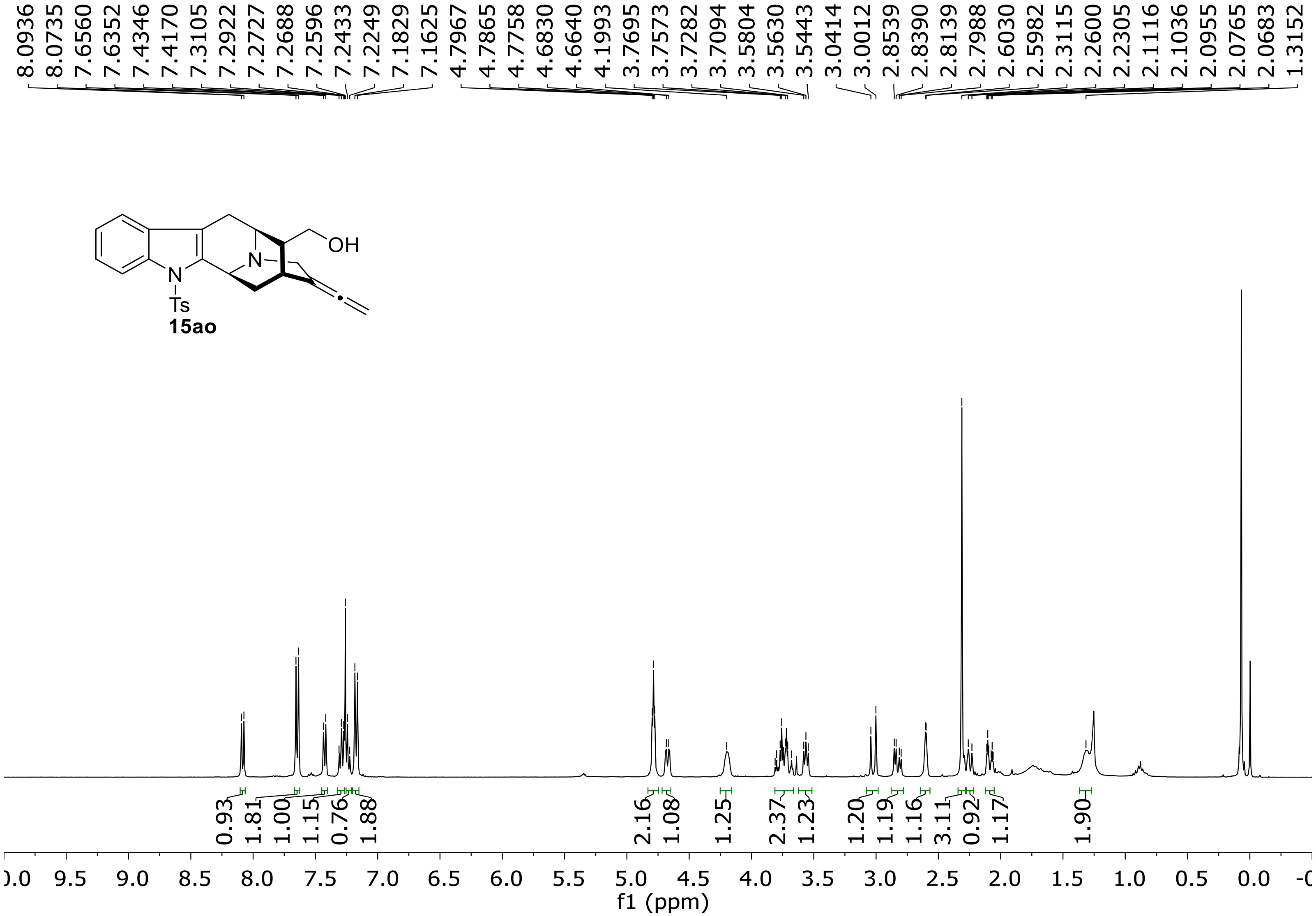
8.0936
8.0735
7.6560
7.6352
7.4346
7.4170
7.3105
7.2922
7.2727
7.2688
7.2596
7.2433
7.2249
7.1829
7.1625
4.7967
4.7865
4.7758
4.6830
4.6640
4.1993
3.7695
3.7573
3.7282
3.7094
3.5804
3.5630
3.5443
3.0414
3.0012
2.8539
2.8390
2.8139
2.7988
2.6030
2.5982
2.3115
2.2600
2.2305
2.1116
2.1036
2.0955
2.0765
2.0683
1.3152
OH
N
N
Ts
15ao
0.93
1.81
1.00
1.15
0.76
1.88
2.16
1.08
1.25
2.37
1.23
1.20
1.19
1.16
3.11
0.92
1.17
1.90
9.5
9.0
8.5
8.0
7.5
7.0
6.5
6.0
5.5
5.0
4.5
4.0
3.5
3.0
2.5
2.0
1.5
1.0
0.5
0.0
f1 (ppm)

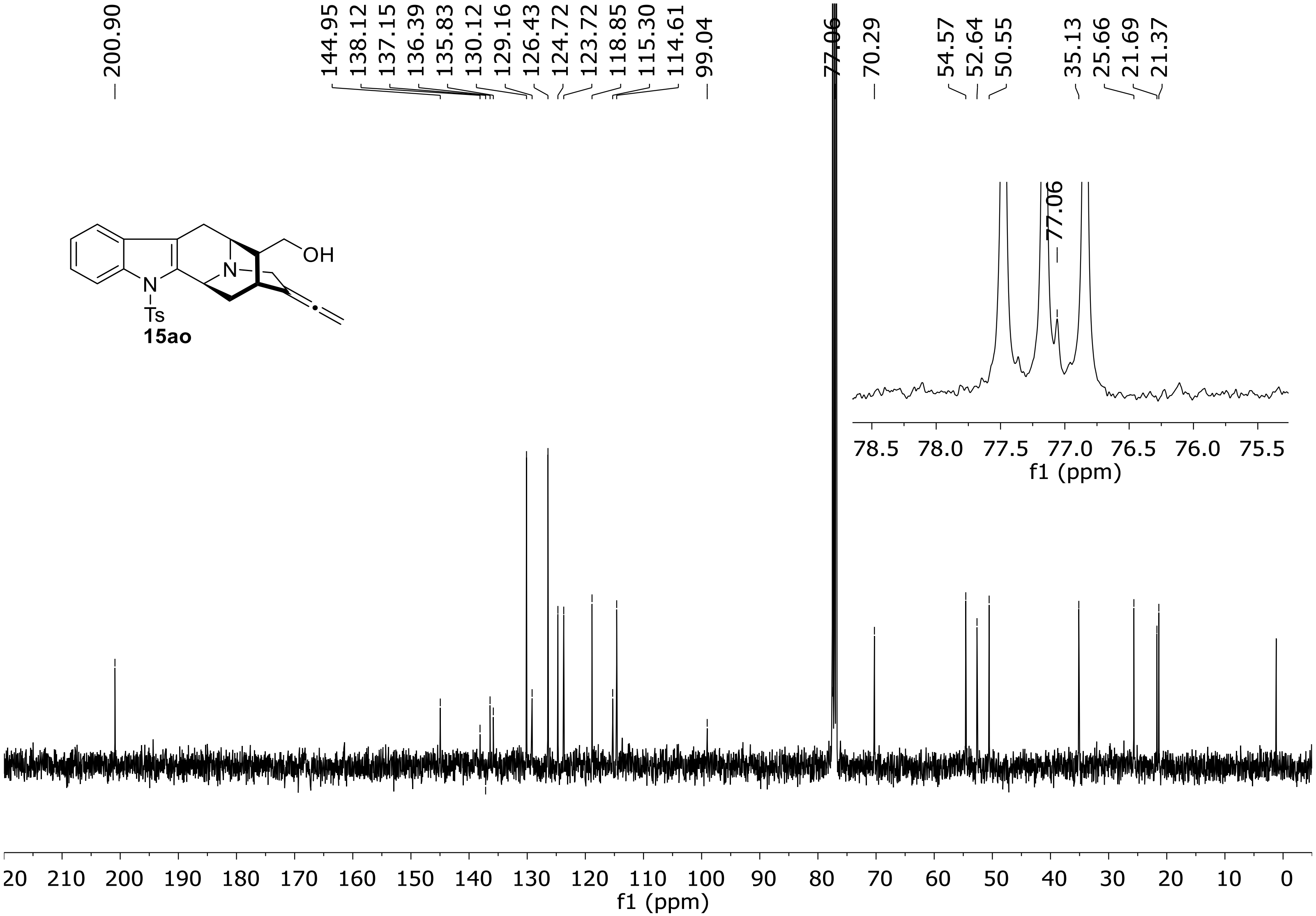
200.90
144.95
138.12
137.15
136.39
135.83
130.12
129.16
126.43
124.72
123.72
118.85
115.30
114.61
99.04
77.06
70.29
54.57
52.64
50.55
35.13
25.66
21.69
21.37
OH
N
N
Ts
15ao
77.06
78.5 78.0 77.5 77.0 76.5 76.0 75.5
f1 (ppm)
20 210 200 190 180 170 160 150 140 130 120 110 100 90 80 70 60 50 40 30 20 10 0
f1 (ppm)

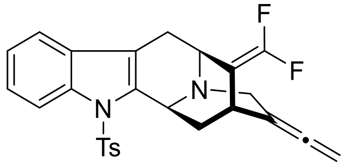
F
F
N
N
Ts

15ap

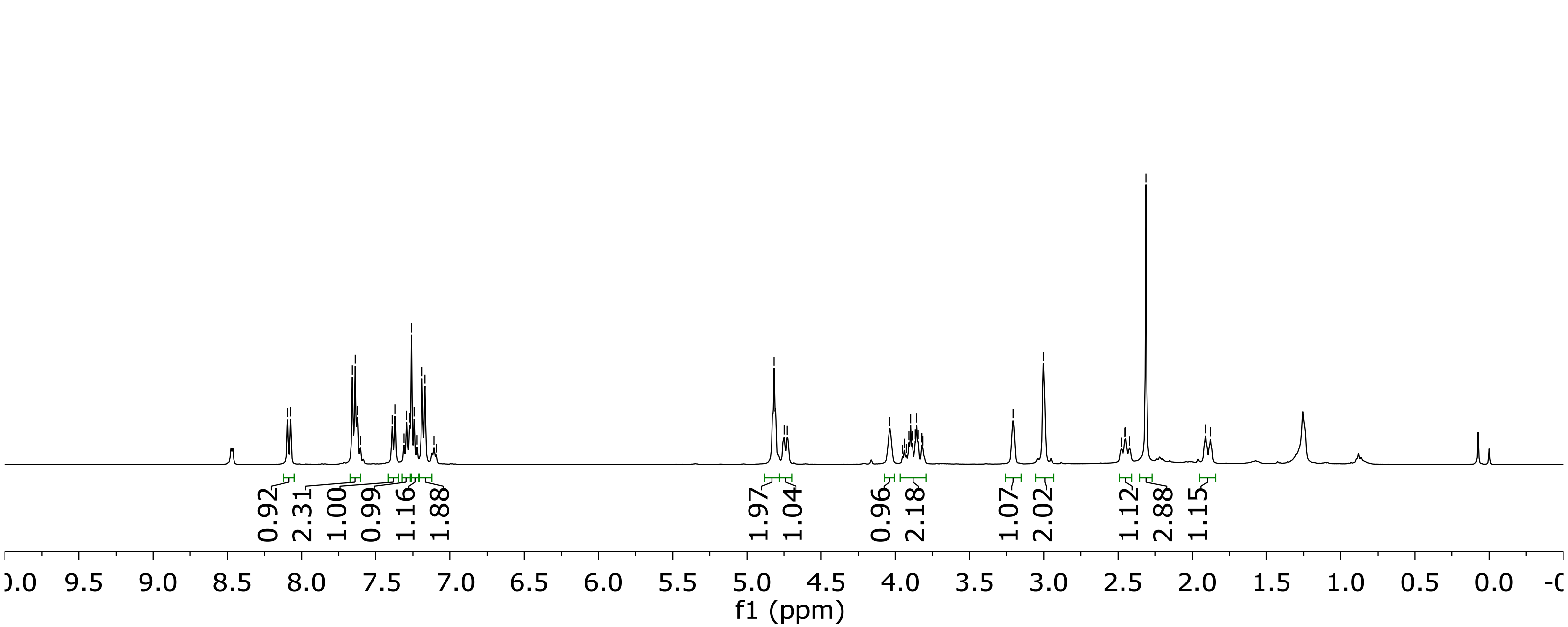
8.0941
8.0739
7.6580
7.6375
7.6236
7.6035
7.3901
7.3714
7.3095
7.2916
7.2707
7.2598
7.2419
7.2239
7.1885
7.1683
7.1081
7.0925
4.8162
4.7486
4.7297
4.0372
3.9510
3.9392
3.9272
3.9095
3.8977
3.8860
3.8654
3.8558
3.8464
3.8214
3.8151
3.2058
3.0036
2.4784
2.4522
2.4477
2.4215
2.3130
1.9110
1.8783
0.92
2.31
1.00
0.99
1.16
1.88
1.97
1.04
0.96
2.18
1.07
2.02
1.12
2.88
1.15
9.5
9.0
8.5
8.0
7.5
7.0
6.5
6.0
5.5
5.0
4.5
4.0
3.5
3.0
2.5
2.0
1.5
1.0
0.5
0.0
f1 (ppm)

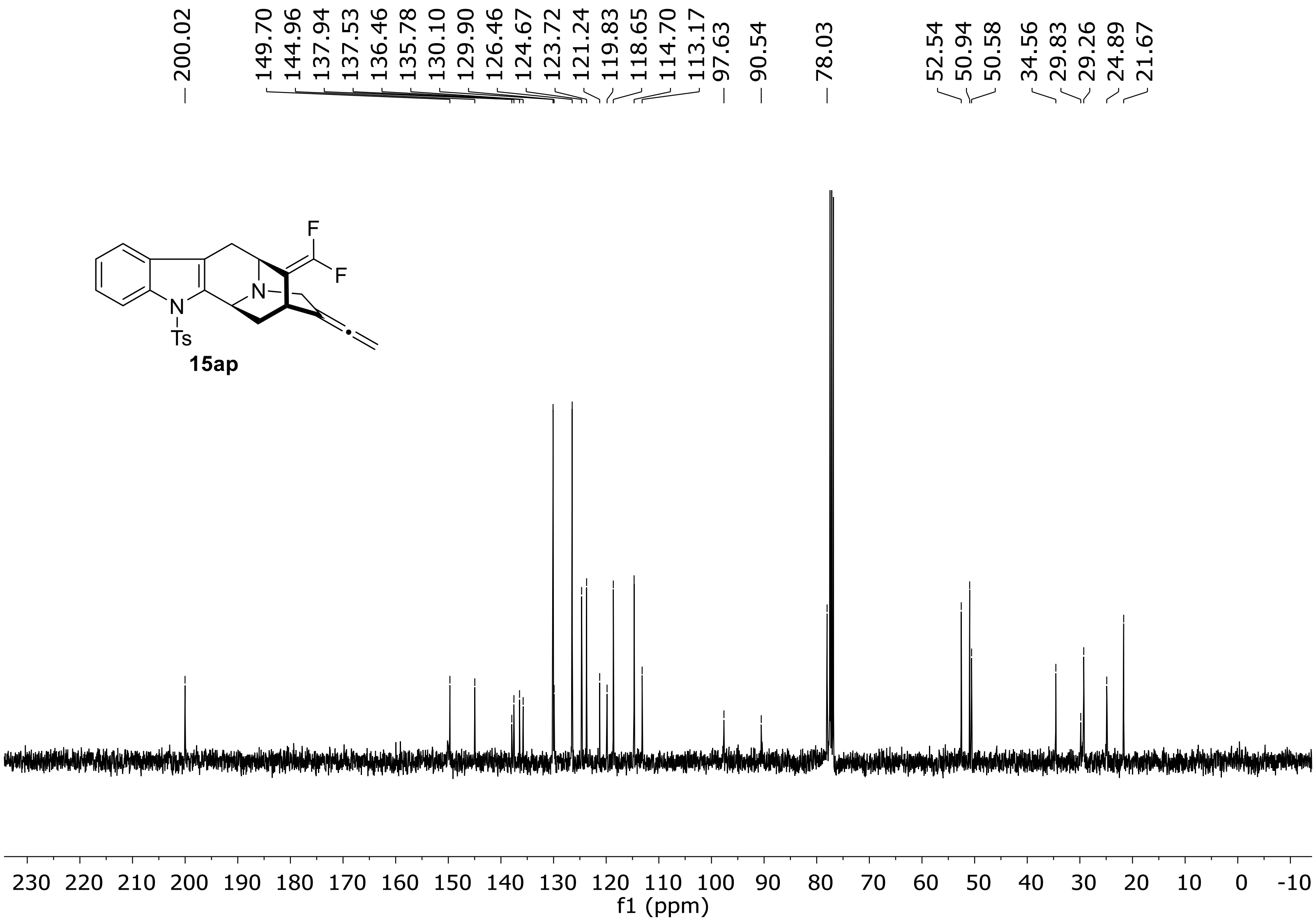
200.02
149.70
144.96
137.94
137.53
136.46
135.78
130.10
129.90
126.46
124.67
123.72
121.24
119.83
118.65
114.70
113.17
97.63
90.54
78.03
52.54
50.94
50.58
34.56
29.83
29.26
24.89
21.67
F
F
N
N
Ts
15ap
230 220 210 200 190 180 170 160 150 140 130 120 110 100 90 80 70 60 50 40 30 20 10 0 -10
f1 (ppm)

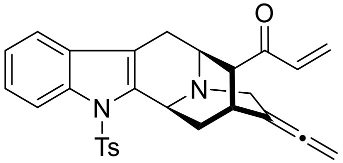
O
N
N
Ts

15aq

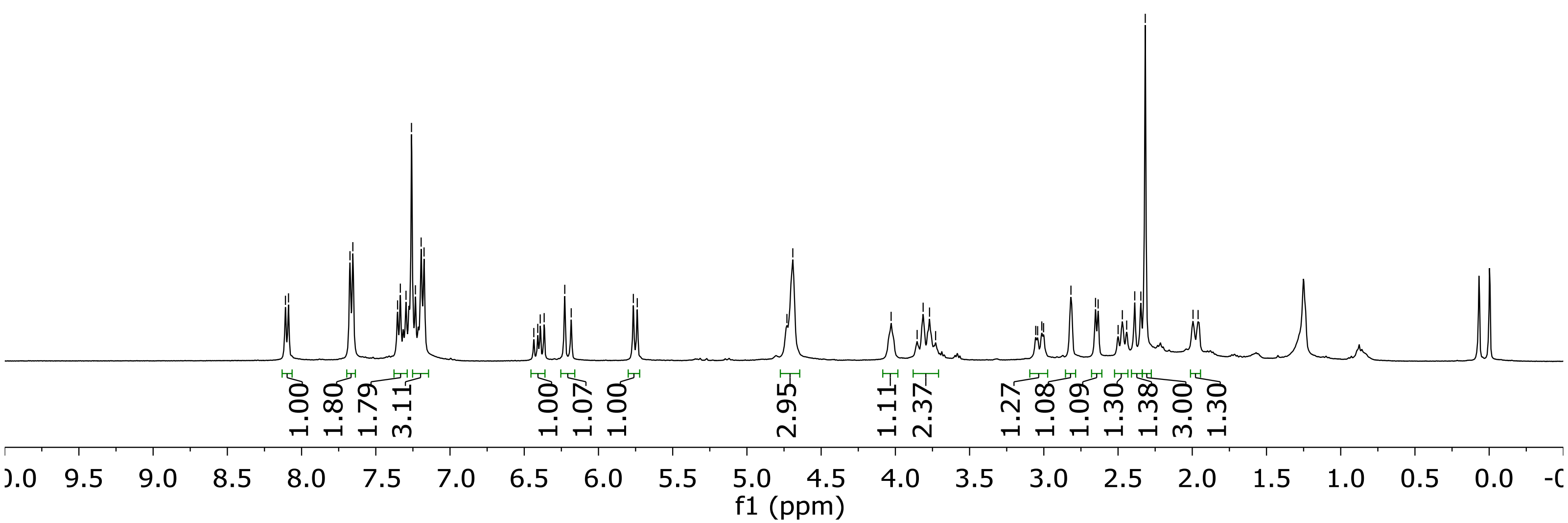
8.1086
8.0880
7.6739
7.6546
7.3533
7.3347
7.2965
7.2595
7.2331
7.1946
7.1750
6.4357
6.4090
6.3923
6.3658
6.2271
6.1835
5.7650
5.7387
4.7306
4.6909
4.0286
3.8534
3.8130
3.7699
3.7290
3.0544
3.0417
3.0138
3.0017
2.8173
2.6519
2.6342
2.4996
2.4710
2.4419
2.3876
2.3464
2.3166
1.9949
1.9607
1.00
1.80
1.79
3.11
1.00
1.07
1.00
2.95
1.11
2.37
1.27
1.08
1.09
1.30
1.38
3.00
1.30
9.5
9.0
8.5
8.0
7.5
7.0
6.5
6.0
5.5
5.0
4.5
4.0
3.5
3.0
2.5
2.0
1.5
1.0
0.5
0.0
f1 (ppm)

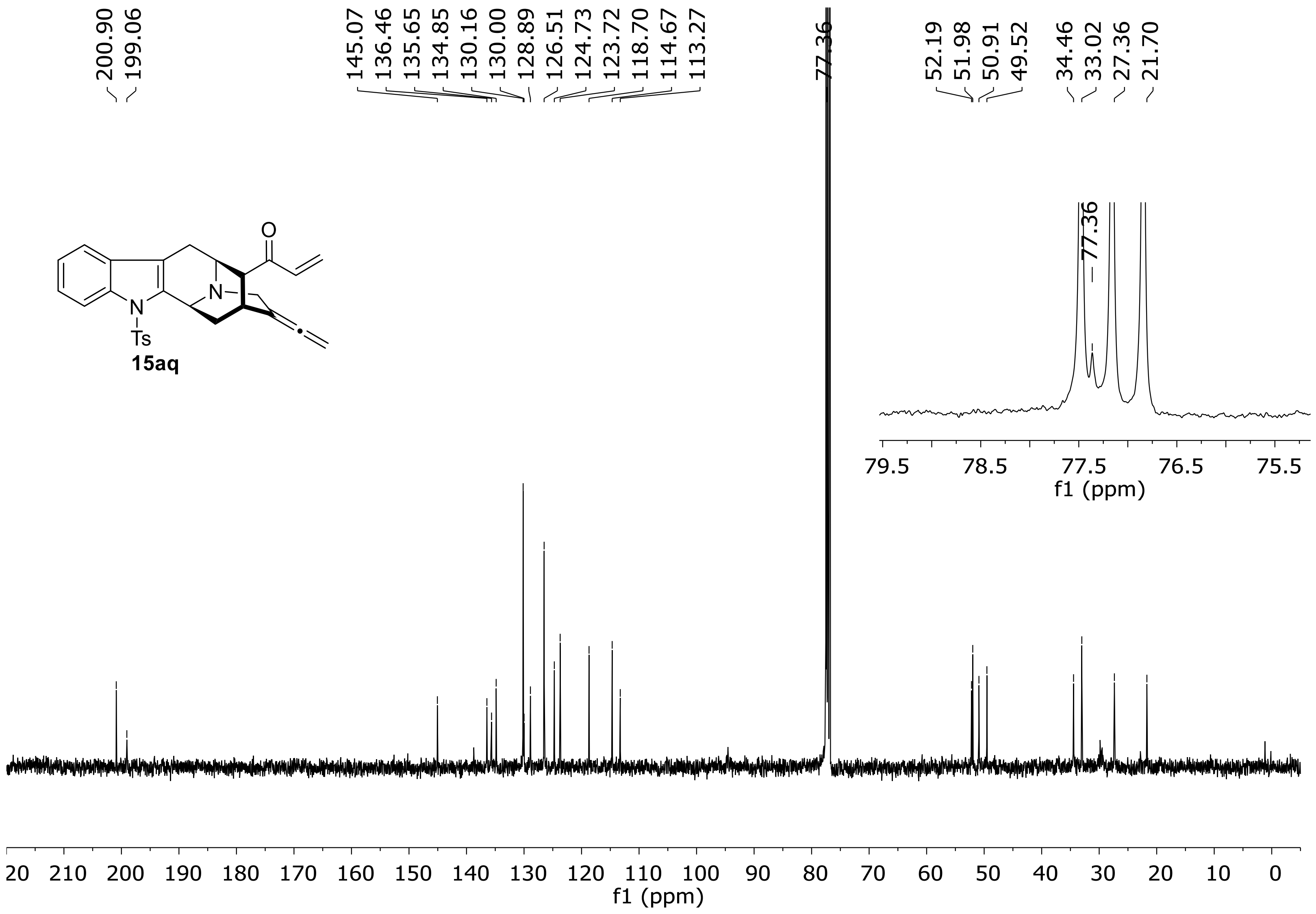
200.90
199.06
145.07
136.46
135.65
134.85
130.16
130.00
128.89
126.51
124.73
123.72
118.70
114.67
113.27
77.36
52.19
51.98
50.91
49.52
34.46
33.02
27.36
21.70
O
N
N
Ts
15aq
77.36
79.5
78.5
77.5
76.5
75.5
f1 (ppm)
20
210
200
190
180
170
160
150
140
130
120
110
100
90
80
70
60
50
40
30
20
10
0
f1 (ppm)

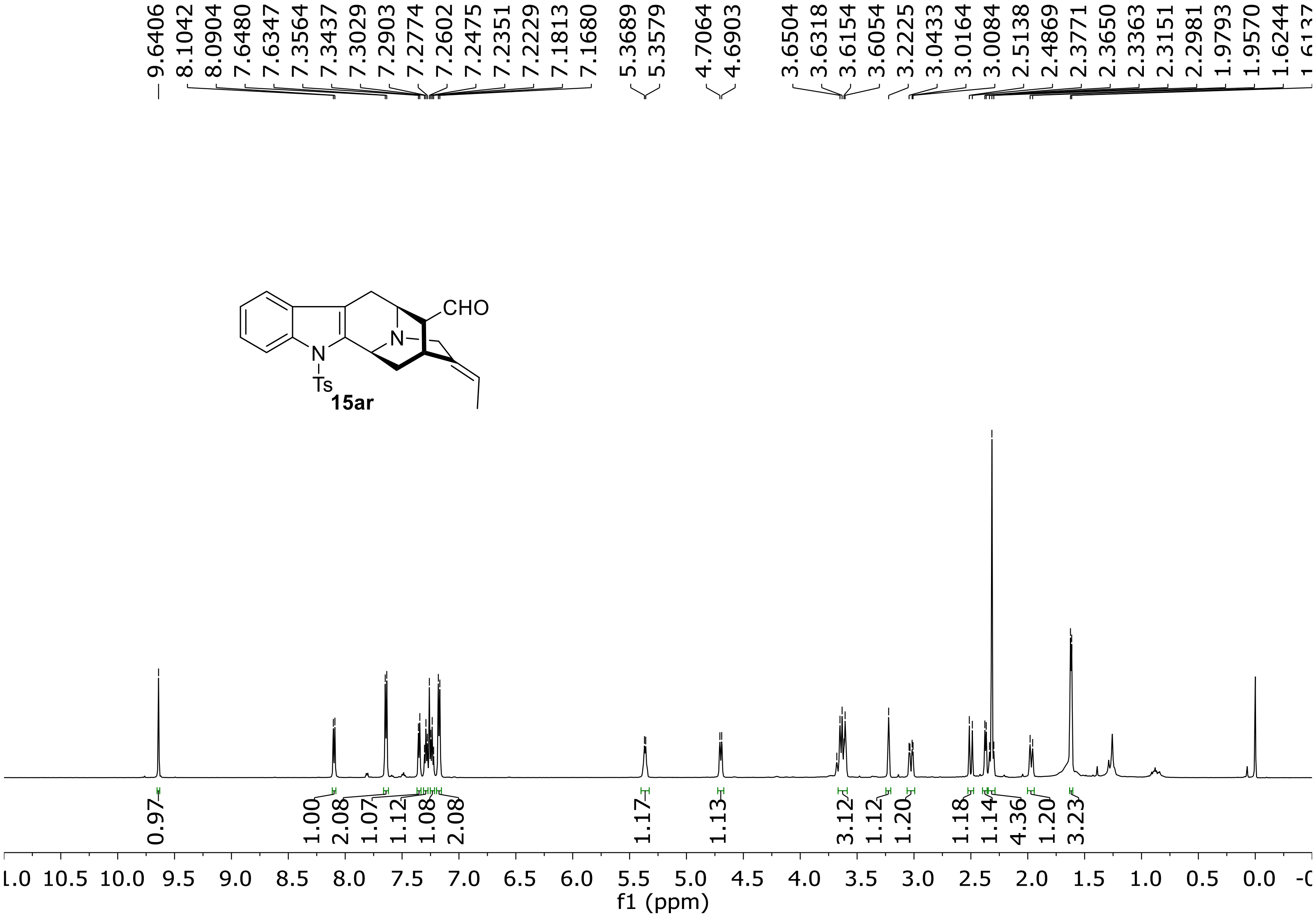

9.6406
8.1042
8.0904
7.6480
7.6347
7.3564
7.3437
7.3029
7.2903
7.2774
7.2602
7.2475
7.2351
7.2229
7.1813
7.1680
5.3689
5.3579
4.7064
4.6903
3.6504
3.6318
3.6154
3.6054
3.2225
3.0433
3.0164
3.0084
2.5138
2.4869
2.3771
2.3650
2.3363
2.3151
2.2981
1.9793
1.9570
1.6244
CHO
N
N
Ts
15ar
0.97
1.00
2.08
1.07
1.12
1.08
2.08
1.17
1.13
3.12
1.12
1.20
1.18
1.14
4.36
1.20
3.23
10.5 10.0 9.5 9.0 8.5 8.0 7.5 7.0 6.5 6.0 5.5 5.0 4.5 4.0 3.5 3.0 2.5 2.0 1.5 1.0 0.5 0.0
f1 (ppm)

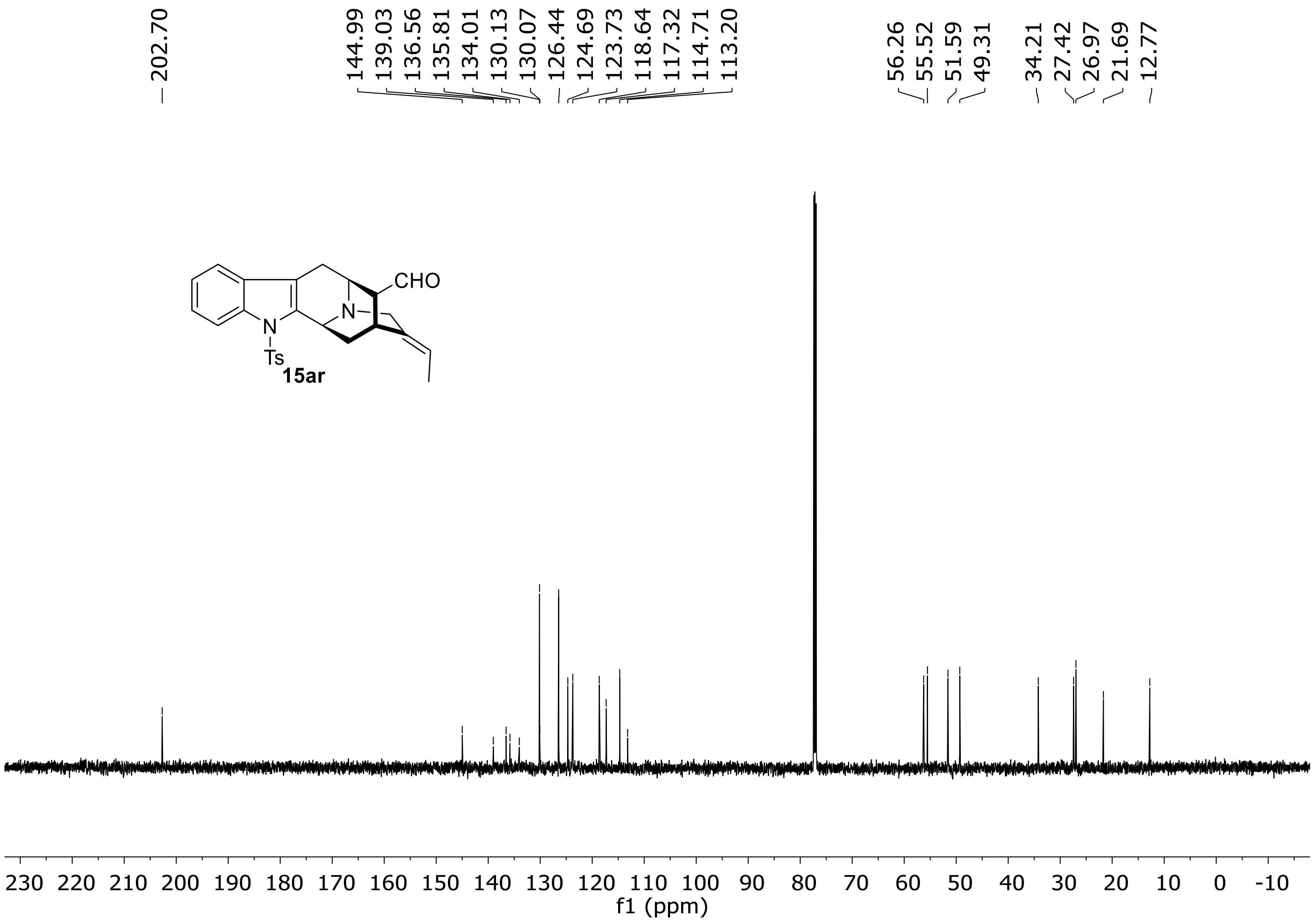
202.70
144.99
139.03
136.56
135.81
134.01
130.13
130.07
126.44
124.69
123.73
118.64
117.32
114.71
113.20
56.26
55.52
51.59
49.31
34.21
27.42
26.97
21.69
12.77
CHO
N
N
Ts
15ar
230 220 210 200 190 180 170 160 150 140 130 120 110 100 90 80 70 60 50 40 30 20 10 0 -10
f1 (ppm)

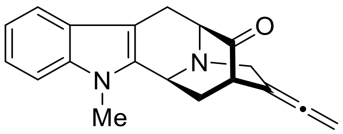
O
N
N
Me

14aa

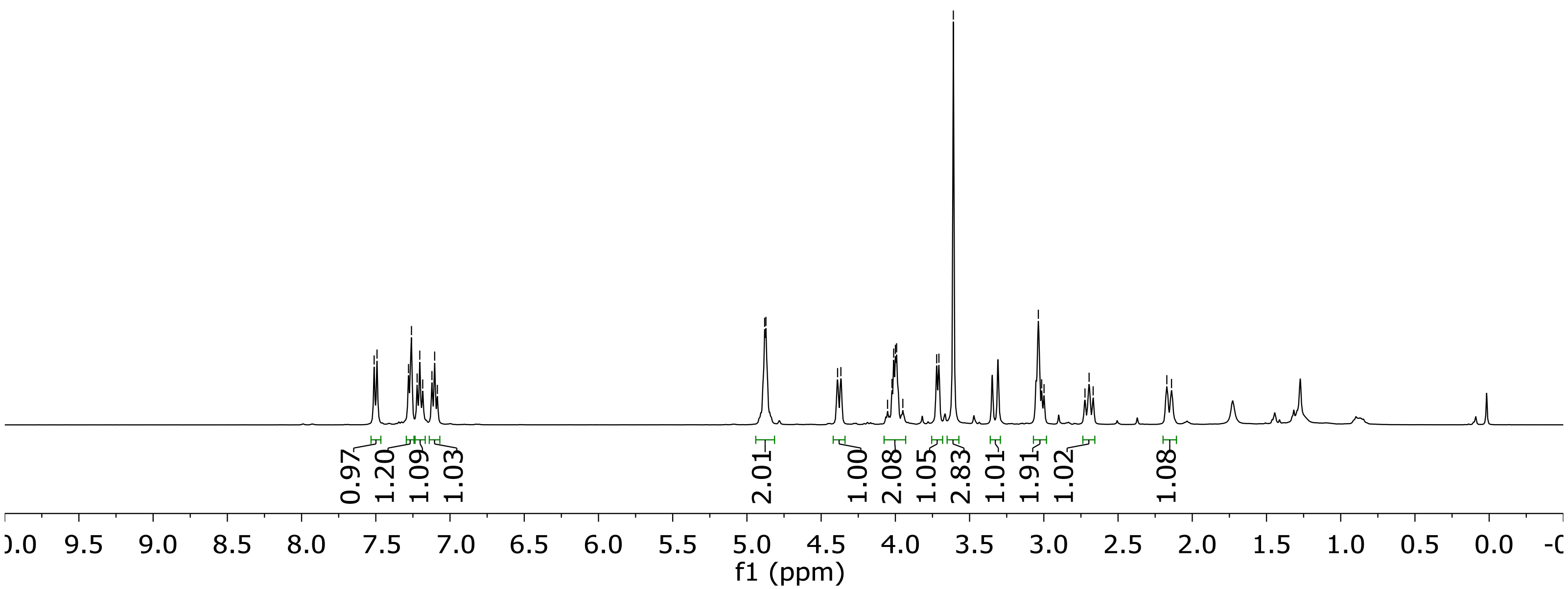
7.5111
7.4918
7.2795
7.2597
7.2216
7.2038
7.1839
7.1218
7.1034
7.0852
4.8806
4.8719
4.3893
4.3671
4.0527
4.0228
4.0112
3.9997
3.9915
3.9498
3.7219
3.7067
3.6094
3.0369
3.0145
2.9986
2.7227
2.6951
2.6678
2.1714
2.1398
0.97
1.20
1.09
1.03
2.01
1.00
2.08
1.05
2.83
1.01
1.91
1.02
1.08
9.5
9.0
8.5
8.0
7.5
7.0
6.5
6.0
5.5
5.0
4.5
4.0
3.5
3.0
2.5
2.0
1.5
1.0
0.5
0.0
f1 (ppm)

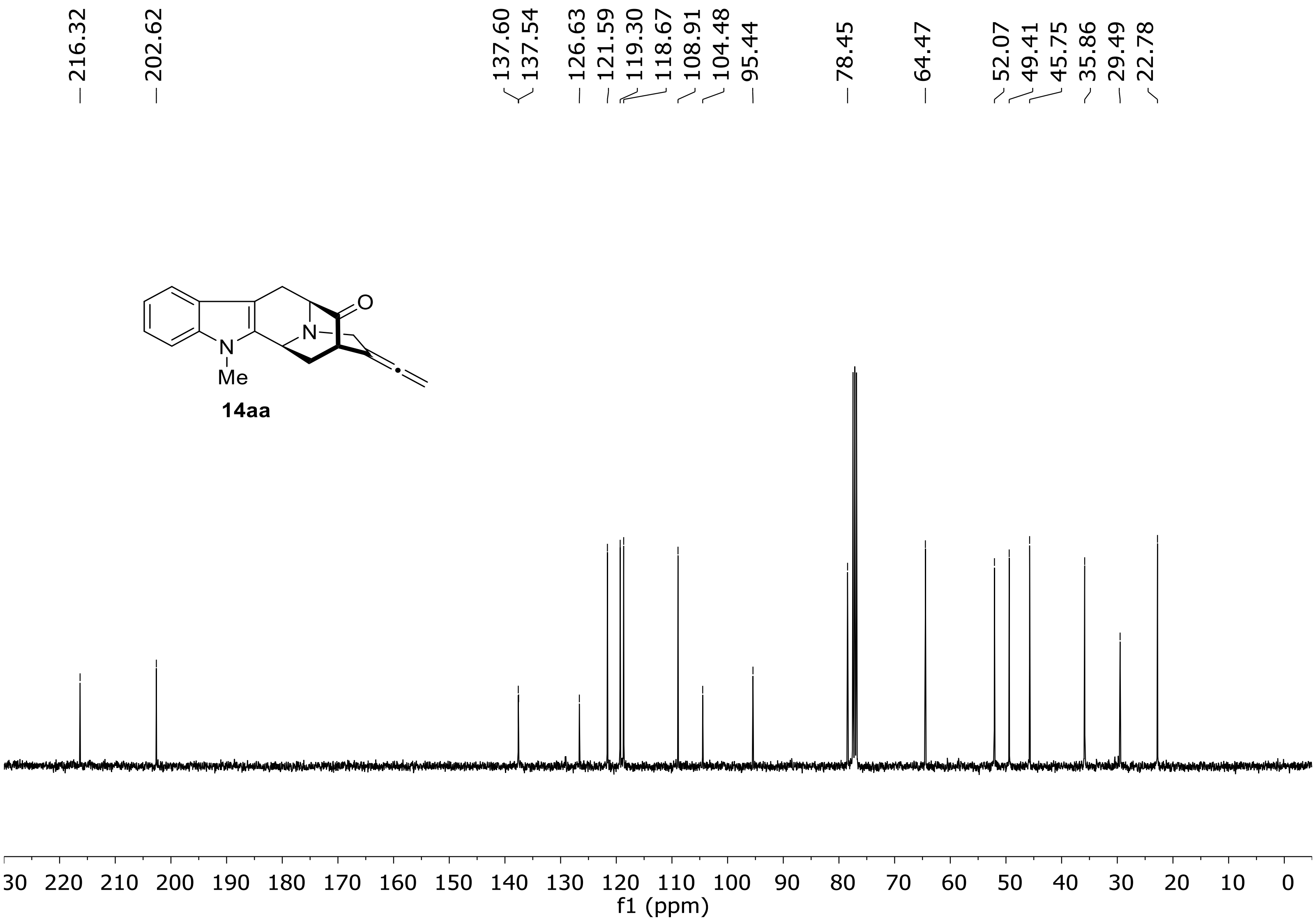
216.32
202.62
137.60
137.54
126.63
121.59
119.30
118.67
108.91
104.48
95.44
78.45
64.47
52.07
49.41
45.75
35.86
29.49
22.78
O
N
N
Me
14aa
30 220 210 200 190 180 170 160 150 140 130 120 110 100 90 80 70 60 50 40 30 20 10 0
f1 (ppm)

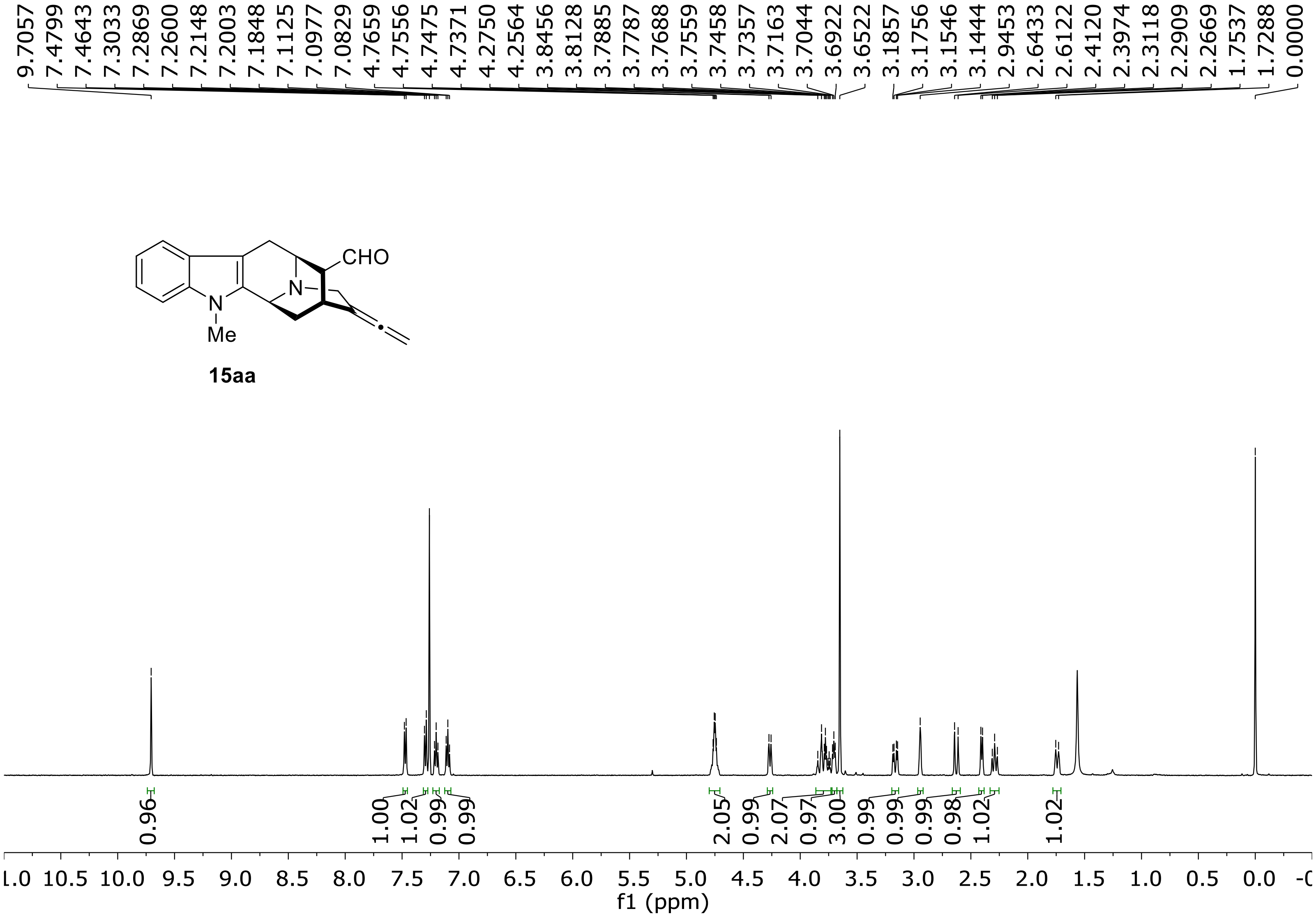

9.7057
7.4799
7.4643
7.3033
7.2869
7.2600
7.2148
7.2003
7.1848
7.1125
7.0977
7.0829
4.7659
4.7556
4.7475
4.7371
4.2750
4.2564
3.8456
3.8128
3.7885
3.7787
3.7688
3.7559
3.7458
3.7357
3.7163
3.7044
3.6922
3.6522
3.1857
3.1756
3.1546
3.1444
2.9453
2.6433
2.6122
2.4120
2.3974
2.3118
2.2909
2.2669
1.7537
1.7288
0.0000
CHO
N
N
Me
15aa
0.96
1.00
1.02
0.99
0.99
2.05
0.99
2.07
0.97
3.00
0.99
0.99
0.99
0.98
1.02
1.02
f1 (ppm)

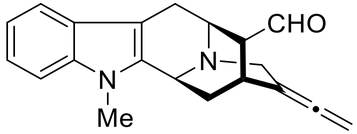

15aa

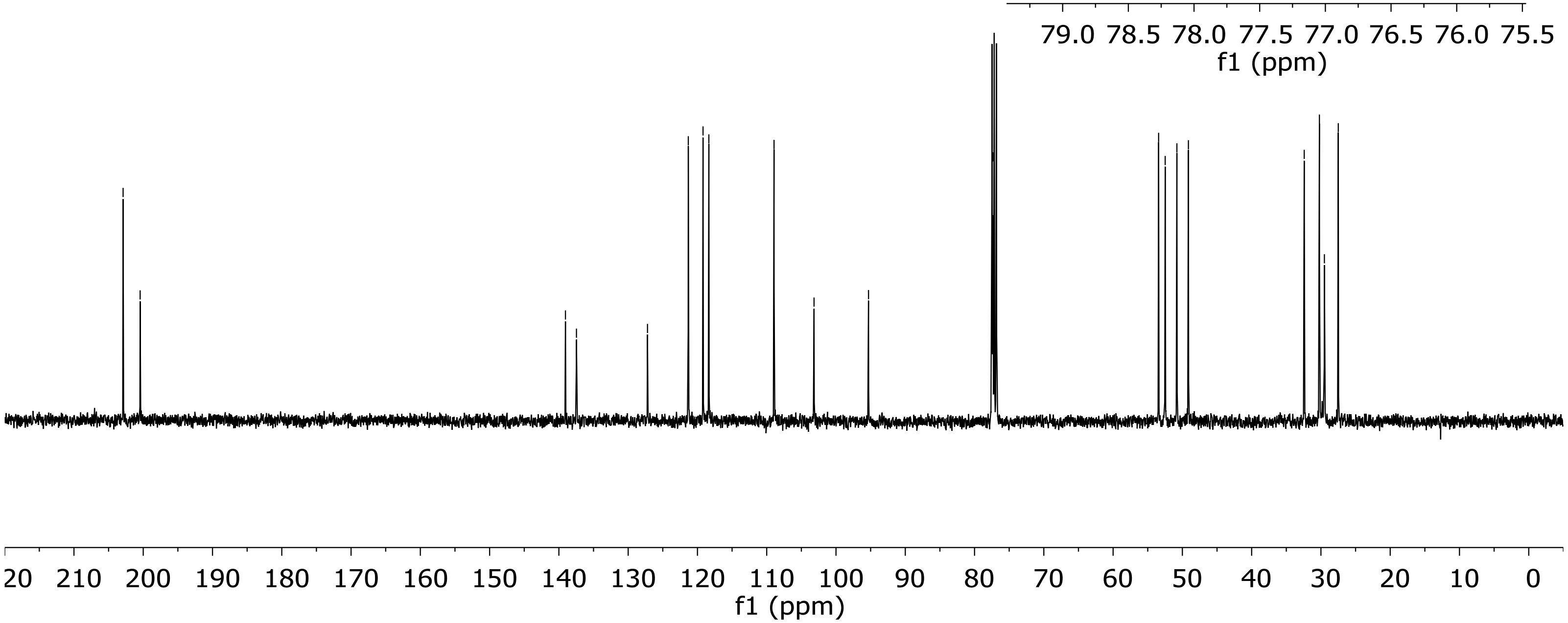
202.91
200.45
139.05
137.46
127.21
121.32
119.19
118.35
108.95
103.17
95.31
77.37
53.43
52.48
50.79
49.13
32.41
30.22
29.50
27.50
77.37
79.0 78.5 78.0 77.5 77.0 76.5 76.0 75.5
f1 (ppm)
20 210 200 190 180 170 160 150 140 130 120 110 100 90 80 70 60 50 40 30 20 10 0
f1 (ppm)

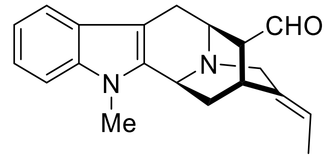

(+)-N_a-methylvellosimine (**5**)

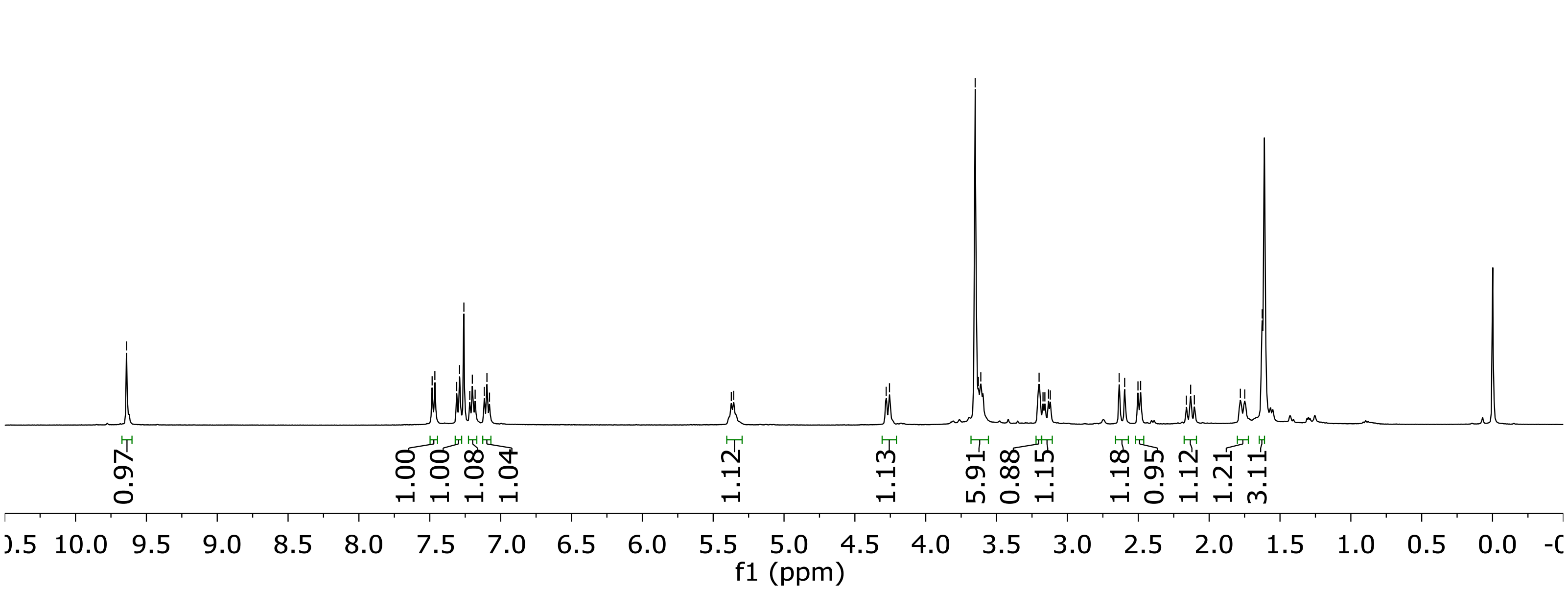

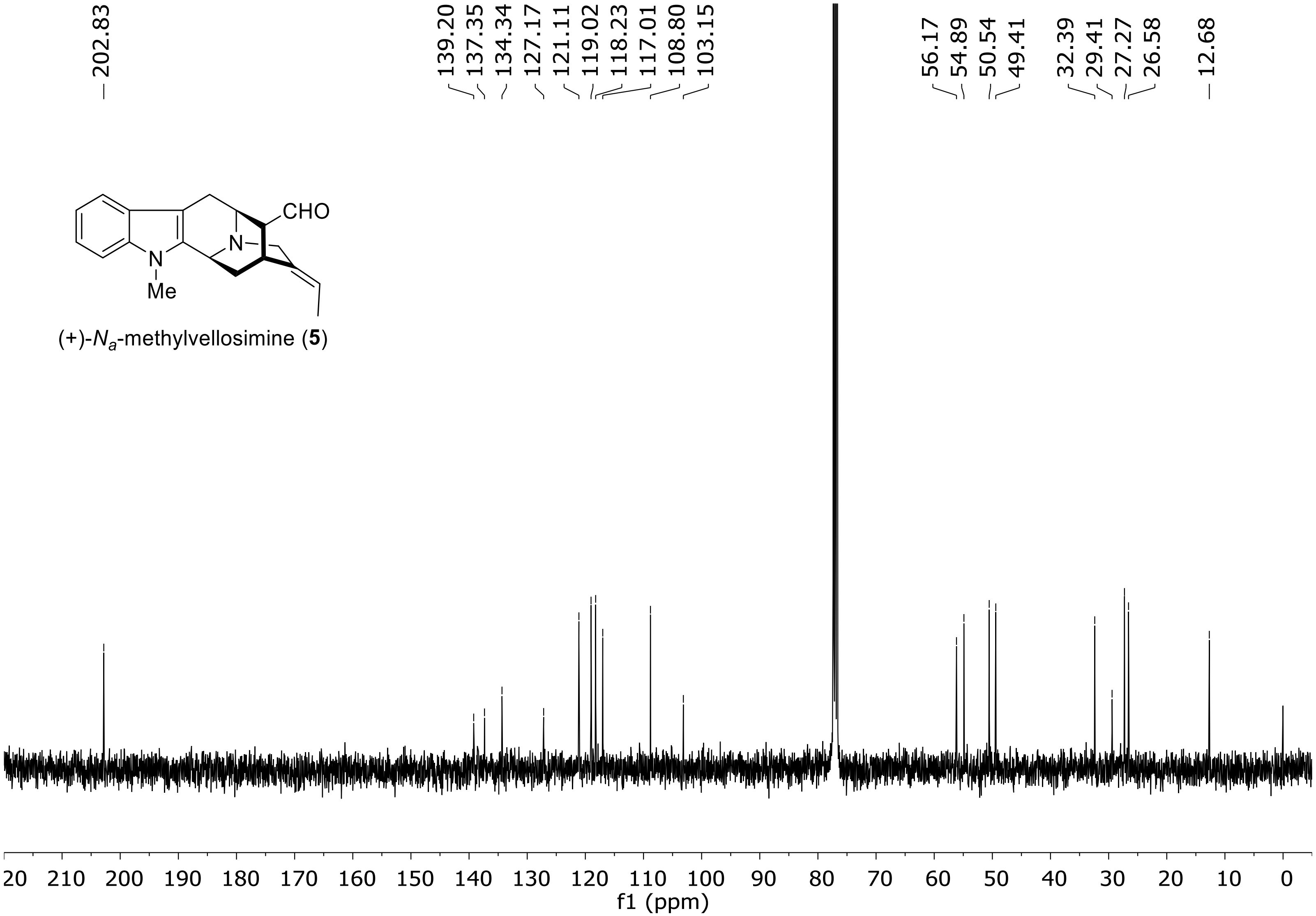
202.83
139.20
137.35
134.34
127.17
121.11
119.02
118.23
117.01
108.80
103.15
56.17
54.89
50.54
49.41
32.39
29.41
27.27
26.58
12.68
CHO
N
N
Me
(+)-N_a-methylvellosimine (**5**)
20 210 200 190 180 170 160 150 140 130 120 110 100 90 80 70 60 50 40 30 20 10 0
f1 (ppm)

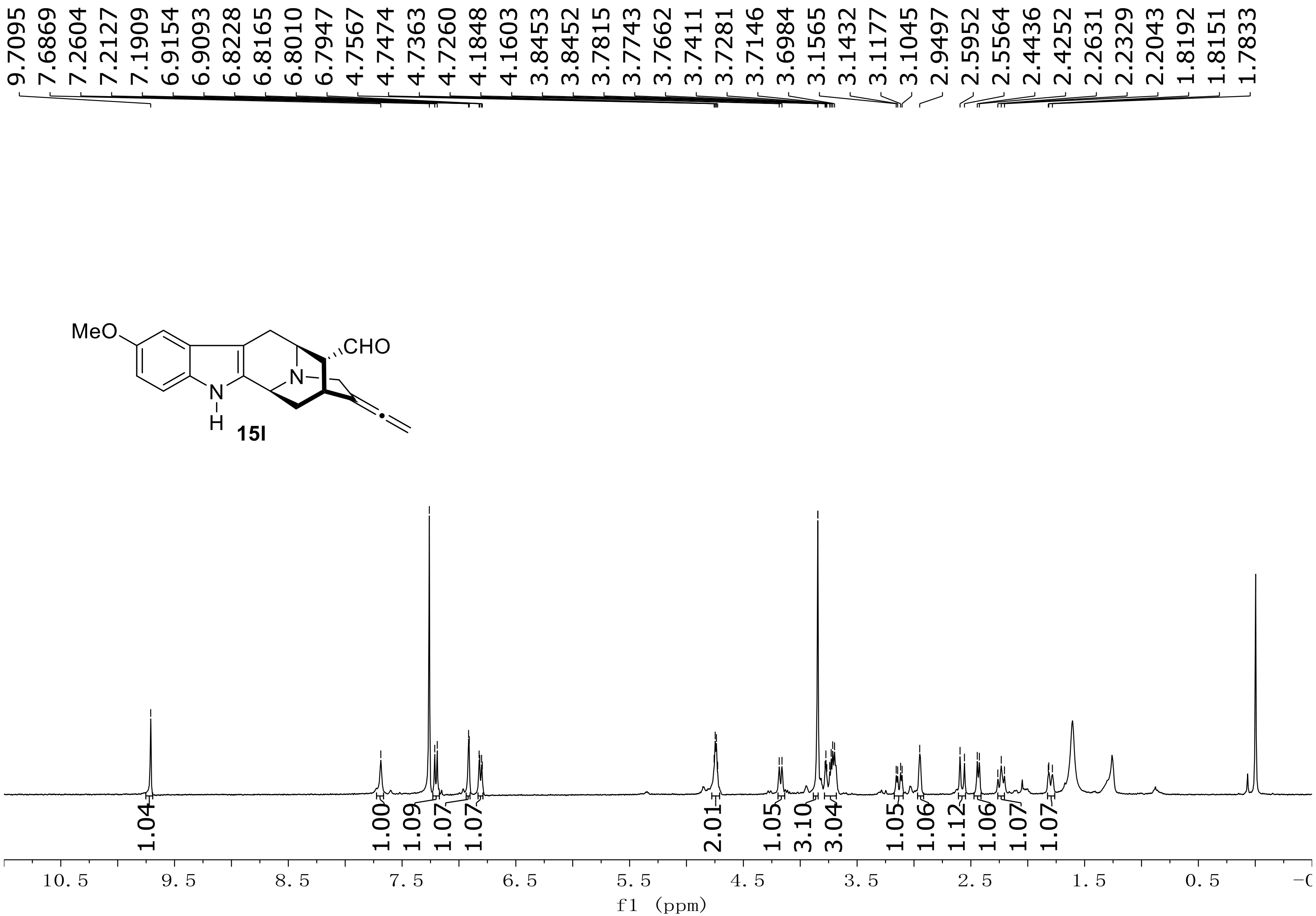

9.7095
7.6869
7.2604
7.2127
7.1909
6.9154
6.9093
6.8228
6.8165
6.8010
6.7947
4.7567
4.7474
4.7363
4.7260
4.1848
4.1603
3.8453
3.8452
3.7815
3.7743
3.7662
3.7411
3.7281
3.7146
3.6984
3.1565
3.1432
3.1177
3.1045
2.9497
2.5952
2.5564
2.4436
2.4252
2.2631
2.2329
2.2043
1.8192
1.8151
1.7833
MeO
CHO
N
N
H
15l
1.04
1.00
1.09
1.07
1.07
2.01
1.05
3.10
3.04
1.05
1.06
1.12
1.06
1.07
1.07
10.5
9.5
8.5
7.5
6.5
5.5
4.5
3.5
2.5
1.5
0.5
f1 (ppm)

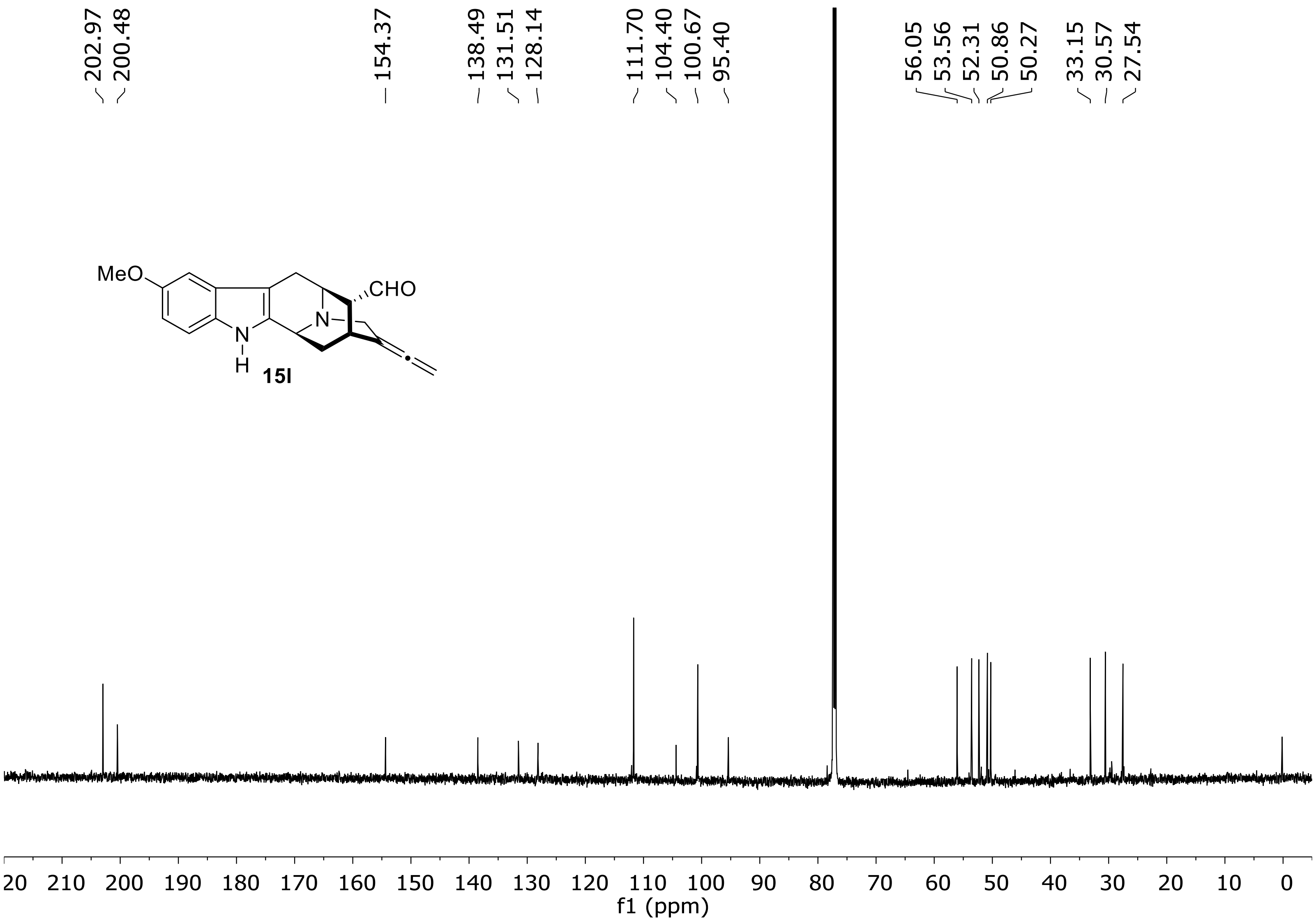

202.97
200.48
154.37
138.49
131.51
128.14
111.70
104.40
100.67
95.40
56.05
53.56
52.31
50.86
50.27
33.15
30.57
27.54
MeO
CHO
N
N
H
15l
20 210 200 190 180 170 160 150 140 130 120 110 100 90 80 70 60 50 40 30 20 10 0
f1 (ppm)

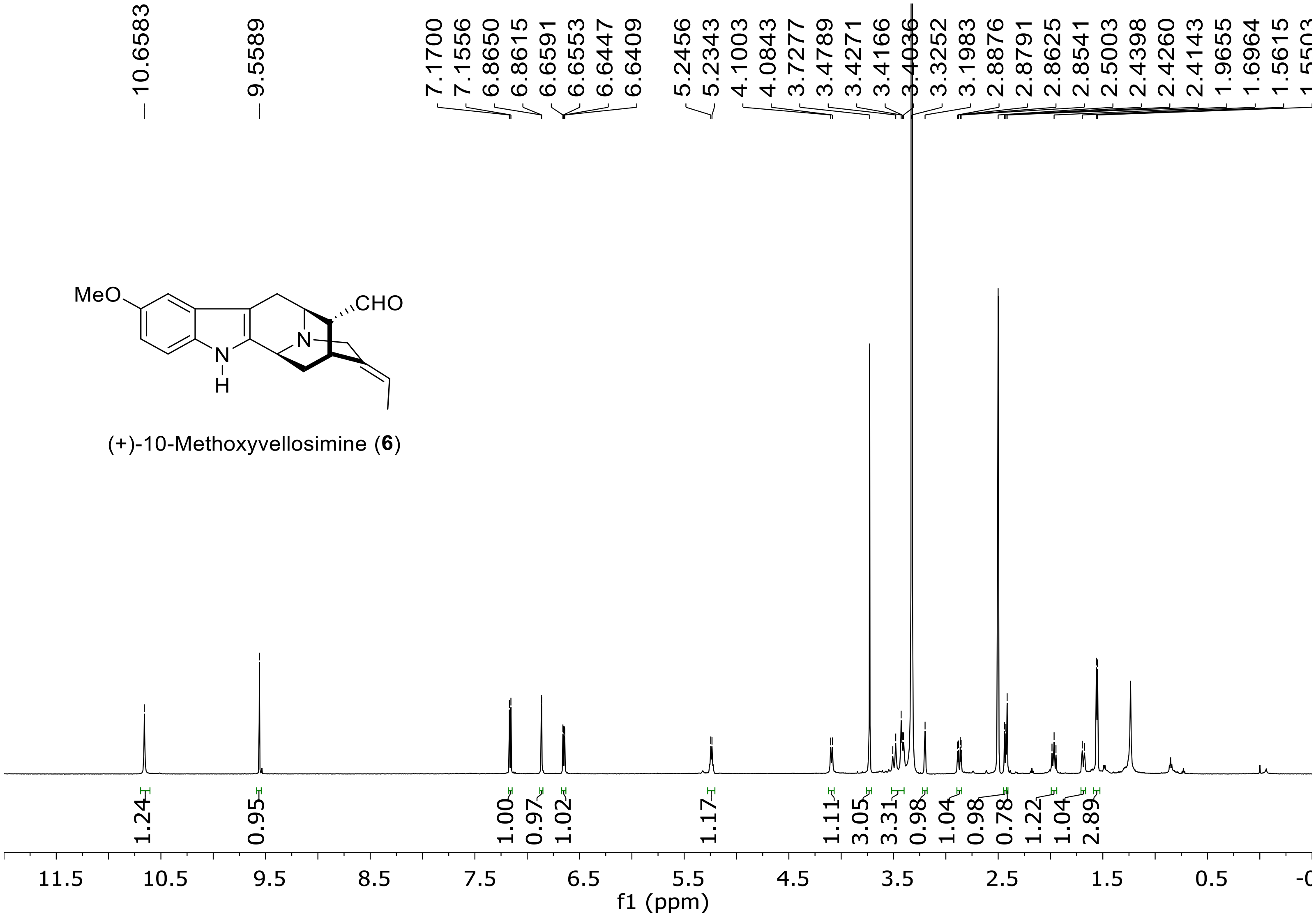

(+)-10-Methoxyvellosimine (**6**)

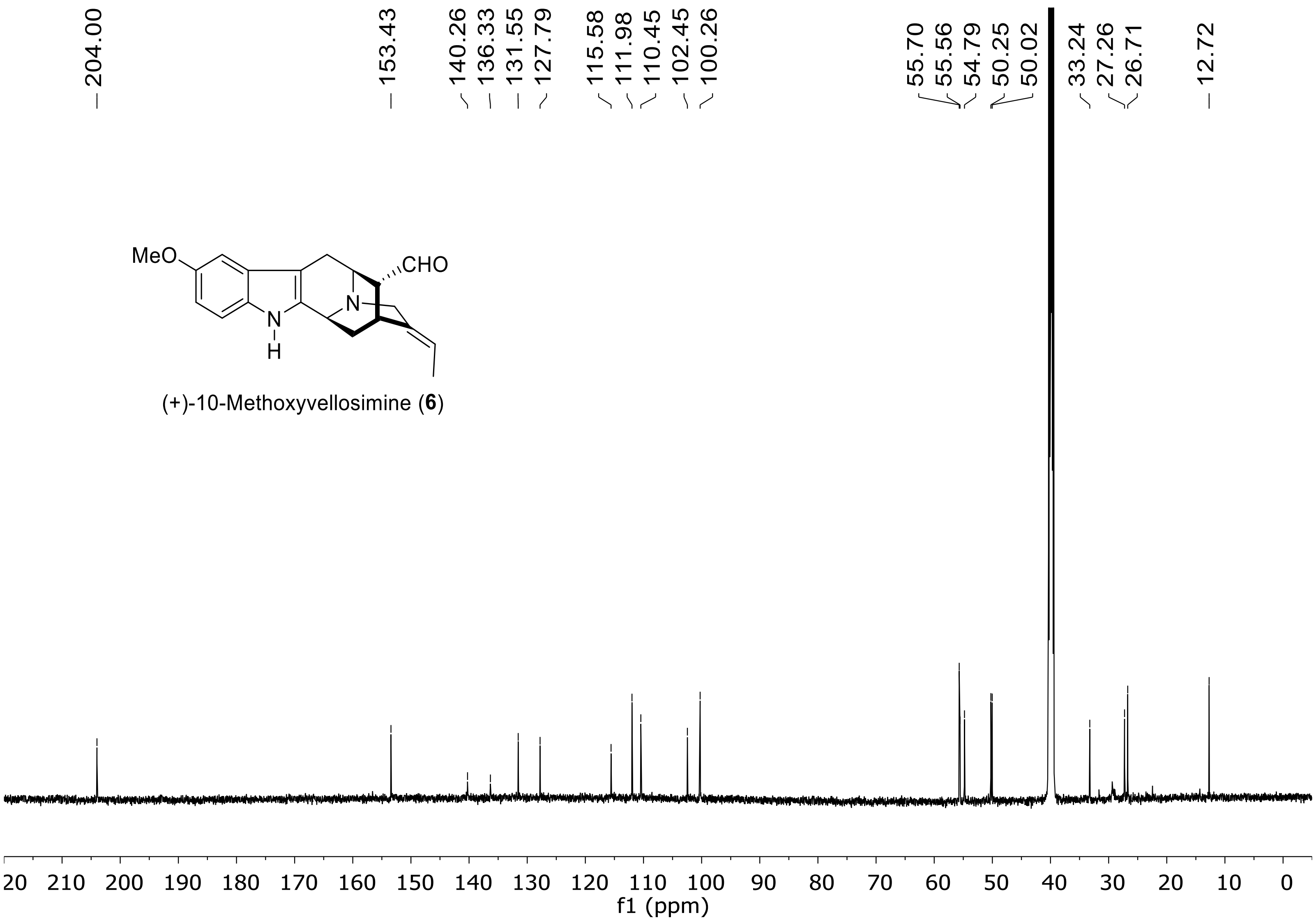
204.00
153.43
140.26
136.33
131.55
127.79
115.58
111.98
110.45
102.45
100.26
55.70
55.56
54.79
50.25
50.02
33.24
27.26
26.71
12.72
MeO
CHO
N
N
H
(+)-10-Methoxyvellosimine (6)
20 210 200 190 180 170 160 150 140 130 120 110 100 90 80 70 60 50 40 30 20 10 0
f1 (ppm)

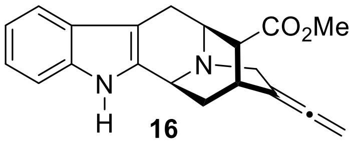

16

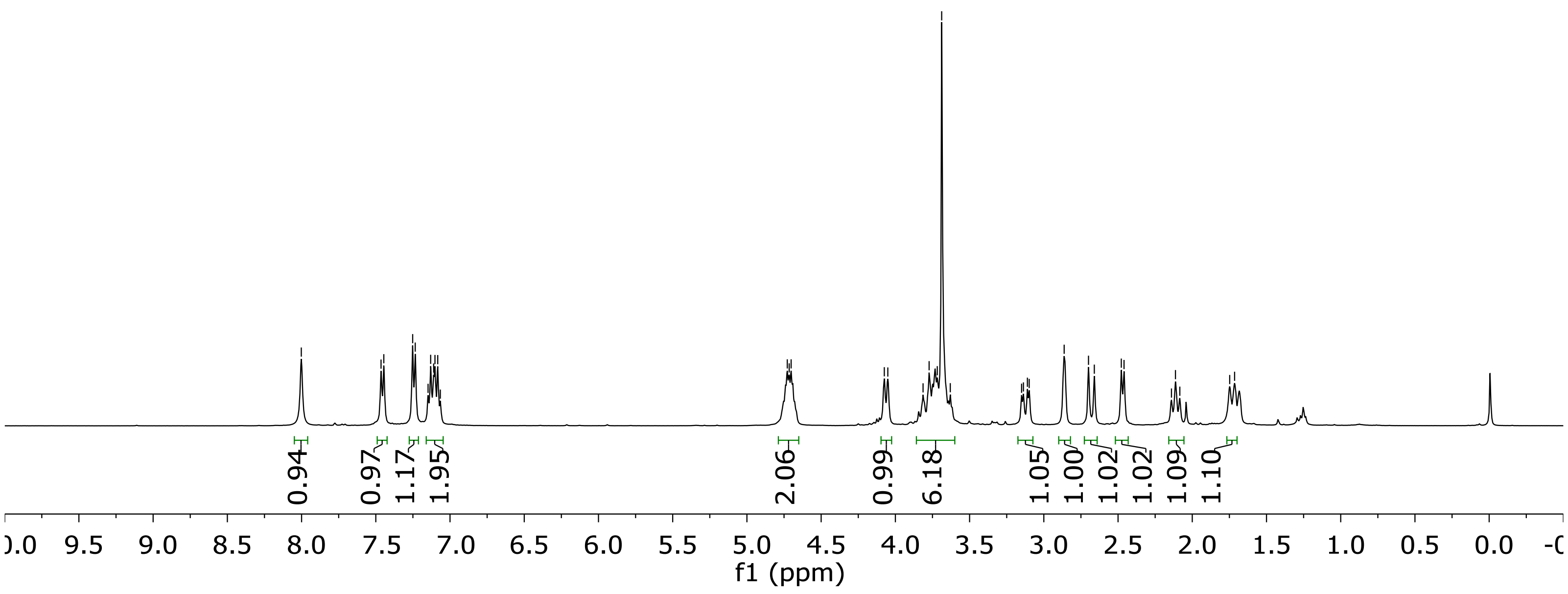
8.0019
7.4647
7.4461
7.2516
7.2344
7.1479
7.1304
7.1102
7.1014
7.0828
7.0654
4.7282
4.7152
4.7022
4.0746
4.0509
3.8135
3.7727
3.7177
3.6881
3.6303
3.1498
3.1375
3.1107
3.0985
2.8629
2.6990
2.6599
2.4780
2.4597
2.1401
2.1133
2.0839
1.7479
1.7149
0.94
0.97
1.17
1.95
2.06
0.99
6.18
1.05
1.00
1.02
1.02
1.09
1.10
f1 (ppm)

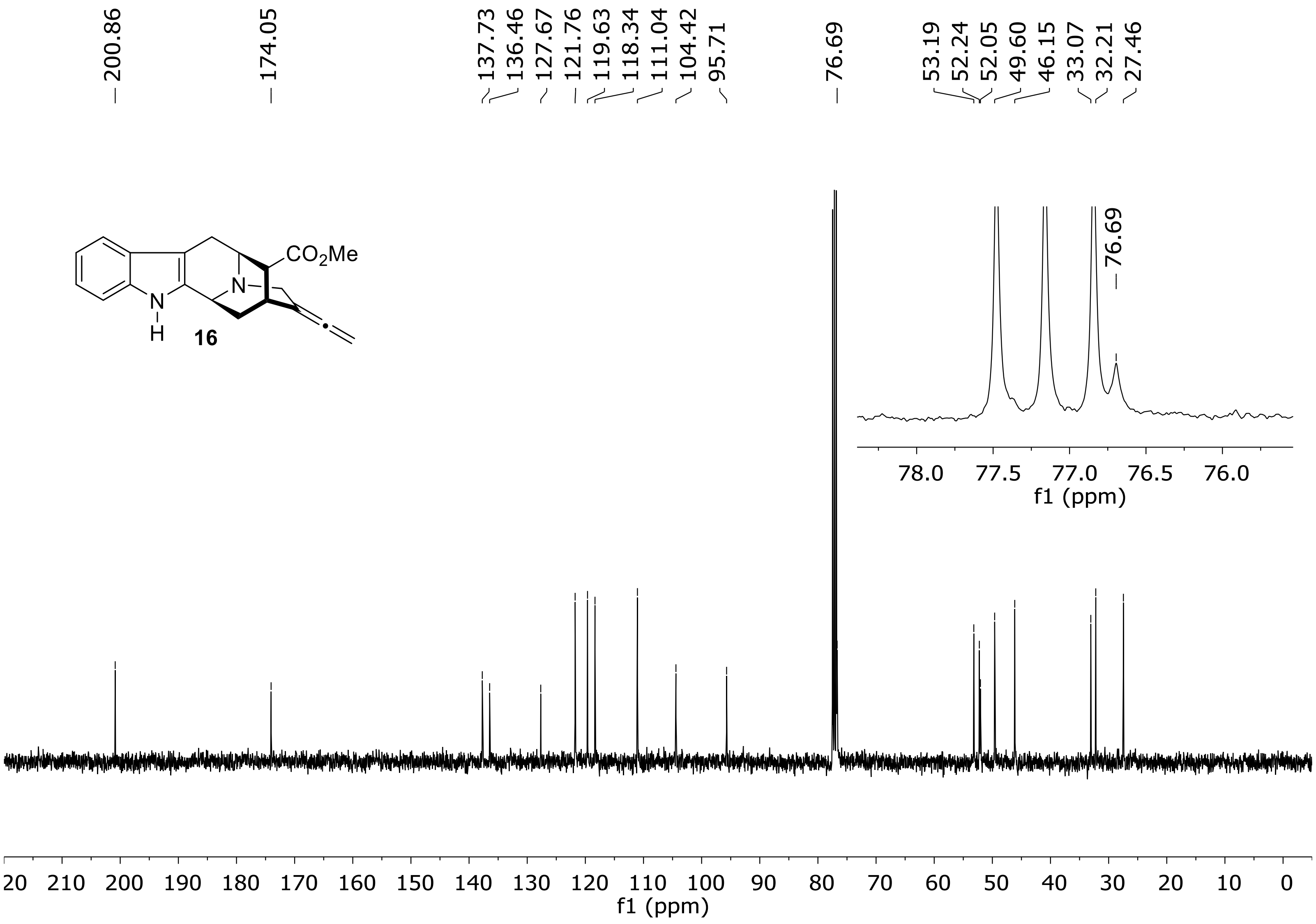
200.86
174.05
137.73
136.46
127.67
121.76
119.63
118.34
111.04
104.42
95.71
76.69
53.19
52.24
52.05
49.60
46.15
33.07
32.21
27.46
CO_2Me
N
N
H
16
76.69
78.0 77.5 77.0 76.5 76.0
f1 (ppm)
20 210 200 190 180 170 160 150 140 130 120 110 100 90 80 70 60 50 40 30 20 10 0
f1 (ppm)

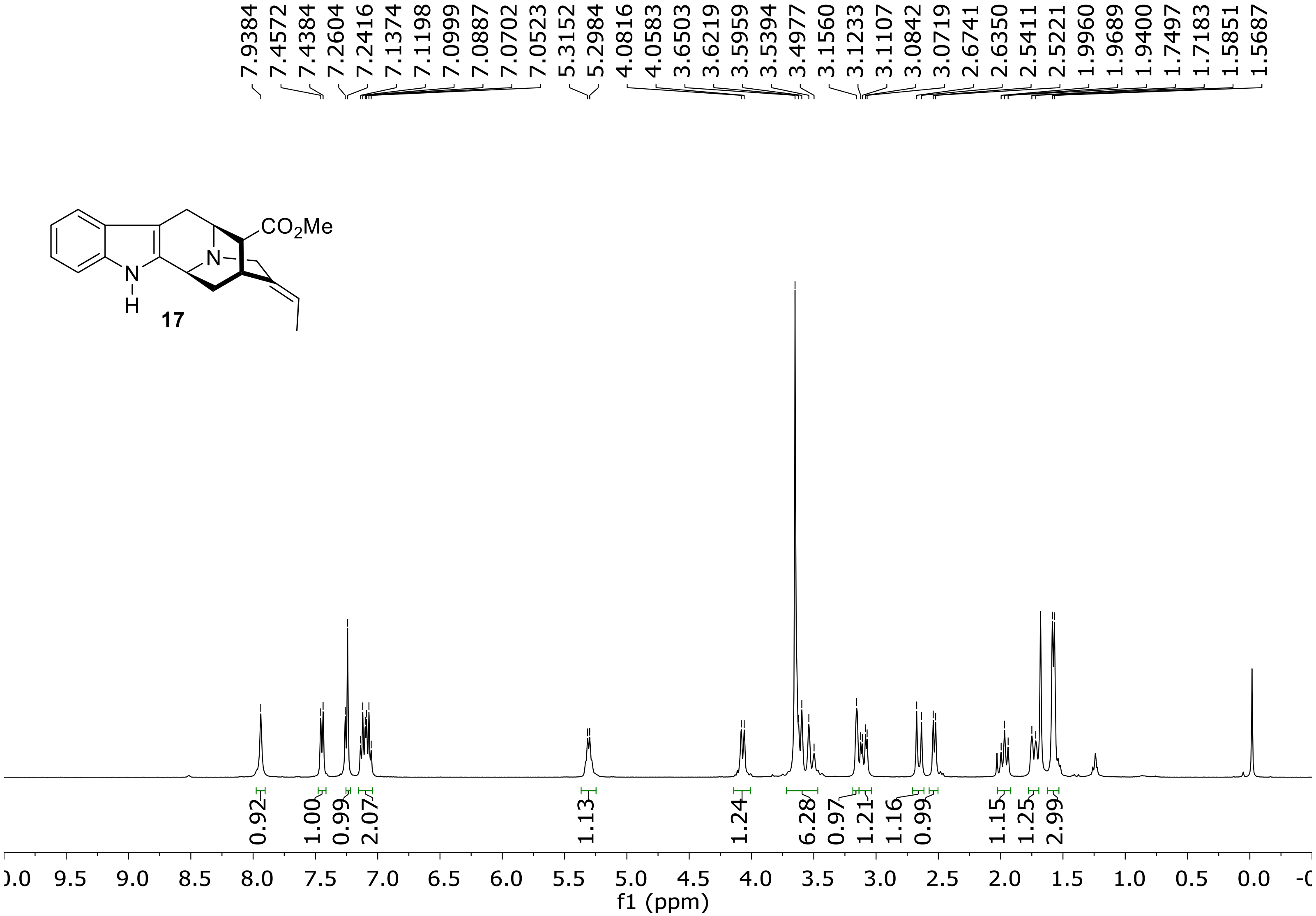
7.9384
7.4572
7.4384
7.2604
7.2416
7.1374
7.1198
7.0999
7.0887
7.0702
7.0523
5.3152
5.2984
4.0816
4.0583
3.6503
3.6219
3.5959
3.5394
3.4977
3.1560
3.1233
3.1107
3.0842
3.0719
2.6741
2.6350
2.5411
2.5221
1.9960
1.9689
1.9400
1.7497
1.7183
1.5851
1.5687
CO2Me
N
N
H
17
0.92
1.00
0.99
2.07
1.13
1.24
6.28
0.97
1.21
1.16
0.99
1.15
1.25
2.99
9.5 9.0 8.5 8.0 7.5 7.0 6.5 6.0 5.5 5.0 4.5 4.0 3.5 3.0 2.5 2.0 1.5 1.0 0.5 0.0
f1 (ppm)

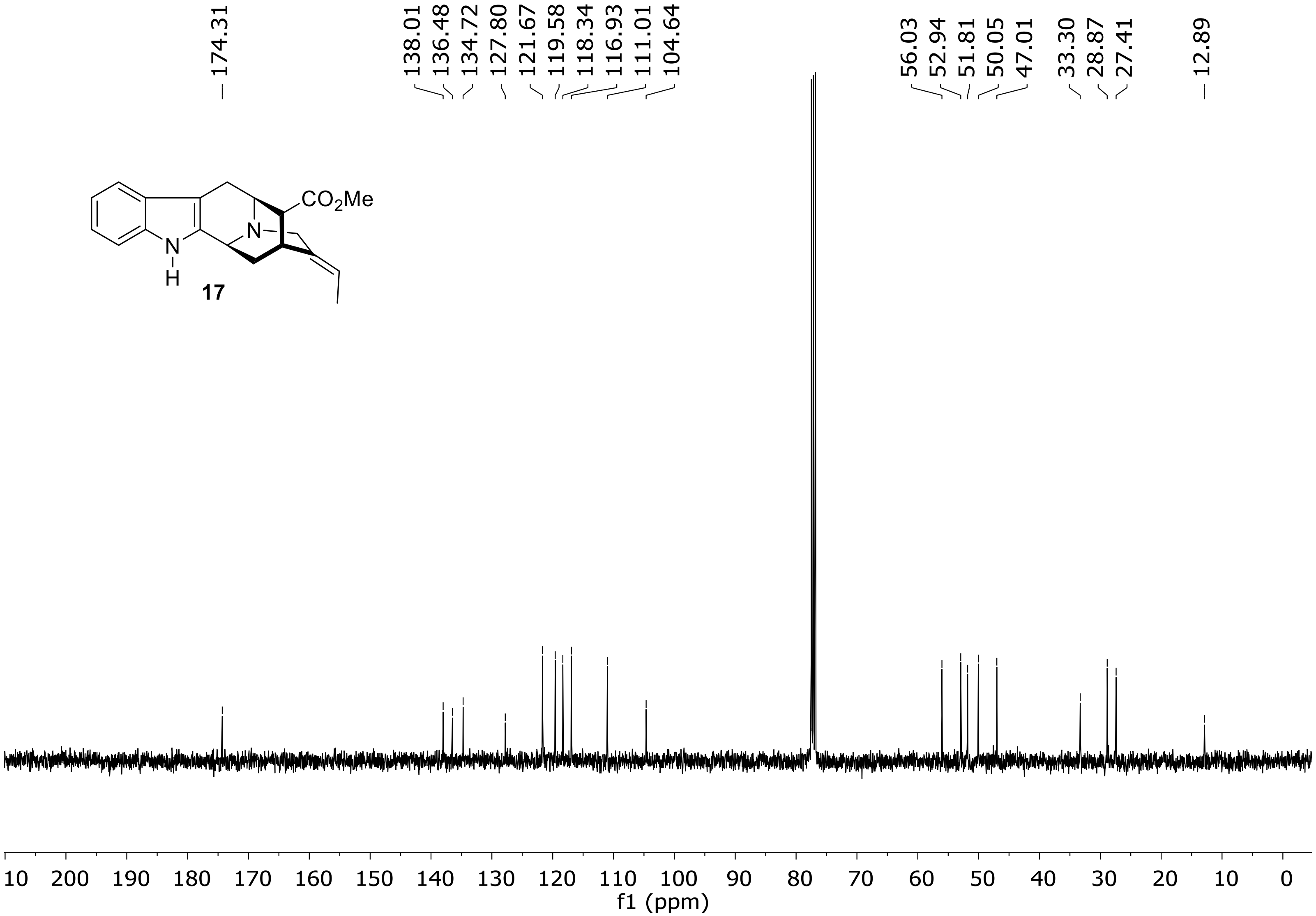
174.31
138.01
136.48
134.72
127.80
121.67
119.58
118.34
116.93
111.01
104.64
56.03
52.94
51.81
50.05
47.01
33.30
28.87
27.41
12.89
CO_2Me
N
N
H
17
10 200 190 180 170 160 150 140 130 120 110 100 90 80 70 60 50 40 30 20 10 0
f1 (ppm)

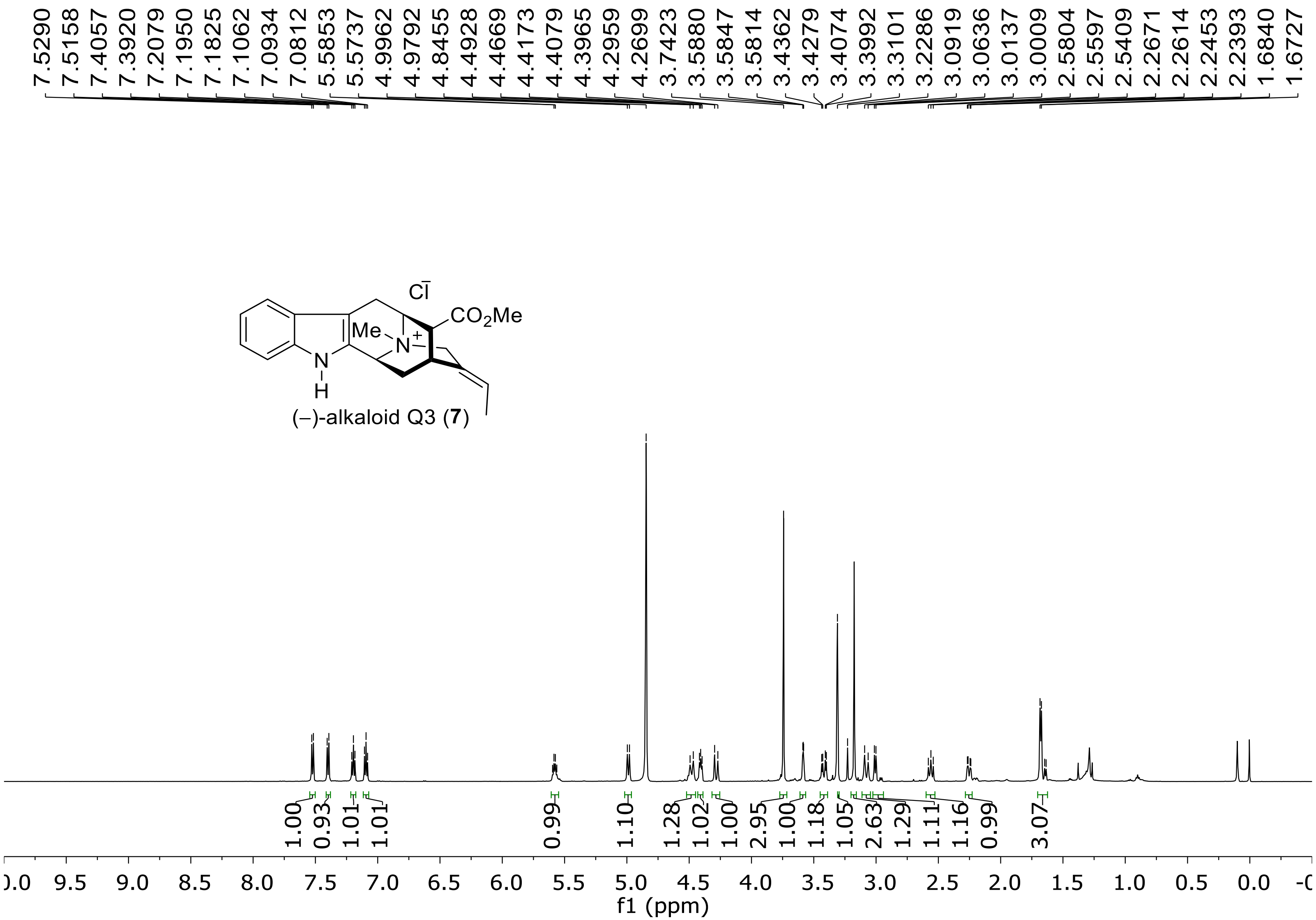

7.5290
7.5158
7.4057
7.3920
7.2079
7.1950
7.1825
7.1062
7.0934
7.0812
5.5853
5.5737
4.9962
4.9792
4.8455
4.4928
4.4669
4.4173
4.4079
4.3965
4.2959
4.2699
3.7423
3.5880
3.5847
3.5814
3.4362
3.4279
3.4074
3.3992
3.3101
3.2286
3.0919
3.0636
3.0137
3.0009
2.5804
2.5597
2.5409
2.2671
2.2614
2.2453
2.2393
1.6840
1.6727
Cl
CO2Me
Me
N
N
H
(–)-alkaloid Q3 (7)
1.00
0.93
1.01
1.01
0.99
1.10
1.28
1.02
1.00
2.95
1.00
1.18
1.05
2.63
1.29
1.11
1.16
0.99
3.07
f1 (ppm)

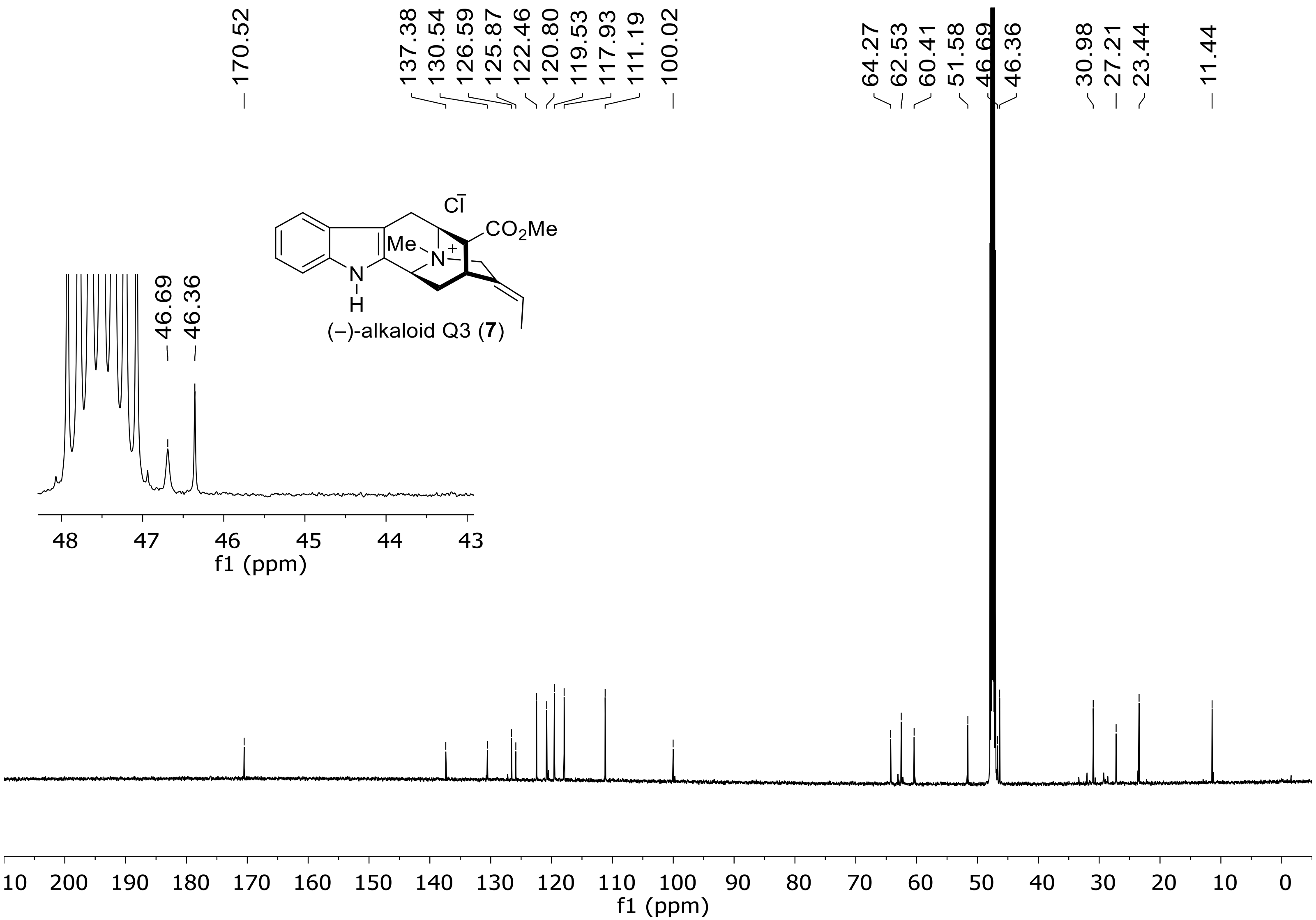
170.52
137.38
130.54
126.59
125.87
122.46
120.80
119.53
117.93
111.19
100.02
64.27
62.53
60.41
51.58
46.69
46.36
30.98
27.21
23.44
11.44
Cl⁻
CO2Me
Me
N+
N
H
(–)-alkaloid Q3 (7)
46.69
46.36
48 47 46 45 44 43
f1 (ppm)
10 200 190 180 170 160 150 140 130 120 110 100 90 80 70 60 50 40 30 20 10 0
f1 (ppm)

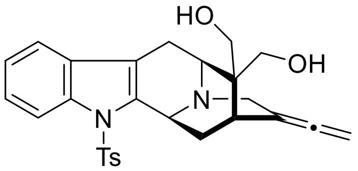
HO
OH
N
N
Ts

15as

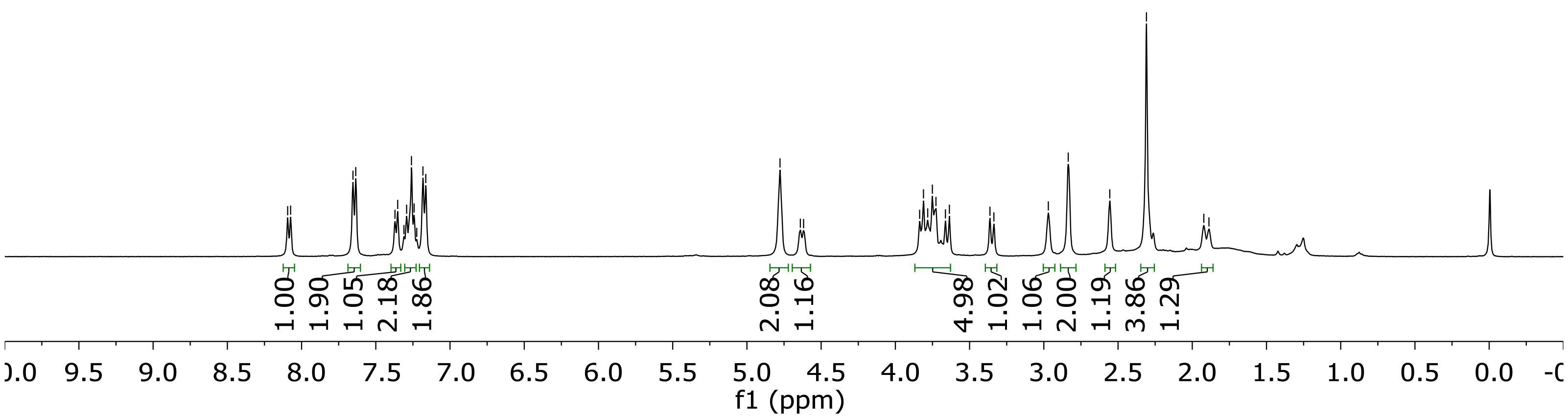
8.0936
8.0737
7.6537
7.6349
7.3707
7.3525
7.3104
7.2923
7.2597
7.2420
7.2242
7.1825
7.1633
4.7772
4.6401
4.6183
3.8364
3.8107
3.7821
3.7508
3.7266
3.6629
3.6362
3.3628
3.3363
2.9696
2.8357
2.5557
2.3086
1.9222
1.8877
1.00
1.90
1.05
2.18
1.86
2.08
1.16
4.98
1.02
1.06
2.00
1.19
3.86
1.29
f1 (ppm)

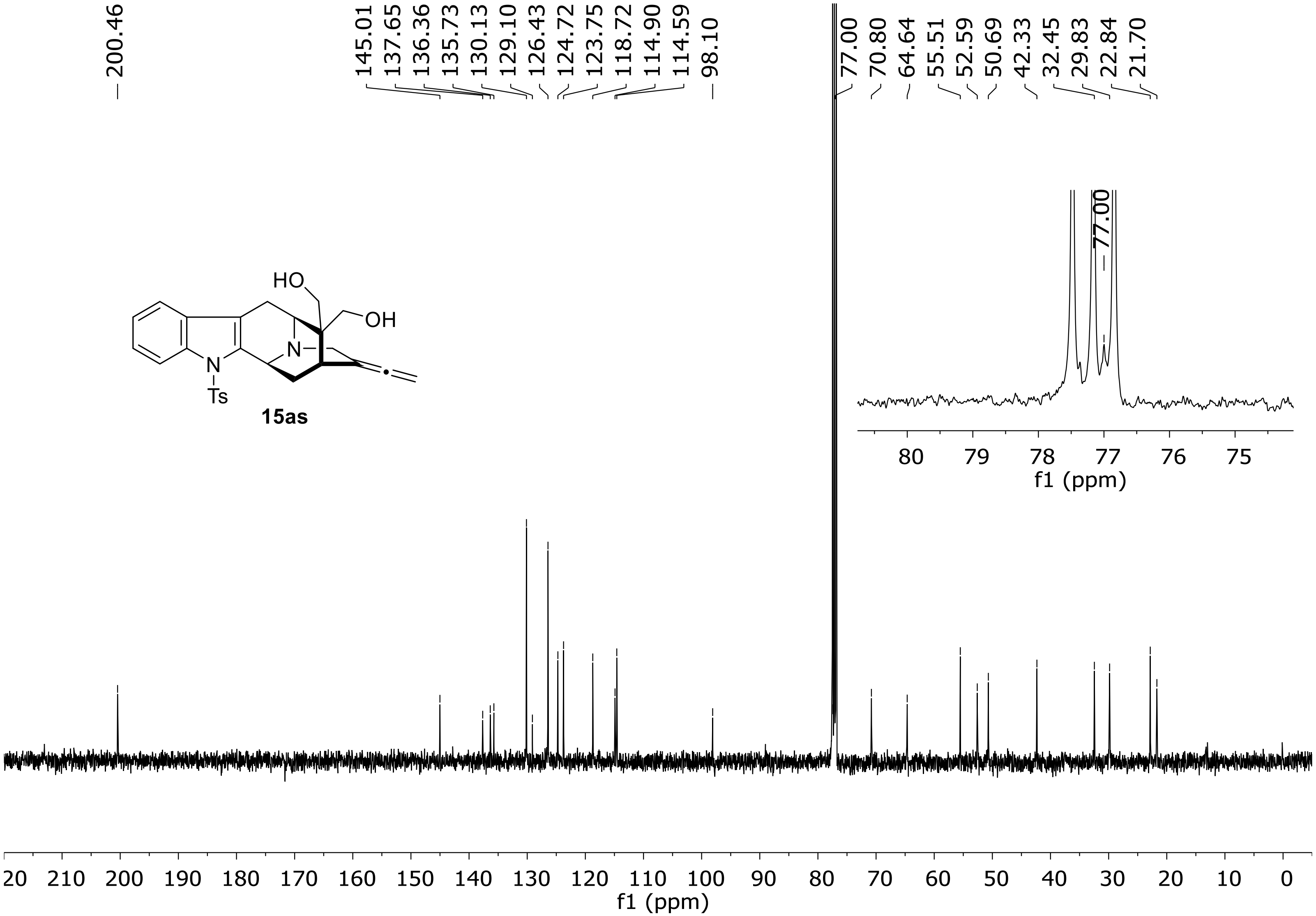
200.46
145.01
137.65
136.36
135.73
130.13
129.10
126.43
124.72
123.75
118.72
114.90
114.59
98.10
77.00
70.80
64.64
55.51
52.59
50.69
42.33
32.45
29.83
22.84
21.70
HO
OH
N
N
Ts
15as
77.00
80 79 78 77 76 75
f1 (ppm)
20 210 200 190 180 170 160 150 140 130 120 110 100 90 80 70 60 50 40 30 20 10 0
f1 (ppm)

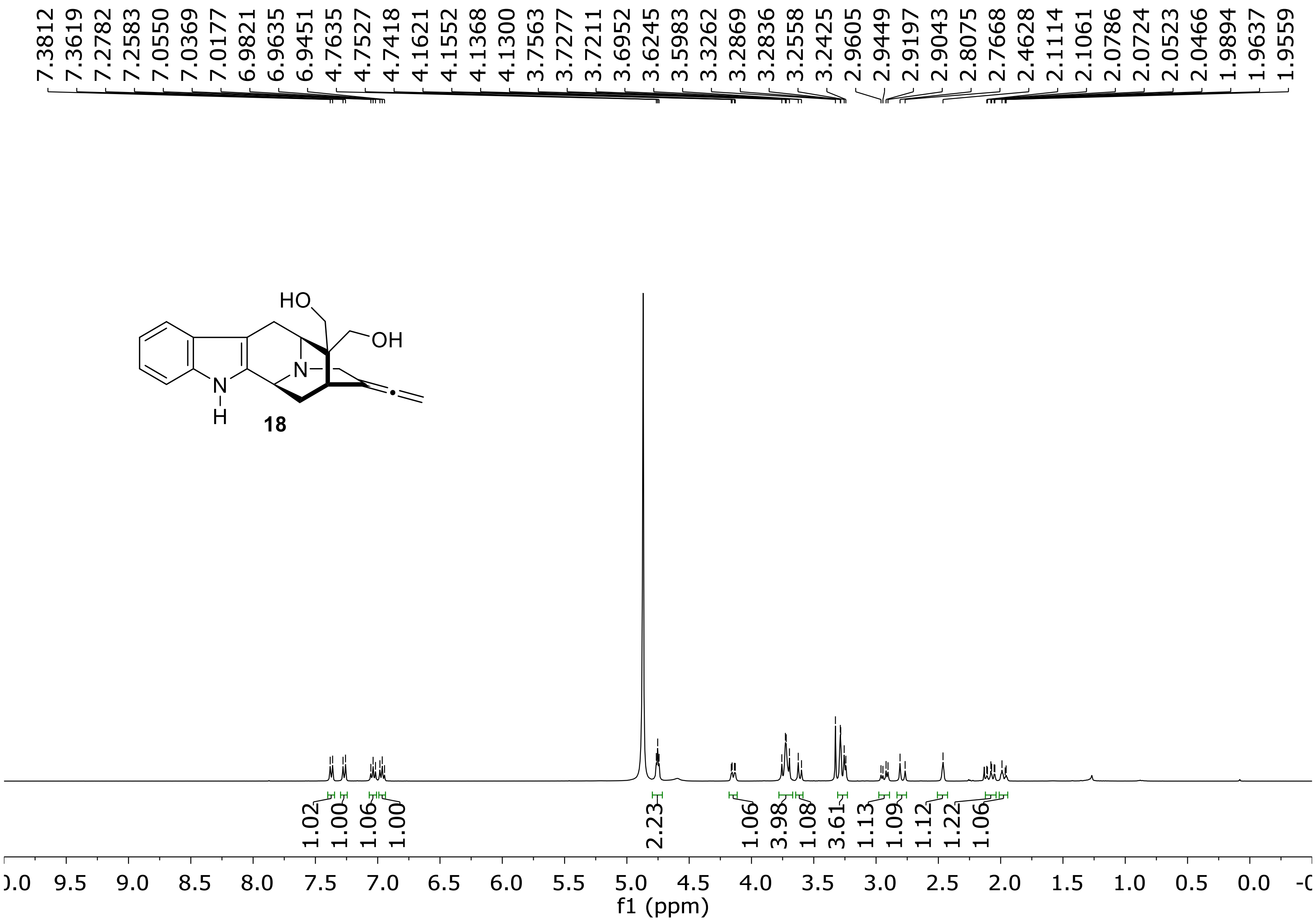
7.3812
7.3619
7.2782
7.2583
7.0550
7.0369
7.0177
6.9821
6.9635
6.9451
4.7635
4.7527
4.7418
4.1621
4.1552
4.1368
4.1300
3.7563
3.7277
3.7211
3.6952
3.6245
3.5983
3.3262
3.2869
3.2836
3.2558
3.2425
2.9605
2.9449
2.9197
2.9043
2.8075
2.7668
2.4628
2.1114
2.1061
2.0786
2.0724
2.0523
2.0466
1.9894
1.9637
1.9559
HO
OH
N
N
H
18
1.02
1.00
1.06
1.00
2.23
1.06
3.98
1.08
3.61
1.13
1.09
1.12
1.22
1.06
f1 (ppm)

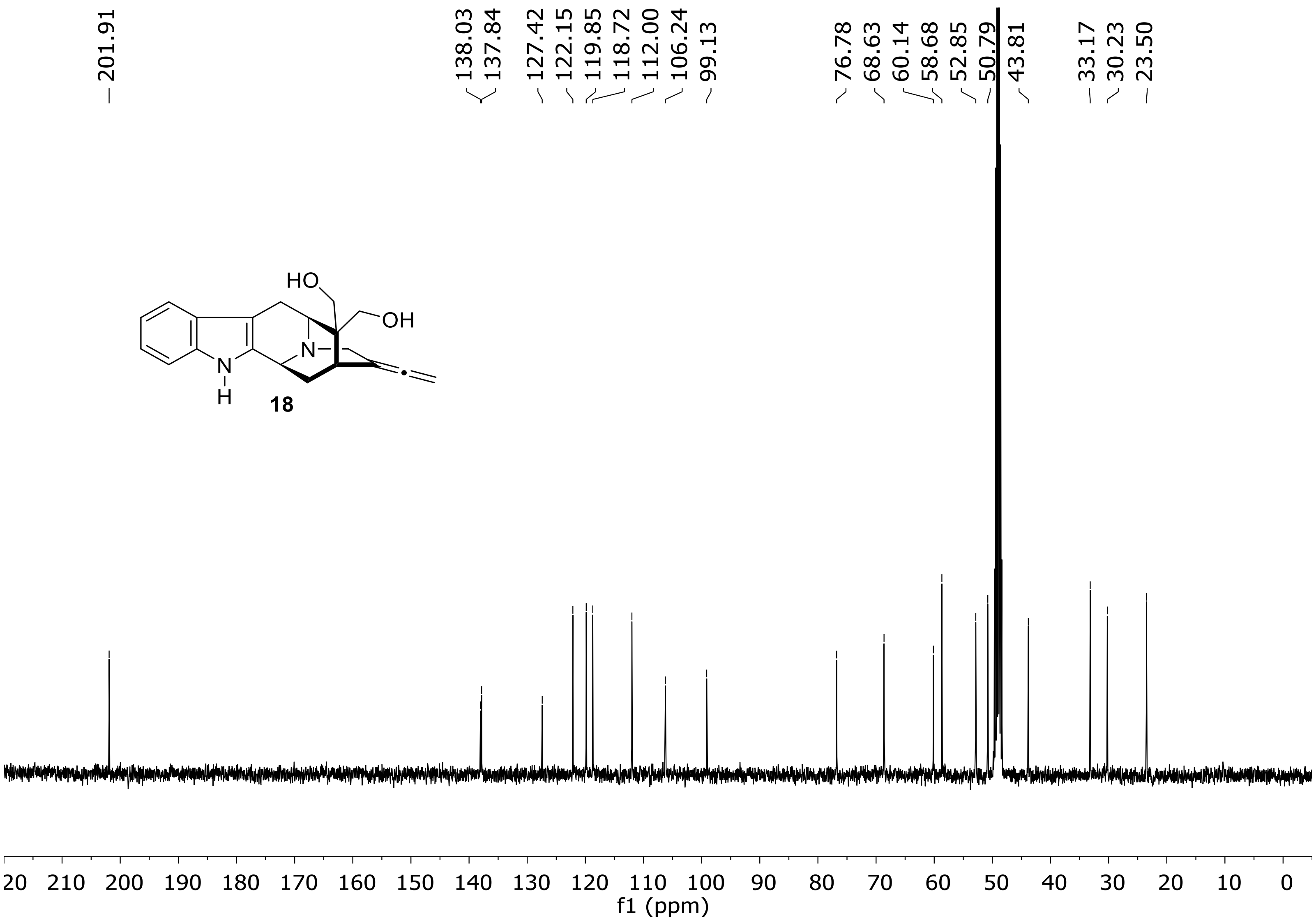
201.91
138.03
137.84
127.42
122.15
119.85
118.72
112.00
106.24
99.13
76.78
68.63
60.14
58.68
52.85
50.79
43.81
33.17
30.23
23.50
HO
OH
N
N
H
18
f1 (ppm)

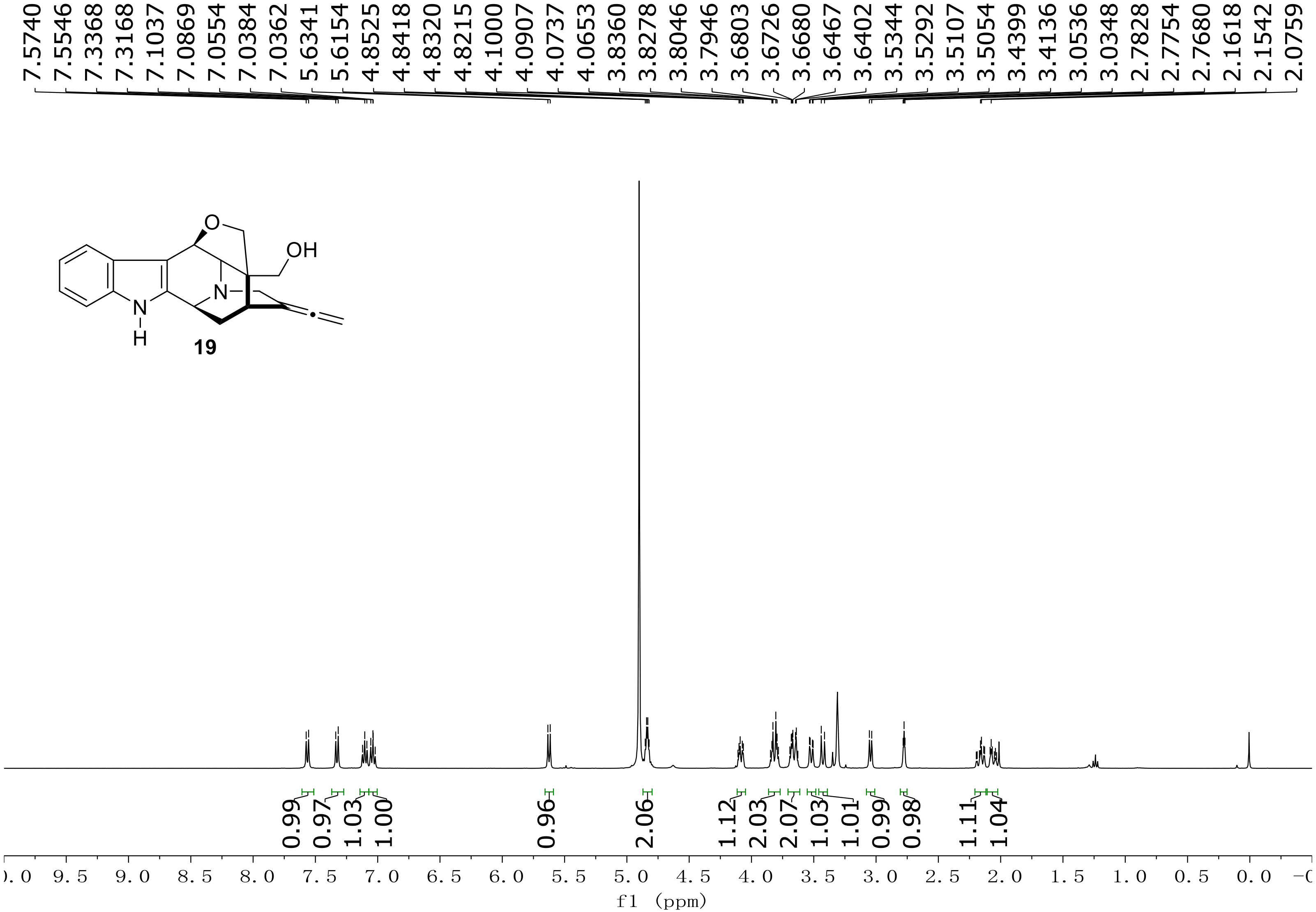
7.5740
7.5546
7.3368
7.3168
7.1037
7.0869
7.0554
7.0384
7.0362
5.6341
5.6154
4.8525
4.8418
4.8320
4.8215
4.1000
4.0907
4.0737
4.0653
3.8360
3.8278
3.8046
3.7946
3.6803
3.6726
3.6680
3.6467
3.6402
3.5344
3.5292
3.5107
3.5054
3.4399
3.4136
3.0536
3.0348
2.7828
2.7754
2.7680
2.1618
2.1542
2.0759
OH
O
N
N
H
19
0.99
0.97
1.03
1.00
0.96
2.06
1.12
2.03
2.07
1.03
1.01
0.99
0.98
1.11
1.04
f1 (ppm)

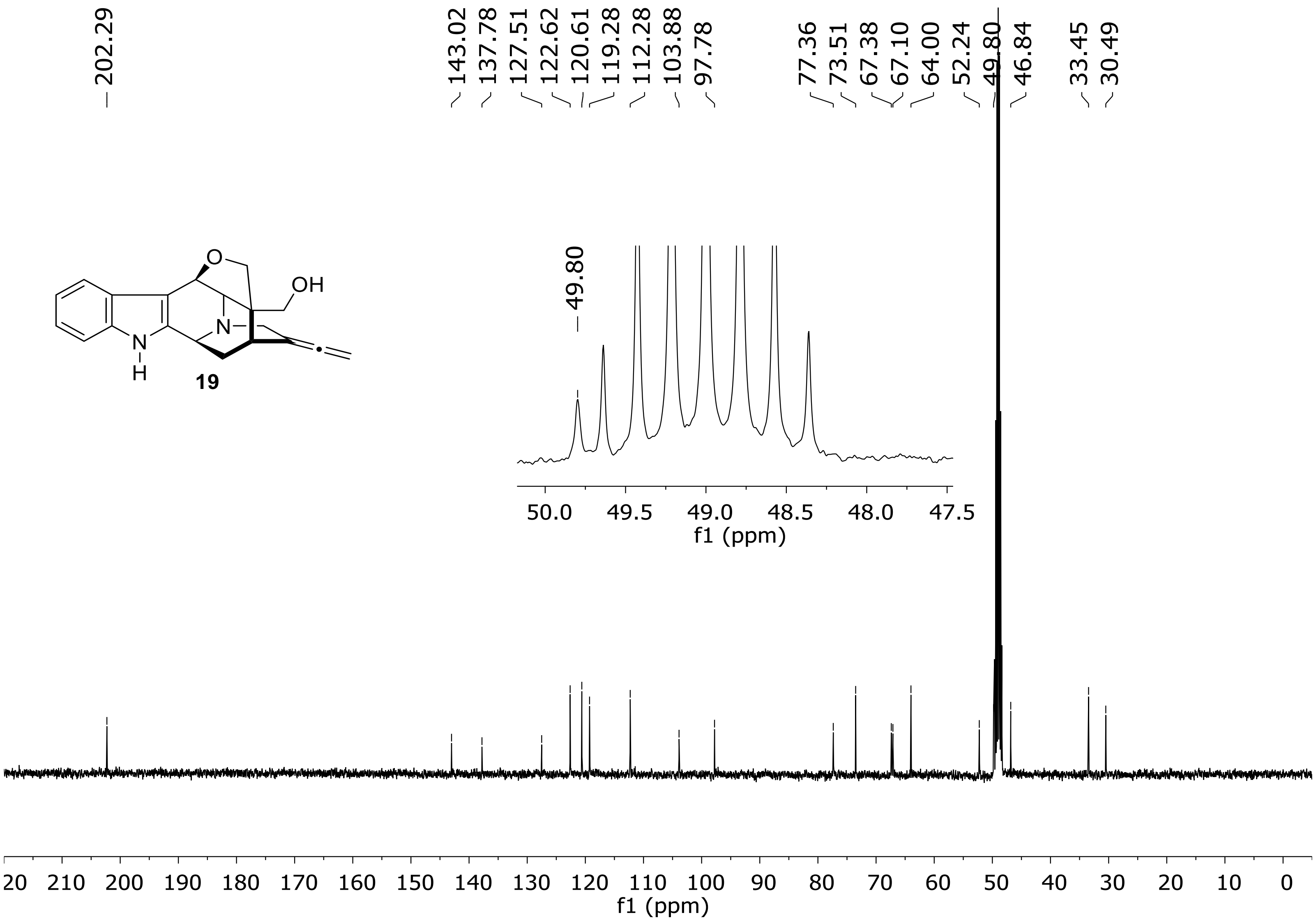

202.29
143.02
137.78
127.51
122.62
120.61
119.28
112.28
103.88
97.78
77.36
73.51
67.38
67.10
64.00
52.24
49.80
46.84
33.45
30.49
O
OH
N
N
H
19
49.80
50.0
49.5
49.0
48.5
48.0
47.5
f1 (ppm)
20
210
200
190
180
170
160
150
140
130
120
110
100
90
80
70
60
50
40
30
20
10
0
f1 (ppm)

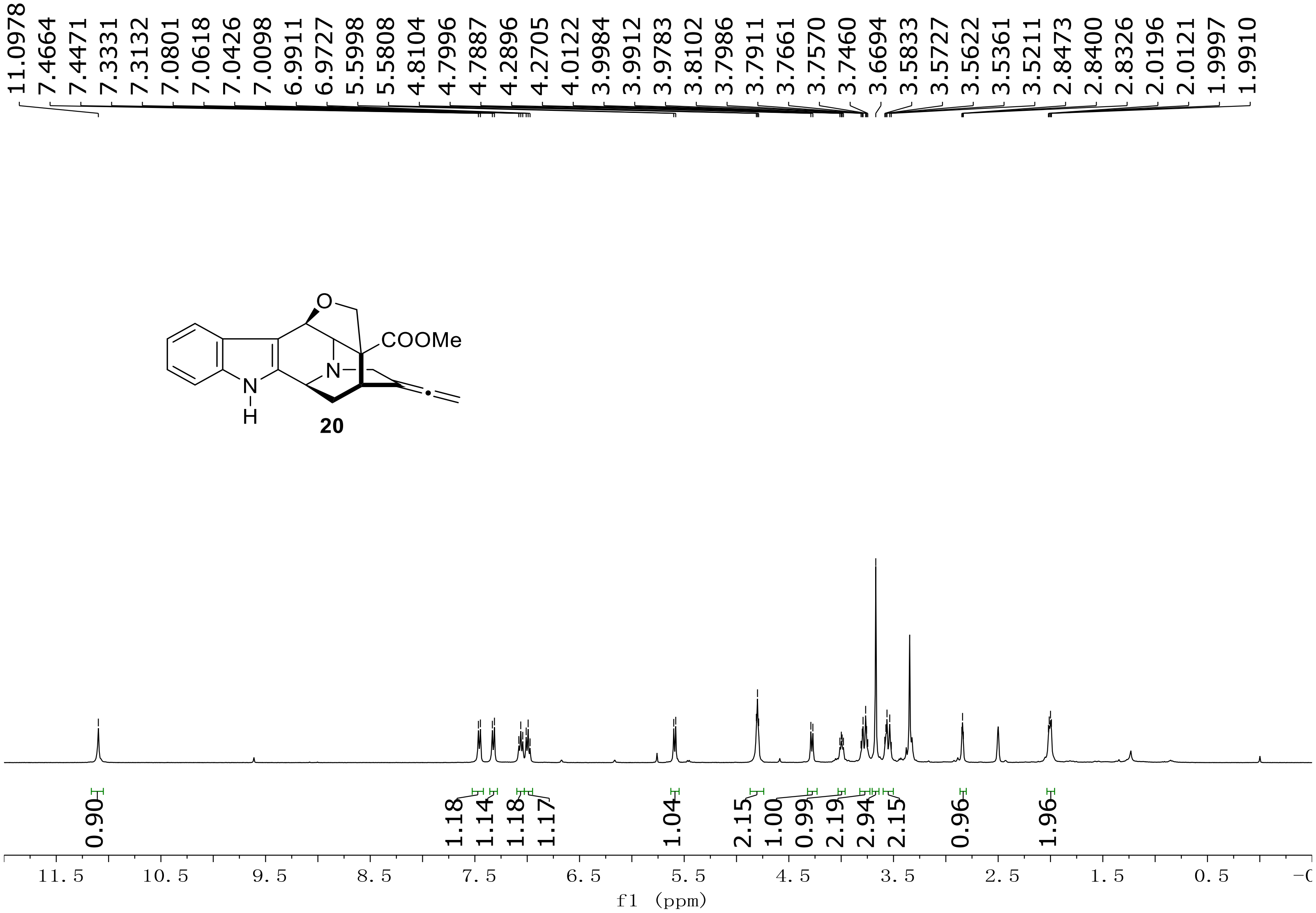
11.0978
7.4664
7.4471
7.3331
7.3132
7.0801
7.0618
7.0426
7.0098
6.9911
6.9727
5.5998
5.5808
4.8104
4.7996
4.7887
4.2896
4.2705
4.0122
3.9984
3.9912
3.9783
3.8102
3.7986
3.7911
3.7661
3.7570
3.7460
3.6694
3.5833
3.5727
3.5622
3.5361
3.5211
2.8473
2.8400
2.8326
2.0196
2.0121
1.9997
1.9910
O
COOMe
N
N
H
20
0.90
1.18
1.14
1.18
1.17
1.04
2.15
1.00
0.99
2.19
2.94
2.15
0.96
1.96
11.5
10.5
9.5
8.5
7.5
6.5
5.5
4.5
3.5
2.5
1.5
0.5
f1 (ppm)

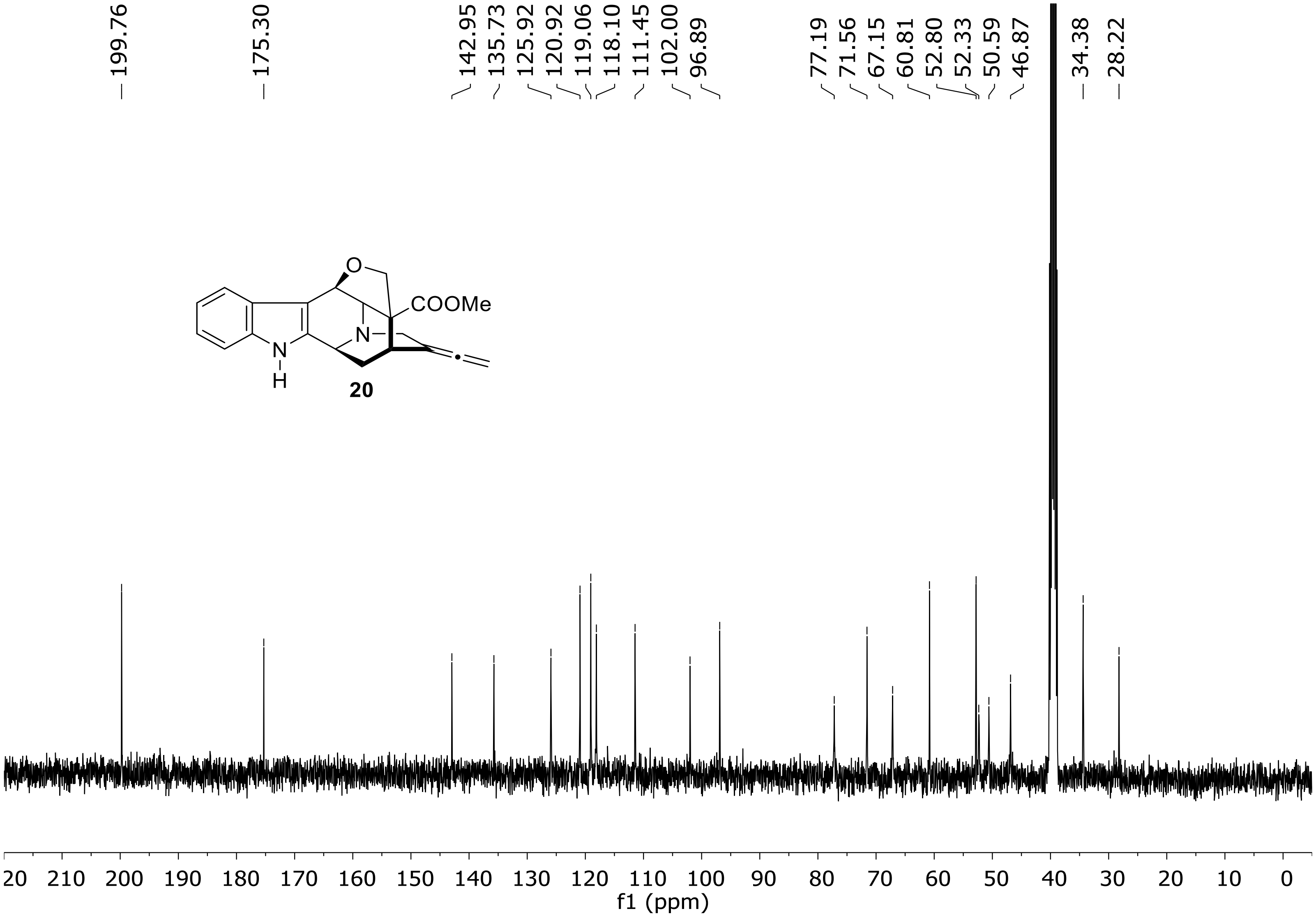

199.76
175.30
142.95
135.73
125.92
120.92
119.06
118.10
111.45
102.00
96.89
77.19
71.56
67.15
60.81
52.80
52.33
50.59
46.87
34.38
28.22
O
COOMe
N
N
H
20
20 210 200 190 180 170 160 150 140 130 120 110 100 90 80 70 60 50 40 30 20 10 0
f1 (ppm)

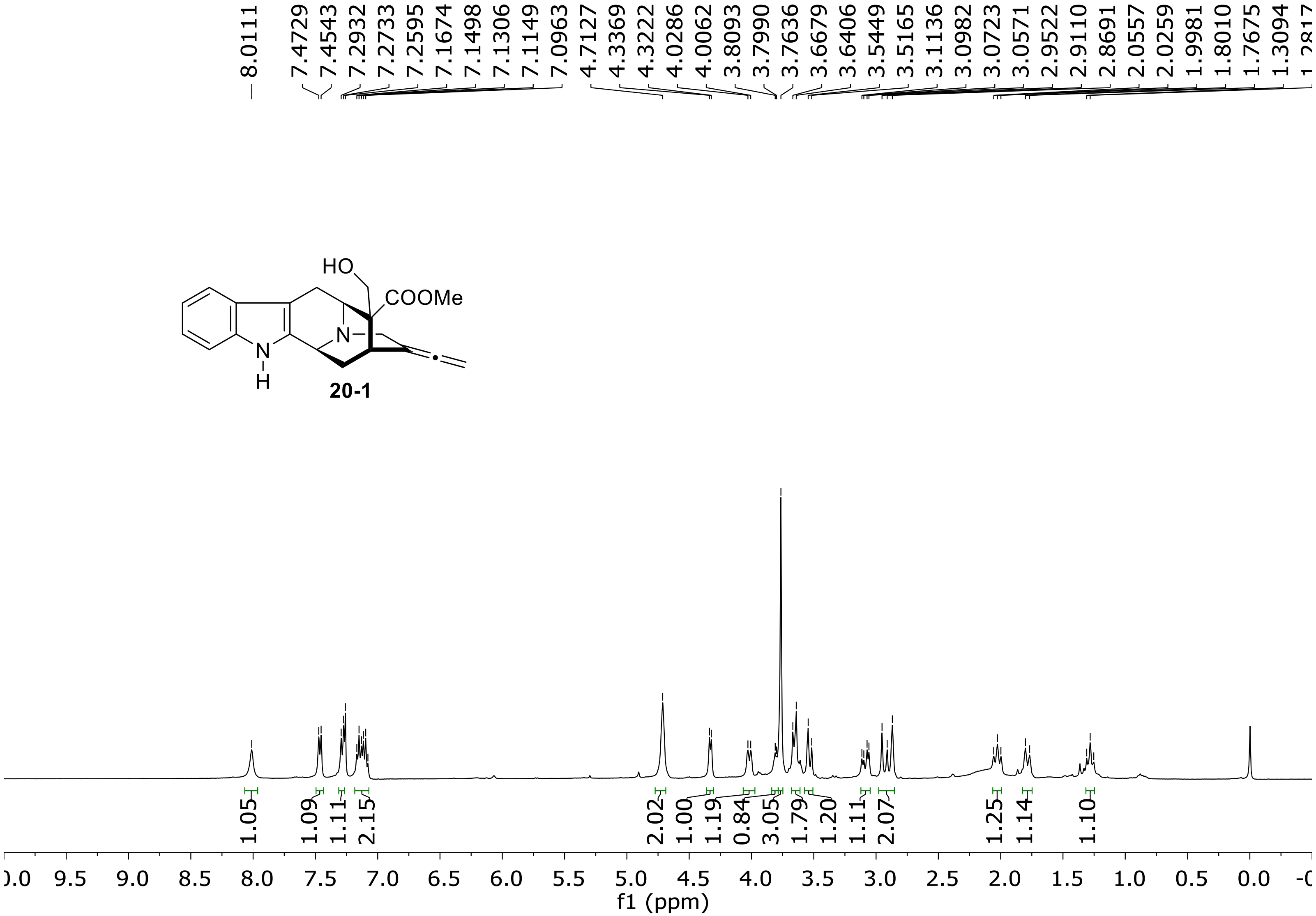
8.0111
7.4729
7.4543
7.2932
7.2733
7.2595
7.1674
7.1498
7.1306
7.1149
7.0963
4.7127
4.3369
4.3222
4.0286
4.0062
3.8093
3.7990
3.7636
3.6679
3.6406
3.5449
3.5165
3.1136
3.0982
3.0723
3.0571
2.9522
2.9110
2.8691
2.0557
2.0259
1.9981
1.8010
1.7675
1.3094
HO
COOMe
N
N
H
20-1
1.05
1.09
1.11
2.15
2.02
1.00
1.19
0.84
3.05
1.79
1.20
1.11
2.07
1.25
1.14
1.10
9.5
9.0
8.5
8.0
7.5
7.0
6.5
6.0
5.5
5.0
4.5
4.0
3.5
3.0
2.5
2.0
1.5
1.0
0.5
0.0
f1 (ppm)

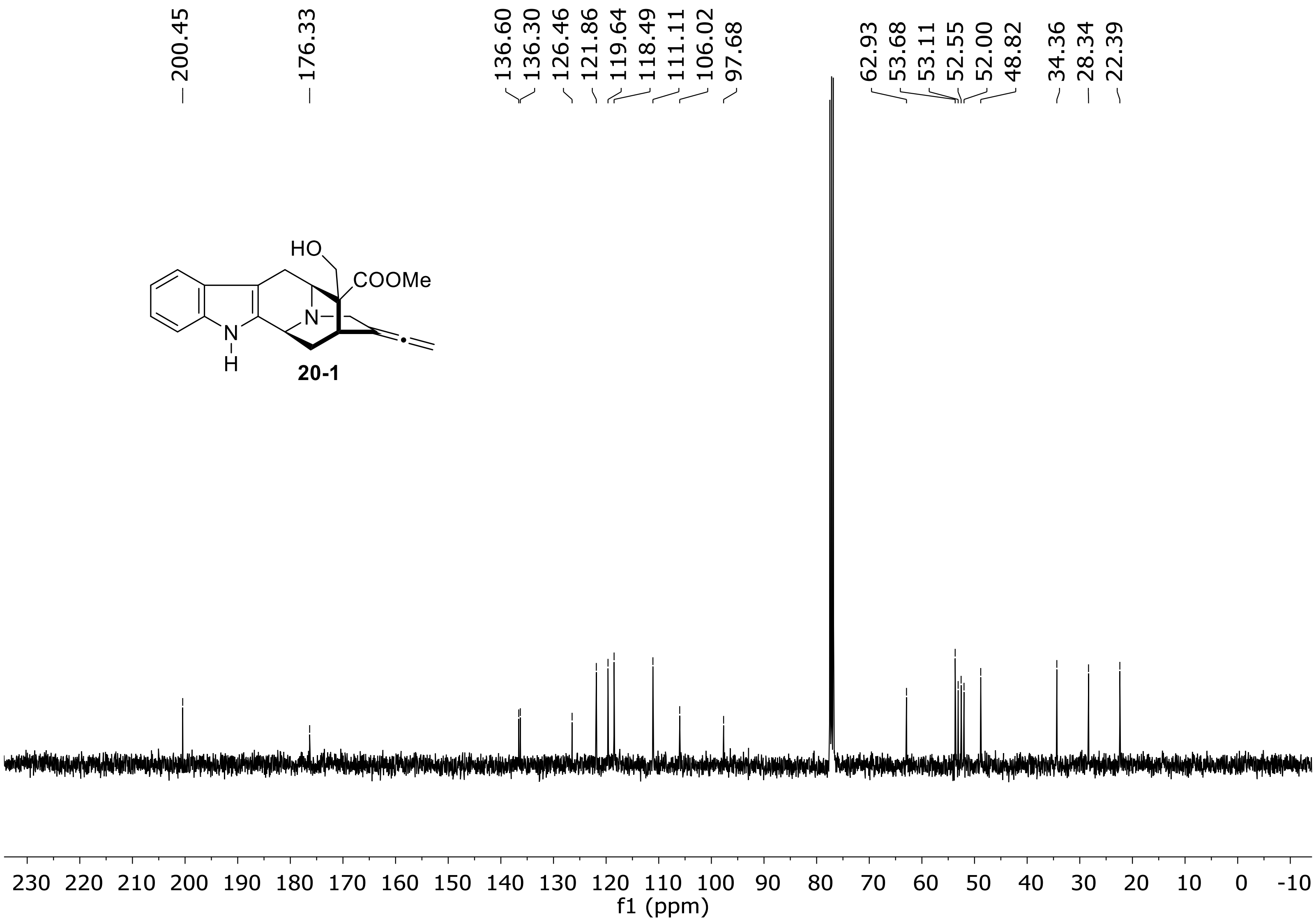
200.45
176.33
136.60
136.30
126.46
121.86
119.64
118.49
111.11
106.02
97.68
62.93
53.68
53.11
52.55
52.00
48.82
34.36
28.34
22.39
HO
COOMe
N
N
H
20-1
230 220 210 200 190 180 170 160 150 140 130 120 110 100 90 80 70 60 50 40 30 20 10 0 -10
f1 (ppm)

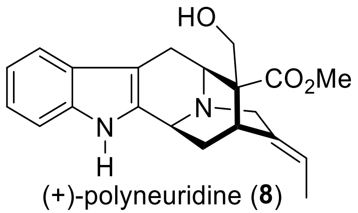

(+)-polyneuridine (**8**)

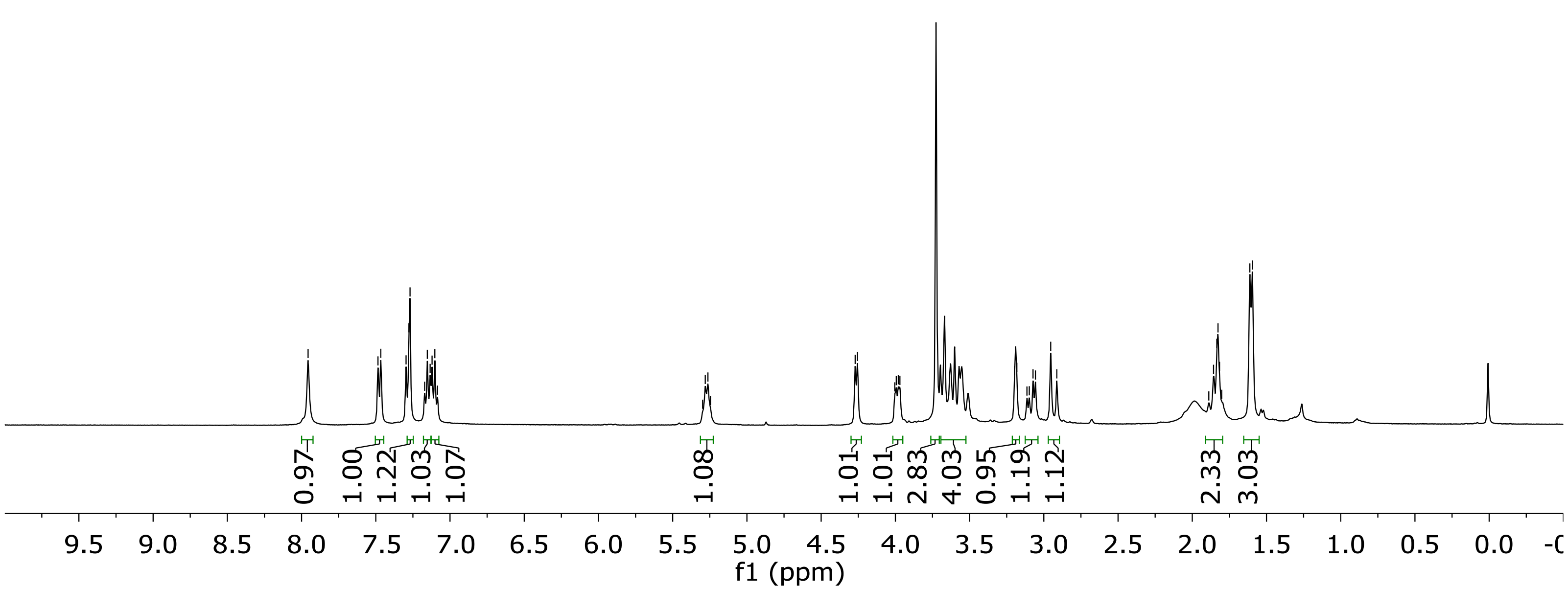

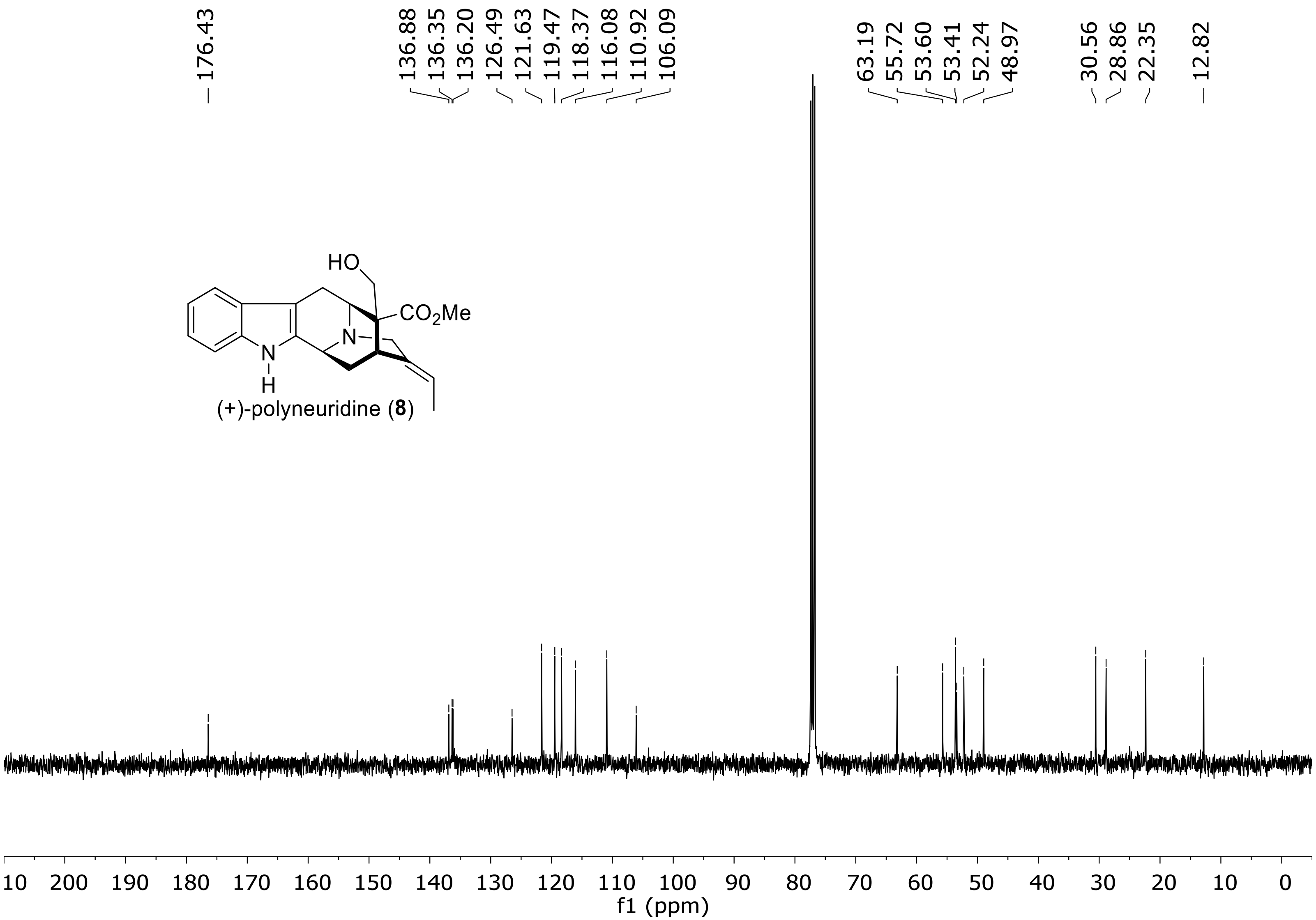
176.43
136.88
136.35
136.20
126.49
121.63
119.47
118.37
116.08
110.92
106.09
63.19
55.72
53.60
53.41
52.24
48.97
30.56
28.86
22.35
12.82
HO
CO2Me
N
N
H
(+)-polyneuridine (8)
10 200 190 180 170 160 150 140 130 120 110 100 90 80 70 60 50 40 30 20 10 0
f1 (ppm)

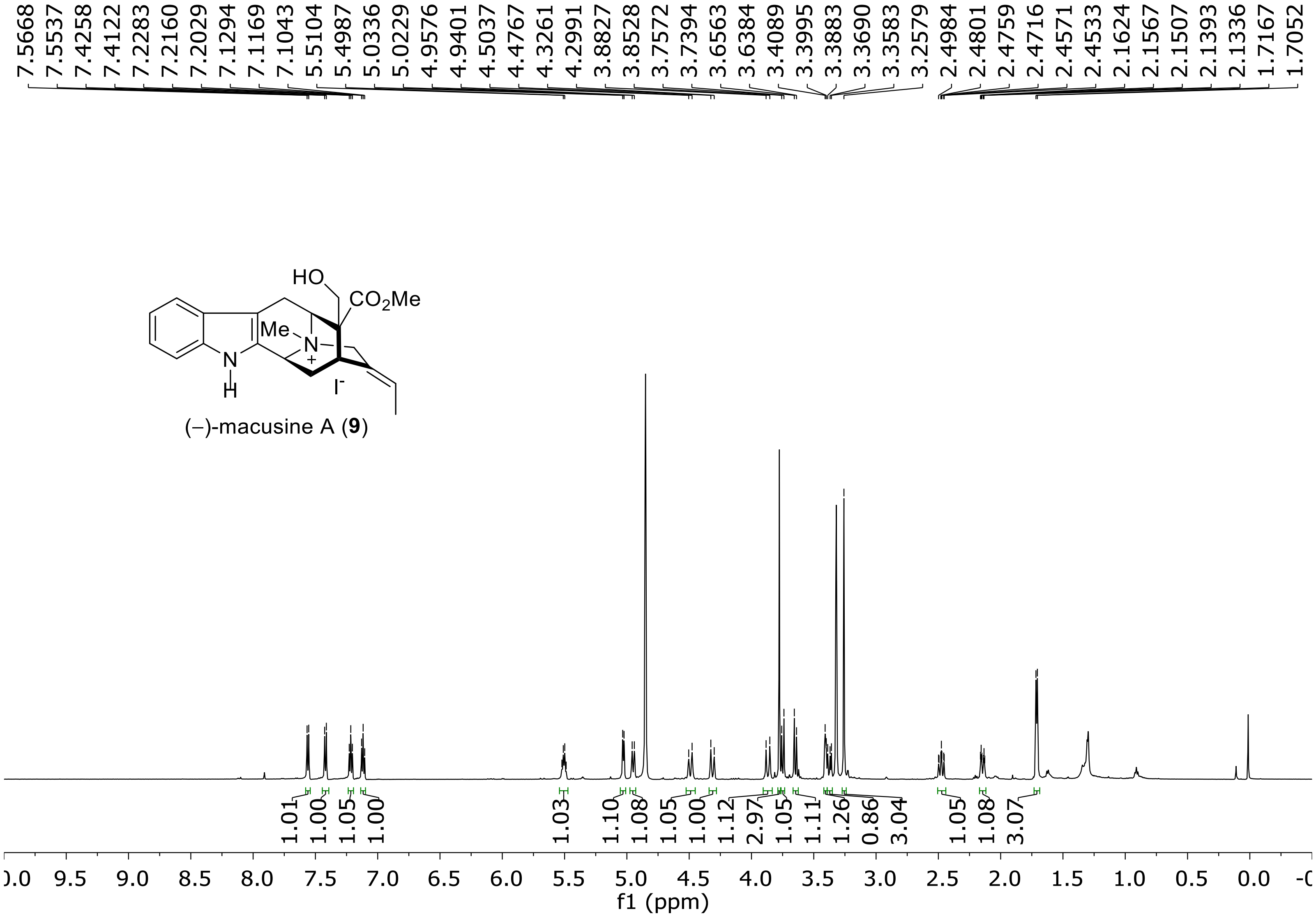
7.5668 7.5537 7.4258 7.4122 7.2283 7.2160 7.2029 7.1294 7.1169 7.1043 5.5104 5.4987 5.0336 5.0229 4.9576 4.9401 4.5037 4.4767 4.3261 4.2991 3.8827 3.8528 3.7572 3.7394 3.6563 3.6384 3.4089 3.3995 3.3883 3.3690 3.3583 3.2579 2.4984 2.4801 2.4759 2.4716 2.4571 2.4533 2.1624 2.1567 2.1507 2.1393 2.1336 1.7167 1.7052
HO
CO2Me
Me
N
+
N
H
I-
(−)-macusine A (9)
1.01 1.00 1.05 1.00 1.03 1.10 1.08 1.05 1.00 1.12 2.97 1.05 1.11 1.26 0.86 3.04 1.05 1.08 3.07
9.5 9.0 8.5 8.0 7.5 7.0 6.5 6.0 5.5 5.0 4.5 4.0 3.5 3.0 2.5 2.0 1.5 1.0 0.5 0.0
f1 (ppm)

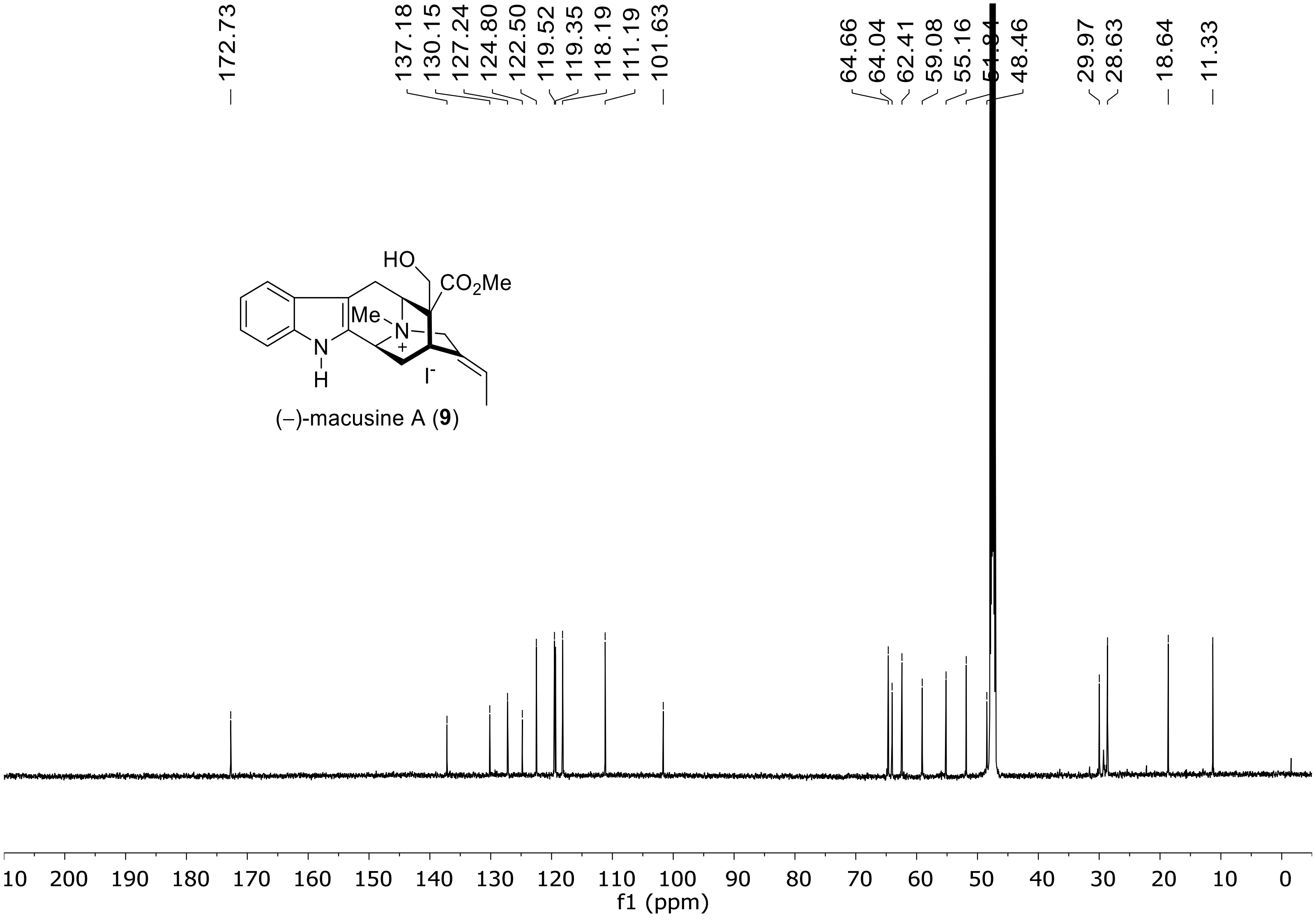
172.73
137.18
130.15
127.24
124.80
122.50
119.52
119.35
118.19
111.19
101.63
64.66
64.04
62.41
59.08
55.16
51.84
48.46
29.97
28.63
18.64
11.33
HO
CO2Me
Me
N
+
N
H
I-
(–)-macusine A (9)
10 200 190 180 170 160 150 140 130 120 110 100 90 80 70 60 50 40 30 20 10 0
f1 (ppm)

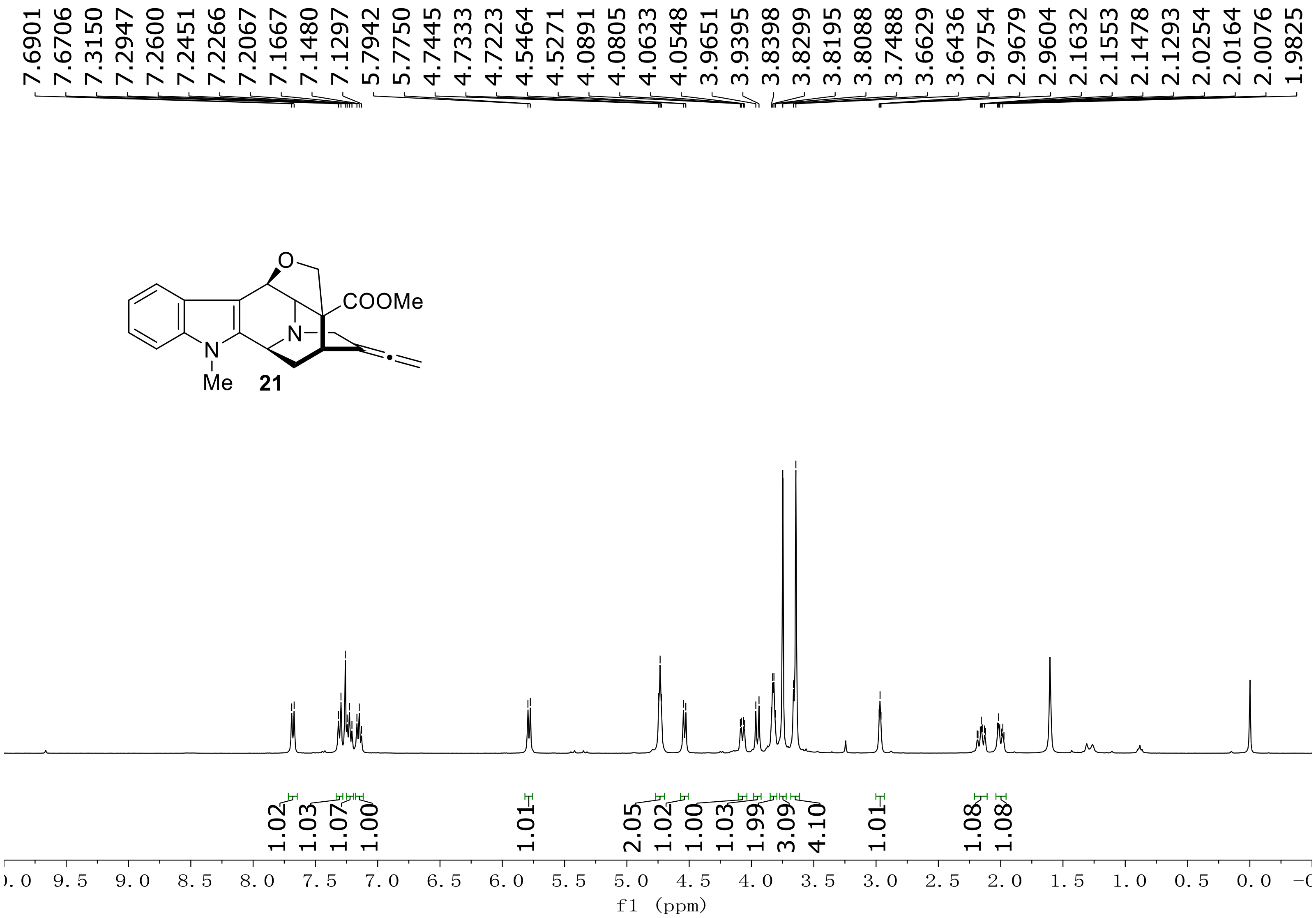

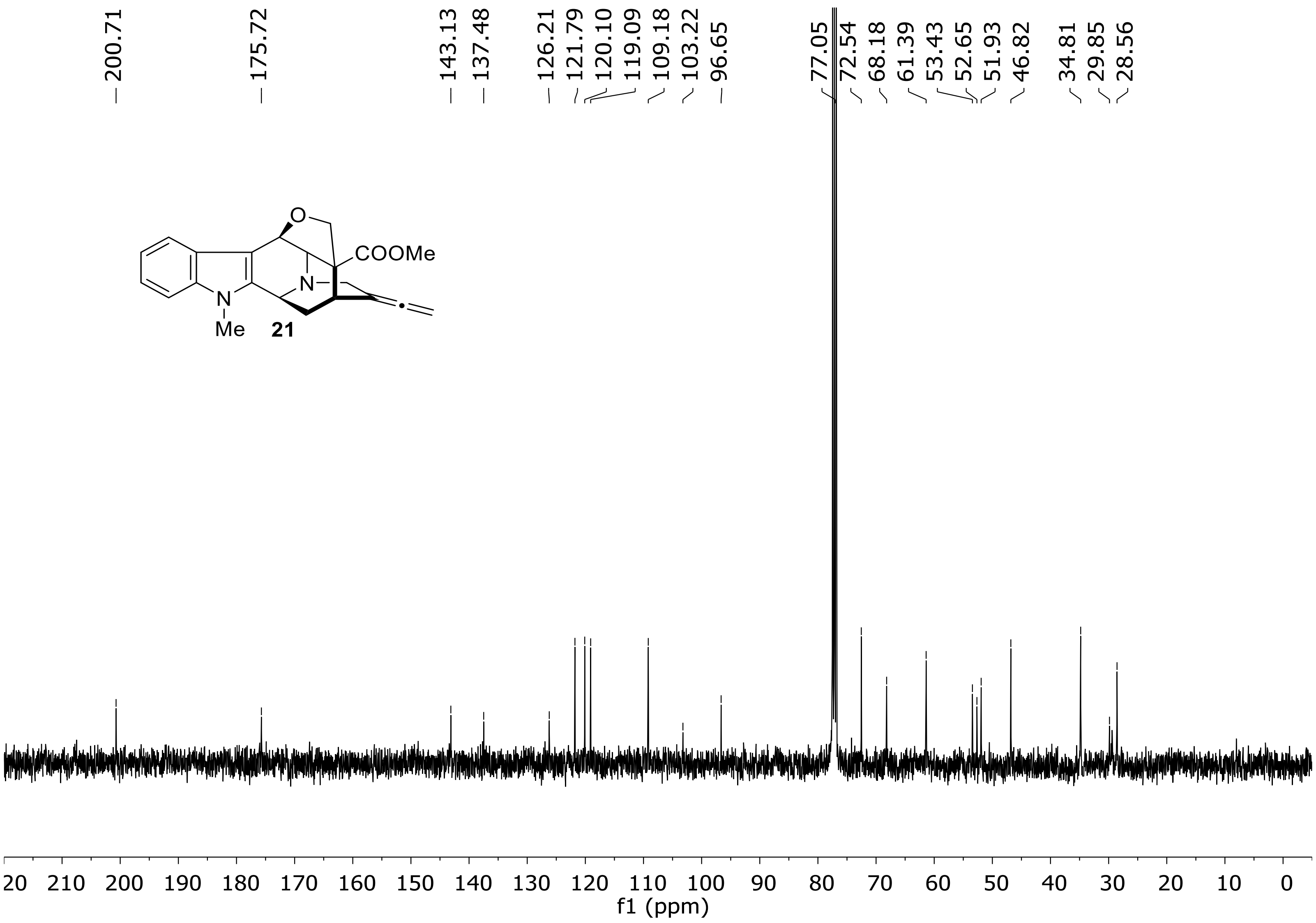
200.71
175.72
143.13
137.48
126.21
121.79
120.10
119.09
109.18
103.22
96.65
77.05
72.54
68.18
61.39
53.43
52.65
51.93
46.82
34.81
29.85
28.56
O
COOMe
N
N
Me
21
20 210 200 190 180 170 160 150 140 130 120 110 100 90 80 70 60 50 40 30 20 10 0
f1 (ppm)

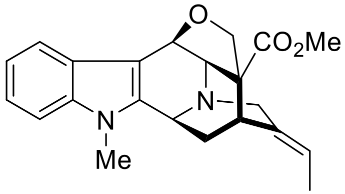

(+)-dehydrovoachalotine (**10**)

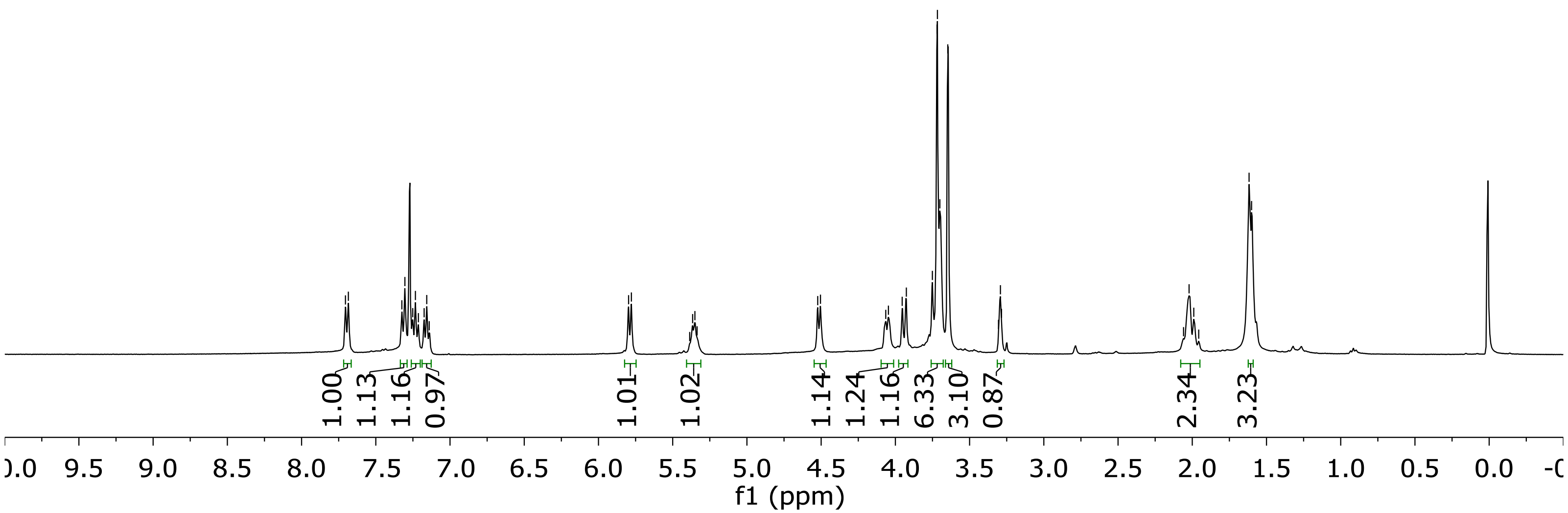

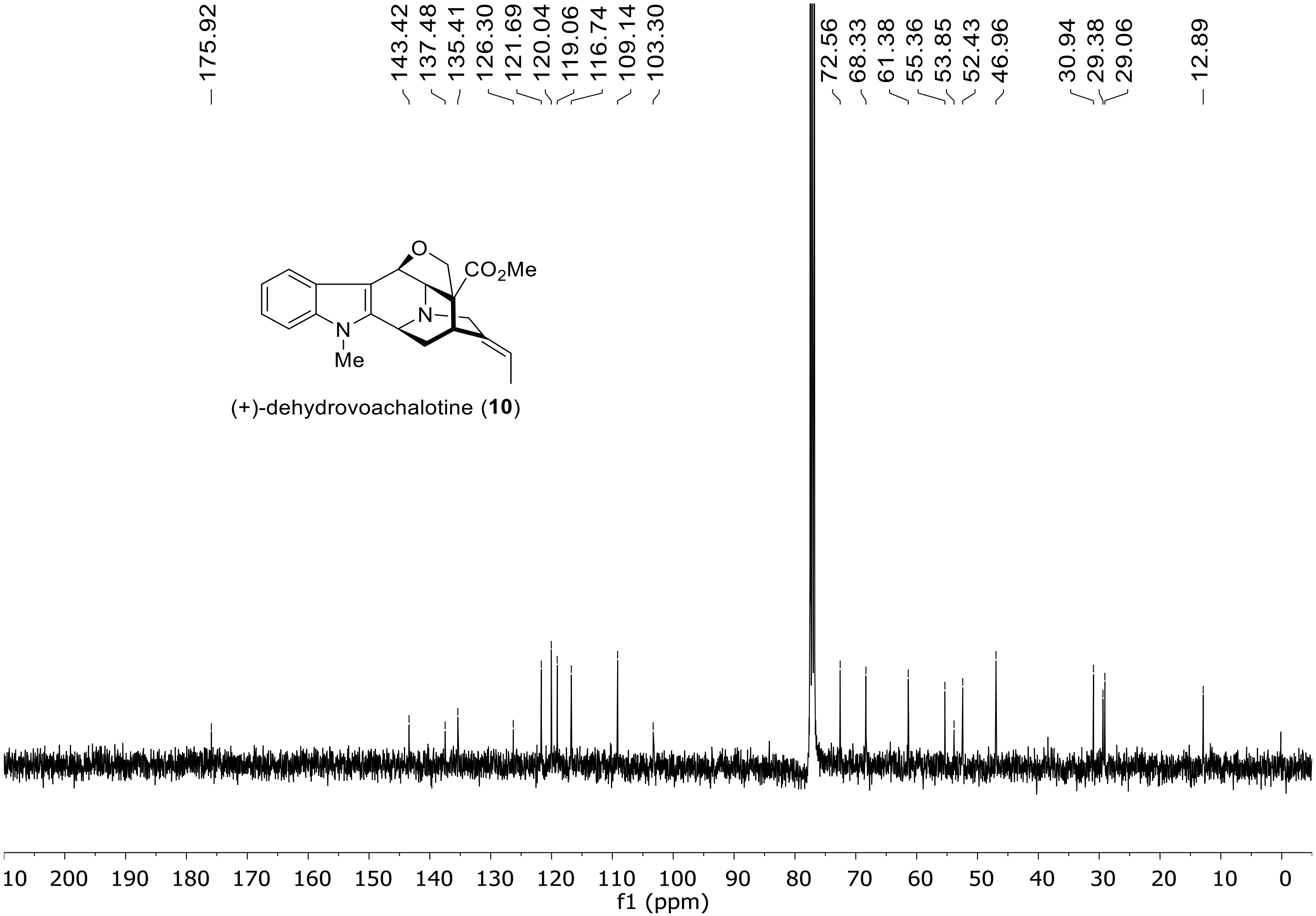
175.92
143.42
137.48
135.41
126.30
121.69
120.04
119.06
116.74
109.14
103.30
72.56
68.33
61.38
55.36
53.85
52.43
46.96
30.94
29.38
29.06
12.89
O
CO2Me
N
N
Me
(+)-dehydrovoachalotine (10)
10
200
190
180
170
160
150
140
130
120
110
100
90
80
70
60
50
40
30
20
10
0
f1 (ppm)

5. References

1. Yang, Z. et al. Asymmetric Total Synthesis of Sarpagine and Koumine Alkaloids. *Angew. Chem. Int. Ed*. **60**, 13105–13111 (2021).

2. Zhao, S., Liao, X. & Cook, J. M. Enantiospecific, Stereospecific Total Synthesis of (+)-Majvinine, (+)-10-Methoxyaffinisine, and (+)-N_a-Methylsarpagine as Well as the Total Synthesis of the Alstonia Bisindole Macralstonidine. *Org. Lett.* **4**, 687–690 (2002).

3. Okada, M. et al. Towards Structural Determination of the ComX Pheromone: Synthetic Studies on Peptides Containing Geranyltryptophan. *Biosci. Biotechnol. Biochem.* **68**, 2374–2387 (2004).

4. Deiters, A., Chen, K., Eary, C. T. & Martin, S. F. Biomimetic Entry to the Sarpagan Family of Indole Alkaloids: Total Synthesis of (+)-Geissoschizine and (+)-*N*-Methylvellosimine. *J. Am. Chem. Soc.* **125**, 4541–4550 (2003).

5. Edwankar, C. R., Edwankar, R. V., Deschamps, J. R. & Cook, J. M. Nature-Inspired Stereospecific Total Synthesis of *P*-(+)-Dispegatrine and Four Other Monomeric Sarpagine Indole Alkaloids. *Angew. Chem. Int. Ed*. **51**, 11762–11765 (2012).

6. Yu, J. et al. General Approach for the Synthesis of Sarpagine Indole Alkaloids. Enantiospecific Total Synthesis of (+)-Vellosimine, (+)-Normacusine B, (–)-Alkaloid Q_3, (–)-Panarine, (+)-N_a-Methylvellosimine, and (+)-N_a-Methyl-16-epipericyclivine. *J. Org. Chem*. **68**, 7565–7581 (2003).

7. Yin, W. et al. Enantiospecific Total Synthesis of the Important Biogenetic Intermediates along the Ajmaline Pathway, (+)-Polyneuridine and (+)-Polyneuridine Aldehyde, as well as 16-Epivellosimine and Macusine A. *J. Org. Chem*. **75**, 3339–3349 (2010).

8. Jokela, R. & Lounasmaa, M. ^{1}H- and ^{13}C-NMR Spectral Data of Five Sarpagine-type Alkaloids. *Heterocycles* **43**, 1015–1020 (1996).

9. Yu, J., Wearing, X. Z. & Cook, J. M. A General Strategy for the Synthesis of Vincamajine-Related Indole Alkaloids: Stereocontrolled Total Synthesis of (+)-Dehydrovoachalotine, (–)-Vincamajinine, and (–)-11-Methoxy-17-epivincamajine as Well as the Related Quebrachidine Diol, Vincamajine Diol, and Vincarinol. *J. Org. Chem*. **70**, 3963–3979 (2005).
