# Supplementary material for: A Bischler-Napieralski and homo-Mannich sequence enables diversified syntheses of sarpagine alkaloids and analogues
Source: Nat Commun. 2023 Sep 9;14:5560. doi: 10.1038/s41467-023-41268-9 (PMC10492809; doi:10.1038/s41467-023-41268-9)
Supplement: Supplementary file 3 — Reporting Summary [file 41467_2023_41268_MOESM3_ESM.pdf]

nature portfolio

Corresponding author(s): Min Zhang

Last updated by author(s): Aug 23, 2023

Reporting Summary

Nature Portfolio wishes to improve the reproducibility of the work that we publish. This form provides structure for consistency and transparency in reporting. For further information on Nature Portfolio policies, see our Editorial Policies and the Editorial Policy Checklist.

Statistics

For all statistical analyses, confirm that the following items are present in the figure legend, table legend, main text, or Methods section.

n/a	Confirmed	
☐	☒	The exact sample size (*n*) for each experimental group/condition, given as a discrete number and unit of measurement
☐	☒	A statement on whether measurements were taken from distinct samples or whether the same sample was measured repeatedly
☒	☐	The statistical test(s) used AND whether they are one- or two-sided<br>*Only common tests should be described solely by name; describe more complex techniques in the Methods section.*
☒	☐	A description of all covariates tested
☒	☐	A description of any assumptions or corrections, such as tests of normality and adjustment for multiple comparisons
☐	☒	A full description of the statistical parameters including central tendency (e.g. means) or other basic estimates (e.g. regression coefficient) AND variation (e.g. standard deviation) or associated estimates of uncertainty (e.g. confidence intervals)
☒	☐	For null hypothesis testing, the test statistic (e.g. *F*, *t*, *r*) with confidence intervals, effect sizes, degrees of freedom and *P* value noted<br>*Give P values as exact values whenever suitable.*
☒	☐	For Bayesian analysis, information on the choice of priors and Markov chain Monte Carlo settings
☒	☐	For hierarchical and complex designs, identification of the appropriate level for tests and full reporting of outcomes
☒	☐	Estimates of effect sizes (e.g. Cohen's *d*, Pearson's *r*), indicating how they were calculated

Our web collection on statistics for biologists contains articles on many of the points above.

Software and code

Policy information about availability of computer code

Data collection	Microplate reader (SpectraMax i3x, Molecular Devices, US); Analytical Flow cytometry (CytoFLEX, Becton Dickinson, US); Transmission electron microscopy (H-7650 100kv, Hitachi, Japan); ChemiDoc system (BioRad, Shanghai, China)
Data analysis	GraphPad Prism 9; FlowJo 7.6.1; ImageJ 1.50

For manuscripts utilizing custom algorithms or software that are central to the research but not yet described in published literature, software must be made available to editors and reviewers. We strongly encourage code deposition in a community repository (e.g. GitHub). See the Nature Portfolio guidelines for submitting code & software for further information.

Data

Policy information about availability of data

All manuscripts must include a data availability statement. This statement should provide the following information, where applicable:

- Accession codes, unique identifiers, or web links for publicly available datasets
- A description of any restrictions on data availability
- For clinical datasets or third party data, please ensure that the statement adheres to our policy

All relevant data supporting the findings of this study, including experimental procedures, compound characterizations, biological activity studies are available within the Article and its Supplementary Information. The raw data of Flow Cytometry, TEM and Western Blot had been deposited in Figshare (https://doi.org/10.6084/m9.figshare.24038868). Source data are provided with this paper.

Research involving human participants, their data, or biological material

Policy information about studies with human participants or human data. See also policy information about sex, gender (identity/presentation), and sexual orientation and race, ethnicity and racism.

Reporting on sex and gender	N/A
Reporting on race, ethnicity, or other socially relevant groupings	N/A
Population characteristics	N/A
Recruitment	N/A
Ethics oversight	N/A

Note that full information on the approval of the study protocol must also be provided in the manuscript.

Field-specific reporting

Please select the one below that is the best fit for your research. If you are not sure, read the appropriate sections before making your selection.

☒ Life sciences ☐ Behavioural & social sciences ☐ Ecological, evolutionary & environmental sciences

For a reference copy of the document with all sections, see nature.com/documents/nr-reporting-summary-flat.pdf

Life sciences study design

All studies must disclose on these points even when the disclosure is negative.

Sample size	For the assays, n >= 3 is a must to correct errors. The methodology is widely employed, for instance, doi.org/10.1016/j.ejmech.2014.03.049.
Data exclusions	No data has been excluded from the analyses presented in this manuscript.
Replication	All assays have been replicated for at least three times and replications were successful.
Randomization	For each cellular assay, cells were counted and incubated evenly into the 96-wells plates, 12-wells plates or 6-wells plates before treated with compounds.
Blinding	Blinding tests are usually employed in the clinical trials to eliminate subjective factors. For the cell assays, all data were acquired by instruments instead of human observation and record. So it is not applicable here.

Reporting for specific materials, systems and methods

We require information from authors about some types of materials, experimental systems and methods used in many studies. Here, indicate whether each material, system or method listed is relevant to your study. If you are not sure if a list item applies to your research, read the appropriate section before selecting a response.

Materials & experimental systems

n/a	Involved in the study	
☐	☒	Antibodies
☐	☒	Eukaryotic cell lines
☒	☐	Palaeontology and archaeology
☒	☐	Animals and other organisms
☒	☐	Clinical data
☒	☐	Dual use research of concern
☒	☐	Plants

Methods

n/a	Involved in the study	
☒	☐	ChIP-seq
☐	☒	Flow cytometry
☒	☐	MRI-based neuroimaging

Antibodies

Antibodies used	GPX4 Polyclonal antibody (Proteintech, Cat No: 30388-1-AP); SLC7A11/xCT Rabbit pAb (ABclonal, Cat No: A13685); GAPDH (Wanlei, Cat No: WL01114); HRP conjugated goat anti rabbit IgG (H+L) (Wanlei, Cat No: WLA023)

Validation	The following antibodies were quality-checked and validated based on the information provided on the manufacturers' websites: For For GPX4 Monoclonal antibody: https://www.ptgcn.com/products/GPX4-Antibody-30388-1-AP.htm; Species: Human, Mouse, Rat; Application: FC, WB, ELISA<br>For SLC7A11/xCT Rabbit mAb: https://abclonal.com.cn/catalog/A13685; Species: Human, Mouse, Rat; Application: IF, IHC, WB, IP.<br>For anti-GAPDH: https://shop.wanleibio.cn/Product/ProductIntroduce?act=Standard&pid=1455#detail; Species: Human, Mouse, Rat; Application: IF, IHC, WB.<br>For HRP conjugated goat anti rabbit IgG (H+L): https://shop.wanleibio.cn/Product/ProductIntroduce?pid=277; Species: Rabbit Application: ELISA, IHC, WB.

Eukaryotic cell lines

Policy information about cell lines and Sex and Gender in Research

Cell line source(s)	All cell lines were purchased from the National Collection of Authenticated Cell Cultures, China.<br>MIA PaCa-2 (Serial number SCSP-568)<br>MDA-MB-231 (Serial number TCHu227)<br>Hela (Serial number TCHu187)<br>A549 (Serial number TCHu150)<br>MCF-7 (Serial number TCHu 74)<br>CT26 (Serial number TCM37)<br>HCT 116 (Serial number SCSP-5076)<br>A375 (Serial number SCSP-533)<br>BEAS-2B (Serial number SCSP-5067)
Authentication	All the cell lines were commercially available and have not been authenticated after receiving them.
Mycoplasma contamination	All cell lines were tested negative for mycoplasma contamination.
Commonly misidentified lines (See ICLAC register)	No commonly misidentified cell lines were used.

Flow Cytometry

Plots

Confirm that:

☒ The axis labels state the marker and fluorochrome used (e.g. CD4-FITC).

☒ The axis scales are clearly visible. Include numbers along axes only for bottom left plot of group (a 'group' is an analysis of identical markers).

☒ All plots are contour plots with outliers or pseudocolor plots.

☒ A numerical value for number of cells or percentage (with statistics) is provided.

Methodology

Sample preparation	Cells were harvested and washed two times with PBS followed by respectively incubating with PBS containing AnnexinV/PI, BODIPY-C11, H2DCFDA.
Instrument	Analytical Flow cytometry (CytoFLEX)
Software	FlowJo 7.6.1
Cell population abundance	10,000 cells were analyzed for each sample
Gating strategy	The starting cell population gating by Forward Scatter and Side Scatter was used to make sure doublet exclusion. Only single cell was used for analysis.

☒ Tick this box to confirm that a figure exemplifying the gating strategy is provided in the Supplementary Information.
